# Photoredox Nucleophilic (Radio)fluorination of Alkoxyamines

**DOI:** 10.1021/jacs.4c02474

**Published:** 2024-04-23

**Authors:** Sebastiano Ortalli, Joseph Ford, Andrés A. Trabanco, Matthew Tredwell, Véronique Gouverneur

**Affiliations:** † Department of Chemistry, Chemistry Research Laboratory, University of Oxford, Mansfield Road, Oxford OX1 3TA, United Kingdom; ∥ Global Discovery Chemistry, Therapeutics Discovery, Johnson & Johnson Innovative Medicine, Janssen-Cilag, S.A., E-45007 Toledo, Spain; # Wales Research and Diagnostic PET Imaging Centre, Cardiff University, University Hospital of Wales, Heath Park, Cardiff CF14 4XN, United Kingdom; ¶ School of Chemistry, Cardiff University, Main Building, Park Place, Cardiff CF10 3AT, United Kingdom

## Abstract

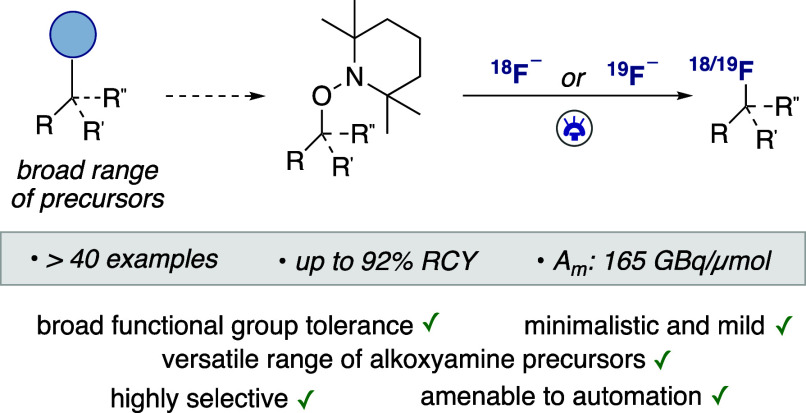

Herein, we report a photoredox nucleophilic (radio)fluorination using TEMPO-derived alkoxyamines, a class of substrates accessible in a single step from a diversity of readily available carboxylic acids, halides, alkenes, alcohols, aldehydes, boron reagents, and C–H bonds. This mild and versatile one-electron pathway affords radiolabeled aliphatic fluorides that are typically inaccessible applying conventional nucleophilic substitution technologies due to insufficient reactivity and competitive elimination. Automation of this photoredox process is also demonstrated with a user-friendly and commercially available photoredox flow reactor and radiosynthetic platform, therefore expediting access to labeled aliphatic fluorides in high molar activity (*A*_*m*_) for (pre)clinical evaluation.

Positron emission tomography (PET) stands out among other imaging modalities as this noninvasive and highly sensitive technique allows the interrogation of biological processes *in vivo* and in real time.^1,2^ Moreover, the recent invention of total-body PET offers new opportunities by capturing images of patients’ entire bodies and the use of less radioactivity.^3^ The decay profile of ^18^F is advantageous (97% β^+^) and its half-life of 109.7 min well suited to the synthesis of complex radiopharmaceuticals.^4^ Transportation to remote imaging centers is also possible enabling multipatient scanning for the acquisition of high-resolution images. ^18^F therefore predominates in the clinic, as best exemplified by the leading role of [^18^F]fluorodeoxyglucose. An additional incentive for the use of ^18^F in PET ligands for drug development campaigns stems from the prominence of ^19^F in pharmaceuticals.^5^ Nevertheless, the bottleneck for PET undeniably remains the development of robust radiosynthetic methodologies, ideally amenable to automation, for the mild and selective incorporation of ^18^F into radiotracers.^6^

Access to secondary and tertiary ^18^F-labeled alkyl fluorides still poses an unmet challenge in ^18^F-radiochemistry. Two-electron pathways with displacement of leaving groups by [^18^F]fluoride are ineffective as they require extensive synthetic effort to secure complex prefunctionalized precursors, and harsh radiofluorination conditions (>100 °C).^6a,7^ Poor or no reactivity is common alongside the formation of elimination byproducts. Methods exploiting one-electron pathways have been considered to improve this state of play (Figure 1).^8^ Doyle and co-workers reported the radiofluorination of *N-*hydroxyphthalimide esters,^8a^ a class of substrates accessible from carboxylic acids. This methodology, operating via a radical-polar crossover (RPCO) mechanism, led to three ^18^F-labeled products derived from tertiary alkyl or oxocarbenium intermediates. *A*_*m*_ reached 37 GBq/μmol. An alternative protocol by Groves and co-workers features a manganese complex serving as ^18^F-fluorine atom transfer agent with iodosobenzene as the stoichiometric oxidant.^8b−8d^ These conditions were successfully applied to carboxylic acid substrates.^8b^ An elegant C–H activation protocol was also investigated but, for selected substrates, this radiochemistry suffers from site-selectivity and purification issues.^8c,8d^ Our goal was to offer radiochemists a novel versatile method to prepare alkyl ^18^F-fluorides using a broader range of both starting materials and carbocations. We selected TEMPO-derived alkoxyamines that are amenable to photoredox-induced functionalization with various nucleophiles.^9^ Nucleophilic fluorination has however not been investigated. Mechanistically, this chemistry subtly differs from RPCO carbocation generation from *e.g*. *N-*hydroxyphthalimide esters since the mesolytic cleavage of alkoxyamines bypasses the formation of reactive C-centered radicals.^8a,9^ Synthetically, the TEMPO group also stands out as it is easily installed in a single step from a remarkable range of substrate classes,^10^ including carboxylic acids,^10a^ halides,^10b^ alkenes,^10c^ alcohols,^10d^ aldehydes,^10e^ boron reagents,^10f^ thiols,^10g^ and C–H bonds.^10h^ We noted that the fluorination of a TEMPO-substituted substrate was reported using Selectfluor serving both as oxidant and fluorinating agent.^11^ This reaction was not retained for labeling because the synthesis of [^18^F]Selectfluor requires [^18^F]F_2_, a reagent not available in most radiochemistry facilities and leading to radiolabeled products in low *A*_*m*_.^6d,7b^ Our aim was therefore to develop a protocol using a fluoride source for extension to radiochemistry. Herein, we report such a protocol with the first redox-neutral, light-mediated nucleophilic fluorination of alkoxyamines, and demonstrate suitability for ^18^F-labeling and applications in PET imaging.

**Figure 1:**
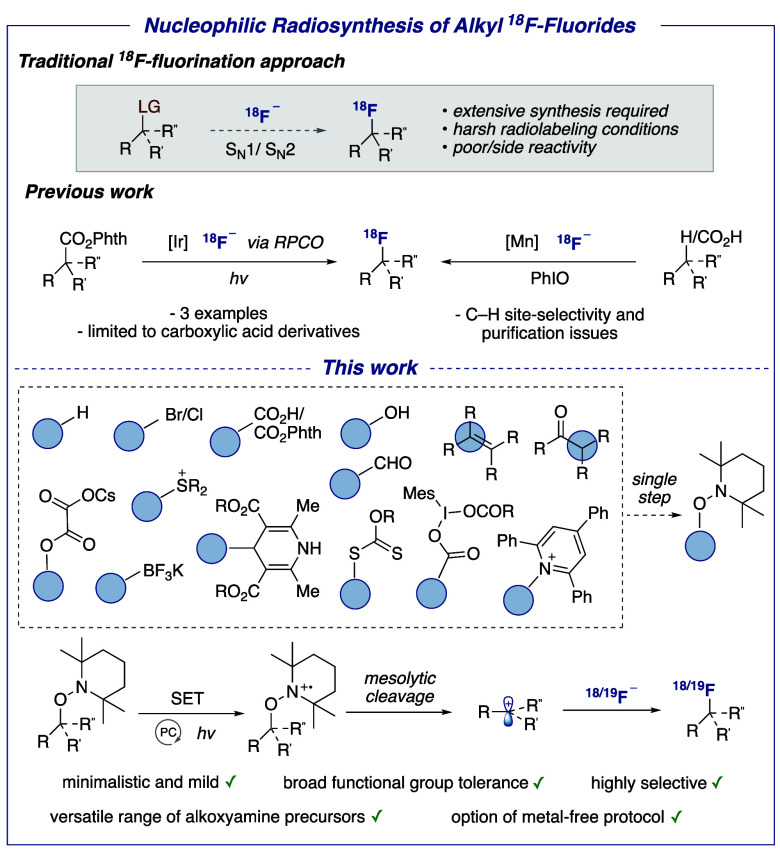
Prior art and this work.

We began our investigations with secondary benzylic alkoxyamine **1** (Table 1), a model substrate prone to elimination. Preliminary investigation demonstrated that the fluorination of **1** was successful in the presence of NEt_3_·3HF and photocatalyst **3a** (Table 1, entry 1). This chemistry was not deemed ideal for ^18^F-labeling due to possible loss of activity in the form of gaseous [^18^F]HF (bp 19.5 °C) and the requirement for cumbersome setups incompatible with common automated platforms.^12^ Further investigation therefore focused on KF and CsF as the fluoride source (Table 1, entries 2 and 3), well aware that these reagents are also Bro̷nsted bases that may induce competing elimination. Upon extensive screening of reaction conditions, hexafluoroisopropanol (HFIP) was found crucial for reactivity by serving both as proton source and solubilizing agent.^13,14^ Benzylic fluoride **2** was formed in 20% and 59% yield with KF and CsF respectively, alongside alkene resulting from elimination and HFIP-trapped ether byproduct.^14^ This result served as entry point to radiochemistry (Table 1, entries 4–12).

**Table 1:**
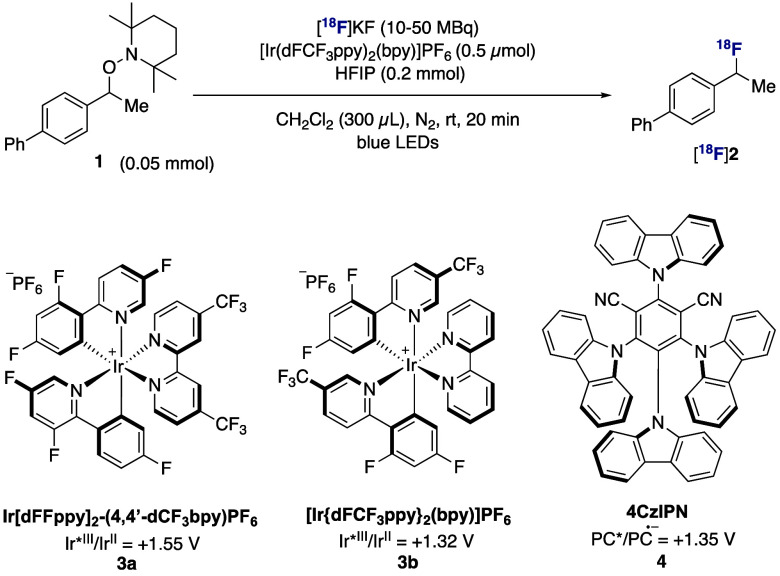
Reaction Optimization*

Entry	Deviation from Standard Conditions	Yield^a^ (%)	RCY (%)
1	with NEt_3_·3HF (2 equiv), **3a** (1 mol%) in CH_2_Cl_2_ (0.2 M) for 16 h	84%	
2	with KF (2 equiv), HFIP (10 equiv), **3a** (1 mol%) in CH_2_Cl_2_ (0.05 M) for 16 h	20%	
3	with CsF (2 equiv), HFIP (10 equiv), **3a** (1 mol%) in CH_2_Cl_2_ (0.05 M) for 16 h	59%	
4	none		92 ± 4_*n*=3_
5	no photocatalyst		0_*n*=1_
6	no irradiation		0_*n*=1_
7	no HFIP		11_*n*=1_
8	in THF		51_*n*=1_
9	with 4CzIPN		90_*n*=1_
10	2 min reaction time		71_*n*=1_
11	0.005 mmol substrate loading		66_*n*=1_
12	no N_2_ degassing		82_*n*=1_

* [^18^F]KF was prepared with a K_2_CO_3_ (0.011 mmol)/K_222_ (0.020 mmol) elution protocol. RCY: radiochemical yield determined by radioHPLC. Redox potentials given versus SCE.^15^

a Determined by quantitative ^19^F NMR.

For radiofluorination, the reaction volume was decreased and the stoichiometry of HFIP relative to **1** was reduced to 4 equiv with no detrimental effect. Photocatalyst **3b** was preferred over **3a** as it was equally effective and is a milder oxidant. The radiofluorination of **1** proceeded in 92% radiochemical yield (RCY) using [^18^F]KF/K_222_ with **3b** when the reaction mixture was irradiated with visible light in dichloromethane solvent at room temperature over 20 min (Table 1, entry 4). Reactivity was abolished in the absence of **3b** (Table 1, entry 5) or irradiation (Table 1, entry 6). While the reaction still proceeds without HFIP, this modification was detrimental (Table 1, entry 7). Alternative solvents such as THF were less effective (Table 1, entry 8).^14^ The radiofluorination was successful with organocatalyst 4CzIPN (**4**) in place of iridium-based **3b** (Table 1, entry 9), giving the option of a metal-free protocol. Photocatalyst **3b** was however selected for further studies as its ionic nature facilitates purification (e.g., cartridge filtration) of radiolabeled products. The reaction gave [^18^F]**2** in 71% RCY after just 2 min of irradiation (Table 1, entry 10), tolerated lower substrate loading (Table 1, entry 11), and proceeded without N_2_ degassing (Table 1, entry 12). With the optimized conditions in hand, a robustness screen was carried out to evaluate the compatibility of the protocol with functionalities commonly encountered in medicinal chemistry.^14,16^ The results of this study boded well for broad functional group tolerance,^14^ and encouraged further investigation on the scope of this reaction (Scheme 1).

**Scheme 1:**
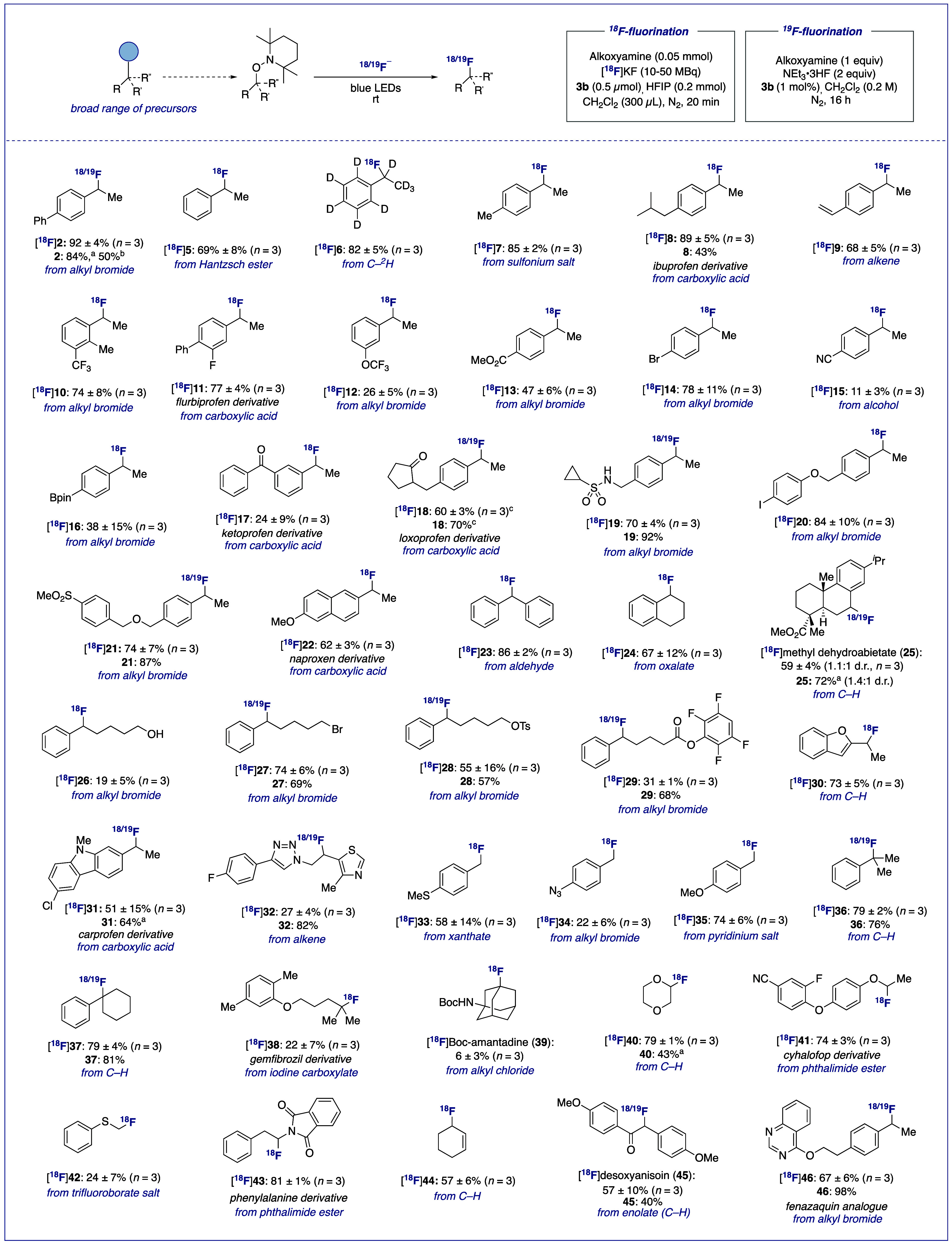
Substrate Scope Radiochemical yields determined by radioHPLC. Precursors for the synthesis of TEMPO-derived substrates are specified in blue below products. Determined by quantitative ^19^F NMR. ^19^F-Fluorination conditions: **1** (0.5 mmol, 1 equiv), CsF (2 equiv), HFIP (10 equiv), **3a** (1 mol %), CH_2_Cl_2_ (0.05 M), blue LEDs, N_2_, rt, 16 h. Unresolved diastereomers.

Conveniently, all TEMPO substrates were accessed in one step from a multitude of precursors such as halide, carboxylic acid, alkene, phthalimide ester, alcohol, aldehyde, oxalate, xanthate ester, hypervalent iodine compound, trifluoroborate salt, Hantzsch ester, ketone, amine-derived pyridinium salt, sulfonium salt, or C–H bonds.^14^ Numerous functional groups were tolerated including ether (e.g., [^18^F]**20**), ester (e.g., [^18^F]**13**), ketone (e.g., [^18^F]**17**), nitrile (e.g., [^18^F]**15**), carbamate ([^18^F]**39**), sulfonamide ([^18^F]**19**) and sulfone ([^18^F]**21**). A range of secondary benzylic fluorides ([^18^F]**2**, [^18^F]**5**–**32**, [^18^F]**45**, [^18^F]**46**) were accessed with RCYs ranging from 11% to 92%. Notably, molecules containing hydridic benzylic (e.g., [^18^F]**8**) as well as tertiary aliphatic C–H bonds, including biologically active methyl dehydroabietate^17^ ([^18^F]**25**) and Boc-protected amantadine ([^18^F]**39**), exclusively afforded the desired products with no issues of site-selectivity. This radiofluorination protocol displayed tolerance toward functional groups prone to oxidation ([^18^F]**26**, [^18^F]**33**) and several handles for cross-couplings including aryl chloride ([^18^F]**31**), bromide ([^18^F]**14**), iodide ([^18^F]**20**), as well as an azide ([^18^F]**34**) and pinacol boronic ester group ([^18^F]**16**). A styrene derivative, known to act as a radical trap under analogous one-electron transformations,^18^ afforded the desired product ([^18^F]**9**) in excellent RCY. This example illustrates how the mechanistic diversion offered by the mesolytic cleavage of alkoxyamines relative to RPCO serves us well in terms of functional group compatibility. A valuable tetrafluorophenyl (TFP) active ester, routinely employed in radiochemistry for conjugation,^19^ was also competent providing [^18^F]**29** in 31% RCY. No competitive displacement by [^18^F]fluoride was observed for substrates incorporating electrophilic groups such as a primary alkyl bromide ([^18^F]**27**) and tosylate ([^18^F]**28**).^14^ Such versatility demonstrates the orthogonality of this transformation with respect to traditional two-electron pathways that operate under forceful reaction conditions. Medicinally relevant heterocycles such as benzofuran ([^18^F]**30**), carprofen-derived carbazole [^18^F]**31**, thiazole and triazole ([^18^F]**32**), quinazoline ([^18^F]**46**), and dioxane ([^18^F]**40**) were tolerated. Primary benzylic fluorides were also within reach ([^18^F]**33**–**35**), as well as challenging tertiary alkyl fluorides ([^18^F]**36**–**39**). Radiofluorination at the α-heteroatom position is also feasible, providing access to medicinally relevant α-fluorinated ethers and thioethers,^20^ as exemplified with dioxane-derived [^18^F]**40**, cyhalofop derivative [^18^F]**41**, and α-fluoro thioether [^18^F]**42**. In addition, ^18^F was successfully introduced at the α-amino position of a phenylalanine derivative in excellent RCY ([^18^F]**43**). Radiolabeling of an allylic substrate was efficient ([^18^F]**44**), as well as an α-fluoro carbonyl compound, providing immunosuppressant [^18^F]desoxyanisoin ([^18^F]**45**) in 57% RCY.^21^ Lastly, an analogue of mitochondrial complex 1 (MC-I) inhibitor fenazaquin ([^18^F]**46**) could be radiofluorinated in very good yield, offering an orthogonal labeling strategy to that previously reported.^22^ In summary, this technology is best applied to precursors leading to sufficiently stabilized carbocationic intermediates. As expected, unactivated primary and secondary aliphatic substrates were unreactive.^14^

In line with previous mechanistic scenarios reported for reactions other than fluorination,^9^ we propose that upon irradiation with blue light, the excited state of photocatalyst **3b** (Ir^*III^/Ir^II^ = +1.32 V vs SCE)^15b^ can undergo single-electron transfer (SET) with the alkoxyamine substrate (*E*_ox_ ≈ 1.1 V vs SCE).^9^ Mesolytic cleavage of the resulting radical cation furnishes a carbocation, which is trapped by fluoride, yielding the desired (radio)fluorinated product. Concurrently, reduction of the TEMPO radical by the photocatalyst, promoted by the presence of HFIP as the proton source,^9^ regenerates the photocatalyst ground state.^14^

The method is amenable to a one-pot protocol bypassing time-consuming purification of the alkoxyamine precursor (Scheme 2). When aliquots of the crude reaction mixture containing alkyl bromide-derived **1** were submitted to the standard radiofluorination conditions, [^18^F]**2** was formed with no drop in RCY.^14^ Notably, when the radiofluorination of alkyl bromide precursor **1a** was attempted under classical two-electron conditions ([^18^F]KF/K_222_ in MeCN), no desired radiolabeled product was observed at temperatures up to 100 °C.^14^

**Scheme 2:**
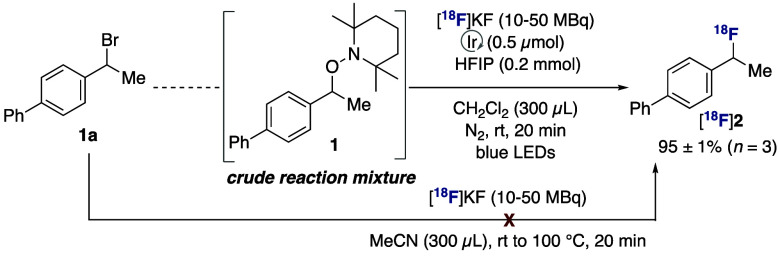
One-Pot Radiofluorination [^18^F]KF prepared with a K_2_CO_3_ (0.011 mmol)/K_222_ (0.020 mmol) elution protocol. Radiochemical yields determined by radioHPLC. Crude reaction mixture (**1**) was filtered and used in aliquots.^14^

For (pre)clinical applications, scale-up of the reaction is crucial alongside automation of all necessary steps for radiotracer production on a commercial radiosynthesis platform (Scheme 3). Such development enhances safety, reproducibility, and simplifies the quality control process. Despite the growing interest in photoredox catalysis,^23^ translation of these powerful synthetic strategies to radiochemistry has been slow.^24^ To the best of our knowledge, a single ^18^F-photoradiochemical reaction has been performed on an automated radiosynthesis platform.^25^ This process required a bespoke 3D-printed reactor, which limits general adoption by other practitioners. With these challenges in mind, a commercially available photoflow device was selected due to operational simplicity and reliable irradiation of the reaction mixture for maximum reproducibility.^14^ This device was combined with a readily available photoreactor equipped with a blue LED light, and a TRASIS AllinOne radiosynthesizer (Scheme 3). With this setup, an automated program enabled the radiosynthesis of [^18^F]**2** in an activity yield (AY) of 2.6 ± 0.6 GBq (non-decay corrected, *n* = 2) from 10 GBq starting activity, high molar activity (*A*_*m*_: 165 ± 54 GBq/μmol (*n* = 2)), and radiochemical purity (>99%).

**Scheme 3:**
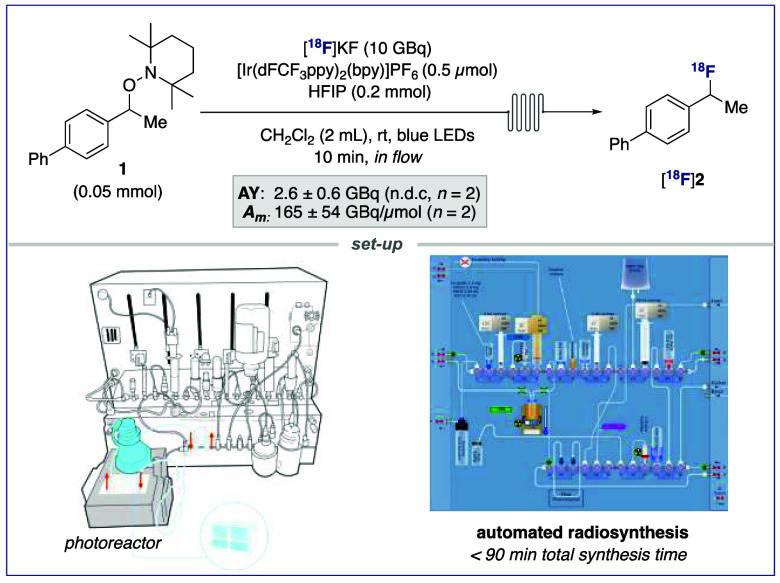
Automated Radiofluorination AY (n.d.c.) = activity yield, non-decay corrected; *A*_*m*_ = molar activity.

In conclusion, we have developed a novel photoredox-mediated fluorination of TEMPO-derived alkoxyamines that was extended to radiolabeling with [^18^F]KF. The method permits the synthesis of alkyl ^18^F-fluorides derived from stabilized carbocations, including biorelevant targets. This minimalistic technology stands out for its operational simplicity, mildness, compatibility with sensitive functional groups, and orthogonality to conventional two-electron pathways. Scalability and translation to automated radiosynthesis were implemented on a user-friendly and commercial platform furnishing radiolabeled products in good AY, *A*_*m*_, and radiochemical purity. This protocol can be immediately adopted without the need for building bespoke 3D-printed reactors. Combined with the striking versatility of precursors available for the synthesis of TEMPO-derived substrates, we expect broad interest from radiochemists applying PET to interrogate biological process *in vivo* or to develop diagnostics and pharmaceutical drugs.

## Supporting Information Available

Preparation of starting materials, labeling precursors and reference materials; radiofluorination methods; (radio)HPLC traces; NMR spectra (PDF)
