# Supplementary material for: Photoredox Nucleophilic (Radio)fluorination of Alkoxyamines
Source: J Am Chem Soc. 2024 Apr 23;146(17):11599–604. doi: 10.1021/jacs.4c02474 (PMC11066844; doi:10.1021/jacs.4c02474)

Supporting Information for

Photoredox Nucleophilic (Radio)fluorination of Alkoxyamines

Sebastiano Ortalli[†], Joseph Ford[†], Andrés A. Trabanco[‖], Matthew Tredwell[#,¶] and Véronique Gouverneur[†,*]

[†] Department of Chemistry, Chemistry Research Laboratory, University of Oxford, Mansfield Road, Oxford, OX1 3TA, U.K.

[‖] Discovery Chemistry, Janssen Research and Development, Janssen-Cilag, S.A., E-45007, Toledo, Spain.

[#] Wales Research and Diagnostic PET Imaging Centre, Cardiff University, University Hospital of Wales, Heath Park, Cardiff, CF14 4XN, U.K.

[¶] School of Chemistry, Cardiff University, Main Building, Park Place, Cardiff, CF10 3AT, U.K.

[*]Correspondence should be addressed to veronique.gouverneur@chem.ox.ac.uk

Table of contents

General information……….…………………..……………………………..................s3

Non-radioactive experiments………………….…………………………….....................s4

Optimization of the reaction conditions……………………………………………........s4

Time course experiment…………………………………………………………......s10

Proposed mechanism………………………………………………………………...s13

Synthesis of starting materials…………………………......…...………………........s14

Synthesis of reference compounds ……………….…………………………......……….s56

Radiochemistry…………………………….……………………………......................s81

General experimental details……...……...………………………………...……..……s81

Optimization of the reaction conditions………………………………………………...s83

Time course experiment…………………………………………………………......s87

Robustness screen……………………………………………………………….…......s89

Radiofluorination of crude reaction mixture……………………………………………...s92

Alternative [^{18}F]KF elution and drying conditions…………………….…..……...…………s94

Automated radiosynthesis………………….………………………………………….s95

Radiotrace overlays and radiochemical yields…………...…………...……...……...…....s100

References…………………………...……………………………………..................s145

NMR spectra………………………………………………………………..............s149

General information

Dry solvents were purchased from commercial suppliers or dried on a column of alumina. Reactions were monitored by thin-layer chromatography (TLC) on silica gel pre-coated aluminium sheets (Merck Kieselgel 60 F254 plates). Visualization was accomplished by irradiation with UV light at 254 nm, and/or potassium permanganate stain. Column chromatography was performed on Merck silica gel (60, particle size 0.040-0.063 mm). Preparative thin layer chromatography was performed on Analtech silica gel GF 20x20 cm 500-1500 micron plates. All NMR spectra were recorded on Bruker AVIIIHD 400, Bruker NEO 400, AVIIIHD 500, or Bruker AVIII 600, or Bruker NEO 600. ^{1}H and ^{13}C NMR spectral data are reported as chemical shifts (δ) in parts per million (ppm) relative to the solvent peak using the Bruker internal referencing procedure (edlock). ^{19}F NMR spectra are referenced relative to $CFCl_3$. Coupling constants, *J*, are reported in Hz to the nearest 0.1 Hz. Unless otherwise stated, ^{13}C spectra are ^{1}H decoupled and reported coupling constants for ^{13}C spectra correspond to ^{19}F–^{13}C heteronuclear coupling. Data are reported as follows: chemical shift, multiplicity (s = singlet, d = doublet, t = triplet, q = quartet, pent = pentet, hept = heptet, br = broad, m = multiplet), coupling constants (Hz) and integration. NMR spectra were processed with MestReNova 14.1.2. High resolution mass spectra were determined on a Thermo Exactive mass spectrometer, for electrospray ionization (ESI-TOF), or an Agilent 7200 Accurate Mass Q-TOF GC-MS connected to a 7890 GC system, for electron ionization (GC-EI). Some compounds were found to be unstable under a variety of MS ionization methods (CI, EI, ESI, GC-MS) and therefore no HRMS could be obtained for them; this is stated for the relevant compounds. Infrared spectra were recorded as the neat compound or as an evaporated solution using a Bruker Tensor 27 FTIR spectrometer. Absorptions are reported in wavenumber (cm^{-1}). Melting points of solids were measured on a Stuart SMP20 melting point apparatus and are uncorrected. IUPAC names were obtained using the ChemDraw service. Weighing was performed with a 4 or 5 decimal place balance. All commercially available chemicals were purchased from commercial suppliers or otherwise synthesized according to literature. CsF and KF were ground in a mortar and stored in a desiccator. Photochemical reactions were performed in 4 mL glass vials, or 10/20 mL glass microwave vials in a SynLED Parallel Photoreactor (equipped with a fan) with an EvoluChem 18 W blue LED lamp (λ = 450 nm) or Kessil 40 W LED lamp (λ = 390nm). For reactions in flow, a HepatoChem photoredox flow reactor (PFA tubing, 2 mL volume) was used. For manual radiochemistry experiments: [^{18}F]Fluoride was produced by Invicro (UK) or PETIC (UK) via the ^{18}O(p,n)^{18}F reaction and delivered as [^{18}F]fluoride in [^{18}O]water. Radiosynthesis and azeotropic drying were performed on a NanoTek® automated microfluidic device (Advion) or on an AllinOne radiosynthesizer (TRASIS). For automated radiochemistry experiments: [^{18}F]Fluoride was produced in an IBA Cyclon 18/9 cyclotron using the ^{18}O(p,n)^{18}F reaction in PETIC (UK). All experiments were performed on an AllinOne radiosynthesizer (TRASIS). All isolated activity yields are non-decay corrected (n.d.c.). All molar activities are decay corrected to the end of synthesis (EOS), unless stated otherwise.

Non-radioactive experiments

Iridium photocatalysts were purchased from commercial suppliers (Sigma Aldrich, TCI, BLD, ABCR). 4CzIPN was synthesized according to literature procedure.[1] All spectroscopic data were in accordance with the literature.[1]

Optimization of the reaction conditions

Procedure for the optimization studies

To an oven-dried 4 mL glass vial equipped with a magnetic stirrer was added CsF (given equivalents) followed by anhydrous HFIP (given equivalents) or alternative proton source, and the suspension was sonicated until homogeneous. The solvent was then added (anhydrous, given concentration) followed by photocatalyst (1.0 mol%) and 1-(1-([1,1'-biphenyl]-4-yl)ethoxy)-2,2,6,6-tetramethylpiperidine (16.9 mg, 0.05 mmol, 1.0 equiv) **1**. The vial was sealed with a septum, degassed with nitrogen bubbling for 10 seconds and wrapped in parafilm. The reaction mixture was stirred in a SynLED Parallel Photoreactor with an EvoluChem 18 W blue LED lamp (λ = 450 nm) at room temperature (with a fan) for 16 h. 4-Fluoroanisole (internal standard, 10 µL, 88 µmol, 1.76 equiv) was added and the reaction mixture was diluted with $CDCl_3$ and analyzed by quantitative ^{19}F NMR.

Table s1: Solvent screen

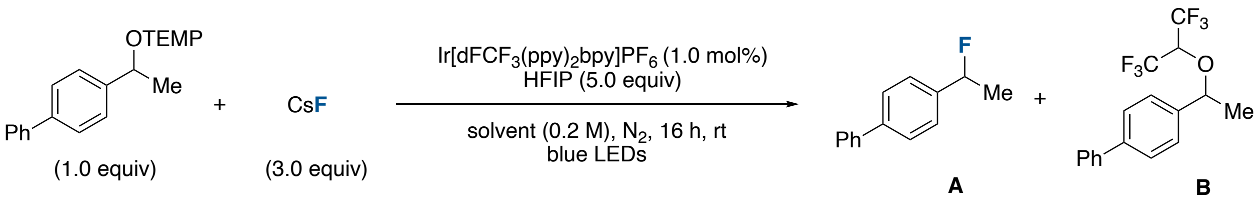

Entry	Solvent	Yield (A)	Yield (B)
**1**	CH_2Cl_2	41%	27%
**2**	$CHCl_3$	11%	5%
**3**	benzene	traces	traces
**4**	MeCN	traces	traces
**5**	$MeNO_2$	traces	4%
**6**	DMF	traces	traces
**7**	THF	27%	19%
**8**	CPME	34%	42%
**9**	DME	33%	17%
**10**	1,2-DFB	4%	3%
**11**	MeTHF	28%	33%
**12**	MTBE	18%	19%
**13**	Et_2O	27%	39%
**14**	DCE	23%	12%
**15**	1,4-dioxane	25%	17%
**16**	$^{i}Pr_2O$	19%	42%

Table s2: HFIP equivalents screen

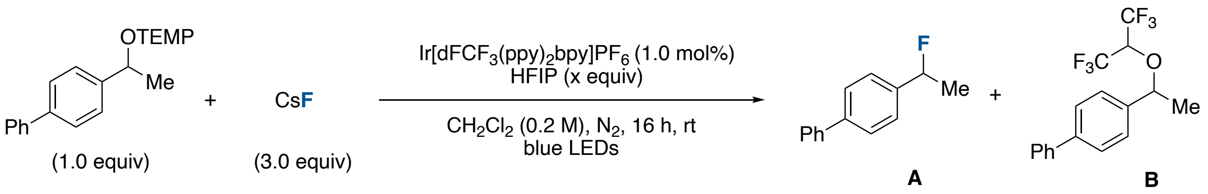

Entry	HFIP equivalents	Yield (A)	Yield (B)
**1**	1.0	10%	45%
**2**	5.0	41%	27%
**3**	10.0	43%	17%
**4**	20.0	4%	41%

Table s3: Solvent concentration screen

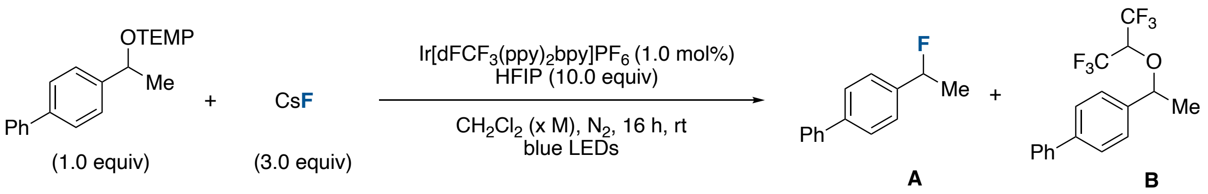

Entry	Concentration	Yield (A)	Yiel (B)
**1**	0.2 M	43%	17%
**2**	0.1 M	43%	11%
**3**	0.05 M	56%	15%
**4**	0.025 M	52%	15%

Table s4: Screen of alcohols

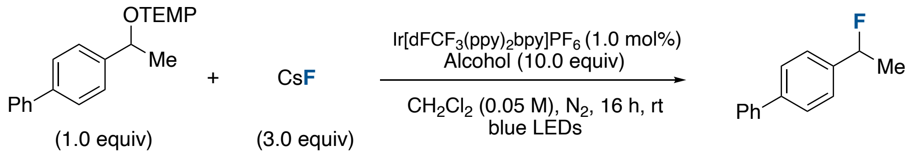

Entry	Alcohol	Yield (A)
**1**	hexafluoroisopropanol	59%
**2**	trifluoroethanol	17%
**3**	nonafluoro-*tert*-butanol	13%
**4**	hexafluorophenylpropanol	39%
**5**	trifluorophenylethanol	46%
**6**	methanol	4%
**7**	ethanol	6%
**8**	isopropanol	5%
**9**	*tert*-Butanol	0%

Table s5: Screen of photocatalysts

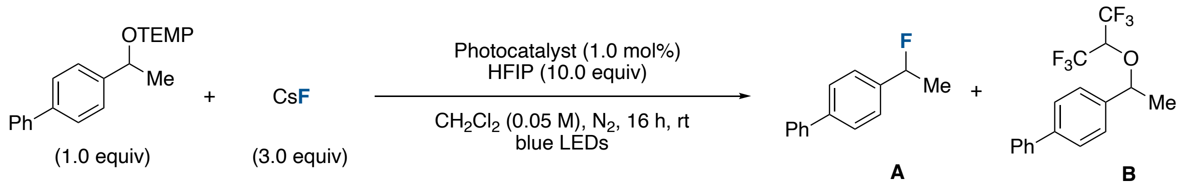

Entry	Photocatalyst	$E_{1/2}$ (PC*/PC$^{\cdot-}$)	Yield (A)	Yield (B)
**1**	Ir[dFCF$_3$(ppy)$_2$bpy]PF$_6$	+1.32	56%	15%
**2**	Ir[dFFppy]$_2$-(4,4’-dCF$_3$bpy)PF$_6$	+1.55	59%	21%
**3**	[Ir(dFCF$_3$ppy)$_2$-5,5’-dCF$_3$bpy)]PF$_6$	+1.68	55%	21%
**4**	4CzIPN	+1.35	52%	20%

Table s6: Temperature screen

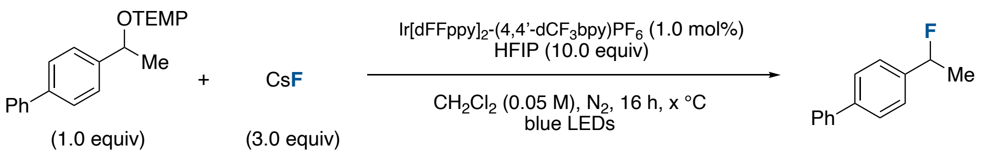

Entry	Temperature	Yield (A)
**1**	rt (20-25 °C)	59%
**2**	40 °C	61%

Table s7: CsF equivalents screen

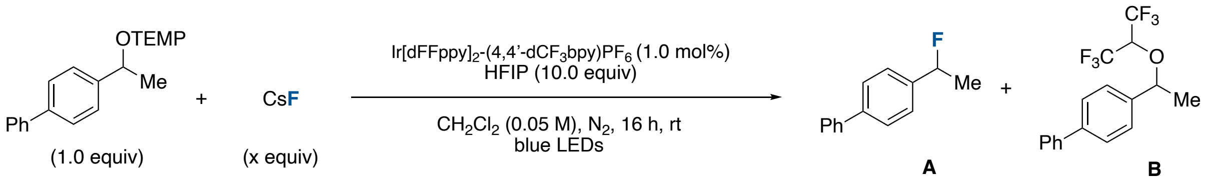

Entry	CsF equivalents	Yield (A)	Yield (B)
**1**	1.0	38%	38%
**2**	2.0	59% (50%)[a]	25% (27%)[a]
**3**	3.0	59%	21%
**4**	5.0	44%	23%

[a] isolated yield (0.5 mmol scale)

Table s8: Screen of fluoride sources

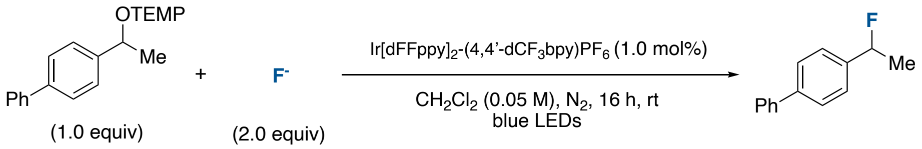

Entry	Fluoride source	Yield (A)
**1**	CsF + 10.0 equiv HFIP	59% (50%)[a]
**2**	TBAF•3H_2O	traces
**3**[a]	TBAF•3H_2O + 10.0 equiv HFIP	55%
**4**	3HF•NEt_3	84%[b]
**5**	KF + 10.0 equiv HFIP	20%
**6**	CsF	0%
**7**	CsF + Schreiner's urea (10.0 mol%)	traces

[a] isolated yield (0.5 mmol scale)
[b] Reaction performed with 0.2 M solvent concentration

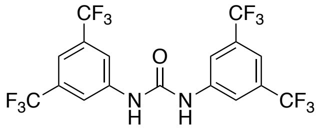

Schreiner's urea

Table s9: Control experiments

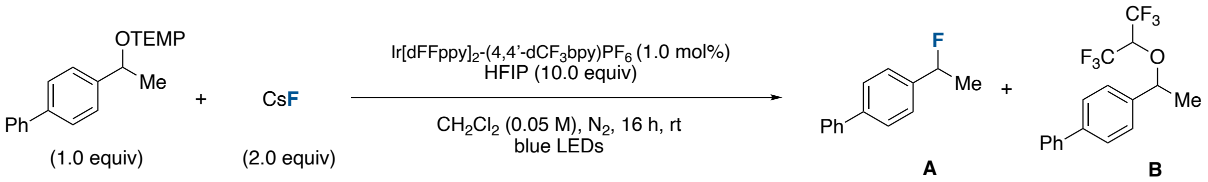

Entry	Deviation from standard conditions	Yield (A)	Yield (B)
**1**	none	59% (50%)[a]	25% (27%)[a]
**2**	no photocatalyst	0%	0%
**3**	no irradiation	0%	0%

[a] isolated yield (0.5 mmol scale)

Time course experiment

Procedure

To an oven-dried 20 mL glass microwave vial equipped with a magnetic stirrer was added CsF (152 mg, 1.0 mmol, 2.0 equiv) followed by anhydrous HFIP (526 μL, 5.0 mmol, 10.0 equiv) and the suspension was sonicated until homogeneous. Anhydrous and degassed CH_2Cl_2 was then added (10.0 mL, 0.05 M) followed by 4-fluoroanisole (56.6 μL, 0.5 mmol, 1.0 equiv), Ir[dFFppy]$_2$-(4,4'-dCF$_3$bpy)PF$_6$ (5.2 mg, 5.0 μmol, 1.0 mol%) and 1-(1-([1,1'-biphenyl]-4-yl)ethoxy)-2,2,6,6-tetramethylpiperidine **1** (169 mg, 0.5 mmol, 1.0 equiv). The vial was sealed with a septum, degassed with nitrogen bubbling for 10 seconds and wrapped in parafilm. The reaction mixture was stirred in a SynLED Parallel Photoreactor with an EvoluChem 18 W blue LED lamp (λ = 450 nm) for 48 h at room temperature (with a fan). Aliquots (200 μL) of the reaction mixture were taken at given time intervals, diluted with $CDCl_3$ and analyzed by quantitative ^{19}F NMR to monitor the progress of the reaction.

Table s10: Time course experiment

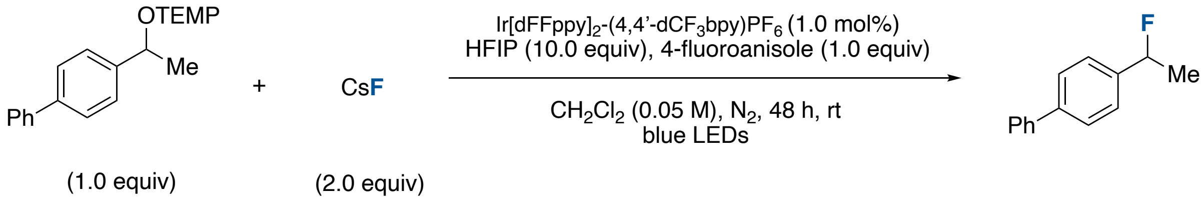

Entry	Time	Yield
**1**	1 min	12%
**2**	10 min	53%
**3**	1 h	57%
4	2 h	59%
**5**	4 h	59%
**6**	6 h	59%
**7**	24 h	59%
**8**	48 h	59%

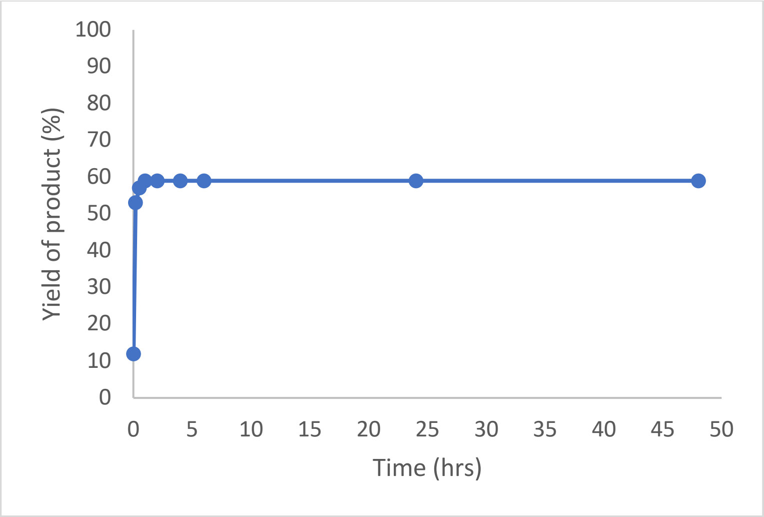

Figure s1: Plot of yield *vs* time

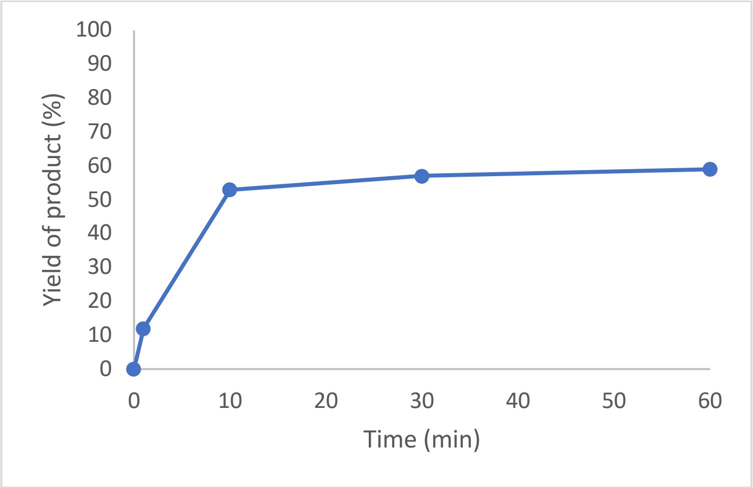

Figure s2: Plot of yield *vs* time for the first hour of irradiation

Proposed mechanism

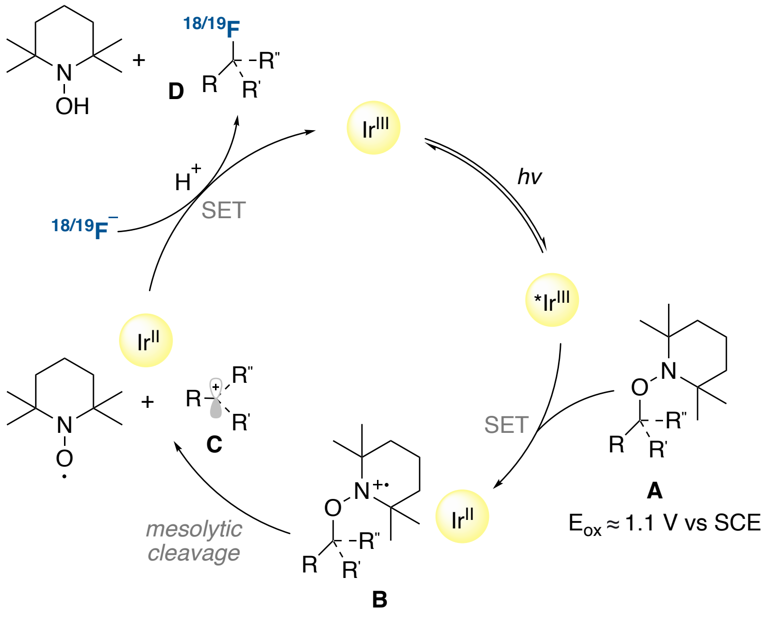

Scheme 1: Proposed reaction mechanism

<u>Discussion</u>

In line with previous mechanistic observations,[2] we propose that upon irradiation with blue light, the excited state of the photocatalyst can undergo single-electron transfer (SET) with the alkoxyamine substrate. Mesolytic cleavage of the resulting radical cation furnishes a carbocation, which is readily trapped by fluoride, yielding the desired (radio)fluorinated product. Concurrently, reduction of the TEMPO radical by the photocatalyst, promoted by the presence of a proton source,[2] regenerates the photocatalyst ground state.

Synthesis of starting materials

1,2,3,4-tetrahydronaphthalene, 1,2,3,4-tetrahydronaphthalen-1-ol, 1-(1-bromoethyl)-2-methyl-3-(trifluoromethyl)benzene, 1-(1-bromoethyl)-4-nitrobenzene, 1-(3-trifluoromethoxy)phenylethanol, 1-(4-bromophenyl)ethan-1-ol, 1-(*p*-tolyl)ethan-1-ol, 1-(pyridin-3-yl)ethan-1-amine, 1-([1,1'-biphenyl]-4-yl)ethan-1-ol, 1-ethyl-4-methoxybenzene, 1,4-divinylbenzene, 1,3-dioxoisoindolin-2-yl 5-(2,5-dimethylphenoxy)-2,2-dimethylpentanoate, 2-(bromomethyl)naphthalene, 2,2-diphenylacetaldehyde, 2,4,6-tetraphenylpyrilium tetrafluoroborate, 2-ethylbenzofuran, 3-chloro-1-aminoadamantane hydrochloride, 3,5-di-*tert*-butyl-[1,1'-biphenyl]-4-ol, 4-chloroquinazoline, 4-formylbenzonitrile, 4,4,5,5-tetramethyl-2-((phenylthio)methyl)-1,3,2-dioxaborolane, (4-(bromomethyl)phenyl)(methyl)sulfane, (4-aminophenyl)methanol, (4-methoxyphenyl)methanamine, 5-bromo-5-phenylpentanoate, Carprofen, cyclohexene, cyclohexylbenzene, cumene, Cyhalofop, desoxyanisoin, ethylbenzene-d_{10}, Flurbiprofen, Ibuprofen, Ketoprofen, Loxoprofen, methyl 2-(4-formylphenyl)acetate, methyl 4-(1-bromoethyl)benzoate, Naproxen and *N*-phthaloyl-L-phenylalanine were purchased from commercial suppliers (Fluorochem, Sigma Aldrich, TCI, BLDpharm, Apollo Scientific, Ambeed, Manchester Organics, Biosynth) and used as received, without further purification.

General literature procedure A:[2]

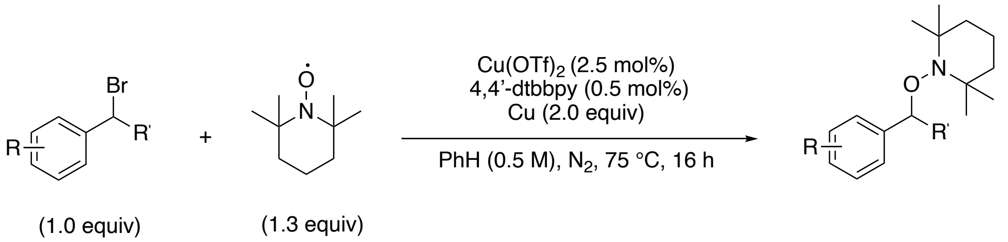

Benzylic bromide (1.0 equiv, if solid), $Cu(OTf)_2$ (2.5 mol%), 4,4'-di-*tert*-butyl-2,2'-bipyridine (10.0 mol%), Cu powder (2.0 equiv) and TEMPO (1.3 equiv) were added into a flame-dried round bottom flask equipped with a magnetic stirrer. The flask was evacuated and backfilled with N_2 three times. Anhydrous and degassed benzene (0.5 M) was added followed by the benzylic bromide (if liquid). The reaction mixture was stirred at 75°C for 16 h under N_2, then cooled to room temperature, filtered over a short silica pad with the aid of some Et_2O and

concentrated *in vacuo*. The crude product was purified by silica gel column chromatography (pentane/ethyl acetate) to give the requisite product.

General literature procedure B:[3]

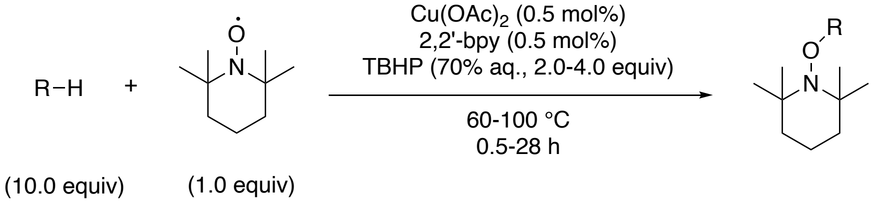

Under air, TEMPO (1.0 equiv), substrate (10.0 equiv), $Cu(OAc)_2$ (0.5 mol%), 2,2'-bpy (0.5 mol%), TBHP (70% aq, 2.0 equiv) were added into a round bottom flask equipped with a magnetic stirrer. The reaction was stirred at the given temperature until its color turned from dark red to light green (indication of the consumption of TEMPO starting material). Upon completion, the mixture was diluted with Et_2O and washed with a saturated aqueous NaCl solution twice. The organic phase was dried over Na_2SO_4 and evaporated *in vacuo*. The crude product was purified by silica gel column chromatography (pentane/ethyl acetate) to give the requisite product.

General literature procedure C:[4]

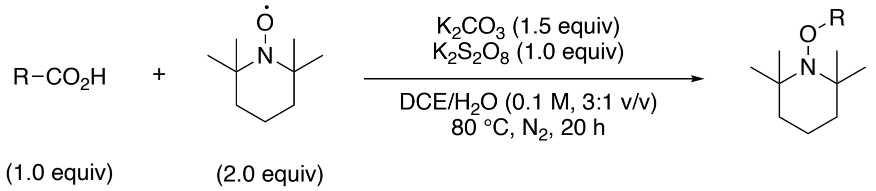

To a flame-dried round bottom flask equipped with a magnetic stirrer were added carboxylic acid (1.0 equiv), TEMPO (2.0 equiv), K_2CO_3 (1.5 equiv) and $K_2S_2O_8$ (1.0 equiv). The flask was evacuated and backfilled with nitrogen three times prior to the addition of DCE and H_2O (0.1 M, 3:1 v/v). The reaction mixture was then stirred at 80 °C for 20 h under N_2. Upon completion, the reaction mixture was extracted with EtOAc three times. The combined organic layers were washed with a saturated aqueous NaCl solution, dried over Na_2SO_4 and evaporated *in vacuo*. The crude product was purified by silica gel column chromatography (pentane/ethyl acetate) to give the requisite product.

General literature procedure D:[5]

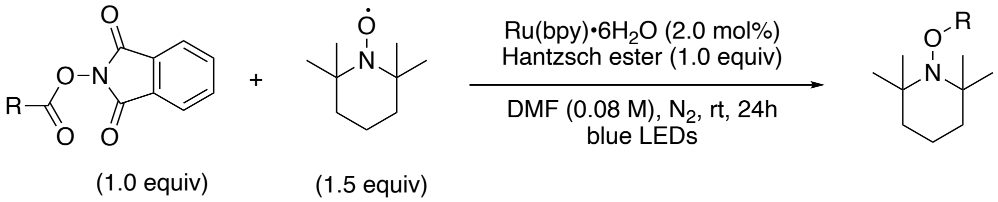

To a flame-dried round bottom flask equipped with a magnetic stirrer were added redox-active ester (1.0 equiv, if solid), TEMPO (1.5 equiv), $Ru(bpy)_3Cl_2•6H_2O$ (2.0 mol%), and Hantzsch ester (1.0 equiv). The flask was evacuated and backfilled with nitrogen three times prior to the addition of anhydrous and degassed DMF (0.08 M) and redox-active ester (if liquid). The reaction mixture was stirred under the irradiation of an 18 W blue LED lamp (λ = 450 nm) at room temperature for 24 h. Upon completion, the reaction mixture was quenched by the addition of a saturated aqueous NaCl solution and extracted with EtOAc three times. The combined organic layers were dried over Na_2SO_4 and evaporated *in vacuo*. The crude product was purified by silica gel column chromatography (pentane/ethyl acetate) to give the requisite product.

1-(1-([1,1'-Biphenyl]-4-yl)ethoxy)-2,2,6,6-tetramethylpiperidine (1)

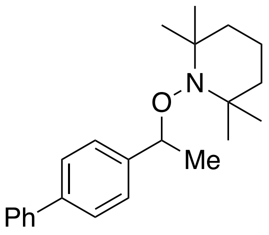

Synthesized according to general literature procedure A from 4-(1-bromoethyl)-1,1'-biphenyl, which was synthesized according to literature procedure from 1-([1,1'-biphenyl]-4-yl)ethan-1-ol.[6] Purified by silica gel column chromatography (pentane/EtOAc, 100:0 to 98:2) to give the product as a white solid (1.411 g, 4.18 mmol, 47%). **^{1}H NMR** (500 MHz, $CDCl_3$) δ 7.63–7.59 (m, 2H), 7.57–7.53 (m, 2H), 7.45–7.37 (m, 4H), 7.35–7.30 (m, 1H), 4.84 (q, J = 6.6 Hz, 1H), 1.52 (d, J = 6.7 Hz, 6H), 1.38 (brs, 2H), 1.31 (brs, 4H), 1.19 (brs, 3H), 1.06 (brs, 3H), 0.72 (brs, 3H); **^{13}C NMR** (101 MHz, $CDCl_3$) δ 145.1, 141.3, 139.8, 128.9, 127.2, 127.2, 126.9, 82.9, 59.9, 40.6, 34.7, 34.5 23.7, 20.6, 17.4; **HRMS** (ESI-TOF) calculated for $C_{23}H_{31}NO$ $[M+H^+]^+$:

338.2478, found 338.2477; **IR**: 3077, 3028, 3005, 2973, 2934, 2868, 2847, 2399, 2349, 2282, 1600, 1485, 1450, 1404, 1375, 1362, 1299, 1262, 1240, 1207, 1132, 1093, 1058, 1023, 1009, 986, 957, 935, 882, 841, 784, 768, 738, 728, 702, 617; **m.p.**: 92–93 °C.

2,2,6,6-Tetramethyl-1-(1-phenylethoxy)piperidine (S5)

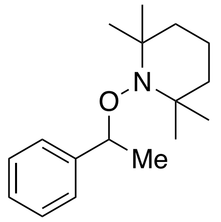

Synthesized according to general literature procedure B from ethylbenzene. All spectroscopic data were in accordance with the literature.[3] The synthesis of this compound has also been reported from dimethyl 2,6-dimethyl-4-(1-phenylethyl)-1,4-dihydropyridine-3,5-dicarboxylate.[7]

2,2,6,6-Tetramethyl-1-(1-(phenyl-*d*5)ethoxy-1,2,2,2-*d*4)piperidine (S6)

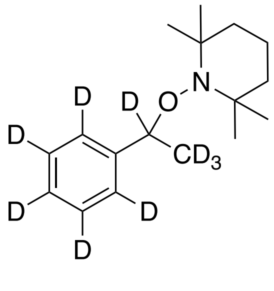

Synthesized according to general literature procedure B with 3.0 equivalents of ethylbenzene-d_{10}. The reaction mixture was stirred at 60 °C for 4 h and the crude product was purified by silica gel column chromatography (pentane/EtOAc, 100:0 to 99:1) to give the product as a pale-yellow solid (170 mg, 0.63 mmol, 12%). **^{1}H NMR** (500 MHz, $CDCl_3$) δ 1.62–1.23 (m, 9H), 1.17 (s, 3H), 1.03 (s, 3H), 0.68 (s, 3H); **^{13}C NMR** (500 MHz, $CDCl_3$) (^{13}C.-^{2}H coupling observed) δ 145.7, 127.6 (t, J= 24.3), 126.4 (t, J = 24.3), 126.3 (t, J = 24.3), 82.6 (t, J = 22.3), 59.8, 40.5, 34.6 (br), 34.2 (br), 22.7 (h, J = 19.1), 20.5 (br), 17.4; **HRMS** (ESI-TOF) calculated for $C_{17}H_{19}D_9NO$ $[M+H^+]^+$: 271.2730, found 271.2729; **IR**: 3734, 2974, 2933, 2360, 2341,

1734, 1717, 1699, 1684, 1653, 1636, 1559, 1541, 1507, 1473, 1375, 1361, 1260, 1209, 1134, 1035, 958, 933, 795, 669, 654; **m.p.**: 46–47 °C.

1**-Methyl-4-(1-((2,2,6,6-tetramethylcyclohexyl)oxy)ethyl)benzene (S7)**

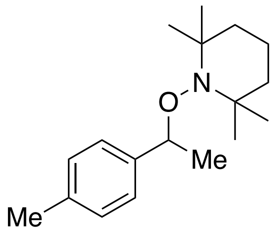

To a flame-dried 20 mL glass vial equipped with a magnetic stirrer was added 1-(1-(*p*-tolyl)ethyl)tetrahydro-1*H*-thiophen-1-ium tetrafluoroborate (294 mg, 1.00 mmol, 1.0 equiv) followed by anhydrous DMA (5.0 mL, 0.2 M), TEMPO (234 mg, 1.5 mmol, 1.5 equiv), and Mn (110 mg, 2.0 mmol, 2.0 equiv). The vial was sealed with a septum and the reaction mixture was degassed with N_2 bubbling while stirring for one minute, then stirred at 60 °C under N_2 for 24 h. The reaction mixture was then diluted with EtOAc and filtered over a short silica pad. The filtrate was washed with a saturated aqueous NaCl solution three times. The organic layer was dried over Na_2SO_4 and evaporated *in vacuo*. The crude product was purified by silica gel column chromatography (pentane/EtOAc, 100:0 to 99:1) to give the product as a white solid (137 mg, 0.50 mmol, 50%). **^{1}H NMR** (400 MHz, $CDCl_3$) δ 7.21 (d, J = 8.1 Hz, 2H), 7.12 (d, J = 7.7 Hz, 2H), 4.75 (q, J = 6.6 Hz, 1H), 2.34 (s, 3H), 1.47 (d, J = 6.7 Hz, 6H), 1.38 (brs, 2H), 1.29 (brs, 4H), 1.16 (brs, 3H), 1.03 (brs, 3H), 0.70 (brs, 3H); **^{13}C NMR** (101 MHz, $CDCl_3$) δ 143.0, 136.4, 128.8, 126.6, 83.0, 59.8, 40.5, 34.6 (br), 34.4 (br), 23.7, 21.3, 20.5 (br), 17.4; **HRMS**: (ESI-TOF) calculated for $C_{18}H_{29}NO$ $[M+H^+]^+$: 276.2322, found 276.2316. All spectroscopic data were in accordance with the literature.[2]

1-(1-(*p*-Tolyl)ethyl)tetrahydro-1*H*-thiophen-1-ium tetrafluoroborate **(S7a)**

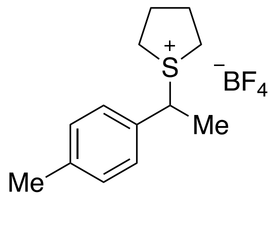

To a flame-dried round bottom flask tube equipped with a magnetic stirrer was added 1-(4-methylphenyl)ethanol (970 mg, 7.12 mmol, 1.0 equiv). The flask vas evacuated and backfilled with N_2 three times prior to the addition of anhydrous CH_2Cl_2 (23.7 mL, 0.3 M). PBr_3 (1.004 mL, 10.68 mmol, 1.5 equiv) was then added dropwise at 0 °C. The reaction mixture was stirred at 0 °C under N_2 for 3 h, then quenched *via* the addition of a saturated aqueous $NaHCO_3$ solution and extracted with CH_2Cl_2 three times. The combined organic layers were washed with a saturated aqueous NaCl solution, dried over Na_2SO_4 and evaporated *in vacuo* to give 1-(1-bromoethyl)-4-methylbenzene, which was used in the next step without further purification. To the round bottom flask containing 1-(1-bromoethyl)-4-methylbenzene, equipped with a magnetic stirrer, was added acetone (35.6 mL, 0.2 M), followed by tetrahydrothiophene (942 μL, 10.68 mmol, 1.5 equiv), and $AgBF_4$ (1.386 g, 7.12 mmol, 1.0 equiv) as a solid. The reaction mixture was stirred at room temperature under N_2 for 1 h, then filtered over a short celite pad, which was washed with MeCN. The filtrate was evaporated *in vacuo* and redissolved in MeCN. Et_2O was then added and the flask was kept in a freezer (-20 °C) for 16 h. The solvent was decanted in order to separate it from a viscous clear oil that had deposited onto the walls of the flask. The oil was redissolved in MeCN and filtered over a short celite pipette. The solvent was removed *in vacuo* to give the product as a clear oil (1.850 g, 6. 29 mmol, 88% over two steps). **^{1}H NMR** (500 MHz, CD_3CN) δ 7.36 (d, J = 8.4 Hz, 2H), 7.32 (d, J = 7.9 Hz, 2H), 4.46 (q, J = 7.0 Hz, 1H), 3.55 (dt, J = 13.7, 6.9 Hz, 1H), 3.47 (dt, J = 13.4, 6.6 Hz, 1H), 3.21 (dt, J = 14.3, 7.2 Hz, 1H), 2.95 (dt, J = 13.3, 6.5 Hz, 1H), 2.37 (s, 3H), 2.26–2.11 (m, 4H), 1.81 (d, J = 7.0 Hz, 3H); **^{13}C NMR** (126 MHz, CD_3CN) δ 141.7, 131.9, 131.3, 129.5, 56.1, 43.6, 42.6, 29.6, 29.2, 21.2, 18.7; **^{19}F NMR** (471 MHz, CD_3CN) δ -151.21 – -151.39 (m); **HRMS** (ESI-TOF) calculated for $C_{13}H_{19}S$ [M^+]: 207.1202, found 207.1201; **IR**: 2978, 2361, 2341, 1517, 1455, 1425, 1387, 1285, 1261, 1191, 1061, 827, 726, 668, 651.

1-(1-(4-*iso*-Butylphenyl)ethoxy)-2,2,6,6-tetramethylpiperidine (S8)

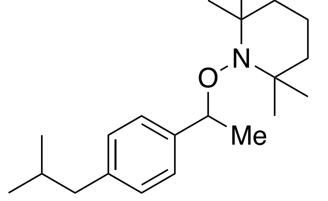

Synthesized according to general literature procedure C from Ibuprofen. All spectroscopic data were in accordance with the literature.[4]

2,2,6,6-Tetramethyl-1-(1-(4-vinylphenyl)ethoxy)piperidine (S9)

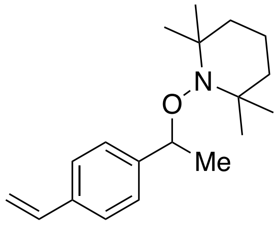

Synthesized according to literature procedure from 1,4-divinylbenzene:[8] Purified by silica gel column chromatography (pentane/EtOAc, 100:0 to 99:1) to give the product as a clear oil. **^{1}H NMR** (400 MHz, $CDCl_3$) δ 7.37 (d, J = 8.2 Hz, 2H), 7.28 (d, J = 8.2 Hz, 2H), 6.71 (dd, J = 17.6, 10.9 Hz, 1H), 5.73 (dd, J = 17.6, 0.8 Hz, 1H), 5.21 (dd, J = 10.9, 0.8 Hz, 1H), 4.78 (q, J = 6.6 Hz, 1H), 1.48 (d, J = 6.7 Hz, 6H), 1.38 (brs, 2H), 1.30 (brs, 4H), 1.17 (brs, 3H), 1.04 (brs, 3H), 0.69 (brs, 3H); **^{13}C NMR** (101 MHz, $CDCl_3$) δ 145.7, 136.9, 136.2, 126.9, 126.1, 113.3, 83.0, 59.8, 40.5, 34.6 (br), 34.4 (br), 23.7, 20.5 (br), 17.4; **HRMS** (ESI-TOF) calculated for $C_{19}H_{29}NO$ [M+H$^+$]$^+$: 288.2322, found 288.2318; **IR**: 3003, 2973, 2931, 2361, 2341, 1911, 1802, 1684, 1632, 1559, 1510, 1466, 1410, 1375, 1361, 1293, 1242, 1213, 1181, 1132, 1097, 1063, 988, 955, 933, 901, 882, 843, 813, 788, 748, 734, 699, 669, 650.

2,2,6,6-Tetramethyl-1-(1-(2-methyl-3-(trifluoromethyl)phenyl)ethoxy)piperidine (S10)

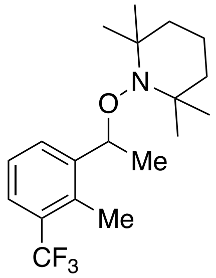

Synthesized according to general literature procedure A from 1-(1-bromoethyl)-2-methyl-3-(trifluoromethyl)benzene. Purified by silica gel column chromatography (pentane/EtOAc, 100:0 to 90:2) to give the product as a clear oil in quantitative yield (1.327 g, 3.86 mmol). **^{1}H**

NMR (500 MHz, $CDCl_3$) δ 7.64 (d, J = 7.7 Hz, 1H), 7.51 (d, J = 8.0 Hz, 1H), 7.28 (t, J = 8.1 Hz, 1H), 5.10 (q, J = 6.7 Hz, 1H), 2.40 (q, J = 1.7 Hz, 3H), 1.62–1.47 (m, 3H), 1.45 (d, J = 6.7 Hz, 3H), 1.39 (brs, 2H), 1.32 (brs, 4H), 1.18 (brs, 3H), 1.00 (brs, 3H), 0.66 (brs, 3H); **^{13}C NMR** (101 MHz, $CDCl_3$) δ 147.0, 132.4, 130.2, 124.9 (q, J = 274.0 Hz), 129.0 (q, J = 28.6 Hz), 125.7, 124.3 (q, J = 6.0 Hz), 80.2, 59.9, 59.7, 40.5, 34.8, 33.5, 23.1, 20.5, 20.4, 17.3, 14.7 (q, J = 2.4 Hz); **^{19}F NMR** (377 MHz, $CDCl_3$) δ -60.22 (s); **IR**: 2976, 2934, 1602, 1456, 1376, 1362, 1316, 1276, 1242, 1204, 1167, 1123, 1096, 1066, 1045, 1018, 991, 975, 958, 936, 909, 884, 804, 729, 705, 646; **HRMS** (ESI-TOF) calculated for $C_{19}H_{28}F_3NO$ [M+H$^+$]$^+$: 344.2196, found 344.2190.

1-(1-(2-Fluoro-[1,1'-biphenyl]-4-yl)ethoxy)-2,2,6,6-tetramethylpiperidine (S11)

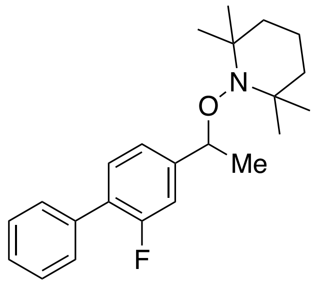

Synthesized according to general literature procedure D from Flurbiprofen. Purified by silica gel column chromatography (pentane/Et_2O, 100:0 to 99:1) to give the product as a white solid (1.182 g, 3.32 mmol, 81%). **^{1}H NMR** (500 MHz, $CDCl_3$) δ 7.59–7.52 (m, 2H), 7.47–7.41 (m, 2H), 7.40–7.32 (m, 2H), 7.17–7.11 (m, 2H), 4.82 (q, J = 6.6 Hz, 1H), 1.51 (d, J = 6.6 Hz, 6H), 1.40 (brs, 2H), 1.30 (brs, 4H), 1.18 (brs, 3H), 1.07 (brs, 3H), 0.76 (brs, 3H); **^{13}C NMR** (101 MHz, $CDCl_3$) δ 159.7 (d, J = 247.6 Hz), 147.6 (d, J = 7.2 Hz), 136.0, 130.4 (d, J = 3.6 Hz), 129.1 (d, J = 3.2 Hz), 128.5, 127.6, 127.4 (d, J = 14.3 Hz), 122.6 (d, J = 3.2 Hz), 114.2 (d, J = 23.4 Hz), 82.5, 59.9, 40.5, 34.6, 34.3, 23.6, 20.5 (br), 17.4; **^{19}F NMR** (377 MHz, $CDCl_3$) δ -118.58 (dd, J = 12.1, 7.8 Hz); **IR**: 2972, 2943, 2872, 2360, 2341, 1951, 1901, 1741, 1625, 1582, 1563, 1513, 1484, 1456, 1417, 1373, 1354, 1293, 1271, 1244, 1228, 1209, 1183, 1160, 1129, 1104, 1069, 1047, 1014, 992, 976, 956, 941, 916, 872, 853, 835, 787, 766, 735, 725, 697, 657, 614; **HRMS** (ESI-TOF) calculated for $C_{23}H_{30}FNO$ [M+H$^+$]$^+$: 356.2384, found 356.2378; **m.p.**: 84–85 °C.

2,2,6,6-Tetramethyl-1-(1-(3-(trifluoromethoxy)phenyl)ethoxy)piperidine (S12)

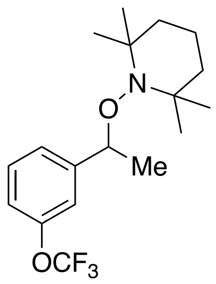

Synthesized according to general literature procedure A from 1-(1-bromoethyl)-3-(trifluoromethoxy)benzene. Purified by silica gel column chromatography (pentane/EtOAc, 100:0 to 99:1) to give the product as a yellow oil (224 mg, 0.65 mmol, 55%). **^{1}H NMR** (500 MHz, $CDCl_3$) δ 7.32 (t, J = 7.9 Hz, 1H), 7.24–7.19 (m, 2H), 7.10–7.06 (m, 1H), 4.79 (q, J = 6.6 Hz, 1H), 1.48 (d, J = 6.7 Hz, 6H), 1.37 (brs, 2H), 1.29 (brs, 4H), 1.17 (brs, 3H), 1.03 (brs, 3H), 0.62 (brs, 3H); **^{13}C NMR** (101 MHz, $CDCl_3$) δ 149.3 (q, J = 1.8 Hz), 148.4, 129.4, 125.1, 120.7 (overlapping peaks) (q, J = 256.6 Hz), 119.4, 119.4, 82.7, 60.0 (br), 59.8 (br), 40.5, 34.5 (br), 34.2 (br), 23.5, 20.4 (br), 17.3; **^{19}F NMR** (470 MHz, $CDCl_3$) δ -57.75 (s); **HRMS** (ESI-TOF) calculated for $C_{18}H_{26}F_3NO_2$ $[M+H^+]^+$: 346.1988, found 346.1982; **IR**: 2934, 1599, 1494, 1456, 1361, 1307, 1189, 1177, 1134, 1099, 941, 913, 815, 759, 702, 665.

1-(1-Bromoethyl)-3-(trifluoromethoxy)benzene (**S12a**)

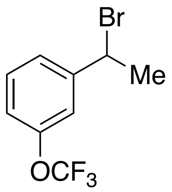

To a flame-dried round bottom flask equipped with a magnetic stirrer was added 1-(3-trifluoromethoxy)phenylethanol (750 mg, 3.64 mmol, 1.0 equiv) and the flask was evacuated and backfilled with N_2 three times prior to the addition of anhydrous CH_2Cl_2 (12.1 mL, 0.3 M). PBr_3 (513 µL, 5.46 mmol, 1.5 equiv) was then added dropwise at 0 °C. The reaction was stirred at room temperature under N_2 for 3 h, then quenched by the addition of a saturated aqueous $NaHCO_3$ solution and extracted three times with CH_2Cl_2. The combined organic layers were washed with a saturated aqueous NaCl solution, dried over Na_2SO_4 and evaporated *in vacuo* to the give the product as a clear oil (424 mg, 1.58 mmol, 43%). **^{1}H NMR** (400 MHz, $CDCl_3$) δ 7.37 (d, J = 4.7 Hz, 2H), 7.30 (brs, 1H), 7.18–7.12 (m, 1H), 5.17 (q, J = 6.9 Hz, 1H), 2.04 (d,

J = 6.9 Hz, 3H); **^{13}C NMR** (101 MHz, $CDCl_3$) δ 149.5 (q, J = 2.3 Hz), 145.6, 130.2, 125.4, 120.8, 120.6 (q, J = 257.5 Hz), 119.7, 47.7, 26.8; **^{19}F NMR** (471 MHz, $CDCl_3$) δ 57.81 (brs); **HRMS**: not found; **IR**: 2994, 2918, 2849, 2361, 2341, 1611, 1591, 1489, 1451, 1379, 1259, 1217, 1165, 1096, 1065, 1045, 1003, 984, 947, 886, 859, 796, 754, 693, 648, 635, 618.

1-(1-(4-Methoxyphenyl)ethoxy)-2,2,6,6-tetramethylpiperidine (S13)

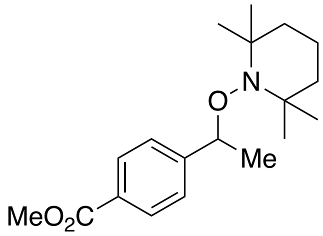

Synthesized according to general literature procedure A from methyl 4-(1-bromoethyl)benzoate. Purified by silica gel column chromatography (pentane/EtOAc, 100:0 to 99:1) to give the product as a white solid (828 mg, 2.59 mmol, 79%). **^{1}H NMR** (400 MHz, $CDCl_3$) δ 8.04–7.90 (m, 2H), 7.43–7.28 (m, 2H), 4.82 (q, J = 6.7 Hz, 1H), 1.47 (d, J = 6.7 Hz, 6H), 1.36 (br s, 2H), 1.29 (br s, 4H), 1.16 (br s, 3H), 1.01 (br s, 3H), 0.61 (br s, 3H); **^{13}C NMR** (101 MHz, $CDCl_3$) δ 167.24, 151.25, 129.61, 128.73, 126.59, 83.13, 59.82 (br), 52.09, 40.45, 34.54 (br), 34.31 (br), 23.66, 20.42 (br), 17.28; **HRMS** (ESI-TOF) calculated for $C_{19}H_{29}NO_3$ $[M+H^+]^+$: 320.2220, found 320.2215. All spectroscopic data were in accordance with the literature.[9]

1-(1-(4-Bromophenyl)ethoxy)-2,2,6,6-tetramethylpiperidine (S14)

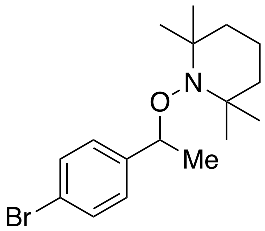

Synthesized according to literature procedure from 1-bromo-4-(1-bromoethyl)benzene,[10] which was synthesized according to literature procedure from 1-(4-bromophenyl)ethan-1-ol.[11] All spectroscopic data were in accordance with the literature.[10]

4-(1-((2,2,6,6-Tetramethylpiperidin-1-yl)oxy)ethyl)benzonitrile (S15)

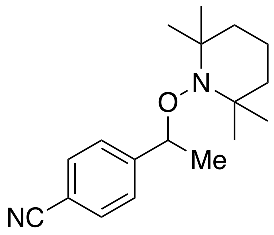

Synthesized according to general literature procedure A from 4-(1-bromoethyl)benzonitrile, which was synthesized according to literature procedure from 4-formylbenzonitrile.[12] Purified by silica gel column chromatography (pentane/EtOAc, 100:0 to 5:95) to give the product as a white solid (1.504 g, 5.25 mmol, 55%). The synthesis of this compound has also been reported from 4-(1-hydroxyethyl)benzonitrile.[13] **^{1}H NMR** (500 MHz, $CDCl_3$) δ 7.60 (d, J = 8.1 Hz, 2H), 7.41 (d, J = 8.4 Hz, 2H), 4.82 (q, J = 6.7 Hz, 1H), 1.46 (d, J = 6.7 Hz, 6H), 1.37 (br s, 2H), 1.28 (br s, 4H), 1.16 (br s, 3H), 1.01 (br s, 3H), 0.60 (br s, 3H); **^{13}C NMR** (126 MHz, $CDCl_3$) δ 151.3, 132.2, 127.3, 119.2, 110.7, 82.9, 59.9 (br), 40.4, 34.6 (br), 34.3 (br), 23.6, 20.4 (br), 17.3; **HRMS** (ESI-TOF) calculated for $C_{18}H_{26}N_2O$ $[M+H^+]^+$: 287.2188, found 287.2155; **IR**: 3469, 3428, 3066, 2998, 2972, 2935, 2870, 2360, 2232, 1929, 1730,1688, 1608, 1504, 1462, 1445, 1375, 1361, 1303, 1292, 1276, 1257, 1242, 1211, 1184, 1131, 1096, 1061, 1046, 1020, 990, 974, 955, 933, 883, 842, 814, 787, 741, 720, 689, 663, 641, 624, 611; **m.p.**: 90–92 °C. All spectroscopic data were in accordance with the literature.[13]

2,2,6,6-Tetramethyl-1-(1-(4-(4,4,5,5-tetramethyl-1,3,2-dioxaborolan-2-yl)phenyl)ethoxy)piperidine (S16)

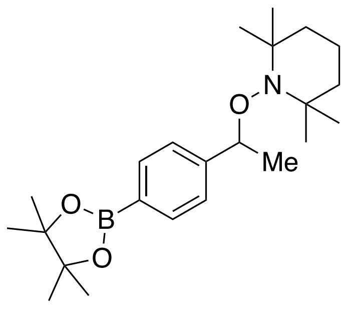

Synthesized according to an adapted literature procedure:[14]

To a flame-dried Schlenk tube equipped with a magnetic stirrer were added 1-(1-(4-bromophenyl)ethoxy)-2,2,6,6-tetramethylpiperidine **S14** (340 mg, 1.0 mmol, 1.0 equiv), B_2Pin_2 (762 mg, 3.0 mmol, 3.0 equiv), K_3PO_4 (637 mg, 3.0 mmol, 3.0 equiv), $Pd(OAc)_2$ (5 mg, 0.02 mmol, 2.0 mol%) and Sphos (21 mg, 0.05 mmol, 5.0 mol%). The tube vas evacuated and backfilled with N_2 three times prior to the addition of anhydrous 1,4-dioxane (2.0 mL, 0.5 M). The reaction mixture was stirred at room temperature under N_2 for 16 h, then filtered through a short celite pad and washed with EtOAc. The filtrate was then evaporated *in vacuo* and the crude product was purified by silica gel column chromatography (pentane/EtOAc, 100:0 to 99:1) to give the product as a white solid (88 mg, 0.23 mmol, 23%). **^{1}H NMR** (500 MHz, $CDCl_3$) δ 7.76 (d, J = 8.1 Hz, 2H), 7.32 (d, J = 8.1 Hz, 2H), 4.78 (q, J = 6.7 Hz, 1H), 1.46 (d, J = 6.7 Hz, 6H), 1.34 (s, 15H), 1.29 (br s, 3H), 1.16 (br s, 3H), 1.01 (br s, 3H), 0.65 (br s, 3H), **^{13}C NMR** (126 MHz, $CDCl_3$) δ 167.3, 151.3, 129.6, 128.7, 126.6, 83.12, 59.9, 52.1, 40.5, 34.6, 34.3, 23.7, 20.4 (br), 17.3; **^{11}B NMR** (161 MHz, $CDCl_3$) δ 31.49 (brs); **HRMS** (ESI-TOF) calculated for $C_{23}H_{38}BNO_3$ $[M+H^+]^+$: 388.3018, found 388.3010; **IR**: 3727, 3628, 2976, 2930,2361, 2341, 2111, 1613, 1559, 1508, 1457, 1399,1361, 1322, 1291, 1270, 1214, 1145, 1114, 1092, 1063, 1021, 964, 938, 860, 830, 799, 713, 677, 668, 659; **m.p.**: 130–132 °C.

Phenyl(3-(1-((2,2,6,6-tetramethylpiperidin-1-yl)oxy)ethyl)phenyl)methanone (S17)

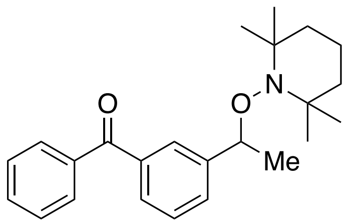

Synthesized according to general literature procedure C from Ketoprofen. Purified by silica gel column chromatography (pentane/EtOAc, 100:0 to 98:2) to give the product a pale-yellow oil (687 mg, 1.88 mmol, 24%). **^{1}H NMR** (500 MHz, $CDCl_3$) δ 7.83–7.80 (m, 2H), 7.76 (brs, 1H), 7.68 (dt, J = 7.6, 1.5 Hz, 1H), 7.61–7.54 (m, 2H), 7.47 (t, J = 7.7 Hz, 2H), 7.43 (t, J = 7.6 Hz, 1H), 4.86 (q, J = 6.6 Hz, 1H), 1.50 (d, J = 6.7 Hz, 6H), 1.38 (brs, 2H), 1.29 (brs, 4H), 1.15 (brs, 3H), 1.04 (brs, 3H), 0.68 (brs, 3H); **^{13}C NMR** (126 MHz, $CDCl_3$) δ 197.0, 146.2, 137.9, 137.5, 132.5, 130.9, 130.2, 128.8, 128.5, 128.4, 128.2, 83.0, 59.9, 59.8, 40.5, 34.5 (br), 23.5, 20.5 (br), 17.3; **HRMS** (ESI-TOF) calculated for $C_{24}H_{31}NO_2$ $[M+H^+]^+$: 366.2428, found 366.2422. All spectroscopic data were in accordance with the literature.[5]

2-(4-(1-((2,2,6,6-Tetramethylpiperidin-1-yl)oxy)ethyl)benzyl)cyclopentan-1-one (S18)

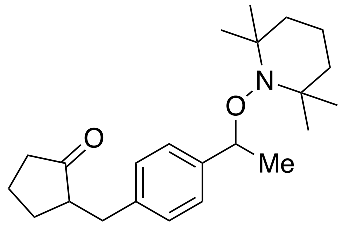

Synthesized according to general literature procedure C from Loxoprofen. Purified by silica gel column chromatography (pentane/EtOAc, 100:0 to 90:2) to give the product as a clear oil (831 mg, 2.32 mmol, 57%) (diastereomers not resolved). **^{1}H NMR** (500 MHz, $CDCl_3$) δ 7.22 (d, J = 8.1 Hz, 2H), 7.09 (d, J = 8.1 Hz, 2H), 4.74 (q, J = 6.6 Hz, 1H), 3.12 (dd, J = 13.9, 4.1 Hz, 1H), 2.52 (dd, J = 13.9, 9.6 Hz, 1H), 2.40–2.28 (m, 2H), 2.15–2.01 (m, 2H), 1.98–1.88 (m, 1H), 1.78–1.67 (m, 1H), 1.61–1.51 (m, 2H), 1.46 (d, J = 6.6 Hz, 5H), 1.36 (brs, 2H), 1.28 (brs, 4H), 1.15 (brs, 3H), 1.01 (brs, 3H), 0.62 (brs, 3H); **^{13}C NMR** (101 MHz, $CDCl_3$) δ 220.5, 143.6, 143.6, 138.4, 138.4, 128.6, 126.8, 82.8, 59.8 (br), 59.6 (br), 51.1, 40.4, 38.4, 35.4, 34.4 (br), 34.1 (br), 29.1, 23.3, 23.3, 20.6, 20.4, 17.3; **IR**: 3457, 2971, 2934, 2872, 2360, 1909, 1736, 1614, 1514, 1459, 1404, 1374, 1361, 1349, 1300, 1281, 1258, 1242, 1209, 1183, 1155, 1134, 1085, 1062, 1021, 988, 974, 956, 933, 882, 856, 823, 802, 789, 757, 736, 718, 702, 645, 625; **HRMS** (ESI-TOF) calculated for $C_{23}H_{35}NO_2$ $[M+H^+]^+$: 358.2741, found 358.2736.

***N*-(4-(1-((2,2,6,6-Tetramethylpiperidin-1-yl)oxy)ethyl)benzyl)cyclopropanesulfonamide (S19)**

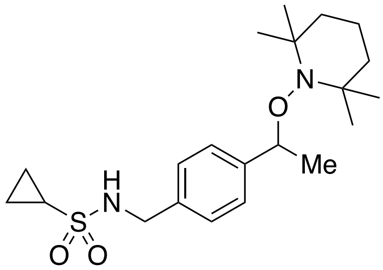

To a flame-dried round bottom flask equipped with a magnetic stirrer was added (4-(1-((2,2,6,6-tetramethylpiperidin-1-yl)oxy)ethyl)phenyl)methanamine **S19a** (250 mg, 0.86 mmol, 1.0 equiv). The flask was evacuated and backfilled with N_2 three times prior to the addition of

anhydrous CH_2Cl_2 (4.3 mL, 0.2 M), anhydrous NEt_3 (240 µL, 1.72 mmol, 2.0 equiv) and cyclopropanesulfonyl chloride (105 µL, 1.03 mmol, 1.2 equiv). The reaction mixture was stirred at room temperature under N_2 for 48 h, then washed with a saturated aqueous $NaHCO_3$ solution, an aqueous 1 M HCl solution, and a saturated aqueous NaCl solution. The organic layer was dried over Na_2SO_4 and evaporated *in vacuo*. The crude product was purified by silica gel column chromatography (pentane/EtOAc, 100:0 to 60:40) to give the product as a white solid (240 mg, 0.61 mmol, 71%). **^{1}H NMR** (400 MHz, $CDCl_3$) δ 7.30 (s, 4H), 4.77 (q, J = 6.7 Hz, 1H), 4.54 (brt, J = 6.2 Hz, 1H), 4.32 (d, J = 6.2 Hz, 2H), 2.31 (tt, J = 8.0, 4.9 Hz, 1H), 1.46 (d, J = 6.7 Hz, 6H), 1.37 (brs, 2H), 1.29 (brs, 4H), 1.21–1.09 (m, 5H), 0.95–0.86 (m, 2H), 0.62 (brs, 3H); **^{13}C NMR** (101 MHz, $CDCl_3$) δ 146.0, 135.6, 127.7, 127.2, 83.0, 59.8 (br), 47.4, 40.5, 34.6 (br), 34.4 (br), 30.7, 23.8, 20.5 (br), 17.3, 5.7; **HRMS** (ESI-TOF) calculated for $C_{21}H_{34}N_2O_3S$ $[M+H^+]^+$: 395.2363, found 395.2354; **IR**: 3215, 3014, 2968, 2934, 2872, 2849, 1742, 1620, 1450, 1435, 1414, 1375, 1323, 1295, 1258, 1214, 1187, 1160, 1143, 1063, 1042, 1020, 1002, 989, 958, 932, 911, 900, 883, 852, 837, 821, 785, 749, 723, 704, 648, 621; **m.p.**: 126–127 °C.

<u>(4-(1-((2,2,6,6-Tetramethylpiperidin-1-yl)oxy)ethyl)phenyl)methanamine</u> (**S19a**)

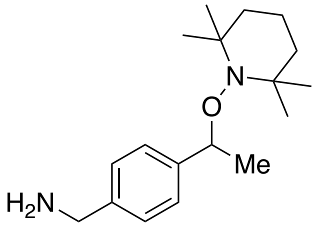

To a flame-dried round bottom flask equipped with a magnetic stirrer was added 4-(1-((2,2,6,6-tetramethylpiperidin-1-yl)oxy)ethyl)benzonitrile **S15** (1.000 g, 3.49 mmol, 1.0 equiv). The flask was evacuated and backfilled with N_2 three times prior to the addition of anhydrous THF (11.6 mL, 0.3 M) and $LiAlH_4$ (199 mg, 5.24 mmol, 1.5 equiv). The reaction mixture was stirred at room temperature under N_2 for 3 h, then quenched with a few drops of H_2O and filtered over a short celite pad with the aid of some Et_2O. An aqueous 1 M HCl solution was added to the filtrate, which was then washed with Et_2O twice. The aqueous layer was then basified by the addition of a saturated aqueous $NaHCO_3$ solution and extracted with CH_2Cl_2 five times. The combined organic layers were dried over Na_2SO_4 and evaporated *in vacuo* to give the product as an off-white solid (996 mg, 3.43 mmol, 98%). **^{1}H NMR** (400 MHz, $CDCl_3$) δ 7.29 (d, J =

7.6 Hz, 2H), 7.25 (d, *J* = 8.2 Hz, 2H), 4.78 (q, *J* = 6.7 Hz, 1H), 3.86 (s, 2H), 1.47 (d, *J* = 6.7 Hz, 6H), 1.38 (brs, 2H), 1.29 (brs, 4H), 1.17 (brs, 3H), 1.03 (brs, 3H), 0.68 (brs, 3H);**^{13}C NMR** (101 MHz, $CDCl_3$) δ 144.5, 141.9, 126.8, 126.8, 82.9, 59.7, 46.4, 40.4, 34.5, 34.3, 23.7, 20.4, 17.3; **HRMS** (ESI-TOF) calculated for $C_{18}H_{30}N_2O$ $[M+H^+]^+$: 291.2431, found 291.2425; **IR**: 3372, 3012, 2974, 2934, 2871, 2847, 1648, 1511, 1478, 1446, 1418, 1377, 1361, 1335, 1295, 1258, 1242, 1210, 1184, 1133, 1079, 1060, 1020, 988, 957, 934, 902, 882, 865, 810, 771, 721, 698, 647); **m.p.**: 47–48 °C.

1-(1-(4-((4-Iodophenoxy)methyl)phenyl)ethoxy)-2,2,6,6-tetramethylpiperidine (S20)

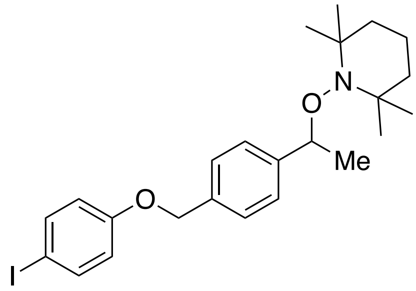

Synthesized according to general literature procedure A from 1-(1-bromoethyl)-4-((4-iodophenoxy)methyl)benzene. Purified by silica gel column chromatography (pentane/CH_2Cl_2, 100:0 to 90:10) to give the product as a white solid (272 mg, 0.54 mmol, 50%). **^{1}H NMR** (400 MHz, $CDCl_3$) δ 7.58–7.52 (m, 2H), 7.34 (s, 4H), 6.78–6.73 (m, 2H), 5.01 (s, 2H), 4.80 (q, *J* = 6.7 Hz, 1H), 1.47 (d, *J* = 6.6 Hz, 6H), 1.38 (brs, 2H), 1.29 (brs, 4H), 1.17 (brs, 3H), 1.03 (brs, 3H), 0.67 (brs, 3H); **^{13}C NMR** (101 MHz, $CDCl_3$) δ 158.9, 146.0, 138.4, 135.0, 127.4, 127.0, 117.5, 83.1, 82.9, 70.2, 59.8, 40.5, 34.6 (br), 34.4 (br), 23.7, 20.5 (br), 17.4.; **HRMS** (ESI-TOF) calculated for $C_{25}H_{34}INO_2$ $[M+H^+]^+$: 494.1550, found 494.1554; **IR**: 3006, 2982, 2980, 2967, 2936, 2870, 2844, 1907, 1885, 1740, 1584, 1567, 1486, 1462, 1445, 1375, 1282, 1243, 1215, 1187, 1172, 1134, 1101, 1062, 1007, 996, 958, 934, 885, 869, 822, 765, 726, 707, 650; **m.p.**: 99–100 °C.

2,2,6,6-Tetramethyl-1-(1-(4-(((4-(methylsulfonyl)benzyl)oxy)methyl)phenyl)ethoxy)piperidine (S21)

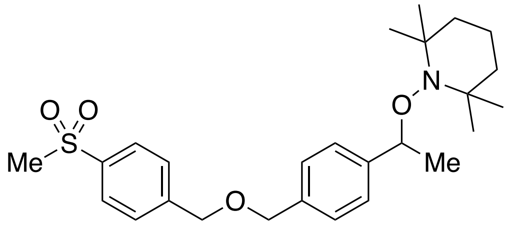

To a flame-dried Schlenk flask equipped with a magnetic stirrer was added (4-(1-((2,2,6,6-tetramethylpiperidin-1-yl)oxy)ethyl)phenyl)methanol **S21a** (201 mg, 0.69 mmol, 1.0 equiv). The flask was evacuated and backfilled with N_2 three times prior to the addition of anhydrous THF (2.3 mL, 0.3 M) and NaH (60% dispersion in mineral oil, 33 mg, 0.83 mmol, 1.2 equiv). The reaction mixture was stirred at room temperature for 1 h, then 1-(bromomethyl)-4-(methylsulfonyl)benzene (207 mg, 0.83 mmol, 1.2 equiv) was added. The reaction mixture was stirred at 50 °C under N_2 for 16 h, then additional 1-(bromomethyl)-4-(methylsulfonyl)benzene (52 mg, 0.21 mmol, 0.3 equiv) and 2.0 mL of anhydrous THF were added and the reaction mixture was further stirred for 16 h at 50 °C. Upon completion, the reaction was diluted with a saturated aqueous NaCl solution and extracted with CH_2Cl_2 three times. The combined organic layers were dried over Na_2SO_4 and evaporated *in vacuo*. The crude product was purified by silica gel column chromatography (pentane/EtOAc, 100:0 to 80:20) to give the pure product as a white solid (150 mg, 0.33 mmol, 47%). **^{1}H NMR** (400 MHz, $CDCl_3$) δ 7.93 (d, J = 8.6 Hz, 2H), 7.57 (d, J = 8.6 Hz, 2H), 7.35–7.28 (m, 4H), 4.79 (q, J = 6.7 Hz, 1H), 4.62 (s, 3H), 4.59 (s, 2H), 3.04 (s, 3H), 1.47 (d, J = 6.7 Hz, 6H), 1.37 (brs, 2H), 1.29 (brs, 4H), 1.16 (brs, 3H), 1.02 (brs, 3H), 0.67 (brs, 3H); **^{13}C NMR** (101 MHz, $CDCl_3$) δ 145.8, 145.1, 139.7, 136.1, 128.2, 127.8, 127.7, 126.9, 83.0, 72.9, 71.1, 59.8, 44.7, 40.5, 34.6 (br), 34.3 (br), 23.7, 20.5 (br), 17.5; **HRMS** (ESI-TOF) calculated for $C_{26}H_{37}NO_4S$ $[M+H^+]^+$: 460.2516, found 460.2508; **IR**: 3002, 2972, 2934, 2872, 2855, 2361, 2341, 1600, 1458, 1411, 1375, 1361, 1313, 1287, 1258, 1241, 1210, 1180, 1146, 1127, 1091, 1058, 1018, 986, 967, 936, 882, 822, 771, 735, 704, 668, 648; **m.p.**: 56–57 °C.

<u>(4-(1-((2,2,6,6-Tetramethylpiperidin-1-yl)oxy)ethyl)phenyl)methanol</u> (**S21a**)

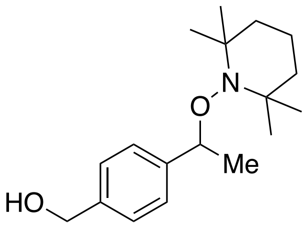

To a flame-dried round bottom flask equipped with a magnetic stirrer was added 1-(1-(4-methoxyphenyl)ethoxy)-2,2,6,6-tetramethylpiperidine **S13** (281 mg, 0.88 mmol, 1.0 equiv). The flask was evacuated and backfilled with N_2 three times prior to the addition of anhydrous THF (2.9 mL, 0.3 M). $LiAlH_4$ (50 mg, 1.32 mmol, 1.5 equiv) was then added as a solid at 0 °C. The reaction mixture was stirred at room temperature for one hour, then quenched with a few drops of H_2O and filtered over a short celite pad with the aid of some Et_2O. The filtrate was evaporated *in vacuo* and the crude product was purified by silica gel column chromatography (pentane/EtOAc, 100:0 to 60:40) to give the product as a clear oil in quantitative yield. **^{1}H NMR** (400 MHz, $CDCl_3$) δ 7.31 (m, 4H), 4.79 (q, J = 6.7 Hz, 1H), 4.66 (d, J = 4.4 Hz, 2H), 1.88 (brs, 1H), 1.47 (d, J = 6.7 Hz, 6H), 1.37 (brs, 2H), 1.30 (brs, 4H), 1.17 (brs, 3H), 1.03 (brs, 3H), 0.67 (brs, 3H); **^{13}C NMR** (126 MHz, $CDCl_3$) δ 145.5, 139.4, 126.9, 126.9, 83.0, 65.4, 59.8, 40.4, 34.6 (br), 34.3 (br), 23.8, 20.5 (br), 17.3; **HRMS** (ESI-TOF) calculated for $C_{18}H_{29}NO_2$ $[M+H^+]^+$: 292.2271, found 292.2266; **IR**: 332, 2997, 2974, 2932, 2871, 2361, 2340, 1513, 1456, 1420, 1376, 1362, 1283, 1258, 1242, 1210, 1183, 1133, 1063, 1046, 1016, 991, 958, 937, 883, 843, 820, 791, 723, 701, 641.

1-(1-(6-Methoxynaphthalen-2-yl)ethoxy)-2,2,6,6-tetramethylpiperidine (S22)

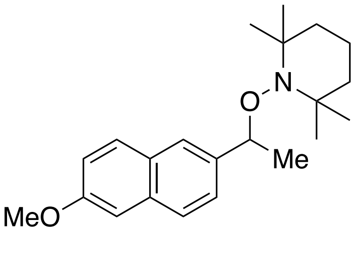

Synthesized according to general literature procedure C from Naproxen. Purified by silica gel column chromatography (pentane/Et_2O, 100:0 to 95:5) to give the product as a white solid (631 mg, 1.85 mmol, 21%).**^{1}H NMR** (500 MHz, $CDCl_3$) δ 7.74–7.69 (m, 2H), 7.65 (brs, 1H), 7.47 (dd, J = 8.5, 1.8 Hz, 1H), 7.15–7.10 (m, 2H), 4.91 (q, J = 6.6 Hz, 1H), 3.92 (s, 3H), 1.55 (d, J = 6.6 Hz, 4H), 1.51 (brs, 2H), 1.34 (brs, 6H), 1.20 (brs, 3H), 1.03 (brs, 3H), 0.61 (brs, 3H); **^{13}C NMR** (126 MHz, $CDCl_3$) δ 157.5, 141.2, 133.8, 129.5, 128.8, 126.8, 125.9, 125.1, 118.7, 105.8, 83.4, 60.0, 59.7, 55.4, 40.5, 34.8, 34.4, 23.7, 20.5, 17.4; **HRMS** (ESI-TOF) calculated for $C_{22}H_{31}NO_2$ $[M+H^+]^+$: 342.2428, found 342.2423. All spectroscopic data were in accordance with the literature.[5]

1-(Benzhydryloxy)-2,2,6,6-tetramethylpiperidine (S23)

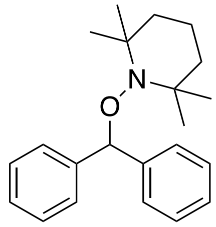

Synthesized according to an adapted literature procedure:[15a]

To a Schlenk tube equipped with a magnetic stirrer were added TEMPO (156 mg, 1.0 mmol, 1.0 equiv) and CuCl (4 mg, 35 µmol, 0.035 equiv). The flask vas evacuated and backfilled with N_2 three times prior to the addition of ^{t}BuOH (0.7 mL, 1.4 M) and 2,2-diphenylacetaldehyde (370 µL, 2.0 mmol, 2.0 equiv). H_2O_2 (30% in H_2O v/v, 204 µL, 2.0 mmol, 2.0 equiv) was then added dropwise over 10 min. The reaction mixture was stirred at 50 °C under N_2 for 1 h, then diluted with EtOAc and washed with a saturated aqueous NaCl solution. The organic layer was dried over Na_2SO_4 and evaporated *in vacuo*. The crude product was purified by silica gel column chromatography (pentane/CH_2Cl_2, 100:0 to 80:20) to give the product as a white solid (281 mg, 0.87 mmol, 87%). **^{1}H NMR** (400 MHz, $CDCl_3$) δ 7.40–7.35 (m, 4H), 7.27 (t, J = 7.6 Hz, 4H), 7.19–7.13 (m, 2H), 5.65 (s, 1H), 1.68–1.28 (m, 6H), 1.16 (brs, 6H), 0.75 (brs, 6H); **^{13}C NMR** (101 MHz, $CDCl_3$) δ 145.0, 128.2, 126.8, 126.6, 90.8, 60.0, 40.5, 34.0 (br), 20.5 (br), 17.2; **HRMS**: (ESI-TOF) calculated for $C_{22}H_{29}NO$ $[M+H^+]^+$: 324.2322, found 324.2312. All spectroscopic data were in accordance with the literature.[3]

2,2,6,6-Tetramethyl-1-((1,2,3,4-tetrahydronaphthalen-1-yl)oxy)piperidine (S24)

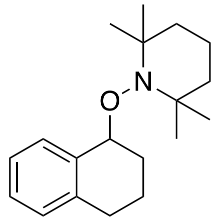

To a flame-dried 10 mL microwave vial equipped with a magnetic stirrer were cesium 2-oxo-2-((1,2,3,4-tetrahydronaphthalen-1-yl)oxy)acetate **S24a** (176 mg, 0.5 mmol, 1.0 equiv), TEMPO (156 mg, 1.0 mmol, 2.0 equiv), and Ir[dFCF$_3$(ppy)$_2$bpy]PF$_6$ (11 mg, 0.01 mmol, 0.02

equiv). The vial was sealed with a septum and evacuated and backfilled with N_2 three times prior to the addition of anhydrous 1,4-dioxane (5.0 mL, 0.1 M). The reaction mixture was degassed by bubbling with N_2 while stirring for 2 min, then stirred under the irradiation of an 18 W blue LED lamp (λ = 450 nm) at room temperature for 8 h. Upon completion, the reaction mixture was diluted with EtOAc and filtered over a short silica pad. The filtrate was washed with a saturated aqueous NaCl solution, then dried over Na_2SO_4 and evaporated *in vacuo.* The crude product was purified by silica gel column chromatography (pentane/EtOAc, 100:0 to 99:1) to give the product as a white solid (18 mg, 0.063 mmol, 13%). The substrate was also synthesized according to literature procedure from 1,2,3,4-tetrahydronaphthalene.[2] **^{1}H NMR** (500 MHz, $CDCl_3$) δ 7.65 (dd, J = 7.3, 2.0 Hz, 1H), 7.21–7.14 (m, 2H), 7.09 (d, J = 7.9 Hz, 1H), 4.90 (dd, J = 6.2, 3.9 Hz, 1H), 2.86 (dt, J = 16.8, 6.3 Hz, 1H), 2.74 (dt, J = 16.8, 6.9 Hz, 1H), 2.13–1.95 (3H), 1.79–1.68 (m, 1H), 1.67–1.30 (m, 6H), 1.28 (brs, 3H), 1.19 (brs, 3H), 1.16 (brs, 3H), 0.75 (brs, 3H); **^{13}C NMR** (126 MHz, $CDCl_3$) δ 137.9, 137.9, 130.0, 128.7, 127.3, 125.0, 78.4, 60.4 (br), 60.0 (br), 40.6, 34.8 (br), 33.4 (br), 29.4, 29.2, 21.0 (br), 20.7 (br), 19.4, 17.5; **HRMS** (ESI-TOF) calculated for $C_{19}H_{29}NO$ $[M+H^{+}]^{+}$: 288.2322, found 288.2316. All spectroscopic data were in accordance with the literature.[5]

<u>Cesium 2-oxo-2-((1,2,3,4-tetrahydronaphthalen-1-yl)oxy)acetate</u> (**S24a**)

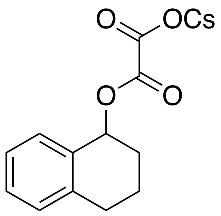

To a 7 ml glass vial equipped with a magnetic stirrer was added methyl (1,2,3,4-tetrahydronaphthalen-1-yl) oxalate **S24b** (117 mg, 0.5 mmol, 1.0 equiv), followed by THF (1.0 mL, 0.5 M). The reaction mixture was then cooled to 0 °C prior to the dropwise addition of a 1.0 M aqueous solution of $CsOH{\bullet}H_2O$ (0.4 mL, 0.4 mmol, 0.8 equiv). The reaction mixture was stirred at 0 °C for 5 min, then diluted with H_2O and washed with hexane three times. The aqueous layer was then evaporated *in vacuo* to give the product as a white solid (96 mg, 0.27 mmol, 55%). **^{1}H NMR** (400 MHz, D_2O) δ 7.39–7.32 (m, 2H), 7.28 (t, J = 7.4 Hz, 2H), 6.07 (t, J = 4.6 Hz, 1H), 2.90 (dt, J = 16.9, 5.1 Hz, 1H), 2.84–2.71 (m, 1H), 2.16–1.78 (m, 4H); **^{13}C NMR** (126 MHz, D_2O) δ 165.1, 165.0, 139.5, 134.0, 130.2, 130.0, 129.4, 127.0, 73.5, 53.4,

28.9, 18.9; ; **IR**: 3390, 2952, 2361, 1719, 1642, 1492, 1454, 1385, 1302, 1216, 1152, 1061, 1001, 977, 956, 890, 872, 836, 762, 736, 719; **HRMS** (ESI-TOF) calculated for $C_{12}H_{11}O_4$ $[M]^-$: 219.0663, found 219.0658; **m.p.**: 104–105 °C.

Methyl (1,2,3,4-tetrahydronaphthalen-1-yl) oxalate (**S24b**)

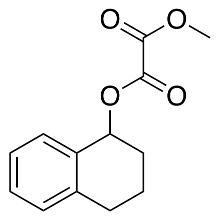

Synthesised according to an adapted literature procedure:[15b]

To a flame-dried round bottom flask equipped with a magnetic stirrer was added 1,2,3,4-tetrahydronaphthalen-1-ol (371 mg, 2.5 mmol, 1.0 equiv). The flask was evacuated and backfilled with N_2 three times prior to the addition of anhydrous Et_2O (10.0 mL, 0.25 M) and anhydrous pyridine (243 μL, 3.0 mmol, 1.2 equiv). Methyl chlorooxoacetate (276 μL, 3.0 mmol, 1.2 equiv) was then added dropwise. The reaction mixture was stirred at room temperature under N_2 for 16 h, then diluted with EtOAc and washed with H_2O, a saturated aqueous $NaHCO_3$ solution and a saturated aqueous NaCl solution. The organic layer was evaporated *in vacuo* and the crude product was purified by silica gel column chromatography (pentane/EtOAc, 100:0 to 85:35) to give the product as a clear oil in quantitative yield. **^{1}H NMR** (400 MHz, $CDCl_3$) δ 7.33–7.23 (m, 2H), 7.22–7.11 (m, 2H), 6.16 (t, J = 4.0 Hz, 1H), 3.87 (s, 3H), 2.90 (dt, J = 16.5, 4.8 Hz, 1H), 2.83–2.72 (m, 1H), 2.19–1.97 (m, 3H), 1.93–1.81 (m, 1H); **^{13}C NMR** (101 MHz, $CDCl_3$) δ 158.6, 157.6, 138.3, 132.9, 129.9, 129.4, 128.9, 126.4, 73.8, 53.6, 28.9, 28.9, 18.7; **IR**: 2963, 2362, 1741, 1653, 1495, 1454, 1394, 1316, 1199, 1166, 1153, 1058, 1002, 976, 942, 908, 885, 871, 831, 762, 737, 655; **HRMS** (ESI-TOF) calculated for $C_{13}H_{14}O_4$ $[M+H^+]^+$: 235.0965, found 235.0853.

Methyl (1*R*,4a*S*,10a*R*)-7-isopropyl-1,4a-dimethyl-9-((2,2,6,6-tetramethylpiperidin-1-yl)oxy)-1,2,3,4,4a,9,10,10a-octahydrophenanthrene-1-carboxylate (S25)

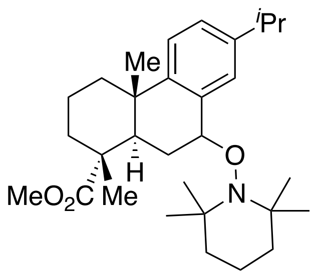

Synthesized according to literature procedure from methyl dehydroabietate.[16] All spectroscopic data were in accordance with the literature.[16]

5-Phenyl-5-((2,2,6,6-tetramethylpiperidin-1-yl)oxy)pentan-1-ol (S26)

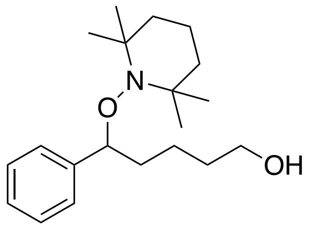

Synthesized according to literature procedure from methyl 5-bromo-5-phenylpentanoate.[2] All spectroscopic data were in accordance with the literature.[2]

1-((5-Bromo-1-phenylpentyl)oxy)-2,2,6,6-tetramethylpiperidine (S27)

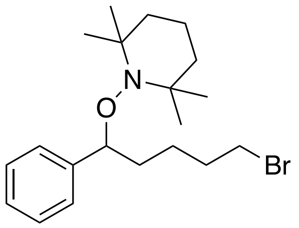

To a flame-dried round bottom flask equipped with a magnetic stirrer were added 5-phenyl-5-((2,2,6,6-tetramethylpiperidin-1-yl)oxy)pentan-1-ol **S26** (249 mg, 0.78 mmol, 1.0 equiv), CBr_4 (311 mg, 0.94 mmol, 1.2 equiv) and PPh_3 (246 mg, 0.94 mmol, 1.2 equiv). The flask was evacuated and backfilled with N_2 three times prior to the addition of anhydrous CH_2Cl_2 (2.6 mL, 0.3 M). The reaction mixture was stirred at room temperature under N_2 for 3 h, then diluted with pentane and additionally stirred for 5 minutes. The reaction mixture was then filtered over

a short celite pad and washed with CH_2Cl_2. The filtrate was evaporated *in vacuo* and the crude product was purified by silica gel column chromatography (pentane/EtOAc, 100:0 to 98:2) to give the product as a clear oil (220 mg, 0.58 mmol, 74%). **^{1}H NMR** (500 MHz, $CDCl_3$) δ 7.34–7.21 (m, 5H), 4.59 (dd, J = 9.8, 4.0 Hz, 1H), 3.35–3.26 (m, 2H), 2.15–2.03 (m, 1H), 1.89–1.71 (m, 3H), 1.62–1.09 (m, 14H), 1.00 (brs, 3H), 0.56 (brs, 3H); **^{13}C NMR** (126 MHz, $CDCl_3$) δ 143.5, 128.0, 127.8, 127.2, 87.2, 60.1 (br), 59.6 (br), 40.6, 35.2, 34.5 (br), 34.2 (br), 33.7, 33.0, 24.1, 20.5 (br), 17.3; **IR**: 3437, 3003, 2933, 2869,1944,1604, 1494, 1455, 1375, 1361, 1298, 1257, 1242, 1208, 1183, 1134, 1044, 1004, 974, 958, 917, 878, 788, 761, 735, 700, 646, 629; **HRMS** (ESI-TOF) calculated for $C_{20}H_{32}BrNO$ $[M+H^+]^+$: 382.1740, found 382.1735.

5-Phenyl-5-((2,2,6,6-tetramethylpiperidin-1-yl)oxy)pentyl 4-methylbenzenesulfonate (S28)

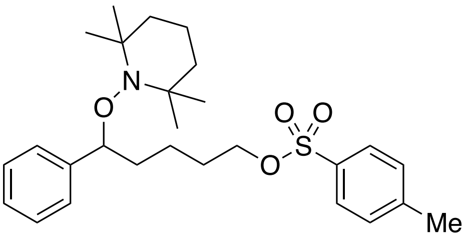

To a flame-dried round bottom flask equipped with a magnetic stirrer were added 5-phenyl-5-((2,2,6,6-tetramethylpiperidin-1-yl)oxy)pentan-1-ol **S26** (399 mg, 1.25 mmol, 1.0 equiv), TsCl (263 mg, 1.38 mmol, 1.1 equiv), and DMAP (15 mg, 0.1 equiv, 125 µmol, 0.1 equiv). The flask was then evacuated and backfilled with N_2 three times prior to the addition of anhydrous CH_2Cl_2 (4.2 mL, 0.3 M) and anhydrous NEt_3 (262 µL, 1.88 mmol, 1.5 equiv). The reaction mixture was stirred at room temperature under N_2 for 16 h, then quenched by the addition of H_2O and extracted twice with CH_2Cl_2. The combined organic layers were washed with a saturated aqueous NaCl solution, dried over Na_2SO_4 and evaporated *in vacuo*. The crude product was purified by silica gel column chromatography (pentane/EtOAc, 100:0 to 95:5) to give the product as a clear oil in quantitative yield. **^{1}H NMR** (400 MHz, $CDCl_3$) δ 7.75–7.70 (m, 2H), 7.34–7.18 (m, 7H), 4.53 (dd, J = 9.7, 4.0 Hz, 1H), 3.92 (td, J = 6.6, 2.6 Hz, 2H), 2.44 (s, 3H), 2.07–1.95 (m, 1H), 1.81–1.68 (m, 1H), 1.65–0.87 (m, 19H), 0.53 (brs, 3H); **^{13}C NMR** (101 MHz, $CDCl_3$) δ 144.7, 143.4, 133.3, 129.9, 128.0, 128.0, 127.8, 127.2, 87.1, 70.6, 60.0 (br), 59.6 (br), 40.5, 35.3, 34.5 (br), 34.1 (br), 29.0, 21.8, 21.3, 20.4 (br), 17.3; **HRMS** (ESI-TOF) calculated for $C_{27}H_{39}NO_4S$ $[M+H^+]^+$: 474.2673, found 474.2661; **IR**: 2976, 2934, 2361,

2341, 1613, 1591, 1448, 1376, 1363, 1257, 1216, 1165, 1134, 1064, 1015, 957, 934, 885, 849, 796, 699, 636.

2,3,5,6-Tetrafluorophenyl 5-phenyl-5-((2,2,6,6-tetramethylpiperidin-1-yl)oxy)pentanoate (S29)

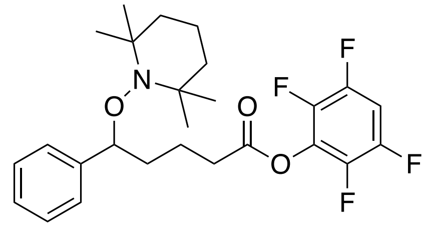

To a flame-dried round bottom flask equipped with a magnetic stirrer were added 5-phenyl-5-((2,2,6,6-tetramethylpiperidin-1-yl)oxy)pentanoic acid **S29a** (193 mg, 0.58 mmol, 1 equiv), 2,3,5,6-tetrafluorophenol (125 mg, 0.75 mmol, 1.3 equiv), DCC (155 mg, 0.75 mmol, 1.3 equiv), and DMAP (3.5 mg, 29 µmol, 0.05 equiv). The flask was evacuated and backfilled with N_2 three times prior to the addition of anhydrous CH_2Cl_2 (2.3 mL, 0.25 M). The reaction mixture was stirred at room temperature for 16 h, then filtered over a short celite pad with the aid of some CH_2Cl_2 and EtOAc. The filtrate was evaporated *in vacuo* and the crude product was purified by silica gel column chromatography twice (pentane/EtOAc, 100:0 to 99:1; then CH_2Cl_2, (100%)) to give the pure product as a clear oil (192 mg, 0.40 mmol, 69%). **^{1}H NMR** (500 MHz, $CDCl_3$) δ 7.36–7.28 (m, 4H), 7.28–7.23 (m, 1H), 6.97 (tt, J = 9.9, 7.1 Hz, 1H), 4.65 (dd, J = 9.6, 4.0 Hz, 1H), 2.59 (t, J = 7.4 Hz, 2H), 2.28–2.16 (m, 1H), 2.00–1.89 (m, 1H), 1.65–1.43 (m, 5H), 1.42–1.24 (m, 6H), 1.19 (brs, 3H), 1.01 (brs, 3H), 0.57 (brs, 3H); **^{13}C NMR** (126 MHz, $CDCl_3$) δ 169.4, 146.1 (dtd, J = 248.4, 12.0, 4.1 Hz), 143.2, 140.7 (dddd, J = 250.2, 15.5, 4.6, 2.0 Hz), 129.8 (tt, J = 14.1, 3.8 Hz), 128.2, 127.8, 127.4, 103.2 (t, J = 22.9 Hz), 87.0, 60.1 (br), 59.7 (br), 40.6, 35.1, 34.5 (br), 34.2 (br), 33.5, 20.8, 20.5 (br), 17.3; **^{19}F NMR** (377 MHz, $CDCl_3$) δ -139.05 – -139.22 (m, FH), -152.86 – -153.09 (m, FH); **HRMS** (ESI-TOF) calculated for $C_{26}H_{31}F_4NO_3$ $[M+H^+]^+$: 482.2313, found 482.2303; **IR**: 3087, 3003, 2973, 2936, 2873, 2361, 2340, 1792, 1645, 1526, 1488, 1456, 1376, 1362, 1273, 1259, 1242, 1219, 1179, 1133, 1089, 1007, 956, 916, 878, 840, 760, 736, 701, 669, 640.

5-Phenyl-5-((2,2,6,6-tetramethylpiperidin-1-yl)oxy)pentanoic acid (**S29a**)

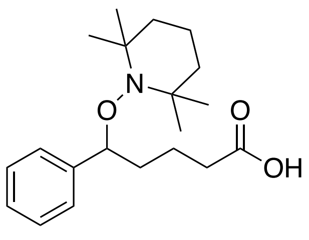

To a round bottom flask equipped with a magnetic stirrer was added methyl 5-phenyl-5-((2,2,6,6-tetramethylpiperidin-1-yl)oxy)pentanoate (792 mg, 2.28 mmol, 1.0 equiv), which was synthesized according to literature procedure from methyl 5-bromo-5-phenylpentanoate,[2] followed by THF/H_2O/MeOH (9.1 mL, 2.5:2.5:1, 0.25 M) and NaOH (912 mg, 22.8 mmol, 10.0 equiv). The reaction mixture was stirred at 50 °C for 3 hrs, then washed with Et_2O three times. The aqueous layer was acidified to pH = 1 *via* the dropwise addition of an aqueous 1 M HCl solution, then extracted with CH_2Cl_2 five times. The combined organic layers were dried over Na_2SO_4 and evaporated *in vacuo*. The crude product was then purified by silica gel column chromatography (CH_2Cl_2/MeOH, 100:0 to 95:5) to give the product as a clear oil in quantitative yield. **^{1}H NMR** (400 MHz, $CDCl_3$) δ 10.41 (brs, 1H), 7.33–7.21 (m, 5H), 4.62 (dd, J = 9.7, 4.0 Hz, 1H), 2.36–2.18 (m, 2H), 2.17–2.05 (m, 1H), 1.94–1.79 (m, 1H), 1.65–1.22 (m, 11H), 1.17 (brs, 3H), 1.00 (brs, 3H), 0.55 (brs, 3H); **^{13}C NMR** (126 MHz, $CDCl_3$) δ 179.8, 143.3, 128.1, 127.8, 127.3, 87.0, 60.2 (br), 59.7 (br), 40.5, 35.3, 34.3 (br), 34.2, 20.7, 20.5 (br), 17.3; **HRMS** (ESI-TOF) calculated for $C_{20}H_{31}NO_3$ $[M-H^+]^-$: 332.2231, found 332.2227; **IR**: 3902, 2933, 2361, 2341, 1710, 1494, 1456, 1413, 1376, 1361, 1296, 1242, 1210, 1183, 1134, 1064, 1046, 973, 957, 917, 879, 761, 701, 669, 647, 629.

1-(1-(Benzofuran-2-yl)ethoxy)-2,2,6,6-tetramethylpiperidine (S30)

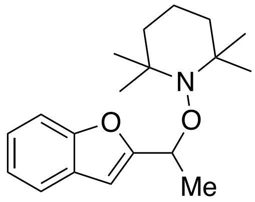

Synthesized according to general literature procedure B from 2-ethylbenzofuran. Purified by silica gel column chromatography (pentane/EtOAc, 100:0 to 99:1) and silica gel preparative thin layer chromatography (pentane/EtOAc, 98:2) to give the product as a clear oil (49 mg, 0.16 mmol, 5%). *Note: purification conditions were not optimised. Whilst the aminoxylation*

reaction was highly successful, this is not reflected in the isolated yield due to co-elution of the desired product with an uncharacterised impurity which resulted in the majority of the fractions being discarded in order to isolate the TEMPO-derived substrate in high purity. **^{1}H NMR** (400 MHz, $CDCl_3$) δ 7.56–7.46 (m, 2H), 7.28–7.16 (m, 2H), 6.61 (s, 1H), 4.93 (q, J = 6.8 Hz, 1H), 1.59 (d, J = 6.8 Hz, 3H), 1.56–1.43 (m, 3H), 1.36 (brs, 2H), 1.29 (brs, 4H), 1.18 (brs, 3H), 1.06 (brs, 3H), 0.62 (brs, 3H); **^{13}C NMR** (101 MHz, $CDCl_3$) δ 159.6, 154.7, 128.5, 123.9, 122.6, 121.0, 111.4, 104.1, 76.2, 60.4, 59.6, 40.3, 34.2, 32.8, 20.5 (br), 20.3 (br), 19.4, 17.3; **HRMS** (ESI-TOF) calculated for $C_{19}H_{28}NO_2$ $[M+H^+]^+$: 302.2115, found 302.2109; **IR**: 3007, 2976, 2933, 2872, 2848, 2361, 2341, 1692, 1599, 1586, 1455, 1376, 1361, 1299, 1277, 1254, 1209, 1185, 1154, 1134, 1097, 1065, 1046, 1008, 990, 944, 929, 881, 805, 788, 752, 741, 718, 668, 651.

6-chloro-9-methyl-2-(1-((2,2,6,6-tetramethylpiperidin-1-yl)oxy)ethyl)-9*H*-carbazole (S31)

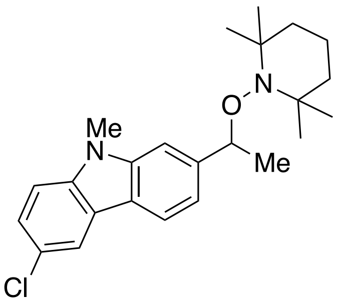

Synthesized according to general literature procedure C from 2-(6-chloro-9-methyl-9H-carbazol-2-yl)propanoic acid, which was synthesized according to literature procedure from Carprofen.[17] Purified by silica gel column chromatography (pentane/CH_2Cl_2, 100:0 to 70:30, then pentane/EtOAc, 100:0 to 80:20) to give the product as a white solid (110 mg, 0.28 mmol, 23%). **^{1}H NMR** (400 MHz, $CDCl_3$) δ 8.01 (d, J = 2.0 Hz, 1H), 7.97 (d, J = 8.0 Hz, 1H), 7.39 (dd, J = 8.6, 2.0 Hz, 1H), 7.34 (s, 1H), 7.29 (d, J = 8.6 Hz, 1H), 7.22 (dd, J = 8.1, 1.4 Hz, 1H), 4.96 (q, J = 6.7 Hz, 1H), 3.84 (s, 3H), 1.58 (d, J = 6.7 Hz, 4H), 1.52 (s, 2H), 1.35 (s, 6H), 1.22 (s, 3H), 1.05 (s, 3H), 0.62 (s, 3H); **^{13}C NMR** (101 MHz, $CDCl_3$) δ 145.2, 141.6, 139.7, 125.4, 124.4, 124.1, 120.8, 120.2, 120.0, 118.5, 109.4, 106.8, 84.1, 59.9 (br), 59.8 (br), 40.5, 34.6 (br), 34.4 (br), 29.4, 24.3, 20.5 (br), 17.4; **HRMS** (ESI-TOF) calculated for $C_{24}H_{31}ClN_2O$ $[M+H^+]^+$: 399.2198, found 399.2192; **IR**: 3003, 2973, 2932, 2361, 2341, 1630, 1604, 1515, 1470, 1423,

1377, 1360, 1285, 1244, 1219, 1186, 1136, 1062, 987, 955, 917, 879, 855, 819, 773, 706, 661, 627; **m.p.**: 141–142 °C.

5-(2-(4-(4-Fluorophenyl)-1*H*-1,2,3-triazol-1-yl)-1-((2,2,6,6-tetramethylpiperidin-1-yl)oxy)ethyl)-4-methylthiazole (S32)

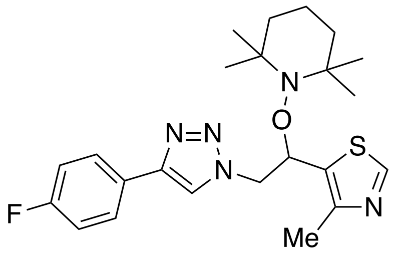

Synthesized according to literature procedure from 5-(2-azido-1-((2,2,6,6-tetramethylpiperidin-1-yl)oxy)ethyl)-4-methylthiazole,[18] which was synthesized according to literature procedure from 4-methyl-5-vinylthiazole.[18] **H NMR** (500 MHz, $CDCl_3$) δ 8.69 (s, 1H), 7.70 (dd, J = 8.9, 5.3 Hz, 2H), 7.37 (s, 1H), 7.09 (t, J = 8.7 Hz, 2H), 5.56 (dd, J = 8.0, 5.3 Hz, 1H), 5.21 (dd, J = 13.5, 5.6 Hz, 1H), 4.49 (dd, J = 13.6, 7.8 Hz, 1H), 2.16 (s, 3H), 1.67–1.31 (m, 6H), 1.27 (s, 3H), 1.13 (s, 3H), 1.05 (s, 3H), 0.79 (s, 3H); **^{13}C NMR** (126 MHz, $CDCl_3$) δ 162.8 (d, J = 247.0 Hz), 152.0, 151.9, 147.0, 130.3, 127.6 (d, J = 8.2 Hz), 126.7 (d, J = 3.2 Hz), 120.6, 116.0 (d, J = 21.3 Hz), 78.4, 60.6, 54.9, 40.6, 34.3 (br), 33.3 (br), 20.6 (br), 20.5 (br), 17.2, 15.4; **^{19}F NMR** (471 MHz, $CDCl_3$) δ -113.48 (td, J = 8.7, 4.3 Hz). All spectroscopic data were in accordance with the literature.[18]

2,2,6,6-Tetramethyl-1-((4-(methylthio)benzyl)oxy)piperidine (S33)

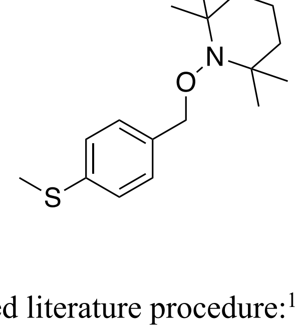

Synthesized according to an adapted literature procedure:[19]

To a flame-dried Schlenk tube equipped with a magnetic stirrer were added *o*-ethyl *S*-(4-(methylthio)benzyl) carbonodithioate **S33a** (155 mg, 0.6 mmol, 1.0 equiv) and TEMPO (141

mg, 0.9 mmol, 1.5 equiv). The tube vas evacuated and backfilled with N_2 three times prior to the addition of anhydrous PhCl (6.0 mL, 0.1 M) and TTMSS (370 µL, 1.2 mmol, 2.0 equiv). The reaction mixture was degassed by subjecting it to three freeze/pump/thaw cycles, then stirred at 100 °C under N_2. After 24 h, additional TEMPO (141 mg, 0.9 mmol, 1.5 equiv) and TTMSS (370 µL, 1.2 mmol, 2.0 equiv) were added and the reaction was stirred at 100 °C for further 24 h. Upon completion, the reaction mixture was diluted with Et_2O and washed with a saturated aqueous $Na_2S_2O_3$ solution, then a saturated aqueous NaCl solution. The organic layer was dried over Na_2SO_4, filtered over a short silica pad with the aid of additional EtOAc and evaporated *in vacuo*. The crude product was purified by silica gel column chromatography (pentane/EtOAc, 100:0 to 99:1) and preparative thin layer chromatography (pentane/EtOAc, 97:3) to give the product as a clear oil (135 mg, 0.46 mmol, 77%). The substrate could also be directly synthesized according to general literature procedure A from (4-(bromomethyl)phenyl)(methyl)sulfane and purified by silica gel column chromatography (pentane/EtOAc, 100:0 to 0:100) to give the product as a clear oil (1.087 g, 3.70 mmol, 95%). **^{1}H NMR** (500 MHz, $CDCl_3$) δ 7.32–7.23 (m, 4H), 4.79 (s, 2H), 2.49 (s, 3H), 1.65–1.45 (m, 5H), 1.40–1.32 (m, 1H), 1.26 (s, 6H), 1.15 (s, 6H); **^{13}C NMR** (126 MHz, $CDCl_3$) δ 137.4, 135.4, 128.2, 126.8, 78.5, 60.1, 39.8, 33.2, 20.4, 17.2, 16.2; **IR**: 3658, 3468, 3079, 2976, 2930, 2871, 2675, 2349, 1891, 1603, 1495, 1470, 1450, 1402, 1374, 1360, 1320, 1299, 1260, 1245, 1209, 1184, 1133, 1094, 1050, 1017, 992, 956, 924, 875, 853, 804, 758, 711, 649, 635; **HRMS** (ESI-TOF) calculated for $C_{17}H_{27}NOS$ $[M+H^+]^+$: 294.1886, found 294.1883.

<u>*O*-ethyl *S*-(4-(methylthio)benzyl) carbonodithioate</u> (**S33a**)

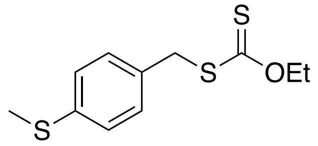

To a flame-dried round bottom flask equipped with a magnetic stirrer were added (4-(bromomethyl)phenyl)(methyl)sulfane (434 mg, 2.0 mmol, 1.0 equiv) and potassium ethyl xanthogenate (481 mg, 3.0 mmol, 1.5 equiv). The tube vas evacuated and backfilled with N_2 three times prior to the addition of anhydrous THF (20.0 mL, 0.1 M). The reaction mixture was stirred at room temperature under N_2 for 16 h, then diluted with CH_2Cl_2 and washed with a saturated aqueous NaCl solution. The organic layer was dried over Na_2SO_4 and evaporated *in vacuo*. The crude product was then purified by silica gel column chromatography

(pentane/EtOAc, 100:0 to 99:1, then pentane/CH_2Cl_2, 100:0 to 80:20) to give the product as a pale-yellow oil (391 mg, 1.51 mmol, 76%). **^{1}H NMR** (400 MHz, $CDCl_3$) δ 7.27 (d, J = 8.4 Hz, 2H), 7.20 (d, J = 8.3 Hz, 2H), 4.66 (q, J = 7.2 Hz, 2H), 4.33 (s, 2H), 2.47 (s, 3H), 1.42 (t, J = 7.2 Hz, 3H); **^{13}C NMR** (101 MHz, $CDCl_3$) δ 214.1, 138.0, 132.6, 129.7, 126.8, 70.2, 40.2, 16.0, 13.9; **IR**: 2983, 2919, 1600, 1494, 1439, 1405, 1362, 1220, 1146, 1111, 1094, 1048, 1016, 968, 812, 729, 630; **HRMS** (ESI-TOF) calculated for $C_{11}H_{14}OS_3$ $[M+H^+]^+$: 259.0280, found 259.0170.

1-Azido-4-(((2,2,6,6-tetramethylcyclohexyl)oxy)methyl)benzene (S34)

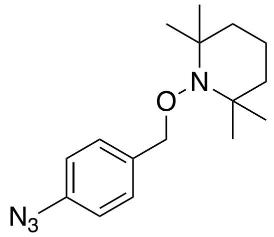

Synthesized according to general literature procedure A from 1-azido-4-(bromomethyl)benzene, which was synthesized according to literature procedure from (4-aminophenyl)methanol.[20] Purified by silica gel column chromatography (pentane/EtOAc, 100:0 to 2:98) to give the product as a pale-yellow oil (1.527 g, 5.30 mmol, 94 %). **^{1}H NMR** (400 MHz, $CDCl_3$) δ 7.36 (d, J = 8.7 Hz, 2H), 7.01 (d, J = 8.6 Hz, 2H), 4.81 (s, 2H), 1.70–1.31 (m, 6H), 1.27 (s, 6H), 1.17 (s, 6H); **^{13}C NMR** (101 MHz, $CDCl_3$) δ 139.1, 135.2, 129.1, 119.0, 78.3, 60.1, 39.9, 33.3, 20.4, 17.3; **HRMS** (ESI-TOF) calculated for $C_{16}H_{24}N_4O$ $[M+H^+]^+$: 289.2023, found 289.2017; **IR**: 3005, 2975, 2931, 2871, 2848, 2410, 2349, 2116, 1608, 1582, 1507, 1469, 1451, 1374, 1360, 1284, 1245, 1209, 1180, 1132, 1050, 992, 973, 956, 924, 875, 859, 827, 812, 774, 709, 647.

1-((4-Methoxybenzyl)oxy)-2,2,6,6-tetramethylpiperidine (S35)

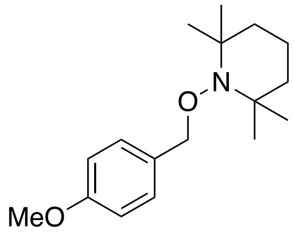

Synthesized according to an adapted literature procedure from 1-(4-methoxybenzyl)-2,4,6-triphenylpyridin-1-ium tetrafluoroborate,[21] which was synthesized according to literature procedure from (4-methoxyphenyl)methanamine:[22]

To a 20 mL oven-dried microwave vial equipped with a magnetic stirrer were added 1-(4-methoxybenzyl)-2,4,6-triphenylpyridin-1-ium tetrafluoroborate (258 mg, 0.5 mmol, 1.0 equiv), TEMPO (167 mg, 0.75 mmol, 1.5 equiv), and Mn (41 mg, 0.75 mmol, 1.5 equiv). The flask was evacuated and backfilled with N_2 three times prior to the addition of anhydrous *N*-methyl-2-pyrrolidone (NMP) (5.0 mL, 0.1 M). The reaction mixture was degassed by N_2 bubbling for 2 min, then stirred at 60 °C under N_2 for 16 h. Upon completion, the reaction mixture was filtered over a short silica pad with the aid of some EtOAc. The filtrate was then washed with a saturated aqueous NaCl solution three times. The organic layer was dried over Na_2SO_4 and evaporated *in vacuo.* The crude product was then purified by silica gel column chromatography (pentane/EtOAc, 100:0 to 99:1). Due to co-elution of the desired product with 2,4,6-triphenyl pyridine, the product was redissolved in a minimal amount of hexane and cooled to -78 °C to induce precipitation of the impurity, then filtered over a short cotton-plugged pipette. The procedure was repeated until no precipitate was observed. The solvent was then evaporated *in vacuo* to give the product as a clear oil (80 mg, 0.29 mmol, 58%). **^{1}H NMR** 7.32–7.27 (m, 2H), 6.91–6.85 (m, 2H), 4.74 (s, 2H), 3.61 (s, 3H), 1.67–1.41 (m, 5H), 1.39–1.30 (m, 1H), 1.26 (s, 6H), 1.13 (s, 6H); **^{13}C NMR** (101 MHz, $CDCl_3$) δ 159.1, 130.6, 129.3, 113.8, 78.6, 60.1, 55.4, 39.9, 33.3, 20.4, 17.3; **HRMS** (ESI-TOF) calculated for $C_{17}H_{27}NO_2$ $[M+H^+]^+$: 278.2115, found 278.2108; All spectroscopic data were in accordance with the literature.[23]

2,2,6,6-Tetramethyl-1-((2-phenylpropan-2-yl)oxy)piperidine (S36)

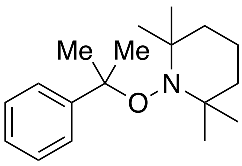

Synthesized according to literature procedure from cumene.[2] All spectroscopic data were in accordance with the literature.[2]

2,2,6,6-Tetramethyl-1-((1-phenylcyclohexyl)oxy)piperidine (S37)

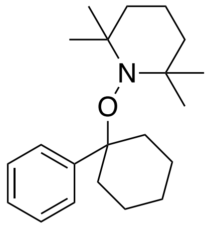

Synthesized according to general literature procedure B from cyclohexylbenzene. Purified by silica gel column chromatography (pentane/EtOAc, 100:0 to 98:2) to give the product as a white solid (473 mg, 1.50 mmol, 23%). **^{1}H NMR** (400 MHz, $CDCl_3$) δ 7.51 (d, J = 7.5 Hz, 2H), 7.29 (t, J = 7.6 Hz, 2H), 7.20 (t, J = 7.2 Hz, 1H), 2.27 (d, J = 12.8 Hz, 2H), 2.16–2.03 (m, 2H), 1.78–1.68 (m, 2H), 1.63–1.18 (m, 10H), 0.99 (s, 6H), 0.82 (s, 6H); **^{13}C NMR** (101 MHz, $CDCl_3$) δ 146.4, 128.3, 127.2, 126.4, 81.1, 59.5, 41.1, 35.6, 34.6, 26.3, 24.4, 20.6, 17.2; **HRMS** (ESI-TOF) calculated for $C_{21}H_{33}NO$ $[M+H^+]^+$: 316.2635, found 316.2632; **IR**: 3468, 3059, 2992, 2971, 2932, 2866, 2360, 2342, 1685, 1601, 1495, 1450, 1375, 1360, 1337, 1296, 1256, 1241, 1208, 1179, 1133, 1081, 1031, 1020, 9767, 957, 942, 927, 911, 877, 852, 810, 787, 761, 721, 700, 669, 639, 619; **m.p.**: 78–79 °C.

1-((5-(2,5-Dimethylphenoxy)-2-methylpentan-2-yl)oxy)-2,2,6,6-tetramethylpiperidine (S38)

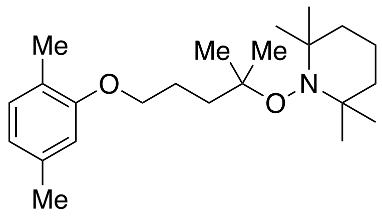

Synthesized according to an adapted literature procedure:[24]

To a flame-dried 10 mL microwave vial equipped with a magnetic stirrer were added mesityl-λ^3-iodanediyl bis(5-(2,5-dimethylphenoxy)-2,2-dimethylpentanoate **S38a** (186 mg, 0.25 mmol, 2.0 equiv) and TEMPO (78 mg, 0.5 mmol, 1.0 equiv). The tube vas evacuated and backfilled with N_2 three times prior to the addition of anhydrous $CHCl_3$ (1.7 mL, 0.15 M). The reaction mixture was degassed by bubbling with N_2 while stirring for 2 min, then stirred under the irradiation of a 40 W LED lamp (λ = 390 nm) at room temperature for 16 h. Upon

completion, the solvent was evaporated *in vacuo* and the crude product was purified by silica gel column chromatography (pentane/EtOAc, 100:0 to 99:1, then pentane/CH_2Cl_2, 100:0 to 50:50) to give the product as a clear oil (57 mg, 0.16 mmol, 32%). **^{1}H NMR** (400 MHz, $CDCl_3$) δ 7.01 (d, J = 7.5 Hz, 1H), 6.65 (d, J = 6.8 Hz, 2H), 3.96 (t, J = 6.3 Hz, 2H), 2.31 (s, 3H), 2.18 (s, 3H), 2.03–1.92 (m, 2H), 1.81–1.73 (m, 2H), 1.64–1.40 (m, 5H), 1.35–1.24 (m, 7H), 1.14 (s, 6H), 1.10 (s, 6H); **^{13}C NMR** (101 MHz, $CDCl_3$) δ 157.3, 136.6, 130.4, 123.7, 120.6, 112.0, 78.5, 68.6, 59.4, 41.0, 40.2, 34.9, 27.2, 24.5, 21.6, 20.9, 17.3, 16.0; **HRMS** (ESI-TOF) calculated for $C_{23}H_{39}NO_2$ $[M+H^+]^+$: 362.3054, found 362.3048; All spectroscopic data were in accordance with the literature.[5]

Mesityl-λ³-iodanediyl bis(5-(2,5-dimethylphenoxy)-2,2-dimethylpentanoate) (**S38a**)

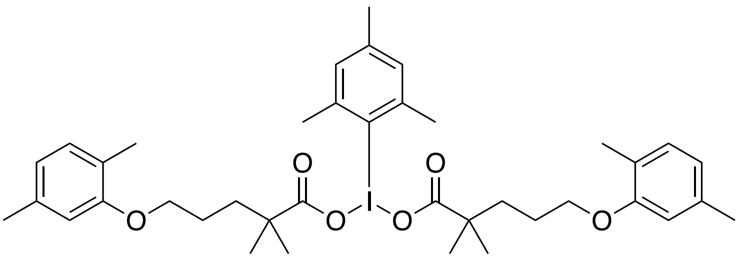

Synthesized according to an adapted literature procedure:[25]

To a flame-dried round bottom flask were added Gemfibrozil (401 mg, 1.6 mmol, 2.0 equiv) and iodomesitylene diacetate (291 mg, 0.8 mmol, 1.0 equiv), followed by anhydrous toluene (100 mL, 0.008 M). The solvent was then evaporated *in vacuo* at 50 °C. The addition of toluene and evaporation steps were repeated two more times (100 mL + 100 mL). Residual toluene was then evaporated under high vacuum to give the product as a clear oil (589 mg, 0.79 mmol, 99%). **^{1}H NMR** (400 MHz, $CDCl_3$) δ 7.00 (d, J = 7.9 Hz, 4H), 6.65 (d, J = 7.4 Hz, 2H), 6.56 (s, 2H), 3.77 (t, J = 5.5 Hz, 4H), 2.70 (s, 6H), 2.31 (s, 6H), 2.28 (s, 3H), 2.13 (s, 6H), 1.60–1.49 (m, 8H), 1.09 (s, 12H); **^{13}C NMR** (101 MHz, $CDCl_3$) δ 182.6, 157.1, 142.8, 141.3, 136.6, 130.9, 130.4, 128.8, 123.7, 120.7, 112.0, 68.2, 42.6, 37.8, 26.6, 25.9, 25.4, 21.6, 21.2, 15.9; **IR**: 2950, 2869, 2360, 2341, 1645, 1616, 1586, 1551, 1510, 1472, 1414, 1388, 1266, 1202, 1158, 1131, 1047, 999, 910, 848, 805, 736, 668, 648; **HRMS** (ESI-TOF) calculated for $C_{39}H_{53}IO_6$ $[M+Na^+]^+$: 767.2779, found 767.2772.

***tert*-Butyl ((1*r*,3*s*,5*R*,7*S*)-3-((2,2,6,6-tetramethylpiperidin-1-yl)oxy)adamantan-1-yl)carbamate (S39)**

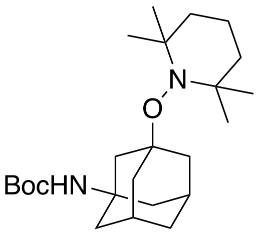

Synthesized according to an adapted literature procedure from *tert*-butyl ((1r,3s,5R,7S)-3-chloroadamantan-1-yl)carbamate **S39a**:[26]

To a flame-dried 10 mL microwave vial equipped with a magnetic stirrer were added *tert*-butyl ((1*r*,3*s*,5*R*,7*S*)-3-chloroadamantan-1-yl)carbamate **S39a** (174 mg, 0.61 mmol, 1.0 equiv), Cs_2CO_3 (398 mg, 1.22 mmol, 2.0 equiv), TEMPOH (115 mg, 0.73 mmol, 1.2 equiv), and 3,5-di-*tert*-butyl-[1,1'-biphenyl]-4-ol (17 mg, 61 μmol, 0.1 equiv). The tube vas evacuated and backfilled with N_2 three times prior to the addition of anhydrous and degassed DMF (6.1 mL, 0.1 M). The reaction mixture was stirred under the irradiation of an 18 W blue LED lamp (λ = 450 nm) at room temperature for 16 h. Upon completion, the reaction mixture was diluted with H_2O and extracted with EtOAc three times. The combined organic layers were washed with a saturated aqueous NaCl solution, dried over Na_2SO_4 and evaporated *in vacuo*. The crude product was purified by silica gel column chromatography (pentane/EtOAc, 100:0 to 99:1) to give the pure product as a white solid (64 mg, 0.16 mmol, 26%). **^{1}H NMR** (400 MHz, $CDCl_3$) *δ* 4.44 (brs, 1H), 2.21 (s, 2H), 2.08–1.61 (m, 12H), 1.56–1.34 (m, 16H), 1.30–1.21 (m, 1H), 1.15 (s, 5H), 1.06 (s, 5H); **^{13}C NMR** (101 MHz, $CDCl_3$) *δ* 154.1, 78.8, 77.4, 59.2, 53.7, 50.5, 47.4, 42.0, 41.4, 41.0, 40.8 (br), 36.4, 35.5, 35.3, 31.2, 29.5, 28.5, 20.6, 17.2; **IR**: 3902, 3853, 3838, 3821, 3735, 3690, 3675, 3649, 3629, 3263, 3126, 2980, 2972, 2918, 2360, 2341, 1693, 1653, 1636, 1558, 1540, 1497, 1474, 1457, 1389, 1363, 1329, 1307, 1278, 1254, 1176, 1147, 1051, 1031, 1008, 956, 943, 922, 883, 844, 772, 760, 736, 711, 669, 648, 617; **HRMS** (ESI-TOF) calculated for $C_{24}H_{42}N_2O_3$ $[M+H^+]^+$: 407.3268, found 407.3269; **m.p.**: 124–126 °C.

<u>*tert*-Butyl ((1r,3s,5R,7S)-3-chloroadamantan-1-yl)carbamate</u> (**S39a**)

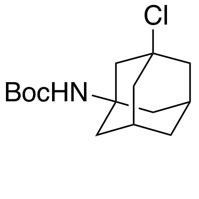

To a flame-dried round bottom flask equipped with a magnetic stirrer were added 3-chloro-1-aminoadamantane hydrochloride (225 mg, 1.0 mmol, 1.0 equiv), Boc_2O (327 mg, 1.5 mmol, 1.5 equiv), and DMAP (6 mg, 50 μmol, 0.05 equiv). The flask vas evacuated and backfilled with N_2 three times prior to the addition of anhydrous CH_2Cl_2 (5.0 mL, 0.2 M) and anhydrous NEt_3 (418 μL, 3.0 mmol, 3.0 equiv). The reaction mixture was stirred at room temperature for 16 h under N_2, then evaporated *in vacuo*. The crude product was purified by silica gel column chromatography (pentane/EtOAc, 100:0 to 80:20) to give the pure product as a white solid (179 mg, 0.63 mmol, 63%). **^{1}H NMR** (500 MHz, $CDCl_3$) δ 4.43 (brs, 1H), 2.33 (s, 2H), 2.28–2.22 (m, 2H), 2.06 (d, J = 3.5 Hz, 4H), 1.94 (d, J = 12.5 Hz, 2H), 1.81 (d, J = 11.9 Hz, 2H), 1.58 (s, 2H), 1.43 (s, 9H); **^{13}C NMR** (126 MHz, $CDCl_3$) δ 154.1, 79.3, 67.3, 53.1, 51.4, 46.6, 40.3, 34.6, 31.7, 28.6; **IR**: 3362, 3258, 3132, 2971, 2929, 2860, 2361, 2342, 1690, 1653, 1559, 1476, 1457, 1392, 1364, 1351, 1328,1302, 1255, 1172, 1147, 1047, 1027, 1007, 984, 967, 932, 863, 838, 780, 758, 706, 668, 636; **HRMS**: (ESI-TOF) calculated for $C_{15}H_{24}ClNO_2$ $[M+H^+]^+$: 286.1568, found 286.1563; **m.p.**: 102–103 °C.

1-((1,4-Dioxan-2-yl)oxy)-2,2,6,6-tetramethylpiperidine (S40)

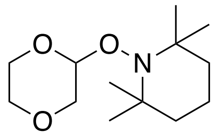

Synthesized according to general literature procedure B from 1,4-dioxane. All spectroscopic data were in accordance with the literature.[3]

3-Fluoro-4-(4-(1-((2,2,6,6-tetramethylpiperidin-1-yl)oxy)ethoxy)phenoxy)benzonitrile (S41)

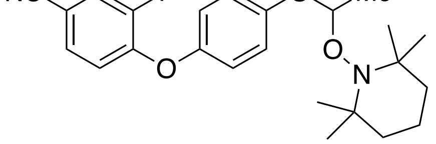

Synthesized according to general literature procedure D from 1,3-dioxoisoindolin-2-yl 2-(4-(4-cyano-2-fluorophenoxy)phenoxy)propanoate, which was synthesized according to literature

procedure from Cyhalofop.[27] Purified by silica gel column chromatography (pentane/EtOAc, 100:0 to 90:10) and preparative thin layer chromatography (pentane/EtOAc, 90:10) to give the product as a clear oil (74 mg, 0.18 mmol, 53%). **^{1}H NMR** (500 MHz, $CDCl_3$) δ 7.45 (dd, J = 10.1, 2.0 Hz, 1H), 7.34 (dt, J = 8.5, 1.7 Hz, 1H), 7.10–7.04 (m, 2H), 7.00–6.96 (m, 2H), 6.89 (t, J = 8.3 Hz, 1H), 5.54 (q, J = 5.3 Hz, 1H), 1.65–1.39 (m, 8H), 1.37–1.30 (m, 1H), 1.22–1.09 (m, 12H); **^{13}C NMR** (126 MHz, $CDCl_3$) δ 155.1, 152.5 (d, J = 251.6 Hz), 150.9 (d, J = 10.4 Hz), 148.3, 129.5 (d, J = 4.1 Hz), 121.0, 120.6 (d, J = 21.3 Hz), 119.0, 118.6 (d, J = 2.3 Hz), 117.9 (d, J = 2.7 Hz), 106.0, 105.9, 61.0, 59.6, 40.5, 40.1, 33.5, 33.4, 20.7, 20.3, 19.4, 17.3; **^{19}F NMR** (377 MHz, $CDCl_3$) δ -130.24 (t, J = 9.1 Hz); **IR**: 3853, 3726,3436, 3011, 2935, 2873, 2360, 2342,2235, 1748, 1617, 1584, 1501, 1457, 1426, 1378, 1363, 1285, 1224, 1193, 1156, 1133, 1097, 1064, 946, 917, 862, 846, 763, 718, 683; **HRMS** (ESI-TOF) calculated for $C_{24}H_{29}FN_2O_3$ $[M+H^+]^+$: 413.2235, found 413.2229.

2,2,6,6-Tetramethyl-1-((phenylthio)methoxy)piperidine (S42)

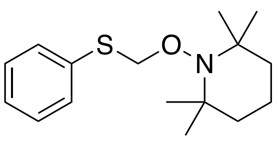

Synthesized according to an adapted literature procedure from potassium trifluoro((phenylthio)methyl)borate,[23] which was synthesized according to literature procedure from 4,4,5,5-tetramethyl-2-((phenylthio)methyl)-1,3,2-dioxaborolane:[28]

To a flame-dried 20 mL microwave vial equipped with a magnetic stirrer were added potassium trifluoro((phenylthio)methyl)borate (230 mg, 1.0 mmol, 1.0 equiv), TEMPO (313 mg, 2.0 mmol, 2.0 equiv), and $Ir[dFCF_3(ppy)_2bpy]PF_6$ (10 mg, 0.01 mmol, 0.01 equiv). The flask was evacuated and backfilled with N_2 three times prior to the addition of acetone (10.0 mL, 0.1 M). The vial was sealed with a septum, degassed with nitrogen bubbling for 10 seconds and wrapped in parafilm. The reaction mixture was stirred in a SynLED Parallel Photoreactor with an EvoluChem 18 W blue LED lamp (λ = 450 nm) at room temperature (with a fan) for 16 h. Upon completion, volatiles were evaporated *in vacuo* and the crude product was purified by silica gel column chromatography (pentane/EtOAc, 100:0 to 98:2) to give the product as a clear oil (225 mg, 0.81 mmol, 81%). **^{1}H NMR** (400 MHz, $CDCl_3$) δ 7.57–7.50 (m, 2H), 7.33–7.27 (m, 2H), 7.26–7.20 (m, 1H), 5.07 (s, 2H), 1.67–1.21 (m, 6H), 1.13 (s, 12H); **^{13}C NMR** (101 MHz, $CDCl_3$) δ 136.4, 130.9, 128.9, 126.9, 80.5, 60.1, 39.8, 33.4, 20.3, 17.2; **HRMS** (ESI-

TOF) calculated for $C_{16}H_{25}NOS$ $[M+H^{+}]^{+}$: 280.1730, found 280.1725. All spectroscopic data were in accordance with the literature.[29]

2-(2-Phenyl-1-((2,2,6,6-tetramethylpiperidin-1-yl)oxy)ethyl)isoindoline-1,3-dione (S43)

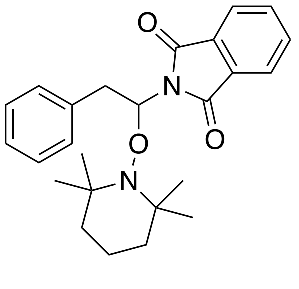

Synthesized according to literature procedure from *N*-phthaloyl-L-phenylalanine.[5] All spectroscopic data were in accordance with the literature.[5]

1-(Cyclohex-2-en-1-yloxy)-2,2,6,6-tetramethylpiperidine (S44)

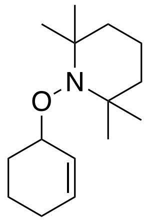

Synthesized according to general literature procedure B from cyclohexene. All spectroscopic data were in accordance with the literature.[3]

1,2-Bis(4-methoxyphenyl)-2-((2,2,6,6-tetramethylpiperidin-1-yl)oxy)ethan-1-one (S45)

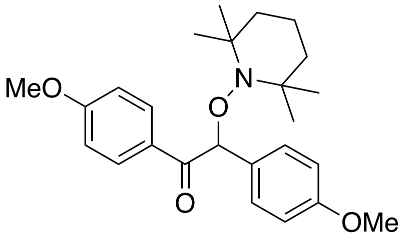

Synthesized according to an adapted literature procedure:[30]

To a flame-dried round bottom flask equipped with a magnetic stirrer was added desoxyanisoin (256 mg, 1.0 mmol, 1.0 equiv). The tube vas evacuated and backfilled with N_2 three times prior to the addition of anhydrous THF (5.0 mL, 0.2 M). LiHMDS (1 M solution in THF, 1.2 mL, 1.2 mmol, 1.2 equiv) was then added dropwise at -78 °C. The reaction mixture was stirred at -78 °C under N_2 for 30 min, then TEMPO (156 mg, 1.0 mmol, 1.0 equiv) was added as a solid, followed by PIDA (322 mg, 1.0 mmol, 1.0 equiv). The reaction was then allowed to reach room temperature and stirred for 16 h. Upon completion, the reaction mixture was diluted with a saturated aqueous NaCl solution and extracted with EtOAc three times. The combined organic layers were dried over Na_2SO_4 and evaporated *in vacuo.* The crude product was purified by silica gel column chromatography (pentane/EtOAc, 100:0 to 90:10) to give the pure product as a pale-yellow oil (141 mg, 0.34 mmol, 34%). **^{1}H NMR** (500 MHz, $CDCl_3$) δ 8.09 (d, J = 9.0 Hz, 2H), 7.42 (d, J = 8.7 Hz, 2H), 6.89 (d, J = 9.0 Hz, 2H), 6.83 (d, J = 8.7 Hz, 2H), 5.90 (s, 1H), 3.83 (s, 3H), 3.75 (s, 3H), 1.64–1.25 (m, 6H), 1.19 (brs, 6H), 1.01 (brs, 3H), 0.83 (brs, 3H); **^{13}C NMR** (126 MHz, $CDCl_3$) δ 196.8, 163.0, 158.7, 131.4, 130.1, 128.2, 128.0, 113.6, 113.4, 92.5, 59.8, 59.5, 55.1, 54.9, 40.0, 33.6, 33.1, 20.2, 20.1, 16.8; **HRMS** (ESI-TOF) calculated for $C_{25}H_{33}NO_4$ $[M+H^+]^+$: 412.2482, found 412.2475. All spectroscopic data were in accordance with the literature.[31]

4-(4-(1-((2,2,6,6-Tetramethylpiperidin-1-yl)oxy)ethyl)phenethoxy)quinazoline (S46)

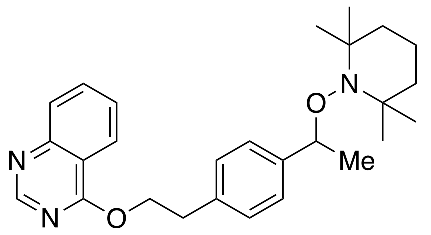

To a flame-dried round bottom flask equipped with a magnetic stirrer were added 2-(4-(1-((2,2,6,6-tetramethylpiperidin-1-yl)oxy)ethyl)phenyl)ethan-1-ol **S46a** (214 mg, 0.7 mmol, 1.0 equiv), 4-chloroquinazoline (173 mg, 1.05 mmol, 1.5 equiv), and Cs_2CO_3 (456 mg, 1.4 mmol, 2.0 equiv). The flask was evacuated and backfilled with N_2 three times prior to the addition of anhydrous MeCN (7.0 mL, 0.1 M). The reaction mixture was stirred at 80 °C for 48 h, then a solution of 4-chloroquinazoline (58 mg, 0.35 mmol, 0.5 equiv) in anhydrous MeCN (5.0 mL) was further added and the reaction was stirred at 80 °C for 16 h. Upon completion, the reaction mixture was diluted with a saturated aqueous NaCl solution and extracted with EtOAc twice.

The combined organic layers were dried over Na_2SO_4 and evaporated *in vacuo*. The crude product was purified by silica gel column chromatography (pentane/EtOAc, 100:0 to 80:20) and preparative thin layer chromatography (pentane/EtOAc, 80:20) to give the product as a white solid (185 mg, 0.43 mmol, 61%). **^{1}H NMR** (400 MHz, $CDCl_3$) δ 8.79 (s, 1H), 8.13 (dd, J = 8.2, 1.0 Hz, 1H), 7.92 (d, J = 8.3 Hz, 1H), 7.82 (ddd, J = 8.5, 6.9, 1.5 Hz, 1H), 7.55 (ddd, J = 8.2, 7.0, 1.3 Hz, 1H), 7.27 (s, 4H), 4.83–4.72 (m, 3H), 3.20 (t, J = 7.0 Hz, 2H), 1.47 (d, J = 6.7 Hz, 6H), 1.35 (brs, 2H), 1.28 (brs, 4H), 1.15 (brs, 3H), 1.01 (brs, 3H), 0.62 (brs, 3H); **^{13}C NMR** (101 MHz, $CDCl_3$) δ 166.8, 154.5, 151.1, 144.3, 136.4, 133.6, 128.8, 127.8, 127.1, 127.0, 123.7, 116.8, 82.9, 67.7, 59.8 (br), 40.5, 35.1, 34.6 (br), 34.3 (br), 23.6, 20.4 (br), 17.4; **HRMS** (ESI-TOF) calculated for $C_{27}H_{35}N_3O_2$ $[M+H^+]^+$: 434.2802, found 434.2791; **IR**: 2977, 2937, 2361, 2342, 1620, 1572, 1496, 1458, 1427, 1360, 1297, 1282, 1258, 1185, 1159, 1134, 1094, 1063, 1019, 988, 971, 934, 905, 884, 829, 801, 768, 723, 685, 667, 642, 623; **m.p.**: 83–84 °C.

<u>2-(4-(1-((2,2,6,6-Tetramethylpiperidin-1-yl)oxy)ethyl)phenyl)ethan-1-ol</u> (**S46a**)

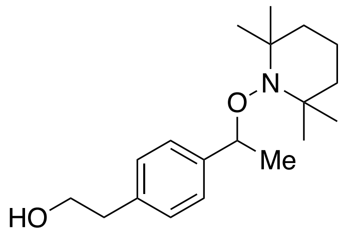

To a flame-dried round bottom flask equipped with a magnetic stirrer was added methyl 2-(4-(1-((2,2,6,6-tetramethylpiperidin-1-yl)oxy)ethyl)phenyl)acetate **S46b** (710 mg, 2.13 mmol, 1.0 equiv). The flask was evacuated and backfilled with N_2 three times prior to the addition of anhydrous THF (7.1 mL, 0.3 M). $LiAlH_4$ (121 mg, 3.20 mmol, 1.5 equiv) was then added at 0 °C. The reaction mixture was stirred at room temperature for one hour, then quenched with a few drops of H_2O and filtered over a short celite pad with the aid of some Et_2O. The filtrate was washed with a saturated aqueous NaCl solution, dried over Na_2SO_4 and evaporated *in vacuo*. The crude product was purified by silica gel column chromatography (pentane/EtOAc, 100:0 to 70:30) to give the product as a clear oil in quantitative yield. **^{1}H NMR** (500 MHz, $CDCl_3$) δ 7.27 (d, J = 8.7 Hz, 2H), 7.17 (d, J = 8.2 Hz, 2H), 4.77 (q, J = 6.6 Hz, 1H), 3.86 (t, J = 6.6 Hz, 2H), 2.86 (t, J = 6.5 Hz, 2H), 1.47 (d, J = 6.7 Hz, 6H), 1.38 (brs, 2H), 1.29 (brs, 4H), 1.16 (brs, 3H), 1.03 (brs, 3H), 0.67 (brs, 3H); **^{13}C NMR** (126 MHz, $CDCl_3$) δ 144.1, 136.8, 128.7, 127.0, 82.8, 63.8, 59.8, 40.4, 39.0, 34.5 (br), 34.2 (br), 23.5, 20.4 (br), 17.3; **HRMS**

(ESI-TOF) calculated for $C_{19}H_{31}NO_2$ [M+H$^+$]$^+$: 306.2428, found 306.2424; **IR**: 3342, 3006, 2974, 2932, 2870, 2360, 2341, 1631, 1513, 1468, 1377, 1362, 1259, 1243, 1219, 1182, 1134, 1064, 1046, 1020, 990, 957, 936, 884, 845, 825, 773, 669.

Methyl 2-(4-(1-((2,2,6,6-tetramethylpiperidin-1-yl)oxy)ethyl)phenyl)acetate (**S46b**)

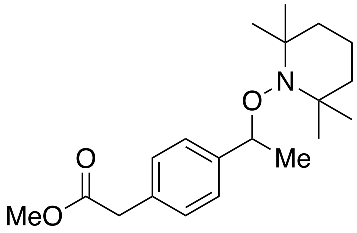

Synthesized according to general procedure B from methyl 2-(4-(1-bromoethyl)phenyl)acetate **S46c**. Purified by silica gel column chromatography (pentane/CH_2Cl_2, 100:0 to 40:60) to give the product as a white solid (762 mg, 2.29 mmol, 78%). **^{1}H NMR** (400 MHz, $CDCl_3$) δ 7.31 (d, J = 8.1 Hz, 2H), 7.25 (d, J = 7.8 Hz, 2H), 4.81 (q, J = 6.6 Hz, 1H), 3.73 (s, 3H), 3.65 (s, 2H), 1.50 (d, J = 6.6 Hz, 6H), 1.41 (brs, 2H), 1.31 (brs, 4H), 1.19 (brs, 3H), 1.06 (brs, 3H), 0.71 (brs, 3H); **^{13}C NMR** (101 MHz, $CDCl_3$) δ 172.3, 144.8, 132.4, 129.0, 126.9, 82.8, 59.8, 52.1, 41.0, 40.5, 34.6 (br), 34.3 (br), 23.6, 20.5 (br), 17.4; **HRMS** (ESI-TOF) calculated for $C_{20}H_{31}NO_3$ [M+H$^+$]$^+$: 334.2377, found 334.2370; **IR**: 3009, 2973, 2940, 2874, 2845, 1739, 1517, 1466, 1435, 1376, 1361, 1322, 1298, 1255, 1246, 1210, 1183, 1132, 1095, 1062, 1020, 1008, 986, 955, 932, 883, 844, 824, 785, 744, 717, 649, 632; **m.p.**: 69–70 °C.

Methyl 2-(4-(1-bromoethyl)phenyl)acetate (**S46bc**)

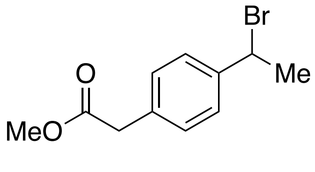

To a flame-dried round bottom flask equipped with a magnetic stirrer was added methyl 2-(4-(1-hydroxyethyl)phenyl)acetate **S46d** (699 mg, 3.60 mmol, 1.0 equiv) and the flask was evacuated and backfilled with N_2 three times prior to the addition of anhydrous CH_2Cl_2 (12.0 mL, 0.3 M). PBr_3 (508 µL, 5.40 mmol, 1.5 equiv) was then added dropwise at 0 °C. The reaction was stirred at room temperature under N_2 for 3 h, then quenched *via* the addition of a

saturated aqueous $NaHCO_3$ solution and extracted three times with CH_2Cl_2. The combined organic layers were washed with a saturated aqueous NaCl solution, dried over Na_2SO_4 and evaporated *in vacuo* to the give the product as a pale-yellow oil (897 mg, 3.49 mmol, 97%). **^{1}H NMR** (400 MHz, $CDCl_3$) δ 7.40 (d, J = 8.2 Hz, 2H), 7.26 (d, J = 8.5 Hz, 2H), 5.21 (q, J = 6.9 Hz, 1H), 3.70 (s, 3H), 3.62 (s, 2H), 2.04 (d, J = 6.9 Hz, 3H); **^{13}C NMR** (101 MHz, $CDCl_3$) δ 171.8, 142.2, 134.2, 129.7, 127.2, 52.2, 49.3, 41.0, 26.9; **HRMS**: not found; **IR**: 2992, 2952, 2360, 2342, 1738, 1614, 1515, 1437, 1377, 1342, 1258, 1223, 1162, 1067, 1044, 1019, 968, 896, 844, 822, 762, 730, 708, 668, 645.

Methyl 2-(4-(1-hydroxyethyl)phenyl)acetate (**S46d**)

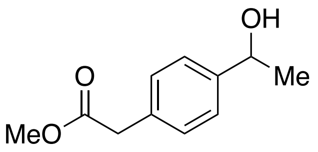

To a flame-dried round bottom flask equipped with a magnetic stirrer was added methyl 2-(4-formylphenyl)acetate (2.138 g, 12.0 mmol, 1.0 equiv). The flask was evacuated and backfilled with N_2 three times prior to the addition of anhydrous THF (60.0 mL, 0.2 M). MeMgBr (1.0 M solution in Et_2O, 12.0 mL, 12.0 mmol, 1.0 equiv) was then added dropwise at -78 °C. The reaction mixture was stirred at room temperature under N_2 for 16 h, then quenched *via* the addition of a saturated aqueous NH_4Cl solution and extracted with Et_2O twice. The combined organic layers were washed with a saturated aqueous NaCl solution, dried over Na_2SO_4 and evaporated *in vacuo*. The crude product was purified by silica gel column chromatography (pentane/EtOAc, 100:0 to 50:50) to give the product as a clear oil (760 mg, 3.91 mmol, 33%). **^{1}H NMR** (400 MH, $CDCl_3$) δ 7.32 (d, J = 8.3 Hz, 2H), 7.25 (d, J = 8.1 Hz, 2H), 4.87 (q, J = 6.6 Hz, 1H), 3.68 (s, 3H), 3.61 (s, 2H), 2.08–1-92 (m, 1H), 1.47 (d, J = 6.4 Hz, 3H); **^{13}C NMR** (101 MHz, $CDCl_3$) δ 172.2, 144.9, 133.2, 129.5, 125.8, 70.2, 52.2, 40.9, 25.2; **HRMS**: not found; **IR**: 3457, 2975, 2360, 2342, 1736, 1516, 1437, 1342, 1258, 1224, 1160, 1089, 1066, 1010, 955, 899, 844, 821, 668, 626.

2,2,6,6-Tetramethyl-1-(naphthalen-2-ylmethoxy)piperidine (S47)

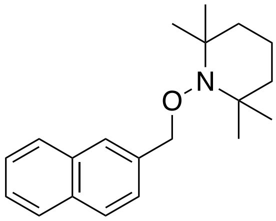

Synthesized according to literature procedure from 2-(bromomethyl)naphthalene.[32] All spectroscopic data were in accordance with the literature.[32]

1-((2,3-Dihydro-1*H*-inden-2-yl)oxy)-2,2,6,6-tetramethylpiperidine (S48)

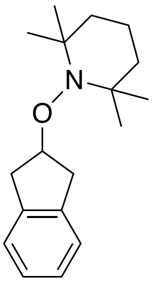

Synthesized according to literature procedure from 2-aminoindane.[33] All spectroscopic data were in accordance with the literature.[33]

1-(1-(4-Methoxyphenyl)ethoxy)-2,2,6,6-tetramethylpiperidine (S49)

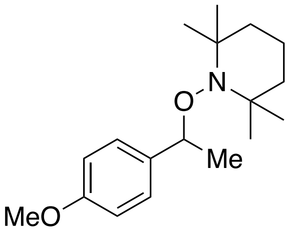

Synthesized according to literature procedure from 1-ethyl-4-methoxybenzene.[2] All spectroscopic data were in accordance with the literature.[2]

2,2,6,6-Tetramethyl-1-(1-(4-nitrophenyl)ethoxy)piperidine (S50)

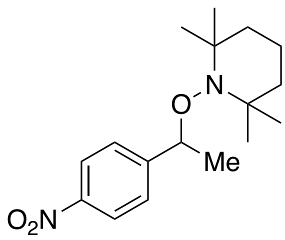

Synthesized according to general literature procedure A from 1-(1-bromoethyl)-4-nitrobenzene. Purified by silica gel column chromatography (pentane/EtOAc, 100:0 to 98:2) to give the product as a white solid (426 mg, 1.51 mmol, 35%). **^{1}H NMR** (400 MHz, $CDCl_3$) δ 8.20–8.14 (m, 2H), 7.47 (d, J = 8.8 Hz, 2H), 4.88 (q, J = 6.7 Hz, 1H), 1.59–1.42 (m, 6H), 1.37 (brs, 2H), 1.28 (brs, 4H), 1.17 (brs, 3H), 1.02 (brs, 3H), 0.61 (brs, 3H); **^{13}C NMR** (101 MHz, $CDCl_3$) 153.4, 147.0, 127.3, 123.6, 82.7, 59.9, 40.4, 34.5, 34.4, 23.7, 20.4, 17.2; **HRMS**: (ESI-TOF) calculated for $C_{17}H_{26}N_2O_3$ $[M+H^+]^+$: 307.2016, found 307.2007. All spectroscopic data were in accordance with the literature.[34]

3-(1-((2,2,6,6-Tetramethylpiperidin-1-yl)oxy)ethyl)pyridine (S51)

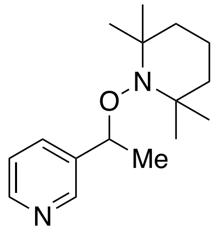

Synthesized according to an adapted literature procedure from 2,4,6-triphenyl-1-(1-(pyridin-3-yl)ethyl)pyridin-1-ium tetrafluoroborate,[21] which was synthesized according to literature procedure from 1-(pyridin-3-yl)ethan-1-amine:[35]

To a flame-dried round bottom flask equipped with a magnetic stirrer were added 2,4,6-triphenyl-1-(1-(pyridin-3-yl)ethyl)pyridin-1-ium tetrafluoroborate (500 mg, 1.0 mmol, 1.0 equiv), TEMPO (234 mg, 1.5 mmol, 1.5 equiv), and Mn (82 mg, 1.5 mmol, 1.5 equiv). The flask was evacuated and backfilled with N_2 three times prior to the addition of anhydrous *N*-methyl-2-pyrrolidone (NMP) (10.0 mL, 0.1 M). The reaction mixture was stirred at 60 °C under N_2 for 24 h, then filtered over a short silica pad with the aid of some EtOAc. The filtrate was washed with a saturated aqueous NaCl solution five times. The organic layer was then dried over Na_2SO_4 and evaporated *in vacuo*. The crude product was purified by silica gel column

chromatography (pentane/EtOAc, 100:0 to 80:20) and preparative thin layer chromatography (pentane/EtOAc, 75:25) to give the product as a pale-yellow oil (136 mg, 0.52 mmol, 52%). **^{1}H NMR** (400 MHz, $CDCl_3$) δ 8.55 (s, 1H), 8.49 (d, J = 3.1 Hz, 1H), 7.65 (d, J = 7.9 Hz, 1H), 7.25 (dd, J = 7.9, 4.8 Hz, 1H), 4.83 (q, J = 6.7 Hz, 1H), 1.50 (d, J = 6.7 Hz, 6H), 1.36 (brs, 2H), 1.30 (brs, 4H), 1.16 (brs, 3H), 1.01 (brs, 3H), 0.60 (brs, 1H); **^{13}C NMR** (101 MHz, $CDCl_3$) δ 148.6, 148.4, 140.7, 134.1, 123.2, 80.7, 59.9 (br), 59.7 (br), 40.3, 34.4 (br), 23.1, 20.3 (br), 17.2; **HRMS** (ESI-TOF) calculated for $C_{16}H_{26}N_2O$ $[M+H^+]^+$: 263.2118, found 263.2114; **IR**: 2975, 2933, 2872, 2365, 1741, 1591, 1577, 1473, 1425, 1376, 1362, 1303, 1258, 1242, 1210, 1183, 1133, 1067, 1045, 1025, 990, 975, 957, 935, 883, 812, 756, 715, 639.

Synthesis of reference compounds

1,2,3,4-tetrahydronaphthalen-1-ol, 1-(2-fluoro-[1,1'-biphenyl]-4-yl)ethan-1-ol, 1-(3-(trifluoromethoxy)phenyl)ethan-1-ol, 1-(4-bromophenyl)ethan-1-ol, 1-(benzofuran-2-yl)ethan-1-ol, 1-phenylethan-1-ol, 2,6-dihydroxypyrrolo[3,4-*f*]isoindole-1,3,5,7(2*H*,6*H*)-tetraone, 2-methyl-3-(trifluoromethyl)benzaldehyde, 4-(1-hydroxyethyl)benzoate, (4-methoxyphenyl)methanol, 4-vinylbenzaldehyde, cyclohex-2-en-1-ol, diphenylmethanol, fluoroiodomethane, methyl 5-phenylpentanoate, *tert*-butyl *N*-(adamantan-1-yl)carbamate, thiophenol, and 1-(bromomethyl)-4-methoxybenzene were purchased from commercial suppliers (Fluorochem, Sigma Aldrich, TCI, BLDpharm, Apollo Scientific, Ambeed, Manchester Organics, Biosynth) and used as received, without further purification.

Note: to prevent degradation of the products, samples of benzylic fluorides were evaporated and stored in plastic vessels.

General procedure E:

To a flame-dried Schlenk tube equipped with a magnetic stirrer was added the alcohol (1.0 equiv) and the flask was evacuated and backfilled with N_2 three times prior to the addition of anhydrous CH_2Cl_2 (0.3 M). The reaction mixture was then cooled to -78 °C prior to the dropwise addition of diethylminosulfur trifluoride (1.5 equiv), then allowed to reach room temperature and stirred under N_2 for 3 h. Upon completion, the reaction mixture was cooled to 0 °C and quenched *via* the dropwise addition of a saturated aqueous $NaHCO_3$ solution, then extracted with CH_2Cl_2 three times. The combined organic layers were washed with a saturated aqueous NaCl solution, then dried over Na_2SO_4 and evaporated *in vacuo*. The crude product was purified by silica gel column chromatography (pentane/EtOAc) to give the requisite product.

General procedure F:

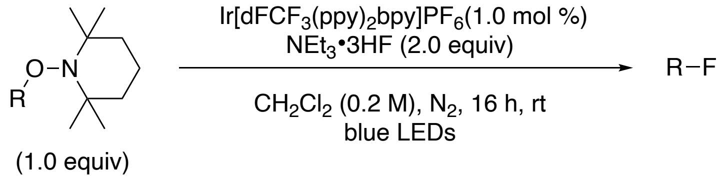

To an oven-dried 4 mL glass vial equipped with a magnetic stirrer was added TEMPO-derived substrate (1.0 equiv), followed by anhydrous CH_2Cl_2 (0.2 M), Ir[dFCF$_3$(ppy)$_2$bpy]PF$_6$ (1.0 mol%) and NEt$_3$•3HF (2.0 equiv). The vial was sealed with a septum, degassed with nitrogen bubbling for 10 seconds and wrapped in parafilm. The reaction mixture was stirred in a SynLED Parallel Photoreactor with an EvoluChem 18 W blue LED lamp (λ = 450 nm) at room temperature (with a fan) for 16 h. Upon completion, the reaction mixture was diluted with a saturated aqueous $NaHCO_3$ solution and extracted with CH_2Cl_2 three times. The combined organic layers were dried over Na_2SO_4 and evaporated *in vacuo*. The crude product was then purified by silica gel column chromatography (pentane/ethyl acetate) to give the requisite product.

Note: Ir[dFCF$_3$(ppy)$_2$bpy]PF$_6$ was elected over comparably performing Ir[dFFppy]$_2$-(4,4'-dCF$_3$bpy)PF$_6$ (see Table s5) due to its greater commercial availability and milder oxidising ability.

4-(1-((1,1,1,3,3,3-Hexafluoropropan-2-yl)oxy)ethyl)-1,1'-biphenyl (2)

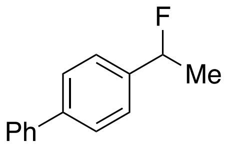

To an oven-dried 20 mL glass vial equipped with a magnetic stirrer was added CsF (152 mg, 1.0 mmol, 2.0 equiv) followed by HFIP (0.526 mL, 5.0 mmol, 10.0 equiv) and the suspension was sonicated until homogeneous. Subsequently, anhydrous and degassed CH_2Cl_2 was added (10.0 mL, 0.05 M) followed by Ir[dFFppy]$_2$-(4,4'-dCF$_3$bpy)PF$_6$ (5.2 mg, 5.0 μmol, 1.0 mol%) and 1-(1-([1,1'-biphenyl]-4-yl)ethoxy)-2,2,6,6-tetramethylpiperidine **1** (169 mg, 0.5 mmol, 1.0 equiv). The vial was sealed with a septum, degassed with nitrogen bubbling for 10 seconds and wrapped in parafilm. The reaction mixture was stirred in a SynLED Parallel Photoreactor with an EvoluChem 18 W blue LED lamp (λ = 450 nm) at room temperature (with a fan) for 16 h. Upon completion, the reaction mixture was diluted with a saturated aqueous $NaHCO_3$ solution and extracted with CH_2Cl_2 three times. The combined organic layers were dried over Na_2SO_4 and evaporated *in vacuo*. The crude product was then purified by silica gel flash chromatography (pentane/CH_2Cl_2, 100/0 to 80/20) to give the pure product as a white solid (50

mg, 0.25 mmol, 50%). **^{1}H NMR** (400 MHz, $CDCl_3$) δ 7.68–7.58 (m, 4H), 7.51–7.43 (m, 4H), 7.41–7.35 (m, 1H), 5.70 (dq, J = 47.7, 6.4 Hz, 1H), 1.71 (dd, J = 23.8, 6.5 Hz, 3H); **^{19}F NMR** (377 MHz, $CDCl_3$) δ -166.60 (dq, J = 48.2, 24.1 Hz); **^{13}C NMR** (101 MHz, $CDCl_3$) δ 141.3 (d, J = 2.4 Hz), 140.8, 140.5 (d, J = 19.5 Hz), 128.9, 127.6, 127.4, 127.3, 125.9 (d, J = 6.8 Hz), 90.9 (d, J = 167.3 Hz), 23.0 (d, J = 25.4 Hz). **HRMS** (EI) calculated for $C_4H_{13}F$ $[M]^{+}$: 200.09958, found 200.10013. All spectroscopic data were in accordance with the literature.[36]

4-(1-((1,1,1,3,3,3-hexafluoropropan-2-yl)oxy)ethyl)-1,1'-biphenyl and 4-vinyl-1,1'-biphenyl were also isolated as by-products:

4-(1-((1,1,1,3,3,3-Hexafluoropropan-2-yl)oxy)ethyl)-1,1'-biphenyl (2a)

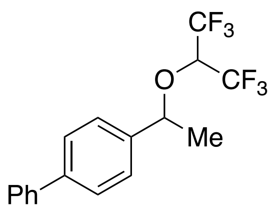

The product was obtained as a white solid (47 mg, 0.14 mmol, 27%). **^{1}H NMR** (400 MHz, $CDCl_3$) δ 7.68–7.58 (m, 4H), 7.52–7.35 (m, 5H), 4.91 (q, J = 6.4 Hz, 1H), 4.06 (hept, J = 6.0 Hz, 1H), 1.64 (d, J = 6.5 Hz, 3H); **^{13}C NMR** (101 MHz, $CDCl_3$) δ 142.1, 140.6, 138.6, 129.0, 127.8, 127.7, 127.3, 124.0–119.6 (m), 81.2, 73.0 (dt, J = 64.4, 32.2 Hz), 23.3; **^{19}F NMR** (377 MHz, $CDCl_3$) δ -72.82 (qd, J = 9.3, 6.0 Hz), -73.60 (qd, J = 9.5, 6.0 Hz); **HRMS**: not found. All spectroscopic data were in accordance with the literature.[37]

4-Vinyl-1,1'-biphenyl (2b)

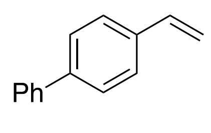

The product was obtained as a white solid (5 mg, 28 µmol, 6%). **^{1}H NMR** (400 MHz, $CDCl_3$) δ 7.64–7.55 (m, 4H), 7.53–7.41 (m, 4H), 7.38–7-32 (m, 1H), 6.77 (dd, J = 17.6, 10.9 Hz, 1H), 5.80 (dd, J = 17.6, 1.0 Hz, 1H), 5.28 (dd, J = 10.9, 1.0 Hz, 1H); **^{13}C NMR** (101 MHz, $CDCl_3$) δ 140.9, 140.7, 136.7, 136.5, 128.9, 127.5, 127.4, 127.1, 126.8, 114.1; **HRMS**: (EI) calculated

for $C_{14}H_{12}$ $[M]^+$: 180.09335, found 180.0939. All spectroscopic data were in accordance with the literature.[38]

(1-Fluoroethyl)benzene (5)

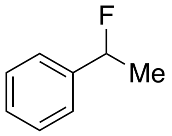

Synthesized according to general procedure E from 1-phenylethan-1-ol. Purified by silica gel column chromatography (pentane, 100%) to give the product as a clear oil (150 mg, 1.21 mmol, 60%). **^{1}H NMR** (500 MHz, $CDCl_3$) δ 7.43–7.30 (m, 5H), 5.64 (dq, J = 47.6, 6.3 Hz, 1H), 1.66 (dd, J = 23.9, 6.4 Hz, 3H); **^{13}C NMR** (126 MHz, $CDCl_3$) δ 141.6 (d, J = 19.5 Hz), 128.6, 128.4 (d, J = 2.3 Hz), 125.4 (d, J = 6.8 Hz), 91.1 (d, J = 167.6 Hz), 23.1 (d, J = 25.0 Hz); **^{19}F NMR** (471 MHz, $CDCl_3$) δ -167.06 (dq, J = 47.7, 24.3 Hz); **HRMS** (EI) calculated for C_8H_9F $[M]^+$: 124.06828, found 124.06883. All spectroscopic data were in accordance with the literature.[39]

1-(1-Fluoroethyl)-4-methylbenzene (7)

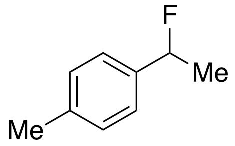

Synthesized according to general procedure E from 1-(*p*-tolyl)ethan-1-ol. Purified by silica gel column chromatography (pentane, 100%) to give the product as a clear oil (35 mg, 0.25 mmol, 50%). **^{1}H NMR** (500 MHz, $CDCl_3$) δ 7.26 (d, J = 7.0 Hz, 2H), 7.20 (d, J = 8.1 Hz, 2H), 5.61 (dq, J = 47.7, 7.0 Hz, 1H), 2.37 (s, 3H), 1.65 (dd, J = 23.8, 6.4 Hz, 3H); **^{13}C NMR** (126 MHz, $CDCl_3$) δ 138.6 (d, J = 19.5 Hz), 138.2 (d, J = 2.3 Hz), 129.3, 125.5 (d, J = 6.4 Hz), 91.1 (d, J = 166.2 Hz), 23.0 (d, J = 25.4 Hz), 21.30; **^{19}F NMR** (471 MHz, $CDCl_3$) δ -164.94 (dq, J = 46.8, 23.8 Hz); **HRMS** (EI) calculated for $C_9H_{11}F$ $[M]^+$: 138.08393, found 138.08201. All spectroscopic data were in accordance with the literature.[40]

1-(1-Fluoroethyl)-4-isobutylbenzene (8)

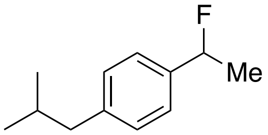

Synthesized according to general procedure F from 1-(1-(4-isobutylphenyl)ethoxy)-2,2,6,6-tetramethylpiperidine **S8**. Purified by silica gel column chromatography (pentane, 100%) to give the product as a clear oil (39 mg, 0.22 mmol, 43%). **^{1}H NMR** (400 MHz, $CDCl_3$) δ 7.28 (d, J = 8.1 Hz, 2H), 7.17 (d, J = 8.1 Hz, 2H), 5.62 (dq, J = 48.0, 6.5 Hz, 1H), 2.49 (d, J = 7.2 Hz, 2H), 1.88 (hept, J = 6.5 Hz, 1H), 1.65 (dd, J = 23.9, 6.5 Hz, 3H), 0.92 (d, J = 6.6 Hz, 6H); **^{13}C NMR** (101 MHz, $CDCl_3$) δ 142.01 (d, J = 2.4 Hz), 138.78 (d, J = 19.9 Hz), 129.34, 125.32 (d, J = 6.4 Hz), 91.16 (d, J = 166.5 Hz), 45.26, 30.36, 22.90 (d, J = 25.8 Hz), 22.50; **^{19}F NMR** (377 MHz, $CDCl_3$) δ -164.62 (dq, J = 47.7, 23.8 Hz); **HRMS** (EI) calculated for $C_{12}H_{17}F$ $[M]^+$: 180.13088, found 180.13080. All spectroscopic data were in accordance with the literature.[41]
Note: signs of decomposition were observed during purification of this substrate.

1-(1-Fluoroethyl)-4-vinylbenzene (9)

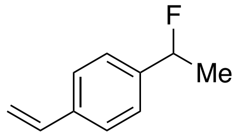

Synthesized according to general procedure E from 1-(4-vinylphenyl)ethan-1-ol **9a**. Purified by silica gel column chromatography (pentane, 100%) to give the product as a clear oil (52 mg, 0.35 mmol, 35%). **^{1}H NMR** (400 MHz, $CDCl_3$) δ 7.43 (d, J = 8.3 Hz, 2H), 7.32 (d, J = 7.2 Hz, 2H), 6.73 (dd, J = 17.6, 10.9 Hz, 1H), 5.77 (d, J = 17.6 Hz, 1H), 5.62 (dq, J = 47.5, 6.4 Hz, 1H), 5.27 (d, J = 10.9 Hz, 1H), 1.65 (dd, J = 23.8, 6.4 Hz, 3H); **^{13}C NMR** (126 MHz, $CDCl_3$) δ 141.1 (d, J = 19.5 Hz), 137.7 (d, J = 2.3 Hz), 136.5, 126.5, 125.6 (d, J = 6.4 Hz), 114.4, 90.9 (d, J = 167.6 Hz), 23.0 (d, J = 25.4 Hz); **^{19}F NMR** (377 MHz, $CDCl_3$) δ -166.83 (dq, J = 47.7, 23.6 Hz); **HRMS** (EI) calculated for $C_{10}H_{11}F$ $[M]^+$: 150.08393, found 150.08448; **IR**: 3091, 2984, 2932, 2360, 2341, 1912, 1829, 1631, 1570, 1514, 1452, 1408, 1376, 1349, 1319, 1296, 1284, 1261, 1217, 1183, 1098, 1068, 1005, 990, 911, 886, 843, 801, 759, 713, 646, 628.

<u>1-(4-Vinylphenyl)ethan-1-ol</u> (**9a**)

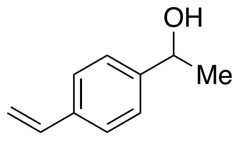

To a flame-dried round bottom flask equipped with a magnetic stirrer was added 4-vinylbenzaldehyde (500 mg, 3.78 mmol, 1.0 equiv). The flask was evacuated and backfilled with N_2 three times prior to the addition of anhydrous THF (10.8 mL, 0.35 M). MeMgBr (3.4 M solution in THF, 1.67 mL, 5.67 mmol, 1.5 equiv) was then added dropwise at -78 °C. The reaction mixture was stirred at room temperature under N_2 for 2 h, then quenched by the addition of a saturated aqueous NH_4Cl solution and extracted with Et_2O twice. The combined organic layers were washed with a saturated aqueous NaCl solution, dried over Na_2SO_4 and evaporated *in vacuo*. The crude product was purified by silica gel column chromatography (pentane/EtOAc, 100:0 to 90:10) to give the product as a clear oil (487 mg, 3.29 mmol, 87%). **^{1}H NMR** (400 MHz, $CDCl_3$) δ 7.43–7.39 (m, 2H), 7.33 (d, J = 8.3 Hz, 2H), 6.72 (dd, J = 17.6, 10.8 Hz, 1H), 5.75 (dd, J = 17.6, 1.0 Hz, 1H), 5.24 (dd, J = 10.8, 1.0 Hz, 1H), 4.89 (q, J = 6.4 Hz, 1H), 1.96–1.78 (brm, 1H), 1.49 (d, J = 6.6 Hz, 3H); **^{13}C NMR** (101 MHz, $CDCl_3$) δ 145.5, 137.0, 136.6, 126.5, 125.7, 113.9, 70.3, 25.2; **HRMS**: not found. All spectroscopic data were in agreement with the literature.[42]

1-(1-Fluoroethyl)-2-methyl-3-(trifluoromethyl)benzene (10)

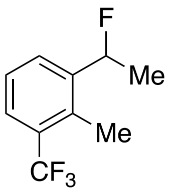

Synthesized according to general procedure E from 1-(2-methyl-3-(trifluoromethyl)phenyl)ethan-1-ol **10a**. Purified by silica gel column chromatography (pentane, 100%) to give the product as a clear oil (105 mg, 0.51 mmol, 26%). **^{1}H NMR** (500 MHz, $CDCl_3$) δ 7.63 (dd, J = 17.4, 7.8 Hz, 2H), 7.34 (t, J = 7.9 Hz, 1H), 5.93 (dq, J = 46.6, 6.4 Hz, 1H), 2.41 (s, 3H), 1.64 (dd, J = 23.9, 6.4 Hz, 3H); **^{13}C NMR** (126 MHz, $CDCl_3$) δ 142.0 (d, J = 19.1 Hz), 133.2–133.0 (m), 129.6 (q, J = 29.1 Hz), 128.4 (d, J = 10.0 Hz), 126.2, 125.8 (qd, J = 5.9, 2.3 Hz), 124.7 (q, J = 168.0 Hz) (overlapping peaks), 87.9 (d, J = 168.0 Hz), 22.2 (d, J = 25.4 Hz), 14.5 (q, J = 2.7 Hz); **^{19}F NMR** (377 MHz, $CDCl_3$) δ -60.46 (s, 3F), -171.05

(dq, J = 46.8, 23.4 Hz, 1F); **HRMS** (EI) calculated for $C_{10}H_{10}F_4$ $[M]^+$: 206.07131, found 206.07186; **IR**: 2991, 2877, 2362, 2341, 1712, 1667, 1621, 1548, 1467, 1449, 1377, 1358, 1317, 1282, 1266, 1206, 1172, 1129, 1099, 1019, 894, 804, 730, 638.

1-(2-Methyl-3-(trifluoromethyl)phenyl)ethan-1-ol (**10a**)

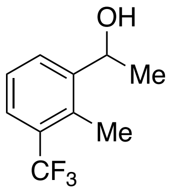

To a flame-dried round bottom flask equipped with a magnetic stirrer was added 2-methyl-3-(trifluoromethyl)benzaldehyde (800 mg, 4.25 mmol, 1.0 equiv). The flask was evacuated and backfilled with N_2 three times prior to the addition of anhydrous THF (12.1 mL, 0.35 M). MeMgBr (3.4 M solution in THF, 1.87 mL, 6.38 mmol, 1.5 equiv) was then added dropwise at -78 °C. The reaction mixture was stirred at room temperature under N_2 for 2 h, then quenched by the addition of a saturated aqueous NH_4Cl solution and extracted with Et_2O twice. The combined organic layers were washed with a saturated aqueous NaCl solution, dried over Na_2SO_4 and evaporated *in vacuo*. The crude product was purified by silica gel column chromatography (pentane/EtOAc, 100:0 to 0:100) to give the product as a clear oil in quantitative yield. **^{1}H NMR** (500 MHz, $CDCl_3$) δ 7.74 (d, J = 7.9 Hz, 1H), 7.55 (d, J = 7.8 Hz, 1H), 7.31 (t, J = 7.9 Hz, 1H), 5.23 (q, J = 6.4 Hz, 1H), 2.42 (s, 3H), 2.03 (brs, 1H), 1.46 (d, J = 6.4 Hz, 3H); **^{13}C NMR** (126 MHz, $CDCl_3$) δ 146.0, 133.1 (q, J = 1.7 Hz), 129.4 (q, J = 29.1 Hz), 128.4 (q, J = 1.3 Hz), 126.2, 125.0 (q, J = 5.9 Hz), 124.8 (q, J = 274.1 Hz), 66.5, 24.3, 14.3 (q, J = 2.4 Hz); **^{19}F NMR** (470 MHz, $CDCl_3$) δ -60.31 (s); **HRMS**: not found; **IR**: 3347, 2980, 2892, 2812, 2361, 2342, 1669, 1589, 1449, 1373, 1318, 1204, 1168, 1123, 1095, 1019, 901, 805, 730, 652.

2-Fluoro-4-(1-fluoroethyl)-1,1'-biphenyl (11)

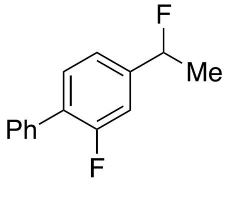

Synthesized according to general procedure E from 1-(2-fluoro-[1,1'-biphenyl]-4-yl)ethan-1-ol. Purified by silica gel column chromatography (pentane, 100%) to give the product as a clear oil. **^{1}H NMR** (500 MHz, $CDCl_3$) δ 7.55 (d, J = 7.3 Hz, 2H), 7.46 (t, J = 7.7 Hz, 3H), 7.38 (t, J = 7.4 Hz, 1H), 7.18 (t, J = 8.9 Hz, 2H), 5.66 (dq, J = 47.4, 6.5 Hz, 1H), 1.68 (dd, J = 23.9, 6.4 Hz, 3H); **^{13}C NMR** (126 MHz, $CDCl_3$) δ 159.9 (d, J = 248.9 Hz), 143.1 (dd, J = 20.2, 7.5 Hz), 135.5, 131.0 (d, J = 4.1 Hz), 129.1 (d, J = 2.7 Hz), 128.9 (dd, J = 13.6, 1.8 Hz), 128.6, 128.0, 121.2 (dd, J = 6.8, 3.6 Hz), 113.2 (dd, J = 24.3, 7.5 Hz), 90.1 (dd, J = 169.2, 1.6 Hz), 23.0 (d, J = 25.0 Hz); **^{19}F NMR** (470 MHz, $CDCl_3$) δ -117.43 – -117.38 (m, 1F), -168.33 (dq, J = 47.7, 23.4 Hz, 1F); **HRMS** (EI) calculated for $C_{14}H_{12}F_2$ $[M]^+$: 218.09016, found 218.09290; **IR** (neat): 3034, 2983, 2918, 2850, 2361, 2341, 1583, 1566, 1518, 1486, 1452, 1418, 1377, 1341, 1297, 1274, 1229, 1164, 1132, 1102, 1072, 1012, 937, 916, 869, 833, 768, 726, 698, 669, 649. All spectroscopic data were in accordance with the literature.[43]

1-(1-Fluoroethyl)-3-(trifluoromethoxy)benzene (12)

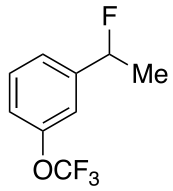

Synthesized according to general procedure E from 1-(3-(trifluoromethoxy)phenyl)ethan-1-ol. Purified by silica gel column chromatography (pentane, 100%) to give the product as a clear oil (131 mg, 0.63 mmol, 63%). **^{1}H NMR** (400 MHz, $CDCl_3$) δ 7.41 (t, J = 7.9 Hz, 1H), 7.27 (d, J = 7.2 Hz, 1H), 7.22 (s, 1H), 7.20–7.15 (m, 1H), 5.64 (dq, J = 47.4, 6.4 Hz, 1H), 1.65 (dd, J = 23.9, 6.4 Hz, 3H); **^{13}C NMR** (101 MHz, $CDCl_3$) δ 149.6 (d, J = 2.2 Hz), 144.1 (d, J = 20.0 Hz), 130.1, 123.6 (d, J = 6.9 Hz), 120.64, 120.61 (q, J = 257.2 Hz), 117.9 (d, J = 7.6 Hz), 90.2 (d, J = 169.7 Hz), 23.1 (d, J = 25.1 Hz); **^{19}F NMR** (377 MHz, $CDCl_3$) δ -57.83 (s, 3F), -169.44 (dq, J = 47.7, 24.1 Hz, 1F); **HRMS** (EI) calculated for $C_9H_8F_4O$ $[M]^+$: 208.05058, found 208.05113; **IR**: 2989, 2933, 2360, 2340, 1616, 1593, 1492, 1452, 1380, 1354, 1258, 1217, 1167, 1156, 1067, 1028, 1011, 958, 891, 845, 798, 751, 699, 633.

Methyl 4-(1-fluoroethyl)benzoate (13)

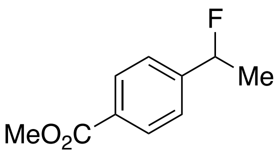

Synthesized according to general procedure E from methyl 4-(1-hydroxyethyl)benzoate. Purified by silica gel column chromatography (pentane/EtOAc, 100/0 to 98/2) to give the product as a clear oil (82 mg, 0.45 mmol, 90%). **^{1}H NMR** (400 MHz, $CDCl_3$) δ 8.04 (d, J = 7.8 Hz, 2H), 7.41 (d, J = 8.8 Hz, 2H), 5.67 (dq, J = 47.6, 6.5 Hz, 1H), 3.92 (s, 3H), 1.64 (dd, J = 23.9, 6.5 Hz, 3H); **^{13}C NMR** (101 MHz, $CDCl_3$) δ (C=O not visible) 166.9, 146.6 (d, J = 19.5 Hz), 130.0, 125.0 (d, J = 7.2 Hz), 90.5 (d, J = 169.7 Hz), 52.29, 23.12 (d, J = 24.6 Hz); **^{19}F NMR** (471 MHz, $CDCl_3$) δ -171.07 (dq, J = 47.7, 24.1 Hz); **HRMS** (EI) calculated for $C_{10}H_{11}FO_2$ $[M]^+$: 182.07376, found 182.07304. All spectroscopic data were in accordance with the literature.[40]

1-Bromo-4-(1-fluoroethyl)benzene (14)

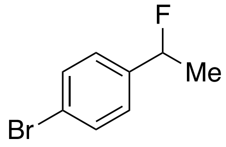

Synthesized according to general procedure E from 1-(4-bromophenyl)ethan-1-ol. Purified by silica gel column chromatography (pentane, 100%) to give the product as a clear oil (306 mg, 1.51 mmol, 75%). **^{1}H NMR** (500 MHz, $CDCl_3$) δ 7.51 (d, J = 8.5 Hz, 2H), 7.23 (d, J = 8.4 Hz, 2H), 5.59 (dq, J = 47.5, 6.4 Hz, 1H), 1.63 (dd, J = 24.0, 6.4 Hz, 3H); **^{13}C NMR** (126 MHz, $CDCl_3$) δ 140.6 (d, J = 20.0 Hz), 131.7, 127.0 (d, J = 6.8 Hz), 122.2 (d, J = 2.3 Hz), 90.4 (d, J = 168.5 Hz), 23.0 (d, J = 25.0 Hz); **^{19}F NMR** (471 MHz, $CDCl_3$) δ -167.80 – -186.16 (m); **HRMS** (EI) calculated for C_8H_8BrF $[M]^+$: 201.97879, found 211.97825. All spectroscopic data were in accordance with the literature.[44]

4-(1-Fluoroethyl)benzonitrile (15)

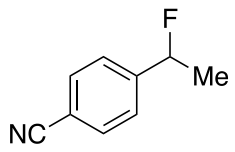

Synthesized according to general procedure E from 4-(1-hydroxyethyl)benzonitrile, which was synthesized according to literature procedure from 4-formylbenzonitrile.[12] Purified by silica gel column chromatography (pentane/EtOAc, 100:0 to 96:4) to give the product as a clear oil (51 mg, 0.34 mmol, 69%) **^{1}H NMR** (500 MHz, $CDCl_3$) δ 7.67 (d, J = 8.1 Hz, 2H), 7.44 (d, J = 8.7 Hz, 2H); 5.67 (dq, J = 47.5, 6.6 Hz, 1H), 1.64 (dd, J = 24.0, 6.4 Hz, 3H); **^{13}C NMR** (126 MHz, $CDCl_3$) δ 146.8 (d, J = 20.0 Hz), 132.5, 125.7 (d, J = 7.7 Hz), 118.7, 112.1 (d, J = 1.8 Hz), 90.0 (d, J = 170.8 Hz), 23.1 (d, J = 24.5 Hz); **^{19}F NMR** (471 MHz, $CDCl_3$) δ -172.58 (tdd, J = 48.0, 25.3, 8.5 Hz); **HRMS** (EI) calculated for C_9H_8FN $[M]^+$: 149.06353, found 149.06220. All spectroscopic data were in accordance with the literature.[45]

2-(4-(1-Fluoroethyl)phenyl)-4,4,5,5-tetramethyl-1,3,2-dioxaborolane (16)

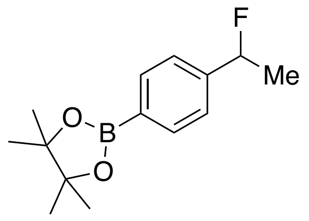

Synthesized according to an adapted literature procedure:[14]

To a flame-dried Schlenk tube equipped with a magnetic stirrer were added 1-bromo-4-(1-fluoroethyl)benzene **14** (51 mg, 0.25 mmol, 1.0 equiv), B_2Pin_2 (64 mg, 0.25 mmol, 1.0 equiv), KOAc (74 mg, 0.75 mmol, 3.0 equiv) and Pd(dppf)Cl_2 (9 mg, 12.5 µmol, 5.0 mol%). The tube vas evacuated and backfilled with N_2 three times prior to the addition of anhydrous and degassed dioxane (1.25 mL, 0.2 M). The reaction mixture was then stirred at 50 °C under N_2 for 16 h, then filtered through a short celite pad and washed with EtOAc. The filtrate was then evaporated *in vacuo* and the crude product was purified by silica gel column chromatography (CH_2Cl_2, 100%) to give the product as a white solid in quantitative yield. **^{1}H NMR** (400 MHz, $CDCl_3$) δ 7.83 (d, J = 7.6 Hz, 2H); 7.35 (d, J = 7.8 Hz, 2H), 5.64 (dq, J = 47.7, 6.5 Hz, 1H), 1.63 (dd, J = 23.9, 6.5 Hz, 3H), 1.35 (s, 12H); **^{13}C NMR** (101 MHz, $CDCl_3$) δ 144.7 (d, J = 19.5 Hz), 135.1, 124.5 (d, J = 7.2 Hz), 91.0 (d, J = 168.1 Hz), 84.0, 25.0, 23.2 (d, J = 25.0 Hz);

^{19}**F NMR** (377 MHz, $CDCl_3$) δ -169.59 (dq, J = 47.7, 23.8 Hz); ^{11}**B NMR** (161 MHz, $CDCl_3$) δ 31.27 (brs); **HRMS** (EI) calculated for $C_{14}H_{20}BFO_2$ $[M]^+$: 249.15712, found 249.15767; **IR**: 3649, 3629, 2979, 2930, 2360, 2341,1733, 1699, 1653, 1617, 1559, 1519, 1458, 1401, 1361, 1322, 1304, 1275, 1216, 1169, 1142, 1089, 1068, 1020, 1012, 963, 890, 859, 838, 762, 739, 674, 657; **m.p.**: 52–53 °C.

(3-(1-Fluoroethyl)phenyl)(phenyl)methanone (17)

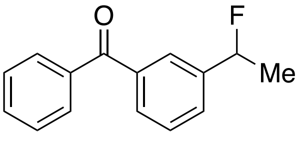

Synthesized according to literature procedure from Ketoprofen.[46] All spectroscopic data were in accordance with the literature.[46]

3-(4-(1-Fluoroethyl)benzyl)cyclopentan-1-one (18)

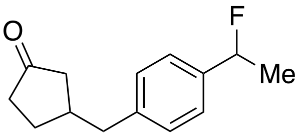

Synthesized according to general procedure F from 3-(4-(1-((2,2,6,6-tetramethylpiperidin-1-yl)oxy)ethyl)benzyl)cyclopentan-1-one **S18**. Purified by silica gel column chromatography (pentane/EtOAc, 100:0 to 90:10) to give the product as a pale-yellow oil (31 mg, 0.14 mmol, 70%) (diastereomers not resolved). ^{1}**H NMR** (500 MHz, $CDCl_3$) δ 7.27 (d, J = 7.4 Hz, 2H), 7.18 (d, J = 8.2 Hz, 2H), 5.60 (dq, J = 47.7, 6.4 Hz, 1H), 3.15 (dd, J = 14.0, 4.2 Hz, 1H), 2.55 (dd, J = 14.0, 9.4 Hz, 1H), 2.39–2.30 (m, 2H), 2.15–2.04 (m, 2H), 2.0–1.92 (m, 1H), 1.79–1.69 (m, 1H), 1.63 (dd, J = 23.8, 6.4 Hz, 3H), 1.60–1.51 (m, 1H); ^{13}**C NMR** (126 MHz, $CDCl_3$) δ (C=O not visible) 140.3 (d, J = 2.2 Hz), 139.4 (d, J = 19.5 Hz), 129.2, 125.6 (d, J = 6.4 Hz), 91.0 (d, J = 166.7 Hz), 51.1, 38.3, 35.4, 29.3, 22.9 (d, J = 25.4 Hz), 20.7; ^{19}**F NMR** (377 MHz, $CDCl_3$) δ -165.41 (dp, J = 47.7, 24.3 Hz); **HRMS** (EI) calculated for $C_{14}H_{17}FO$ $[M]^+$: 220.12579, found 220.12634; **IR**: 3460, 2979, 2935, 2876, 2367, 1914, 1739, 1617, 1516, 1453, 1421, 1406, 1376, 1350, 1294, 1273, 1257, 1216, 1155, 1066, 1022, 1005, 924, 883, 860, 824, 734, 641.

N-(4-(1-fluoroethyl)benzyl)cyclopropanesulfonamide (19)

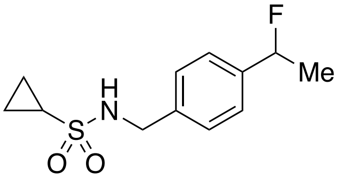

Synthesized according to general procedure F from *N*-(4-(1-((2,2,6,6-tetramethylpiperidin-1-yl)oxy)ethyl)benzyl)cyclopropanesulfonamide **S19**. Purified by silica gel column chromatography (pentane/EtOAc, 100:0 to 80:20) to give the product as a white solid (59 mg, 0.23 mmol, 92%). **^{1}H NMR** (400 MHz, $CDCl_3$) δ 7.41–7.32 (m, 4H), 5.63 (dq, J = 47.6, 6.5 Hz, 1H), 4.54 (brt, J = 6.3 Hz, 1H), 4.35 (d, J = 6.2 Hz, 2H), 2.36 (tt, J = 8.1, 4.8 Hz, 1H), 1.63 (dd, J = 23.9, 6.5 Hz, 3H) 1.19–1.14 (m, 2H), 0.98–0.92 (m, 2H); **^{13}C NMR** (101 MHz, $CDCl_3$) δ 141.3 (d, J = 18.9 Hz), 137.4, 128.0, 125.8 (d, J = 6.5 Hz), 90.7 (d, J = 167.5 Hz), 47.0, 30.6, 23.0 (d, J = 25.4 Hz), 5.6; **^{19}F NMR** (376 MHz, $CDCl_3$) δ -167.50 (dt, J = 47.7, 24.1 Hz); **HRMS** (ESI-TOF) calculated for $C_{12}H_{17}FNO_2S$ $[M]^+$: 258.0959, found 258.0960; **IR**: 3238, 2983, 2917, 2849, 2361, 2341, 2256, 1652, 1619, 1518, 1454, 1438, 1423, 1377, 1324, 1300, 1216, 1191, 1137, 1070, 1063, 1042, 1007, 950, 911, 894, 870, 838, 821, 781, 737, 698, 650; **m.p.**: 70–72 °C.

1-(1-Fluoroethyl)-4-((4-iodophenoxy)methyl)benzene (20)

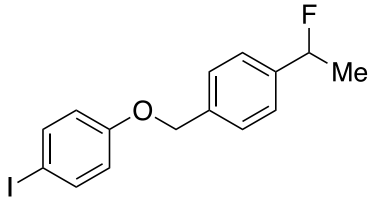

Synthesized according to general procedure E from 1-(4-((4-iodophenoxy)methyl)phenyl)ethan-1-ol. Purified by silica gel column chromatography (pentane/CH_2Cl_2) to give the product as a clear oil. **^{1}H NMR** (400 MHz, $CDCl_3$) δ 7.59–7.53 (m, 2H), 7.42 (d, J = 8.2 Hz, 2H), 7.37 (d, J = 8.5 Hz, 2H), 6.79–6.71 (m, 2H), 5.64 (dq, J = 47.61, 6.4 Hz, 1H), 5.04 (s, 2H), 1.65 (dd, J = 23.88, 6.4 Hz, 3H); **^{13}C NMR** (101 MHz, $CDCl_3$) δ 158.7, 141.6 (d, J = 19.6 Hz), 138.4, 136.7 (d, J = 1.8 Hz), 127.7, 125.7 (d, J = 6.5 Hz), 117.4,

90.8 (d, J = 167.8 Hz), 83.3, 69.9, 23.1 (d, J = 25.4 Hz); ^{19}**F NMR** (376 MHz, $CDCl_3$) δ -167.42 (dq, J = 47.2, 23.6 Hz); **HRMS**: not found; **IR**: 2959, 2925, 2853, 1584, 1467, 1380, 1280, 1235, 1177, 1060, 1009, 861, 809, 741, 633; **m.p.**: 45–47 °C.

1-(1-Fluoroethyl)-4-((4-iodophenoxy)methyl)benzene (21)

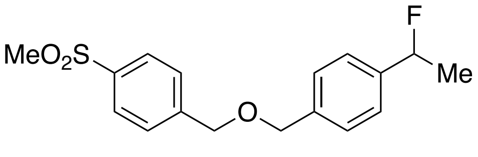

Synthesized according to general procedure F from 2,2,6,6-tetramethyl-1-(1-(4-(((4-(methylsulfonyl)benzyl)oxy)methyl)phenyl)ethoxy)piperidine **S21**. Purified by silica gel column chromatography (pentane/EtOAc, 100:0 to 70:30) to give the product as a clear oil (42 mg, 0.13 mmol, 87%). ^{1}**H NMR** (400 MHz, $CDCl_3$) δ 7.96–7.89 (m, 2H), 7.60–7.53 (m, 2H), 7.41–7.33 (m, 4H), 5.63 (dq, J = 47.7, 6.4 Hz, 1H), 4.64 (s, 2H), 4.61 (s, 2H), 3.04 (s, 3H), 1.64 (dd, J = 23.9, 6.4 Hz, 3H); ^{13}**C NMR** (151 MHz, $CDCl_3$) δ 144.9, 141.4 (d, J = 19.6 Hz), 139.8, 137.8 (d, J = 2.2 Hz), 128.2, 128.0, 127.7, 125.6 (d, J = 6.9 Hz), 90.9 (d, J = 167.5 Hz), 72.6, 71.2, 44.7, 23.1 (d, J = 25.1 Hz); ^{19}**F NMR** (376 MHz, $CDCl_3$) δ -167.01 (dq, J = 47.9, 23.6 Hz); **HRMS**: not found; **IR**: 2983, 2957, 2928, 2851, 2360, 2341,1601, 1519, 1458, 1412, 1377, 1359, 1316, 1304, 1259, 1217, 1181, 1151, 1090, 1066, 1019, 1004, 958, 886, 853, 821, 764, 723, 668, 654.

2-(1-Fluoroethyl)-6-methoxynaphthalene (22)

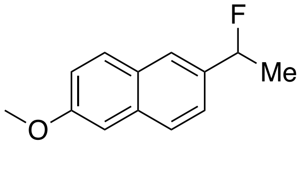

Synthesized according to literature procedure from Naproxen.[27] All spectroscopic data were in accordance with the literature.[27] *Note*: decomposition was observed during purification of the substrate.

(Fluoromethylene)dibenzene (23)

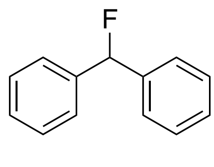

Synthesized according to general procedure E from diphenylmethanol. Purified by silica gel column chromatography (pentane, 100%) to give the product as a clear oil (31 mg, 0.17 mmol, 33%). **^{1}H NMR** (500 MHz, $CDCl_3$) δ 7.42–7.32 (m, 10H), 6.49 (d, J = 47.3 Hz, 1H); **^{13}C NMR** (126 MHz, $CDCl_3$) δ 140.0 (d, J = 21.3 Hz), 128.6, 128.6 (d, J = 2.3 Hz), 126.7 (d, J = 6.4 Hz), 94.6 (d, J = 172.6 Hz); **^{19}F NMR** (471 MHz, $CDCl_3$) δ -166.70 (d, J = 46.8 Hz); **HRMS** (EI) calculated for $C_{13}H_{11}F$ $[M]^+$: 186.08393, found 186.08342. All spectroscopic data were in accordance with the literature.[47]

1-Fluoro-1,2,3,4-tetrahydronaphthalene (24)

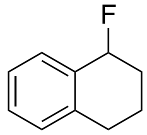

Synthesized according to general procedure E from 1,2,3,4-tetrahydronaphthalen-1-ol. Full degradation of the product was observed upon attempted purification by silica gel chromatography. Hence, the crude product was dissolved in pentane and filtered through a celite pipette plug, which was washed with further pentane. The filtrate was evaporated *in vacuo* and was not subjected to further purification. **^{1}H NMR** (500 MHz, $CDCl_3$) (benzylic proton) δ 5.53 (dt, J = 51.6, 4.0 Hz, 1H); **^{19}F NMR** (471 MHz, $CDCl_3$) δ -155.85 (m); **HRMS** (EI) calculated for $C_{10}H_{11}F$ $[M]^+$: 150.08393, found 150.08167. Spectroscopic data were in accordance with the literature.[48]

Methyl (1*R*,4a*S*,10a*R*)-9-fluoro-7-isopropyl-1,4a-dimethyl-1,2,3,4,4a,9,10,10a-octahydrophenanthrene-1-carboxylate (25)

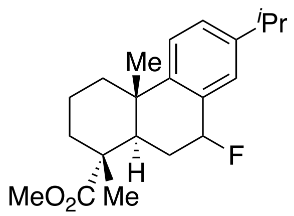

Synthesized according to general procedure F from methyl (1*R*,4a*S*,10a*R*)-7-isopropyl-1,4a-dimethyl-9-((2,2,6,6-tetramethylpiperidin-1-yl)oxy)-1,2,3,4,4a,9,10,10a-octahydrophenanthrene-1-carboxylate **S25** (as a 1:1 mixture of diastereomers). The instability of this product towards chromatographic purification has previously been reported.[16] **^{1}H NMR** (400 MHz, $CDCl_3$) δ (benzylic proton) δ 5.71 (dt, J = 52.8, 8.6 Hz, minor diastereomer), 5.50 (dd, J = 51.7, 2.3 Hz, major diastereomer); **^{19}F NMR** (471 MHz, $CDCl_3$) δ -148.82 (ddd, J = 51.7, 41.7, 21.3 Hz, major diastereomer), -162.04 (dd, J = 52.7, 19.6 Hz, minor diastereomer). **HRMS** (ESI-TOF) calculated for $C_{21}H_{29}FO_2$ $[M+Na^+]^+$: 355.2044, found 355.2044. All spectroscopic data were in accordance with the literature.[16]

A second reaction was performed according to general procedure F on 0.05 mmol scale. Upon completion, 4-Fluoroanisole (internal standard, 10 μL, 88 μmol, 1.76 equiv) was added and the reaction mixture was diluted with $CDCl_3$ and analyzed by quantitative ^{19}F NMR. Hence, the ^{19}F NMR yield was determined to be 72% (1.4:1 d.r).

5-Fluoro-5-phenylpentan-1-ol (26)

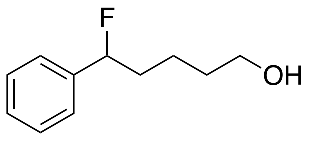

To a flame-dried round bottom flask equipped with a magnetic stirrer was added methyl 5-fluoro-5-phenylpentanoate (51 mg, 0.24 mmol, 1.0 equiv), which was synthesized according to literature procedure from methyl 5-phenylpentanoate.[49] The flask was evacuated and backfilled with N_2 three times prior to the addition of anhydrous THF (1.2 mL, 0.2 M) and $LiAlH_4$ (14 mg, 0.36 mmol, 1.5 equiv) as a solid. The reaction mixture was stirred at room temperature under N_2 for 30 min, then quenched with a few drops of H_2O and filtered over a short celite with the aid of some Et_2O. The filtrate was evaporated *in vacuo*, then purified by

silica gel column chromatography (pentane/EtOAc, 100:0 to 70:30) to give the product as a clear oil (42 mg, 0.23 mmol, 96%). **^{1}H NMR** (600 MHz, $CDCl_3$) δ 7.40–7.35 (m, 2H), 7.34–7.30 (m, 3H), 5.44 (ddd, J = 47.9, 8.1, 4.8 Hz, 1H), 3.65 (t, J = 6.4 Hz, 2H), 2.09–1.95 (m, 1H), 1.93–1.79 (m, 1H), 1.67–1.53 (m, 3H), 1.51–1.42 (m, 2H); **^{13}C NMR** (151 MHz, $CDCl_3$) δ 140.5 (d, J = 19.6 Hz), 128.6, 128.4 (d, J = 2.7 Hz), 125.6 (d, J = 6.5 Hz), 94.7 (d, J = 170.6 Hz), 62.8, 37.1 (d, J = 23.4 Hz), 32.5, 21.6 (d, J = 4.4 Hz); **^{19}F NMR** (377 MHz, $CDCl_3$) δ -174.64 (ddd, J = 46.2, 28.6, 17.1 Hz); **HRMS**: not found; **IR**: 3381, 3068, 3034, 2945, 2865, 2361, 2341, 1502, 1486, 1455, 1433, 1377, 1313, 1261, 1221, 1171, 1073, 1051, 1025, 990, 964, 915, 886, 860, 810, 762, 701, 670, 646, 631.

(5-Bromo-1-fluoropentyl)benzene (27)

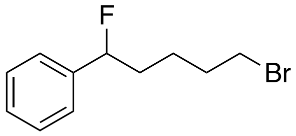

Synthesized according to general procedure F from 1-((5-bromo-1-phenylpentyl)oxy)-2,2,6,6-tetramethylpiperidine **S27**. Purified by silica gel column chromatography (pentane/Et_2O, 100:0 to 98:2) and preparative thin layer chromatography (pentane/Et_2O, 98:2) to give the product as a clear oil (17 mg, 69 μmol, 69%). **^{1}H NMR** (500 MHz, $CDCl_3$) δ 7.41–7.36 (m, 2H), 7.35–7.30 (m, 3H), 5.44 (ddd, J = 47.9, 8.2, 4.7 Hz, 1H), 3.41 (t, J = 6.7 Hz, 2H), 2.07–1.77 (m, 4H), 1.71–1.48 (m, 2H); **^{13}C NMR** (126 MHz, $CDCl_3$) δ 140.3 (d, J = 19.5 Hz), 128.6, 128.5 (d, J = 2.3 Hz), 125.6 (d, J = 6.8 Hz), 94.5 (d, J = 170.8 Hz), 36.5 (d, J = 24.1 Hz), 33.5, 32.6, 24.0 (d, J = 4.5 Hz); **^{19}F NMR** (471 MHz, $CDCl_3$) δ -174.92 (ddd, J = 46.8, 28.6, 16.9 Hz); **HRMS** (EI) calculated for $C_{11}H_{14}BrF$ $[M]^+$: 244.02574, found 244.02629; **IR**: 2948, 2868, 2360, 2342, 1723, 1595, 1497, 1457, 1436, 1370, 1296, 1259, 1212, 1124, 1092, 1062, 1044, 1028, 985, 914, 867, 805, 760, 701, 669, 654, 622.

5-Fluoro-5-phenylpentyl 4-methylbenzenesulfonate (28)

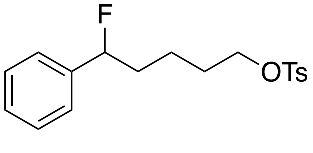

Synthesized according to general procedure F from 5-phenyl-5-((2,2,6,6-tetramethylpiperidin-1-yl)oxy)pentyl 4-methylbenzenesulfonate **S28**. Purified by silica gel column chromatography (pentane/EtOAc, 100:0 to 96:4) to give the product as a white solid (38 mg, 0.11 mmol, 57%). **^{1}H NMR** (400 MHz, $CDCl_3$) δ 7.81–7.55 (m, 2H), 7.40–7.25 (m, 7H), 5.37 (ddd, J = 47.8, 8.2, 4.6 Hz, 1H), 4.02 (t, J = 6.4 Hz, 2H), 2.45 (s, 3H), 1.99–1.65 (m, 4H), 1.59–1.35 (m, 2H); **^{13}C NMR** (126 MHz, $CDCl_3$) δ 144.9, 140.2 (d, J = 19.5 Hz), 133.2, 130.0, 128.6, 128.5 (d, J = 2.3 Hz), 128.0, 125.6 (d, J = 6.8 Hz), 94.3 (d, J = 171.2 Hz), 70.3, 36.6 (d, J = 23.6 Hz), 28.7, 21.8, 21.3 (d, J = 4.5 Hz); **^{19}F NMR** (377 MHz, $CDCl_3$) δ -175.30 (ddd, J = 46.7, 28.6, 17.1 Hz); **HRMS**: not found, **IR**: 2957, 2927, 2873, 2852, 2362, 1931, 1665, 1597, 1496, 1468, 1452, 1431, 1356, 1306, 1260, 1190, 1171, 1098, 1041, 1007, 956, 923, 856, 820, 778, 751, 735, 696, 667, 628; **m.p.**: 53–54 °C.

2,3,5,6-Tetrafluorophenyl 5-fluoro-5-phenylpentanoate (29)

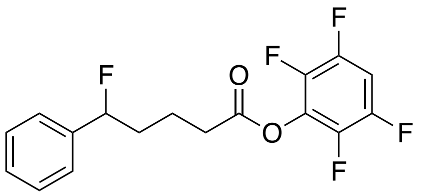

Synthesized according to general procedure F from 2,3,5,6-tetrafluorophenyl 5-phenyl-5-((2,2,6,6-tetramethylpiperidin-1-yl)oxy)pentanoate **S29**. Purified by silica gel column chromatography chromatography (pentane/EtOAc, 100:0 to 99:1) and preparative thin layer chromatography (pentane/EtOAc, 95:5) to give the product as a clear oil (35 mg, 0.10 mmol, 68%). **^{1}H NMR** (400 MHz, $CDCl_3$) δ 7.43–7.30 (m, 5H), 7.00 (tt, J = 9.9, 7.1 Hz, 1H), 5.50 (ddd, J = 47.9, 8.2, 4.0 Hz, 0.5H), 2.75 (td, J = 7.2, 2.4 Hz, 2H), 2.18–1.82 (m, 4H); **^{13}C NMR** (126 MHz, $CDCl_3$) δ 169.2, 146.2 (dtd, J = 248.7, 12.1, 4.1 Hz), 140.7 (dddd, J = 250.3, 15.5, 5.1, 2.3 Hz), 140.0 (d, J = 19.8 Hz), 129.8 (tt, J = 13.7, 3.7 Hz), 128.7, 128.6 (d, J = 2.2 Hz), 125.5 (d, J = 6.6 Hz), 103.3 (t, J = 22.8 Hz), 94.1 (d, J = 171.7 Hz), 36.3 (d, J = 23.7 Hz), 33.08, 20.66 (d, J = 3.9 Hz); **^{19}F NMR** (377 MHz, $CDCl_3$) δ -138.86 – -139.04 (m, 2F), -152-94 – -153.10 (m, 2F), -175.89 – -176.26 (m, 1F); **HRMS**: not found, **IR**: 3088, 2957, 2929, 2851, 2360, 2341, 1792, 1646, 1527, 1489, 1456, 1383, 1274, 1218, 1180, 1135, 1112, 1090, 1070, 957, 916, 843, 759, 718, 701, 647, 628.

2-(1-Fluoroethyl)benzofuran (30)

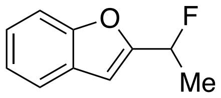

Synthesized according to general procedure E from 1-(benzofuran-2-yl)ethan-1-ol. Purified by silica gel column chromatography (pentane, 100%) to give the product as a clear oil (36 mg, 0.22 mmol, 22%). **^{1}H NMR** (400 MHz, $CDCl_3$) δ 7.58 (d, J = 7.0 Hz, 1H), 7.50 (d, J = 8.2 Hz, 1H), 7.32 (t, J = 7.7 Hz, 1H), 7.25–7.20 (m, 1H), 6.77 (d, J = 3.8 Hz, 1H), 5.73 (dq, J = 48.4, 6.5 Hz, 1H), 1.80 (dd, J = 23.0, 6.6 Hz, 3H); **^{13}C NMR** (151 MHz, $CDCl_3$) δ 155.5 (d, J = 20.6 Hz), 155.2 (d, J = 1.5 Hz), 127.8 (d, J = 2.2 Hz), 125.1 (d, J = 1.7 Hz), 123.1, 121.6 (d, J = 1.6 Hz), 111.7, 104.8 (d, J = 6.1 Hz), 84.1 (d, J = 165.6 Hz), 19.2 (d, J = 24.8 Hz); **^{19}F NMR** (376 MHz, $CDCl_3$) δ -165.8 (dqd, J = 46.2, 23.2, 3.9 Hz); **HRMS** (EI) calculated for $C_{10}H_9FO$ $[M]^+$: 164.06319, found 164.06374; **IR**: 2955, 2924, 2856, 2361, 2342, 1750, 1497, 1455,1368, 1227, 1157, 1103, 1028, 978, 913, 869, 786, 741, 699, 669. All spectroscopic data were in accordance with the literature.[47]

6-Chloro-2-(1-fluoroethyl)-9-methyl-9*H*-carbazole (31)

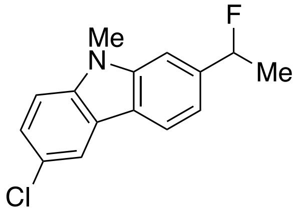

Synthesized according to general procedure F from 6-chloro-9-methyl-2-(1-((2,2,6,6-tetramethylpiperidin-1-yl)oxy)ethyl)-9*H*-carbazole **S31**. Attempted chromatographic purification led to decomposition of the product. **^{1}H NMR** (400 MHz, $CDCl_3$) δ 8.07–8.00 (m, 2H), 7.46–7.41 (m, 2H), 7.33 (d, J = 8.6 Hz, 1H), 7.21 (dt, J = 8.1, 1.4 Hz, 1H), 5.85 (dq, J = 47.7, 6.4 Hz, 1H), 3.86 (s, 3H), 1.77 (dd, J = 23.9, 6.4 Hz, 3H); **^{13}C NMR** (101 MHz, $CDCl_3$) δ 141.1, 139.7 (d, J = 19.3 Hz), 139.3, 125.5, 124.2, 123.2, 121.4 (d, J = 1.5 Hz), 120.2, 119.7, 116.2 (d, J = 6.2 Hz), 109.1, 105.0 (d, J = 8.0 Hz), 91.3 (d, J = 168.2 Hz), 28.9, 23.1 (d, J = 25.4 Hz); **^{19}F NMR** (377 MHz, $CDCl_3$) δ -165.04 (dq, J = 47.7, 24.1 Hz); **HRMS** (ESI-TOF) calculated for $C_{15}H_{13}ClFN$ $[M]^+$: 262.0793, found 262.0930.

A second reaction was performed according to general procedure F on 0.05 mmol scale. Upon completion, 4-Fluoroanisole (internal standard, 10 μL, 88 μmol, 1.76 equiv) was added and the reaction mixture was diluted with $CDCl_3$ and analyzed by quantitative ^{19}F NMR. Hence, the ^{19}F NMR yield was determined to be 64%.

5-(1-fluoro-2-(4-(4-fluorophenyl)-1*H*-1,2,3-triazol-1-yl)ethyl)-4-methylthiazole (32)

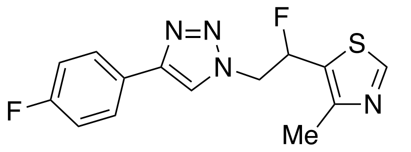

Synthesized according to general procedure F from 5-(2-(4-(4-fluorophenyl)-1H-1,2,3-triazol-1-yl)-1-((2,2,6,6-tetramethylpiperidin-1-yl)oxy)ethyl)-4-methylthiazole **S32**. Purified by silica gel column chromatography (pentane/EtOAc, 100:0 to 20:80) to give the product as a white solid (50 mg, 0.16 mmol, 82%). **^{1}H NMR** (400 MHz, $CDCl_3$) δ 8.80 (s, 1H), 7.87–7.78 (m, 3H), 7.17–7.09 (m, 2H), 6.17 (ddd, J = 47.1, 7.7, 3.9 Hz, 1H), 4.95–4.80 (m, 2H), 2.50 (d, J = 2.5 Hz, 3H); **^{13}C NMR** (101 MHz, $CDCl_3$) δ 163.0 (d, J = 247.8 Hz), 153.2 (d, J = 5.1 Hz), 153.1 (d, J = 1.8 Hz), 147.7, 127.7 (d, J = 8.0 Hz), 126.6 (d, J = 3.3 Hz), 125.1 (d, J = 20.7 Hz), 120.7 (d, J = 2.5 Hz), 116.1 (d, J = 21.8 Hz), 86.2 (d, J = 176.9 Hz), 55.0 (d, J = 26.9 Hz), 15.6; **^{19}F NMR** (376 MHz, $CDCl_3$) δ -113.11 (tt, J = 8.3, 5.2 Hz, 1F), -163.20 – -163.50 (m, 1F); **HRMS** (ESI-TOF) calculated for $C_{14}H_{13}F_2N_4S$ $[M]^+$: 307.0824, found 307.0818; **IR**: 2360, 2341, 1728, 1611, 1563, 1544, 1501, 1460, 1413, 1382, 1354, 1329, 1266, 1236, 1188, 1158, 1046, 1019, 1006, 975, 945, 911, 854, 833, 736, 720, 686, 669; **m.p.**: 148–149 °C.

(4-(Fluoromethyl)phenyl)(methyl)sulfane (33)

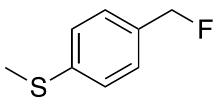

To a flame-dried Schlenk tube equipped with a magnetic stirrer was added (4-(bromomethyl)phenyl)(methyl)sulfane (87 mg, 0.40 mmol, 1.0 equiv). TBAF (1.0 M in THF, 1.20 mL, 1.20 mmol, 3.0 equiv) was then added dropwise. The reaction mixture was stirred at room temperature under N_2 for 16 h, then diluted with H_2O and extracted with CH_2Cl_2 three

times. The combined organic layers were dried over Na_2SO_4 and evaporated *in vacuo*. The crude product was then purified by silica gel column chromatography (pentane/EtOAc, 100:0 to 95:5) to give the product as a clear oil (27 mg, 0.17 mmol, 43%). **^{1}H NMR** (400 MHz, $CDCl_3$) δ 7.32–7.25 (m, 4H), 5.33 (d, J = 48.1 Hz, 2H), 2.50 (s, 3H); **^{13}C NMR** (126 MHz, $CDCl_3$) δ 139.7 (d, J = 3.6 Hz), 132.9 (d, J = 16.8 Hz), 128.5 (d, J = 5.9 Hz), 126.6 (d, J = 1.8 Hz), 84.4 (d, J = 165.3 Hz), 15.8; **^{19}F NMR** (377 MHz, $CDCl_3$) δ -204.37 (t, J = 48.1 Hz); **HRMS** (EI) calculated for C_8H_9FS $[M]^+$: 156.04035, found 156.0409. All spectroscopic data were in accordance with the literature.[27]

1-Azido-4-(fluoromethyl)benzene (34)

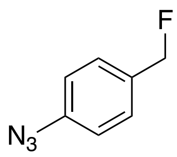

To a flame-dried Schlenk tube equipped with a magnetic stirrer was added azido-4-(bromomethyl)benzene (115 mg, 0.54 mmol, 1.0 equiv), which was synthesized according to literature procedure from (4-aminophenyl)methanol.[20] TBAF (1.0 M in THF, 2.70 mL, 2.70 mmol, 5.0 equiv) was then added dropwise. The reaction mixture was stirred at 40 °C under N_2 for 5 h, then diluted with CH_2Cl_2 and washed with a saturated aqueous NaCl solution. The solvent was removed *in vacuo* and the crude product was purified by silica gel column chromatography (pentane/EtOAc, 100:0 to 99:1) to give the product as a pale-yellow oil (26 mg, 0.17 mmol, 32%). **^{1}H NMR** (500 MHz, $CDCl_3$) δ 7.38 (dd, J = 8.5, 2.1 Hz, 2H), 7.05 (d, J = 7.5 Hz, 2H), 5.34 (d, J = 47.9 Hz, 2H); **^{13}C NMR** (126 MHz, $CDCl_3$) δ 140.8 (d, J = 3.2 Hz), 133.0 (d, J = 17.3 Hz), 123.0 (d, J = 5.9 Hz), 119.3 (d, J = 1.8 Hz), 84.1 (d, J = 166.2 Hz); **^{19}F NMR** (471 MHz, $CDCl_3$) δ -204.67 (t, J = 48.1 Hz), **HRMS** (ESI-TOF) calculated for $C_7H_6FN_3$ $[M+H^+]^+$: 152.0619, found 152.0739. All spectroscopic data were in accordance with the literature.[50]

1-(Fluoromethyl)-4-methoxybenzene (35)

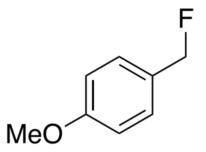

Synthesized according to general procedure E from (4-methoxyphenyl)methanol. Due to instability reasons, the crude product was filtered through a celite pipette plug, which was washed with further CH_2Cl_2. The filtrate was evaporated *in vacuo* and was not subjected to further purification. **^{1}H NMR** (400 MHz, $CDCl_3$) δ 5.31 (d, J = 48.8 Hz) (benzylic protons), 3.83 (s, 3H) (OMe protons); **^{19}F NMR** (376 MHz, $CDCl_3$) δ -199.17 (t, J = 48.9 Hz). All spectroscopic data were in accordance with the literature.[51]

(2-Fluoropropan-2-yl)benzene (36)

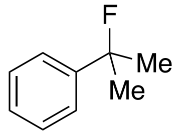

Synthesized according to general procedure F from 2,2,6,6-tetramethyl-1-((2-phenylpropan-2-yl)oxy)piperidine **S36**. Purified by preparative thin layer chromatography (pentane, 100%) to give the product as a pale-yellow oil (21 mg, 0.15 mmol, 76%). **^{1}H NMR** (600 MHz, $CDCl_3$) δ 7.52–7.47 (m, 1H), 7.41–7.33 (m, 4H), 1.69 (d, J = 22.0 Hz, 6H); **^{13}C NMR** (151 MHz, $CDCl_3$) δ 146.0 (d, J = 21.8 Hz), 128.4 (d, J = 1.0 Hz), 127.4 (d, J = 1.1 Hz), 123.9 (d, J = 8.7 Hz), 95.8 (d, J = 168.9 Hz) 29.4 (d, J = 26.2 Hz); **^{19}F NMR** (565 MHz, $CDCl_3$) δ -137.38 (hept, J = 21.7 Hz). **HRMS**: not found. All spectroscopic data were in accordance with the literature.[27]

(1-Fluorocyclohexyl)benzene (37)

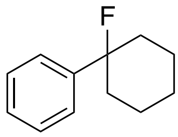

Synthesized according to general procedure F from 2,2,6,6-tetramethyl-1-((1-phenylcyclohexyl)oxy)piperidine **S37**. Purified by preparative thin layer chromatography (pentane, 100%) to give the product as a pale-yellow oil (29 mg, 0.16 mmol, 81%). **^{1}H NMR**

(600 MHz, $CDCl_3$) δ 7.41–7.34 (m, 4H), 7.30–7.25 (m, 1H), 2.07–1.98 (m, 2H), 1.86–1.65 (m, 7H), 1.38–1.25 (m, 1H); **^{13}C NMR** (151 MHz, $CDCl_3$) δ 146.0 (d, J = 21.8 Hz), 128.2 (d, J = 1.1 Hz), 127.2 (d, J = 1.5 Hz), 123.9 (d, J = 9.3 Hz), 96.0 (d, J = 173.8 Hz), 37.2 (d, J = 23.4 Hz), 25.0, 22.0 (d, J = 2.2 Hz); **^{19}F NMR** (565 MHz, $CDCl_3$) δ -158.70 (brs); **HRMS**: not found. All spectroscopic data were in accordance with the literature.[52]

2-((4-Fluoro-4-methylpentyl)oxy)-1,4-dimethylbenzene (38)

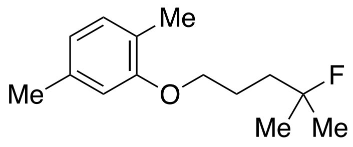

Synthesized according to literature procedure from 1,3-dioxoisoindolin-2-yl 5-(2,5-dimethylphenoxy)-2,2-dimethylpentanoate.[27] All spectroscopic data were in accordance with the literature.[27]

***tert*-Butyl ((1*r*,3*s*,5*R*,7*S*)-3-fluoroadamantan-1-yl)carbamate (39)**

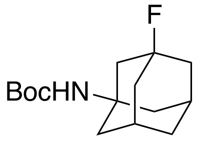

Synthesized according to literature procedure from *tert*-butyl *N*-(adamantan-1-yl)carbamate.[53] All spectroscopic data were in accordance with the literature.[53]

2-Fluoro-1,4-dioxane (40)

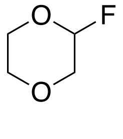

Synthesized from 1-((1,4-dioxan-2-yl)oxy)-2,2,6,6-tetramethylpiperidine **S40** according to an adapted version of general procedure F due to the volatility of the product. CD_2Cl_2 was used as solvent and upon completion, the reaction mixture was washed with a saturated aqueous $NaHCO_3$ solution. The organic layer was dried over Na_2SO_4 and filtered through a celite pipette

plug, which was washed with further CD_2Cl_2. The filtrate was then distilled *in vacuo* at room temperature and collected in a round bottom flask which was cooled to -78 °C. The product was not subjected to further purification and directly analyzed by NMR. **^{1}H NMR** (101 MHz, CD_2Cl_2) δ 5.37 (d, J = 51.7 Hz, 1H), 4.23–4.14 (m, 1H), 3.86–3.74 (m, 3H), 3.72–3.55 (m, 2H); **^{13}C NMR** (101 MHz, CD_2Cl_2) δ 103.9 (d, J = 220.9 Hz), 67.7 (d, J = 25.8 Hz), 66.1 (d, J = 2.2 Hz), 61.3 (d, J = 2.2 Hz); **^{19}F NMR** (376 MHz, CD_2Cl_2) δ -137.87 (ddd, J = 52.0, 36.1, 6.9 Hz); **HRMS**: not found. All spectroscopic data were in accordance with the literature.[54]

A second reaction was performed according to general procedure F on 0.05 mmol scale. Upon completion, 4-Fluoroanisole (internal standard, 10 µL, 88 µmol, 1.76 equiv) was added and the reaction mixture was diluted with $CDCl_3$ and analyzed by quantitative ^{19}F NMR. Hence, the ^{19}F NMR yield was determined to be 43%.

3-Fluoro-4-(4-(1-fluoroethoxy)phenoxy)benzonitrile (41)

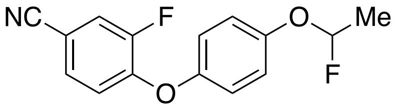

Synthesized according to literature procedure from Cyhalofop.[27] All spectroscopic data were in accordance with the literature.[27]

(Fluoromethyl)(phenyl)sulfane (42)

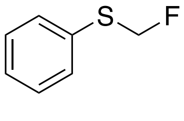

To a flame-dried Schlenk tube equipped with a magnetic stirrer was added Cs_2CO_3 (244 mg, 0.75 mmol, 1.5 equiv) and the tube was evacuated and backfilled with N_2 three times prior to the addition of anhydrous MeCN (5.0 mL, 0.1 M) and thiophenol (51 µL, 0.5 mmol, 1.0 equiv). CH_2FI (41 µL, 0.6 mmol, 1.2 equiv) was then added dropwise and the reaction mixture was stirred at room temperature under N_2 for 3 h, then diluted with Et_2O and filtered over a short silica pad. The filtrate was washed with a saturated aqueous $NaHCO_3$ solution, then a saturated aqueous NaCl solution. The organic layer was dried over Na_2SO_4 and evaporated in *in vacuo*

to give the product as clear oil (59 mg, 0.42 mmol, 83%). **^{1}H NMR** (400 MHz, $CDCl_3$) δ 7.52–7.47 (m, 2H), 7.37–7.28 (m, 3H), 5.73 (d, J = 52.9 Hz, 2H); **^{13}C NMR** (101 MHz, $CDCl_3$) δ 130.79, 130.76, 129.4, 127.9, 88.7 (d, J = 216.2 Hz); **^{19}F NMR** (376 MHz, $CDCl_3$) δ -181.67 (t, J = 52.7 Hz); **HRMS** (EI) calculated for C_7H_7FS $[M]^+$: 142.0247 found 142.02525. All spectroscopic data were in accordance with the literature.[55]

2-(1-Fluoro-2-phenylethyl)isoindoline-1,3-dione (43)

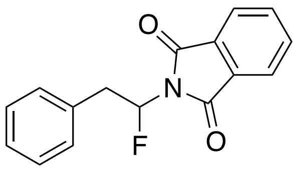

Synthesized according to literature procedure from *N*-phthaloyl-L-phenylalanine.[56] All spectroscopic data were in accordance with the literature.[56]

3-Fluorocyclohex-1-ene (44)

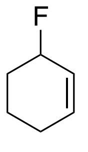

Synthesized according to general procedure E from cyclohex-2-en-1-ol. Due to the volatility of the product, purification by silica gel column chromatography was unsuccessful. Hence, the crude product was dissolved in pentane and filtered through a celite pipette plug, which was washed with further pentane. The filtrate was evaporated under N_2 flow, then distilled *in vacuo* at room temperature and collected in a round bottom flask which was cooled to -78 °C. The product was not subjected to further purification. **^{1}H NMR** (500 MHz, $CDCl_3$) (allylic proton) δ 5.0 (dm, J = 49.6, 1H); **^{19}F NMR** (471 MHz, $CDCl_3$) δ -165.01 – -165.33 (m); **HRMS** (EI) calculated for C_6H_9F $[M]^+$: 100.06828, found 100.06783. Spectroscopic data were in accordance with the literature.[57]

2-Fluoro-1,2-bis(4-methoxyphenyl)ethan-1-one (45)

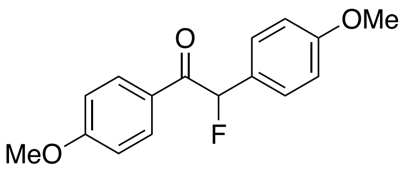

Synthesized according to general procedure F from 1,2-bis(4-methoxyphenyl)-2-((2,2,6,6-tetramethylpiperidin-1-yl)oxy)ethan-1-one **S45**. Purified by preparative thin layer chromatography (pentane/EtOAc, 80:20) to give the product as a white solid (11 mg, 40 µmol, 40%). **^{1}H NMR** (600 MHz, $CDCl_3$) δ 7.92 (d, J = 9.0 Hz, 2H), 7.40 (dd, J = 8.8, 1.9 Hz, 2H), 6.92–6.87 (m, 4H), 6.44 (d, J = 49.0 Hz, 1H), 3.84 (s, 3H), 3.79 (s, 3H); **^{13}C NMR** (151 MHz, $CDCl_3$) δ 192.8 (d, J = 21.5 Hz), 164.0, 160.7 (d, J = 2.8 Hz), 132.5, 131.6 (d, J = 2.8 Hz), 129.5 (d, J = 4.4 Hz), 127.2, 126.9 (d, J = 20.4 Hz), 114.6 (d, J = 1.2 Hz), 114.1, 93.5 (d, J = 184.3 Hz), 55.6, 55.5; **^{19}F NMR** (565 MHz, $CDCl_3$) δ -170.98 (d, J = 49.0 Hz). **HRMS** (ESI-TOF) calculated for $C_{16}H_{15}FO_3$ $[M+Na^+]^+$: 297.0897, found 297.0898. All spectroscopic data were in accordance with the literature.[47]

4-(4-(1-Fluoroethyl)phenethoxy)quinazoline (46)

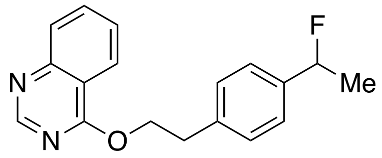

Synthesized according to general procedure F from 4-(4-(1-((2,2,6,6-tetramethylpiperidin-1-yl)oxy)ethyl)phenethoxy)quinazoline **S46**. Purified by silica gel column chromatography (pentane/EtOAc, 100:0 to 80:20) to give the product as a white solid (29 mg, 0.98 mmol, 98%). **^{1}H NMR** (400 MHz, $CDCl_3$) δ 8.80 (s, 1H), 8.14 (d, J = 8.1 Hz, 1H), 7.95 (d, J = 8.2 Hz, 1H), 7.84 (ddd, J = 8.5, 7.0, 1.5 Hz, 1H), 7.57 (ddd, J = 8.2, 7.0, 1.2 Hz, 1H), 7.38–7.29 (m, 4H), 5.62 (dq, J = 47.7, 6.4 Hz, 1H), 4.80 (t, J = 6.9 Hz, 2H), 3.23 (t, J = 6.9 Hz, 2H), 1.64 (dd, J = 23.8, 6.4 Hz, 3H); **^{13}C NMR** (126 MHz, $CDCl_3$) δ 166.9, 154.4, 150.7, 140.0 (d, J = 20.0 Hz), 138.2 (d, J = 2.7 Hz), 133.9, 129.3, 127.6, 127.4, 125.7 (d, J = 6.4 Hz), 123.7, 116.7, 91.0 (d, J = 167.1 Hz), 67.7, 35.1, 23.0 (d, J = 25.0 Hz); **^{19}F NMR** (376 MHz, $CDCl_3$) δ -166.08 (dq, J = 47.9, 24.6 Hz); **HRMS** (ESI-TOF) calculated for $C_{18}H_{17}FN_2O$ $[M+H^+]^+$: 297.1398, found 297.1395; **IR**: 2929, 2360, 2340, 1621, 1574, 1497, 1457, 1421, 1354, 1297, 1217, 1186, 1160, 1095, 1066, 1005, 972, 919, 904, 884, 824, 802, 773, 685, 637; **m.p.**: 43–44 °C.

Radiochemistry

General experimental details

For manual radiochemistry experiments:

[^{18}F]Fluoride was produced by Invicro (UK) or PETIC (UK) via the ^{18}O(p,n)^{18}F reaction and delivered as [^{18}F]fluoride in [^{18}O]water. Radiosynthesis and azeotropic drying were performed on a NanoTek® automated microfluidic device (Advion). HPLC analysis was performed with a Dionex Ultimate 3000 dual channel HPLC system equipped with shared autosampler, parallel UV-detectors and LabLogic NaI/PMT-radiodetectors with Flow-RAM analog output. Due to the separation of the modules the radio-signal is offset by 0.1-0.3 min from the UV signal.

For automated radiochemistry experiments:

[^{18}F]Fluoride was produced in an IBA Cyclon 18/9 cyclotron using the ^{18}O(p,n)^{18}F reaction. All experiments were performed on a TRASIS AllinOne radiosynthesizer (TRASIS). Radio-high-performance liquid (radio-HPLC) chromatography was performed on an Agilent 1200 equipped with a LabLogic gamma-RAM Model 4 detector.

HPLC eluent systems and columns:

Analytical HPLC runs were performed either with a Dionex Ultimate 3000 dual channel HPLC system equipped with shared autosampler, parallel UV-detectors and LabLogic NaI/PMT-radiodetectors with Flow-RAM analog output (Conditions A, approximate radio-UV detector offset = 0.1 min (pump 1) or 0.3 min (pump 2)), or on an Agilent 1200 equipped with a UV detector and LabLogic gamma-RAM Model 4 detector (Conditions B, C, D: approximate radio-UV detector offset = 0.1 min). Semi-preparative purification of radiolabeled products was achieved using the integrated HPLC system (including UV and radio detectors) of a Trasis AllInOne synthesizer.

Conditions A: analytical

Flow rate = 1.0 mL/min; temperature = 25 °C; wavelength = 220 nm (unless otherwise specified); column: Phenomenex Synergi™ 4 µm Hydro RP 80 Å 150 x 4.6 mm LC column; HPLC gradient: H_2O/MeCN, 0-1 min (25% MeCN) isocratic, 1-10 min (25% MeCN to 95% MeCN) linear increase, 10-14 min (95% MeCN) isocratic, 14-17 min (95% MeCN to 25% MeCN) linear decrease, 17-19.4 min (25% MeCN) isocratic.

Conditions B: analytical

Flow rate = 1.0 mL/min; temperature = 25 °C; wavelength = 220 nm (unless otherwise specified); column: Phenomenex Synergi™ 4 µm Hydro RP 80 Å 150 x 4.6 mm LC column; HPLC gradient: H_2O/MeCN, 0-1 min (25% MeCN) isocratic, 1-10 min (25% MeCN to 95% MeCN) linear increase, 10-16 min (95% MeCN) isocratic, 16-18 min (95% MeCN to 25% MeCN) linear decrease, 18-20 min (25% MeCN) isocratic.

Conditions C: analytical

Flow rate = 1.0 mL/min; temperature = 25 °C; wavelength = 220 nm (unless otherwise specified); column: Agilent C18 Eclipse Plus 80 Å 150 x 4.6 mm LC column; HPLC gradient: H_2O/MeCN, 0-1 min (25% MeCN) isocratic, 1-10 min (25% MeCN to 95% MeCN) linear increase, 10-16 min (95% MeCN) isocratic, 16-18 min (95% MeCN to 25% MeCN) linear decrease, 18-20 min (25% MeCN) isocratic.

Conditions D: analytical

Flow rate = 1.0 mL/min; temperature = 25 °C; wavelength = 220 nm (unless otherwise specified); column: Agilent C18 Eclipse Plus 80 Å 150 x 4.6 mm LC column; HPLC gradient: H_2O/MeCN, 0-1 min (5% MeCN) isocratic, 1-10 min (5% MeCN to 95% MeCN) linear increase, 10-14 min (95% MeCN) isocratic, 14-15 min (95% MeCN to 5% MeCN) linear decrease, 15-17 min (25% MeCN) isocratic.

Conditions E: semi-preparative purification

Flow rate = 4.0 mL/min; temperature = room temperature; wavelength = 254 nm; column: Phenomenex Gemini 5 µm 250 x 10 mm LC column; isocratic: water/MeCN.

Optimization of the reaction conditions

Procedure for the radiofluorination of TEMPO-derived substrates

Manual [^{18}F]KF elution and drying: [^{18}F]Fluoride was separated from ^{18}O-enriched-water using an anion exchange cartridge (Waters Sep-Pak AccellPlus QMA Carbonate Plus Light Cartridge), activated with H_2O (10 mL) prior to use and released with a solution of Kryptofix (7.5 mg), and K_2CO_3 (1.5 mg) in MeCN/H_2O (0.75 mL, 4:1, v/v), which was concentrated over a period of 20 min by azeotropic drying using dry MeCN (3 x 0.7 mL) under a flow of N_2 at 110 °C.

^{18}F-fluorination: To an oven-dried 4 mL glass vial equipped with a magnetic stirrer and capped with a septum was added [^{18}F]KF (10-50 MBq) in MeCN (approximately 50 µL) and the MeCN was subsequently removed by drying under a flow of N_2 at 110 °C. To the vial was then added a solution containing TEMPO-derived substrate (given mmol), photocatalyst (0.5 mg, 0.5 µmol), solvent (given volume), proton source (given mmol), and additive(s) if applicable. The mixture was degassed by nitrogen bubbling for 10 seconds, then stirred in a SynLED Parallel Photoreactor with an EvoluChem 18 W blue LED lamp (λ = 450 nm) at room temperature (with a fan) for 20 minutes. The reaction mixture was then diluted with EtOH/H_2O (300 µL, 9:1 v/v) and an aliquot was filtered and analyzed by radioHPLC. The radiochemical yield (RCY) was determined by integration of the ^{18}F-product relative to the total peak area for all radioactive species observed.

Table s11: Solvent screen

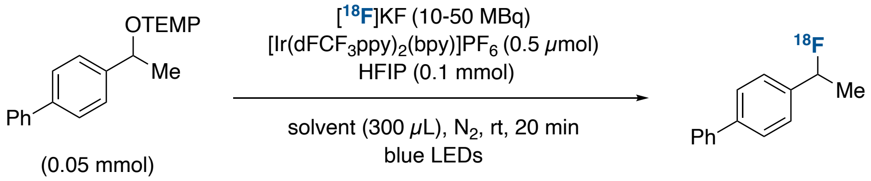

Entry	Solvent	$RCY_{n=1}$
**1**	MeCN	0%[a]
**2**	THF	51%
**3**	DMF	29%
**4**	CH_2Cl_2	71%

[a] starting material was insoluble

Table s12: Screening of proton sources

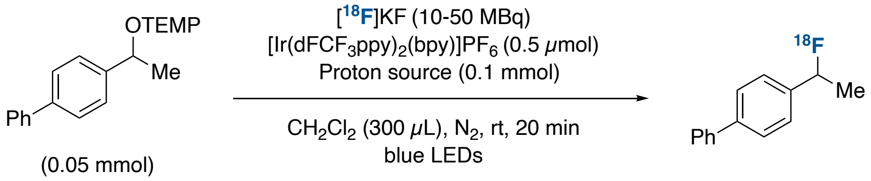

Entry	Proton source	$RCY_{n=1}$
**1**	None	11%
**2**	HFIP	71%
**3**	TFA	13%
**4**	TCAA	3%
**5**	CSA	0%
**6**	MeOH	36%

HFIP: hexafluoroisopropanol, TFA: trifluoroacetic acid, TCAA: trichloroacetic acid, CSA: camphor sulfonic acid, MeOH: methanol

Table s13: Screening of HFIP loading

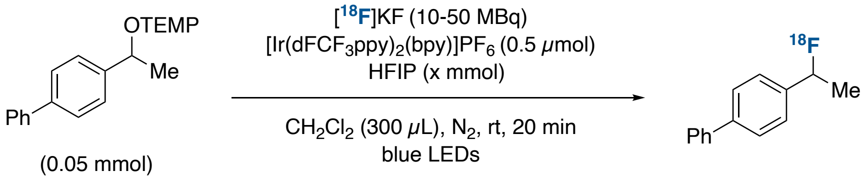

Entry	HFIP mmol	RCY$_{n=1}$
**1**	0	11%
**2**	0.025	47%
**3**	0.1	71%
**4**	0.2	92 ± 2% $_{n=3}$

Table s14: Screening of substrate loading

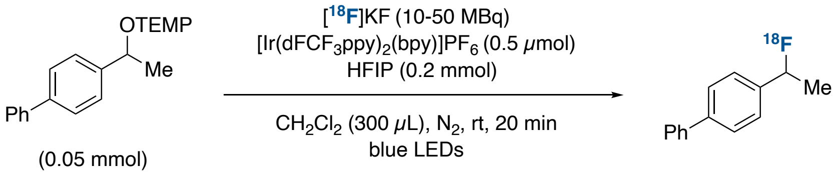

Entry	Substrate loading (mmol)	RCY$_{n=1}$
**1**	0.05	92 ± 2% $_{n=3}$
**2**	0.01	83%
**4**	0.005	66%

Table s15: Additional experiments

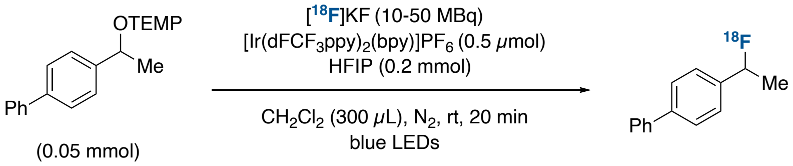

Entry	Deviation from standard conditions	$RCY_{n=1}$
**1**	none	92 ± 2% $_{n=3}$
**2**	no photocatalyst	0%
**3**	no irradiation	0%
**4**	no degassing	82%
**5**	2.0 mL solvent volume	84%
**6**	MeOH as proton source	90% ± 0% $_{n=2}$
**7**	TFA as proton source	0%
**8**	with 4CzIPN as photocatalyst	90%
**9**	with elution salts as additives (7.5 mg K_{222} + 1.5 mg K_2CO_3)	58%

Time course experiment

Procedure

Manual [^{18}F]KF elution and drying: [^{18}F]Fluoride was separated from ^{18}O-enriched-water using an anion exchange cartridge (Waters Sep-Pak AccellPlus QMA Carbonate Plus Light Cartridge), activated with H_2O (10 mL) prior to use and released with a solution of Kryptofix (7.5 mg), and K_2CO_3 (1.5 mg) in MeCN/H_2O (0.75 mL, 4:1, v/v), which was concentrated over a period of 20 min by azeotropic drying using dry MeCN (3 x 0.7 mL) under a flow of N_2 at 110 °C.

^{18}F-fluorination: To an oven-dried 4 mL glass vial equipped with a magnetic stirrer and capped with a septum was added [^{18}F]KF (10-50 MBq) in MeCN (approximately 50 µL) and the MeCN was subsequently removed by drying under a flow of N_2 at 110 °C. To the vial was then added a solution containing 1-(1-([1,1'-biphenyl]-4-yl)ethoxy)-2,2,6,6-tetramethylpiperidine **1** (16.9 mg, 0.05 mmol), [Ir(dFCF$_3$ppy)$_2$(bpy)]PF$_6$ (0.5 mg, 0.5 µmol), CH_2Cl_2 (300 µL), and HFIP (21 µL, 0.2 mmol). The mixture was degassed by nitrogen bubbling for 10 seconds, then stirred in a SynLED Parallel Photoreactor with an EvoluChem 18 W blue LED lamp (λ = 450 nm) at room temperature (with a fan) for 20 minutes. The reaction mixture was then diluted with EtOH/H_2O (300 µL, 9:1 v/v) and an aliquot was filtered and analyzed by radioHPLC. The radiochemical yield (RCY) was determined by integration of the ^{18}F-product relative to the total peak area for all radioactive species observed.

Table s16: Time course experiment

Entry	Reaction time	$RCY_{n=1}$
**1**	1 min	57%
**2**	2 min	71%
**3**	5 min	80%
**4**	10 min	81%
**5**	20 min	87%

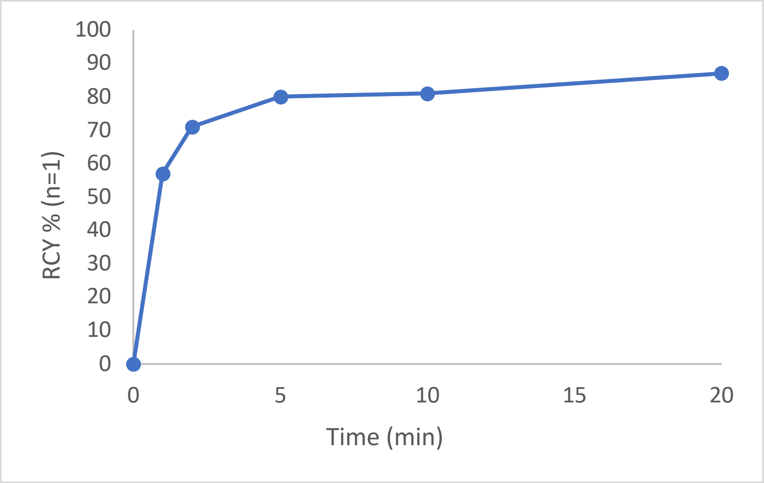

Figure s3: Plot of RCY *vs* time

Robustness screen

Procedure

Manual [^{18}F]KF elution and drying: [^{18}F]Fluoride was separated from ^{18}O-enriched-water using an anion exchange cartridge (Waters Sep-Pak AccellPlus QMA Carbonate Plus Light Cartridge), activated with H_2O (10 mL) prior to use and released with a solution of Kryptofix (7.5 mg), and K_2CO_3 (1.5 mg) in MeCN/H_2O (0.75 mL, 4:1, v/v), which was concentrated over a period of 20 min by azeotropic drying using dry MeCN (3 x 0.7 mL) under a flow of N_2 at 110 °C.

^{18}F-fluorination: To an oven-dried 4 mL glass vial equipped with a magnetic stirrer and capped with a septum was added [^{18}F]KF (10-50 MBq) in MeCN (approximately 50 µL) and the MeCN was subsequently removed by drying under a flow of N_2 at 110 °C. To the vial was then added a solution containing 1-(1-([1,1'-biphenyl]-4-yl)ethoxy)-2,2,6,6-tetramethylpiperidine **1** (16.9 mg, 0.05 mmol), [Ir(dFCF$_3$ppy)$_2$(bpy)]PF$_6$ (0.5 mg, 0.5 µmol), CH_2Cl_2 (300 µL), HFIP (21 µL, 0.2 mmol) and additive (0.05 mmol). The mixture was degassed by nitrogen bubbling for 10 seconds, then stirred in a SynLED Parallel Photoreactor with an EvoluChem 18 W blue LED lamp (λ = 450 nm) at room temperature (with a fan) for 20 minutes. The reaction mixture was then diluted with EtOH/H_2O (300 µL, 9:1 v/v) and an aliquot was filtered and analyzed by radioHPLC. The radiochemical yield (RCY) was determined by integration of the ^{18}F-product relative to the total peak area for all radioactive species observed.

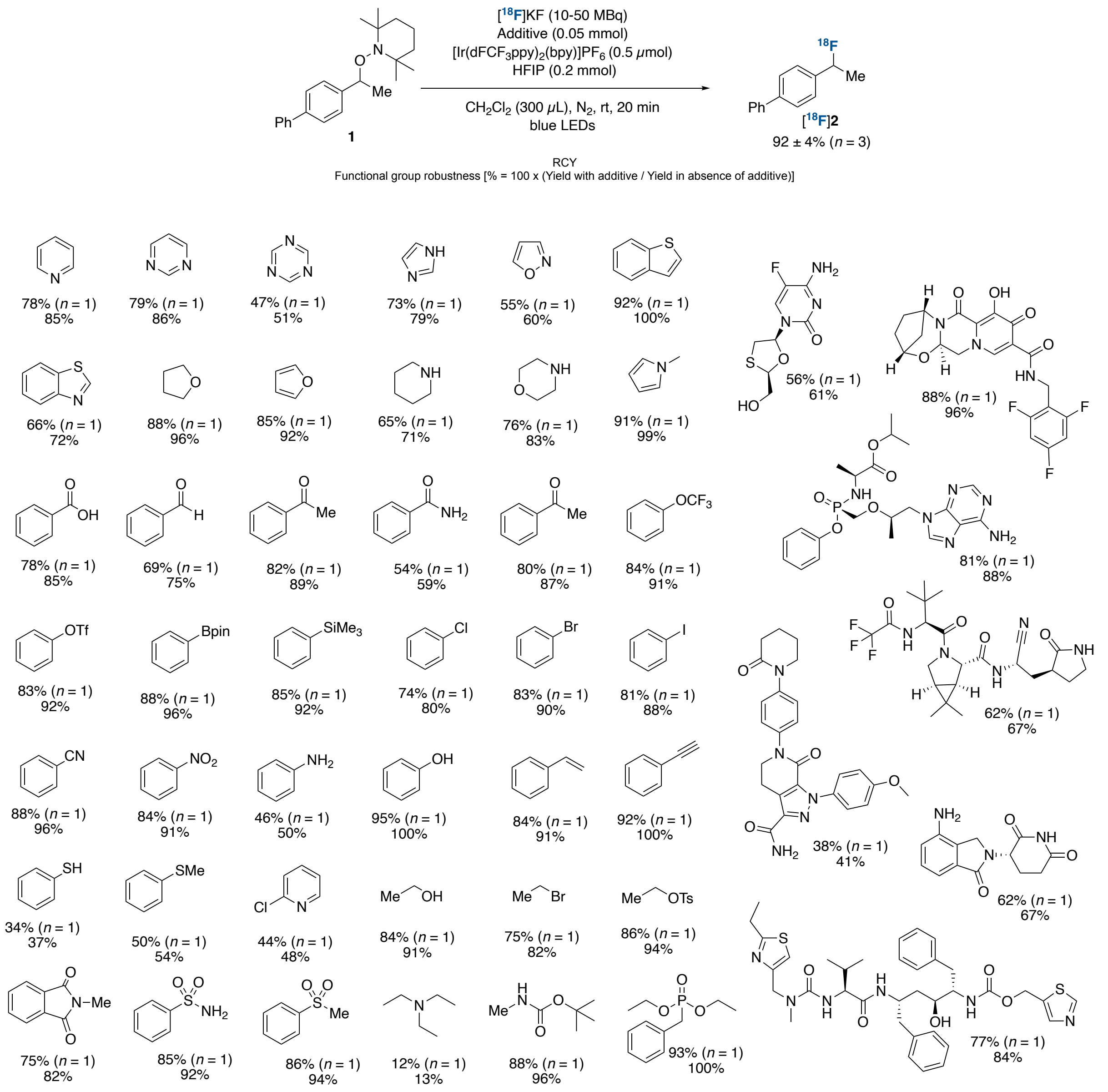

Figure s4: Outcome of the robustness screen expressed as RCY and robustness factor for each additive

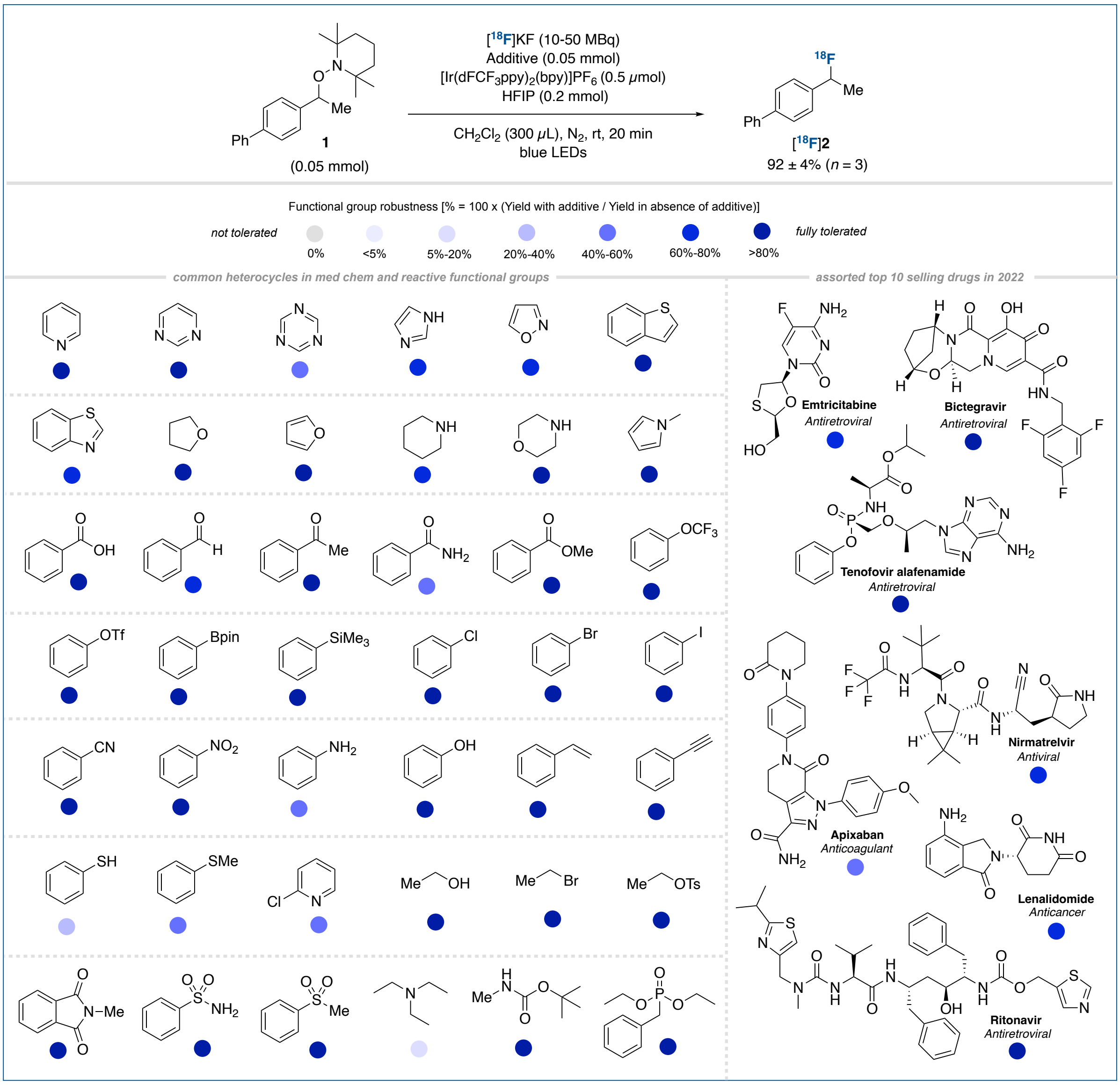

Figure s5: Outcome of the robustness screen expressed as color-coded robustness factor for each additive

Discussion

The sensitivity of the reaction to range of spiking additives was assessed. The radiochemical yield (determined by HPLC analysis of the crude reaction mixture) was then compared to the control reaction in the absence of any additive and expressed as robustness factor. Redox sensitive, electrophilic, nucleophilic, and fluorophilic functional groups were of particular interest as these may interfere with the reaction and/or [^{18}F]fluoride. In line with the mild nature of this transformation, a broad variety of reactive functional groups, scaffolds, and heterocycles, as well as a selection of top selling pharmaceuticals,[58] were found to be well tolerated. It is worth noting that the addition of triethylamine led to a significant decrease in

RCY, likely due to the fact that tertiary amines are capable of quenching the excited state of the photocatalyst.[59] In addition, highly nucleophilic functionality, such as thiophenol, was also not well tolerated. This may likely be attributed to competitive trapping of the carbocation generated in the reaction.

Radiofluorination of crude reaction mixture

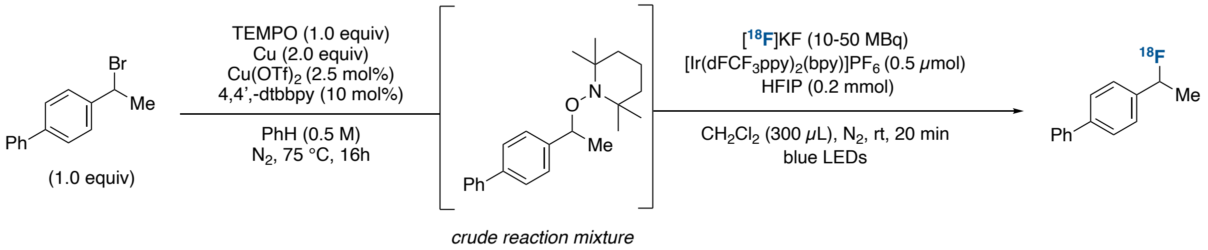

Scheme s2: Radiofluorination of crude reaction mixture

Synthesis of 1-(1-([1,1'-biphenyl]-4-yl)ethoxy)-2,2,6,6-tetramethylpiperidine

Synthesized according to an adapted literature procedure:[2]
Under air, 4-(1-bromoethyl)-1,1'-biphenyl (see synthesis of **1**) (261 mg, 1.0 mmol, 1.0 equiv), TEMPO (156 mg, 1.0 mmol, 1.0 equiv), $Cu(OTf)_2$ (9 mg, 0.025 mmol, 2.5 mol%), 4,4'-di-*tert*-butyl-2,2'-bipyridine (27 mg, 0.1 mmol, 10.0 mol%), and Cu powder (127 mg, 2.0 mmol, 2.0 equiv) were added into a flame-dried Schlenk tube equipped with a magnetic stirrer. The flask was evacuated and backfilled with N_2 three times. Anhydrous and degassed benzene (2.0 mL, 0.5 M) was then added. The reaction mixture was stirred at 75°C for 16 h under N_2, then cooled to room temperature. The solids were then removed by filtration of the reaction mixture through a syringe filter (fisherbrand PTFE 0.2 μm). The filtrate was then diluted with 200 μL of anhydrous CH_2Cl_2.

Procedure for the radiofluorination of 1-(1-([1,1'-biphenyl]-4-yl)ethoxy)-2,2,6,6-tetramethylpiperidine

Manual [^{18}F]KF elution and drying: [^{18}F]Fluoride was separated from ^{18}O-enriched-water using an anion exchange cartridge (Waters Sep-Pak AccellPlus QMA Carbonate Plus Light Cartridge), activated with H_2O (10 mL) prior to use and released with a solution of Kryptofix

(7.5 mg), and K_2CO_3 (1.5 mg) in MeCN/H_2O (0.75 mL, 4:1, v/v), which was concentrated over a period of 20 min by azeotropic drying using dry MeCN (3 x 0.7 mL) under a flow of N_2 at 110 °C.

^{18}F-fluorination: To an oven-dried 4 mL glass vial equipped with a magnetic stirrer and capped with a septum was added [^{18}F]KF (10-50 MBq) in MeCN (approximately 50 µL) and the MeCN was subsequently removed by drying under a flow of N_2 at 110 °C. To the vial were then added a 300 µL aliquot of the crude reaction mixture, [Ir(dFCF$_3$ppy)$_2$(bpy)]PF$_6$ (0.5 mg, 0.5 µmol) and HFIP (21 µL, 0.2 mmol). The mixture was degassed by nitrogen bubbling for 10 seconds, then stirred in a SynLED Parallel Photoreactor with an EvoluChem 18 W blue LED lamp (λ = 450 nm) at room temperature (with a fan) for 20 minutes. The reaction mixture was then diluted with EtOH/H_2O (300 µL, 9:1 v/v) and an aliquot was filtered and analyzed by radioHPLC. The radiochemical yield (RCY) was determined by integration of the ^{18}F-product relative to the total peak area for all radioactive species observed.

Entry	RCY (%)
**1**	95
**2**	96
**3**	93

Average RCY: 95 ± 1% (n = 3)

Procedure for the control experiments with 4-(1-bromoethyl)-1,1'-biphenyl

Manual [^{18}F]KF elution and drying: ^{18}F-Fluoride was separated from ^{18}O-enriched-water using an anion exchange cartridge (Waters Sep-Pak AccellPlus QMA Carbonate Plus Light Cartridge), activated with H_2O (10 mL) prior to use and released with a solution of Kryptofix (7.5 mg), and K_2CO_3 (1.5 mg) in MeCN/H_2O (0.75 mL, 4:1, v/v), which was concentrated over a period of 20 min by azeotropic drying using dry MeCN (3 x 0.7 mL) under a flow of N_2 at 110 °C.

^{18}F-fluorination: To an oven-dried 3 mL v-vial equipped with a magnetic stir and capped with a septum bar was added 4-(1-bromoethyl)-1,1'-biphenyl **1a** (13 mg, 0.05 mmol) followed by anhydrous MeCN (300 µL) and [^{18}F]KF (10-50 MBq) in MeCN (approximately 50 µL). The reaction mixture was then stirred at the given temperature for 20 minutes. The reaction mixture was then diluted with EtOH/H_2O (300 µL, 1:1 v/v) and an aliquot was filtered and analyzed by

radioHPLC. The radiochemical yield (RCY) was determined by integration of the ^{18}F-product relative to the total peak area for all radioactive species observed.

Table s17: Radiofluorination of 4-(1-bromoethyl)-1,1'-biphenyl under thermal activation

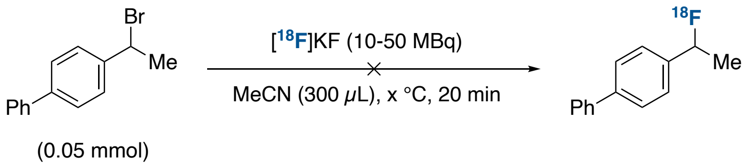

Entry	Temperature	RCY$_{n=1}$
**1**	rt	0%
**2**	80 °C	0%
**3**	100 °C	0%

Alternative [^{18}F]KF elution and drying conditions

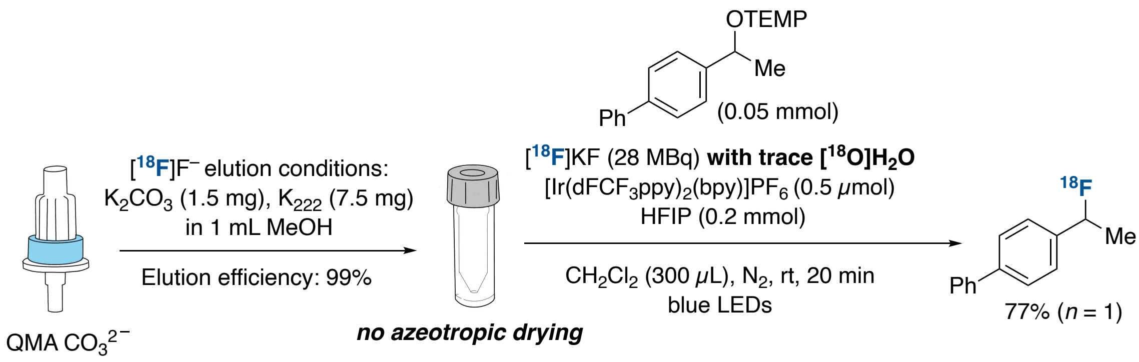

Scheme s3: Azeotropic drying-free radiofluorination

Manual [^{18}F]KF elution and drying: [^{18}F]fluoride (10-50 MBq) was separated from ^{18}O-enriched-water using an anion exchange cartridge (activated with H_2O (10 mL) prior to use). The cartridge was dried by syringe with air (10 mL). The activity trapped on the cartridge was measured in a dose calibrator. The cartridge was moved to an empty vial (charged with a stirrer bar, as necessary) and eluted with a solution of Kryptofix (7.5 mg), and K_2CO_3 (1.5 mg) in MeOH (1.0 mL). The eluate was measured in a dose calibrator, as well as the activity trapped on the cartridge. Elution efficiency was then calculated. The eluate was then concentrated over a period of 10 min under a flow of N_2 at 110 °C with stirring.

^{18}**F-fluorination:** To the vial containing [^{18}F]KF was added a solution containing 1-(1-([1,1'-biphenyl]-4-yl)ethoxy)-2,2,6,6-tetramethylpiperidine **1** (16.9 mg, 0.05 mmol), [Ir(dFCF$_3$ppy)$_2$(bpy)]PF$_6$ (0.5 mg, 0.5 μmol), CH_2Cl_2 (300 μL) and HFIP (21 μL, 0.2 mmol). The mixture was degassed by nitrogen bubbling for 10 seconds, then stirred in a SynLED Parallel Photoreactor with an EvoluChem 18 W blue LED lamp (λ = 450 nm) at room temperature (with a fan) for 20 minutes. The reaction mixture was then diluted with EtOH/H_2O (300 μL, 9:1 v/v) and an aliquot was filtered and analyzed by radioHPLC. The radiochemical yield (RCY) was determined by integration of the ^{18}F-product relative to the total peak area for all radioactive species observed.

Automated radiosynthesis

The automated radiosynthesis of [^{18}F]**2** was performed with a Trasis AllInOne synthesizer using an automated program and pre-built cassette (Figure s6). The vial in slot 2 was charged with Kryptofix® 222 (7.5 mg), K_2CO_3 (0.15 mL, 10 mg/mL aqueous solution) and MeCN (0.6 mL). The amber vial in slot 9 was charged with 1-(1-([1,1'-biphenyl]-4-yl)ethoxy)-2,2,6,6-tetramethylpiperidine **1** (0.05 mmol), [Ir(dFCF$_3$ppy)$_2$(bpy)]PF$_6$ (0.5 μmol) and anhydrous CH_2Cl_2 (1.0 mL). The solvent reservoirs (slots 8 and 17) were filled with anhydrous MeCN (ca. 10 mL) and MeCN/H_2O (3:1 v/v, ca. 10 mL) (slot 10). The Waters Sep-Pak AccellPlus QMA Carbonate Plus Light Cartridge (slot 5) was activated with water (10 mL) prior to use. After [^{18}F]fluoride in [^{18}O]water was received from the cyclotron, it was trapped on a Waters Sep-Pak AccellPlus QMA Carbonate Plus Light cartridge. [^{18}F]Fluoride was then eluted from the cartridge with a solution of K_{222} and K_2CO_3, and this was dried under a flow of air at 120°C. Once drying was complete, the reagents mixture (slot 9) was transferred to the reactor. The resulting mixture was then taken up in a 3 mL syringe (slot 11) and transferred back to the reactor for mixing with [^{18}F]Fluoride. This step was repeated twice. The reaction mixture was finally taken up in a 3 mL syringe (slot 11) and pushed through the photoflow reactor (flow rate = 0.4 mL/min, residence time = 5 min) and collected into the reactor. The loop was then purged with 3 mL of air and the reaction mixture was dried under a flow of air at 50 °C. Thereafter, the loop was rinsed twice with MeCN/H_2O (2 x 2 mL, 3:1 v/v) and this was transferred to the reactor and then to the HPLC sample loop. The crude reaction mixture was purified by reverse-phase semi-preparative HPLC (MeCN/H_2O as eluent, Q = 4 ml/min, HPLC conditions E). The eluate was diluted with water (20 mL). This mixture was then loaded as a

1:1 mixture with additional water onto a Waters Sep-Pak C18 Plus cartridge (preconditioned with EtOH (10 mL), then water (10 mL)), and then washed with water (10 mL) and purged with air. The desired radiofluorinated product was eluted from the C18 cartridge with MeCN (2 mL) and transferred to a vial for analysis. The activity of the collected product was measured in a dose calibrator. An aliquot (ca 20 MBq) of the isolated product was analyzed by radio-HPLC to determine molar activity. HPLC analysis was performed under HPLC conditions C. The molar activity was determined on a chemically and radiochemically pure sample. The same sample was injected in triplicate, with the injected activity and time of injection recorded. The UV response corresponding to the desired radiofluorinated product was then integrated, to give the amount of non-radioactive product that was detected. Molar activity was then calculated.

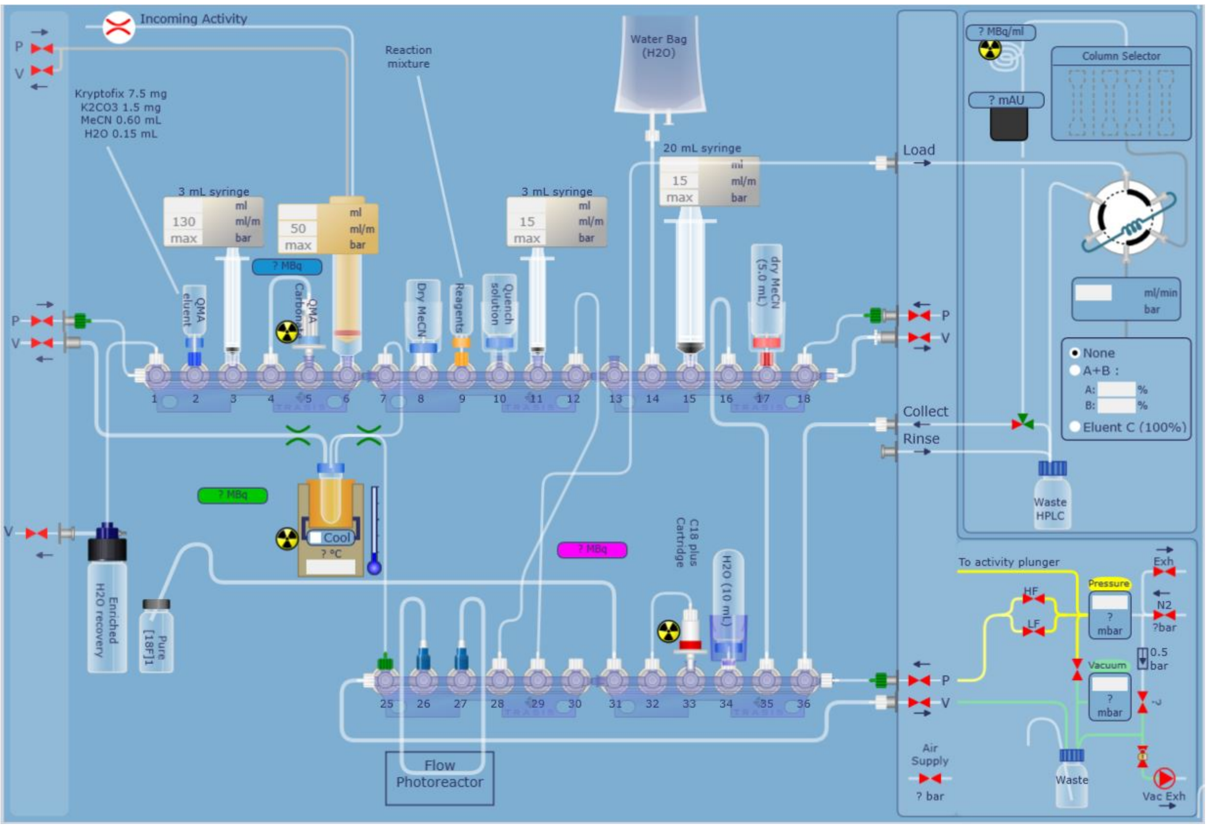

Figure s6: Cassette set-up for the automated photoredox radiofluorination of alkoxyamines using a Trasis AllInOne automated platform.

Procedure for radiosynthesis of [^{18}F]2: The general procedure was followed using 1-(1-([1,1'-biphenyl]-4-yl)ethoxy)-2,2,6,6-tetramethylpiperidine **1** (16.9 mg, 0.05 mmol). HPLC purification was carried out using MeCN/H_2O = 60/:40, v/v, as eluent, t_R([^{18}F]**3**) = 14-18 min. Synthesis data are summarised below (Table s18).

Table s18: Radiosynthesis of **[^{18}F]2**

Starting activity	AY	Radiosynthesis time	RCP	A_m
10 GBq	1.93 GBq	89 min	> 99%	219 GBq/µmol

Procedure for the calibration curve of 2: A calibration curve for authentic reference **2** was recorded by preparing samples of a range of concentrations by serial dilution, starting with a solution of **2** (2.0 mg) in EtOH (1.0 mL) (Figure s7). These were injected onto an HPLC (10 µL injection volume from a 1.0 mL stock, HPLC conditions C) and the UV response was measured by integrating the peak of interest.

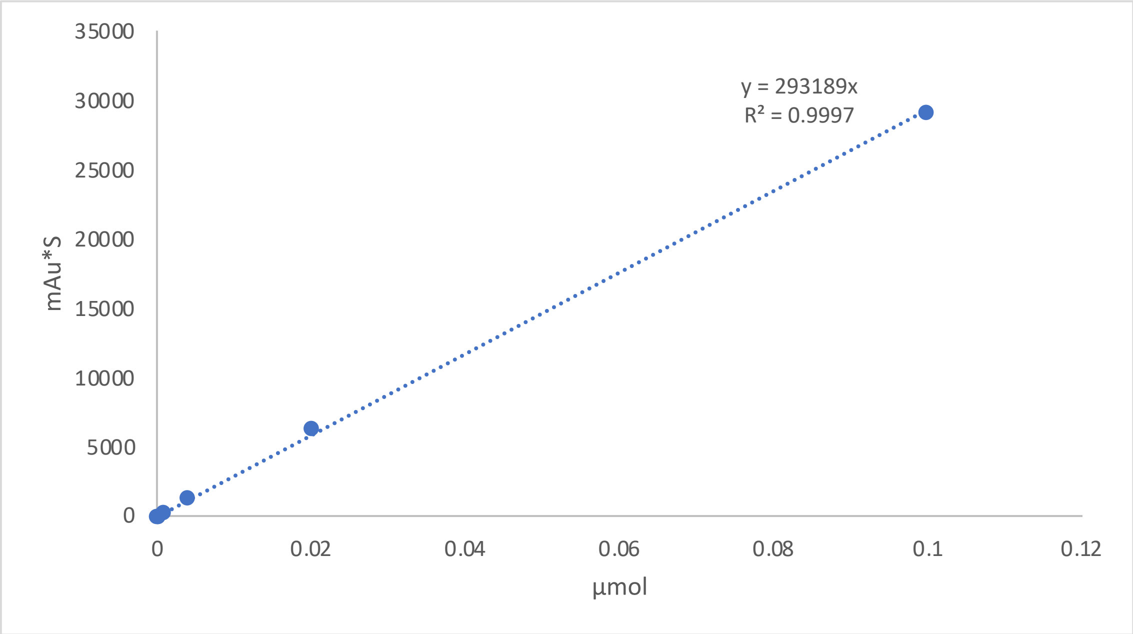

Figure s7: HPLC calibration curve for authentic reference **2**

Molar activity was then calculated. These data are summarised in Table s19.

Table s19: Molar activity calculation of **[^{18}F]2**

Measurement	Activity injected (MBq, d.c.)	Peak area (mAu*s)	2 injected	A_m (GBq/µmol)
1	0.56	1.0	3.41 x 10^{-6}	164
2	0.74	0.9	3.07 x 10^{-6}	241
3	0.69	0.8	2.73 x 10^{-6}	253

Average A_m: 219 ± 39 GBq/µmol

The automated radiosynthesis of [^{18}F]**2** was then repeated following the same procedure and molar activity was calculated. These data are summarised in below.

Table s20: Radiosynthesis of **[^{18}F]2** (n = 2)

Starting activity	AY	Radiosynthesis time	RCP	A_m
10 GBq	3.17 GBq	89 min	> 99%	111 GBq/µmol

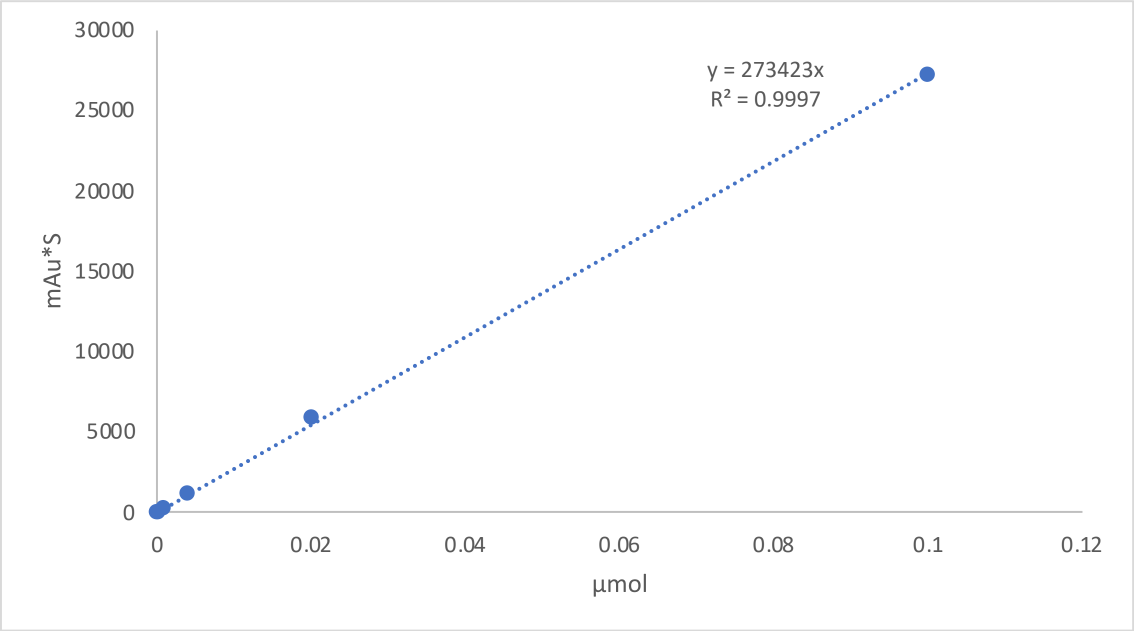

Figure s8: HPLC calibration curve for authentic reference **2**

Table s21: Molar activity calculation of **[^{18}F]2** (n = 2)

Measurement	Activity injected (MBq, d.c.)	Peak area (mAu*s)	2 injected	A_m (GBq/µmol)
1	1.35	3.1	11.34×10^{-6}	119
2	1.35	3.3	12.07×10^{-6}	112
3	1.20	3.2	11.7×10^{-6}	103

Average A_m: 111 ± 7 GBq/µmol

Overall average A_m: 165 ± 54 GBq/µmol (n = 2)

Radiotrace overlays and radiochemical yields

General procedure for the radiofluorination of alkoxyamines

Manual [^{18}F]KF elution and drying: [^{18}F]Fluoride was separated from ^{18}O-enriched-water using an anion exchange cartridge (Waters Sep-Pak AccellPlus QMA Carbonate Plus Light Cartridge), activated with H_2O (10 mL) prior to use and released with a solution of Kryptofix (7.5 mg), and K_2CO_3 (1.5 mg) in MeCN/H_2O (0.75 mL, 4:1, v/v), which was concentrated over a period of 20 min by azeotropic drying using dry MeCN (3 x 0.7 mL) under a flow of N_2 at 110 °C.

^{18}F-fluorination: To an oven-dried 4 mL glass vial equipped with a magnetic stirrer and capped with a septum was added [^{18}F]KF (10-50 MBq) in CH_2Cl_2 (approximately 50 µL). Alternatively, [^{18}F]KF (10-50 MBq) in MeCN (approximately 50 µL) was added and the MeCN was subsequently removed by drying under a flow of N_2 at 110 °C. To the vial was then added a solution containing alkoxyamine (0.05 mmol), [Ir(dFCF$_3$ppy)$_2$(bpy)]PF$_6$ (0.5 mg, 0.5 µmol), CH_2Cl_2 (300 µL) and HFIP (21 µL, 0.2 mmol). The mixture was degassed by nitrogen bubbling for 10 seconds, then stirred in a SynLED Parallel Photoreactor with an EvoluChem 18 W blue LED lamp (λ = 450 nm) at room temperature (with a fan) for 20 minutes. The reaction mixture was then diluted with EtOH/H_2O (300 µL, 9:1 v/v) and an aliquot was filtered and analyzed by radioHPLC. The radiochemical yield (RCY) was determined by integration of the ^{18}F-product relative to the total peak area for all radioactive species observed.

[^{18}F]4-(1-((1,1,1,3,3,3-Hexafluoropropan-2-yl)oxy)ethyl)-1,1'-biphenyl ([^{18}F]2)

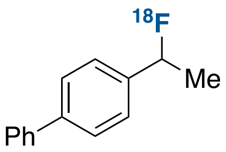

Entry	RCY (%)
**1**	97
**2**	92
**3**	87

Average RCY: 92 ± 4% (n = 3)

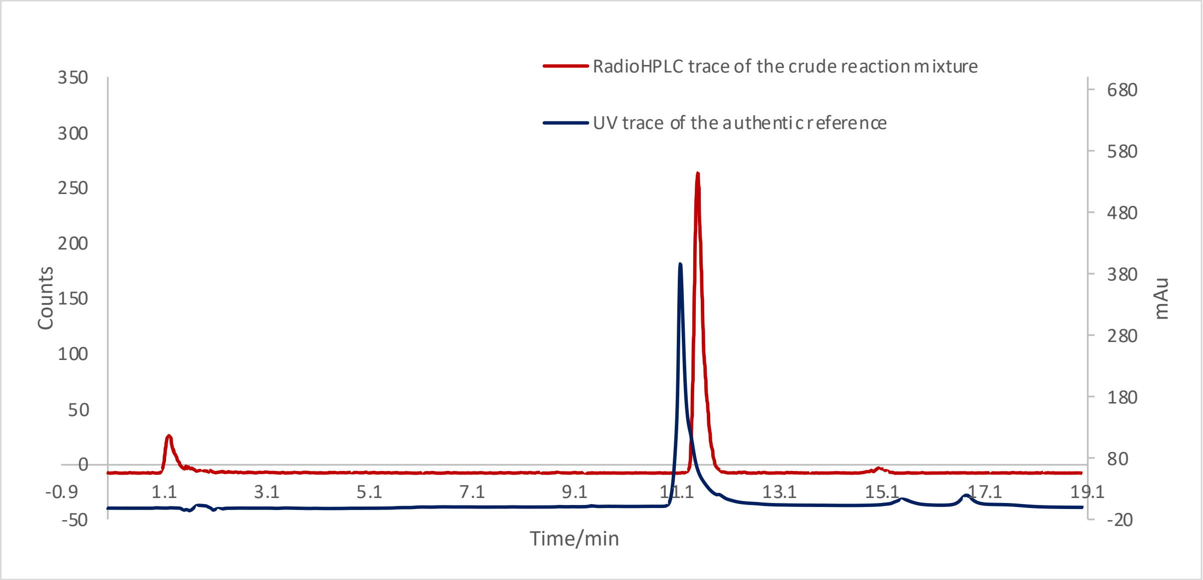

HPLC conditions A

[^{18}F](1-Fluoroethyl)benzene ([^{18}F]5)

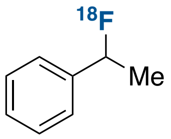

Entry	RCY (%)
1	58
2	72
3	78

Average RCY: 69% ± 8% (n = 3)

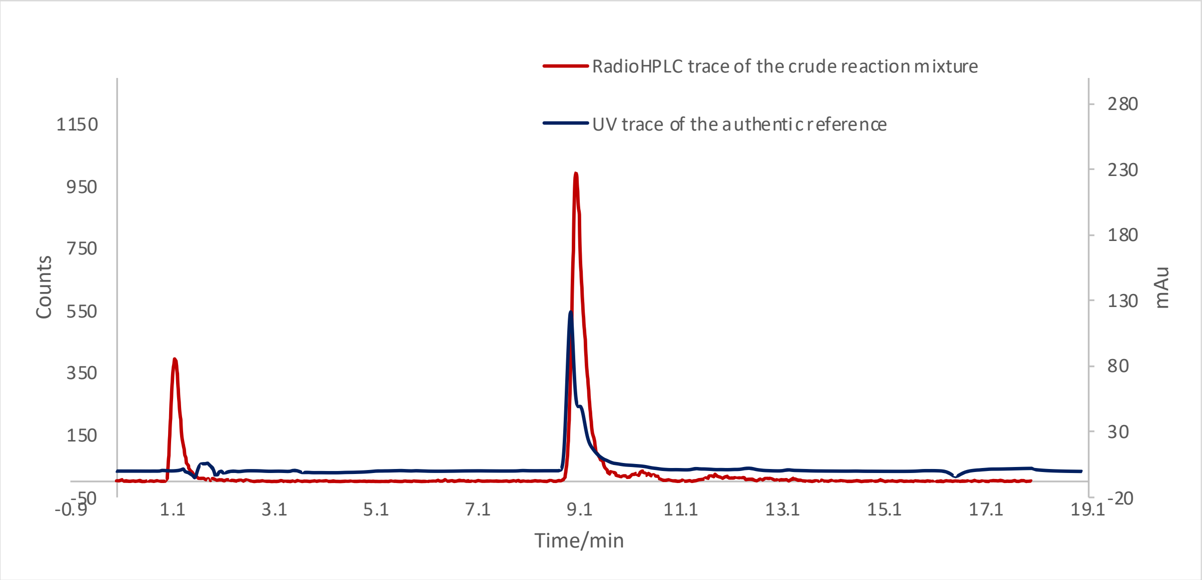

HPLC conditions A

[^{18}F]1-(1-fluoroethyl-1,2,2,2-*d*$_4$)benzene-2,3,4,5,6-*d*$_5$ ([^{18}F]6)

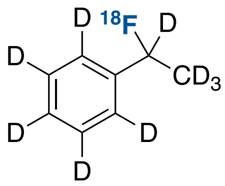

Entry	RCY (%)
1	82
2	76
3	87

Average RCY: 82 ± 5% (*n* = 3)

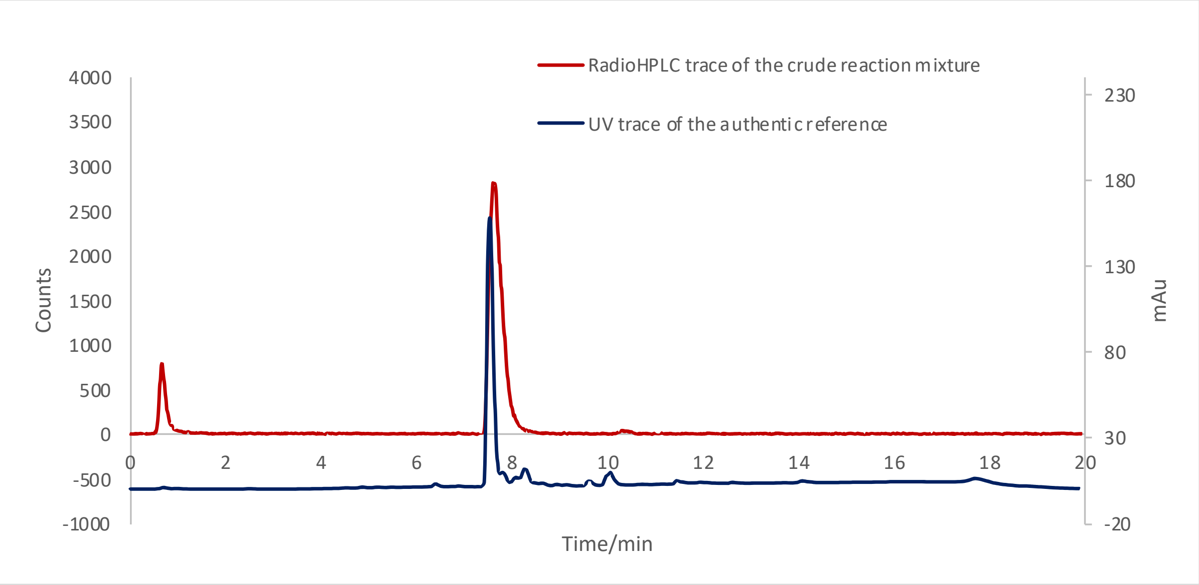

HPLC conditions C

Note: Authentic reference **5** was used.

[^{18}F]1-(1-Fluoroethyl)-4-methylbenzene ([^{18}F]7)

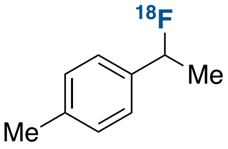

Entry	RCY (%)
1	87
2	85
3	82

Average RCY: 85 ± 2% (n = 3)

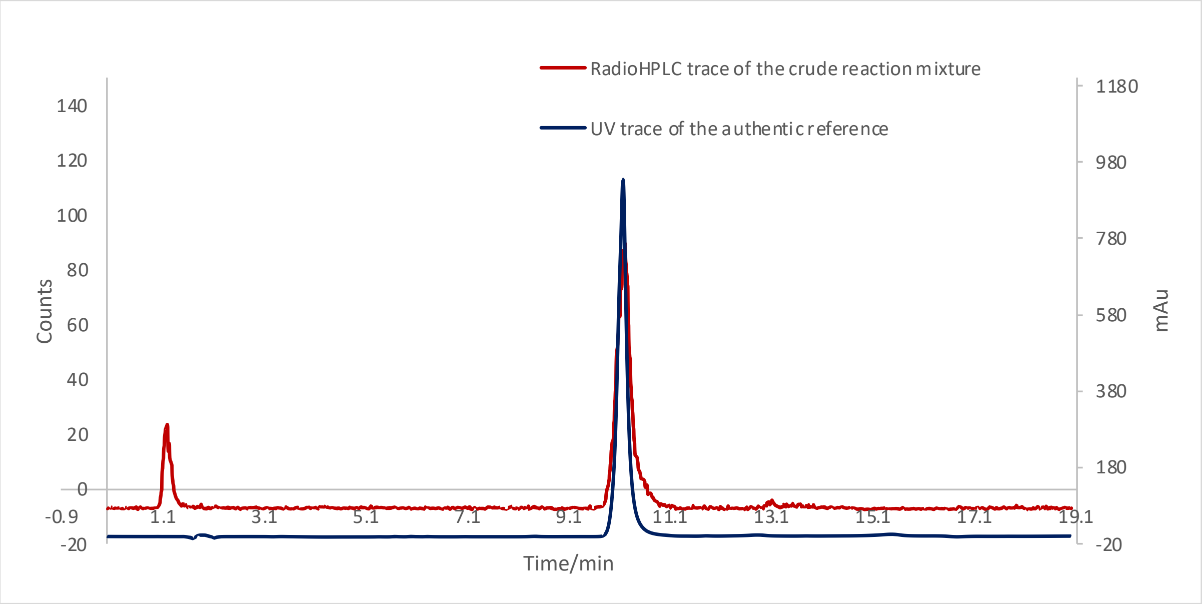

HPLC conditions A

[^{18}F]1-(1-Fluoroethyl)-4-isobutylbenzene ([^{18}F]8)

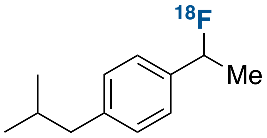

Entry	RCY (%)
**1**	96
**2**	83
**3**	89

Average RCY: 89 ± 5% (n = 3)

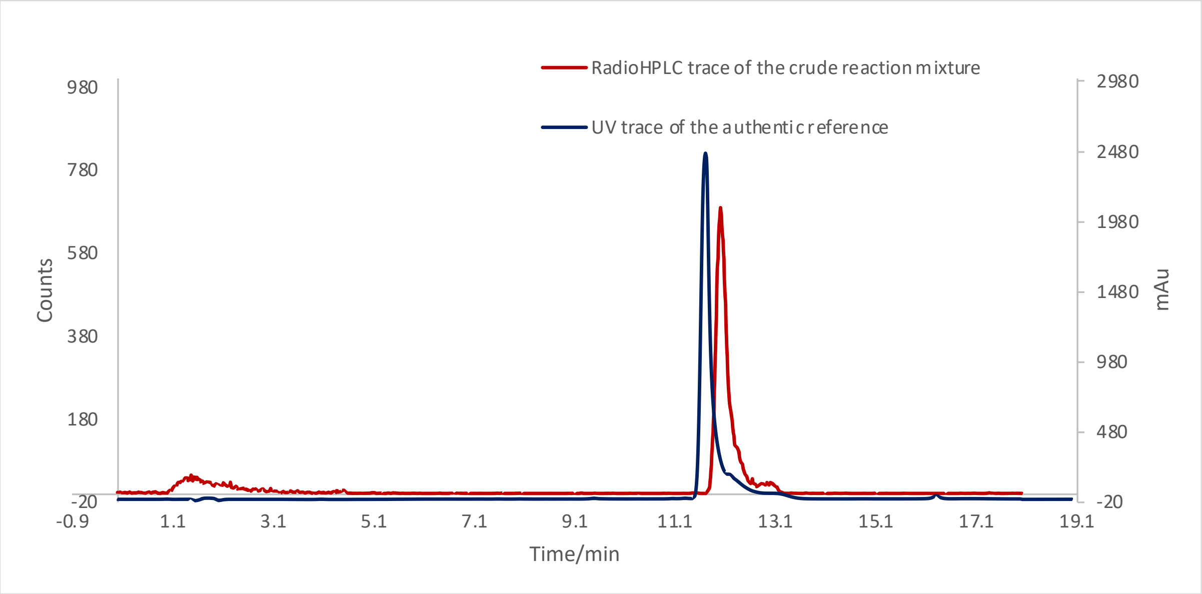

HPLC conditions A

[^18F]1-(1-Fluoroethyl)-4-vinylbenzene ([^18F]9)

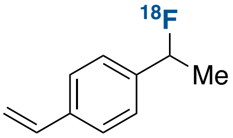

Entry	RCY (%)
**1**	73
**2**	62
**3**	68

Average RCY: 68 ± 5% (n = 3)

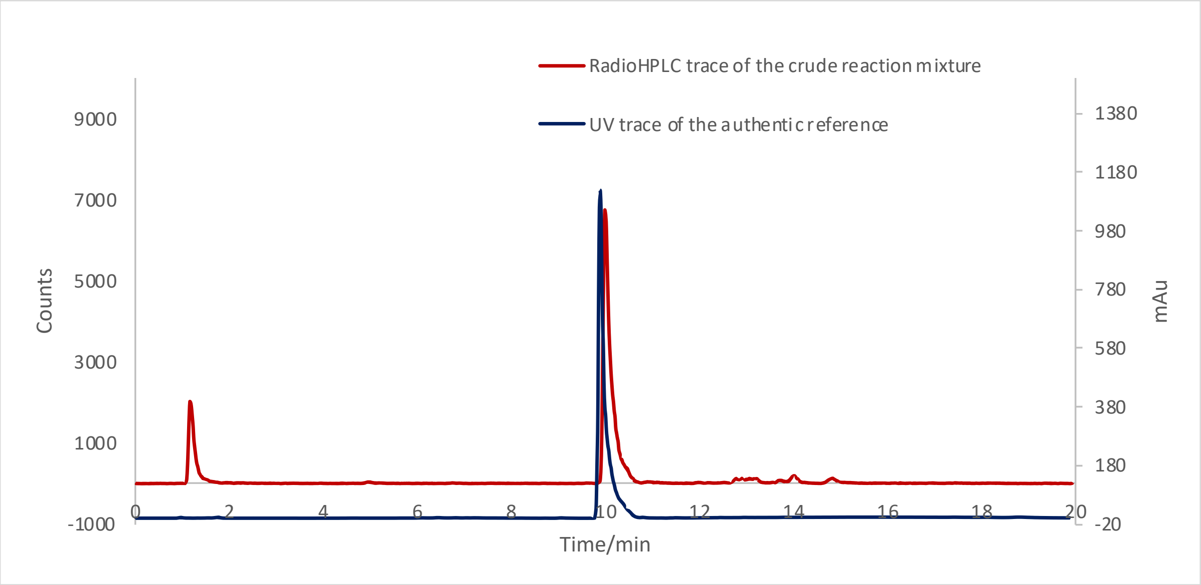

HPLC conditions C

[^{18}F]1-(1-Fluoroethyl)-2-methyl-3-(trifluoromethyl)benzene ([^{18}F]10)

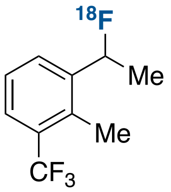

Entry	RCY (%)
1	62
2	80
3	79

Average RCY: 74 ± 8% (n = 3)

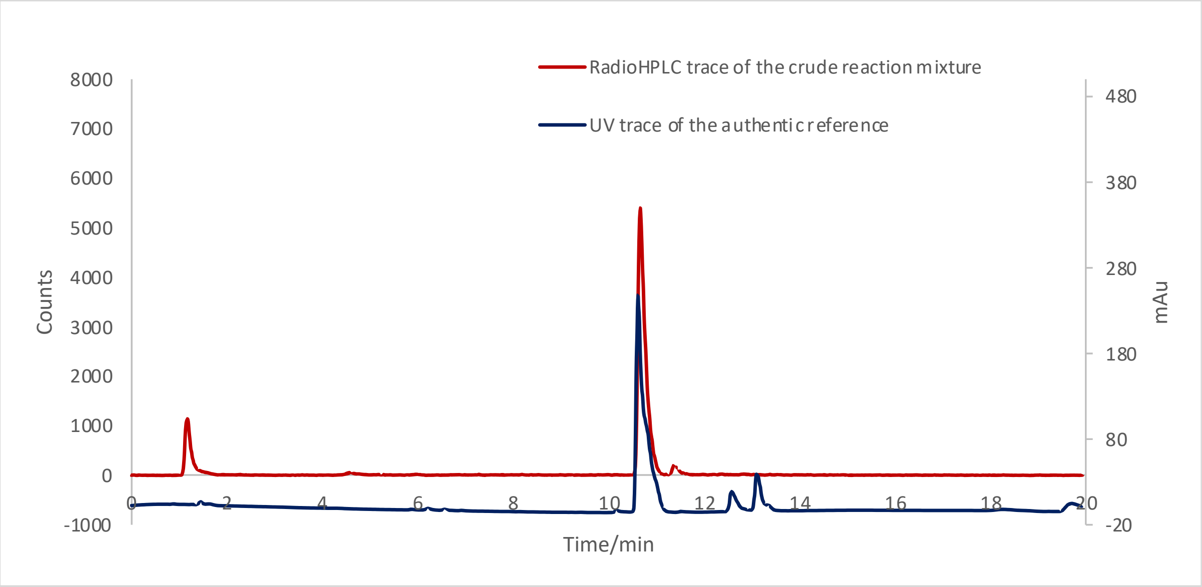

HPLC conditions C

[^{18}F]2-Fluoro-4-(1-fluoroethyl)-1,1'-biphenyl ([^{18}F]11)

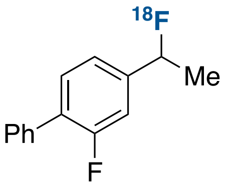

Entry	RCY (%)
**1**	83
**2**	73
**3**	76

Average RCY: 77 ± 4% (n = 3)

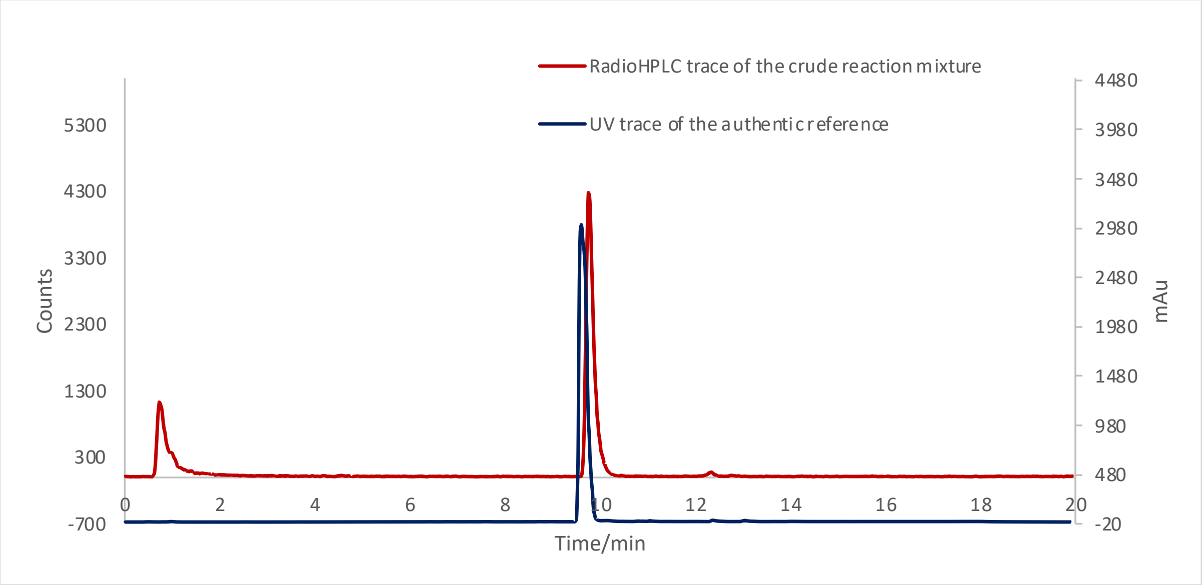

HPLC conditions C

[^{18}F]1-(1-Fluoroethyl)-3-(trifluoromethoxy)benzene ([^{18}F]12)

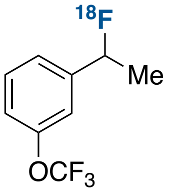

Entry	RCY (%)
**1**	32
**2**	20
**3**	26

Average RCY: 26 ± 5% (n = 3)

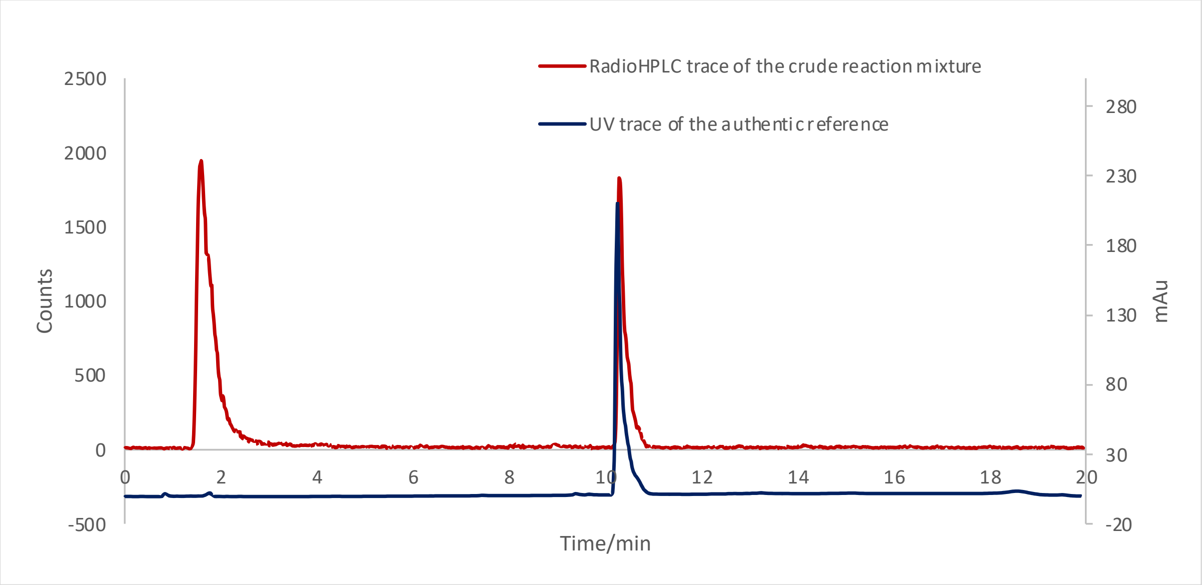

HPLC conditions C

[^{18}F]Methyl 4-(1-fluoroethyl)benzoate ([^{18}F]13)

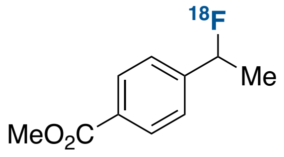

Entry	RCY (%)
**1**	54
**2**	48
**3**	40

Average RCY: 47 ± 6% (n = 3)

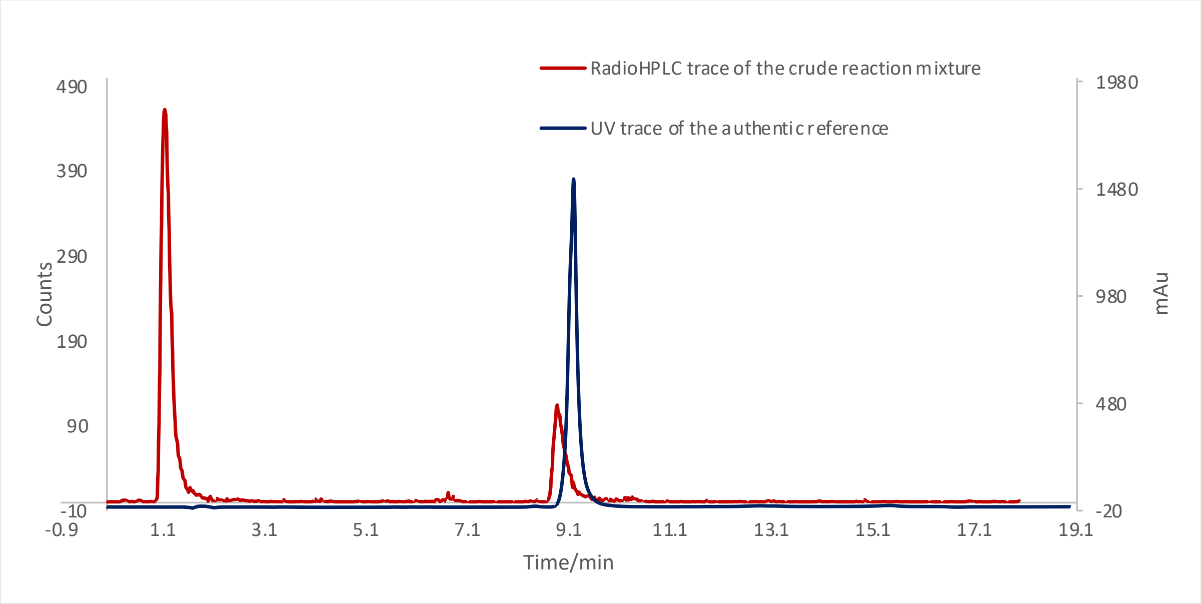

HPLC conditions A

[^{18}F]1-Bromo-4-(1-fluoroethyl)benzene ([^{18}F]14)

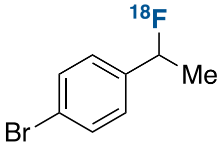

Entry	RCY (%)
1	63
2	86
3	85

Average RCY: 78 ± 11% (*n* = 3)

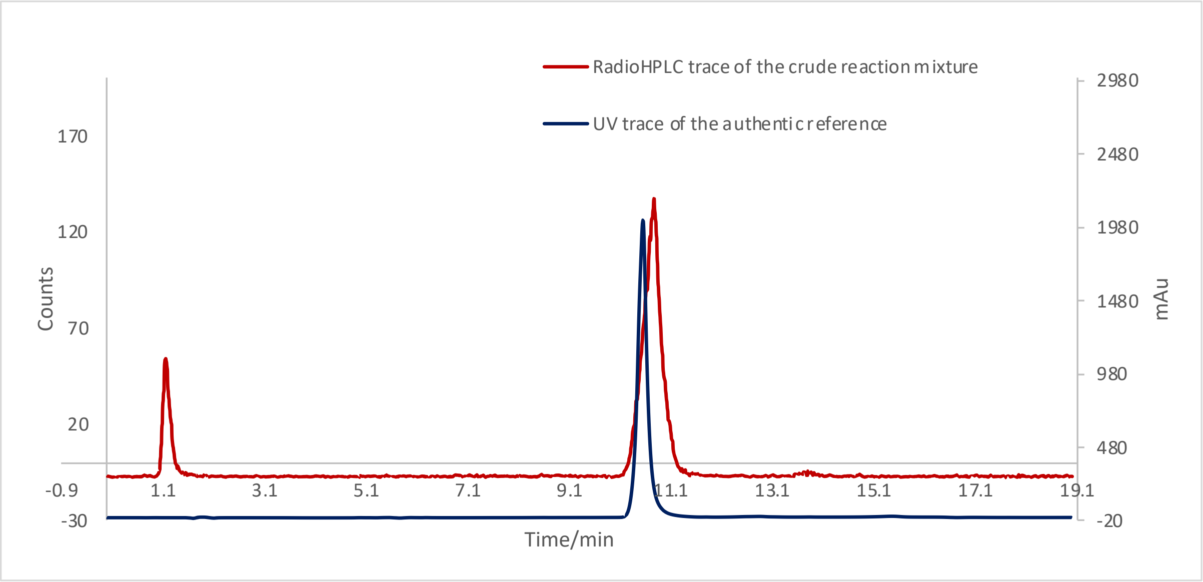

HPLC conditions A

[^{18}F]4-(1-Fluoroethyl)benzonitrile ([^{18}F]15)

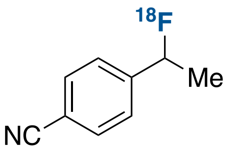

Entry	RCY (%)
**1**	14
**2**	8
**3**	10

Average RCY: 11 ± 3% (n = 3)

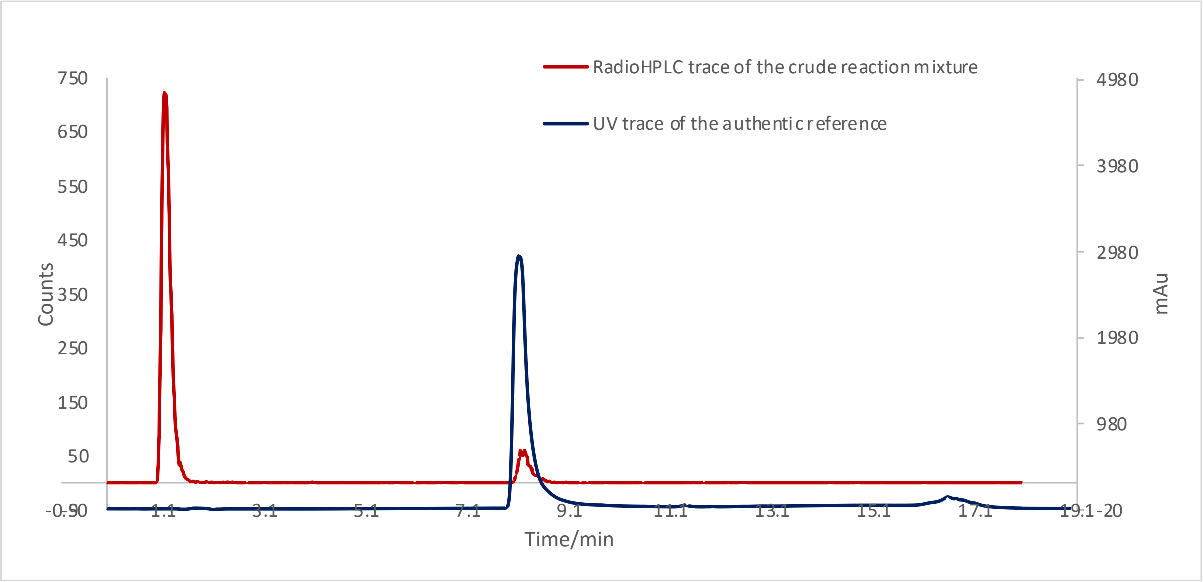

HPLC conditions A

[^{18}F]2-(4-(1-Fluoroethyl)phenyl)-4,4,5,5-tetramethyl-1,3,2-dioxaborolane ([^{18}F]16)

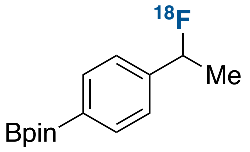

Entry	RCY (%)
1	53
2	43
3	18

Average RCY: 38 ± 15% (n = 3)

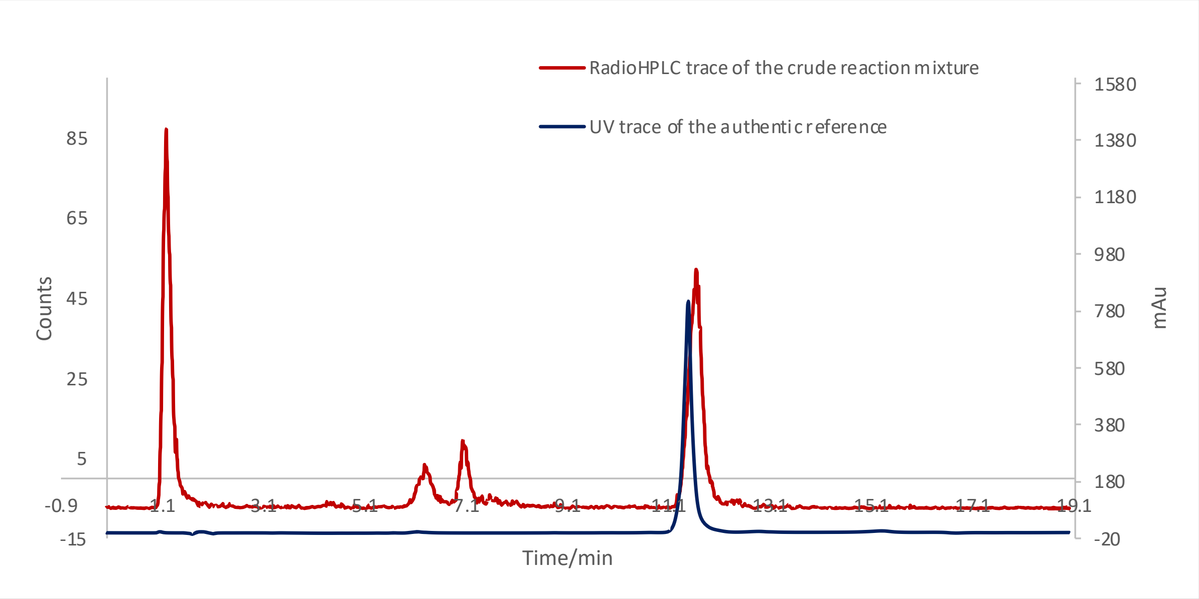

HPLC conditions A

[^{18}F](3-(1-Fluoroethyl)phenyl)(phenyl)methanone ([^{18}F]17)

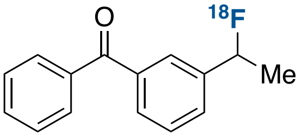

Entry	RCY (%)
1	15
2	36
3	21

Average RCY: 24 ± 9% (n = 3)

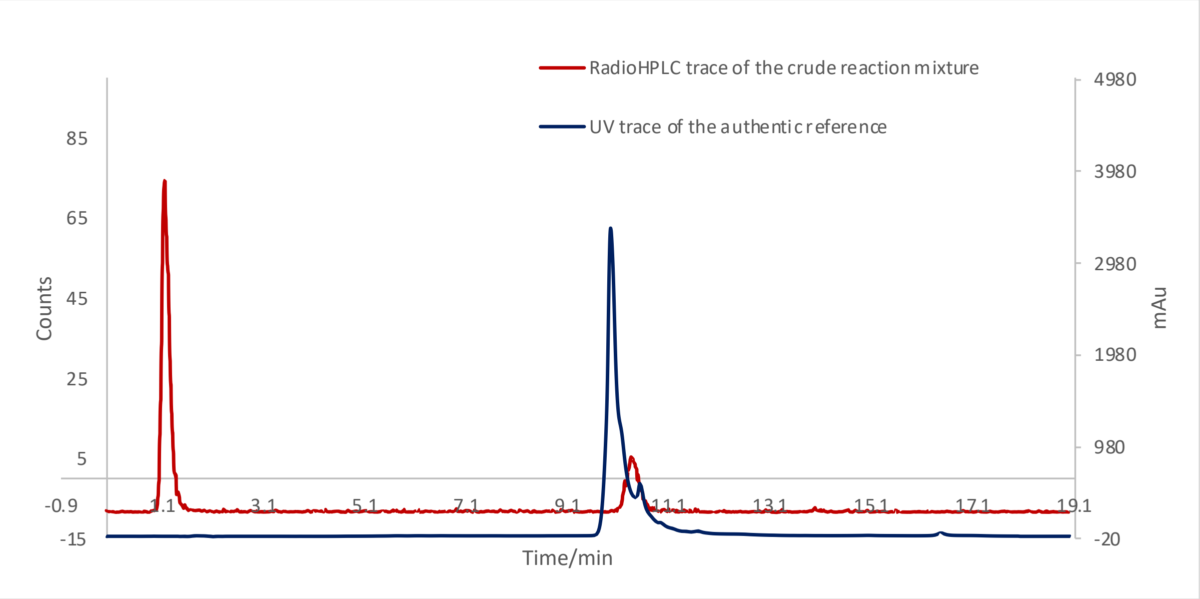

HPLC conditions A

[^{18}F](3-(4-(1-Fluoroethyl)benzyl)cyclopentan-1-one ([18)

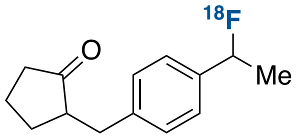

Entry	RCY (%)
**1**	56
**2**	62
**3**	63

Average RCY: 60 ± 3% (n = 3)

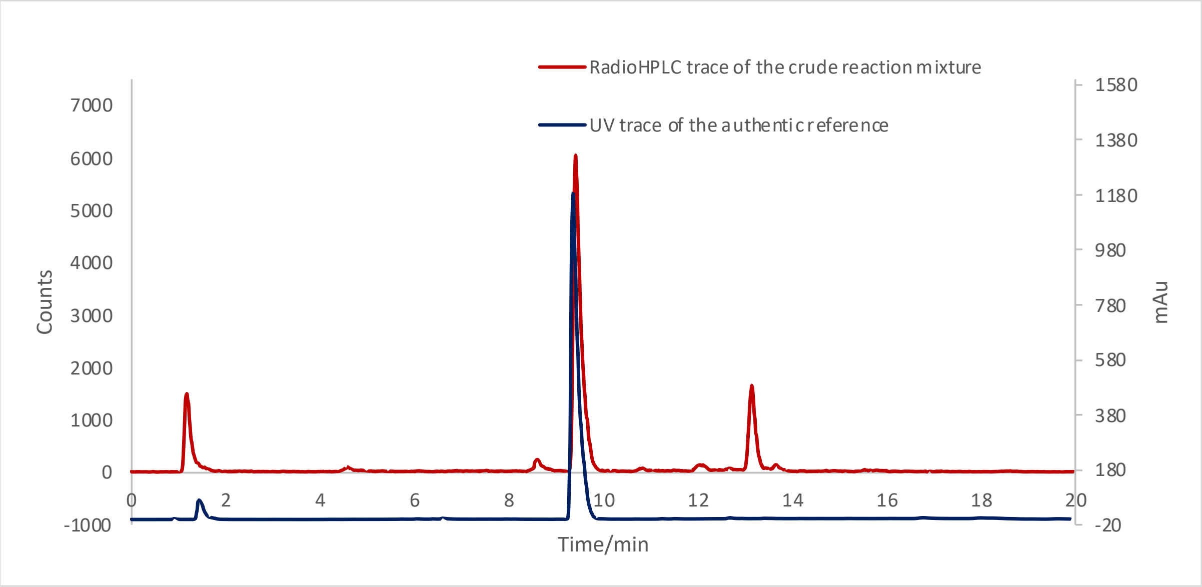

HPLC conditions C

Note: diastereomers were not resolved.

[^{18}F]*N*-(4-(1-fluoroethyl)benzyl)cyclopropanesulfonamide ([^{18}F](19)

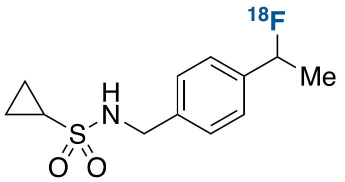

Entry	RCY (%)
1	74
2	70
3	65

Average RCY: 70 ± 4% (*n* = 3)

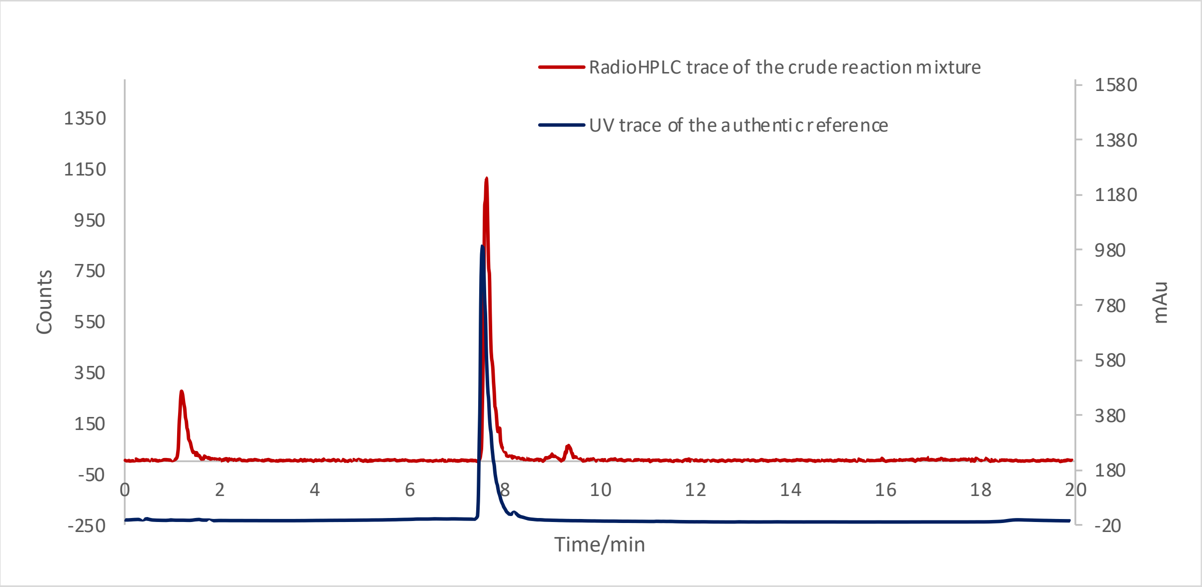

HPLC conditions C

[^{18}F]1-(1-Fluoroethyl)-4-((4-iodophenoxy)methyl)benzene ([^{18}F]20)

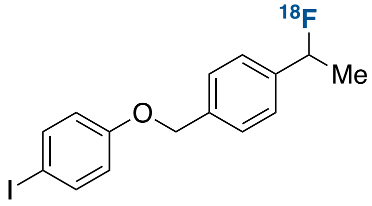

Entry	RCY (%)
**1**	70
**2**	90
**3**	92

Average RCY: 84 ± 10% (n = 3)

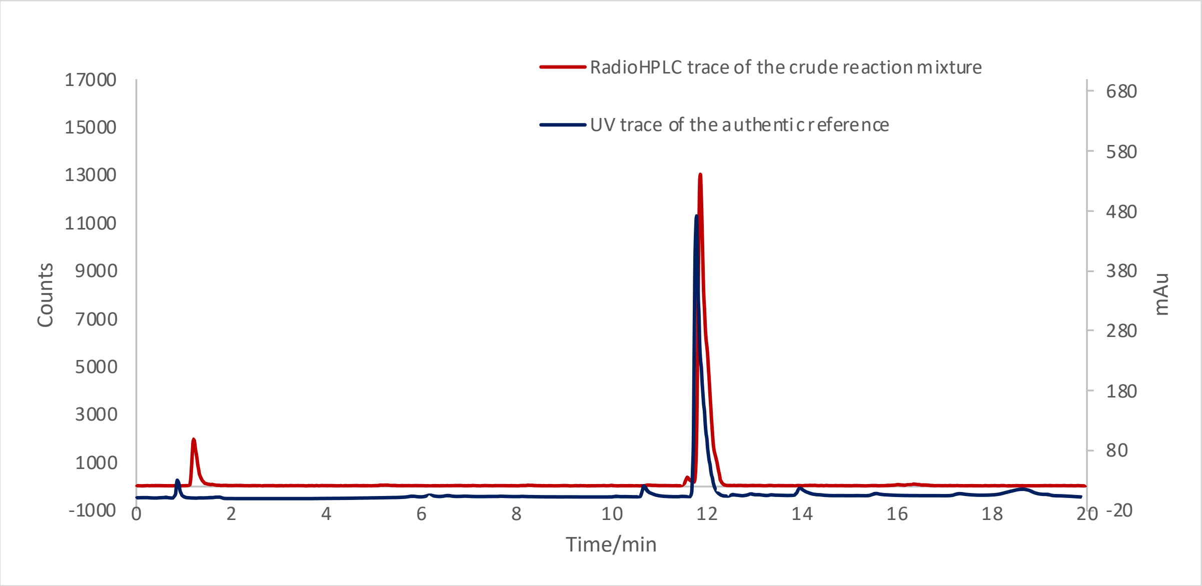

HPLC conditions C

[^{18}F]1-(1-Fluoroethyl)-4-(((4-(methylsulfonyl)benzyl)oxy)methyl)benzene ([^{18}F]21)

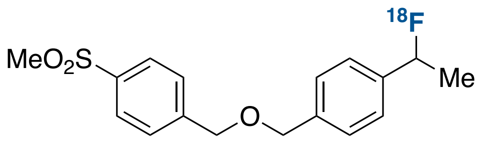

Entry	RCY (%)
**1**	73
**2**	66
**3**	84

Average RCY: 74 ± 7% (n = 3)

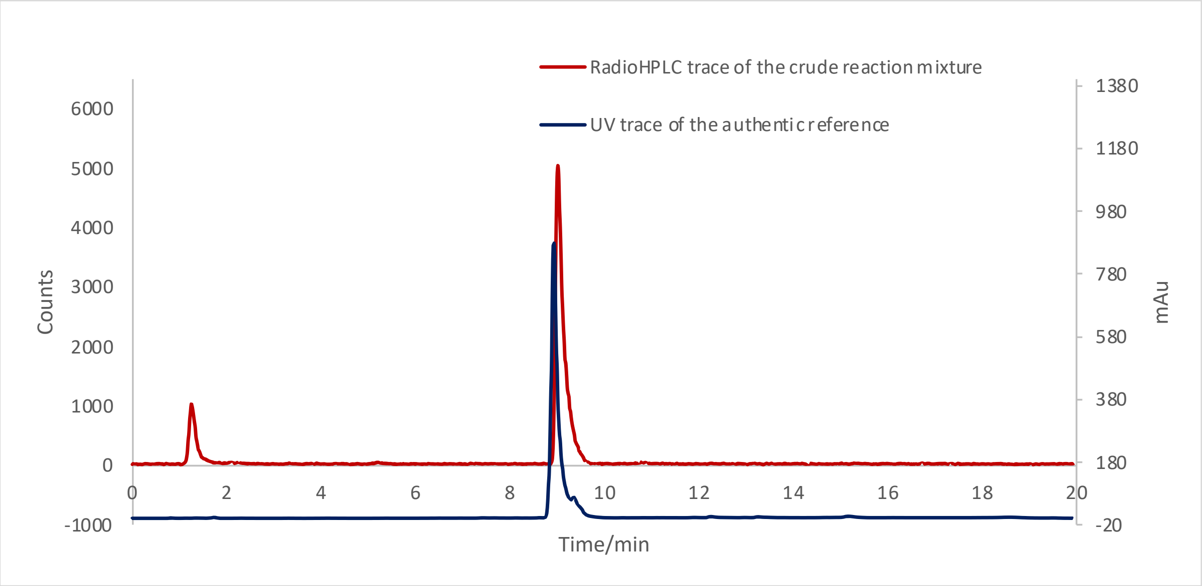

HPLC conditions C

[^{18}F]2-(1-Fluoroethyl)-6-methoxynaphthalene ([^{18}F]22)

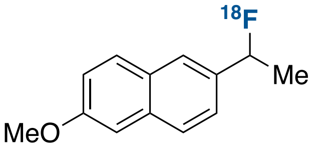

Entry	RCY (%)
1	66
2	61
3	59

Average RCY: 62 ± 3% (*n* = 3)

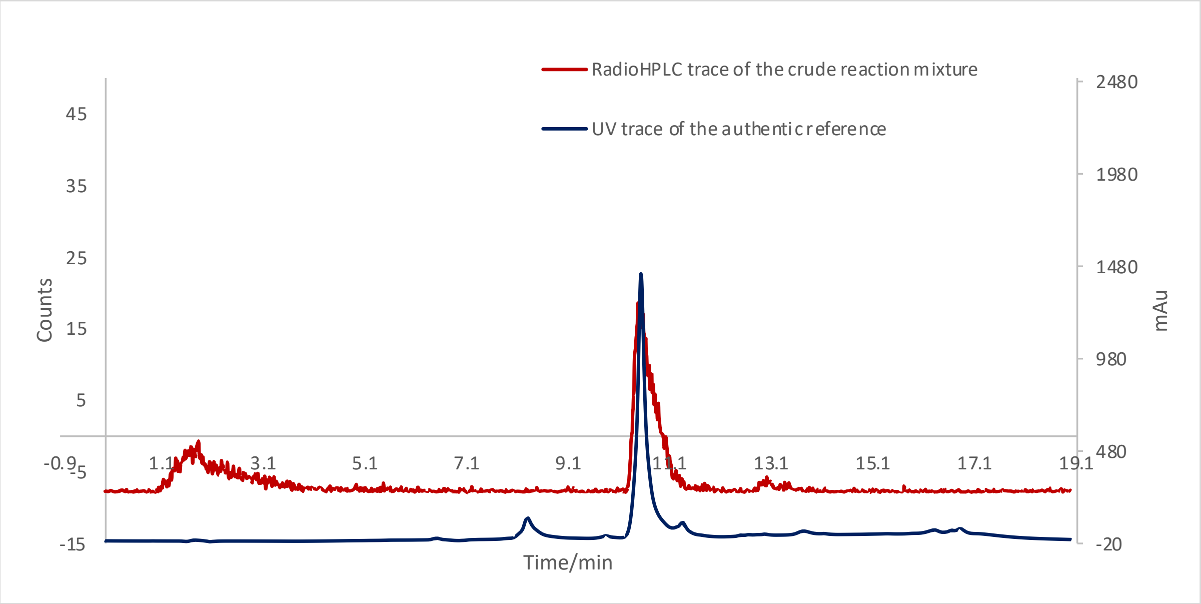

HPLC conditions A

[^{18}F](Fluoromethylene)dibenzene ([^{18}F]23)

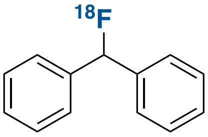

Entry	RCY (%)
1	86
2	84
3	88

Average RCY: 86 ± 2% (n = 3)

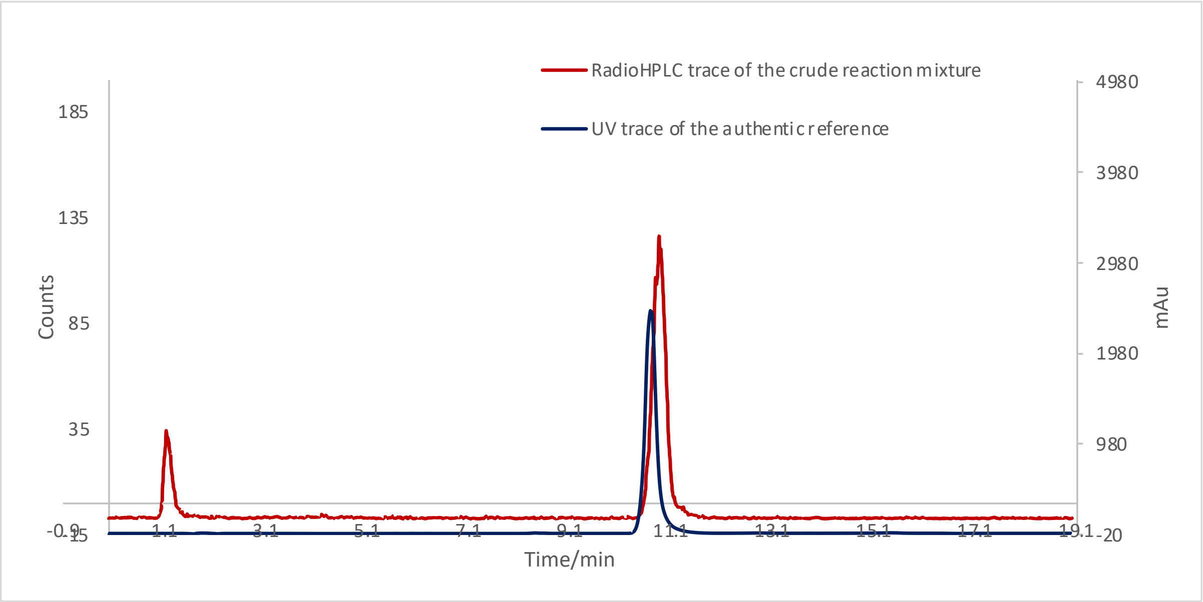

HPLC conditions A

[^{18}F]1-Fluoro-1,2,3,4-tetrahydronaphthalene ([^{18}F]24)

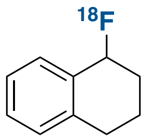

Entry	RCY (%)
**1**	59
**2**	84
**3**	57

Average RCY: 67 ± 12% (n = 3)

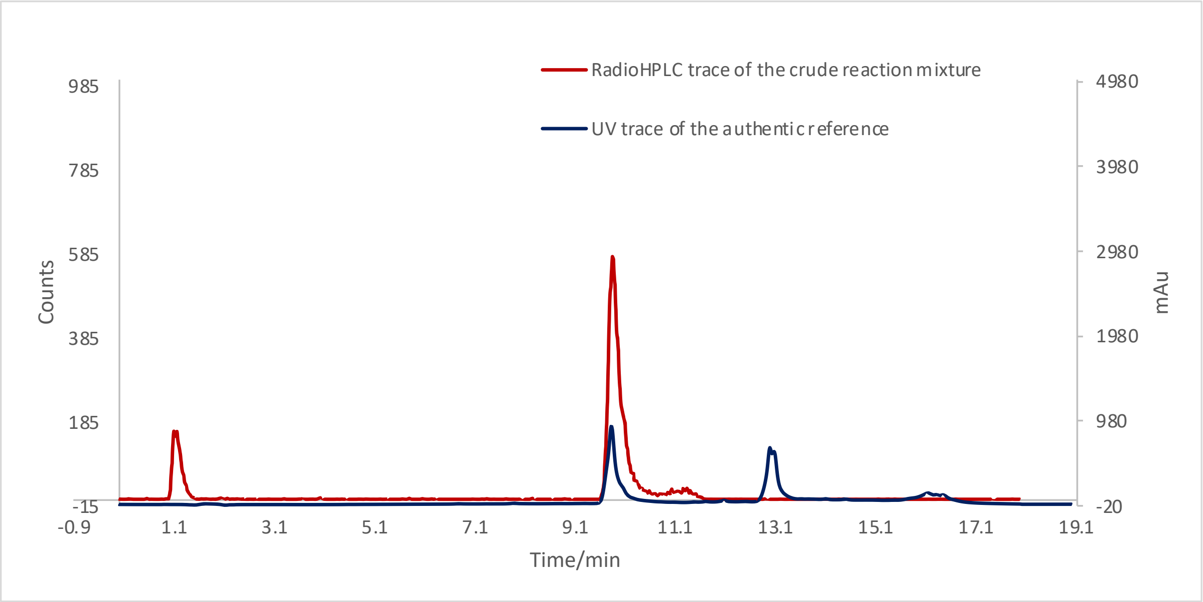

HPLC conditions A

[^{18}F]Methyl (1*R*,4a*S*,10a*R*)-9-fluoro-7-isopropyl-1,4a-dimethyl-1,2,3,4,4a,9,10,10a-octahydrophenanthrene-1-carboxylate ([^{18}F]25)

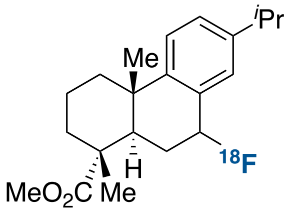

Entry	RCY (%)
1	60
2	63
3	54

Average RCY: 59 ± 4% (*n* = 3) (1.1:1 d.r.)

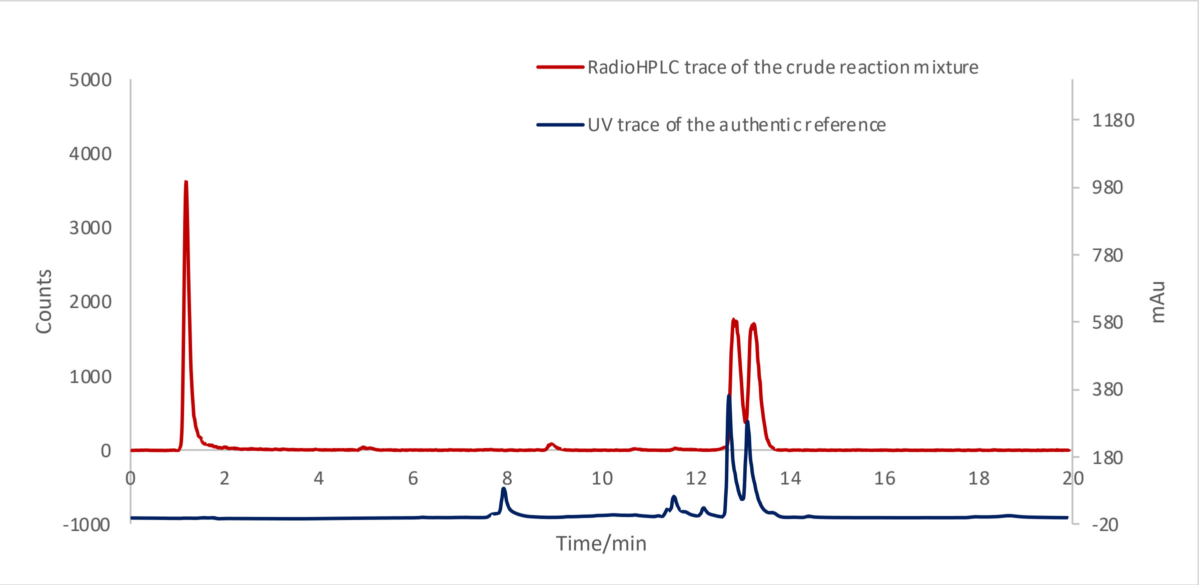

HPLC conditions C

[^{18}F]5-Fluoro-5-phenylpentan-1-ol ([^{18}F]26)

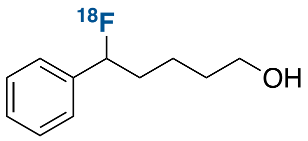

Entry	RCY (%)
1	22
2	12
3	24

Average RCY: 19 ± 5% (n = 3)

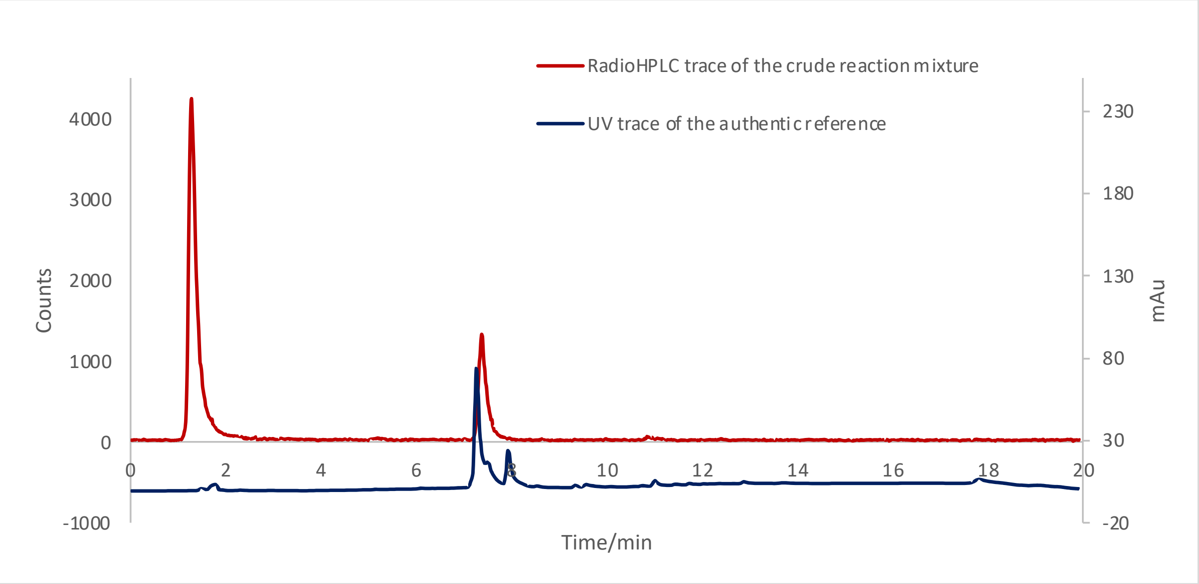

HPLC conditions C

[^{18}F](5-Bromo-1-fluoropentyl)benzene ([^{18}F]27)

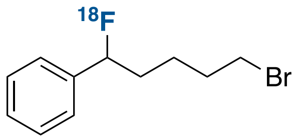

Entry	RCY (%)
**1**	72
**2**	82
**3**	69

Average RCY: 74 ± 6% (*n* = 3)

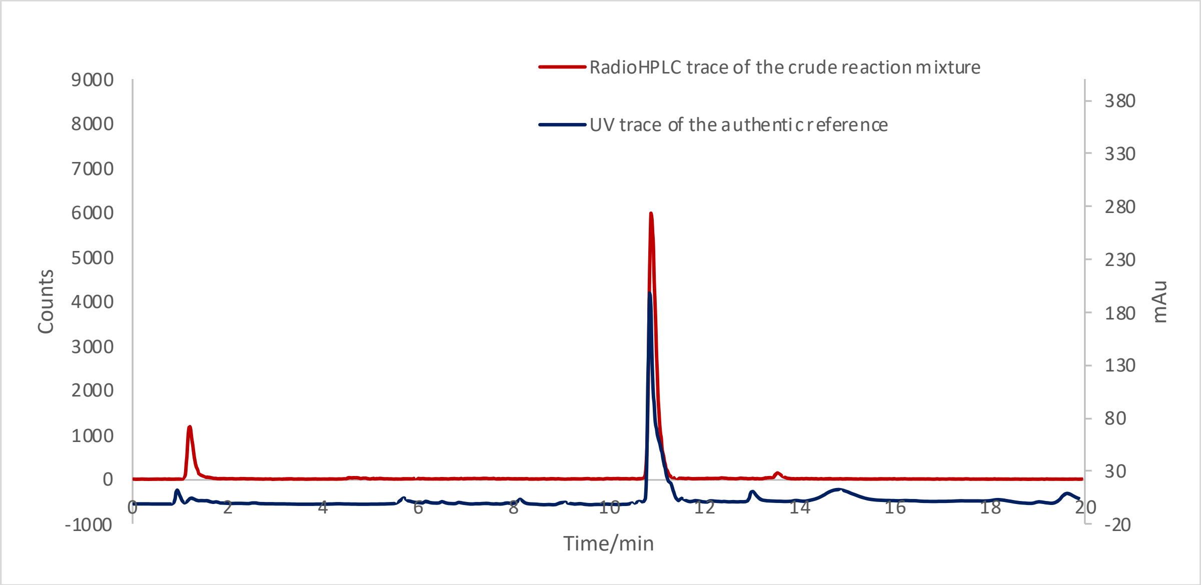

HPLC conditions C

[^{18}F](5-Fluoro-5-phenylpentyl 4-methylbenzenesulfonate ([^{18}F](28)

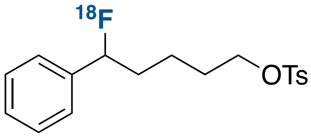

Entry	RCY (%)
1	50
2	38
3	77

Average RCY: 55 ± 16% (*n* = 3)

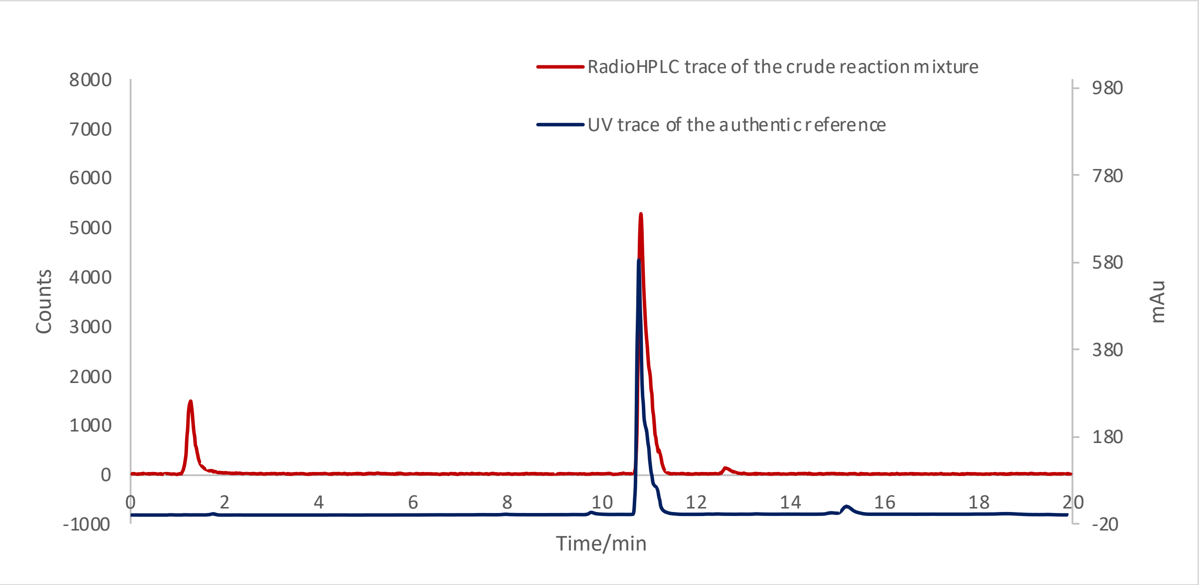

HPLC conditions C

[18F]2,3,5,6-Tetrafluorophenyl 5-fluoro-5-phenylpentanoate ([18F](29))

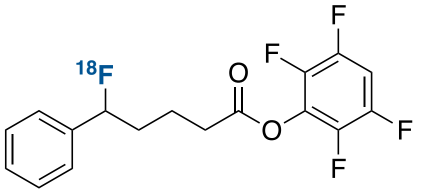

Entry	RCY (%)
1	33
2	31
3	30

Average RCY: 31 ± 1% (*n* = 3)

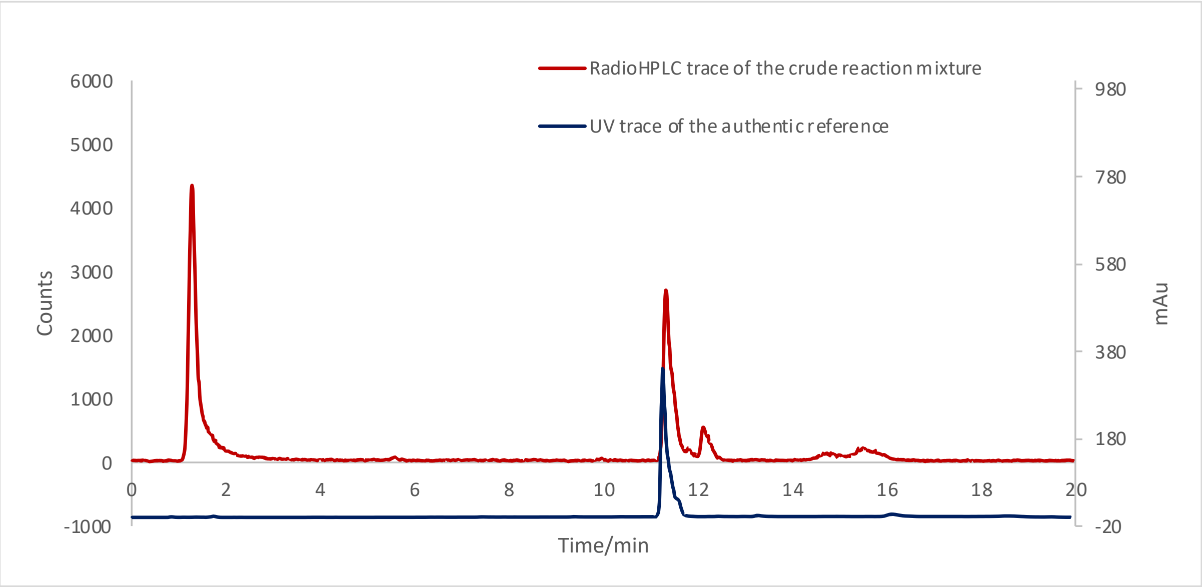

HPLC conditions C

[^{18}F]2-(1-Fluoroethyl)benzofuran ([^{18}F]30)

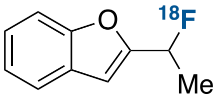

Entry	RCY (%)
1	68
2	79
3	73

Average RCY: 73 ± 5% (n = 3)

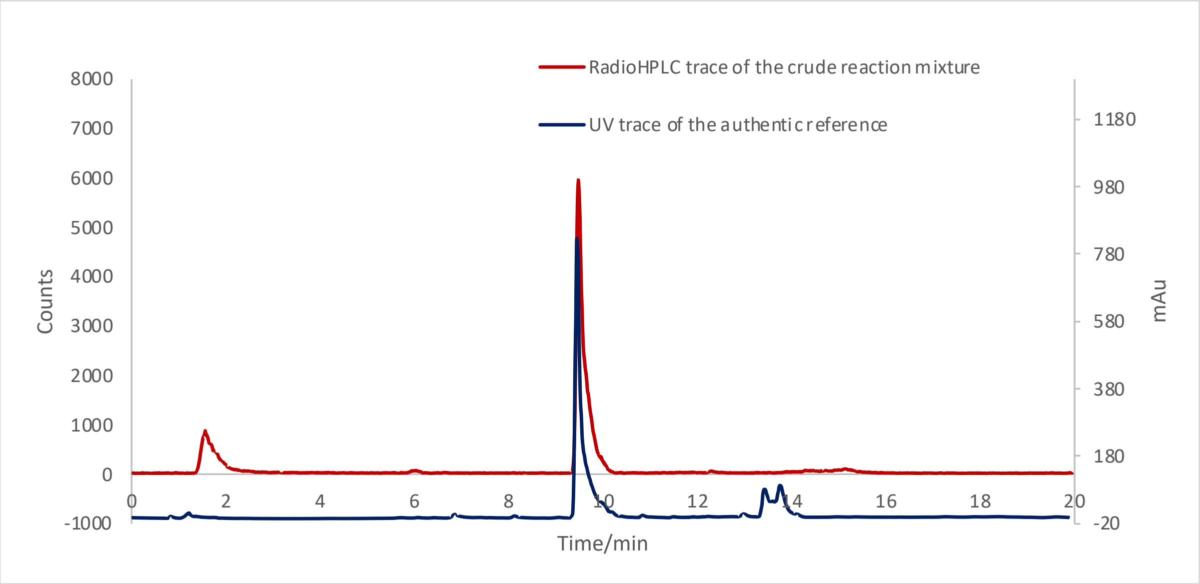

HPLC conditions C

[^{18}F]6-Chloro-2-(1-fluoroethyl)-9-methyl-9*H*-carbazole ([^{18}F]31)

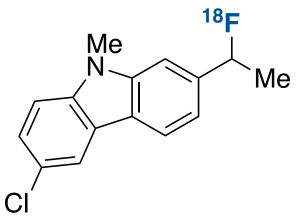

Entry	RCY (%)
1	70
2	49
3	33

Average RCY: 51 ± 15% (n = 3)

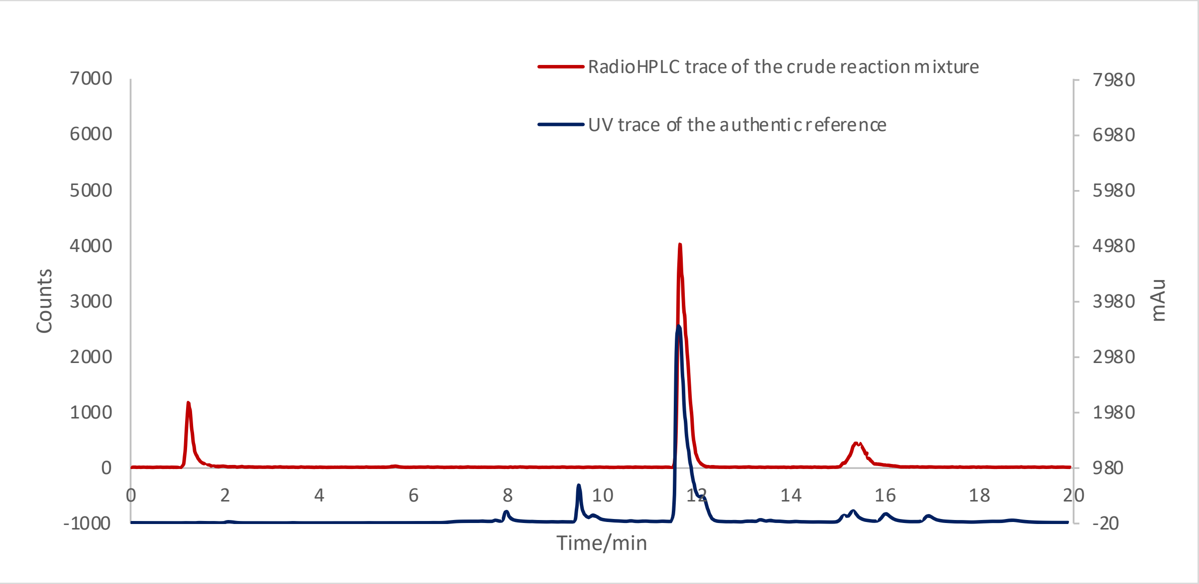

HPLC conditions C

[^{18}F]5-(1-fluoro-2-(4-(4-fluorophenyl)-1*H*-1,2,3-triazol-1-yl)ethyl)-4-methylthiazole ([^{18}F]32)

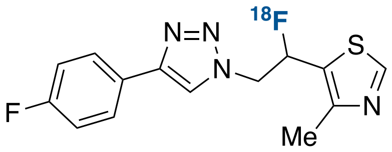

Entry	RCY (%)
**1**	32
**2**	26
**3**	23

Average RCY: 27 ± 4% (*n* = 3)

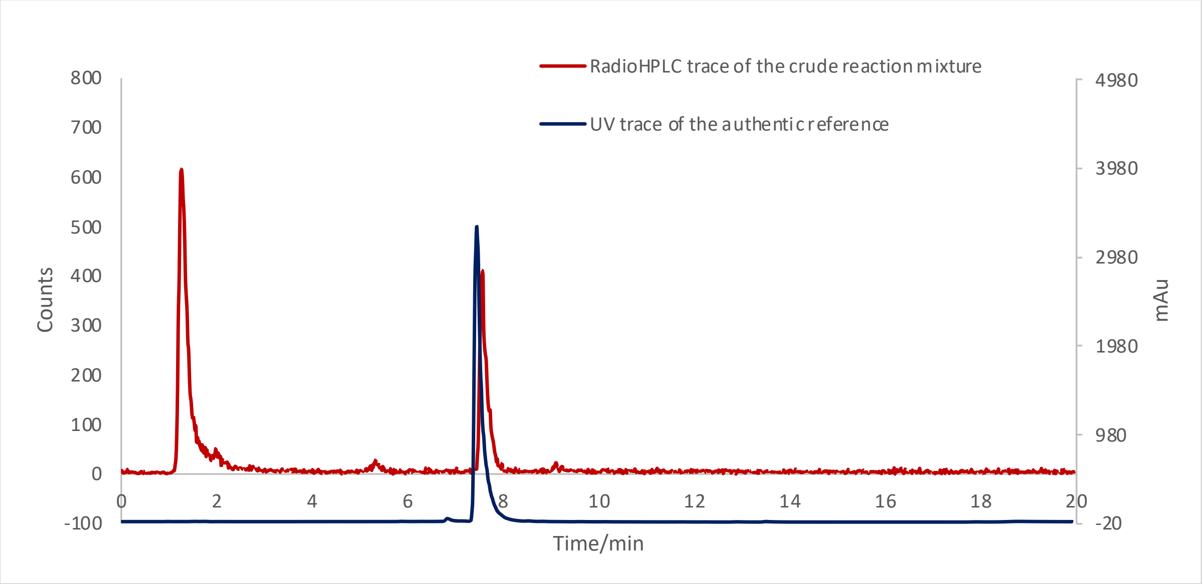

HPLC conditions C

[^{18}F](4-(Fluoromethyl)phenyl)(methyl)sulfane ([^{18}F]33)

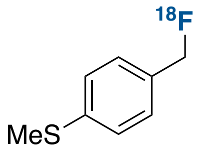

Entry	RCY (%)
1	78
2	51
3	45

Average RCY: 58 ± 14% (*n* = 3)

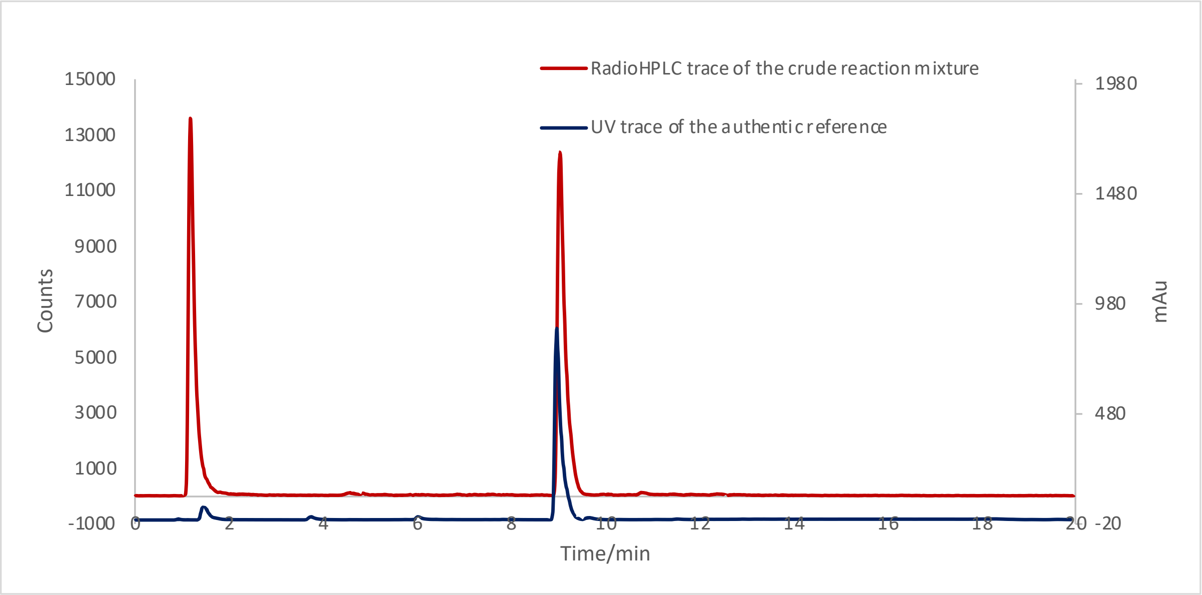

HPLC conditions C

[^{18}F]1-Azido-4-(fluoromethyl)benzene ([^{18}F](34)

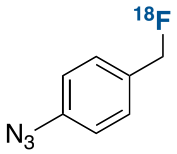

Entry	RCY (%)
**1**	16
**2**	30
**3**	20

Average RCY: 22 ± 6% (n = 3)

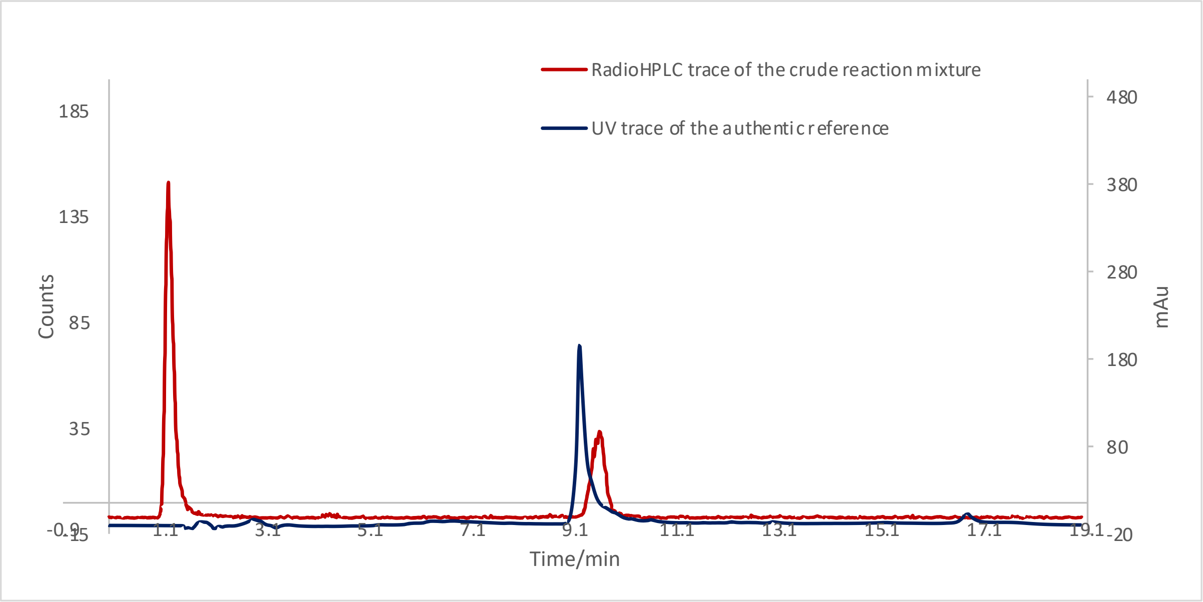

HPLC conditions A

[^{18}F]1-(Fluoromethyl)-4-methoxybenzene ([^{18}F]35)

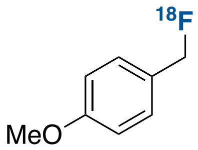

Entry	RCY (%)
1	82
2	68
3	71

Average RCY: 74 ± 6% (n = 3)

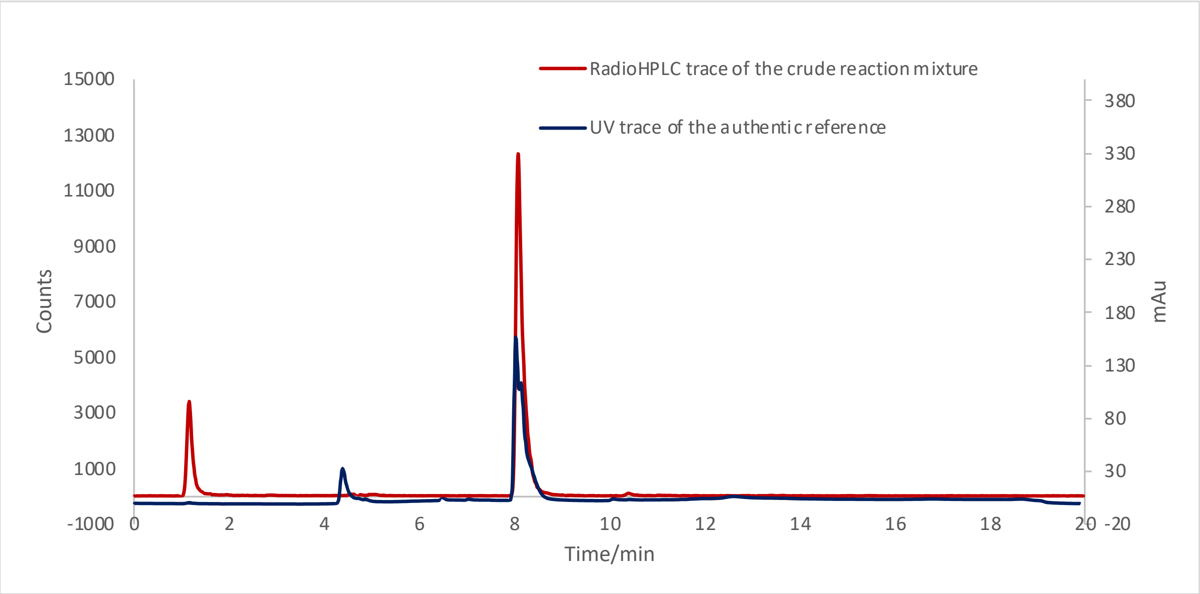

HPLC conditions C

[^{18}F](2-Fluoropropan-2-yl)benzene ([^{18}F]36)

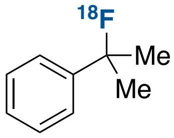

Entry	RCY (%)
**1**	81
**2**	77
**3**	80

Average RCY: 79 ± 2% (n = 3)

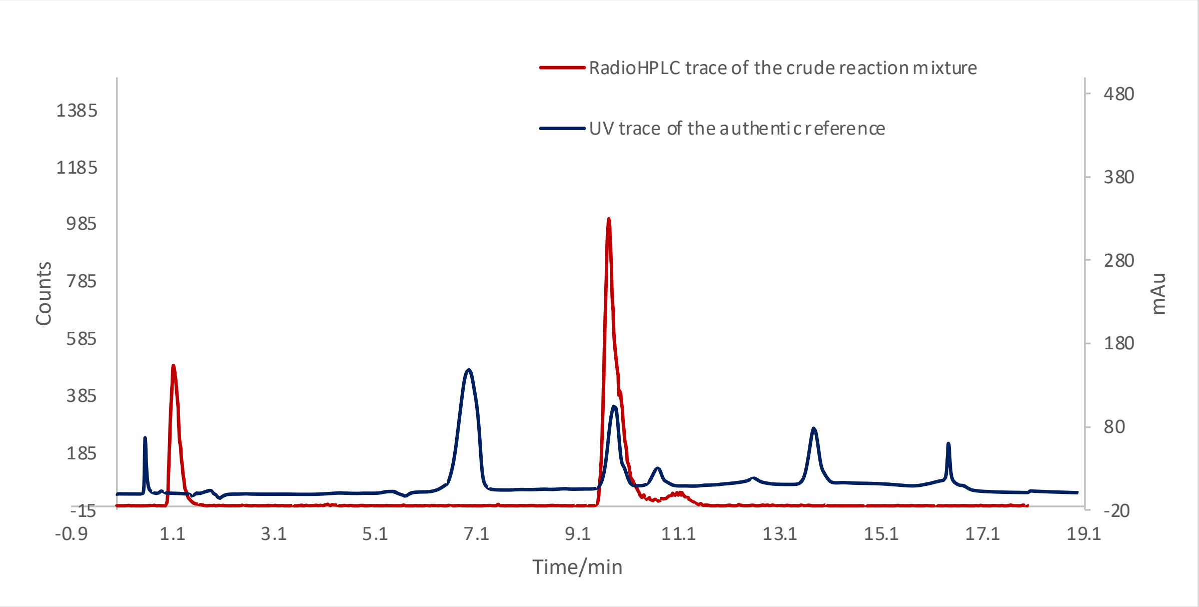

HPLC conditions A

Note: Authentic reference **36** was found to show signs of degradation during HPLC analysis.

[^{18}F](1-Fluorocyclohexyl)benzene ([^{18}F](37)

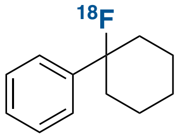

Entry	RCY (%)
**1**	74
**2**	79
**3**	83

Average RCY: 79 ± 4% (*n* = 2)

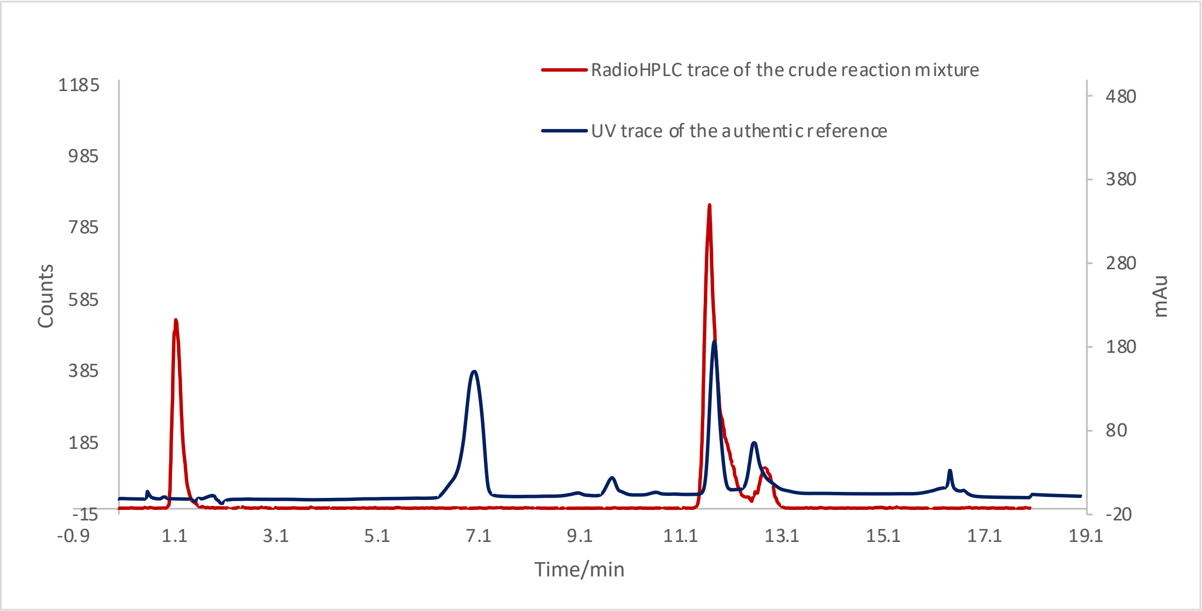

HPLC conditions A

***Note*:** Authentic reference **37** was found to show signs of degradation during HPLC analysis.

[^{18}F]2-((4-Fluoro-4-methylpentyl)oxy)-1,4-dimethylbenzene ([^{18}F](38))

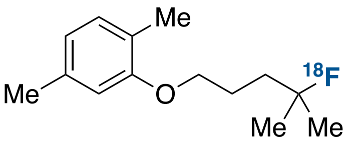

Entry	RCY (%)
1	23
2	30
3	14

Average RCY: 22 ± 7% (n = 3)

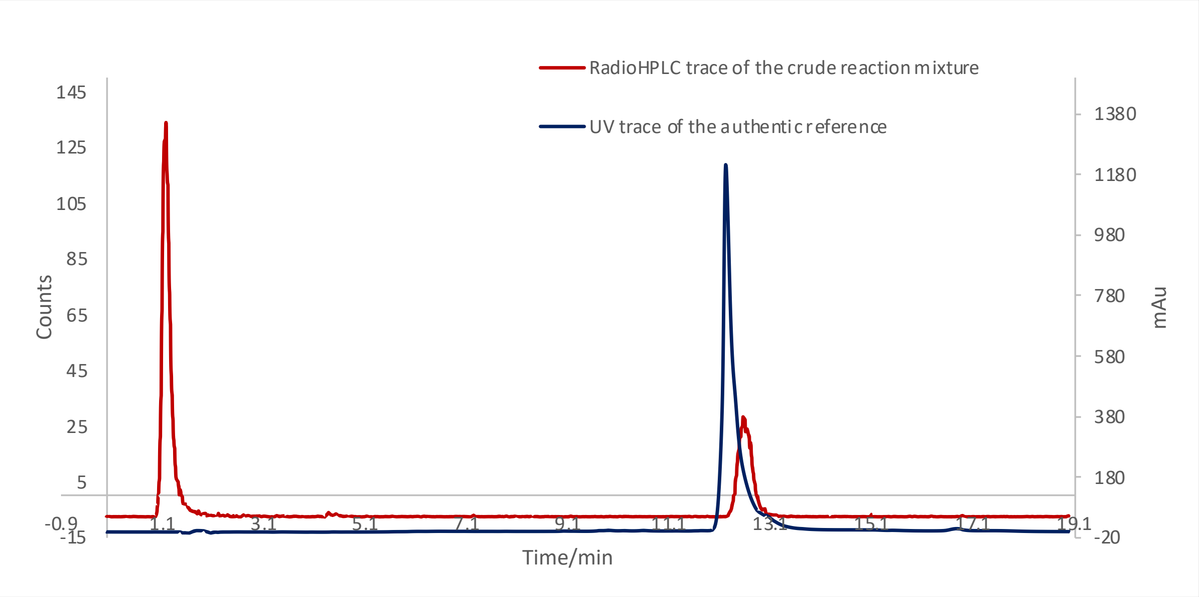

HPLC conditions A

[^{18}F]*tert*-Butyl ((1*r*,3*s*,5*R*,7*S*)-3-fluoroadamantan-1-yl)carbamate ([^{18}F](39)

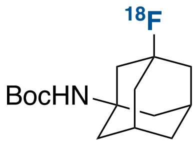

Entry	RCY (%)
1	7
2	9
3	3

Average RCY: 6 ± 3% (n = 3)

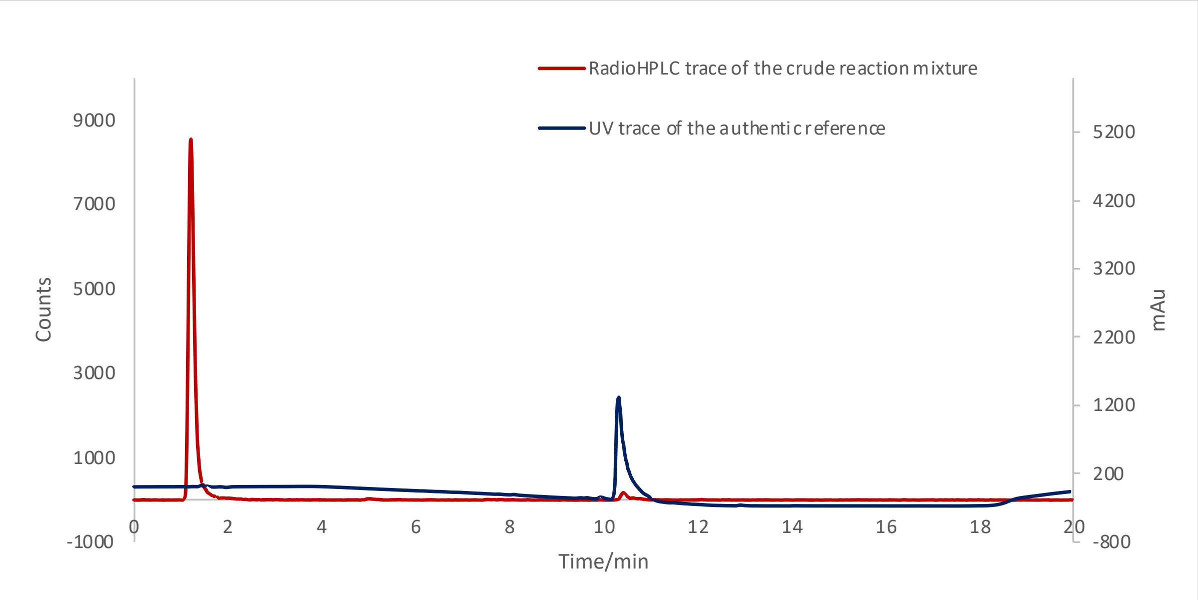

Enlarged HPLC trace:

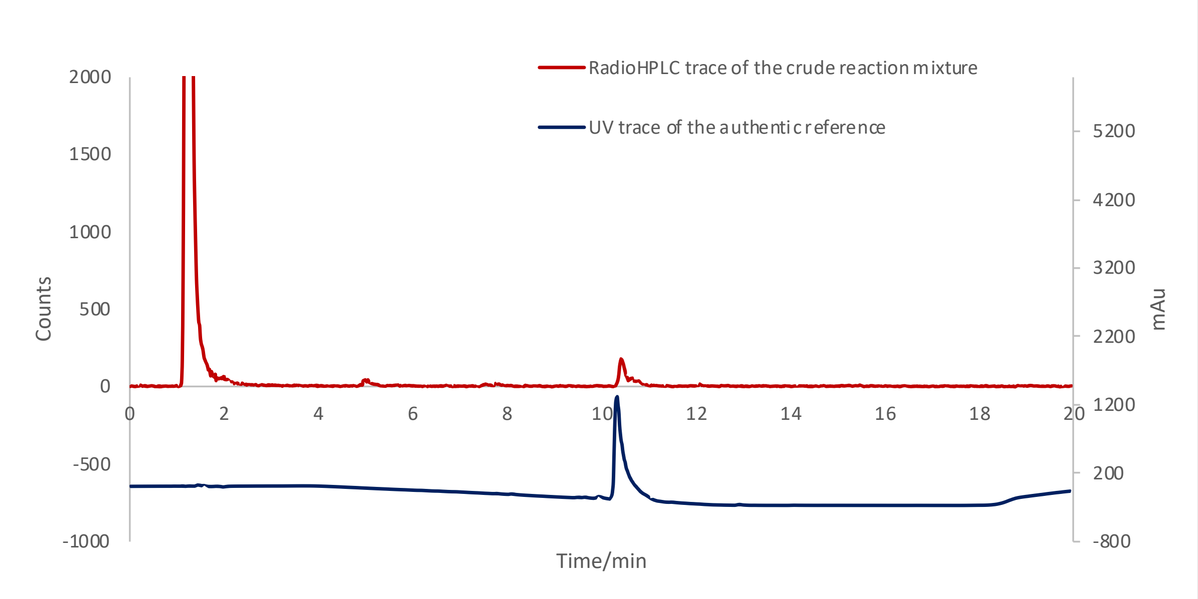

HPLC conditions C

Note: for this run, the wavelength was adjusted to 190 nm.

[^{18}F]2-Fluoro-1,4-dioxane ([^{18}F]40)

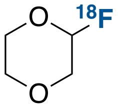

Entry	RCY (%)
1	79
2	80
3	78

Average RCY: 79 ± 1% (*n* = 3)

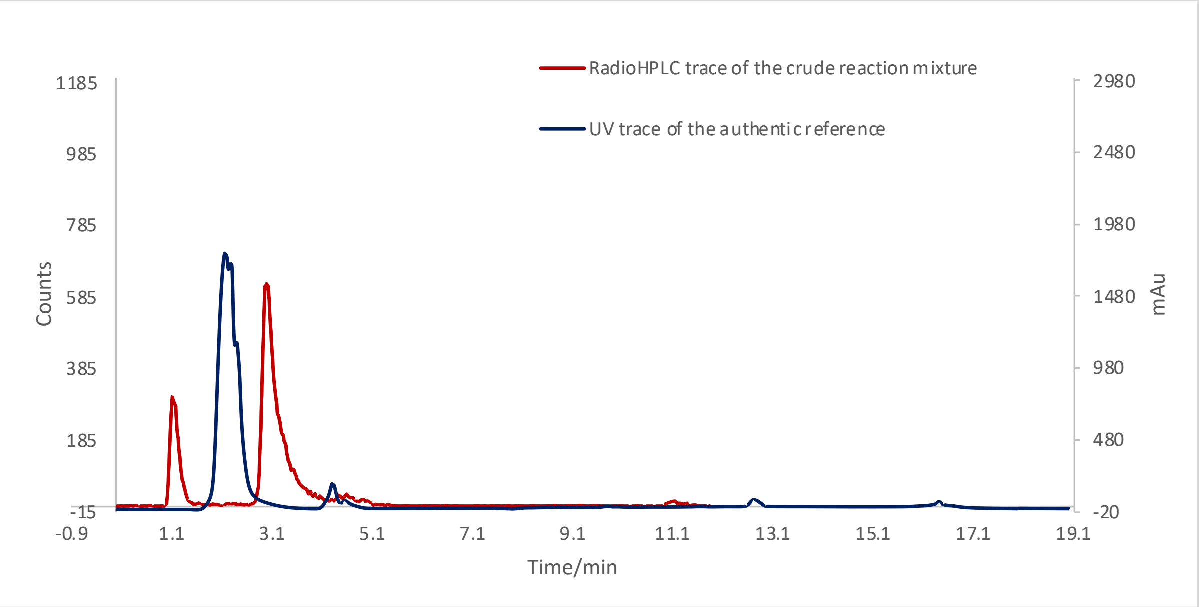

HPLC conditions A

Note: for this run, the wavelength was adjusted to 200 nm. No UV response could be detected for authentic reference **40** at various wavelengths, hence 1,4-dioxane was used.

.

[^{18}F]3-Fluoro-4-(4-(1-fluoroethoxy)phenoxy)benzonitrile ([^{18}F]41)

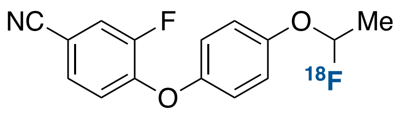

Entry	RCY (%)
1	72
2	78
3	71

Average RCY: 74 ± 3% (n = 3)

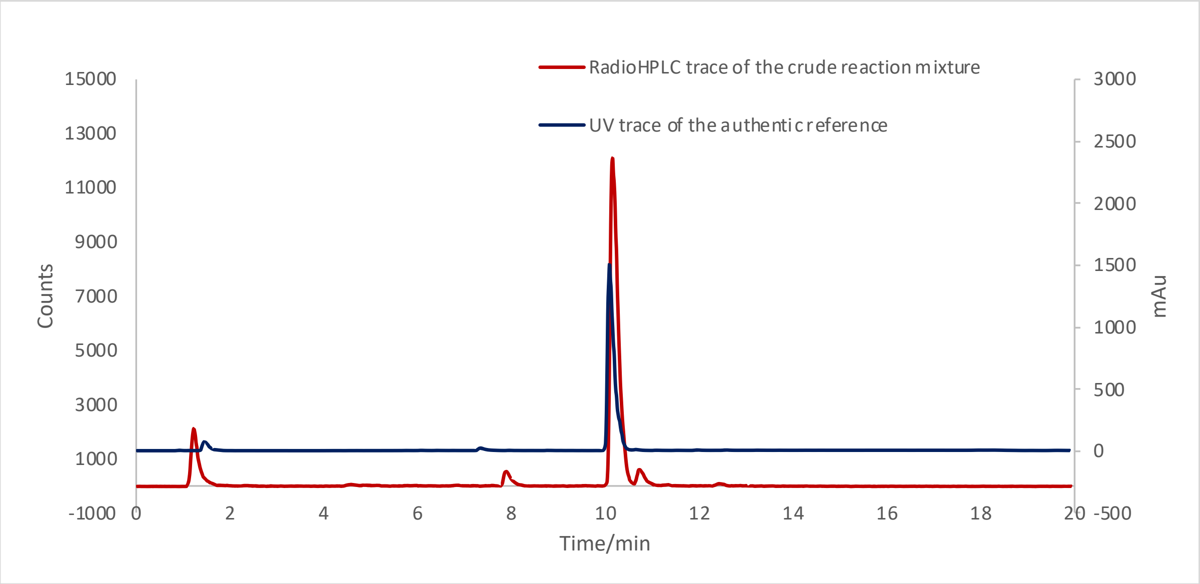

HPLC conditions C

[^{18}F](Fluoromethyl)(phenyl)sulfane ([^{18}F]42)

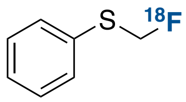

Entry	RCY (%)
1	33
2	21
3	18

Average RCY: 24 ± 7% (n = 3)

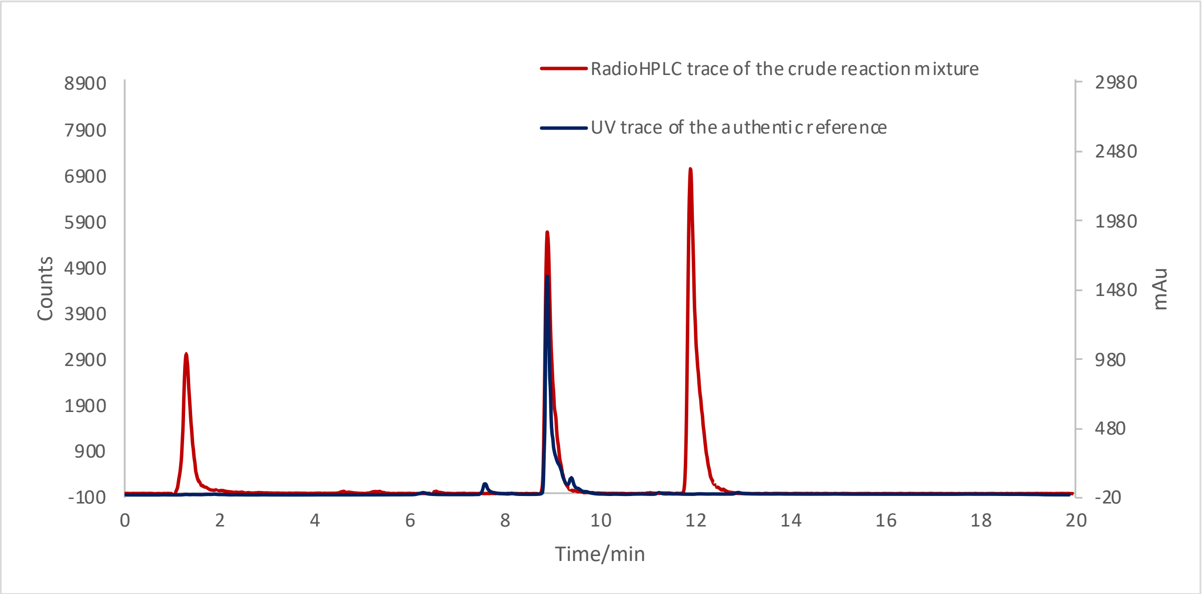

HPLC conditions C

[^{18}F]2-(1-Fluoro-2-phenylethyl)isoindoline-1,3-dione ([^{18}F]43)

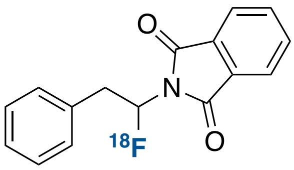

Entry	RCY (%)
1	80
2	83
3	80

Average RCY: 81 ± 1% (n = 3)

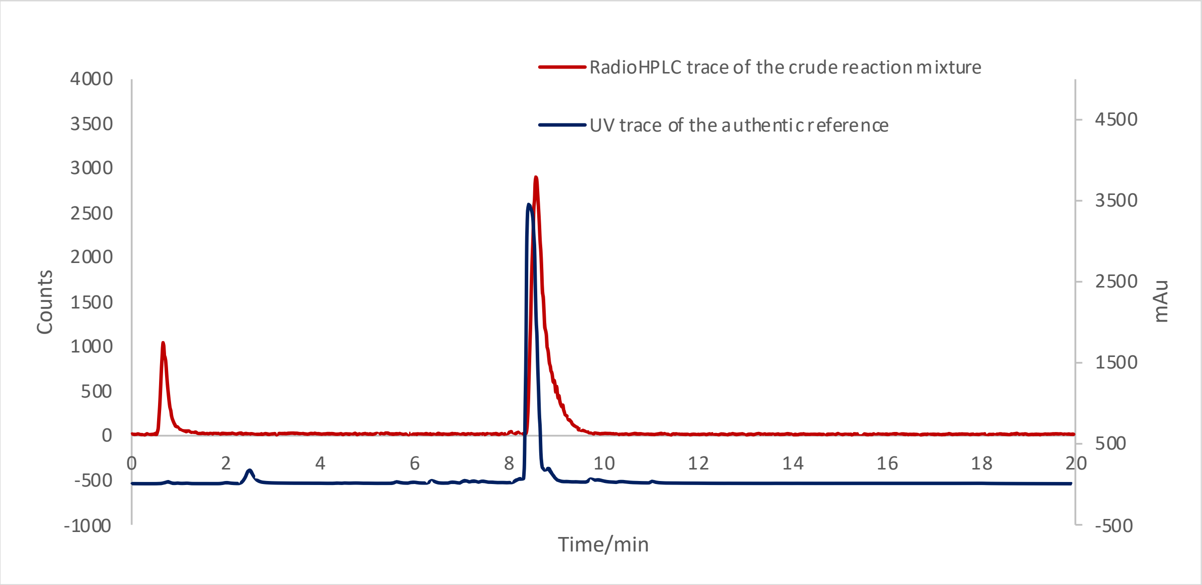

HPLC conditions C

[^{18}F]3-Fluorocyclohex-1-ene ([^{18}F]44)

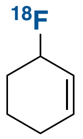

Entry	RCY (%)
1	49
2	64
3	57

Average RCY: 57 ± 6% (n = 3)

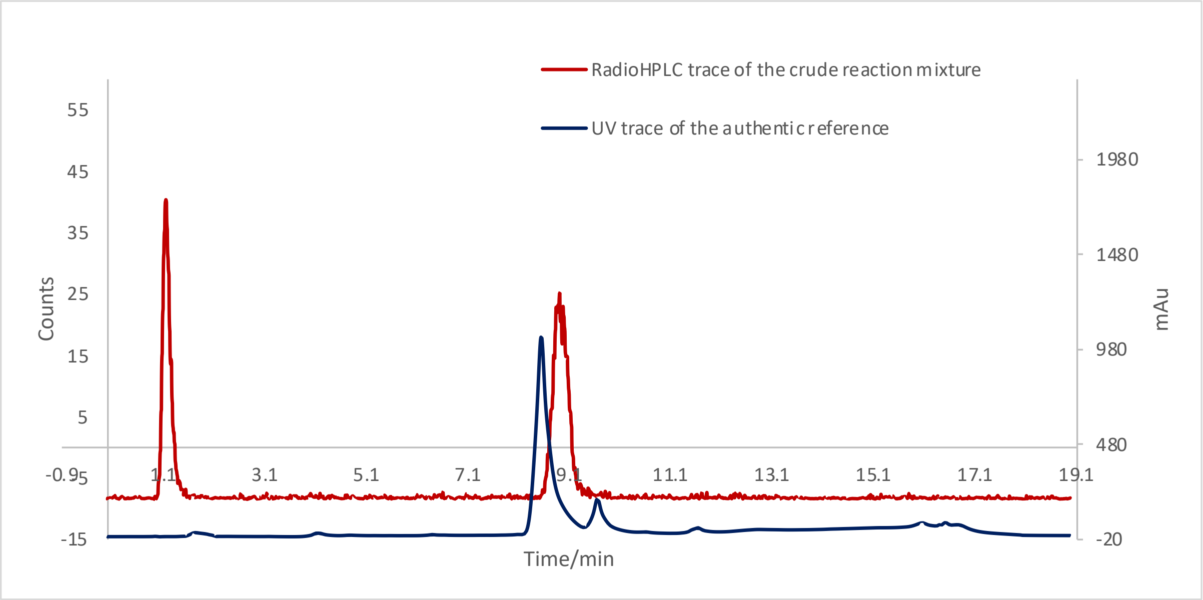

HPLC conditions A

Note: for this run, the wavelength was adjusted to 198 nm.

[^{18}F]2-Fluoro-1,2-bis(4-methoxyphenyl)ethan-1-one ([^{18}F]45)

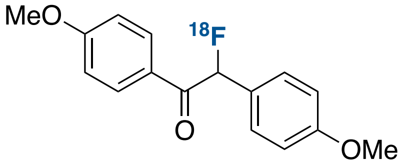

Entry	RCY (%)
**1**	49
**2**	51
**3**	71

Average RCY: 57 ± 10% (n = 3)

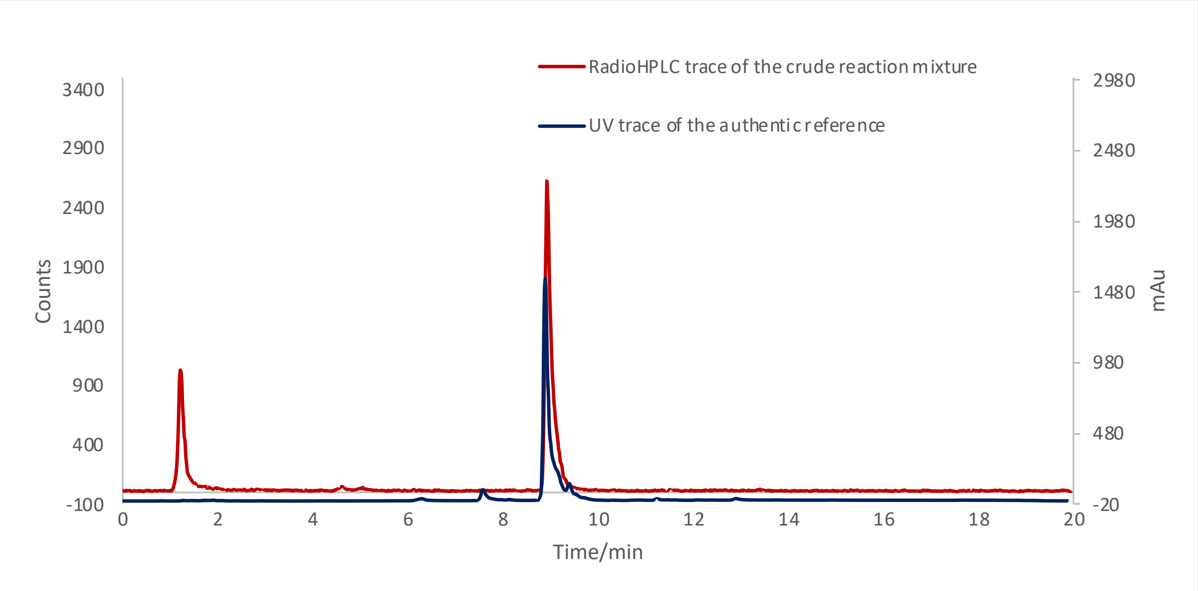

HPLC conditions C

[^{18}F]4-(4-(1-Fluoroethyl)phenethoxy)quinazoline ([^{18}F]46)

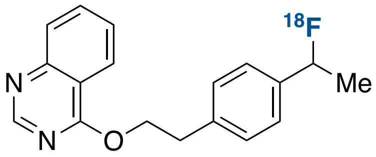

Entry	RCY (%)
**1**	76
**2**	62
**3**	64

Average RCY: 67 ± 6% (n = 3)

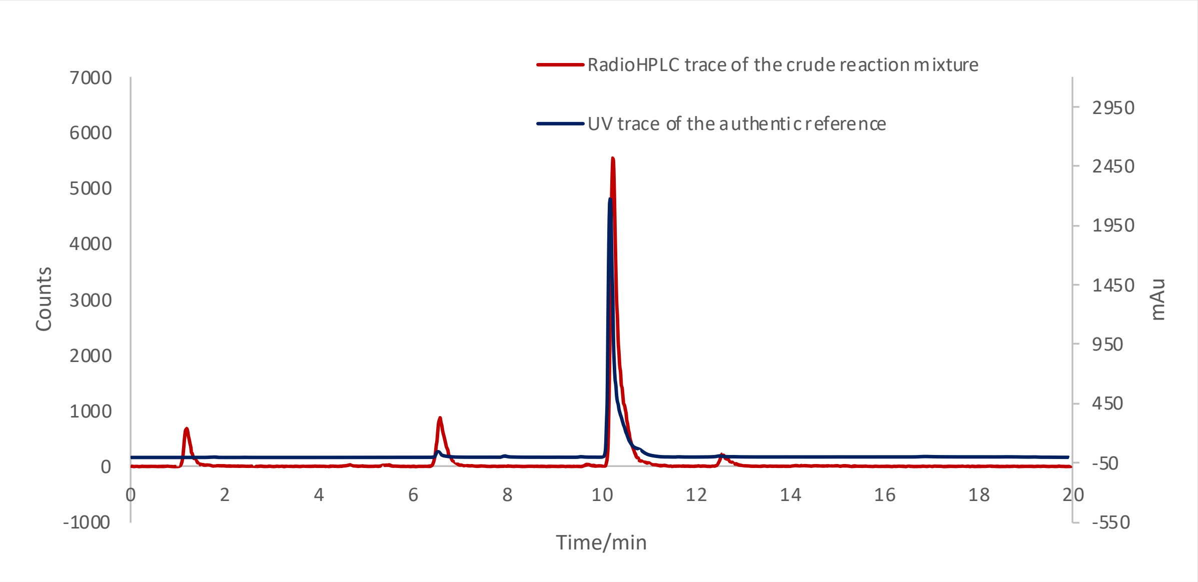

HPLC conditions C

Unsuccessful substrates

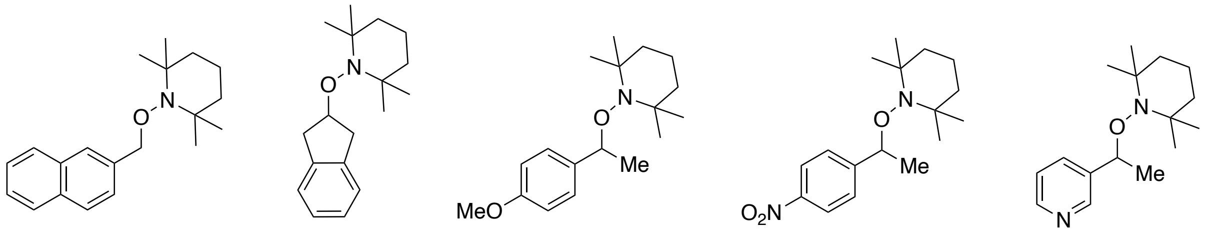

S47 **S48** **S49** **S50** **S51**

Figure s8: Unsuccessful substrates

Unactivated primary (**S47**) and secondary alkyl (**S48**), and strongly electron rich (**S49**) or poor substrates (**S50**) were found to be unsuccessful under the reaction conditions. 3-(1-((2,2,6,6-tetramethylpiperidin-1-yl)oxy)ethyl)pyridine (**S51**) was also unreactive towards radiofluorination.

References

1. Luo, J.; Jian Zhang, J. Donor–Acceptor Fluorophores for Visible-Light-Promoted Organic Synthesis: Photoredox/Ni Dual Catalytic C(sp^3)–C(sp^2) Cross-Coupling. *ACS Catal.* **2016**, *6*, 873–877.
2. Zhu, Q.; Gentry, E. C.; Knowles, R. R. Catalytic Carbocation Generation Enabled by the Mesolytic Cleavage of Alkoxyamine Radical Cations. *Angew. Chem. Int. Ed.* **2016**, *55*, 9969–9973.
3. Li, L.; Yu, Z.; Shen, Z. Copper-Catalyzed Aminoxylation of Different Types of Hydrocarbons with TEMPO: A Concise Route to *N*-Alkoxyamine Derivatives. *Adv. Synth. Catal.* **2015**, *357*, 3495–3500.
4. Schulz, G.; Kirschning, A. Metal free decarboxylative aminoxylation of carboxylic acids using a biphasic solvent system. *Org. Biomol. Chem.* **2021**, *19*, 273–278.
5. Zheng, C.; Wang, Y.; Xu, Y.; Chen, Z.; Chen, G.; Liang, S. H. Ru-Photoredox-Catalyzed Decarboxylative Oxygenation of Aliphatic Carboxylic Acids through *N*-(acyloxy)phthalimide. *Org. Lett.* **2018**, *20*, 4824−4827.
6. Kuang, C.; Zhou, X.; Xie, Q.; Ni, C.; Gu, Y.; Hu, J. Generation of Carbocations under Photoredox Catalysis: Electrophilic Aromatic Substitution with 1-Fluoroalkylbenzyl Bromides. *Org. Lett.* **2020**, *22*, 8670−8675.
7. Byun, Y.; Moon, J.; An, W.; Mishra, N. K.; Kim, H. S.; Ghosh, P.; Kim, I. S. Transition-Metal-Free Alkylation and Acylation of Benzoxazinones with 1,4-Dihydropyridines. *J. Org. Chem.* **2021**, *86*, 12247−12256.
8. Dao, J.; Benoit, D.; Hawker, C. J. A versatile and efficient synthesis of alkoxyamine LFR initiators via manganese based asymmetric epoxidation catalysts. *J. Polym. Sci. Part A: Polym. Chem.* **1998**, *36*, 2161–2167.
9. Marque, S.; Fischer, H.; Baier, E.; Studer, A. Factors Influencing the C−O Bond Homolysis of Alkoxyamines: Effects of H−Bonding and Polar Substituents. *J. Org. Chem.* **2001**, *66*, 1146–1156.
10. Stasiak, M.; Röben, C.; Rosenberger, N.; Schleth, F.; Studer, A.; Greiner, A.; Wendorff, J. H. Design of polymer nanofiber systems for the immobilization of homogeneous catalysts – Preparation and leaching studies. *Polymer* **2007**, *48*, 5208–5218.
11. Thompson, A. M.; Sutherland, H. S.; Palmer, B. D.; Kmentova, I.; Blaser, A.; Franzblau, S. G.; Wan, B.; Wang, Y.; Ma, Z.; Denny, W. A. Synthesis and Structure-Activity Relationships of Varied Ether Linker Analogues of the Antitubercular Drug (6*S*)-2-Nitro-6-{[4-(trifluoromethoxy)benzyl]oxy}-6,7-dihydro-5*H*-imidazo[2,1-*b*][1,3]oxazine (PA-824). *J. Med. Chem.* **2011**, *54*, 6563–6585.
12. Holz, J.; Pfeffer, C.; Zuo, H.; Beierlein, D.; Richter, G.; Klemm, E.; Peters, R. In Situ Generated Gold Nanoparticles on Active Carbon as Reusable Highly Efficient Catalysts for a C_{sp3}–Cs_{p3} Stille Coupling. *Angew. Chem. Int. Ed.* **2019**, *58*, 10330–10334.
13. Li, W.-D.; Wu, Y.; Li, S.-J.; Jiang, Y.-Q.; Li, Y.-L.; Lan, Y.; Xia, J.-B. Boryl Radical Activation of Benzylic C–OH Bond: Cross-Electrophile Coupling of Free Alcohols and CO_2 via Photoredox Catalysis. *J. Am. Chem. Soc.* **2022**, *144*, 8551−8559.
14. Billingsley, K. L.; Barder, T. E.; Buchwald, S. L. Palladium-Catalyzed Borylation of Aryl Chlorides: Scope, Applications, and Computational Studies. *Angew. Chem. Int. Ed.* **2007**, *46*, 5359–5363.
15. (a) Schoening, K.-U.; Fischer, W.; Hauck, S.; Dichtl, A.; Kuepfert, M. Synthetic Studies on *N*-Alkoxyamines: A Mild and Broadly Applicable Route Starting from Nitroxide Radicals and Aldehydes. *J. Org. Chem.* **2009**, *74*, 1567–1573. (b) Guo, L.,

Song, F.; Zhu, S.; Li, H.; Chu, L. *syn*-Selective alkylarylation of terminal alkynes via the combination of photoredox and nickel catalysis. *Nat. Commun*. **2018**, *9*, 4543.

16. Lu, Z.; Ju, M.; Wang, Y.; Meinhardt, J. M.; Martinez Alvarado, J. I.; Villemure, E.; Terrett, J. A.; Lin, S. Regioselective aliphatic C–H functionalization using frustrated radical pairs. *Nature* **2023**, *619*, 514–520.
17. Favia, A. D.; Habrant, D.; Scarpelli, R.; Migliore, M.; Albani, C.; Bertozzi, S. M.; Dionisi, M.; Tarozzo, G.; Piomelli, D.; Cavalli, A.; De Vivo; M. Identification and Characterization of Carprofen as a Multitarget Fatty Acid Amide Hydrolase/Cyclooxygenase Inhibitor. *J. Med. Chem*. **2012**, *55*, 8807−8826.
18. Rao, D. S.; Reddy, T. R.; Gurawa, A.; Kumar, M.; Kashyap, S. Photoswitchable Regiodivergent Azidation of Olefins with Sulfonium Iodate(I) Reagent. *Org. Lett.* **2019**, *21*, 9990−9994.
19. Czaplyski, W. L.; Na, C. G.; Alexanian, E. J. C−H Xanthylation: A Synthetic Platform for Alkane Functionalization. *J. Am. Chem. Soc*. **2016**, *138*, 13854−13857.
20. Belkheira, M.; Abed, D. E.; Pons, J.-M.; Bressy, C. Organocatalytic Synthesis of 1,2,3-Triazoles from Unactivated Ketones and Arylazides. *Chem. Eur. J.* **2011**, *17*, 12917–12921.
21. Martin-Montero, R.; Yatham, V. R.; Yin, H.; Davies, J.; Martin, R. Ni-catalyzed Reductive Deaminative Arylation at sp^3 Carbon Centers. *Org. Lett.* **2019**, *21*, 2947–2951.
22. Andrews, J. A.; Pantaine, L. R. E.; Palmer, C. F.; Poole, D. L.; Willis, M. C. Sulfinates from Amines: A Radical Approach to Alkyl Sulfonyl Derivatives via Donor–Acceptor Activation of Pyridinium Salts. *Org. Lett*. **2021**, *23*, 8488−8493.
23. Yasu, Y.; Koike, T.; Akita, M. Visible Light-Induced Selective Generation of Radicals from Organoborates by Photoredox Catalysis. *Adv. Synth. Catal.* **2012**, *354*, 3414–3420.
24. Nakajima, M.; Nagasawa, S.; Matsumoto, K.; Kuribara, T.; Muranaka, A.; Uchiyama, M.; Nemoto, T. A Direct $S_0 \rightarrow T_n$ Transition in the Photoreaction of Heavy-Atom-Containing Molecules. *Angew. Chem. Int. Ed.* **2020**, *59*, 6847–6852.
25. Liang, Y.; Zhang, X.; MacMillan, D. W. C. Decarboxylative sp^3 C–N coupling via dual copper and photoredox catalysis. *Nature* **2018**, *559*, 83–88.
26. Wei, D.; Li, X.; Shen, L.; Ding, Y.; Liang, K.; Xia, C. Phenolate anion-catalyzed direct activation of inert alkyl chlorides driven by visible light. *Org. Chem. Front*. **2021**, *8*, 6364–6370.
27. Webb, E. W.; Park, J. B.; Cole, E. L.; Donnelly, D. J.; Bonacorsi, S. J.; Ewing, W. R.; Doyle, A. G. Nucleophilic (Radio)Fluorination of Redox-Active Esters via Radical-Polar Crossover Enabled by Photoredox Catalysis. *J. Am. Chem. Soc*. **2020**, *142*, 9493–950.
28. Li, Y.; Miyazawa, K.; Koike, T.; Akita, M. Alkyl- and aryl-thioalkylation of olefins with organotrifluoroborates by photoredox catalysis. *Org. Chem. Front.* **2015**, *2*, 319–323.
29. Tan, Z.; Zhu, S.; Liu, Y.; Feng, X. Photoinduced Chemo-, Site- and Stereoselective α-$C(sp^3)$−H Functionalization of Sulfides. *Angew. Chem. Int. Ed.* **2022**, *61*, e2022033.
30. Ahn, K.-H.; Kim, Y. Oxidation of Enolate Anion by Hypervalent Iodine Compounds: Synthesis of α-Tempo Carbonyl Compound, A New Living Radical Polymerization Initiator. *Syn. Comm.* **1999**, *29*, 4361–4366.
31. Donthiri, R. R.; Samanta, S.; Adimurthy, S. Copper-catalyzed $C(sp^3)$–H functionalization of ketones with vinyl azides: synthesis of substituted-1*H*-pyrroles. *Org. Biomol. Chem.* **2015**, *13*, 10113–10116.

32. Jiang, H.; Xu, M.; Lu, W.; Tian, W.; Wan, W.; Chen, Y.; Deng, H.; Wud, S.; Hao, J. Direct *gem*-difluoromethylenation of sp^3-hybridized carbon center through copper-mediated radical/radical cross-coupling for the construction of a CH_2–CF_2 linkage. *Chem. Commun.* **2015**, 51, 15756–15759.
33. Ociepa, M.; Turkowska, J.; Gryko; D. Redox-Activated Amines in C(sp^3)–C(*sp*) and C(sp^3)–C(sp^2) Bond Formation Enabled by Metal-Free Photoredox Catalysis. *ACS Catal.* **2018**, *8*, 11362–11367.
34. Zhu, Z.; Liu, J.; Dong, S.; Chen, B.; Wang, Z.; Tang, R.; Li, Z. Copper-Catalyzed Cross-Coupling of Benzylic Bromides with Arylboronic Acids: Synthesis of Diarylalkanes and Preliminary Antifungal Evaluation Against Magnaporthe Grisea. *Asian J. Org. Chem.* **2020**, *9*, 631–636.
35. Liao, J.; Guan, W.; Boscoe, B. P.; Tucker, J. W.; Tomlin, J. W.; Garnsey, M. R.; Watson, M. P. Transforming Benzylic Amines into Diarylmethanes: Cross-Couplings of Benzylic Pyridinium Salts via C–N Bond Activation. *Org. Lett.* **2018**, *20*, 3030–3033.
36. Xia, J.-B.; Zhu, C.; Chen, C. Visible Light-Promoted Metal-Free C–H Activation: Diarylketone-Catalyzed Selective Benzylic Mono- and Difluorination. *J. Am. Chem. Soc.* **2013**, *135*, 17494–17500.
37. Hou, Z.-W.; Liu, D.-J.; Xiong, P.; Lai, X.-L.; Song, J.; Xu, H.-C. Site-Selective Electrochemical Benzylic C−H Amination. *Angew. Chem. Int. Ed.* **2021**, *60*, 2943–2947.
38. Bao, H.; Zhou, B.; Jin, H.; Liu, Y. Diboron-Assisted Copper-Catalyzed Z-Selective Semihydrogenation of Alkynes Using Ethanol as a Hydrogen Donor. *J. Org. Chem.* **2019**, *84*, 3579–3589.
39. Bresciani, S.; O'Hagan, D. Stereospecific benzylic dehydroxyfluorination reactions using Bio's TMS-amine additive approach with challenging substrates. *Tet. Lett.* **2010**, *51*, 5795–5797.
40. Sood, D. E.; Champion, S.; Dawson, D. M.; Chabbra, S.; Bode, B. E.; Sutherland, A.; Watson, A. J. B. Deoxyfluorination with CuF_2: Enabled by Using a Lewis Base Activating Group. *Angew. Chem. Int. Ed.* **2020**, *59*, 8460–8463.
41. Pieber, B.; Shalom, M.; Antonietti, M.; Seeberger, P. H.; Gilmore, K. Continuous Heterogeneous Photocatalysis in Serial Micro-Batch Reactors. *Angew. Chem. Int. Ed.* **2018**, *57*, 9976–9979.
42. Barrios, F. J.; Springer, B. C.; Colby, D. A. Control of Transient Aluminum–Aminals for Masking and Unmasking Reactive Carbonyl Groups. *Org. Lett.* **2013**, *15*, 3082–3085.
43. Yin, X.; Chen, B.; Qiu, F.; Wang, X.; Liao, Y.; Wang, M.; Lei, X.; Liao, J. Enantioselective Palladium-Catalyzed Hydrofluorination of Alkenylarenes. *ACS Catal.* **2020**, *10*, 1954–1960.
44. Mandal, A.; Jang, J.; Yang, B.; Kim, H.; Shin, K. Palladium-Catalyzed Electrooxidative Hydrofluorination of Aryl-Substituted Alkenes with a Nucleophilic Fluorine Source. *Org. Lett.* **2023**, *25*, 195−199.
45. Zhao, S.; Guo, Y.; Su, Z.; Cao, W.; Wu, C.; Chen, Q.-Y. A Series of Deoxyfluorination Reagents Featuring OCF_2 Functional Groups. *Org. Lett.* **2020**, *22*, 8634–8637.
46. Huang, X.; Liu, W.; Hooker, J. M.; Groves, J. T. Targeted Fluorination with the Fluoride Ion by Manganese-Catalyzed Decarboxylation. *Angew. Chem. Int. Ed.* **2015**, *54*, 5241–5245.
47. Leibler, I. N.-M.; Tekle-Smith, M. A.; Doyle, A. G. A general strategy for C(sp^3)–H functionalization with nucleophiles using methyl radical as a hydrogen atom abstractor. *Nat. Commun.* **2021**, *12*, 6950.

48. Vasilopoulos, A.; Golden, D. L.; Buss, J. A.; Stahl, S. S. Copper-Catalyzed C–H Fluorination/Functionalization Sequence Enabling Benzylic C–H Cross Coupling with Diverse Nucleophiles. *Org. Lett.* **2020**, *22*, 5753–5757.
49. Amaoka, Y.; Nagatomo, M.; Inoue, M. Metal-Free Fluorination of C(sp^3)–H Bonds Using a Catalytic *N*-Oxyl Radical. *Org. Lett.* **2013**, *15*, 2160–2163.
50. Kuboyama, T.; Nakahara, M.; Yoshino, M.; Cui, Y.; Sako, T.; Wada, Y.; Imanishi, T.; Obika, S.; Watanabe, Y.; Suzuki, M.; Doi, H. Stoichiometry-focused ^{18}F-labeling of alkyne-substituted oligodeoxynucleotides using azido([^{18}F]fluoromethyl)benzenes by Cu-catalyzed Huisgen reaction. *Bioorg. Med. Chem.* **2011**, *19*, 249–255.
51. Su, Y.-M.; Feng, G.-S.; Wang, Z.-Y.; Lan, Q.; Wang, X.-S. Nickel-Catalyzed Monofluoromethylation of Aryl Boronic Acids. *Angew. Chem. Int. Ed.* **2015**, *54*, 6003–6007.
52. Stangier, M.; Scheremetjew, A.; Ackermann, L. Chemo- and Site-Selective Electro-Oxidative Alkane Fluorination by C(sp^3)−H Cleavage. *Chem. Eur. J.* **2022**, *28*, e202201654.
53. Kee, C. W.; Chin, K. F.; Wong, M. W.; Tan, C.-H. Selective fluorination of alkyl C–H bonds via Photocatalysis. *Chem. Commun.* **2014**, *50*, 8211–8214.
54. Bower, J. K.; Cypcar, A. D.; Henriquez, B.; Stieber, S. C. E.; Zhang, S. C(sp^3)–H Fluorination with a Copper(II)/(III) Redox Couple. *J. Am. Chem. Soc.* **2020**, *142*, 8514–8521.
55. Liu, Y.; Lu, L.; Shen, Q. Monofluoromethyl-Substituted Sulfonium Ylides: Electrophilic Monofluoromethylating Reagents with Broad Substrate Scopes. *Angew. Chem. Int. Ed.* **2017**, *56*, 9930–9934.
56. Ventre, S.; Petronijevic, F. R.; MacMillan, D. W. C. Decarboxylative Fluorination of Aliphatic Carboxylic Acids via Photoredox Catalysis. *J. Am. Chem. Soc.* **2015**, *137*, 5654–5657.
57. Zhang, Y.; Fitzpatrick, N. A.; Das, M.; Bedre, I. P.; Yayla, H. G.; Lall, M. S.; Musacchio, P. Z. A photoredox-catalyzed approach for formal hydride abstraction to enable Csp^3–H functionalization with nucleophilic partners (F, C, O, N, and Br/Cl). *Chem Catalysis* **2022**, *2*, 292–308.
58. Urquhart, L. *Nat. Rev. Drug Discov.* **2023**, *22*, 260.
59. Constantin, T.; Zanini, M.; Regni, A.; Shikh, N. S.; Julia, F.; Leonori, D. Aminoalkyl radicals as halogen-atom transfer agents for activation of alkyl and aryl halides. *Science* **2020**, *367*, 1021–1026.

NMR spectra

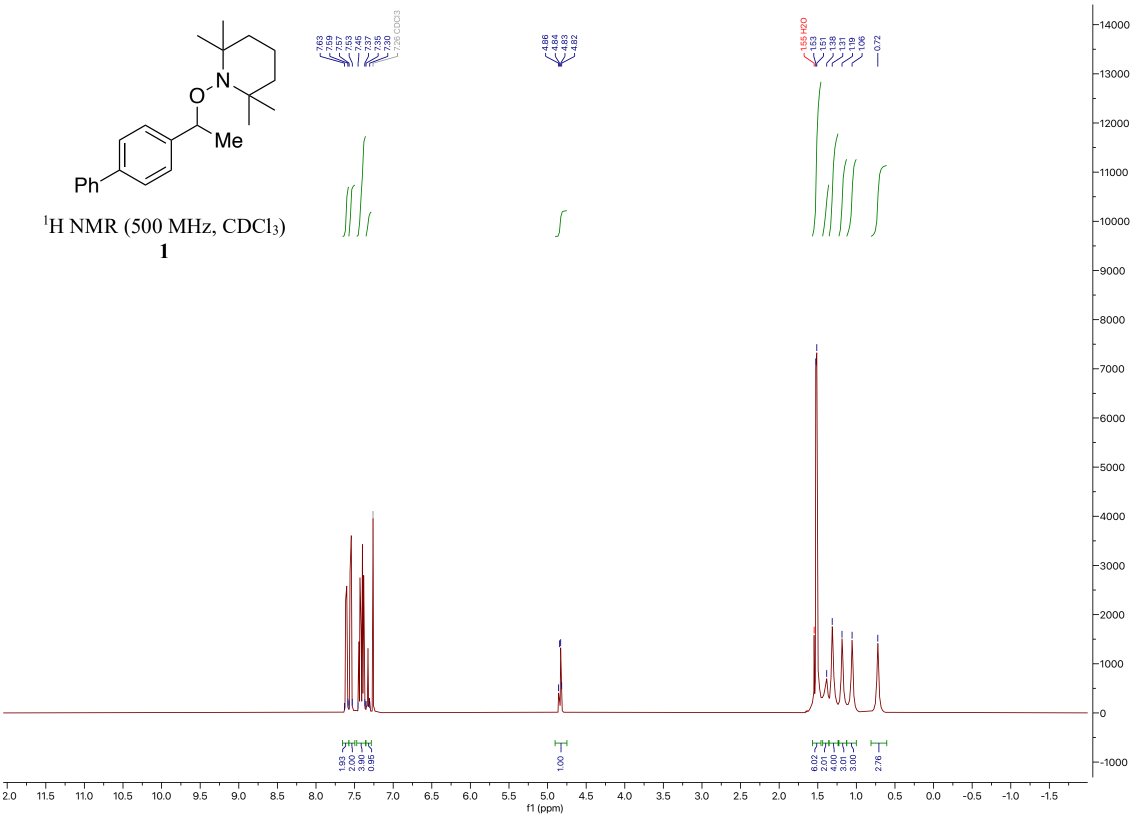

Ph
Me
O
N
^{1}H NMR (500 MHz, CDCl$_3$)
1
f1 (ppm)

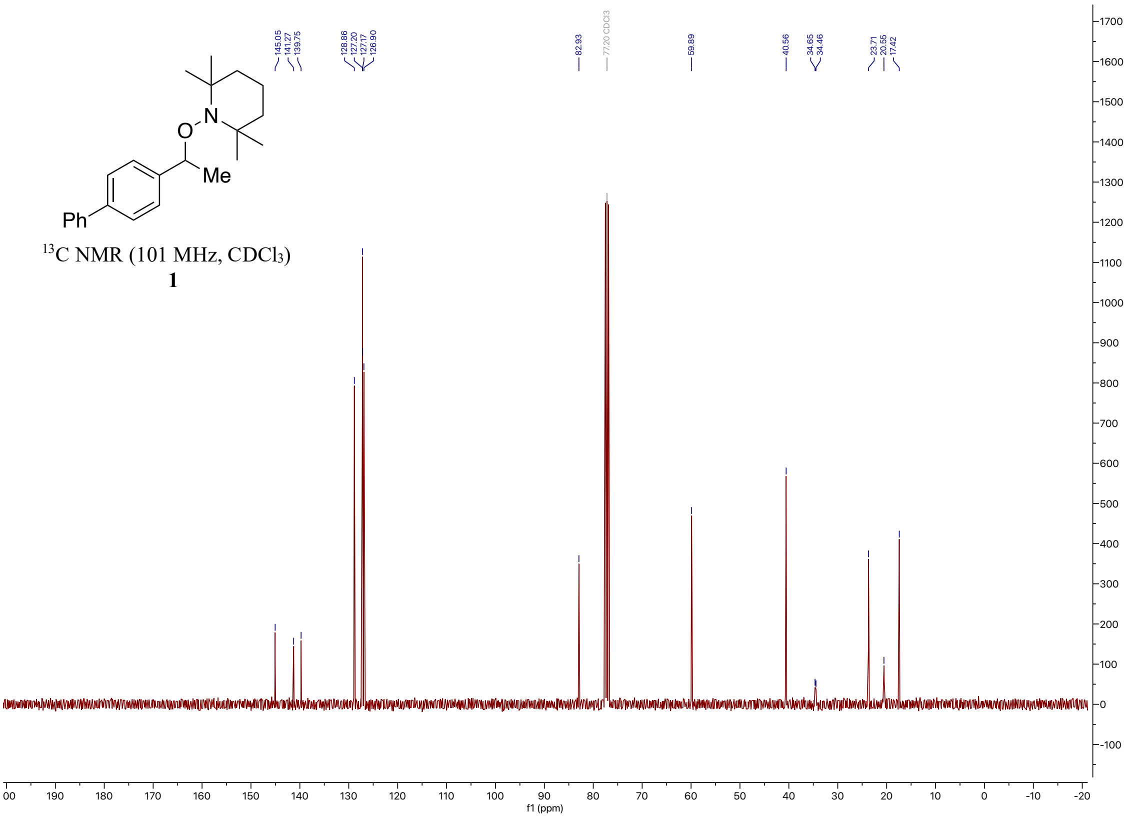

Ph
Me
O
N
^{13}C NMR (101 MHz, CDCl$_3$)
1
f1 (ppm)

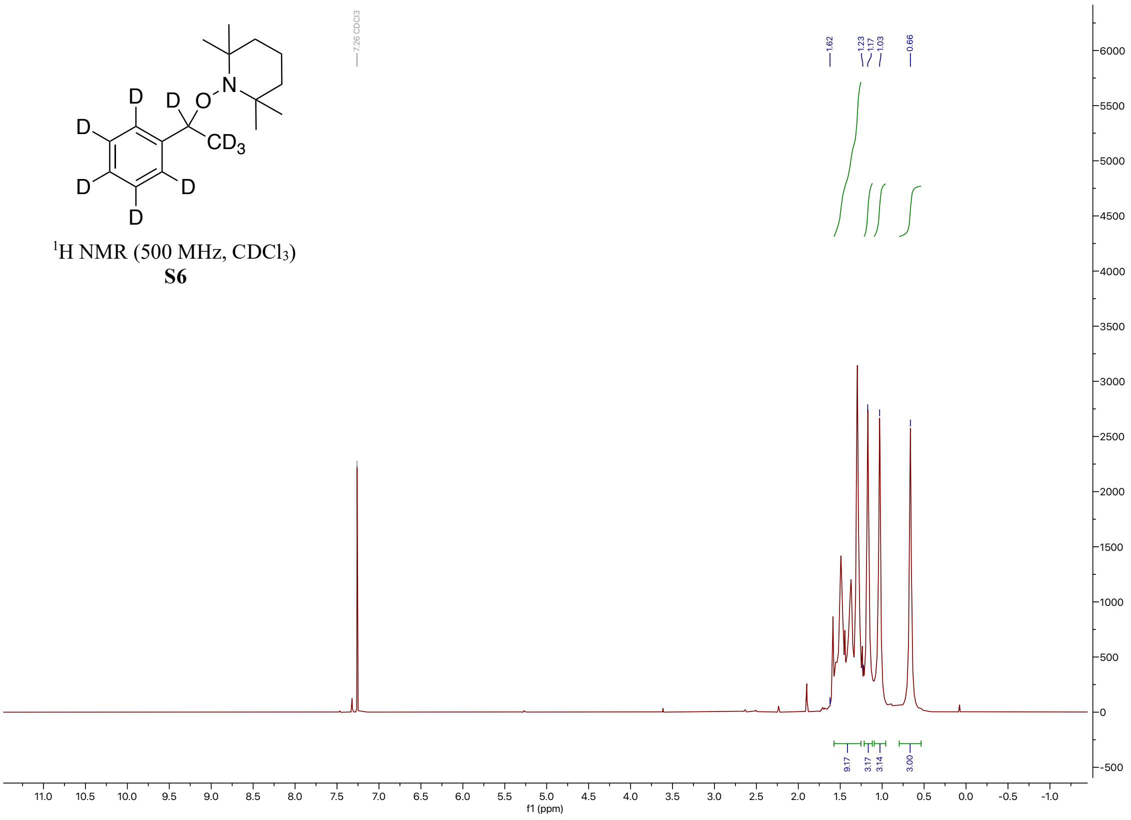

^{1}H NMR (500 MHz, $CDCl_3$)
S6

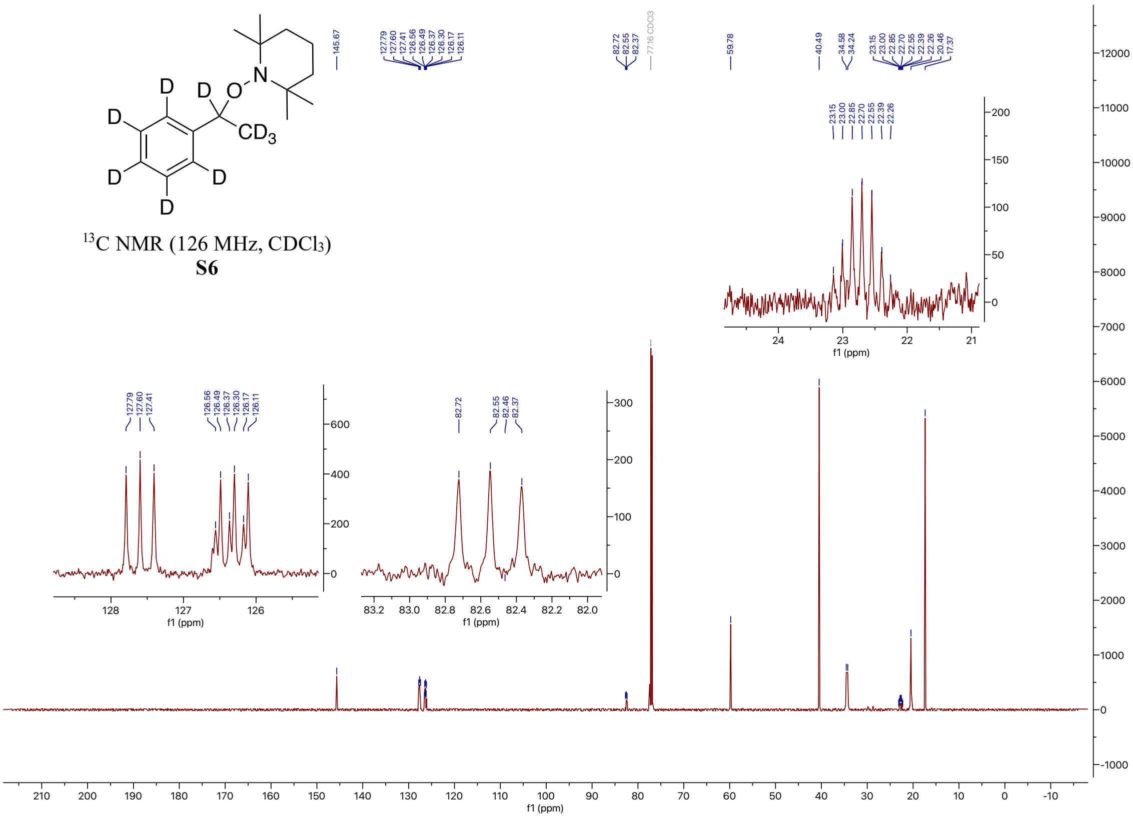

^{13}C NMR (126 MHz, $CDCl_3$)
S6

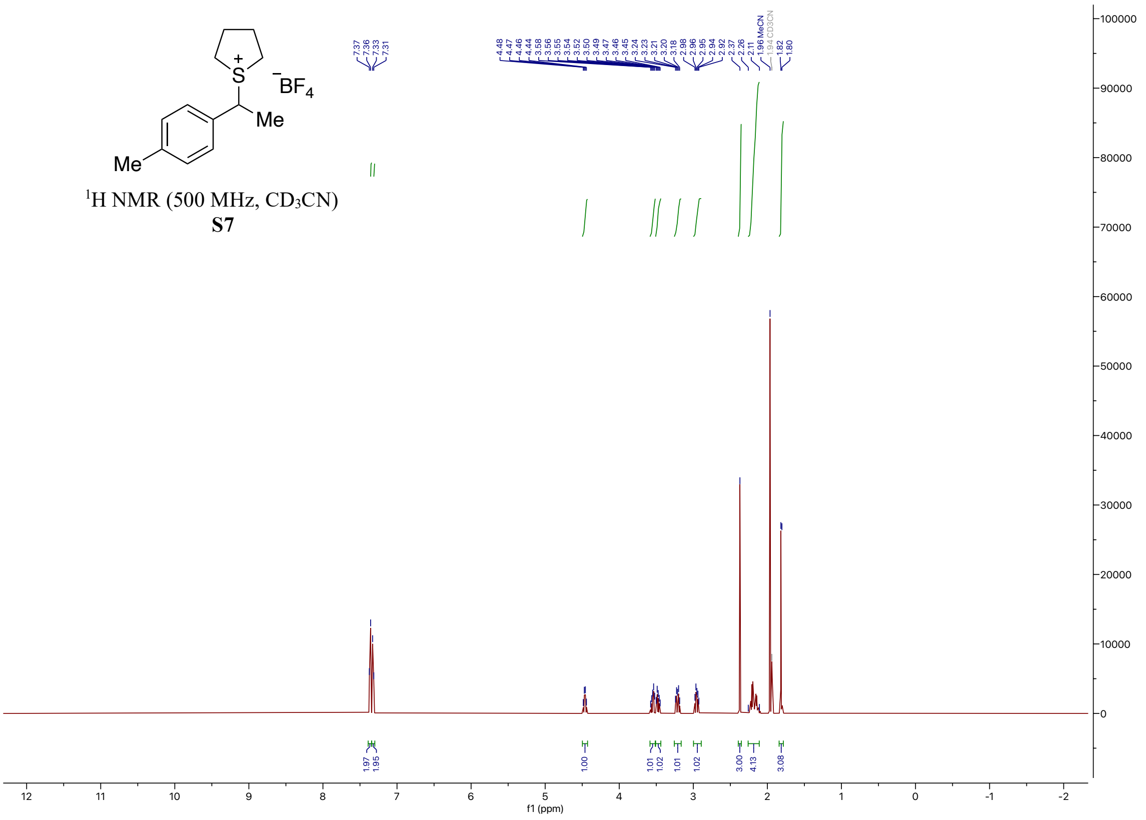

^{1}H NMR (500 MHz, CD_3CN)
S7

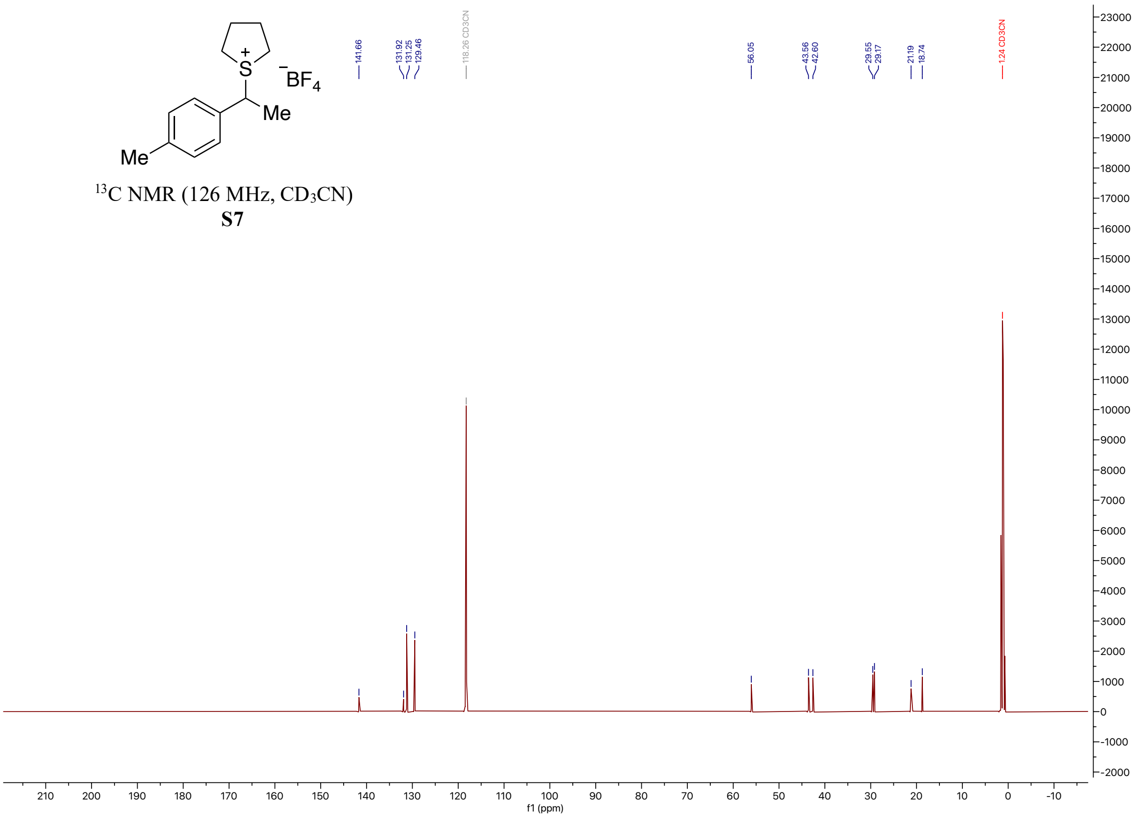

^{13}C NMR (126 MHz, CD_3CN)
S7

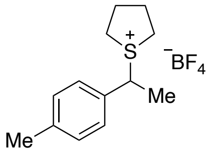

^{19}F NMR (471 MHz, CD_3CN)

S7

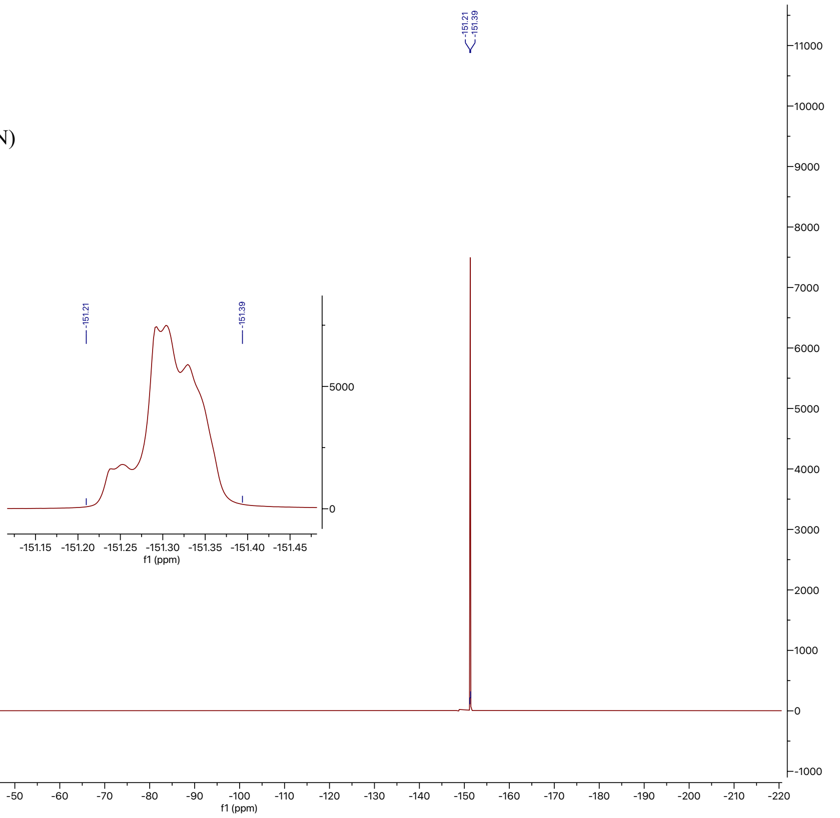

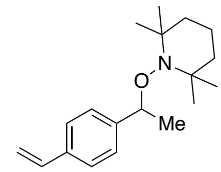

^{1}H NMR (400 MHz, $CDCl_3$)

S9

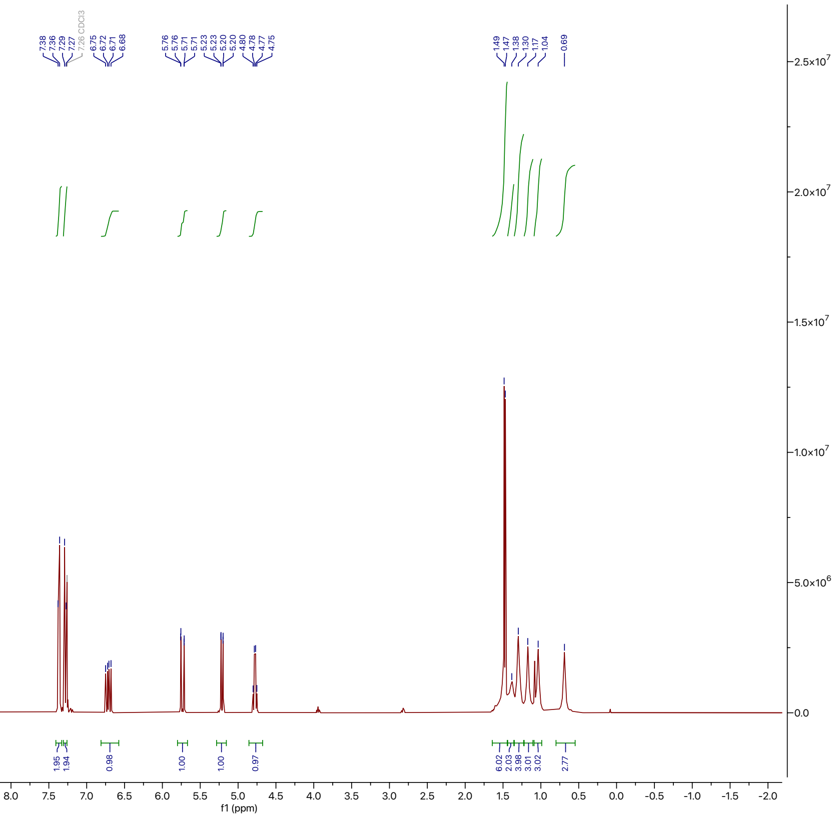

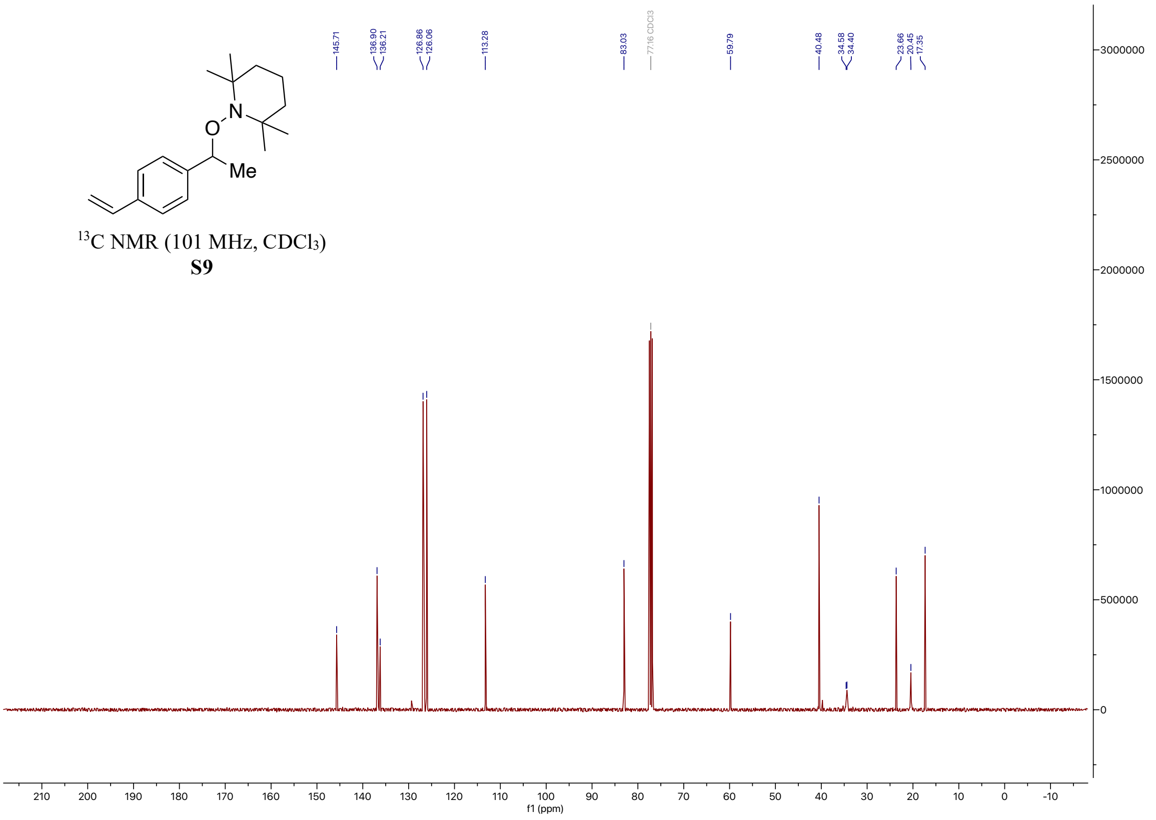

^{13}C NMR (101 MHz, $CDCl_3$)

S9

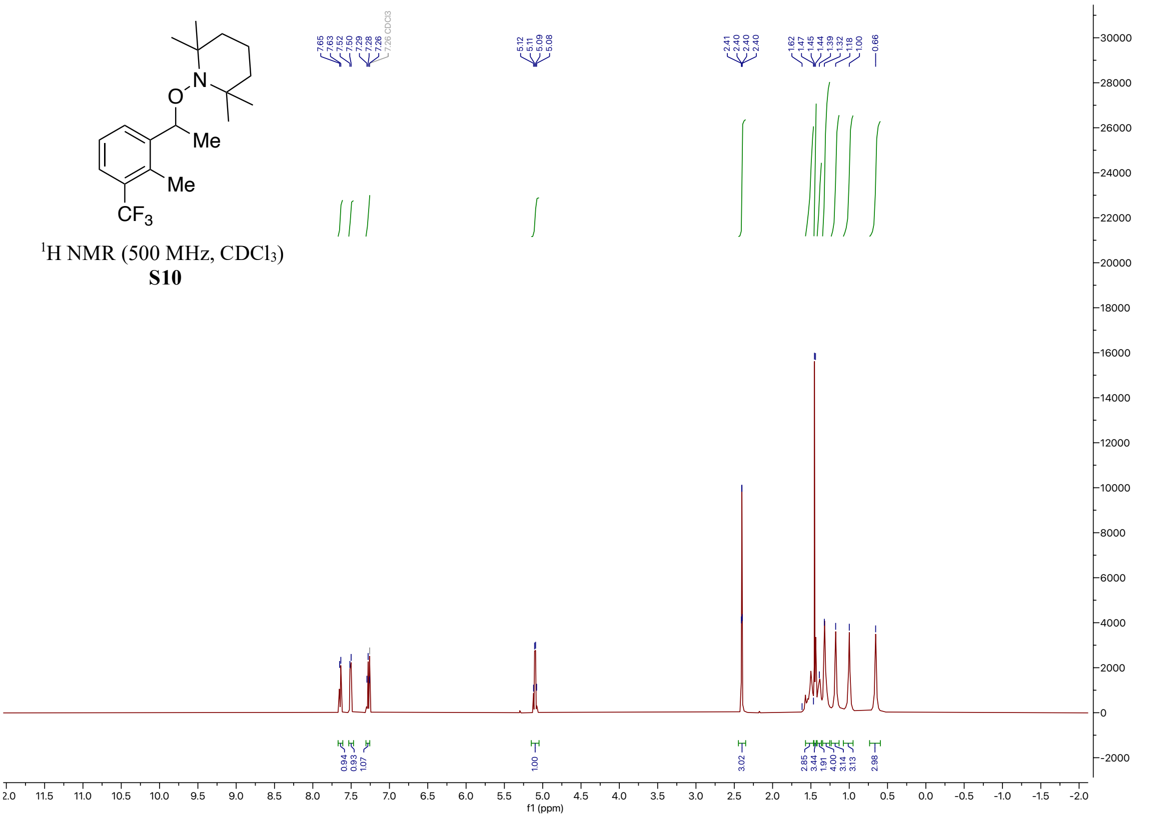

^{1}H NMR (500 MHz, $CDCl_3$)

S10

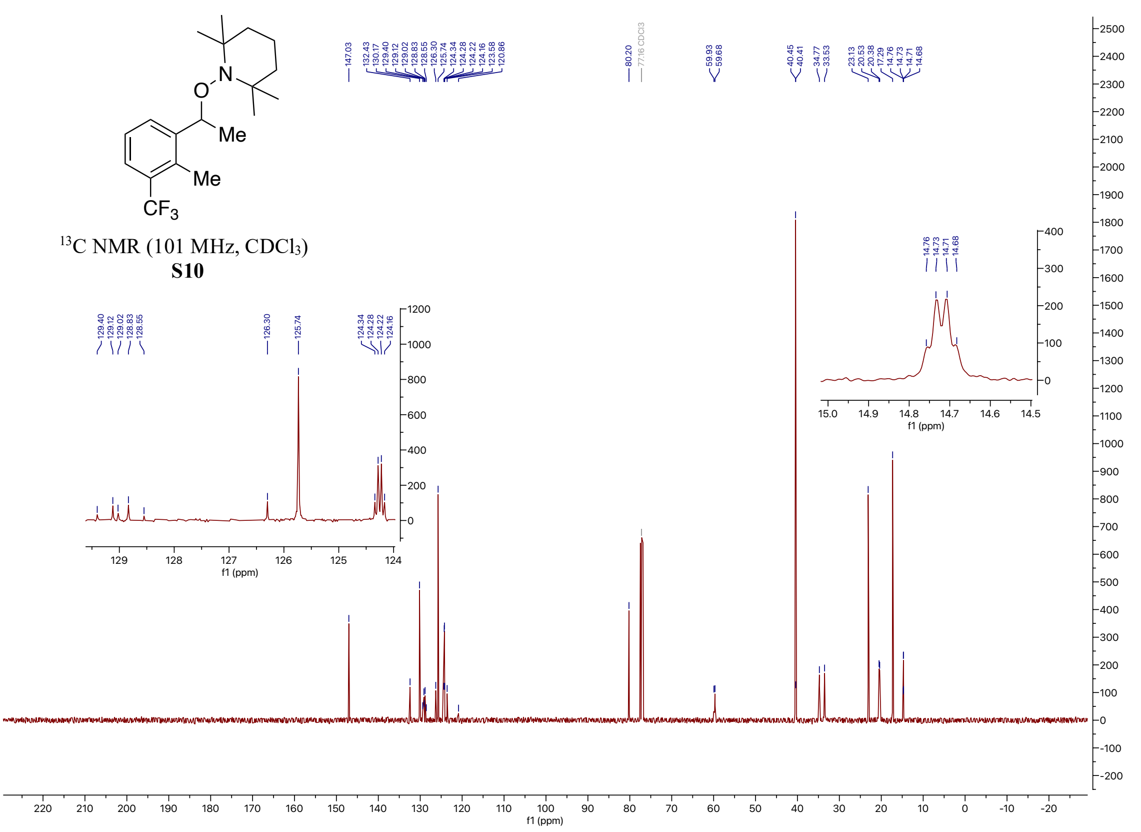

^{13}C NMR (101 MHz, $CDCl_3$)
S10

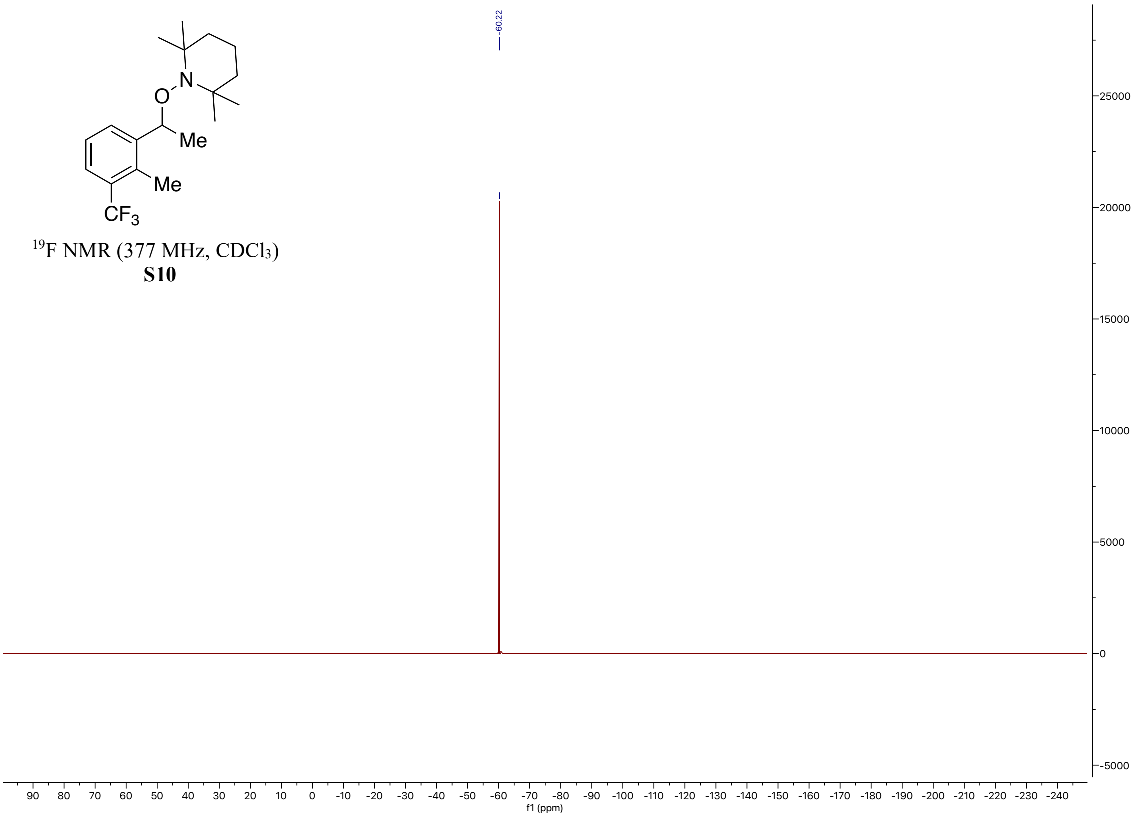

^{19}F NMR (377 MHz, $CDCl_3$)
S10

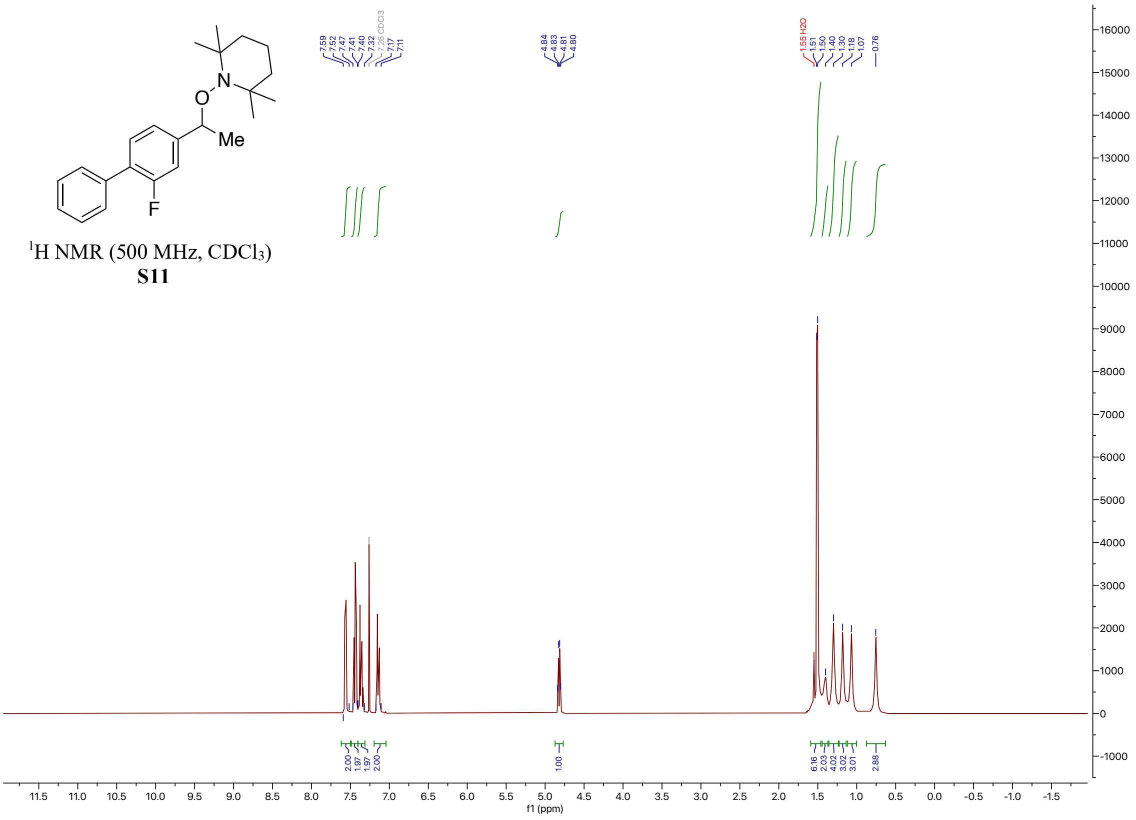
O
N
Me
F
¹H NMR (500 MHz, CDCl₃)
S11
f1 (ppm)

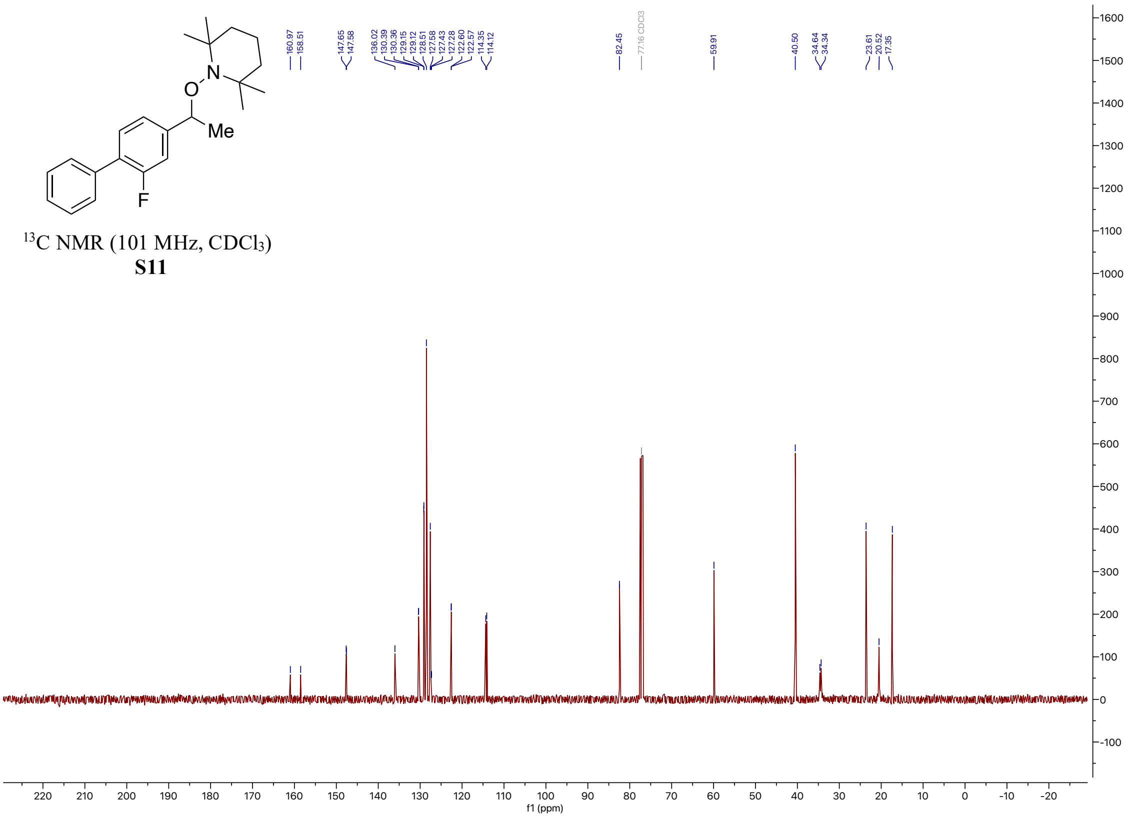
O
N
Me
F
¹³C NMR (101 MHz, CDCl₃)
S11
f1 (ppm)

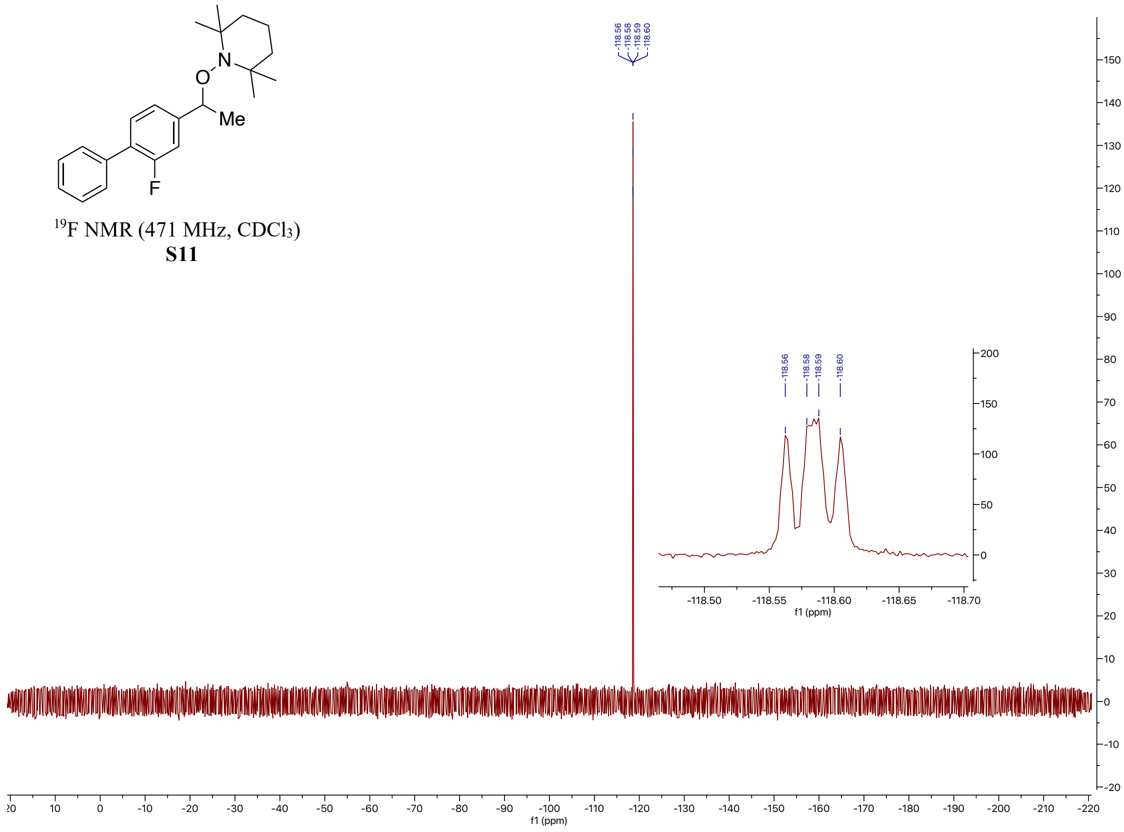

^{19}F NMR (471 MHz, $CDCl_3$)
S11

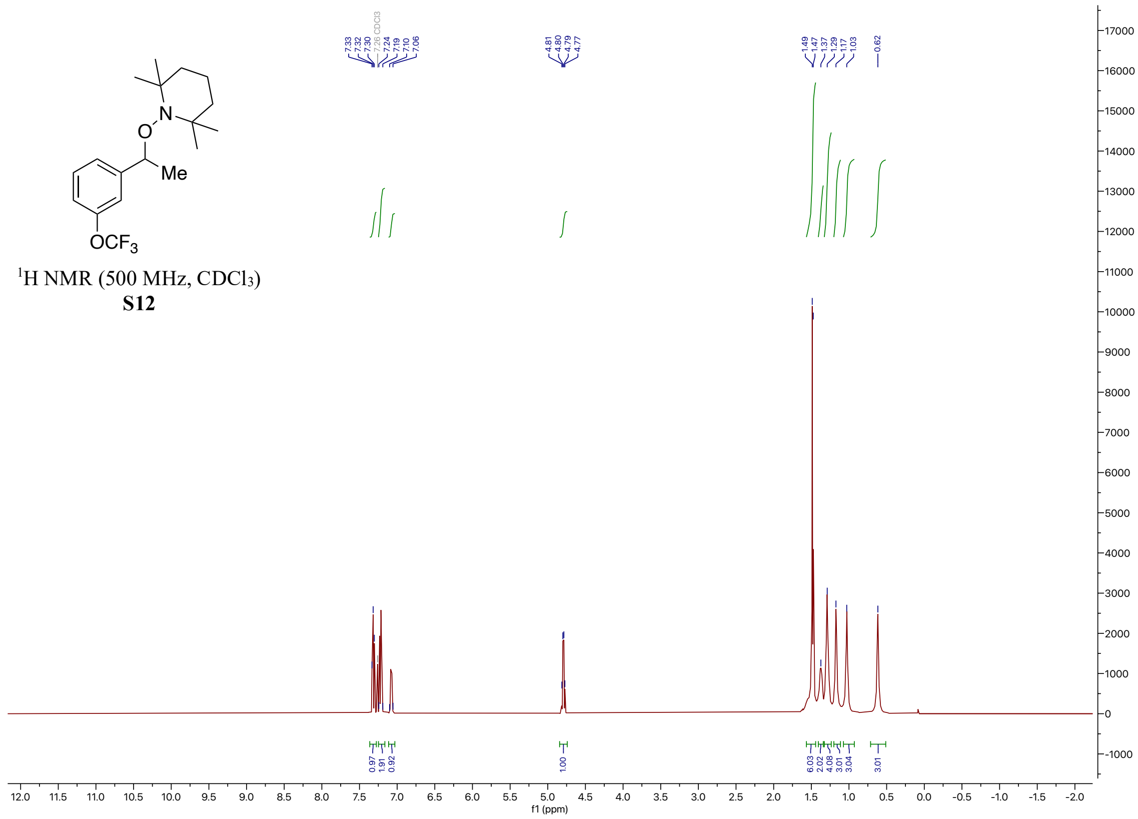

^{1}H NMR (500 MHz, $CDCl_3$)
S12

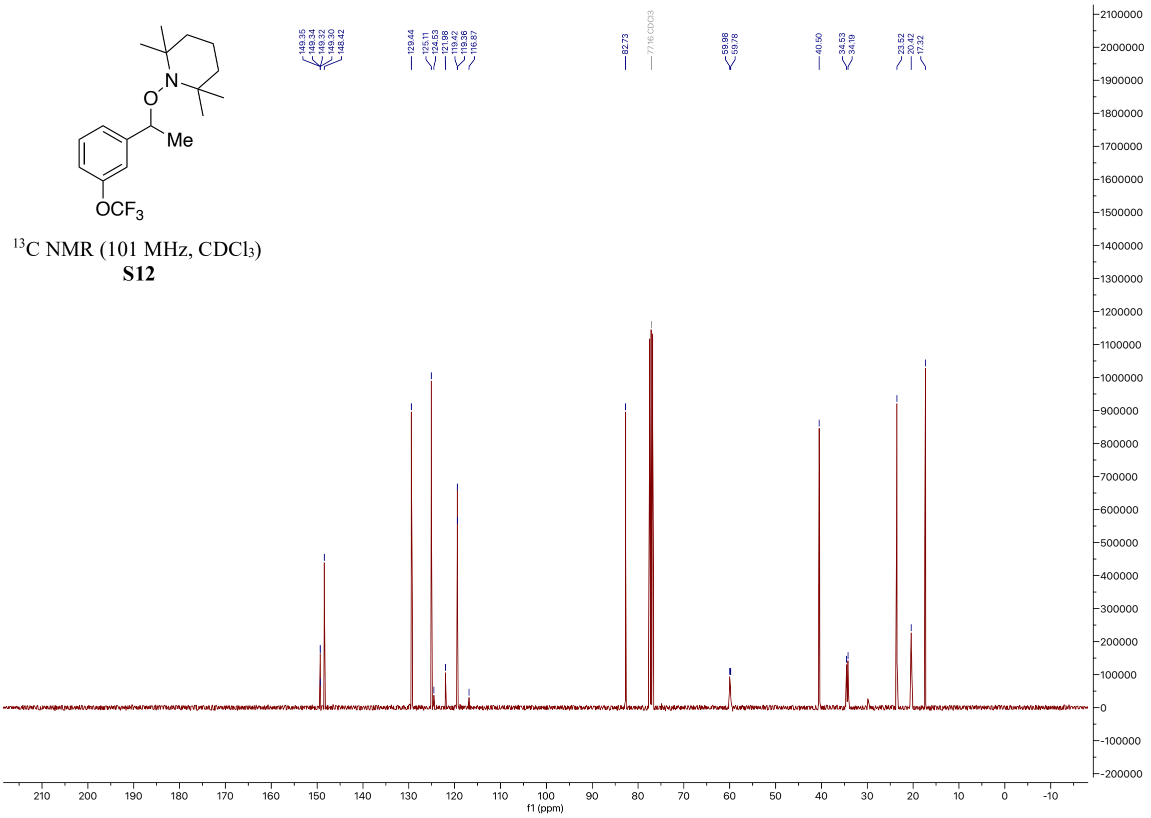

^{13}C NMR (101 MHz, $CDCl_3$)
S12

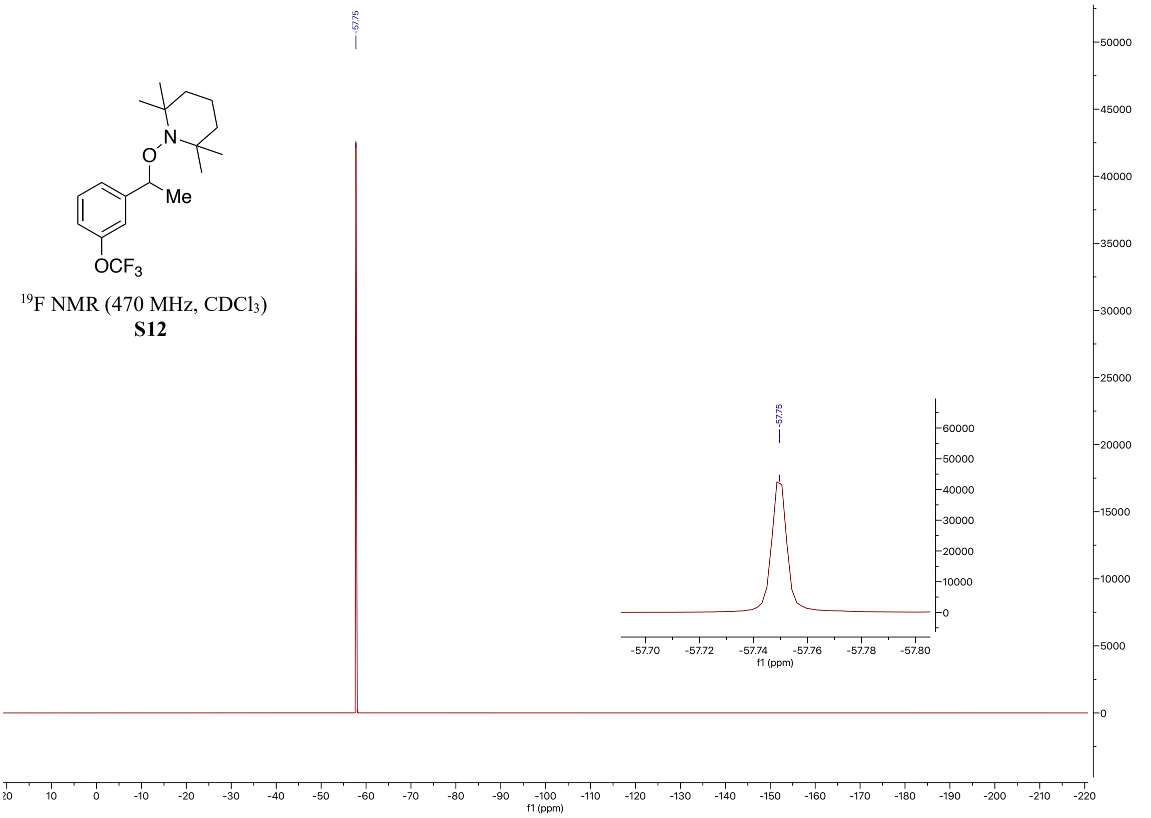

^{19}F NMR (470 MHz, $CDCl_3$)
S12

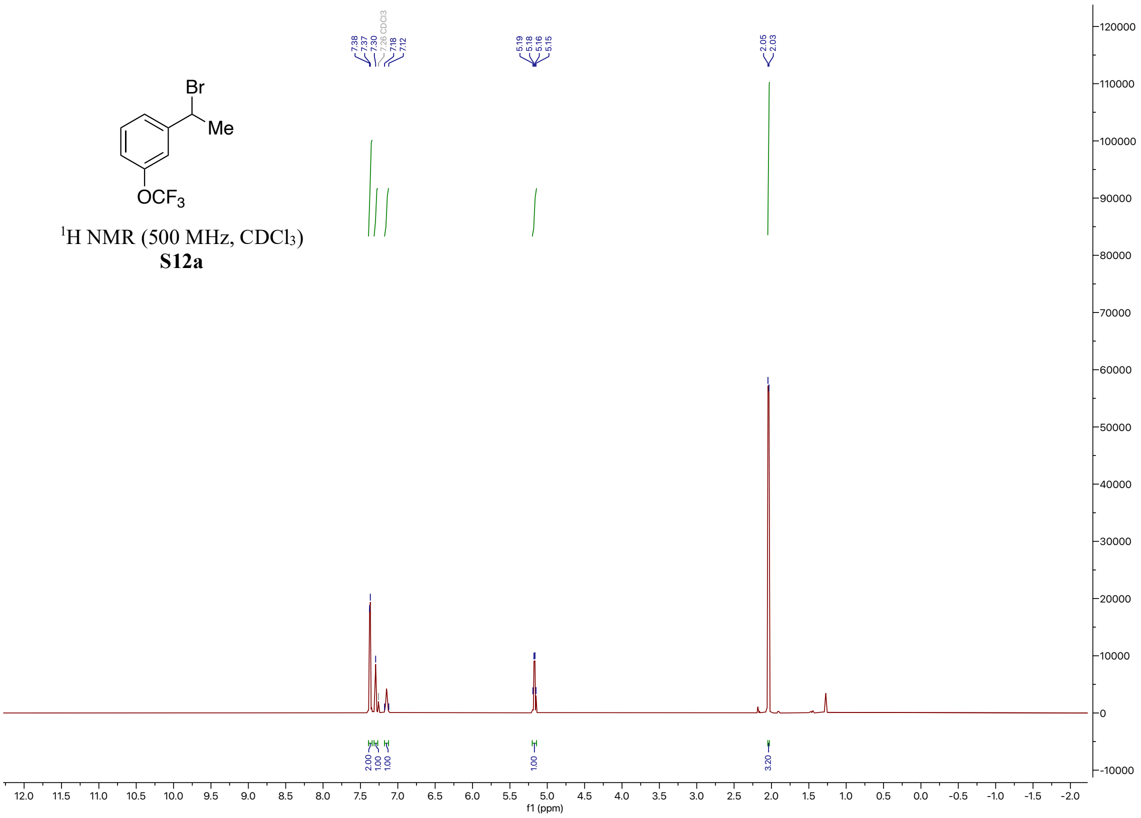

^{1}H NMR (500 MHz, $CDCl_3$)
S12a

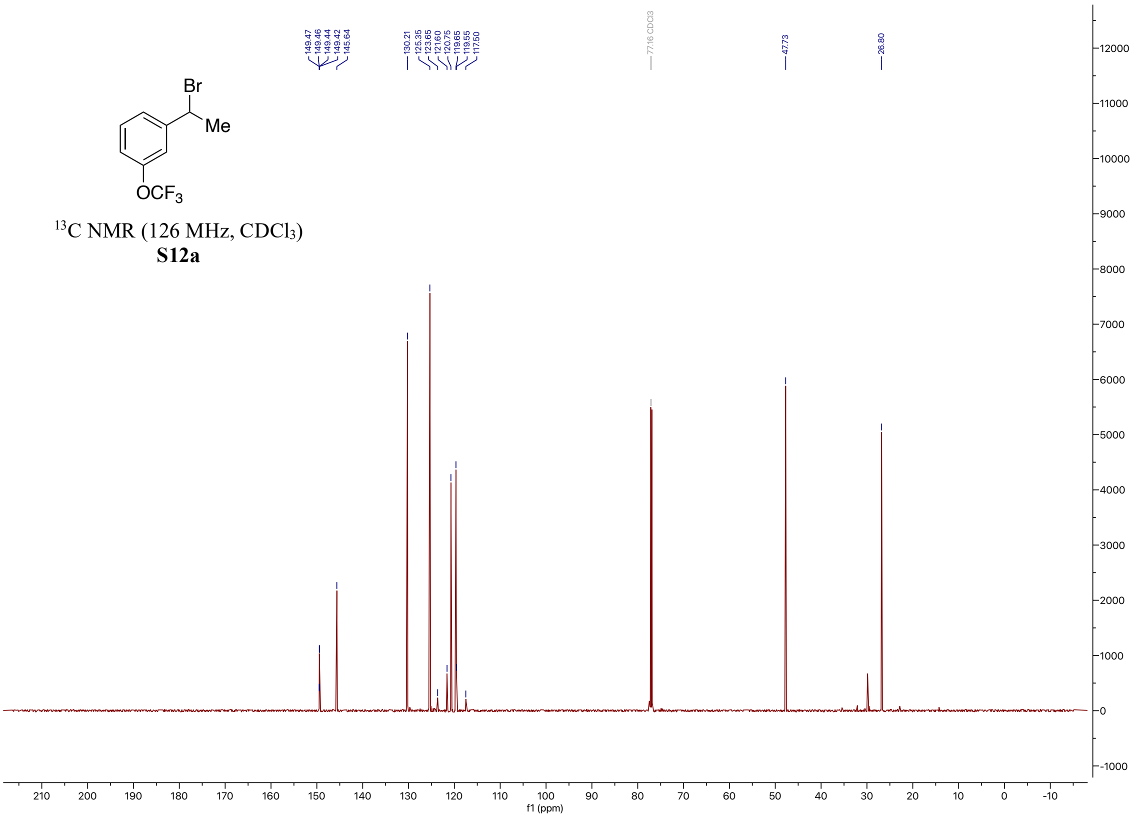

^{13}C NMR (126 MHz, $CDCl_3$)
S12a

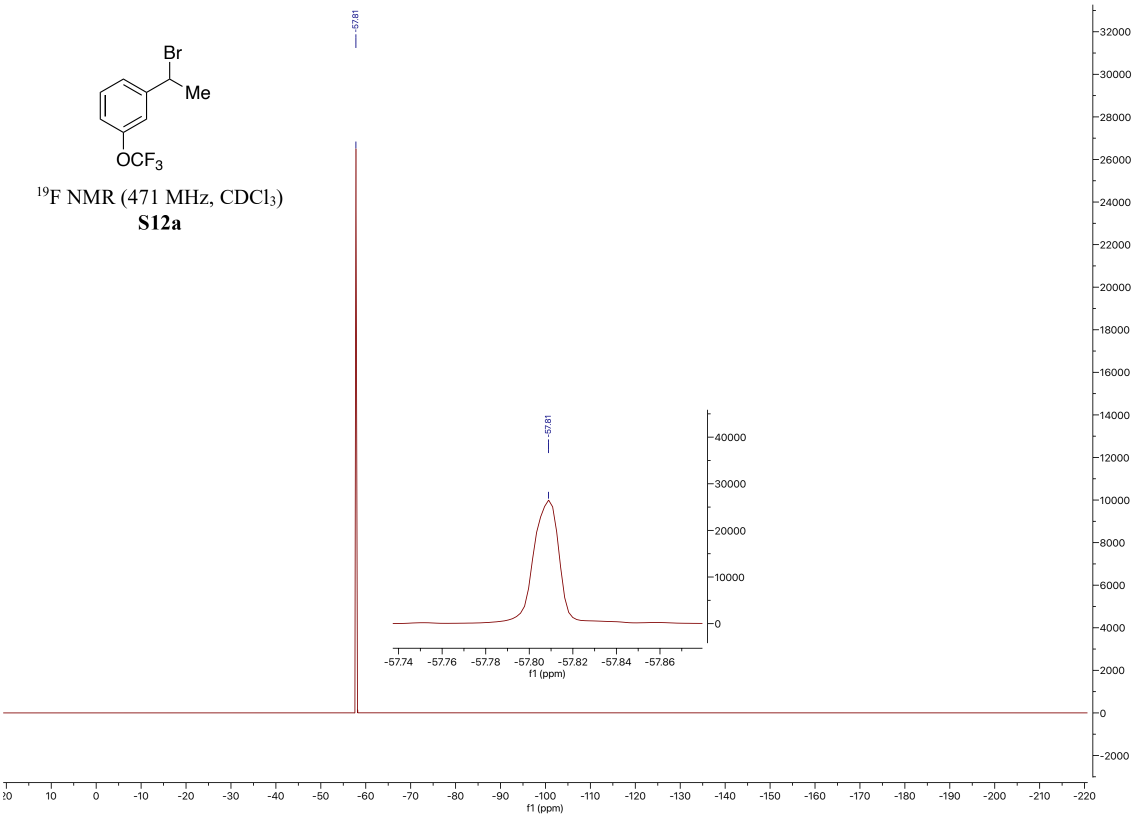

^{19}F NMR (471 MHz, $CDCl_3$)
S12a

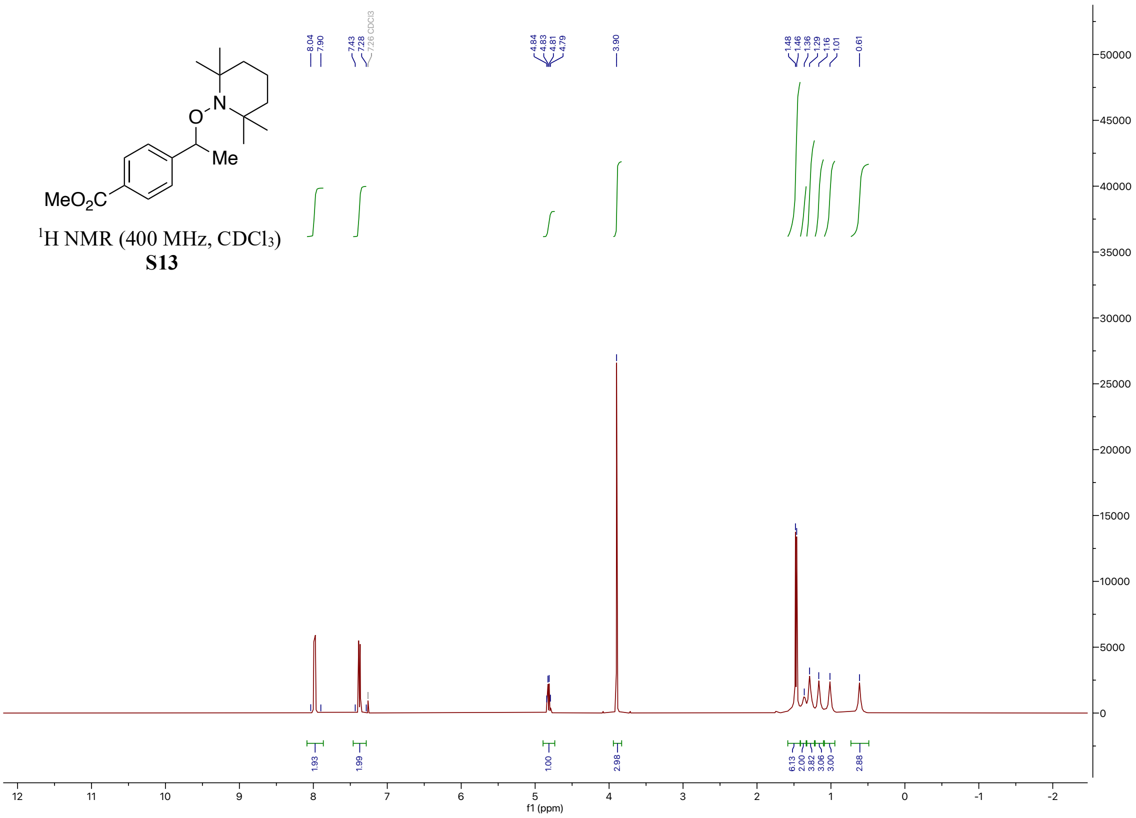

^{1}H NMR (400 MHz, $CDCl_3$)
S13

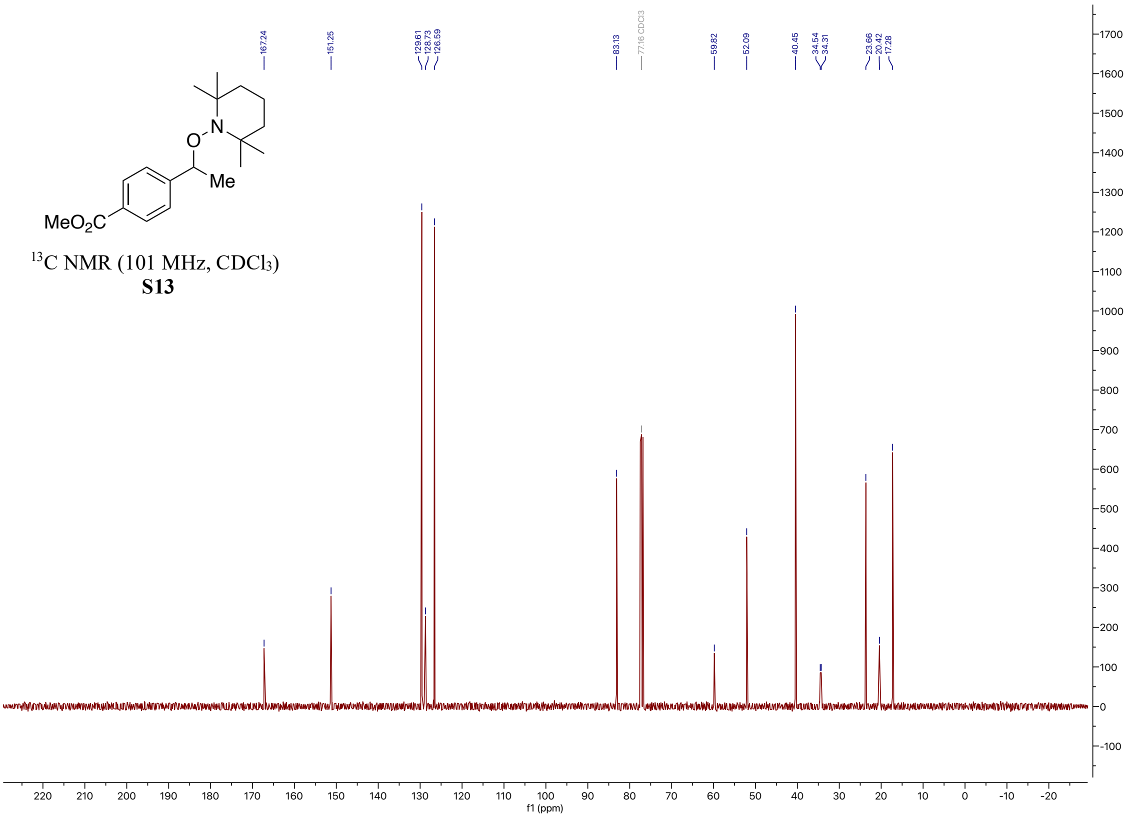

^{13}C NMR (101 MHz, $CDCl_3$)
S13

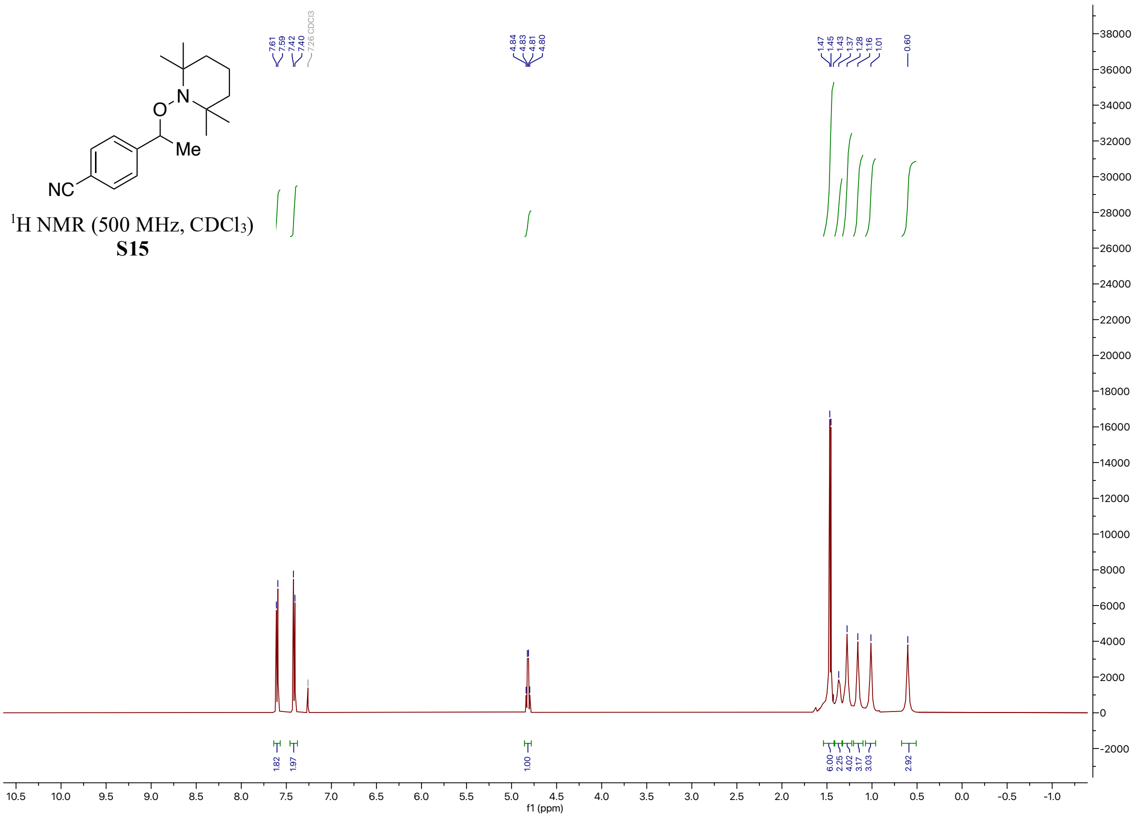

^{1}H NMR (500 MHz, $CDCl_3$)
S15

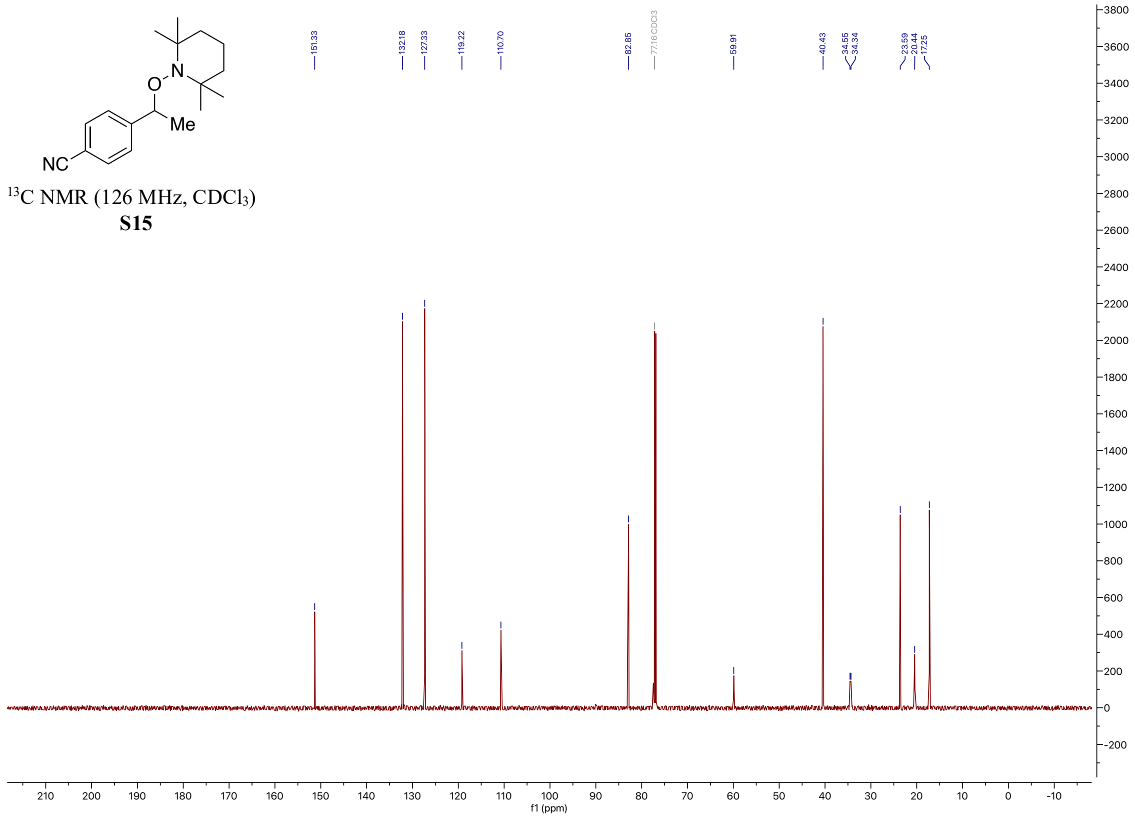
NC
Me
O
N
13C NMR (126 MHz, CDCl3)
S15
151.33
132.18
127.33
119.22
110.70
82.85
77.16 CDCl3
59.91
40.43
34.55
34.34
23.59
20.44
17.25
f1 (ppm)

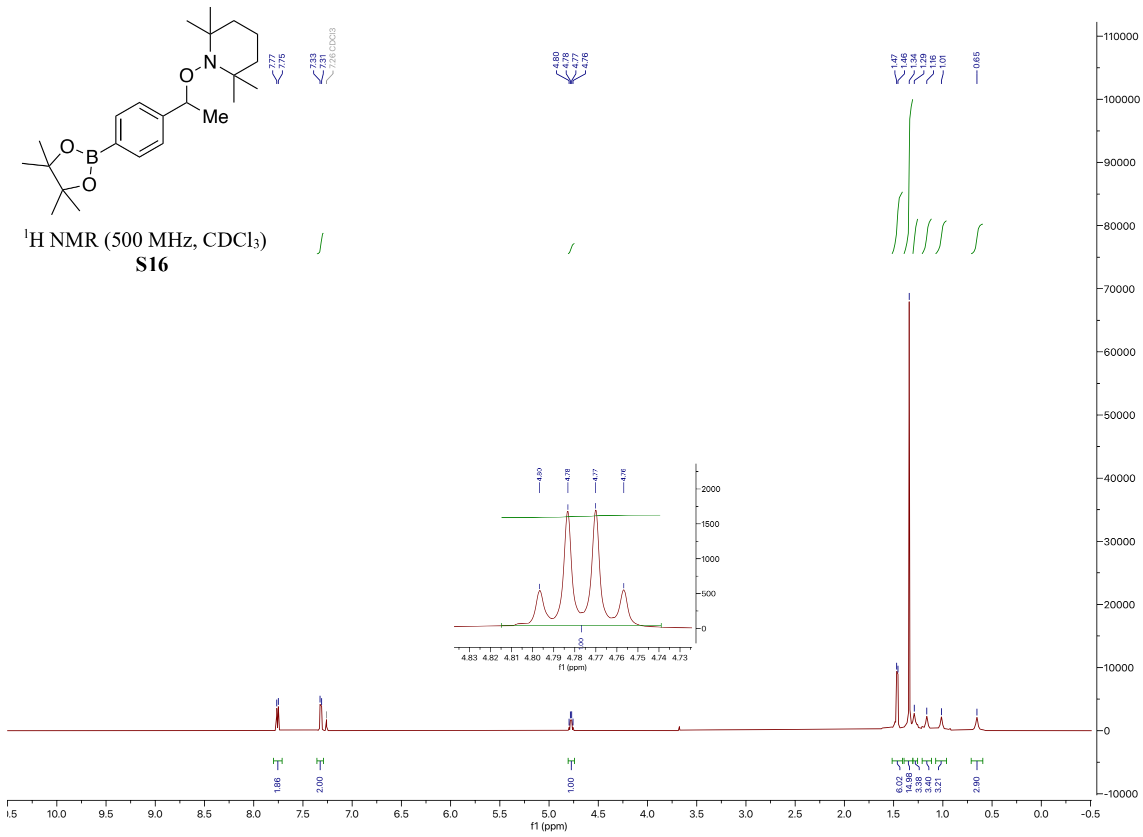
O
B
O
Me
O
N
1H NMR (500 MHz, CDCl3)
S16
7.77
7.75
7.33
7.31
7.26 CDCl3
4.80
4.78
4.77
4.76
1.47
1.46
1.34
1.29
1.16
1.01
0.65
1.86
2.00
1.00
6.02
14.98
3.38
3.40
3.21
2.90
f1 (ppm)

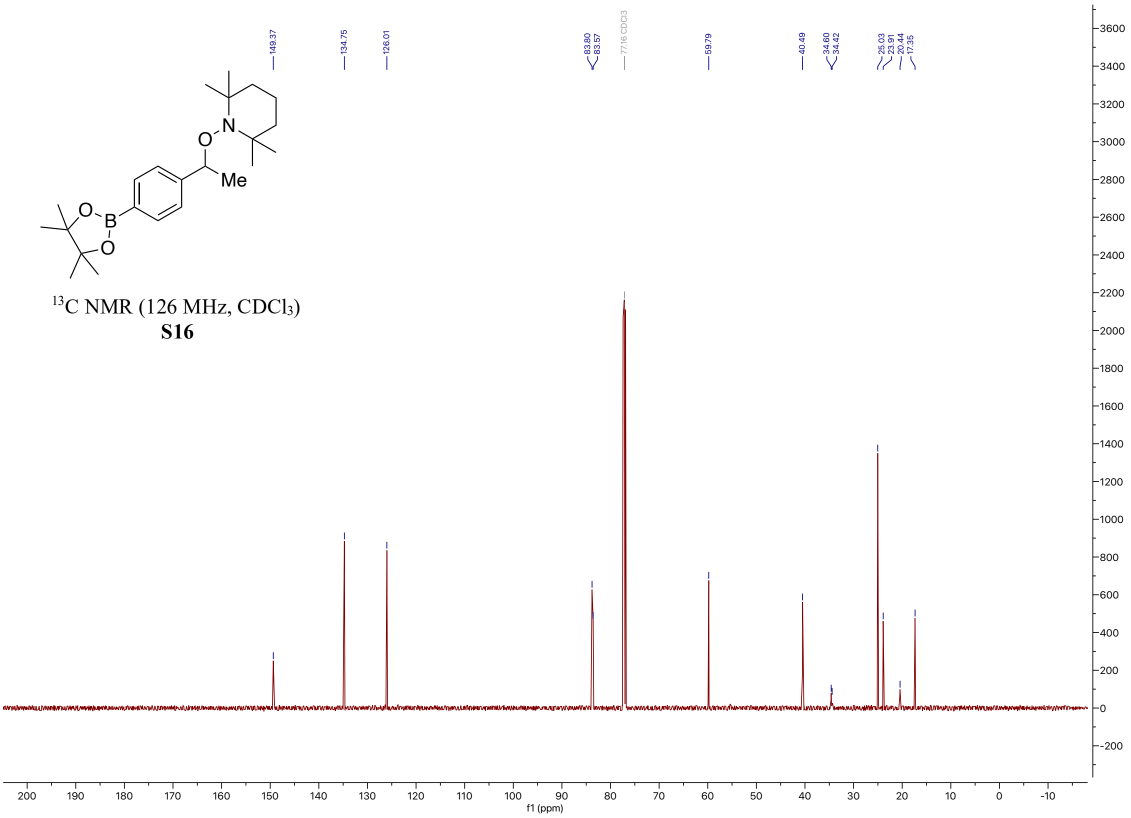

^{13}C NMR (126 MHz, $CDCl_3$)
S16

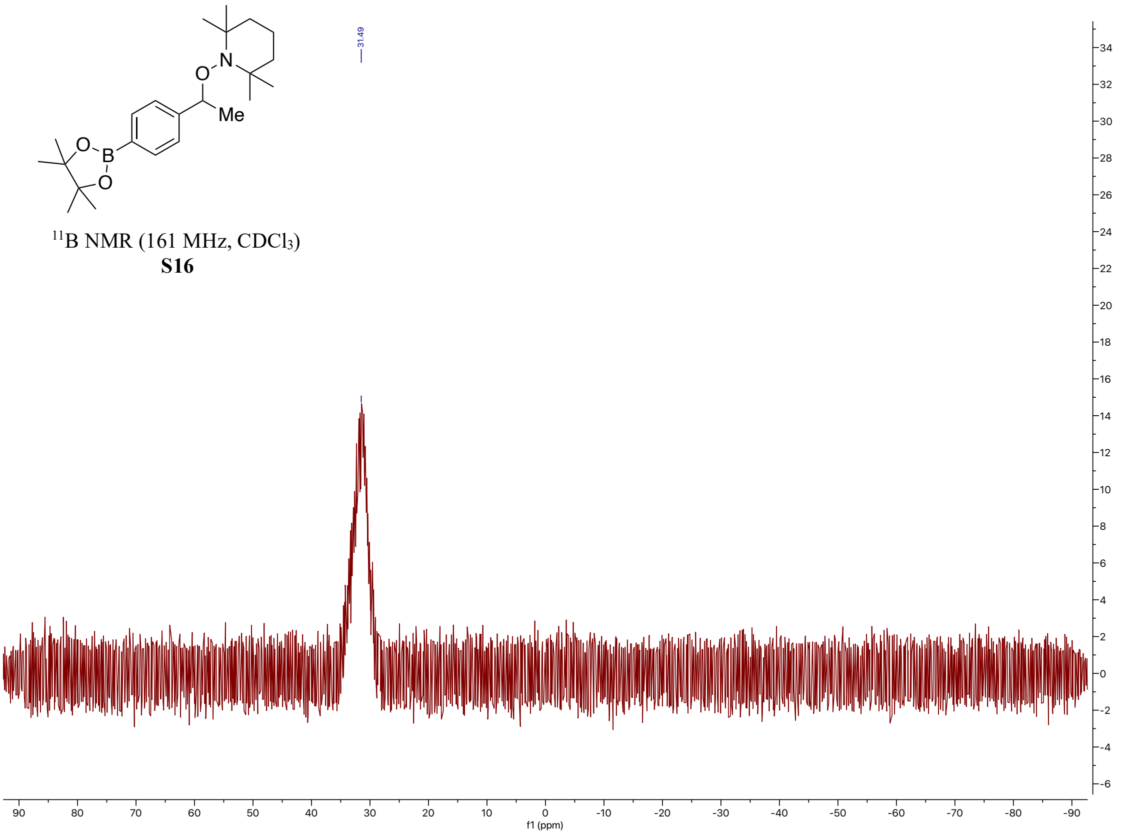

^{11}B NMR (161 MHz, $CDCl_3$)
S16

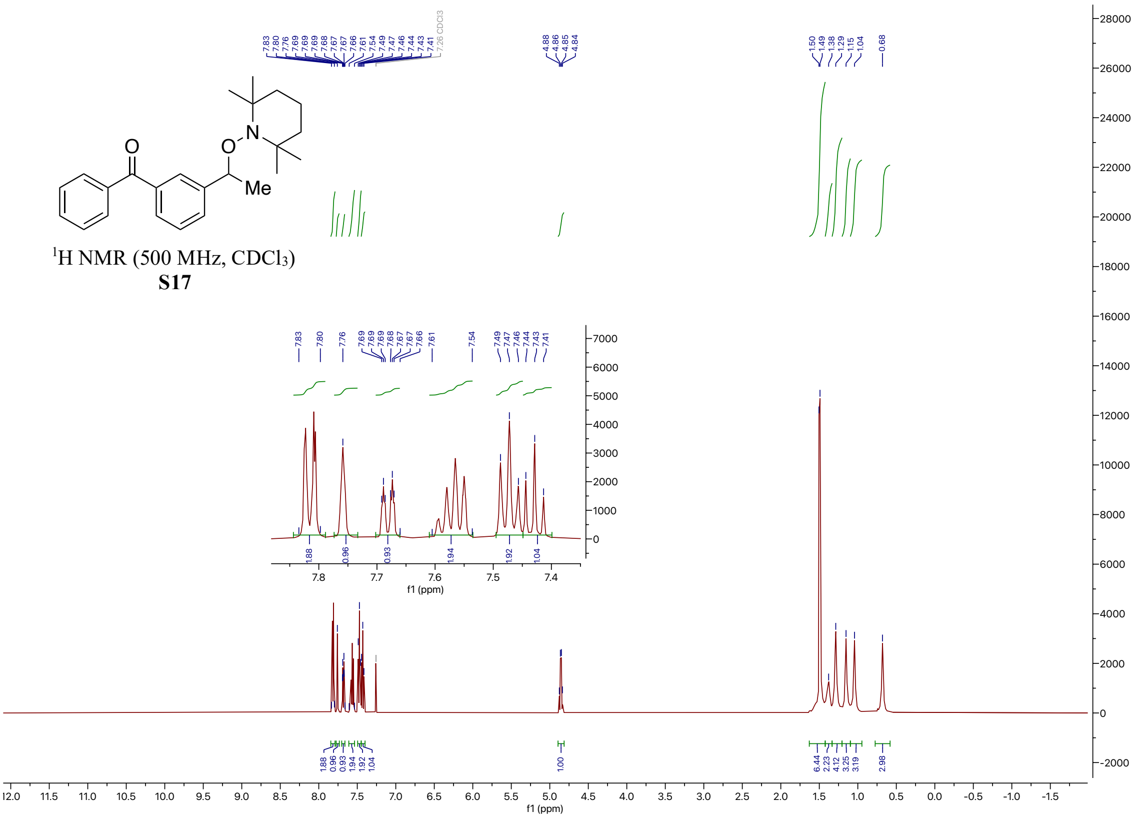

^{1}H NMR (500 MHz, $CDCl_3$)
S17

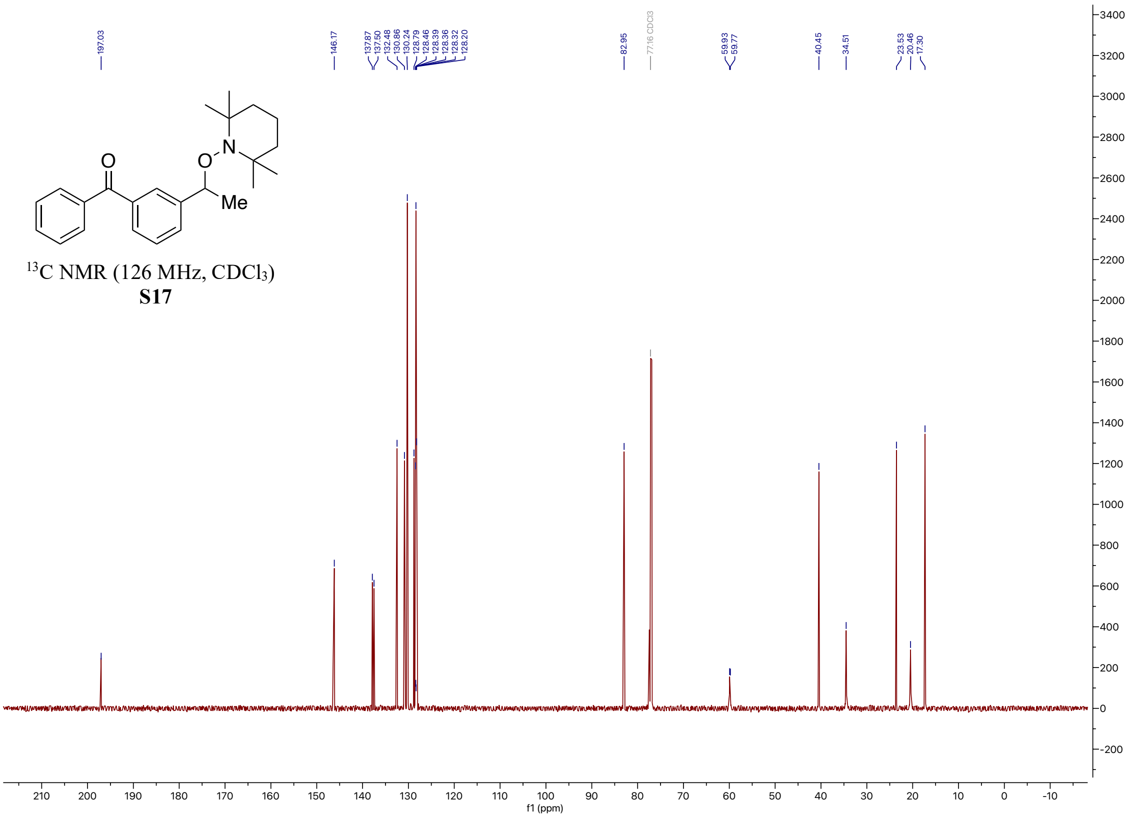

^{13}C NMR (126 MHz, $CDCl_3$)
S17

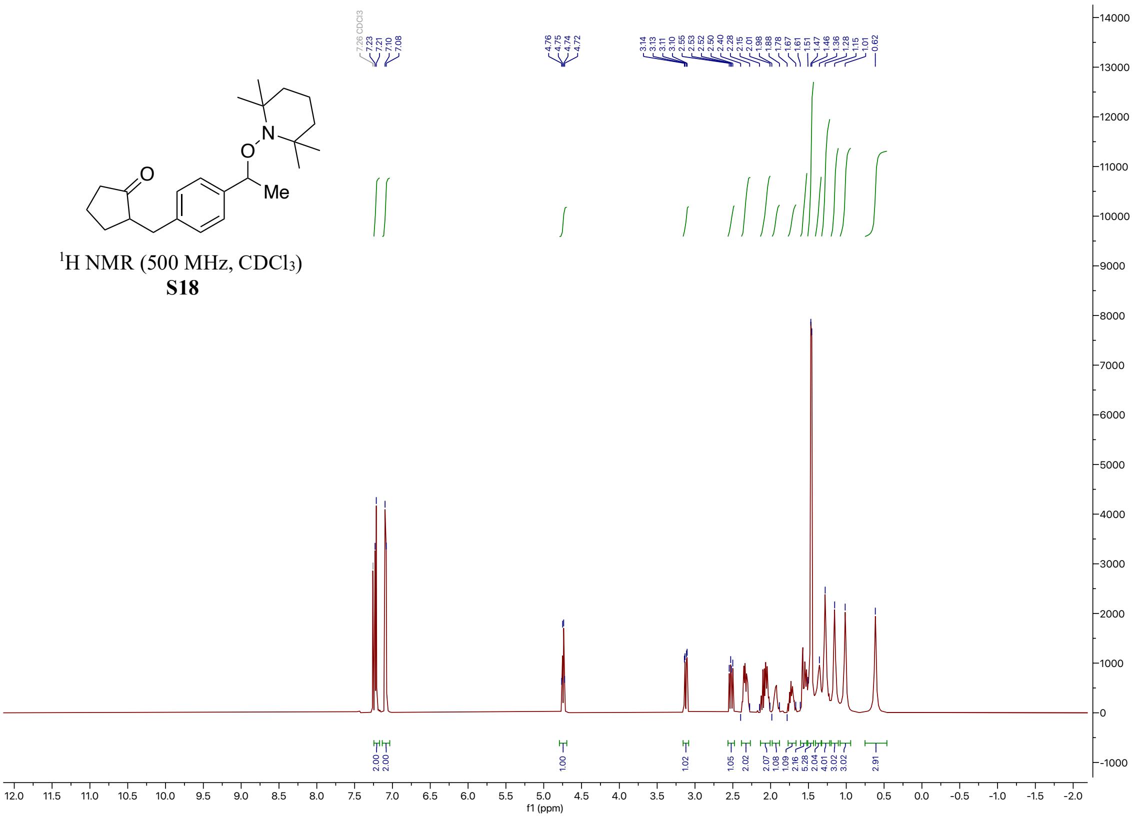

^{1}H NMR (500 MHz, $CDCl_3$)
S18

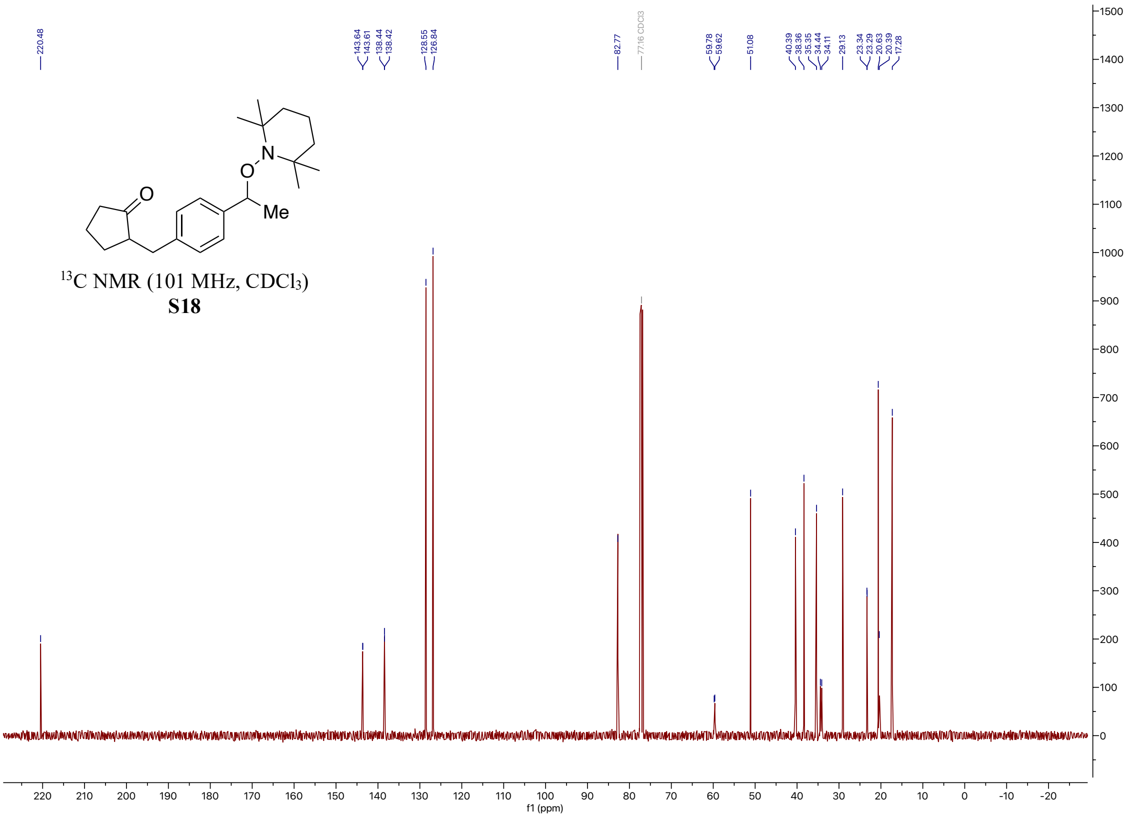

^{13}C NMR (101 MHz, $CDCl_3$)
S18

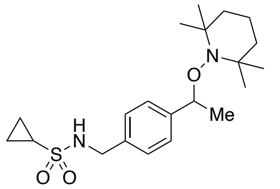

^{1}H NMR (400 MHz, $CDCl_3$)
S19

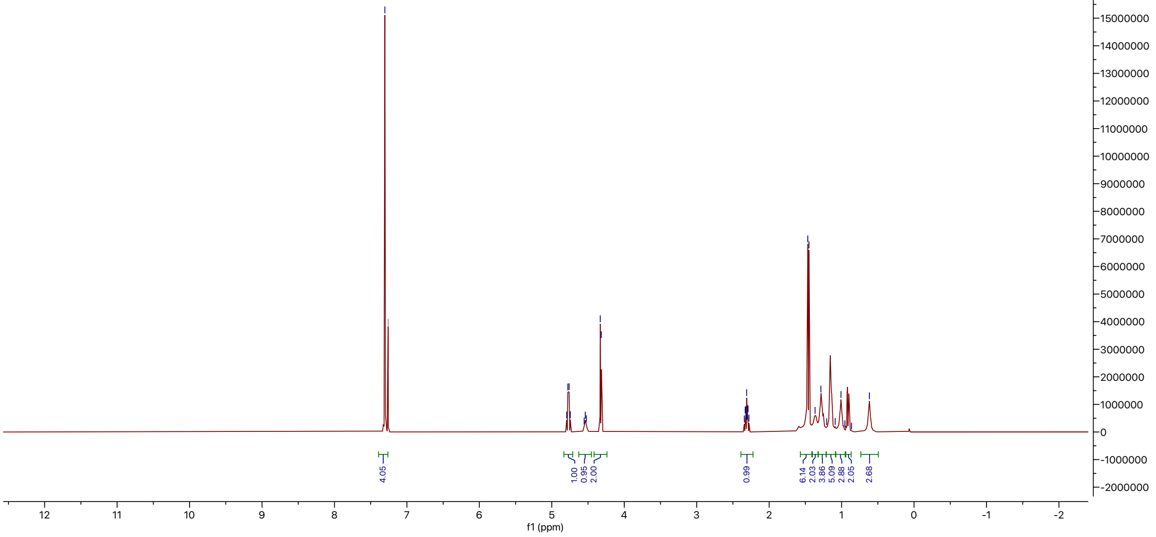

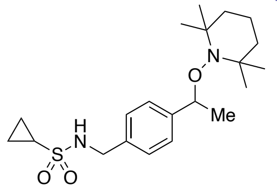

^{13}C NMR (101 MHz, $CDCl_3$)
S19

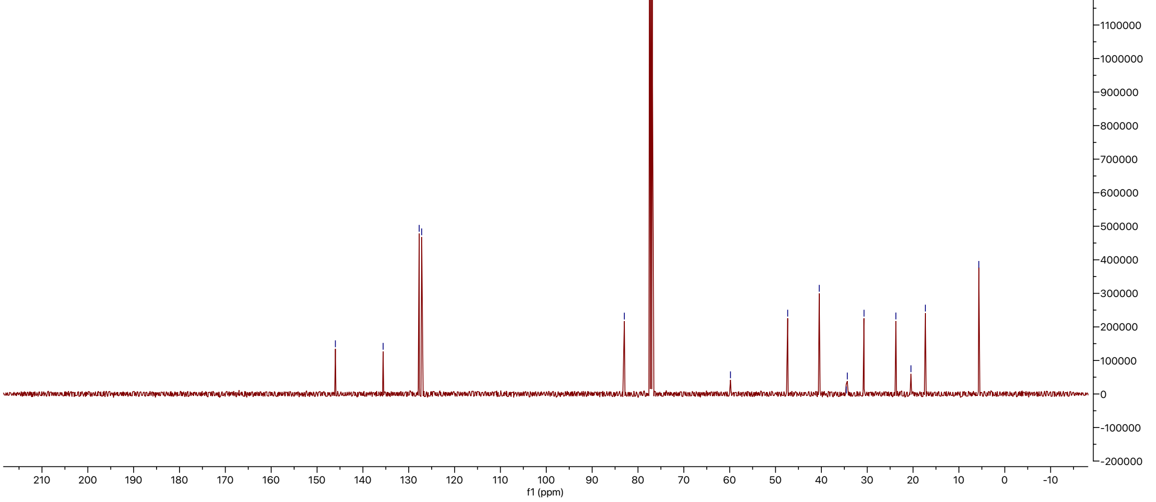

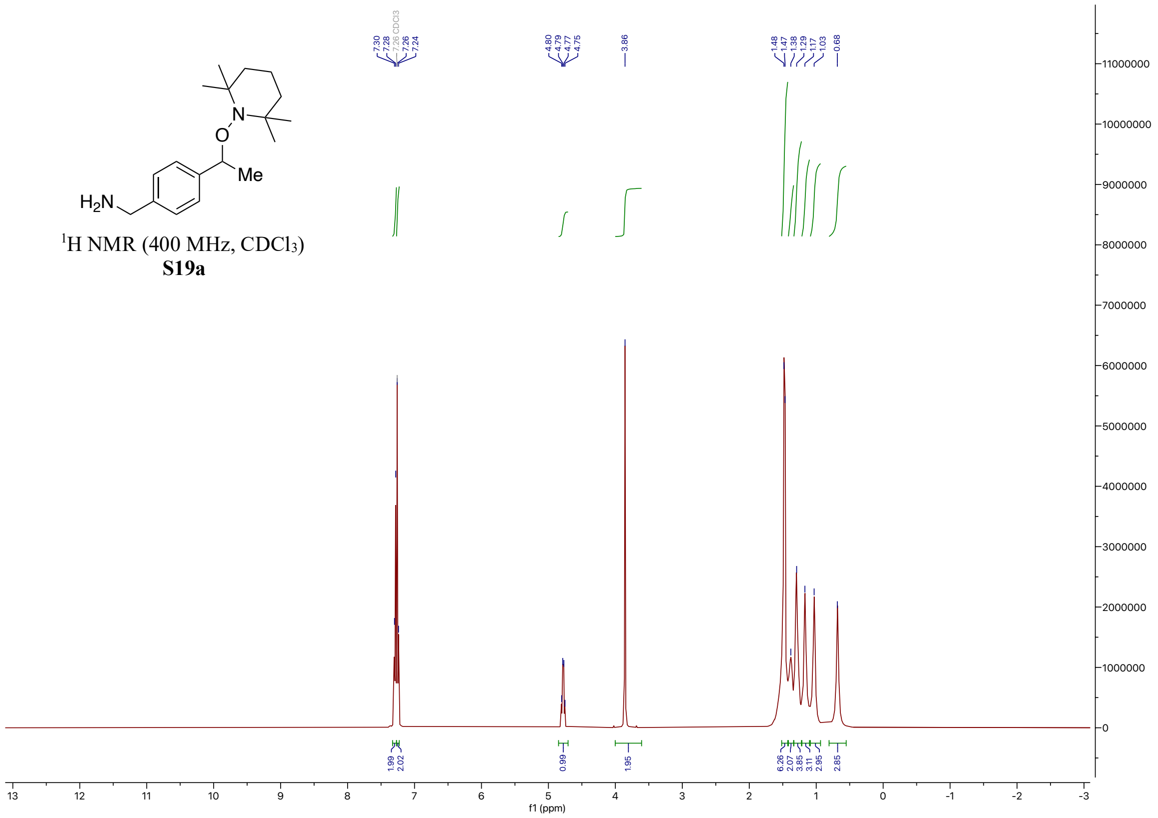

^{1}H NMR (400 MHz, $CDCl_3$)
S19a

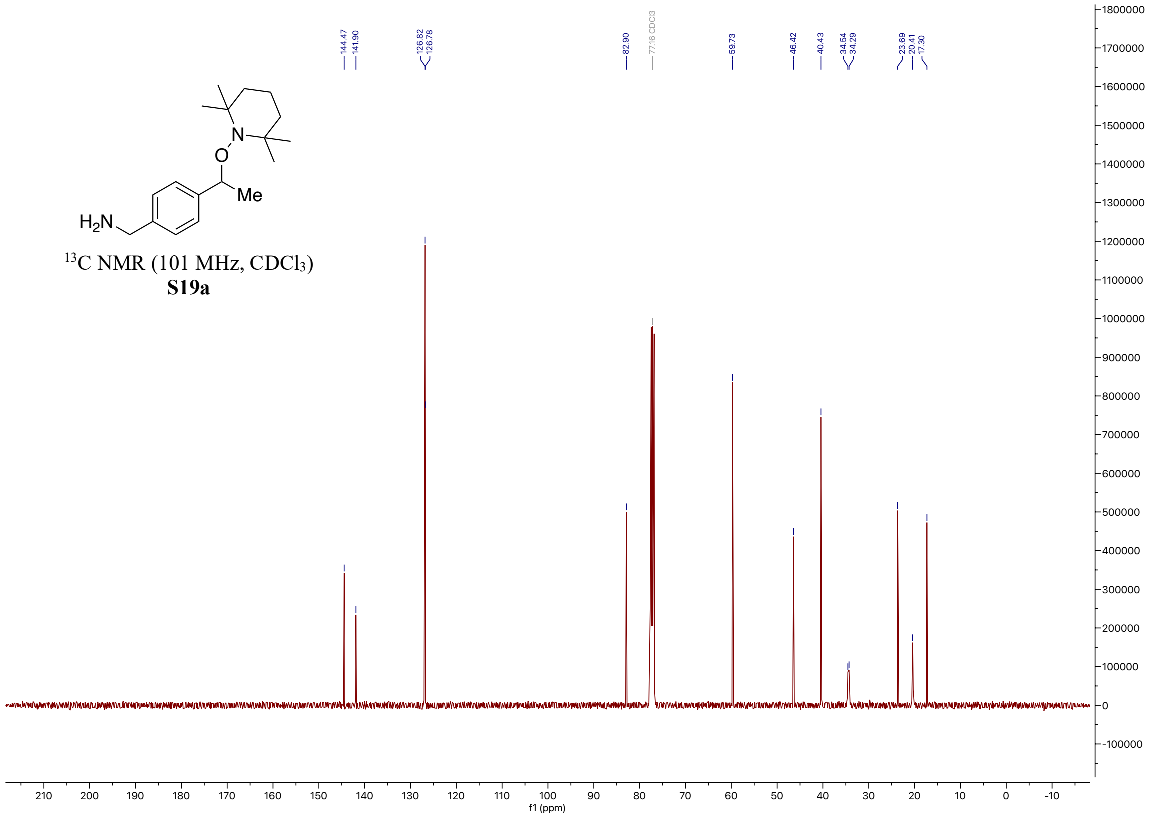

^{13}C NMR (101 MHz, $CDCl_3$)
S19a

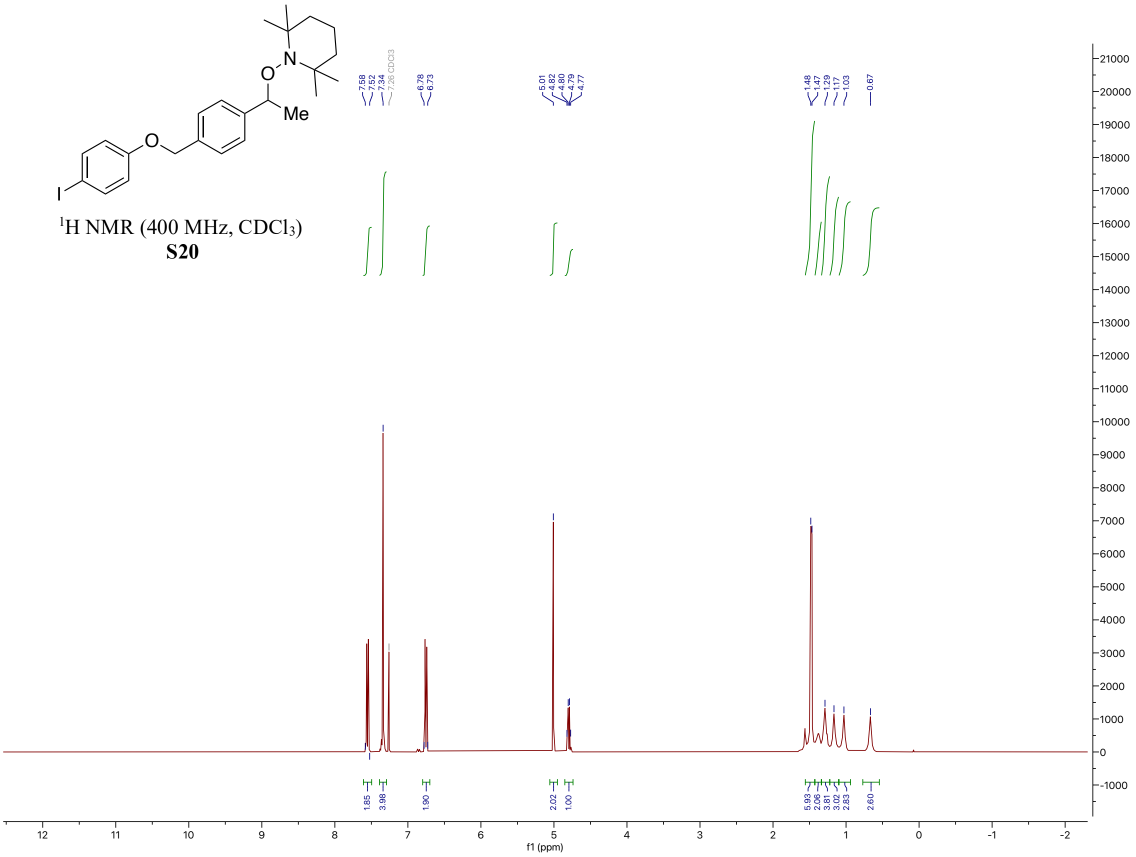

^{1}H NMR (400 MHz, $CDCl_3$)

S20

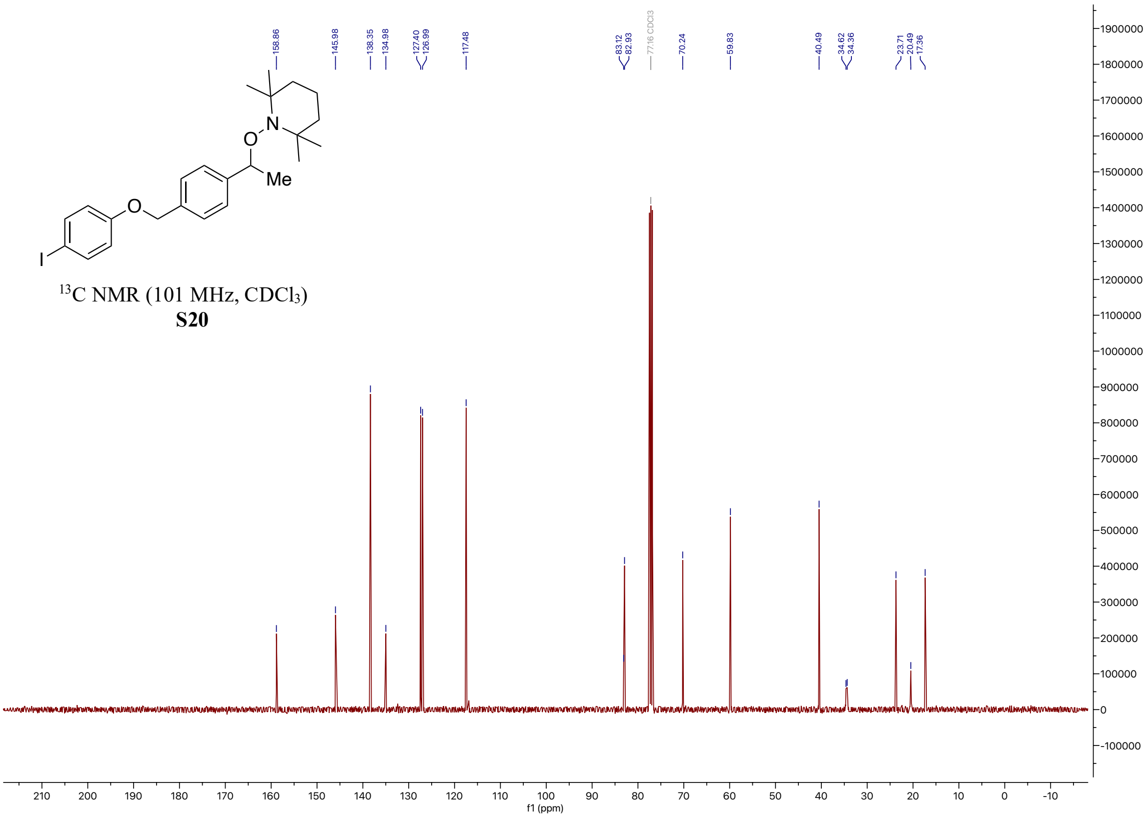

^{13}C NMR (101 MHz, $CDCl_3$)

S20

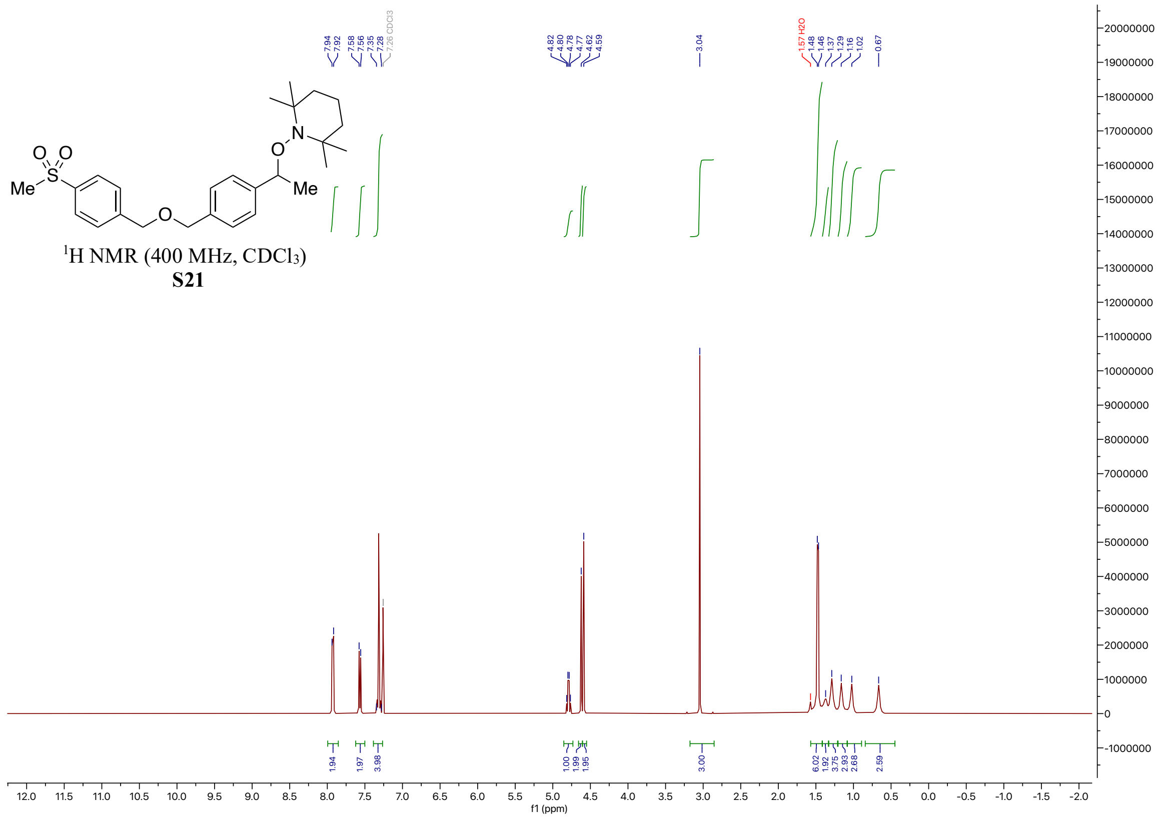

^1H NMR (400 MHz, $CDCl_3$)
S21

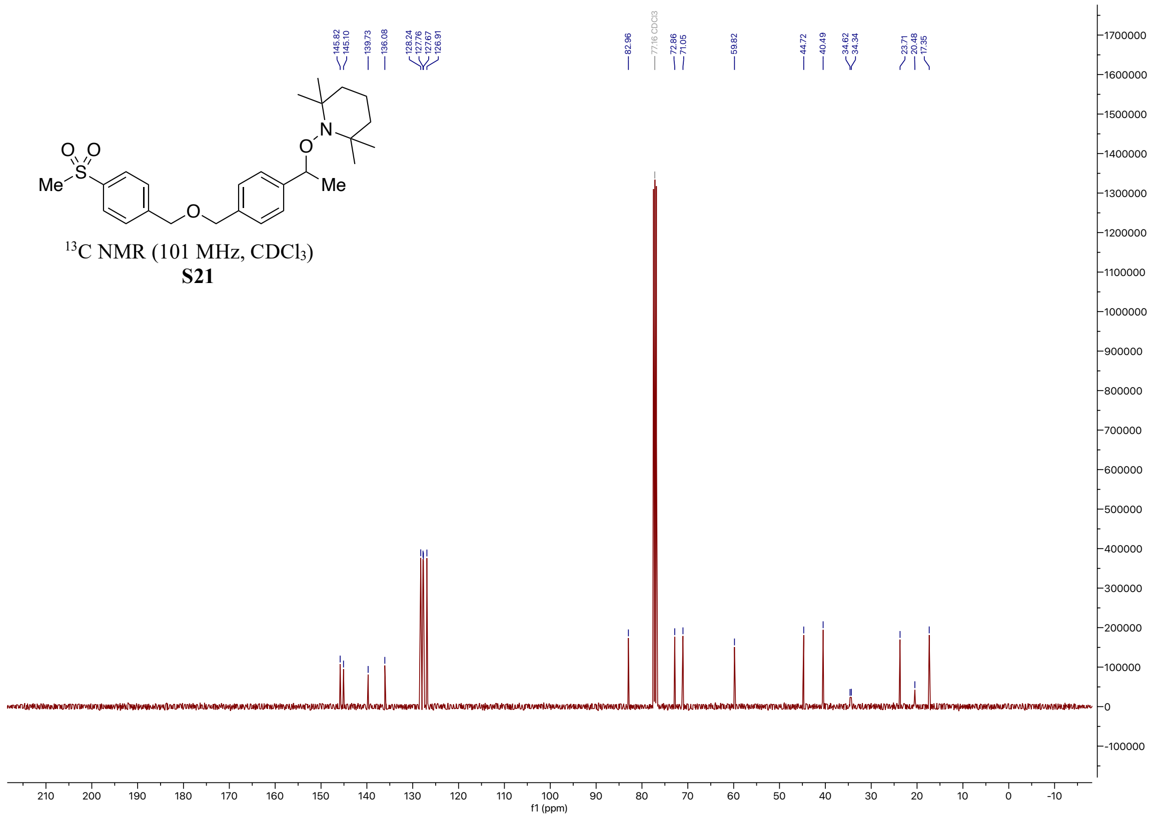

^{13}C NMR (101 MHz, $CDCl_3$)
S21

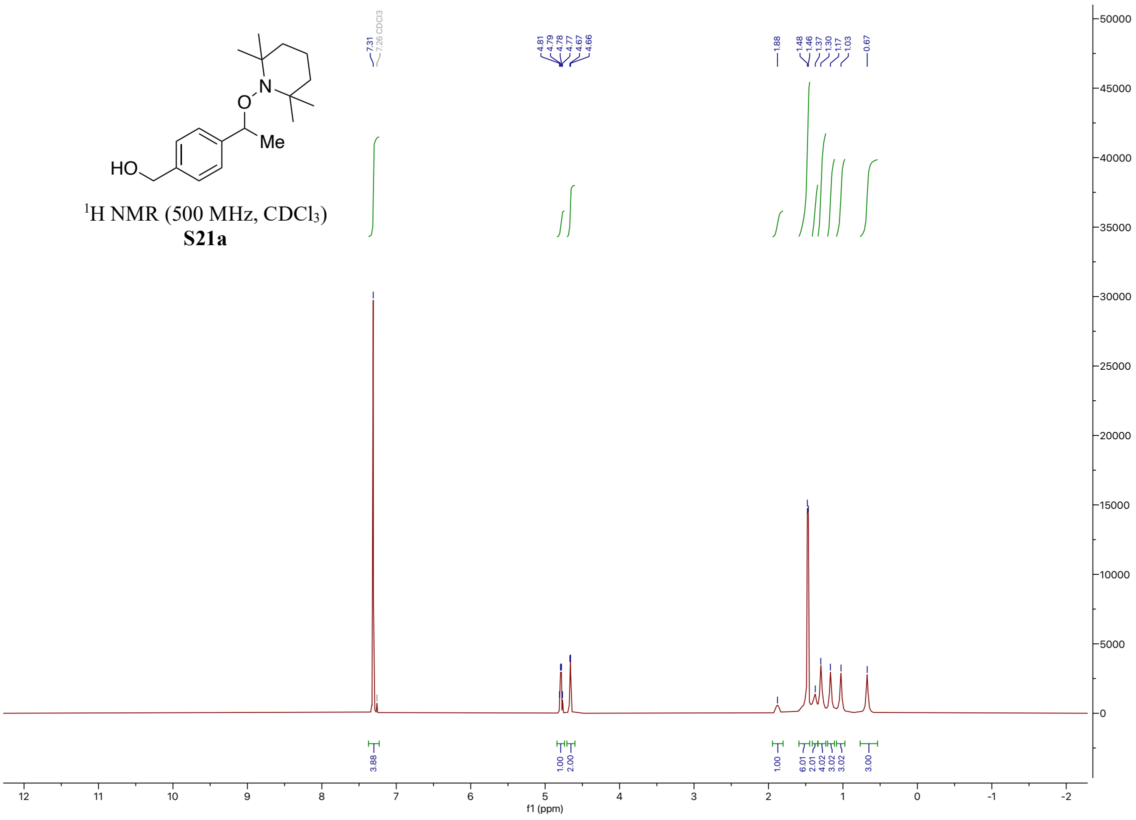

^{1}H NMR (500 MHz, $CDCl_3$)
S21a

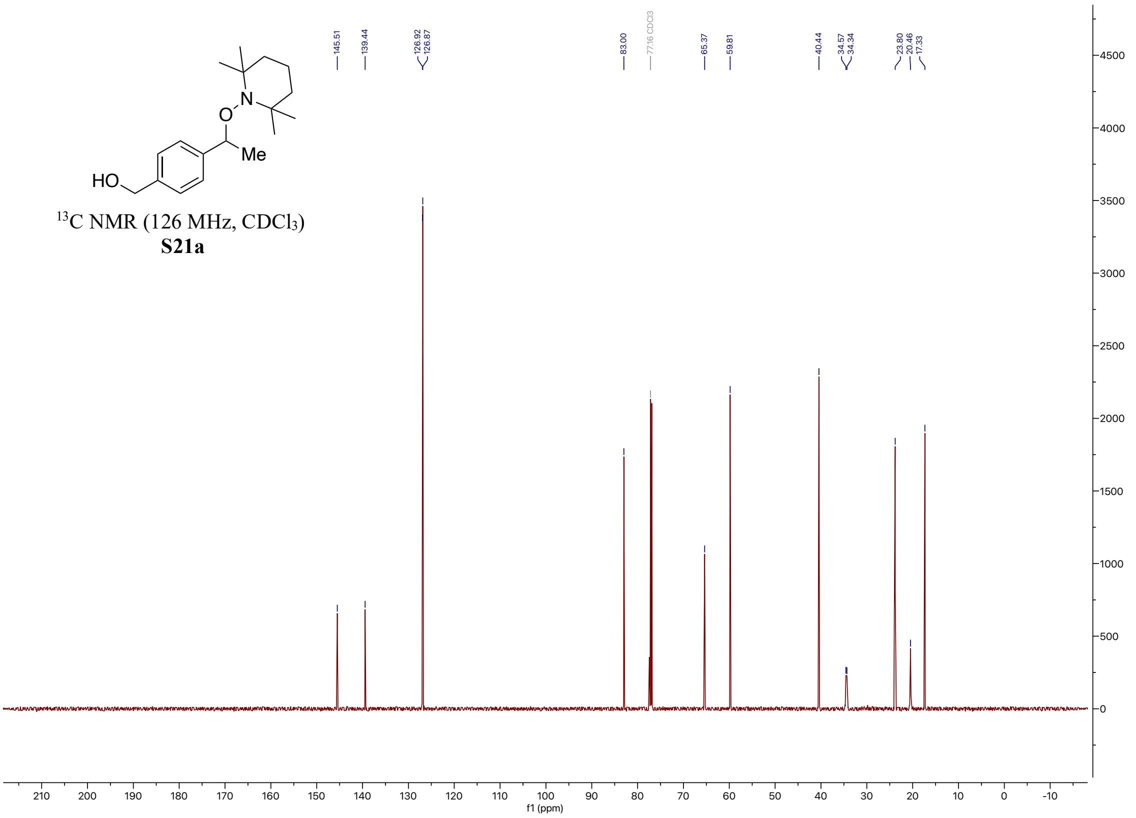

^{13}C NMR (126 MHz, $CDCl_3$)
S21a

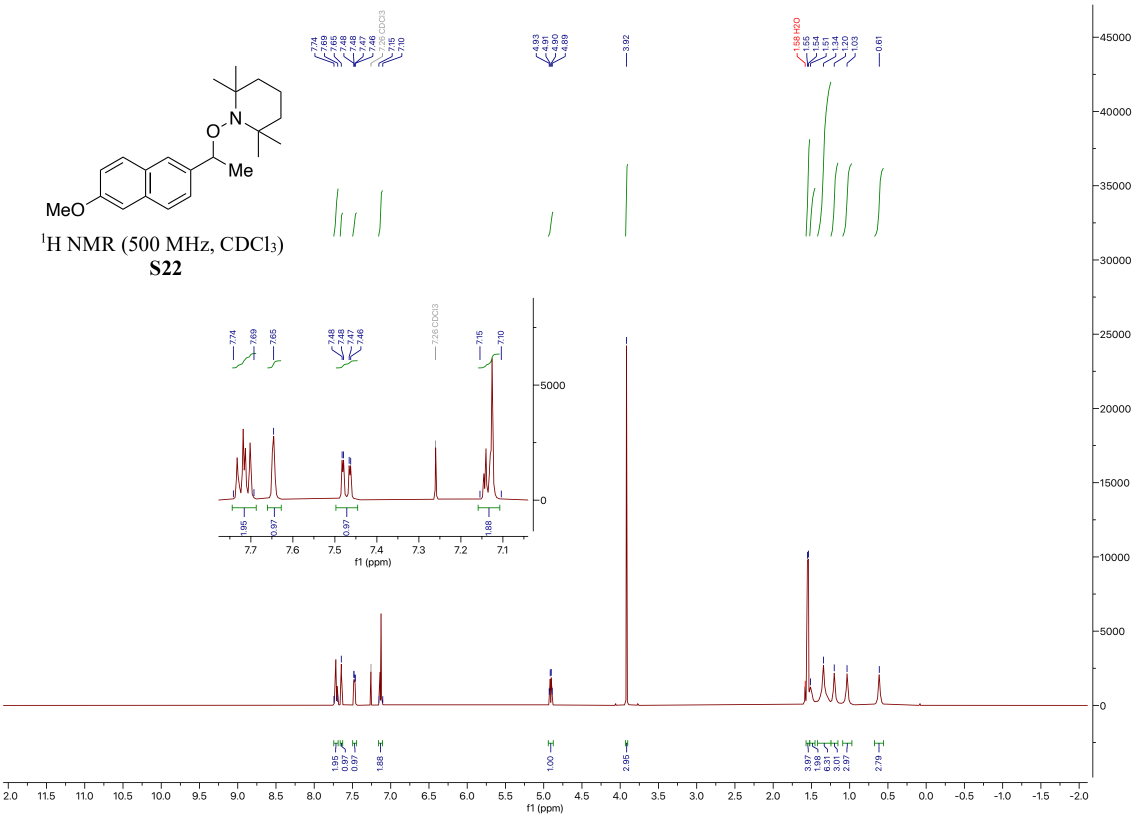

^{1}H NMR (500 MHz, $CDCl_3$)
S22

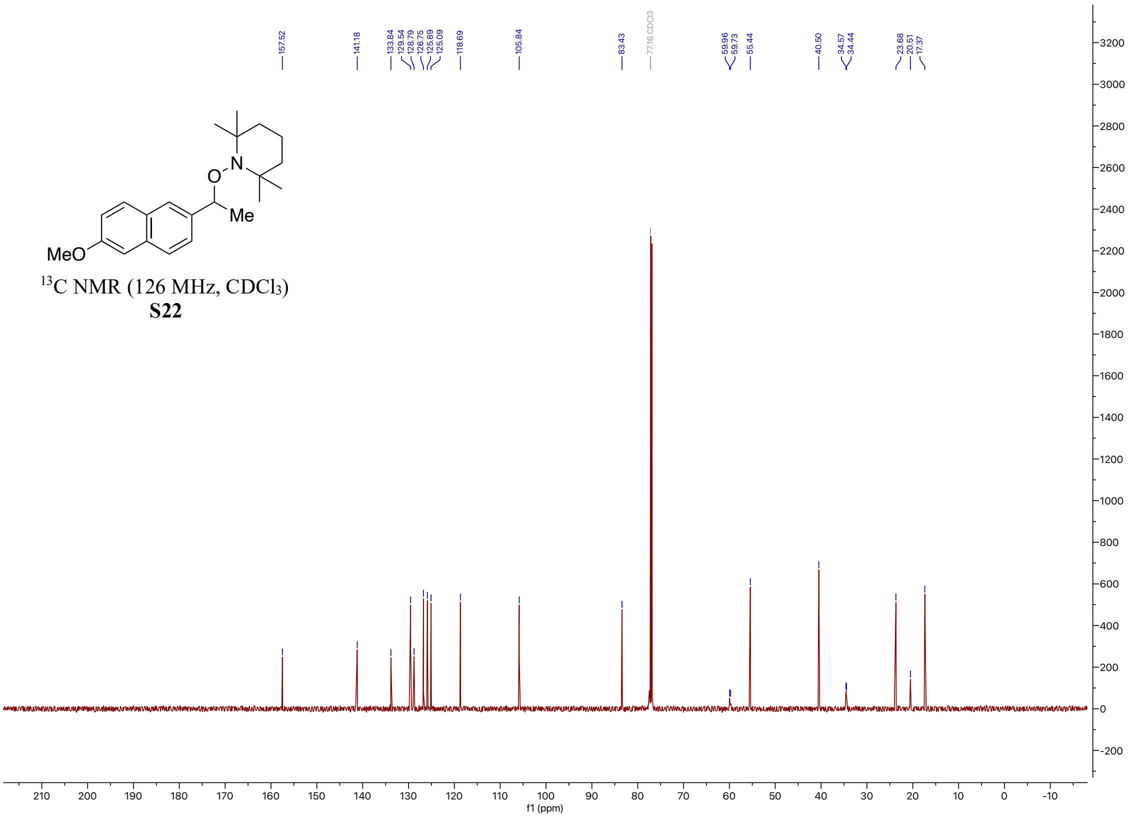

^{13}C NMR (126 MHz, $CDCl_3$)
S22

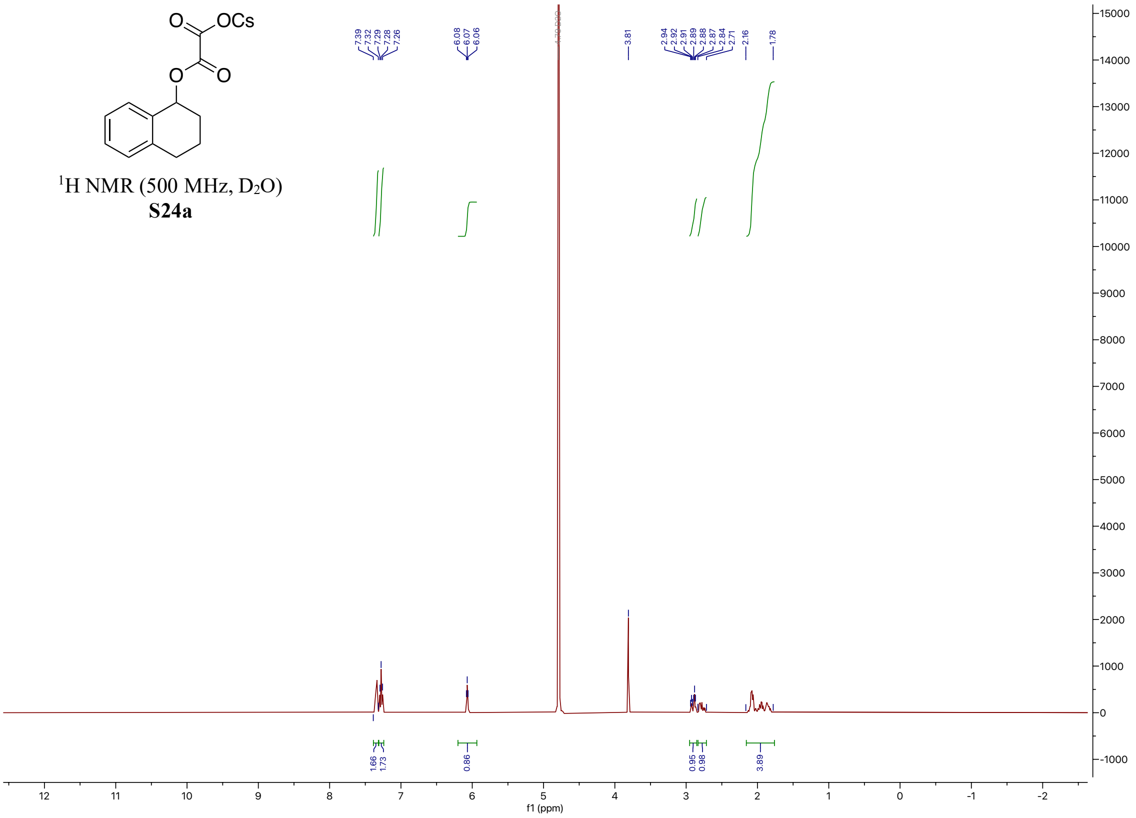

^{1}H NMR (500 MHz, D_2O)

S24a

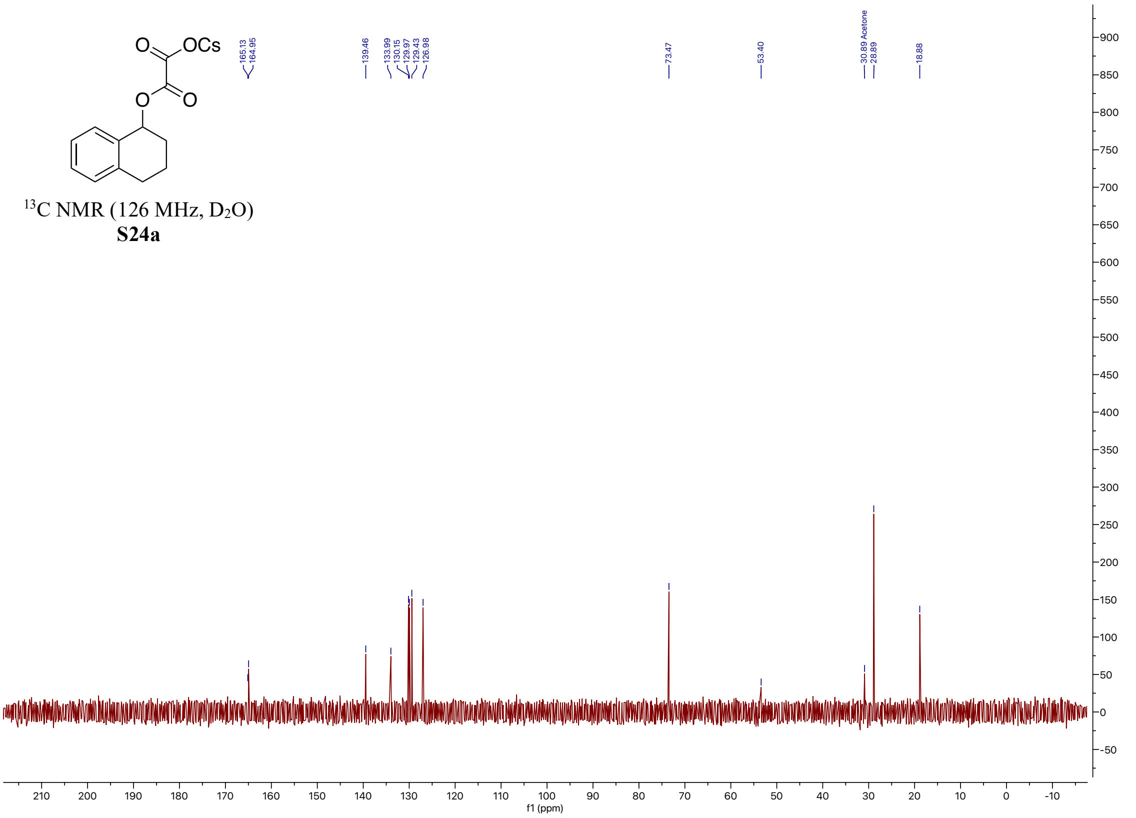

^{13}C NMR (126 MHz, D_2O)

S24a

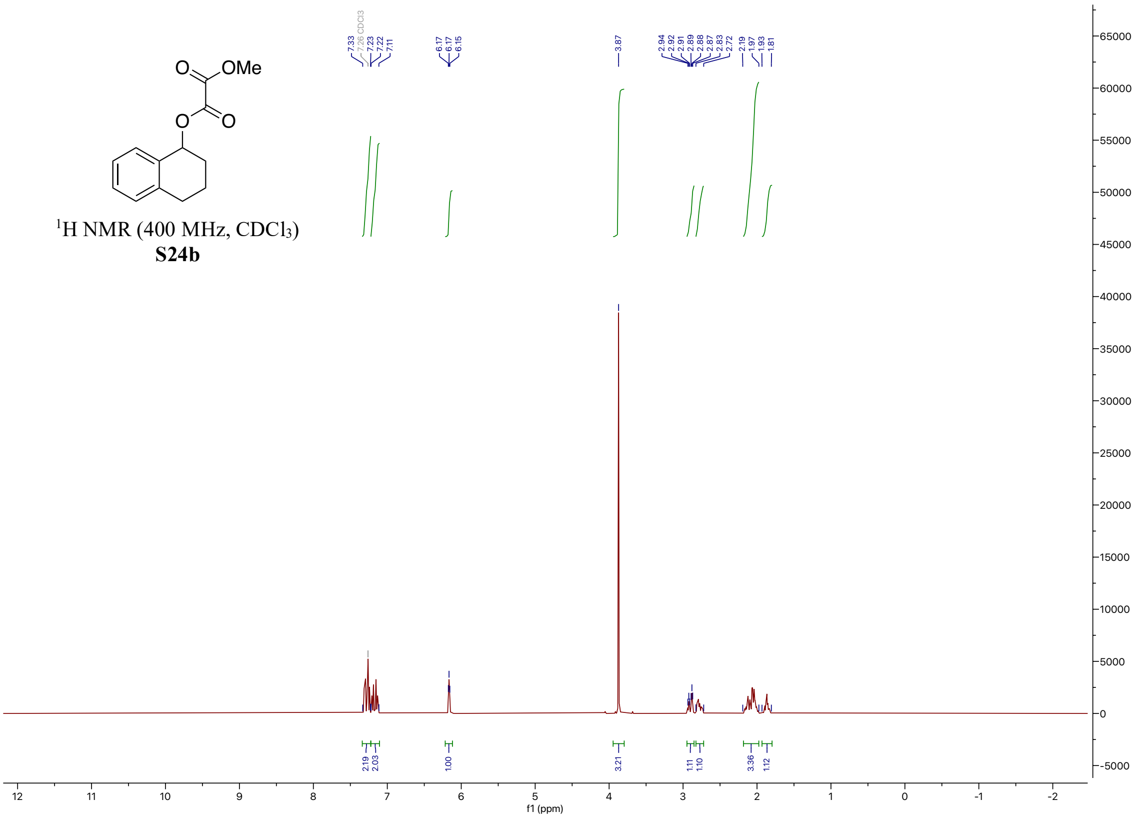

OMe
¹H NMR (400 MHz, CDCl3)
S24b
7.33
7.26 CDCl3
7.23
7.22
7.11
6.17
6.17
6.15
3.87
2.94
2.92
2.91
2.89
2.88
2.87
2.83
2.72
2.19
1.97
1.93
1.81
2.19
2.03
1.00
3.21
1.11
1.10
3.36
1.12
f1 (ppm)

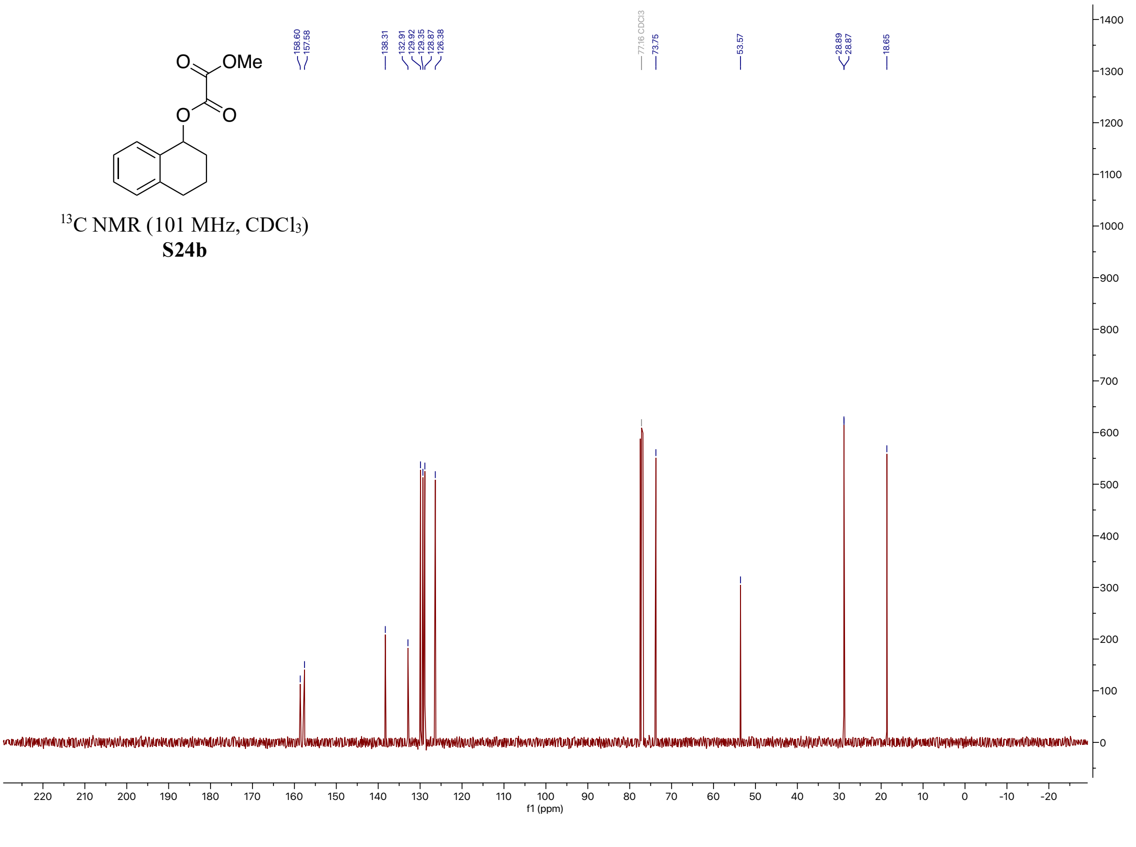

OMe
¹³C NMR (101 MHz, CDCl3)
S24b
158.60
157.58
138.31
132.91
129.92
129.35
128.87
126.38
77.16 CDCl3
73.75
53.57
28.89
28.87
18.65
f1 (ppm)

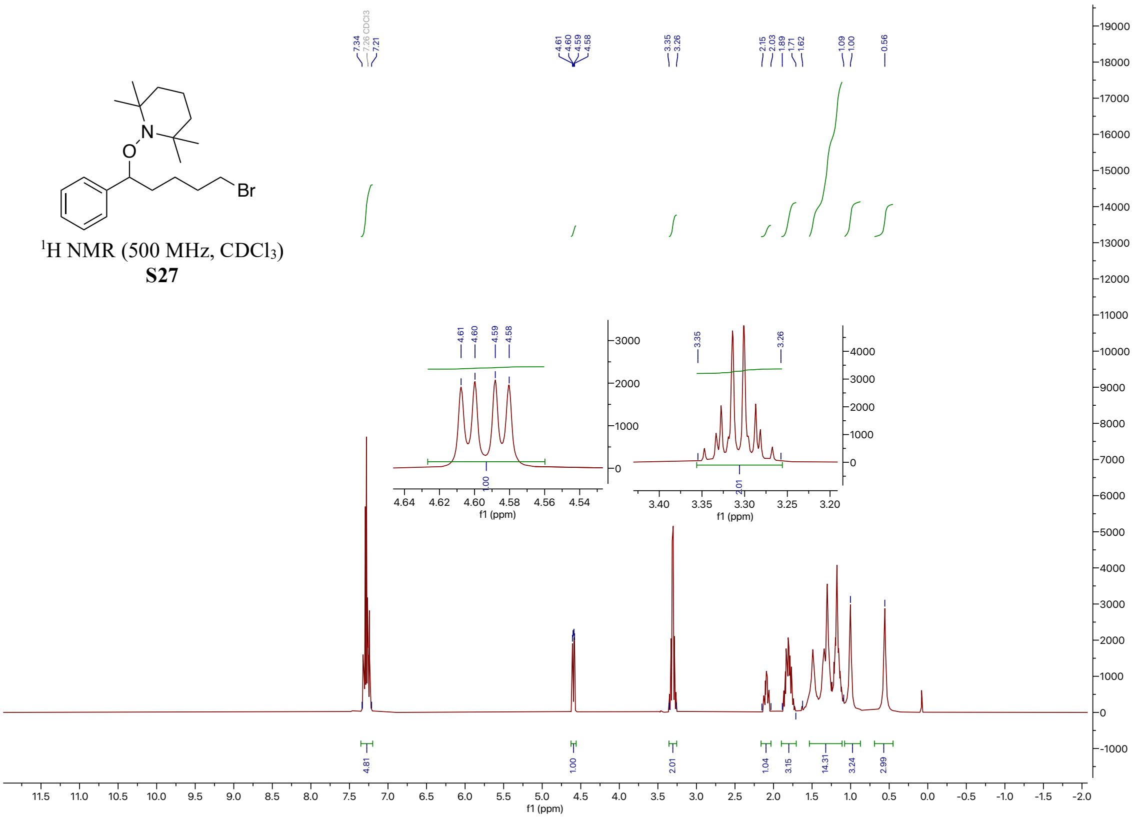

^{1}H NMR (500 MHz, $CDCl_3$)
S27

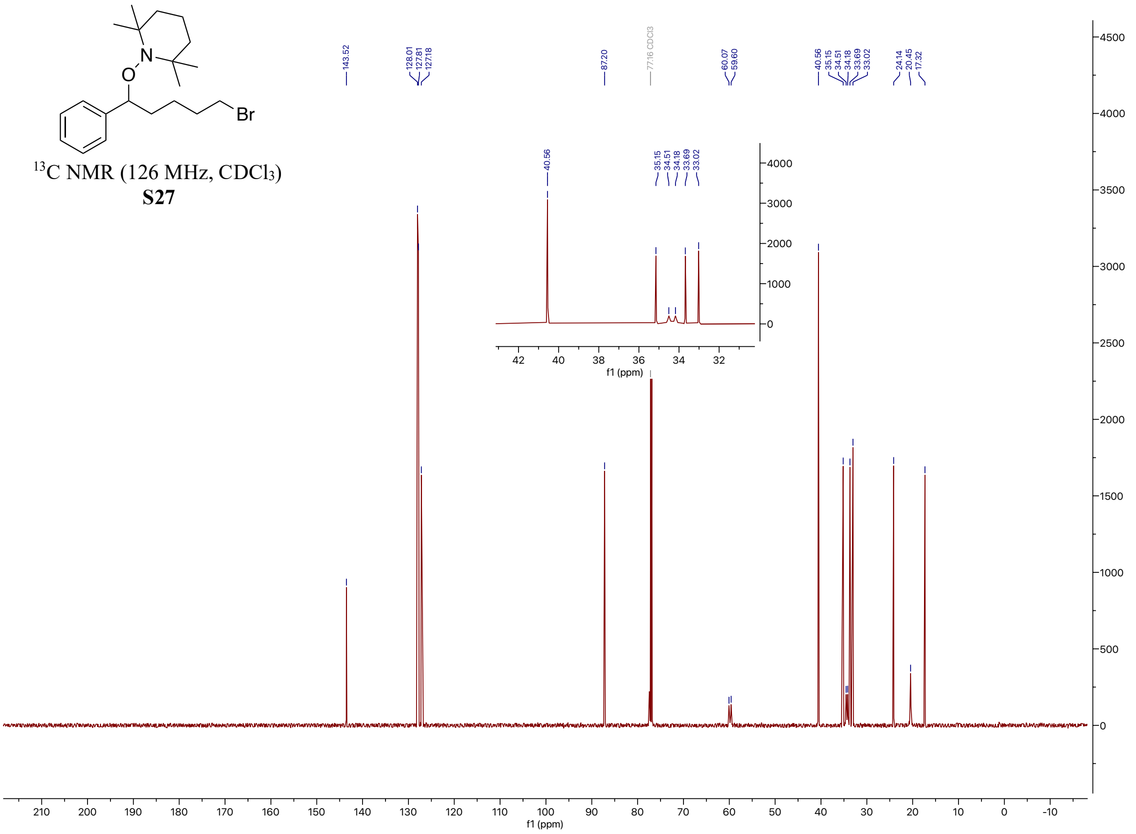

^{13}C NMR (126 MHz, $CDCl_3$)
S27

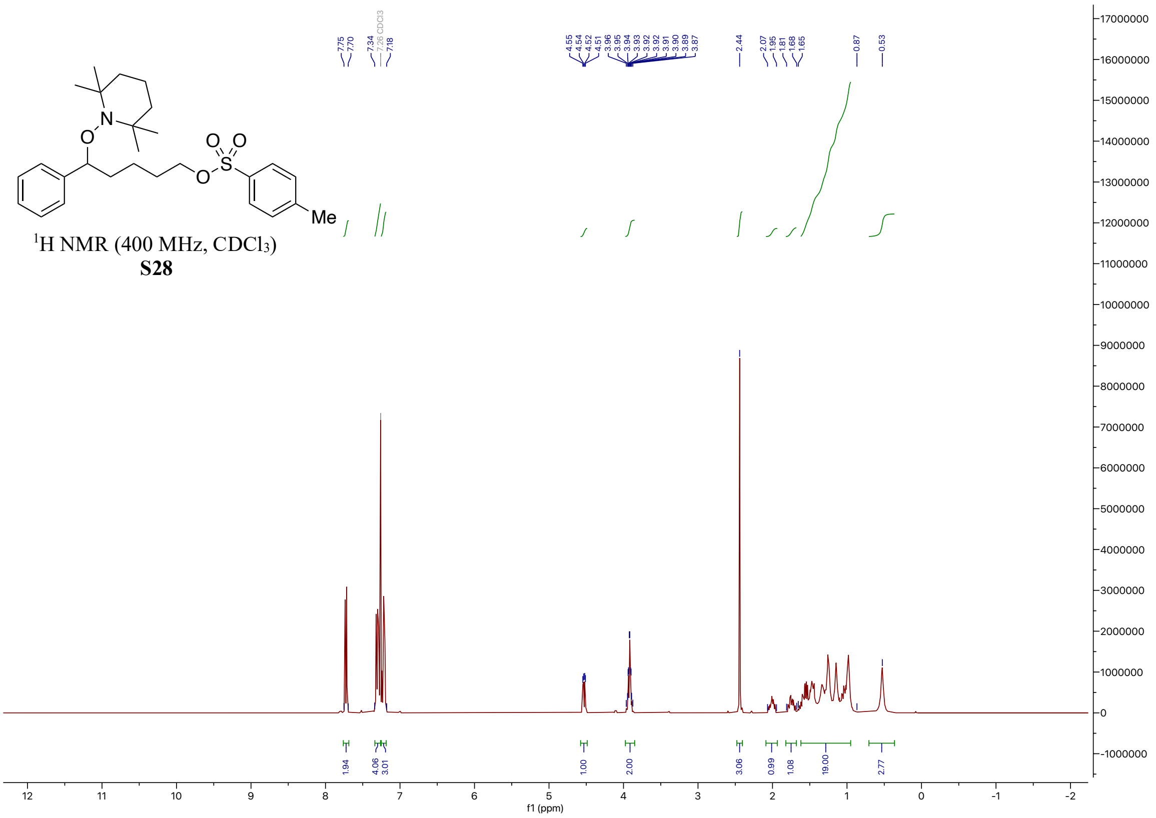

Me
^{1}H NMR (400 MHz, $CDCl_3$)
S28
f1 (ppm)

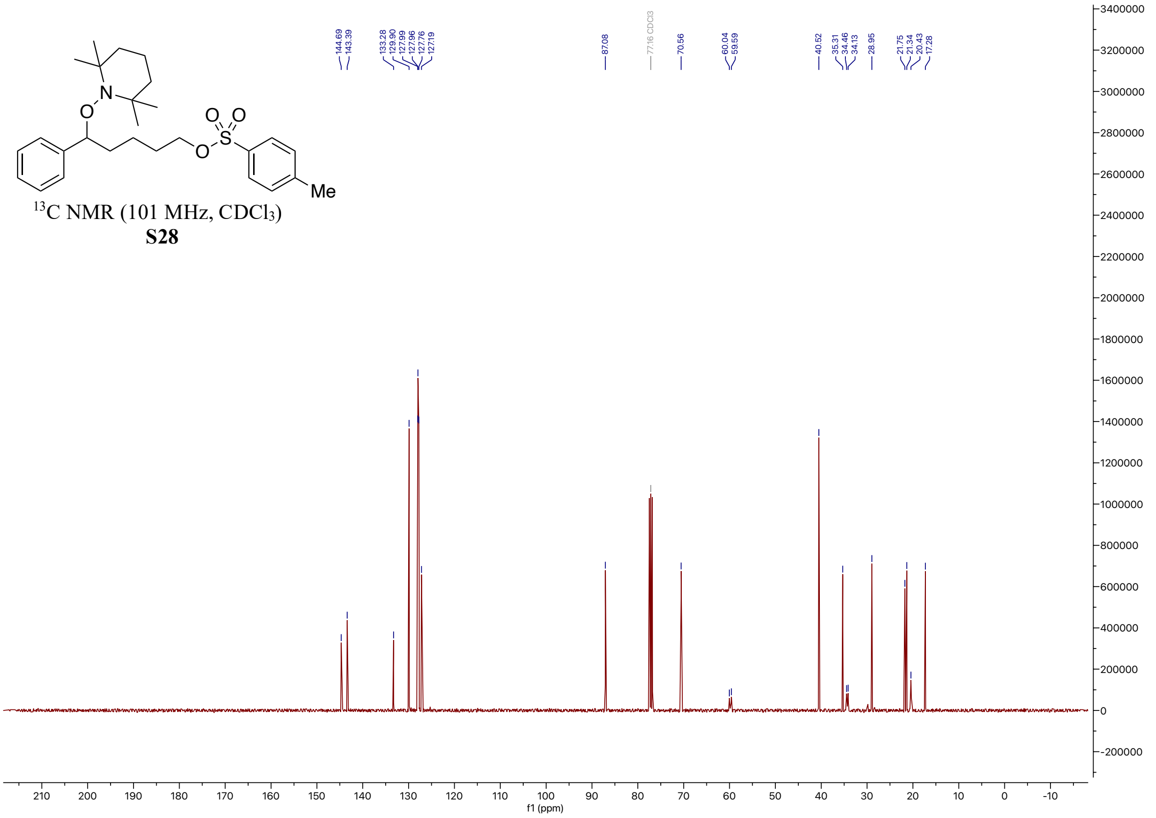

Me
^{13}C NMR (101 MHz, $CDCl_3$)
S28
f1 (ppm)

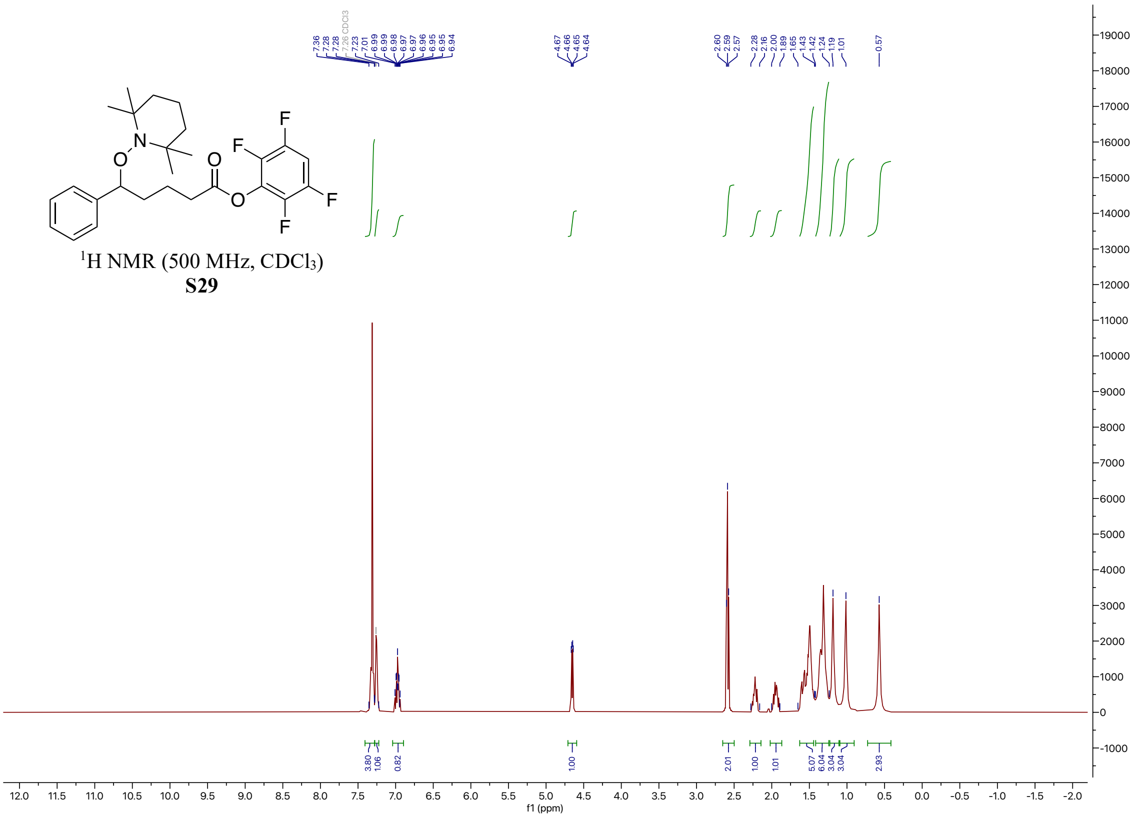

¹H NMR (500 MHz, CDCl₃)
S29
f1 (ppm)

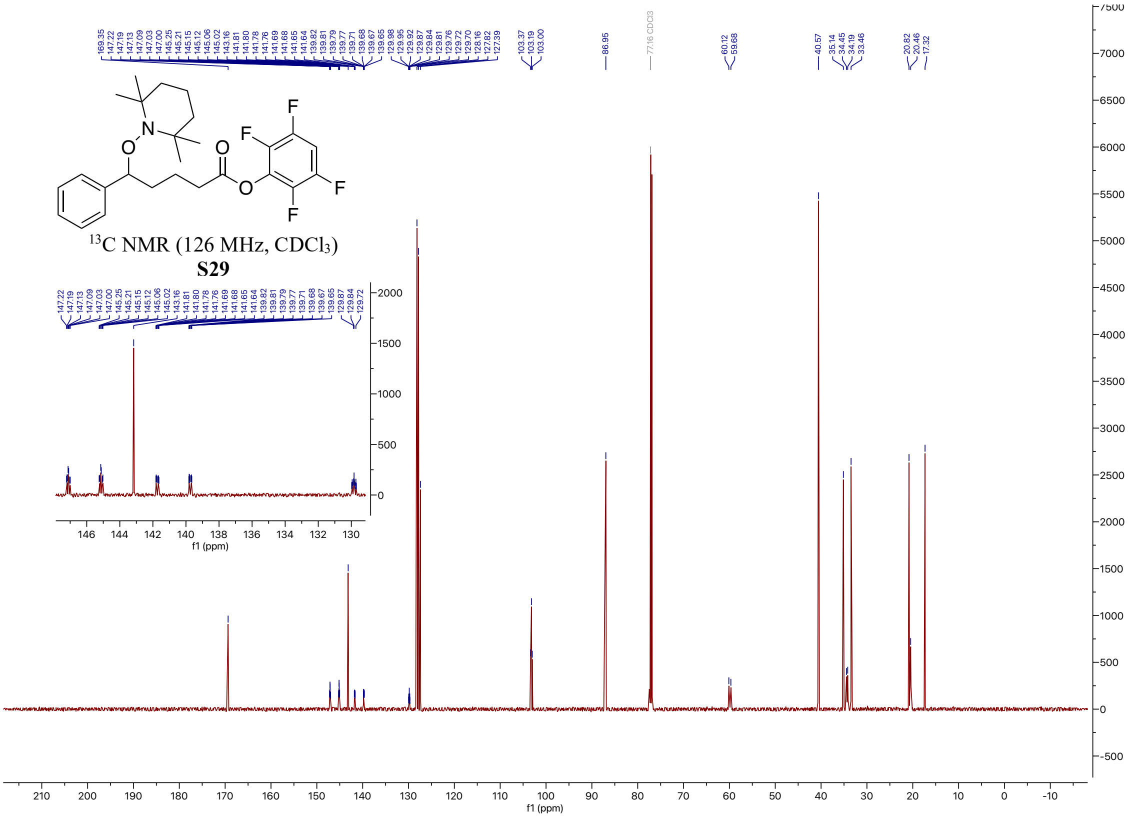

¹³C NMR (126 MHz, CDCl₃)
S29
f1 (ppm)

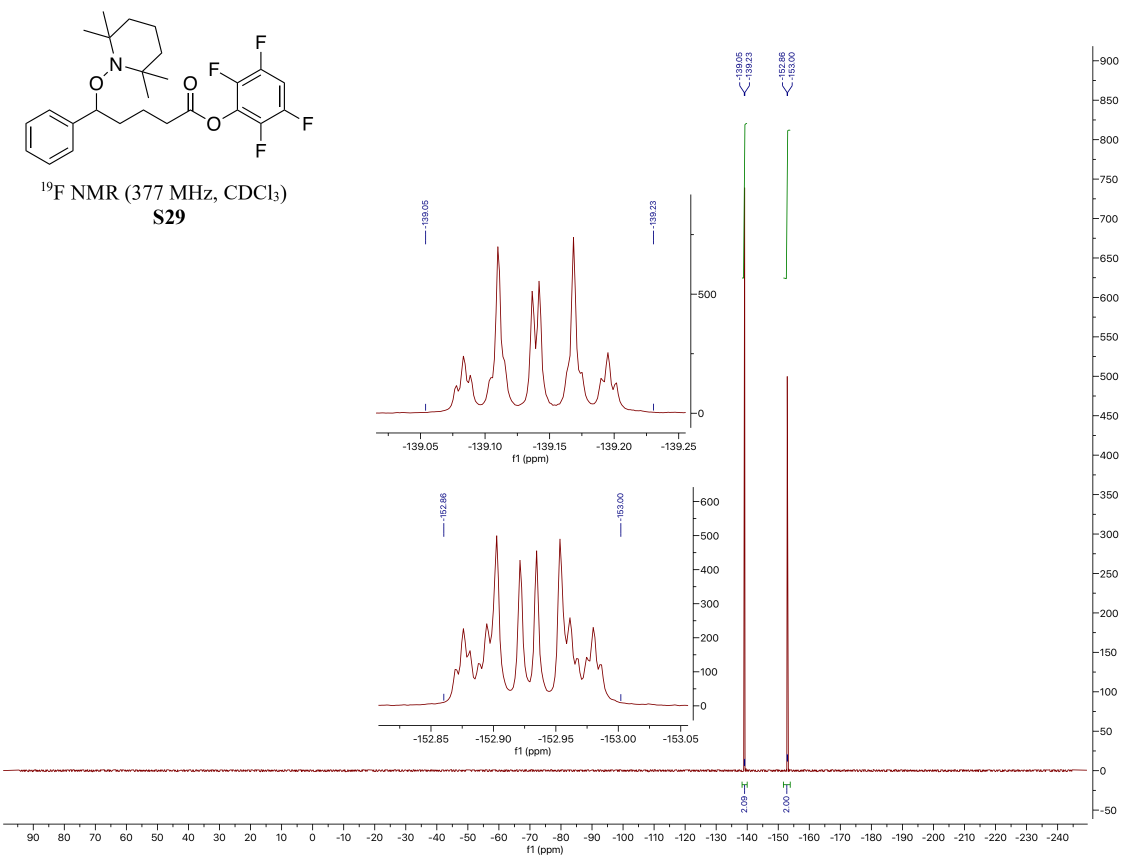

^{19}F NMR (377 MHz, $CDCl_3$)
S29

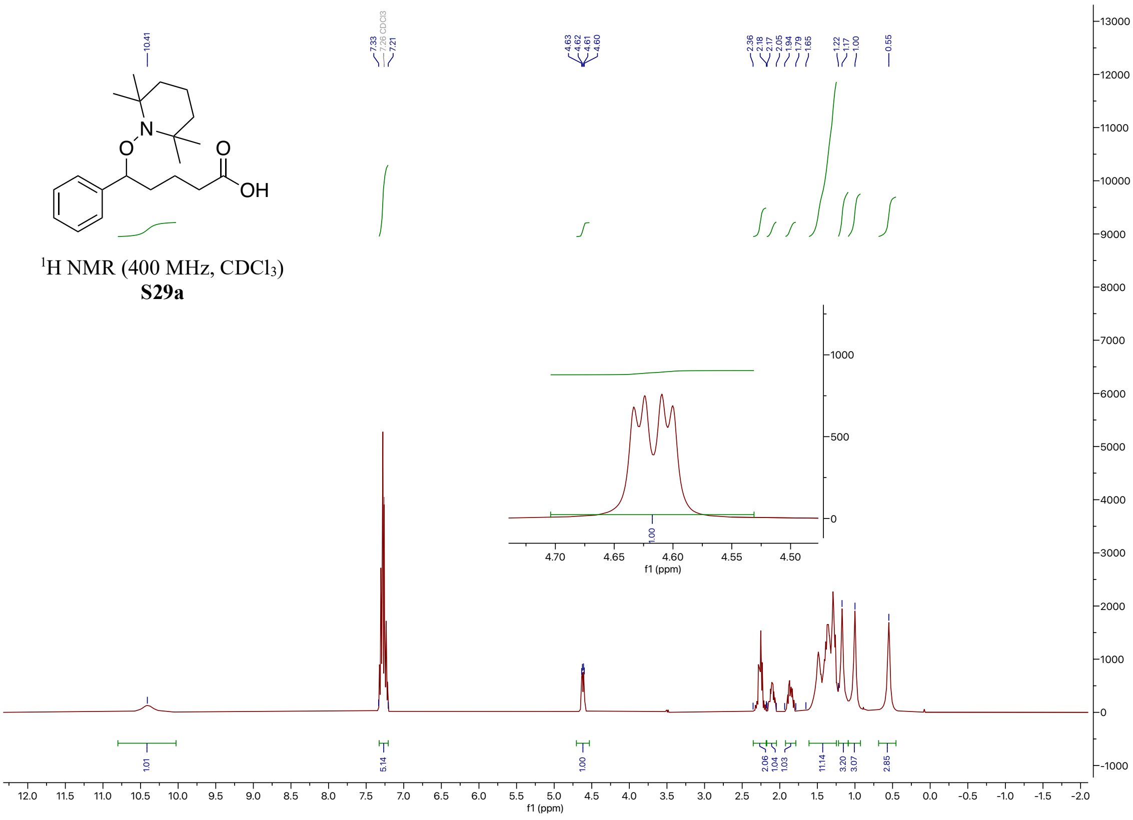

^{1}H NMR (400 MHz, $CDCl_3$)
S29a

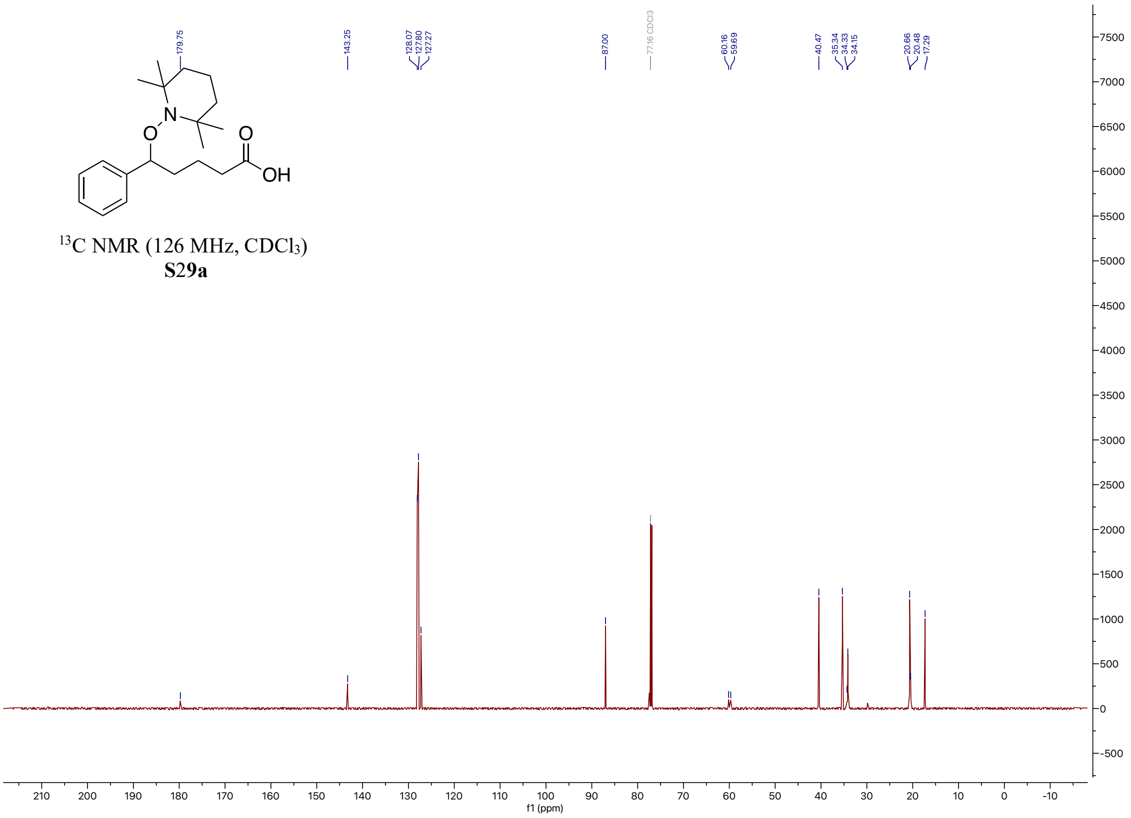

^{13}C NMR (126 MHz, $CDCl_3$)
S29a

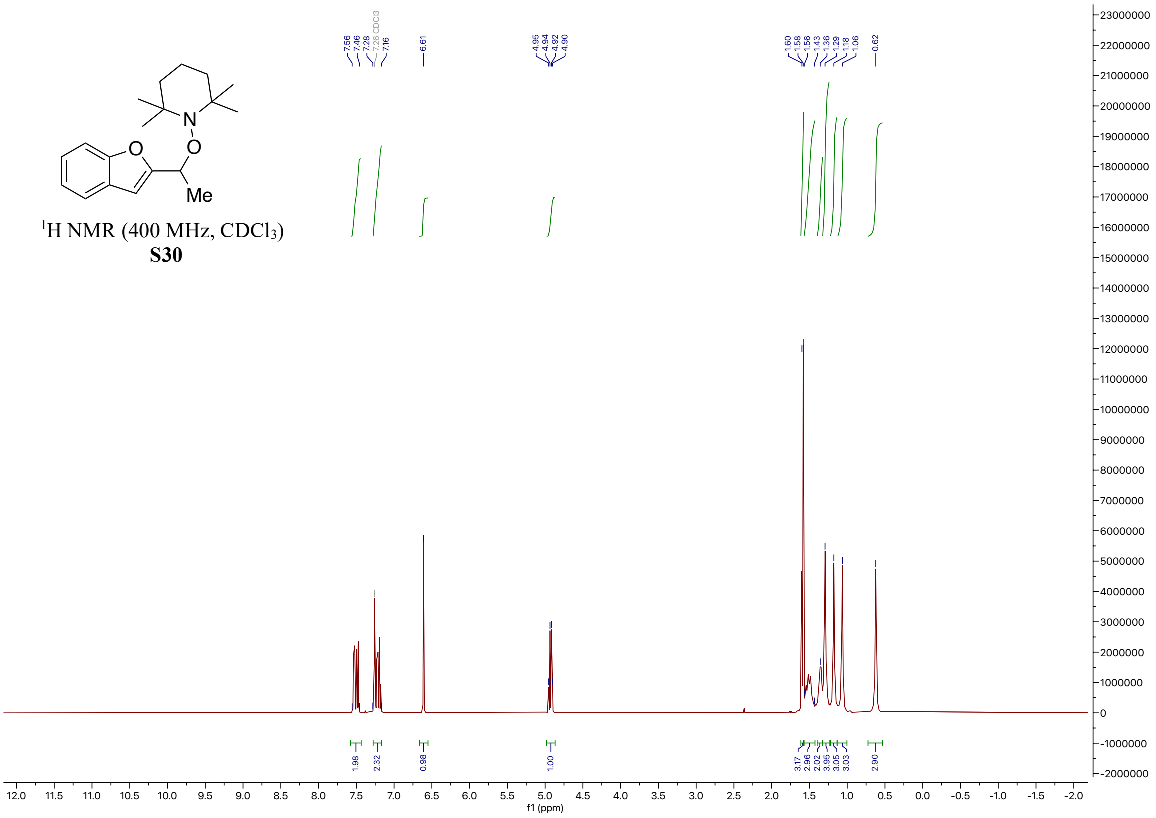

^{1}H NMR (400 MHz, $CDCl_3$)
S30

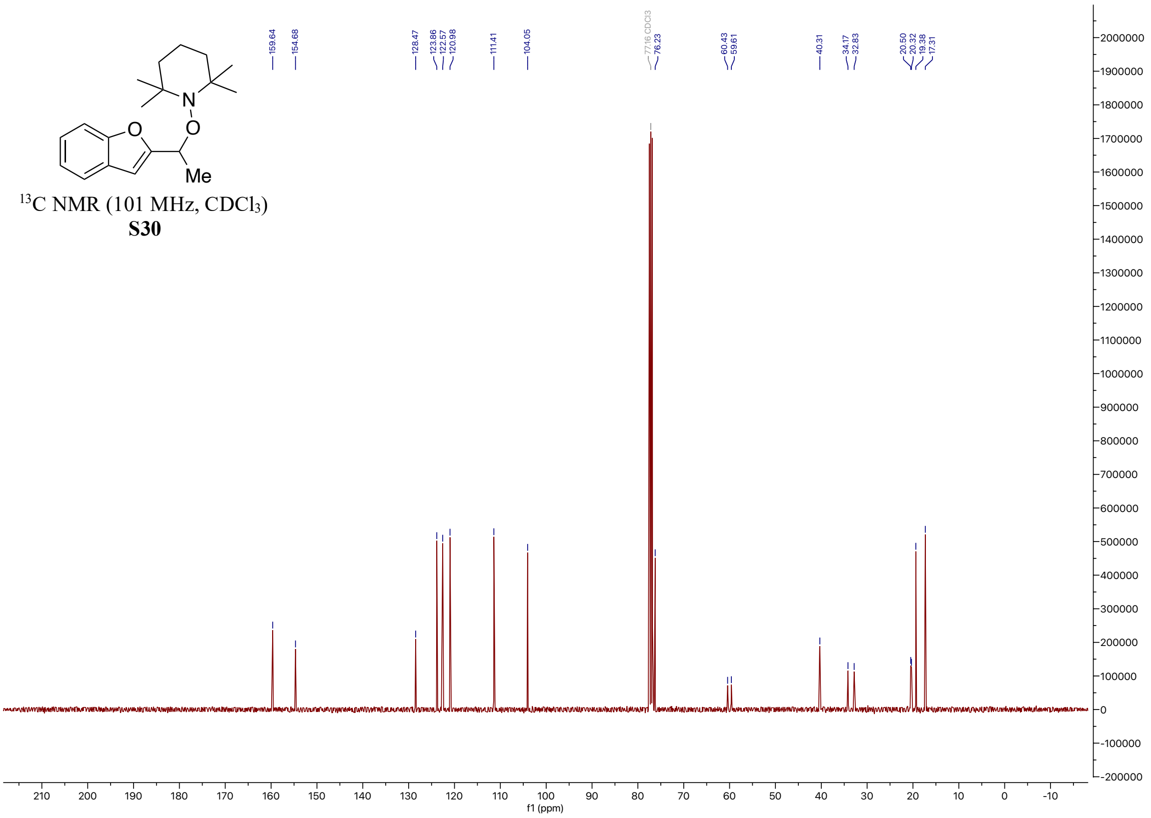

^{13}C NMR (101 MHz, $CDCl_3$)

S30

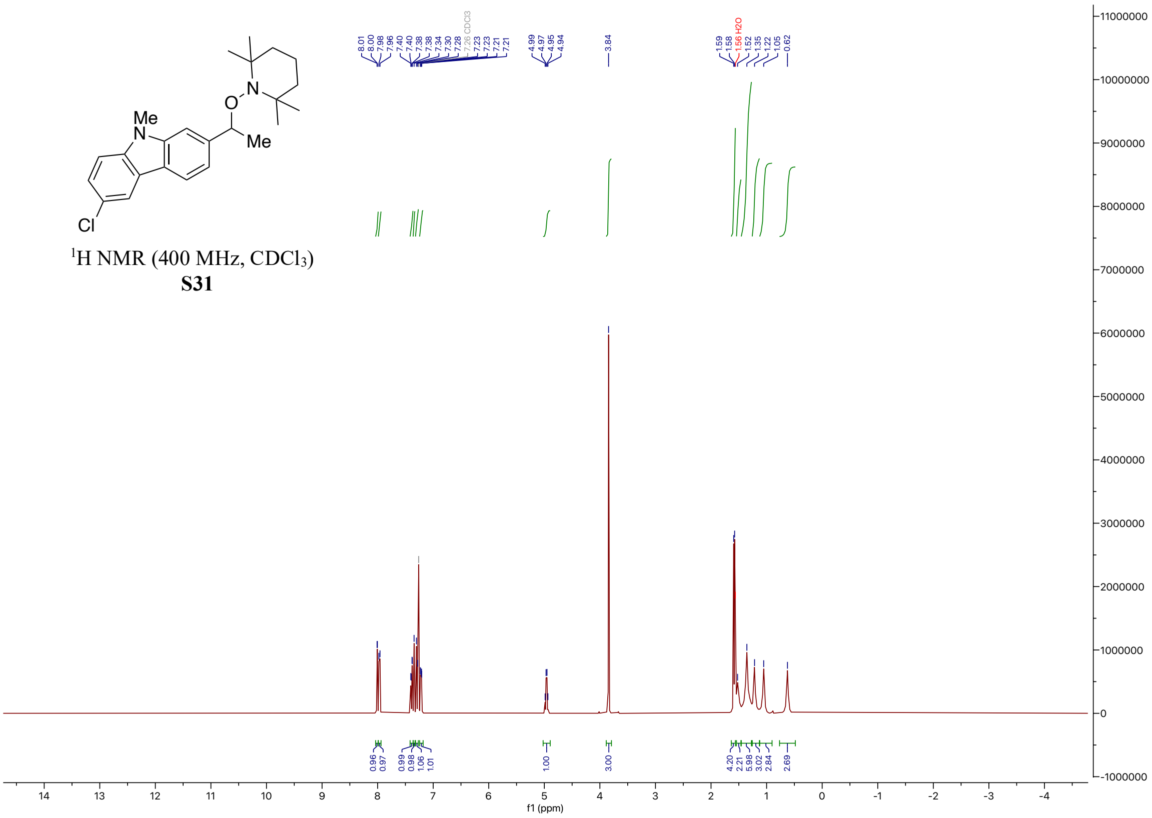

^{1}H NMR (400 MHz, $CDCl_3$)

S31

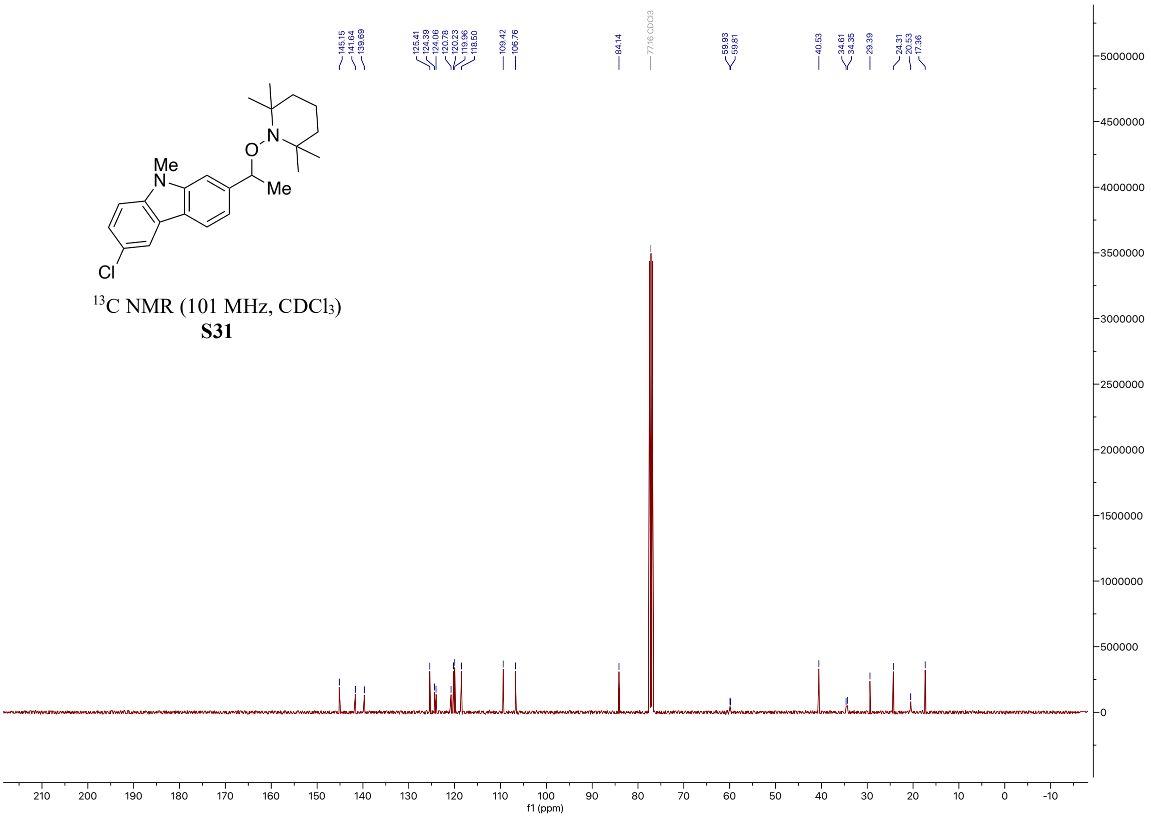
145.15
141.64
139.69
125.41
124.39
124.06
120.78
120.23
119.96
118.50
109.42
106.76
84.14
77.16 CDCl3
59.93
59.81
40.53
34.61
34.35
29.39
24.31
20.53
17.36
Me
N
O
N
Me
Cl
^{13}C NMR (101 MHz, $CDCl_3$)
S31
f1 (ppm)

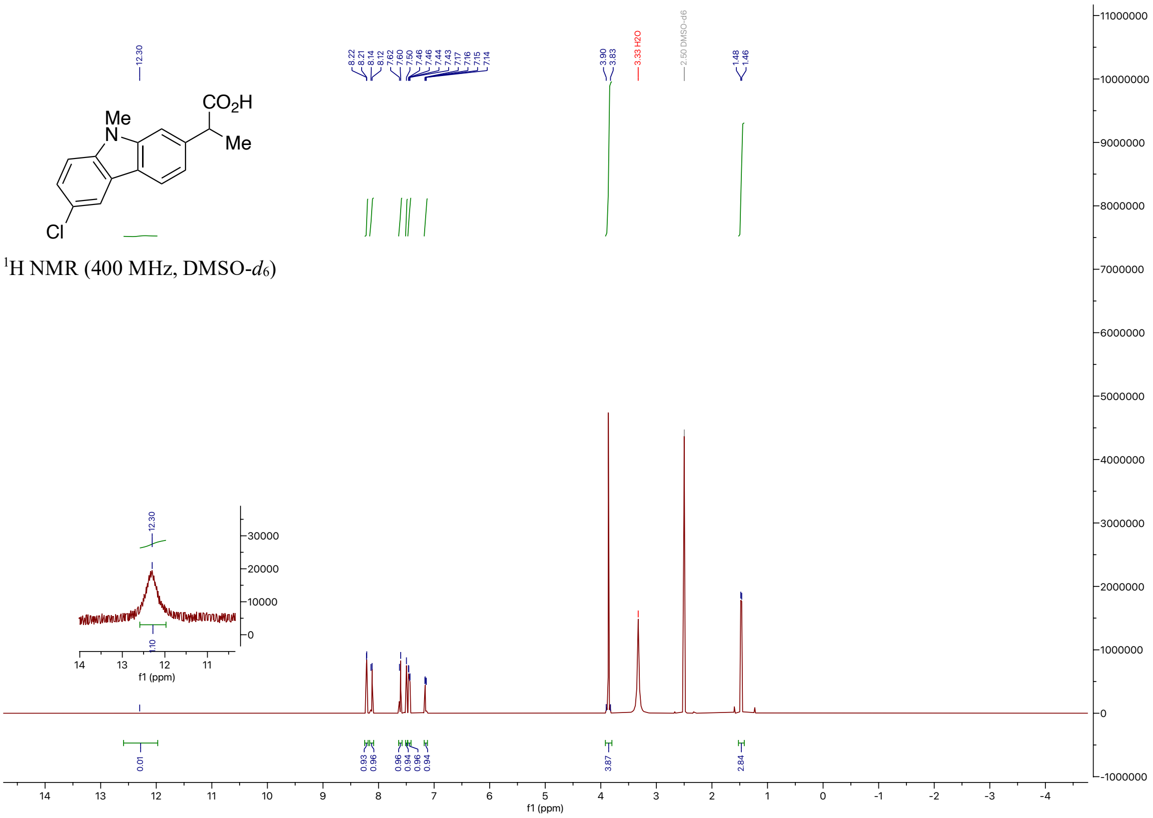
12.30
8.22
8.21
8.14
8.12
7.62
7.60
7.50
7.46
7.46
7.44
7.43
7.17
7.16
7.15
7.14
3.90
3.83
3.33 H2O
2.50 DMSO-d6
1.48
1.46
CO_2H
Me
N
Me
Cl
^{1}H NMR (400 MHz, DMSO-d_6)
1.10
0.01
0.93
0.96
0.96
0.94
0.96
0.94
3.87
2.84
f1 (ppm)

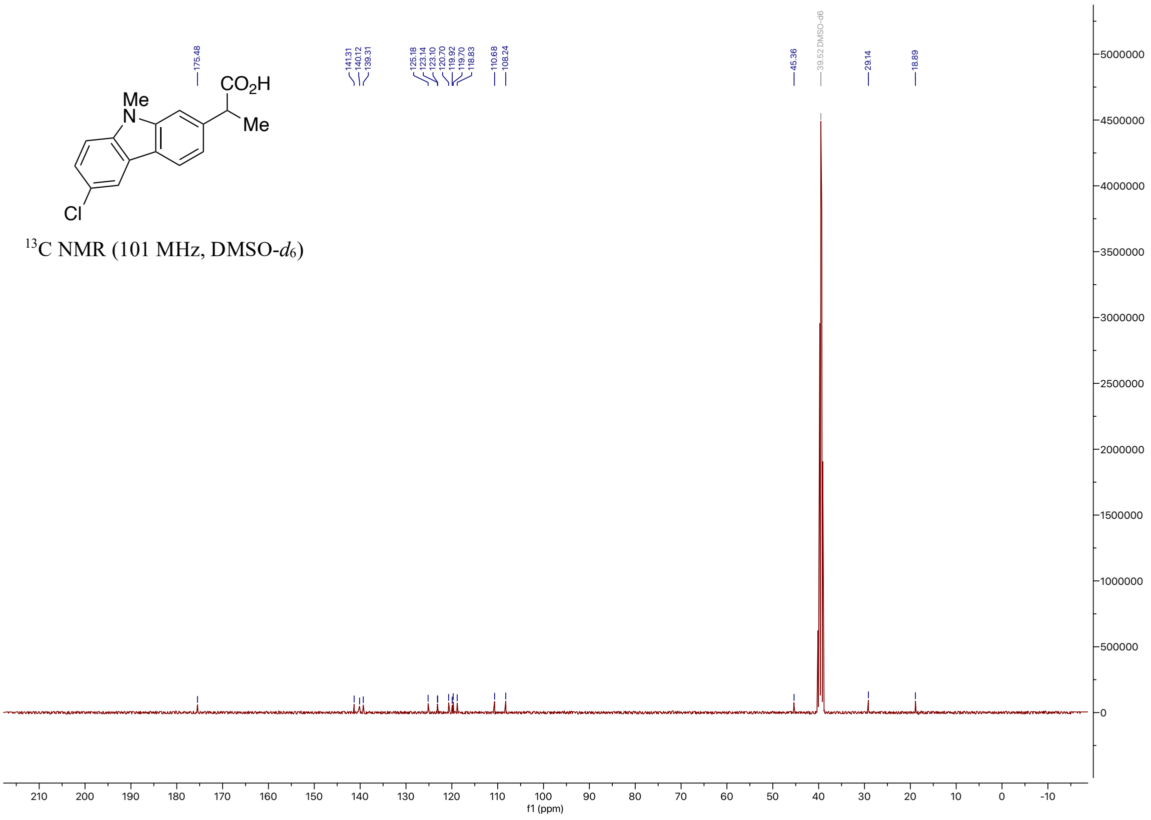
CO2H
Me
N
Me
Cl
13C NMR (101 MHz, DMSO-d6)
175.48
141.31
140.12
139.31
125.18
123.14
123.10
120.70
119.92
119.70
118.83
110.68
108.24
45.36
39.52 DMSO-d6
29.14
18.89
f1 (ppm)

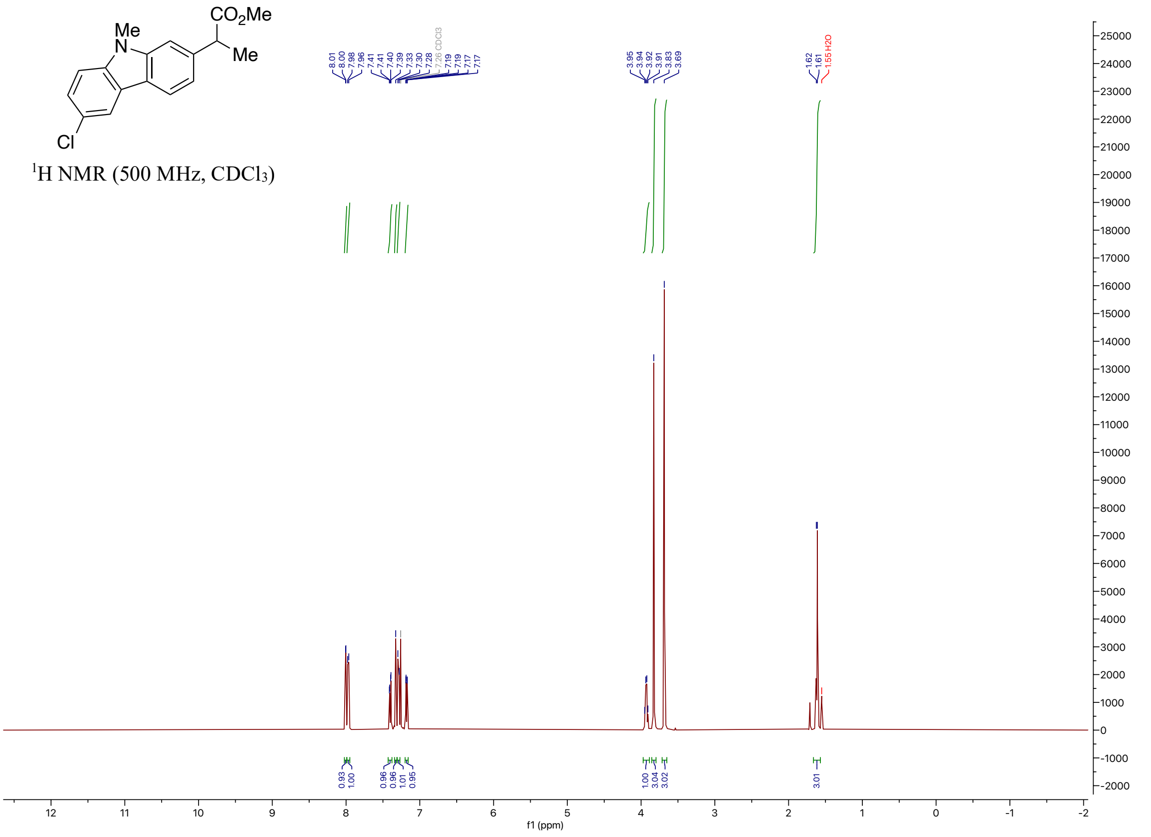
CO2Me
Me
N
Me
Cl
1H NMR (500 MHz, CDCl3)
8.01
8.00
7.98
7.96
7.41
7.40
7.39
7.33
7.30
7.28
7.26 CDCl3
7.19
7.19
7.17
7.17
3.95
3.94
3.92
3.91
3.83
3.69
1.62
1.61
1.55 H2O
0.93
1.00
0.96
0.96
1.01
0.95
1.00
3.04
3.02
3.01
f1 (ppm)

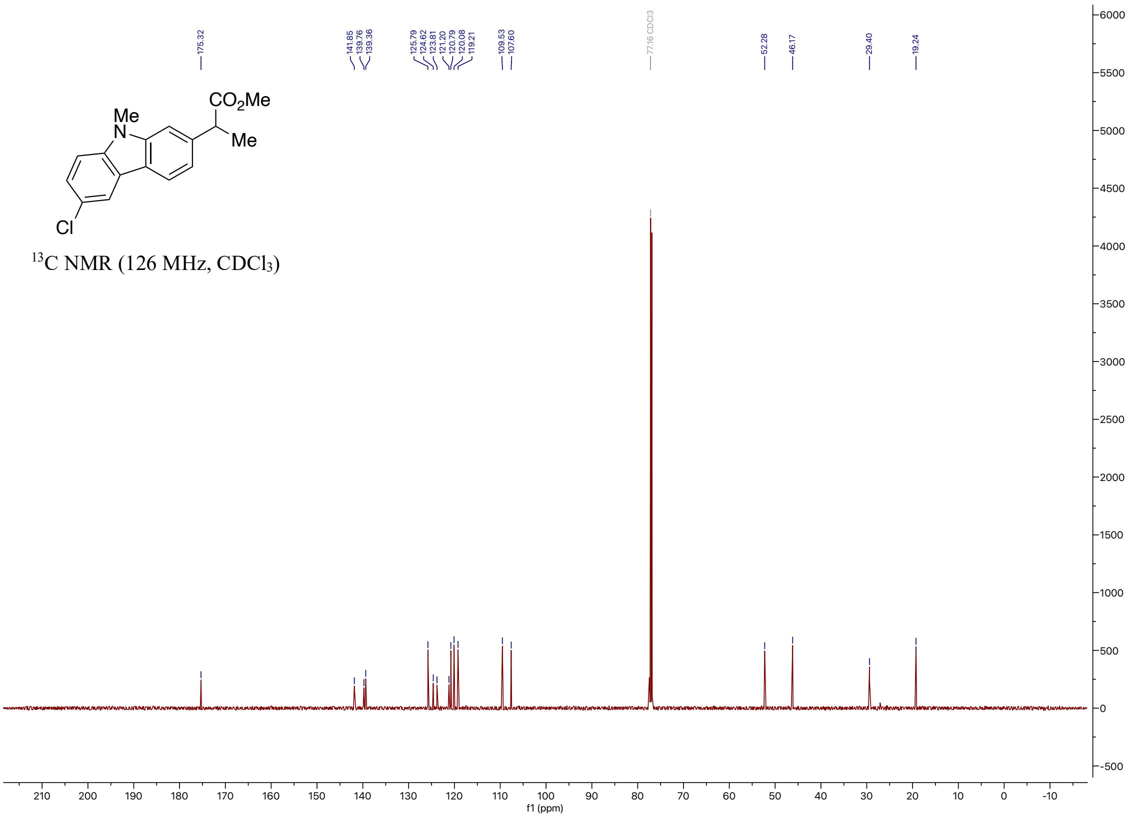

^{13}C NMR (126 MHz, $CDCl_3$)

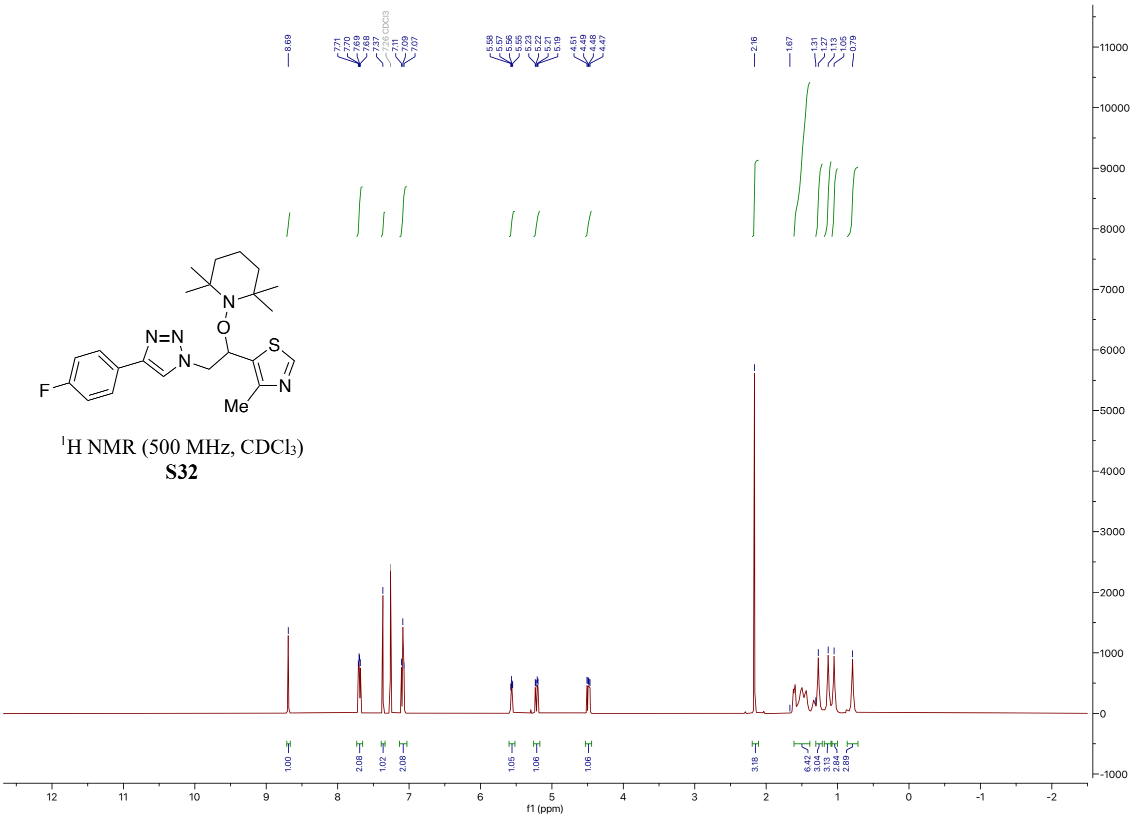

^{1}H NMR (500 MHz, $CDCl_3$)

S32

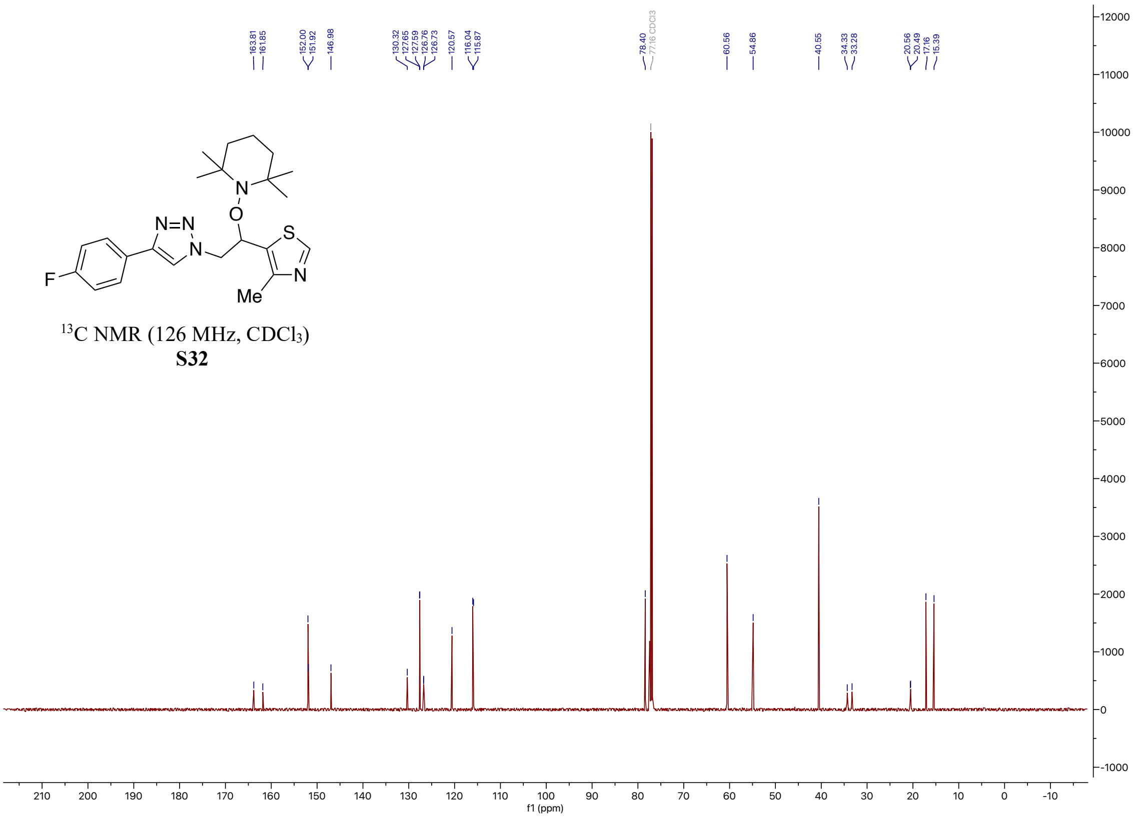

^{13}C NMR (126 MHz, $CDCl_3$)
S32

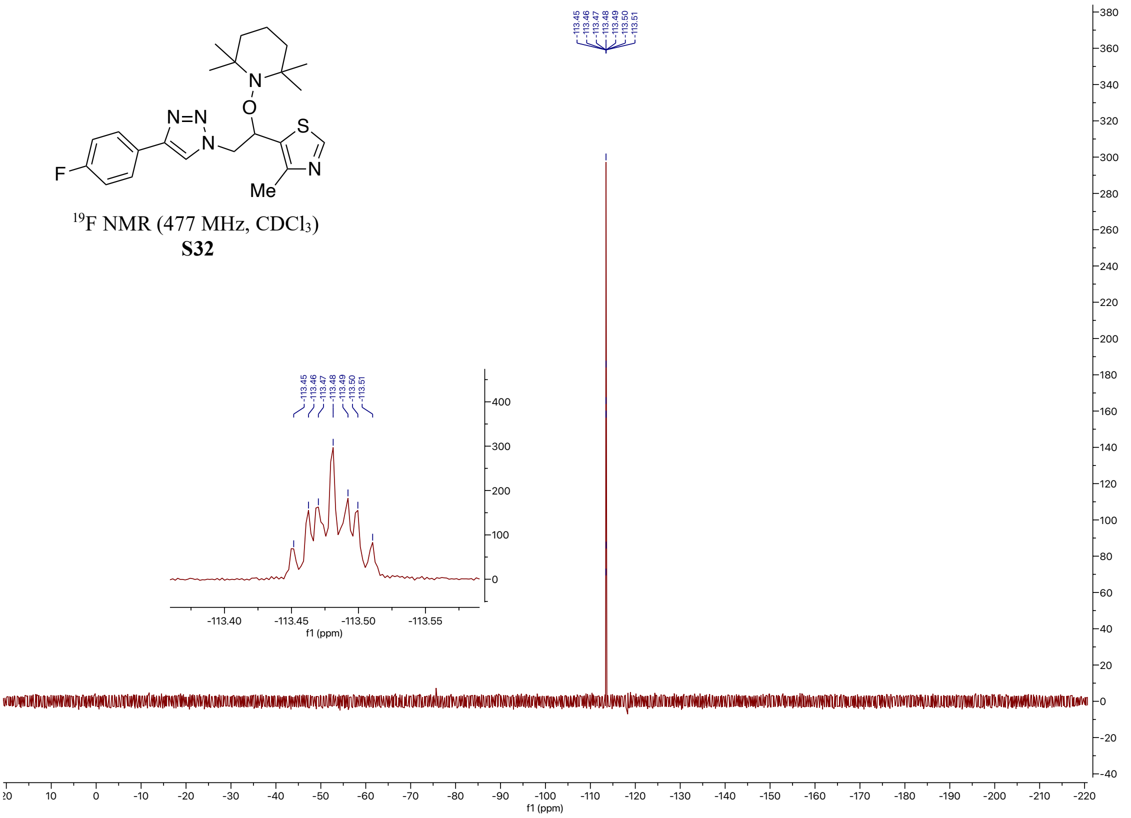

^{19}F NMR (477 MHz, $CDCl_3$)
S32

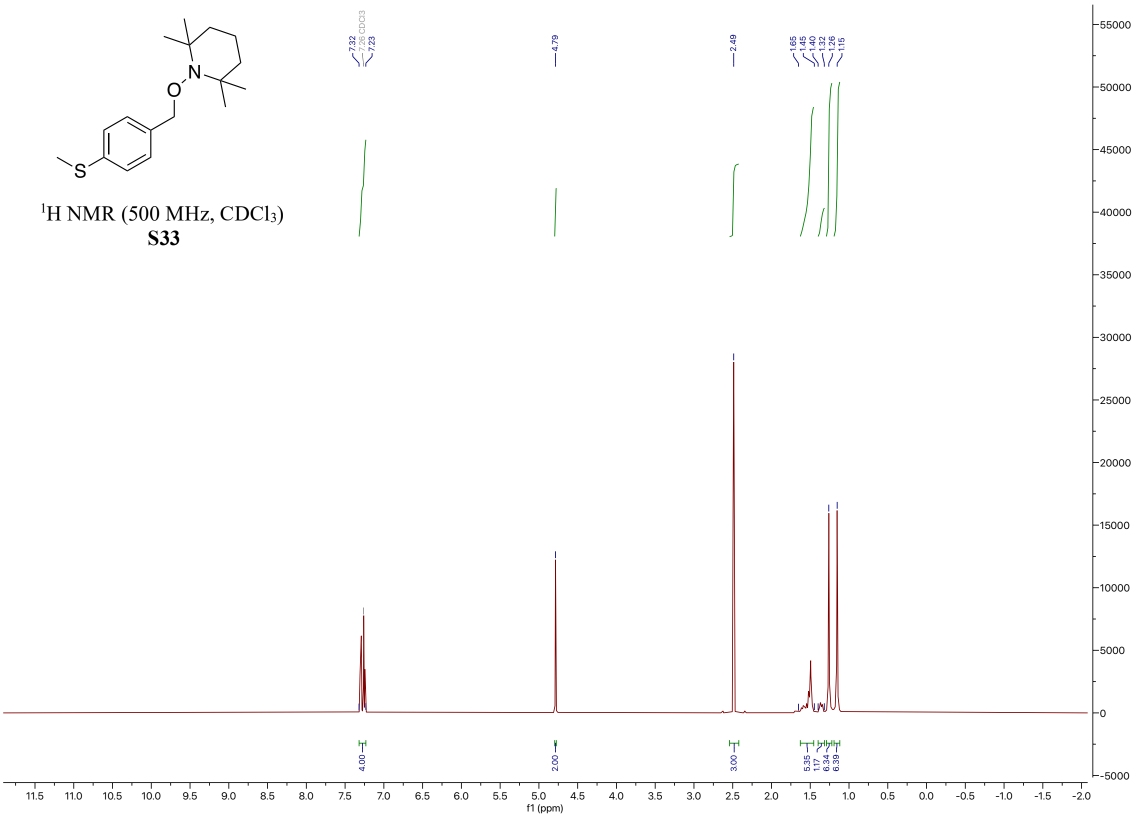

1H NMR (500 MHz, CDCl3)
S33
7.32
7.26 CDCl3
7.23
4.79
2.49
1.65
1.45
1.40
1.32
1.26
1.15
4.00
2.00
3.00
5.35
1.17
6.34
6.39
f1 (ppm)

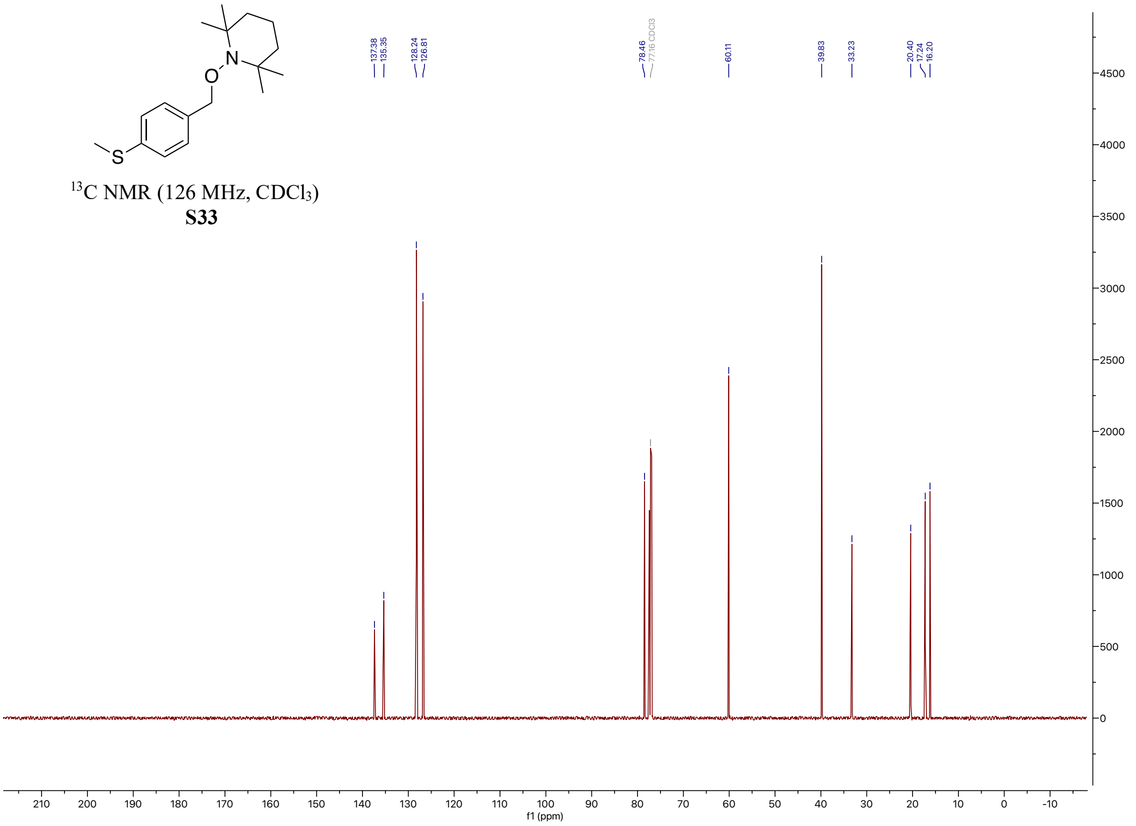

13C NMR (126 MHz, CDCl3)
S33
137.38
135.35
128.24
126.81
78.46
77.16 CDCl3
60.11
39.83
33.23
20.40
17.24
16.20
f1 (ppm)

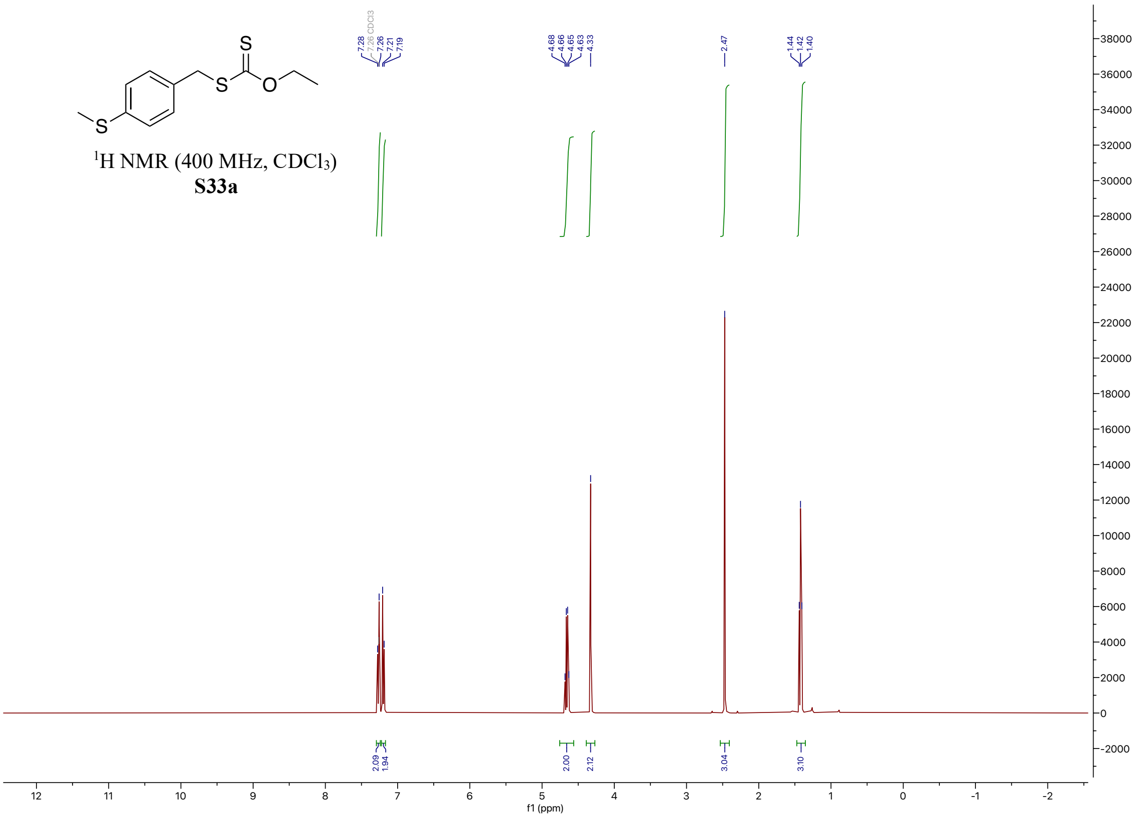

^1H NMR (400 MHz, $CDCl_3$)
S33a

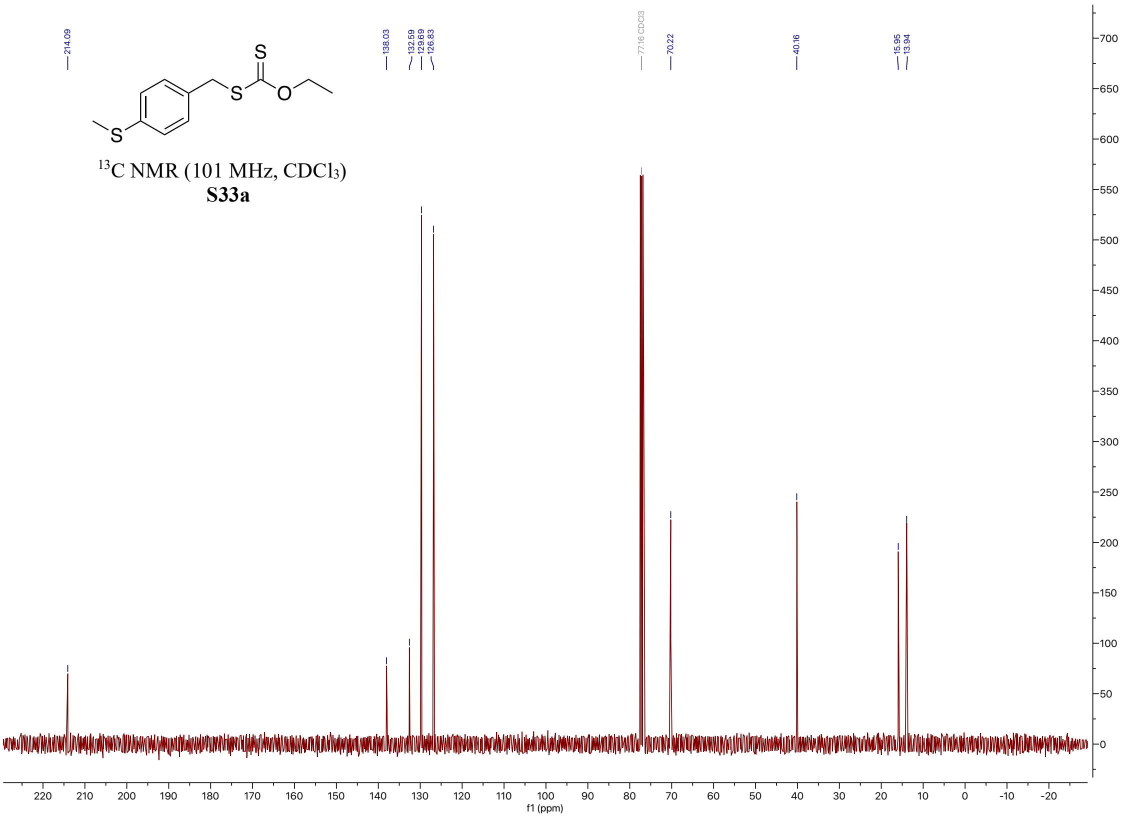

^{13}C NMR (101 MHz, $CDCl_3$)
S33a

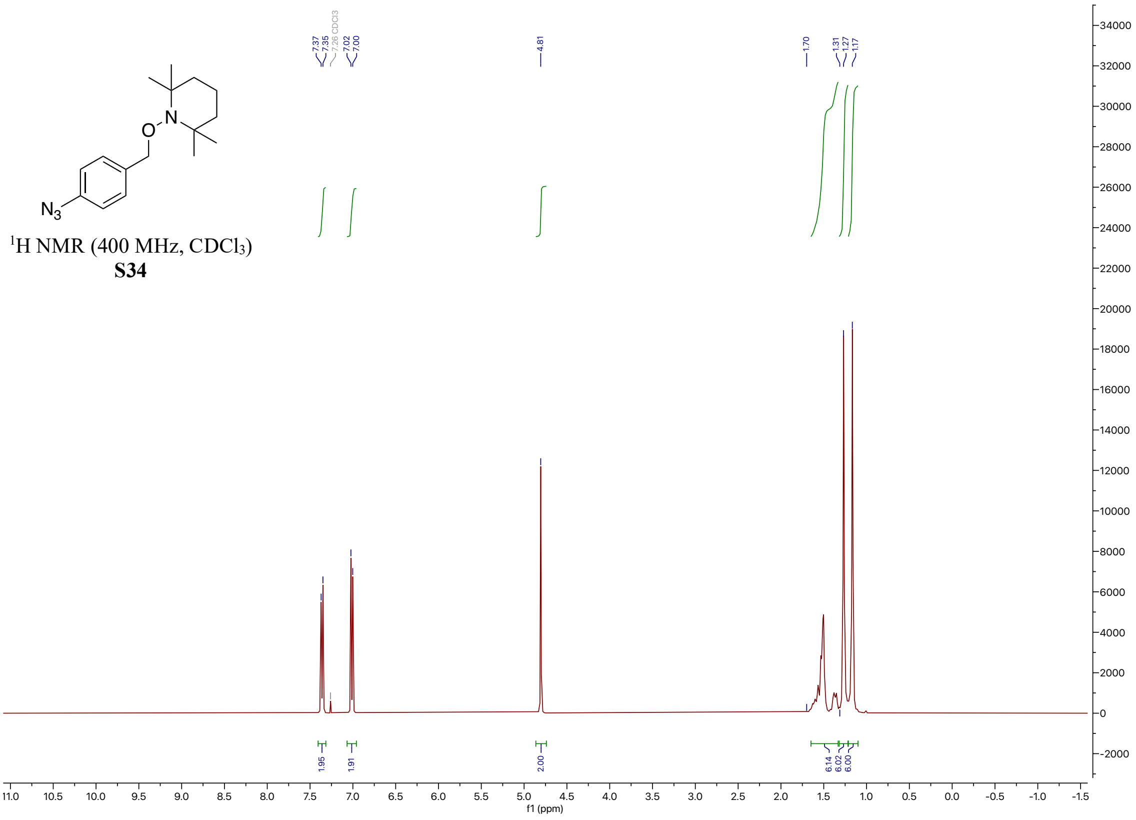

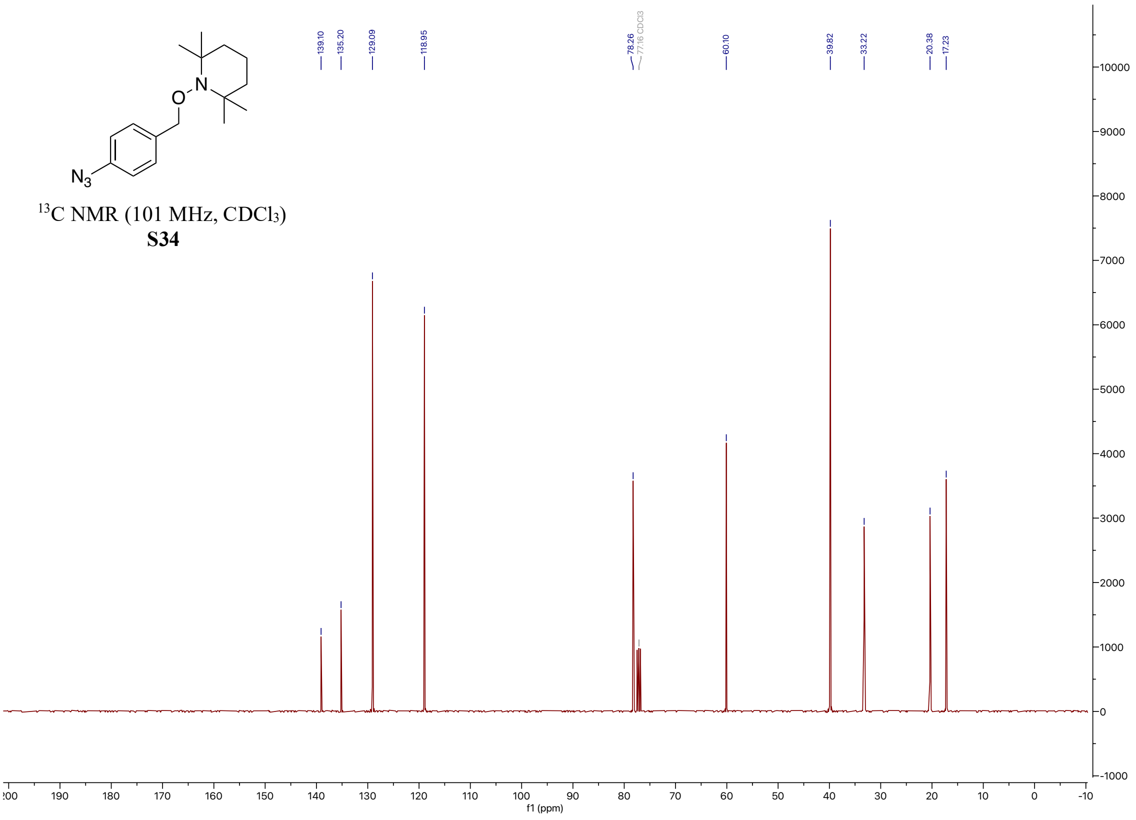

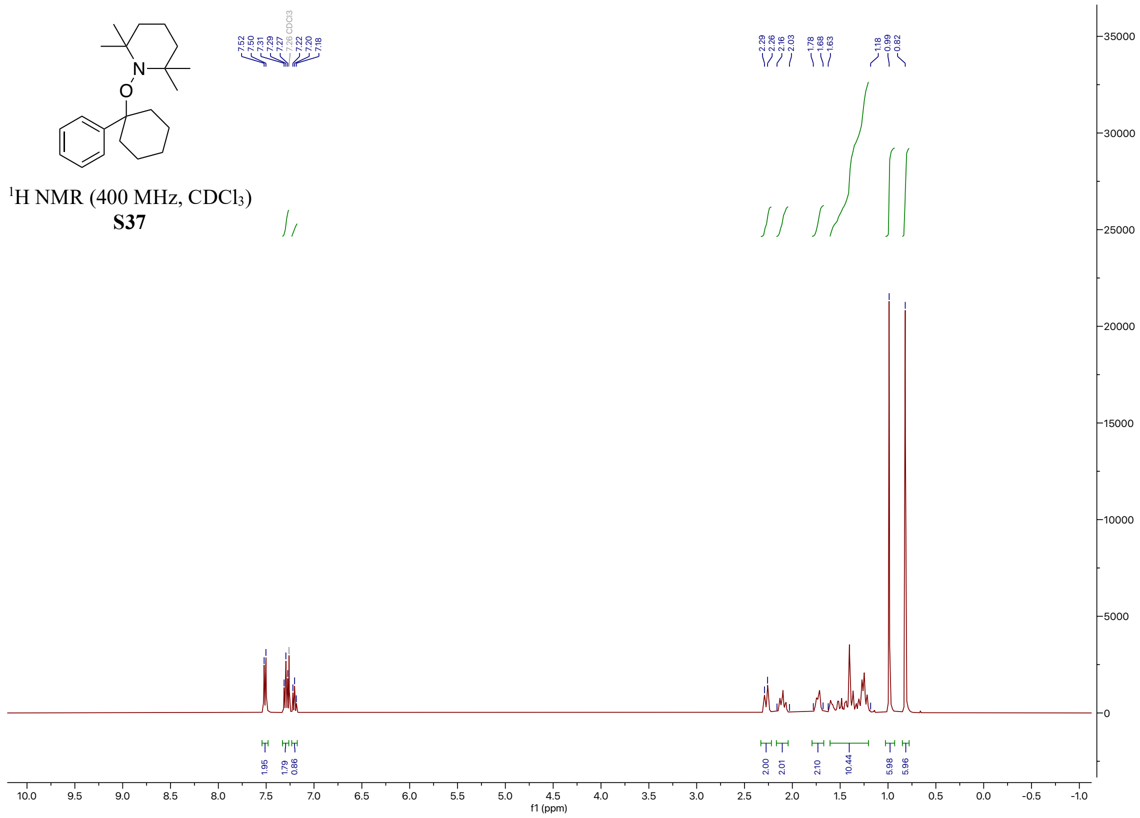

^{1}H NMR (400 MHz, $CDCl_3$)
S37

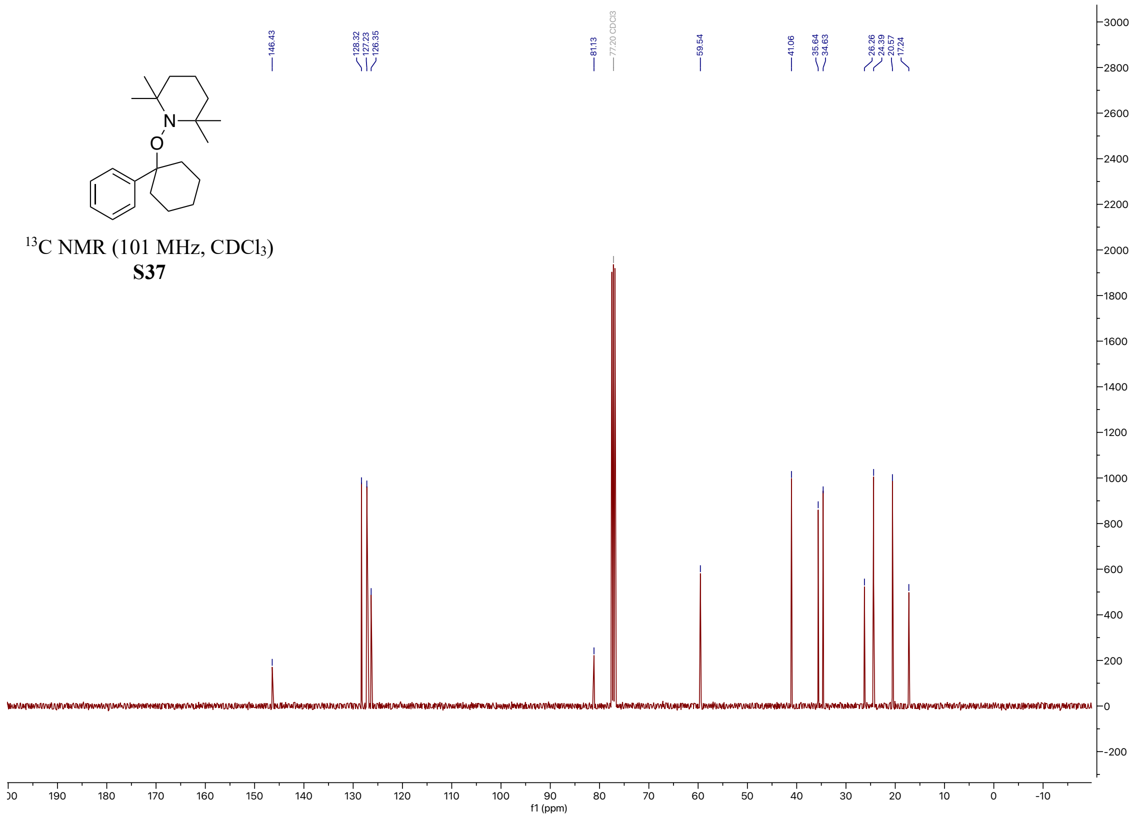

^{13}C NMR (101 MHz, $CDCl_3$)
S37

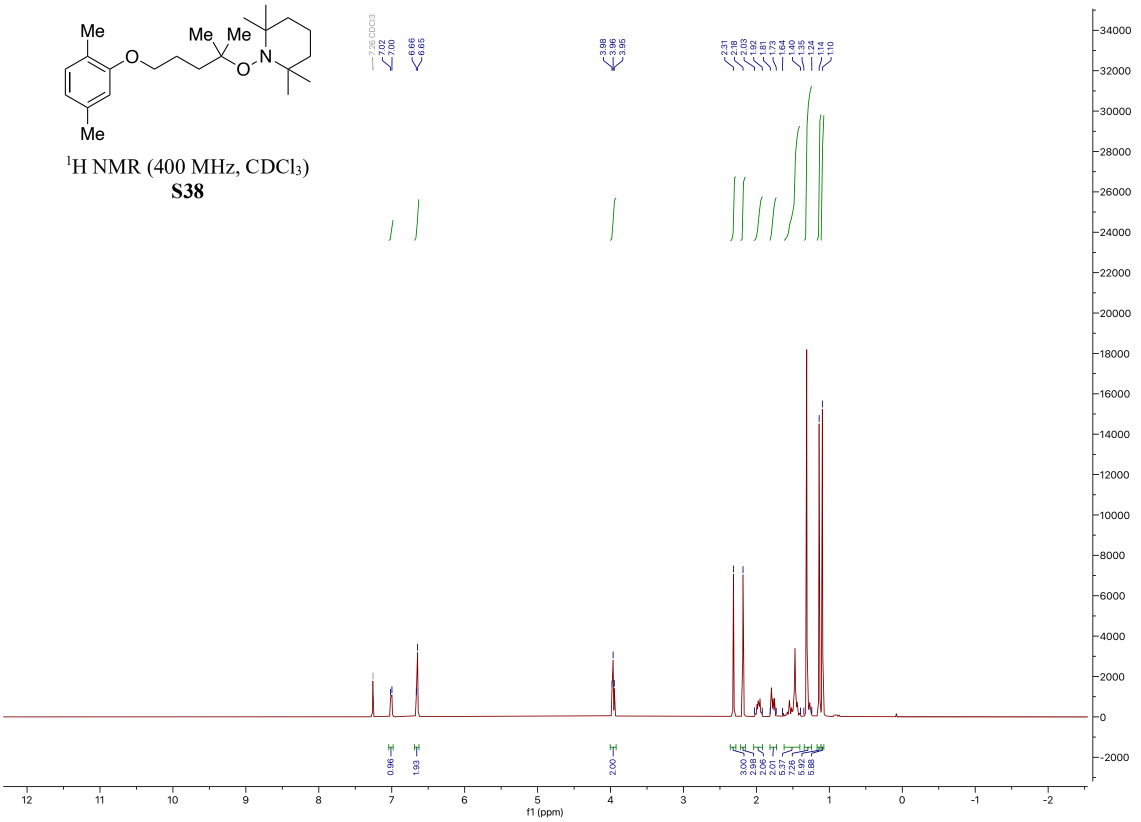

^{1}H NMR (400 MHz, $CDCl_3$)
S38

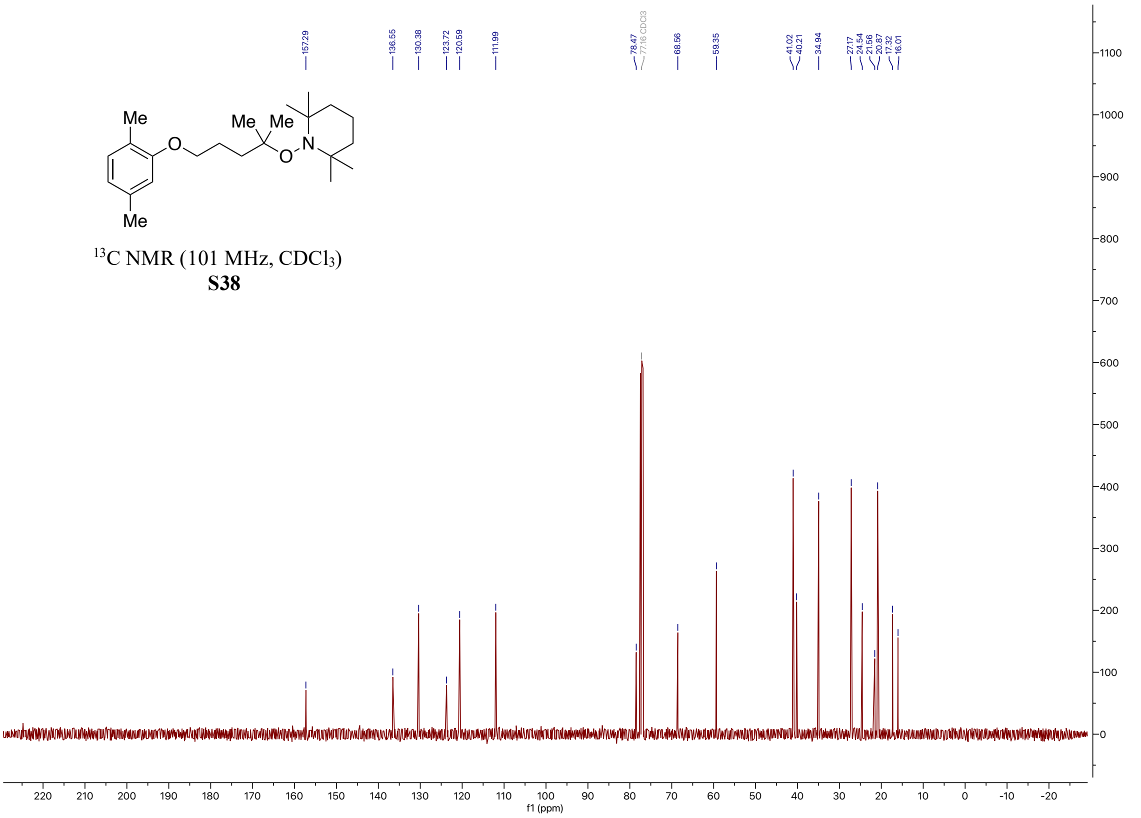

^{13}C NMR (101 MHz, $CDCl_3$)
S38

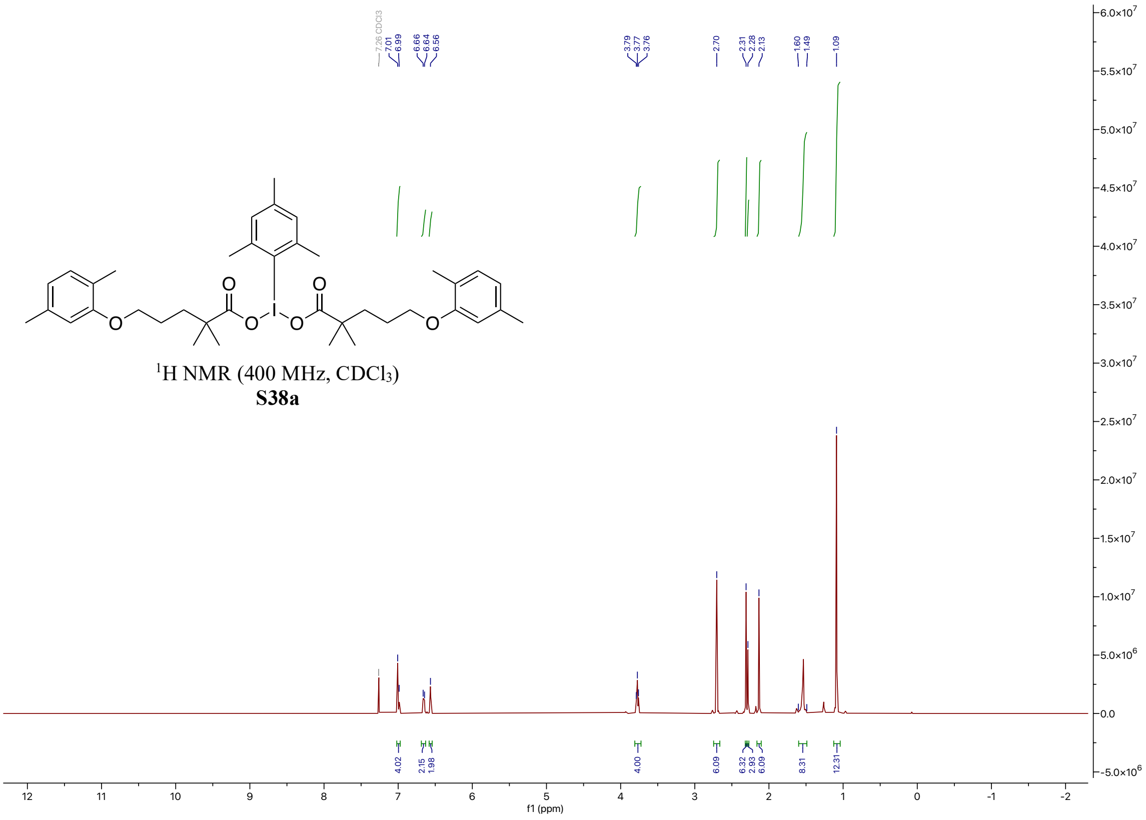

^{1}H NMR (400 MHz, CDCl$_3$)
S38a
7.26 CDCl3
7.01
6.99
6.66
6.64
6.56
3.79
3.77
3.76
2.70
2.31
2.28
2.13
1.60
1.49
1.09
4.02
2.15
1.98
4.00
6.09
6.32
2.93
6.09
8.31
12.31
f1 (ppm)

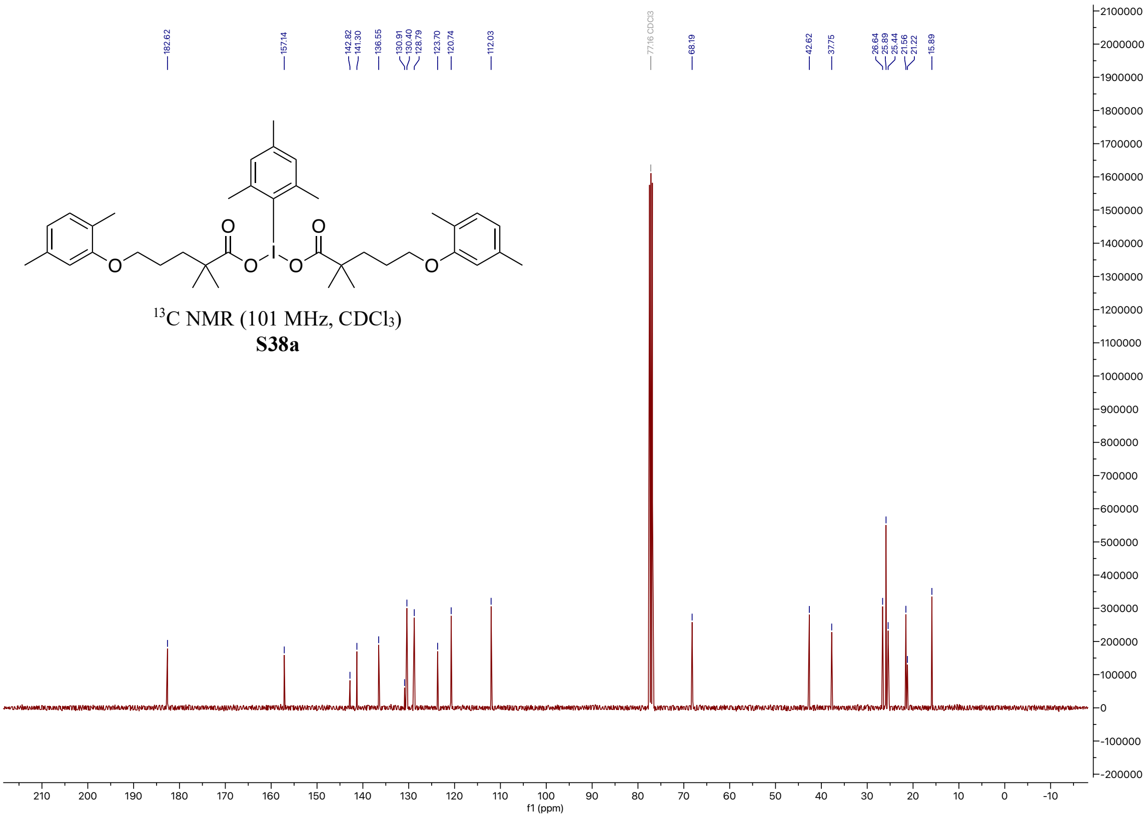

^{13}C NMR (101 MHz, CDCl$_3$)
S38a
182.62
157.14
142.82
141.30
136.55
130.91
130.40
128.79
123.70
120.74
112.03
77.16 CDCl3
68.19
42.62
37.75
26.64
25.89
25.44
21.56
21.22
15.89
f1 (ppm)

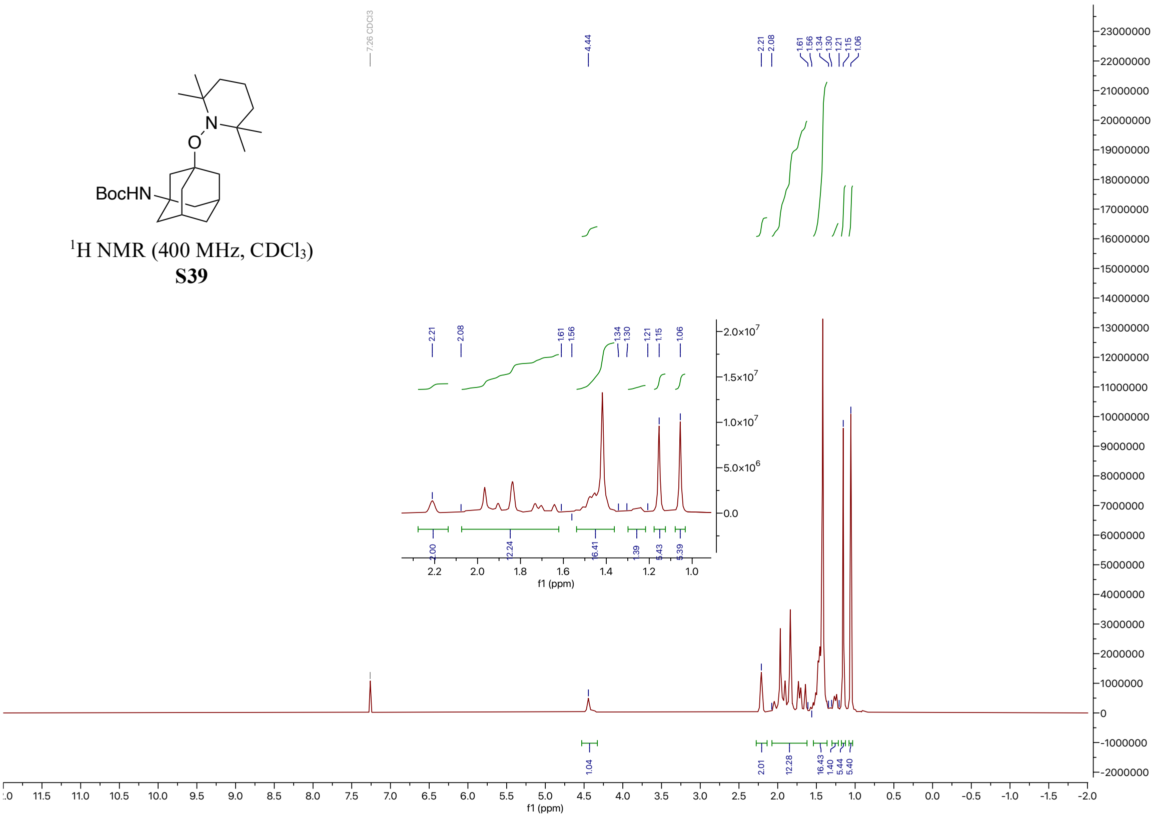
BocHN
O
N
^{1}H NMR (400 MHz, CDCl$_3$)
S39
7.26 CDCl3
4.44
2.21
2.08
1.61
1.56
1.34
1.30
1.21
1.15
1.06
1.04
2.01
12.28
16.43
1.40
5.44
5.40
2.00
12.24
16.41
1.39
5.43
5.39
f1 (ppm)

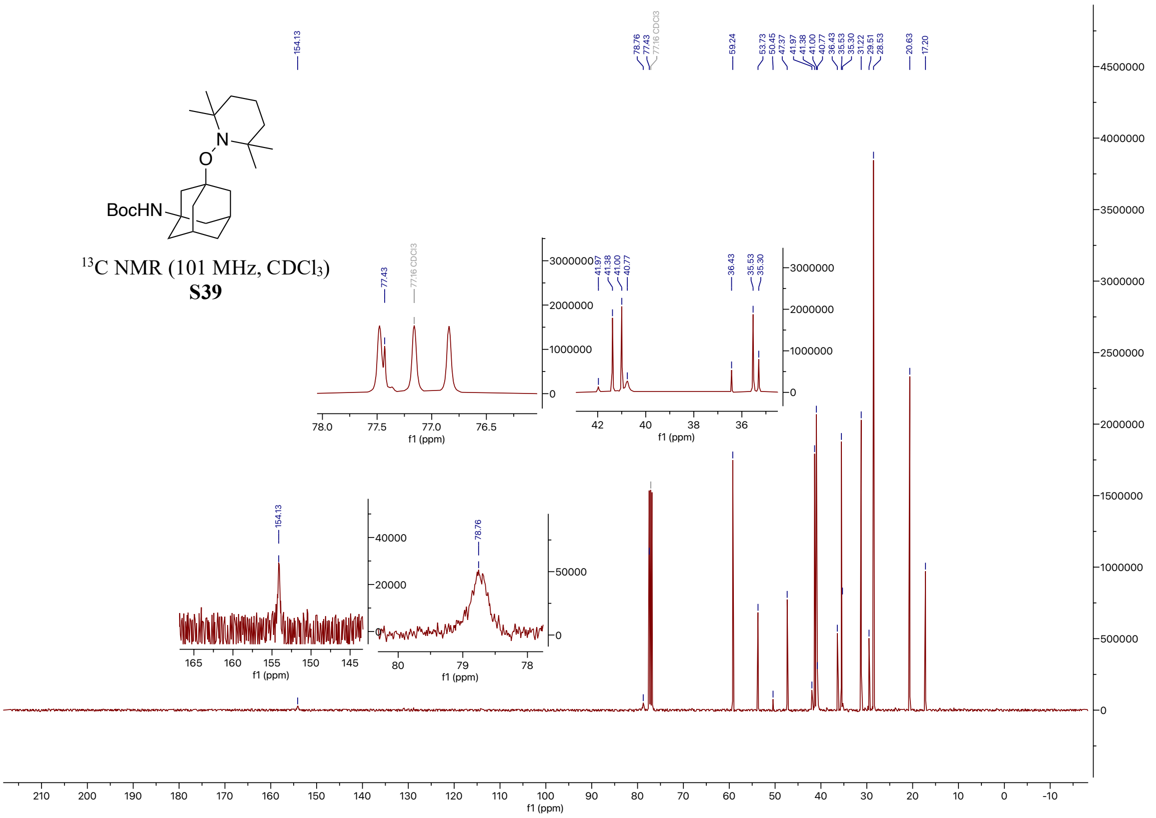
BocHN
O
N
^{13}C NMR (101 MHz, CDCl$_3$)
S39
154.13
78.76
77.43
77.16 CDCl3
59.24
53.73
50.45
47.37
41.97
41.38
41.00
40.77
36.43
35.53
35.30
31.22
29.51
28.53
20.63
17.20
f1 (ppm)

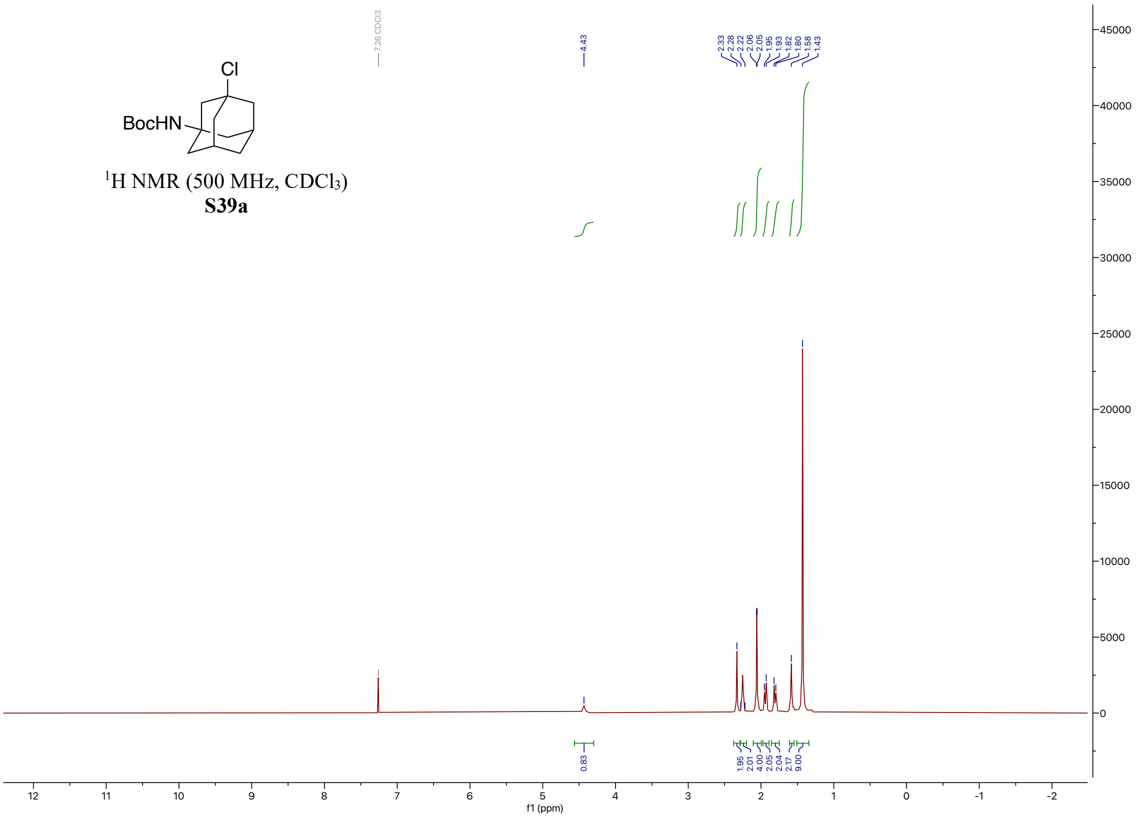
Cl
BocHN
^{1}H NMR (500 MHz, $CDCl_3$)
S39a
7.26 CDCl3
4.43
2.33 2.28 2.22 2.06 2.05 1.95 1.93 1.82 1.80 1.58 1.43
0.83
1.95 2.01 4.00 2.05 2.04 2.17 9.00
f1 (ppm)

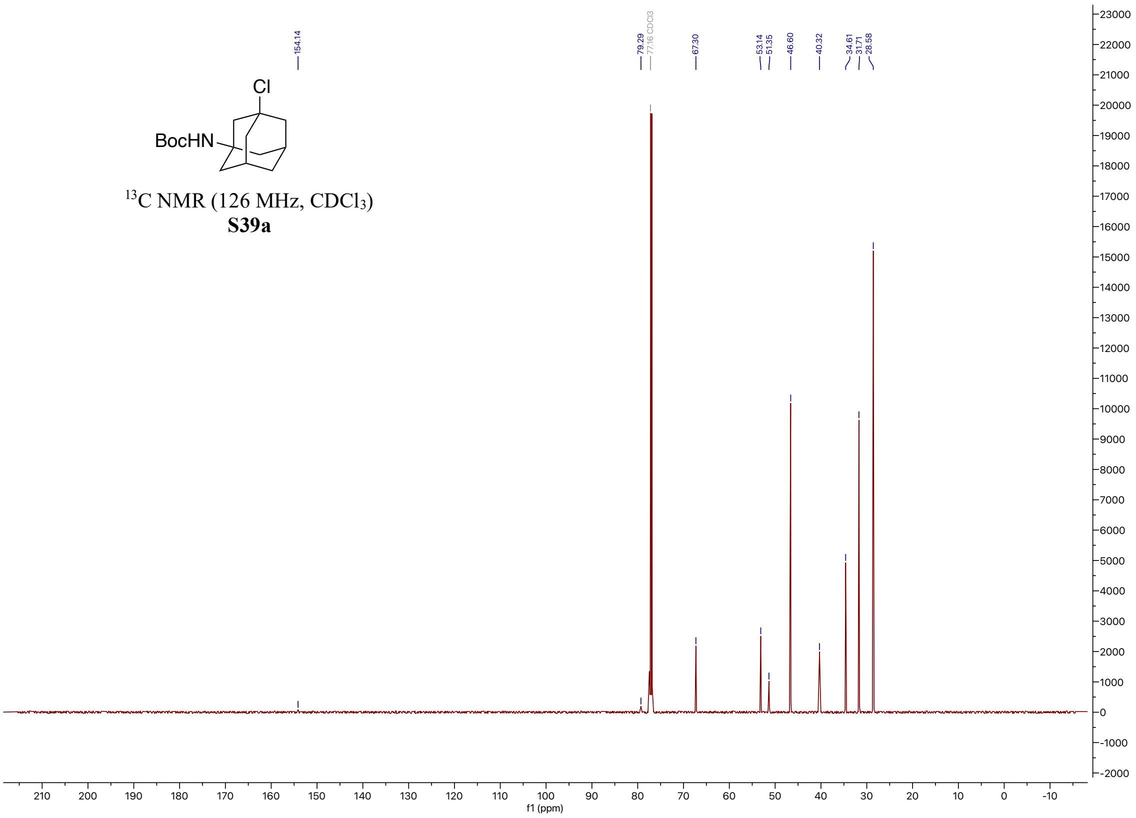
Cl
BocHN
^{13}C NMR (126 MHz, $CDCl_3$)
S39a
154.14
79.29
77.16 CDCl3
67.30
53.14 51.35
46.60
40.32
34.61 31.71 28.58
f1 (ppm)

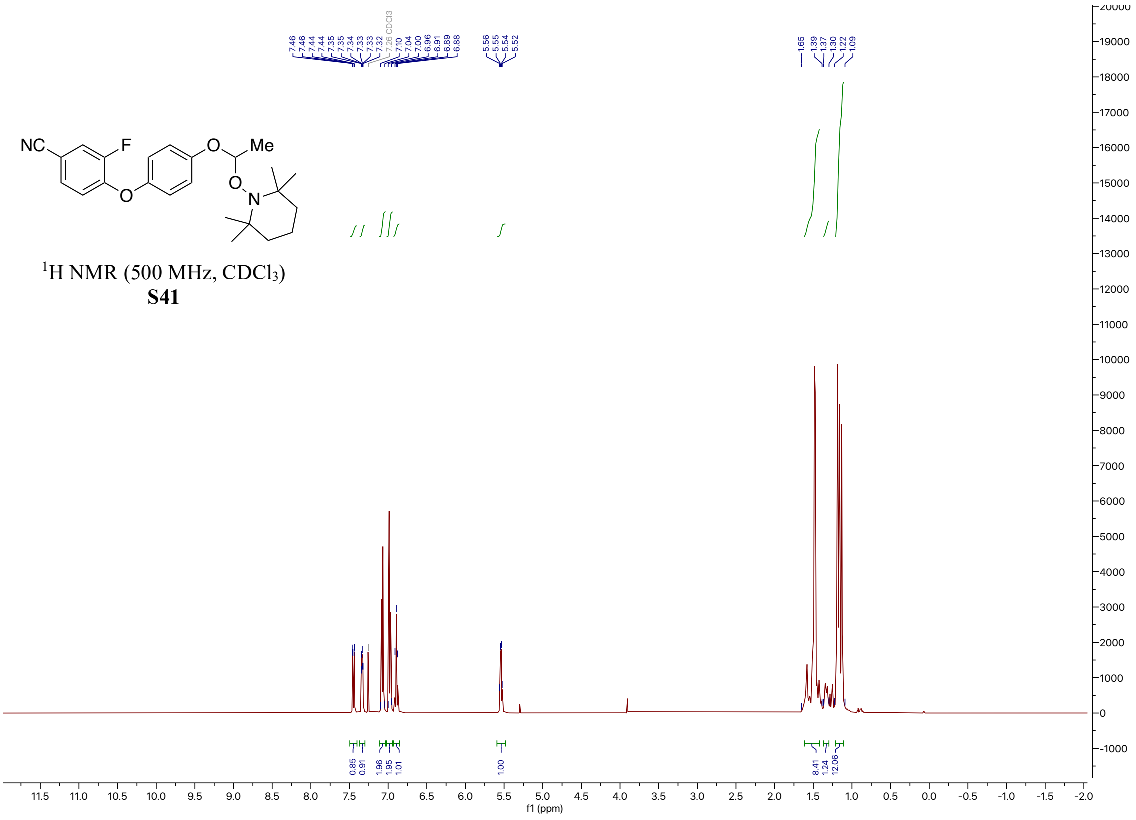

^{1}H NMR (500 MHz, $CDCl_3$)
S41

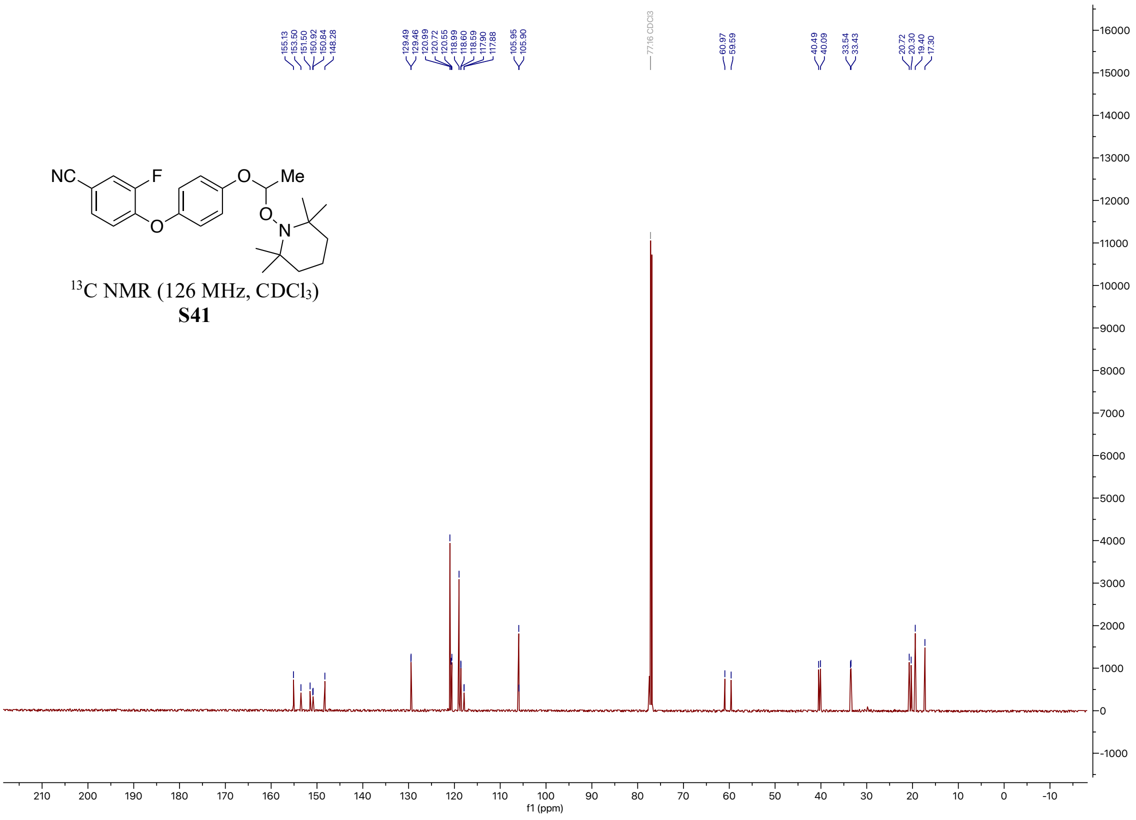

^{13}C NMR (126 MHz, $CDCl_3$)
S41

^{19}F NMR (377 MHz, $CDCl_3$)

S41

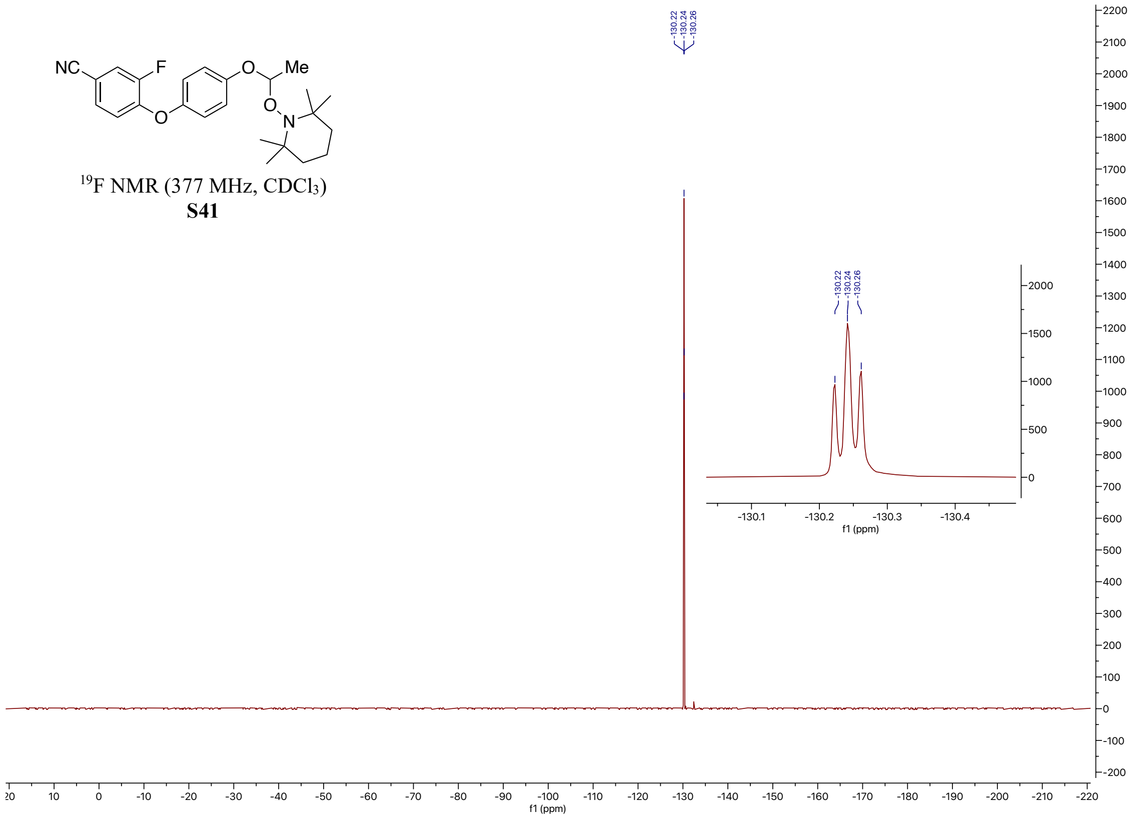

^{1}H NMR (400 MHz, $CDCl_3$)

S42

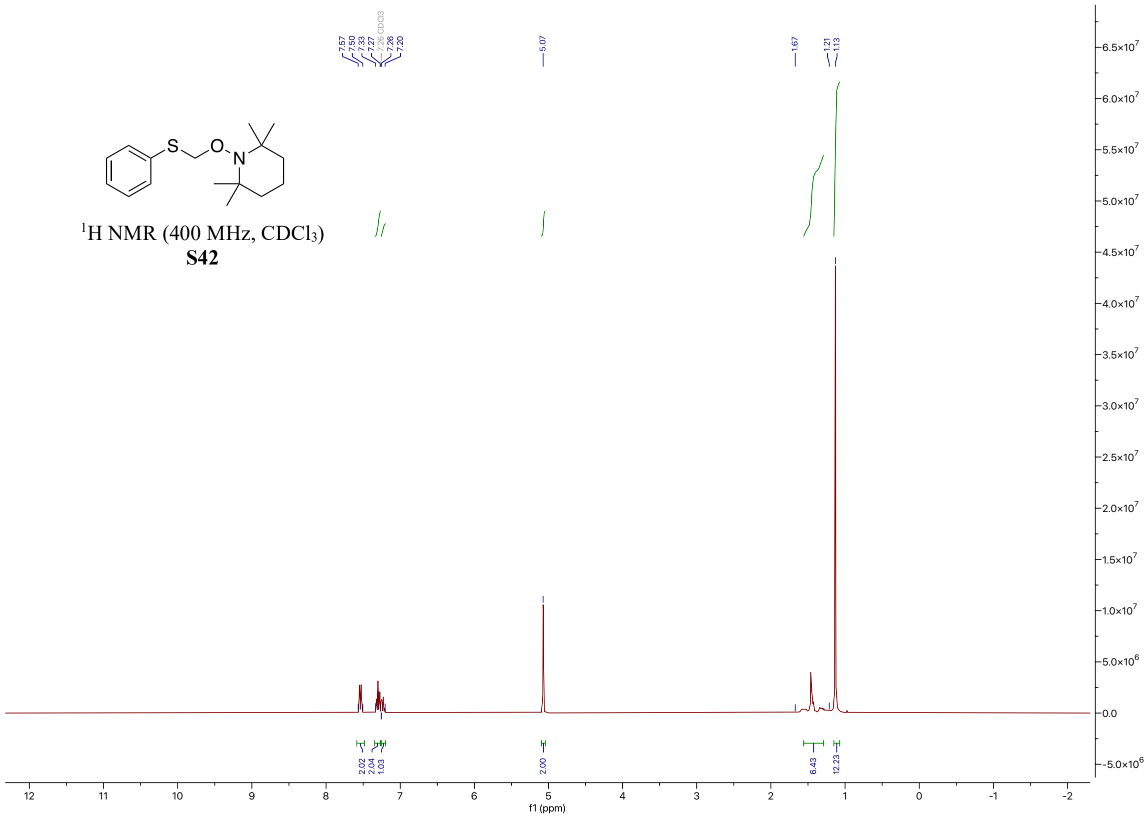

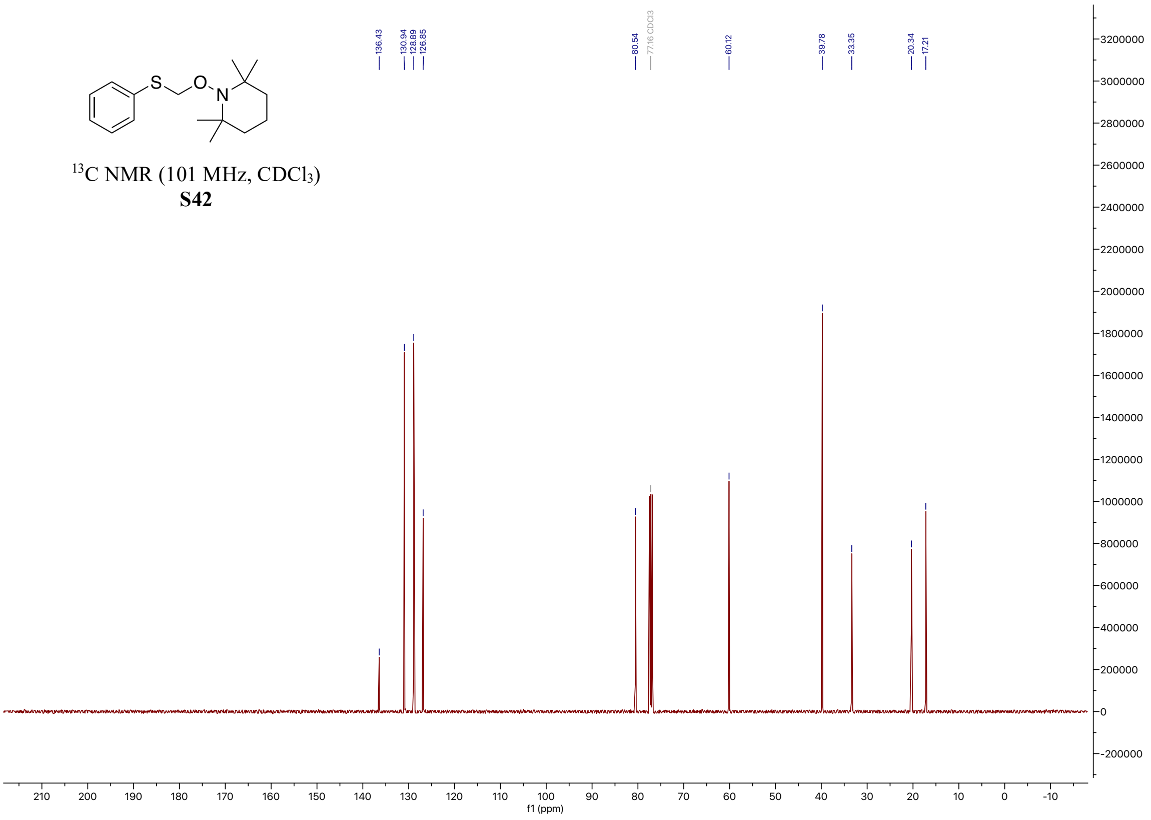

^{13}C NMR (101 MHz, $CDCl_3$)
S42

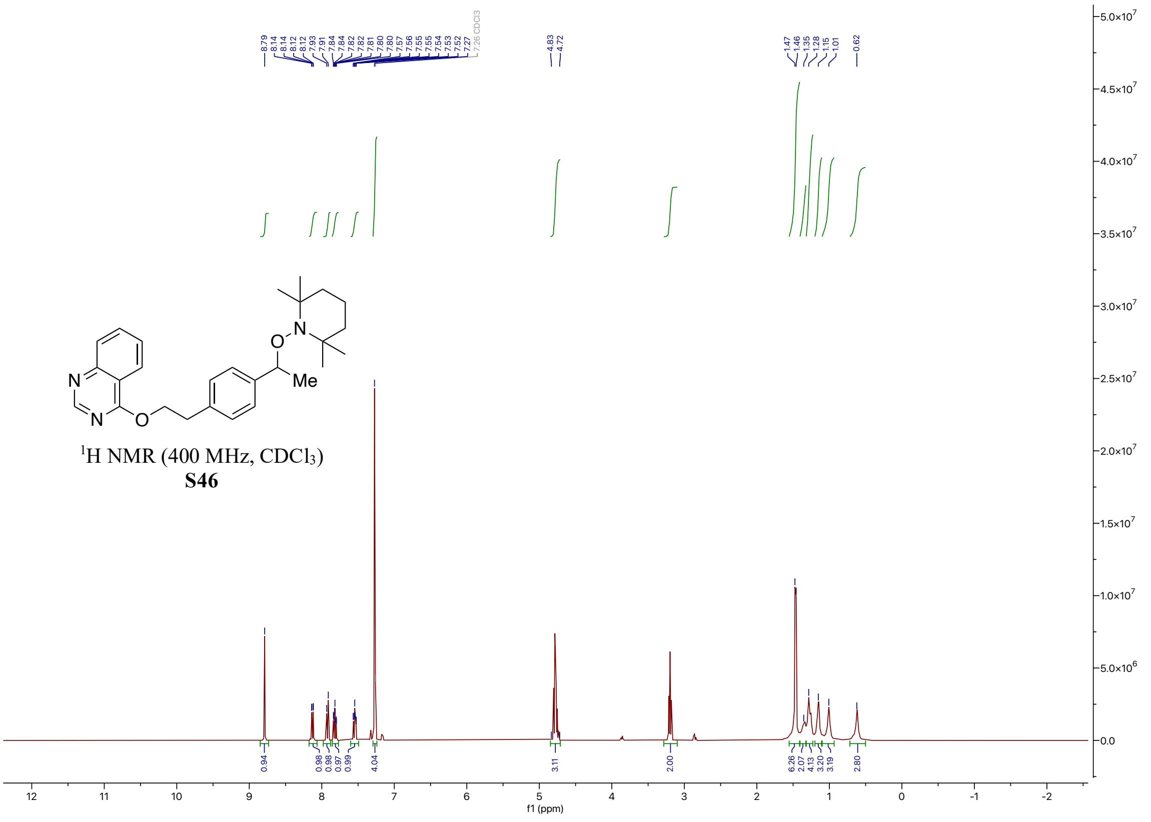

^{1}H NMR (400 MHz, $CDCl_3$)
S46

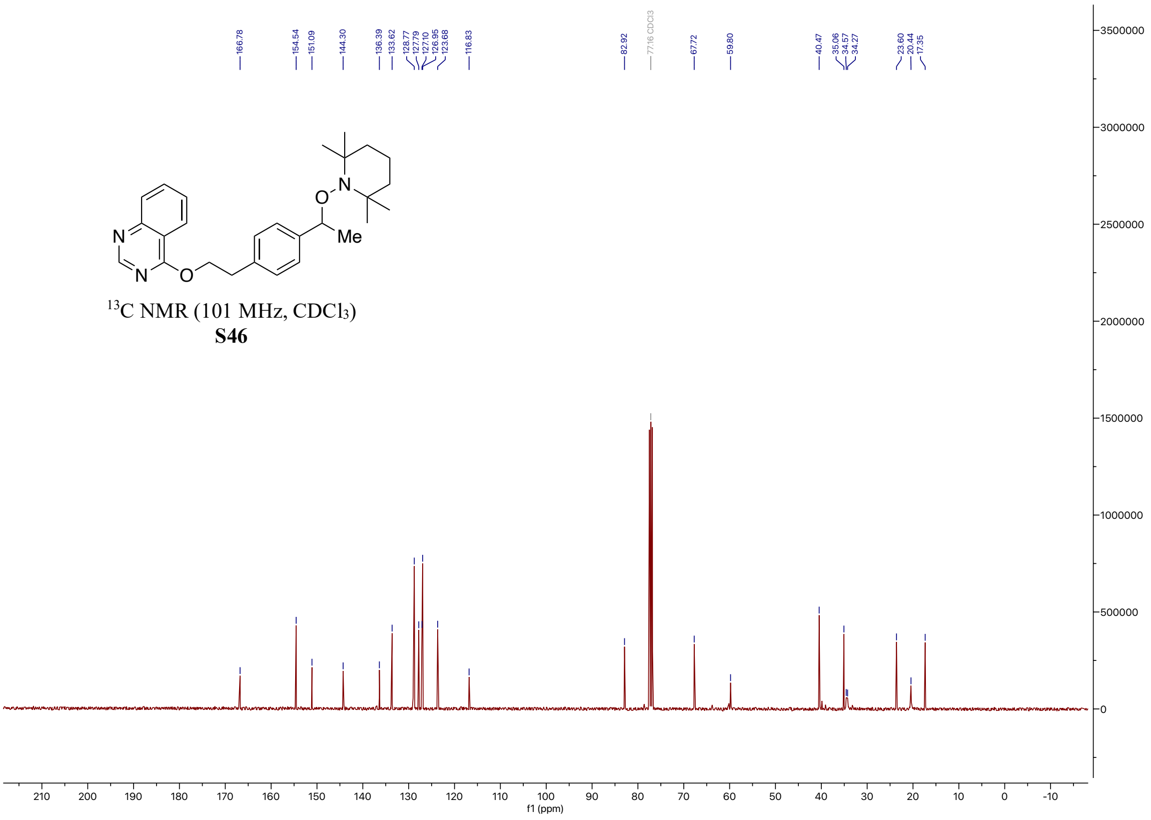

^{13}C NMR (101 MHz, $CDCl_3$)
S46

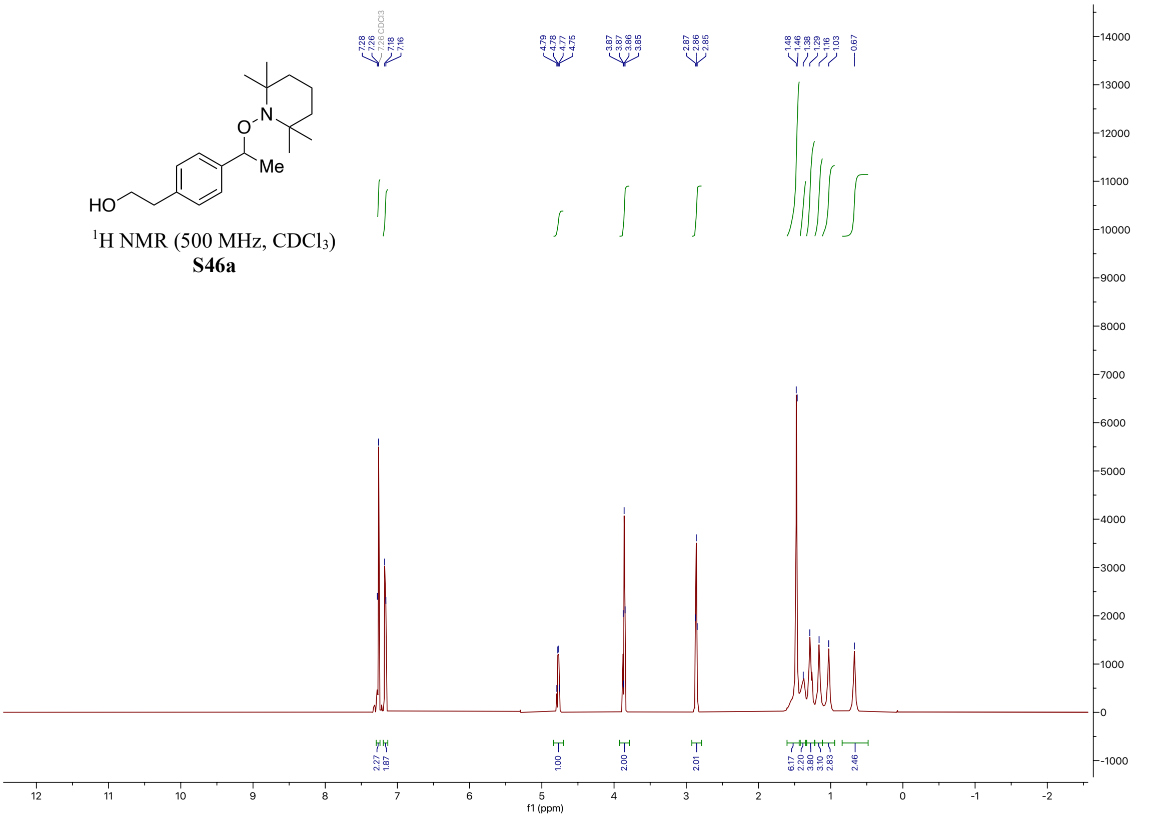

^{1}H NMR (500 MHz, $CDCl_3$)
S46a

^{13}C NMR (121 MHz, $CDCl_3$)
S46a

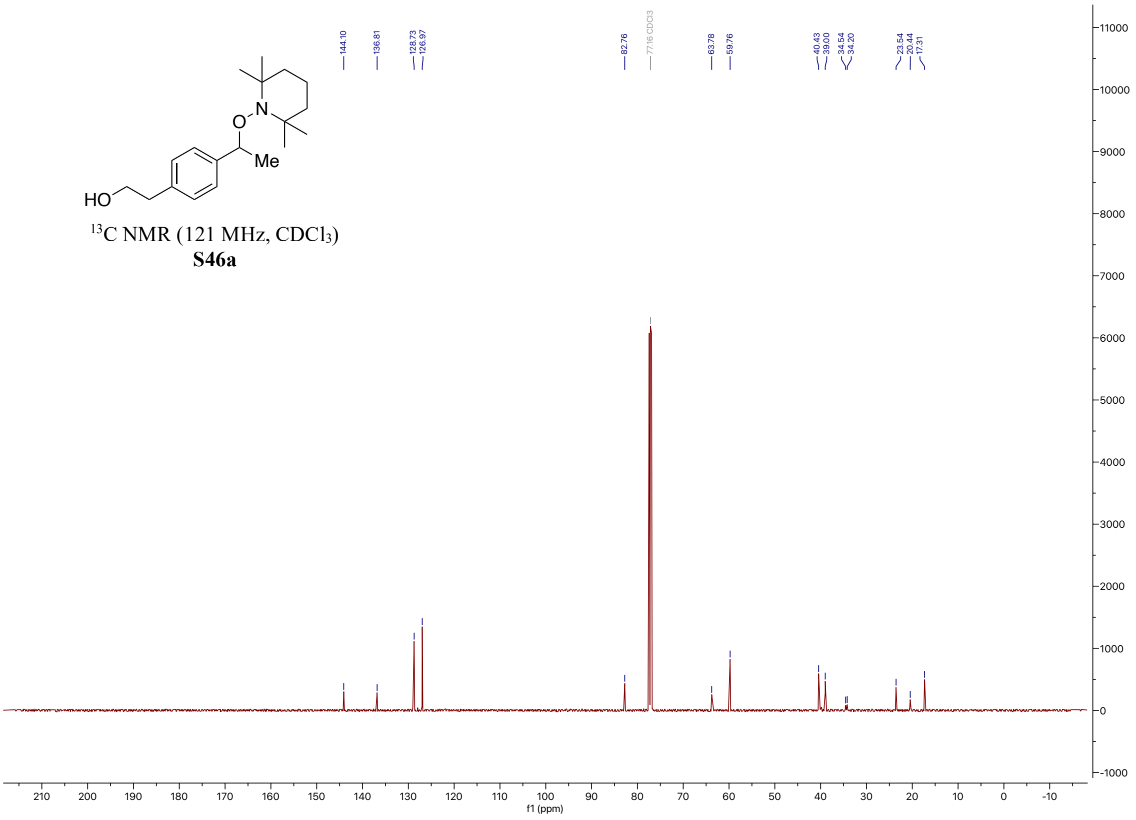

^{1}H NMR (400 MHz, $CDCl_3$)
S46b

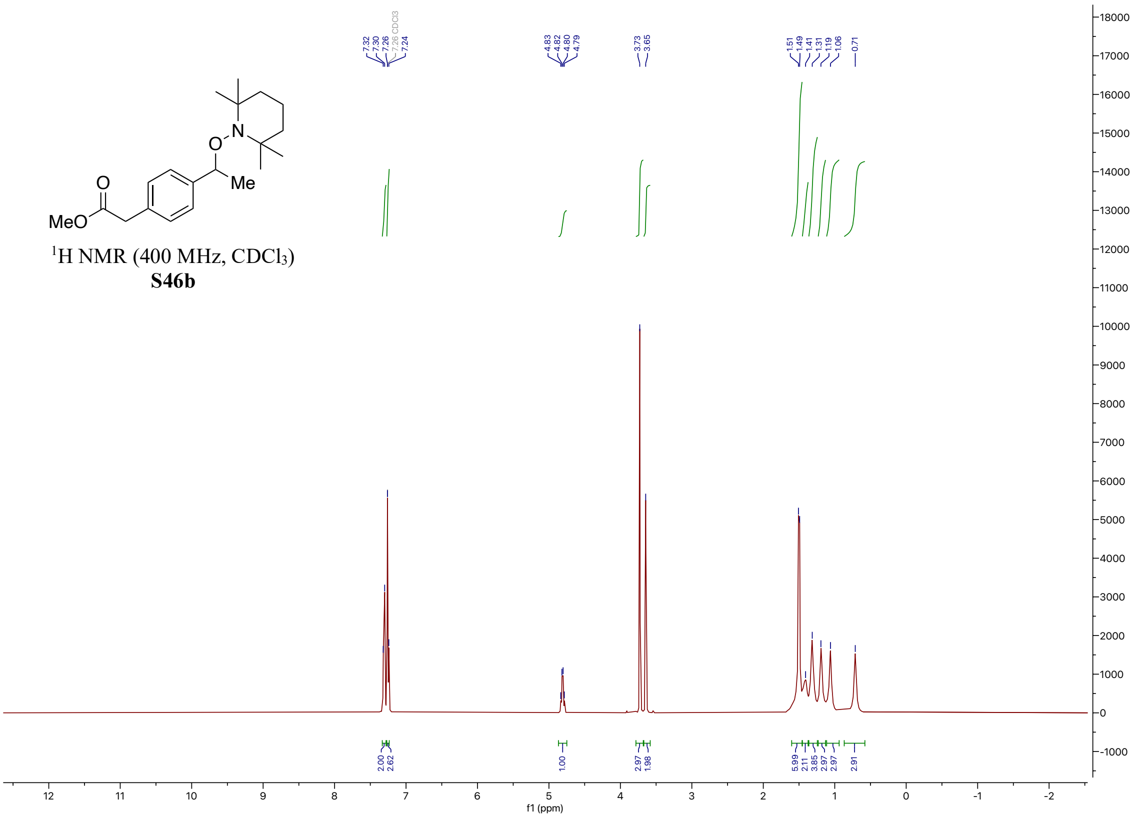

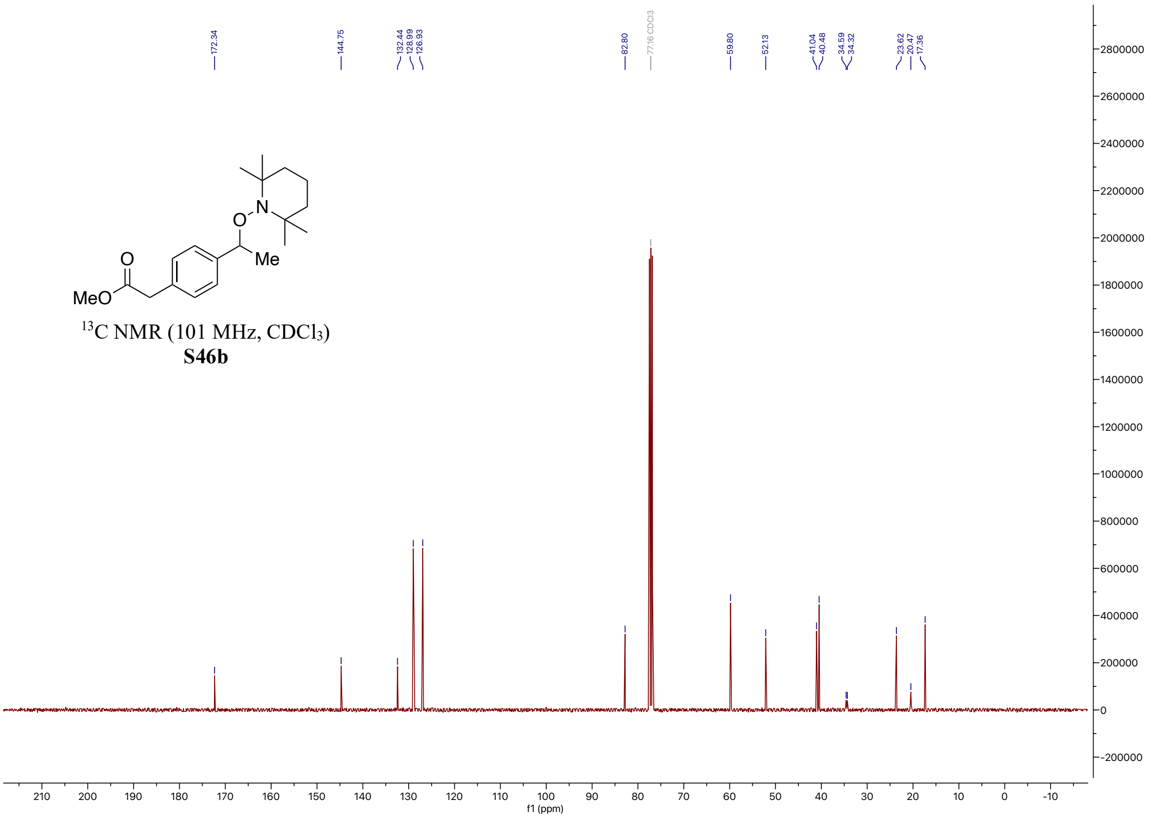

^{13}C NMR (101 MHz, $CDCl_3$)

S46b

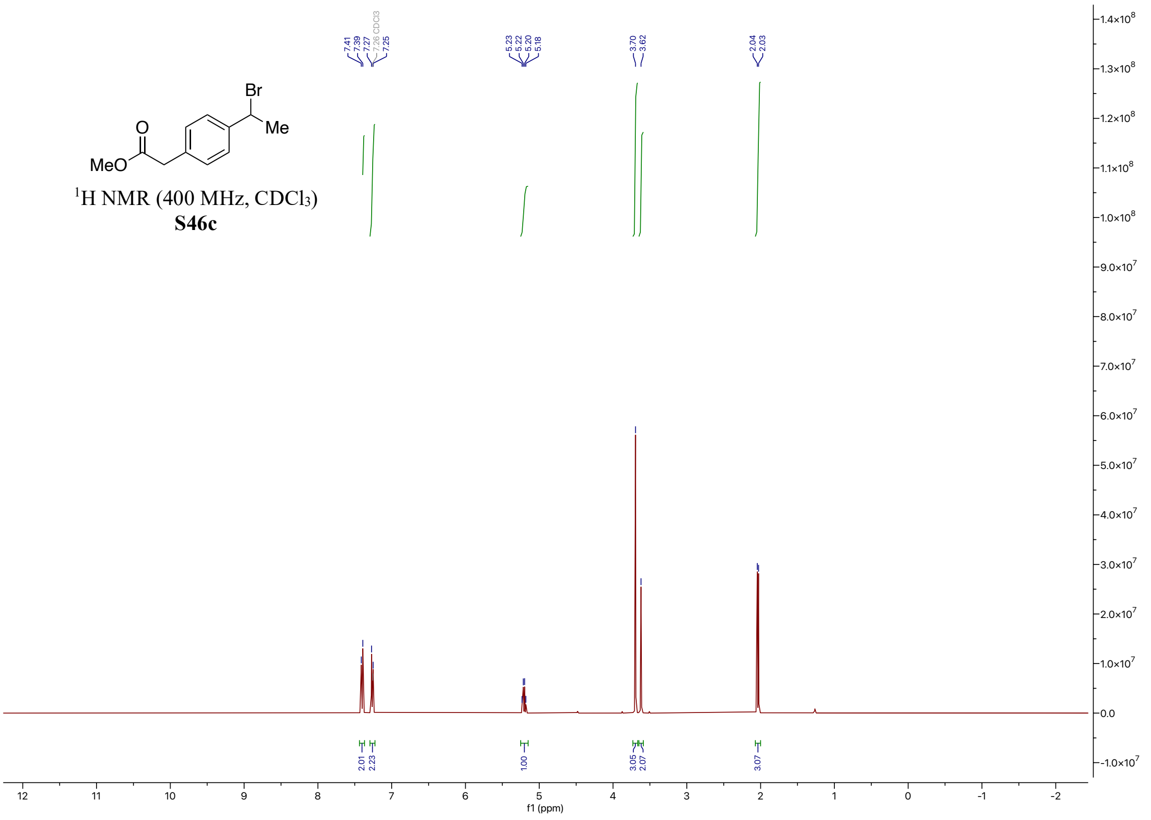

^{1}H NMR (400 MHz, $CDCl_3$)

S46c

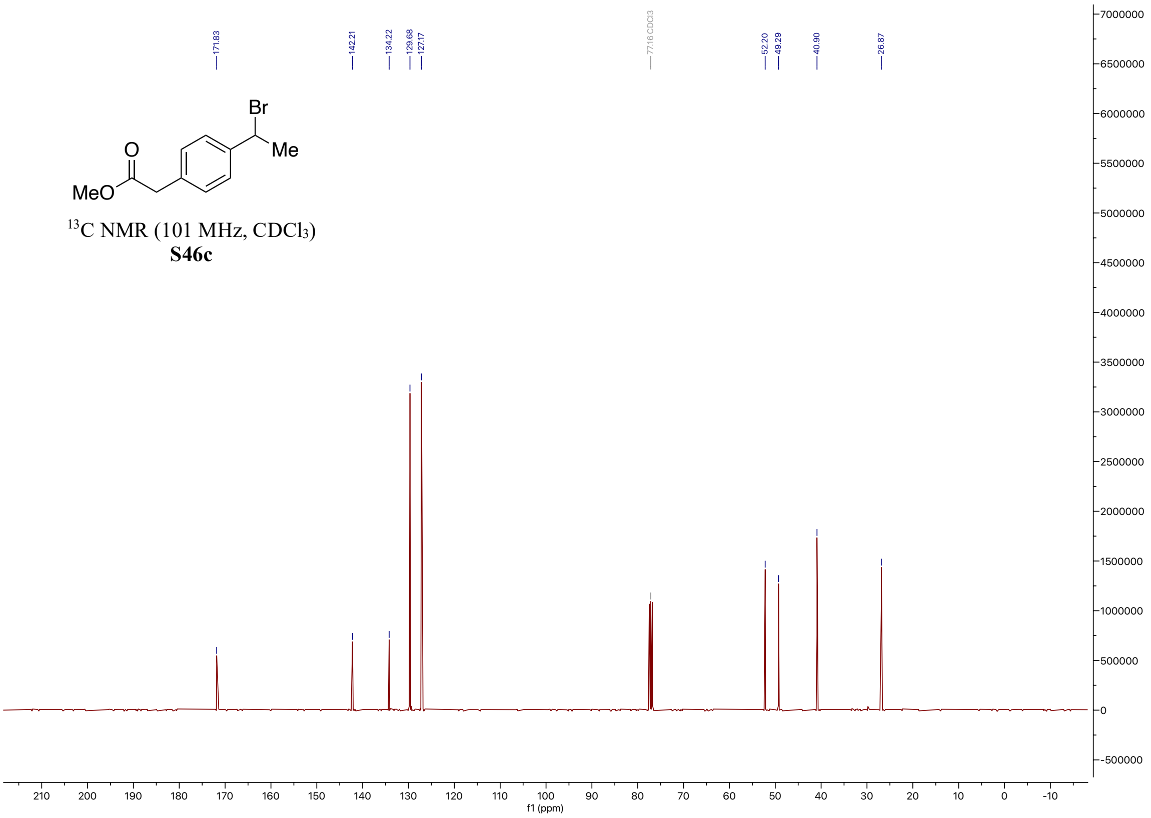

^{13}C NMR (101 MHz, $CDCl_3$)
S46c

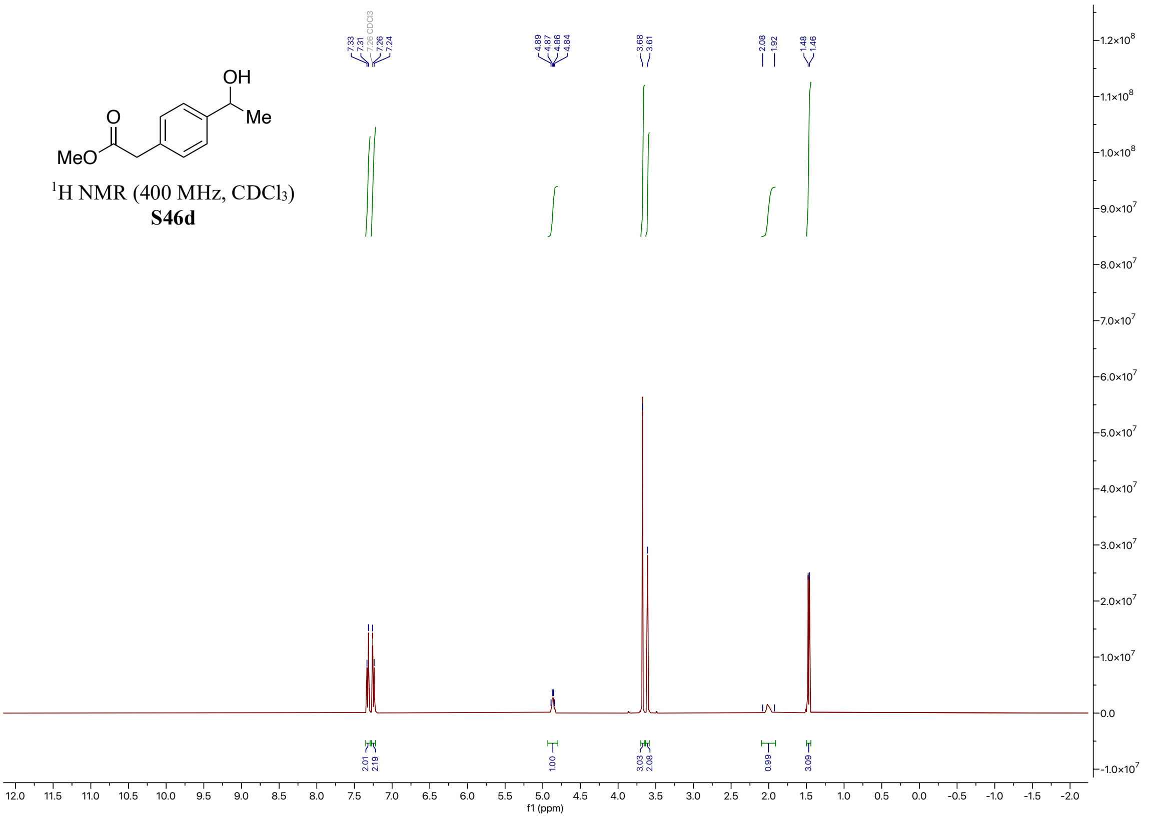

^{1}H NMR (400 MHz, $CDCl_3$)
S46d

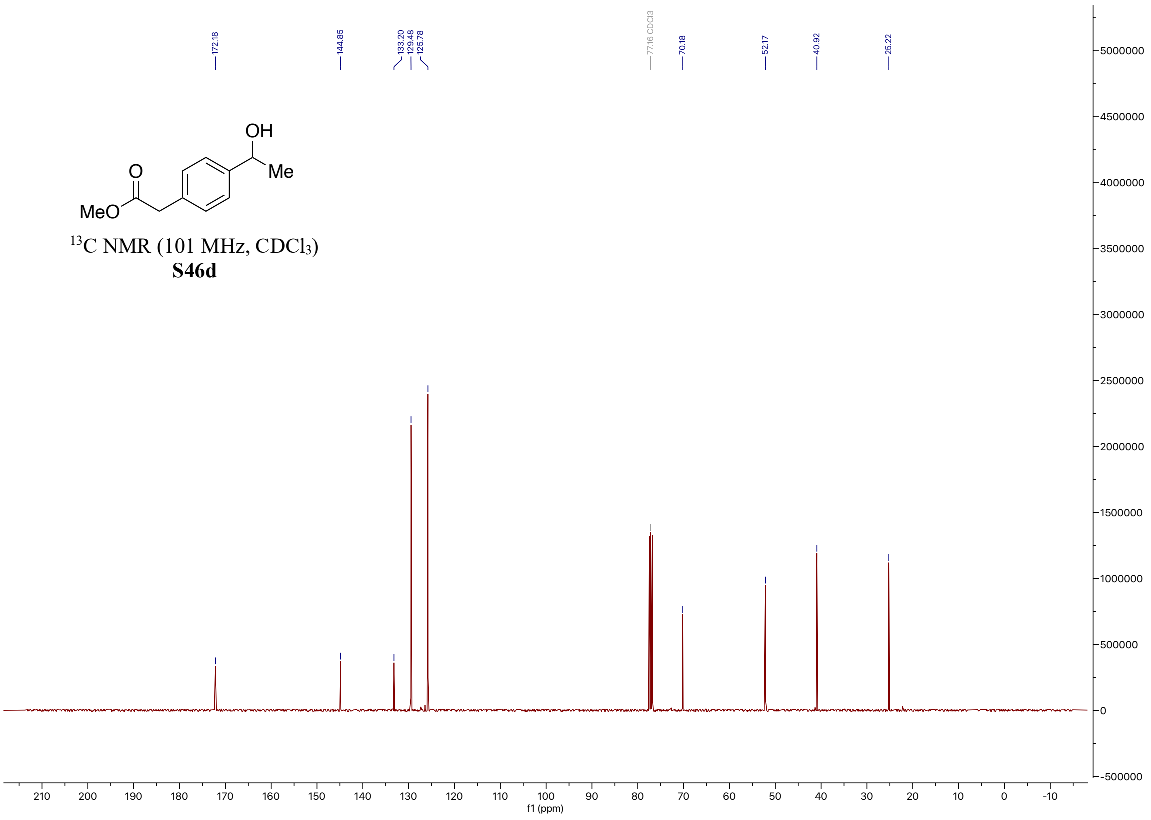

^{13}C NMR (101 MHz, $CDCl_3$)

S46d

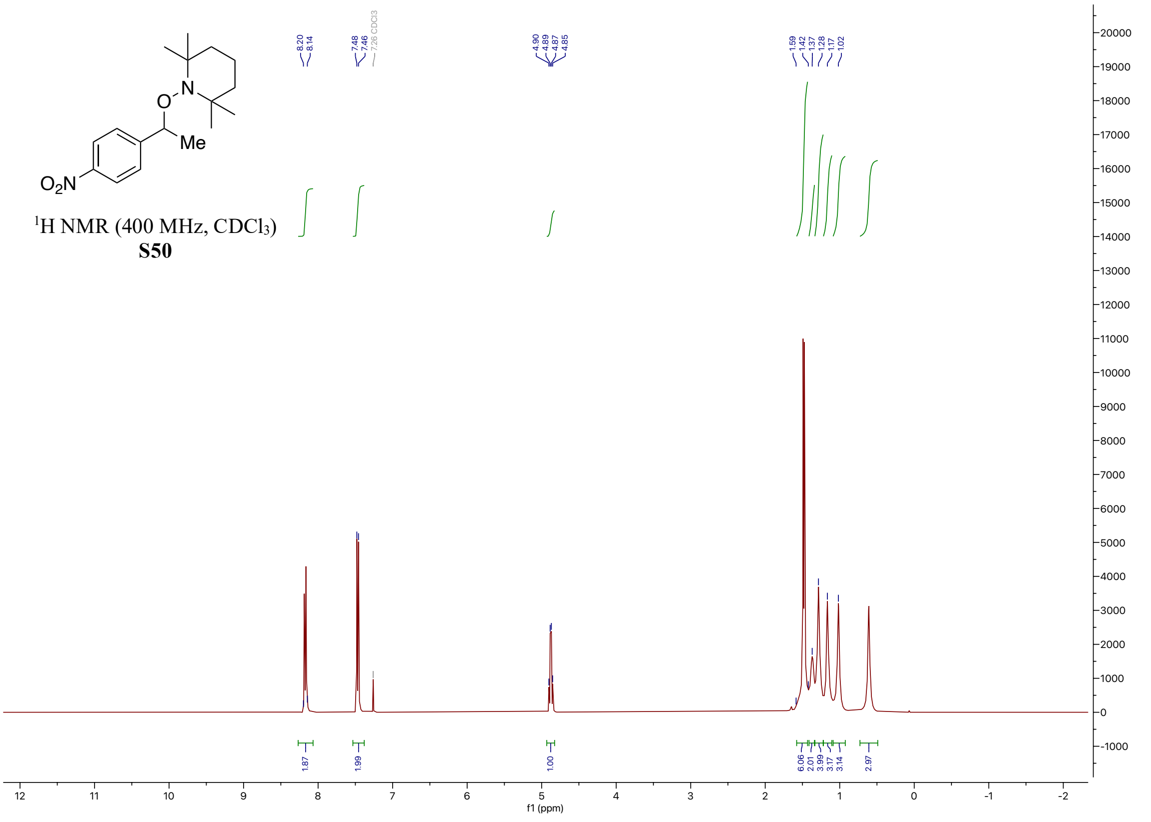

^{1}H NMR (400 MHz, $CDCl_3$)

S50

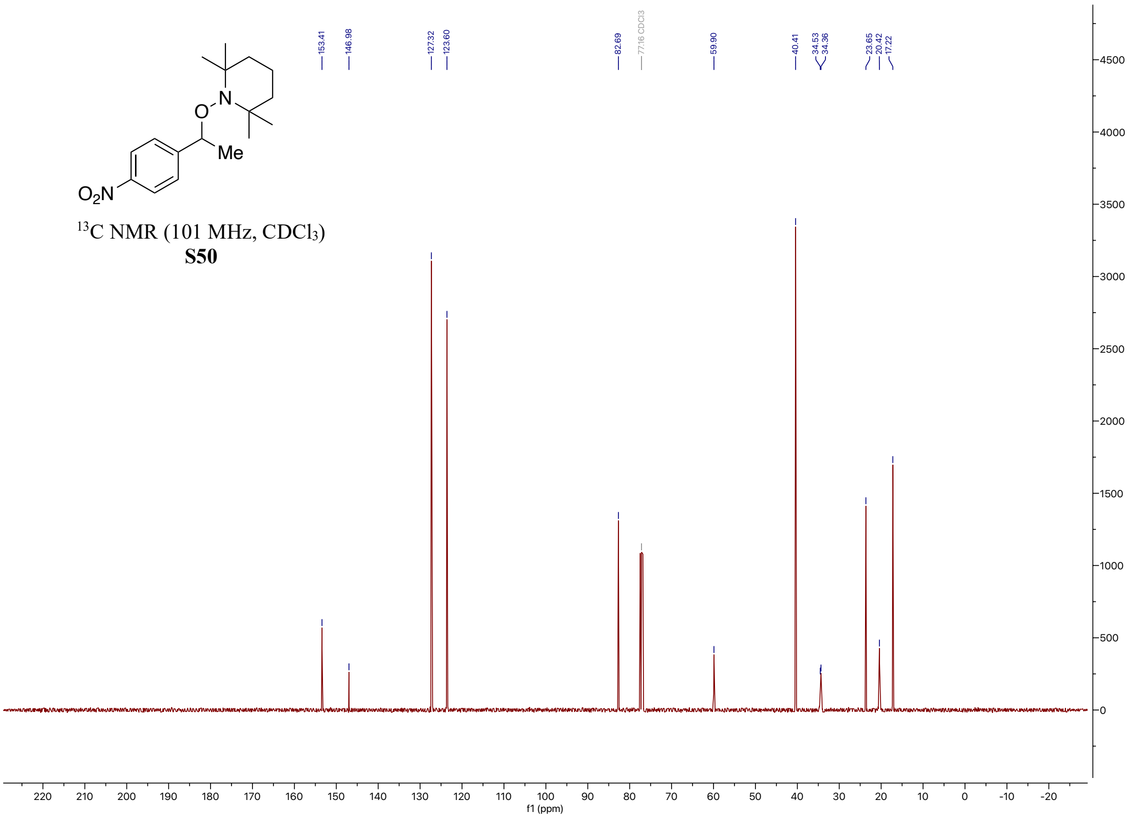

O2N
Me
13C NMR (101 MHz, CDCl3)
S50
153.41
146.98
127.32
123.60
82.69
77.16 CDCl3
59.90
40.41
34.53
34.36
23.65
20.42
17.22
f1 (ppm)

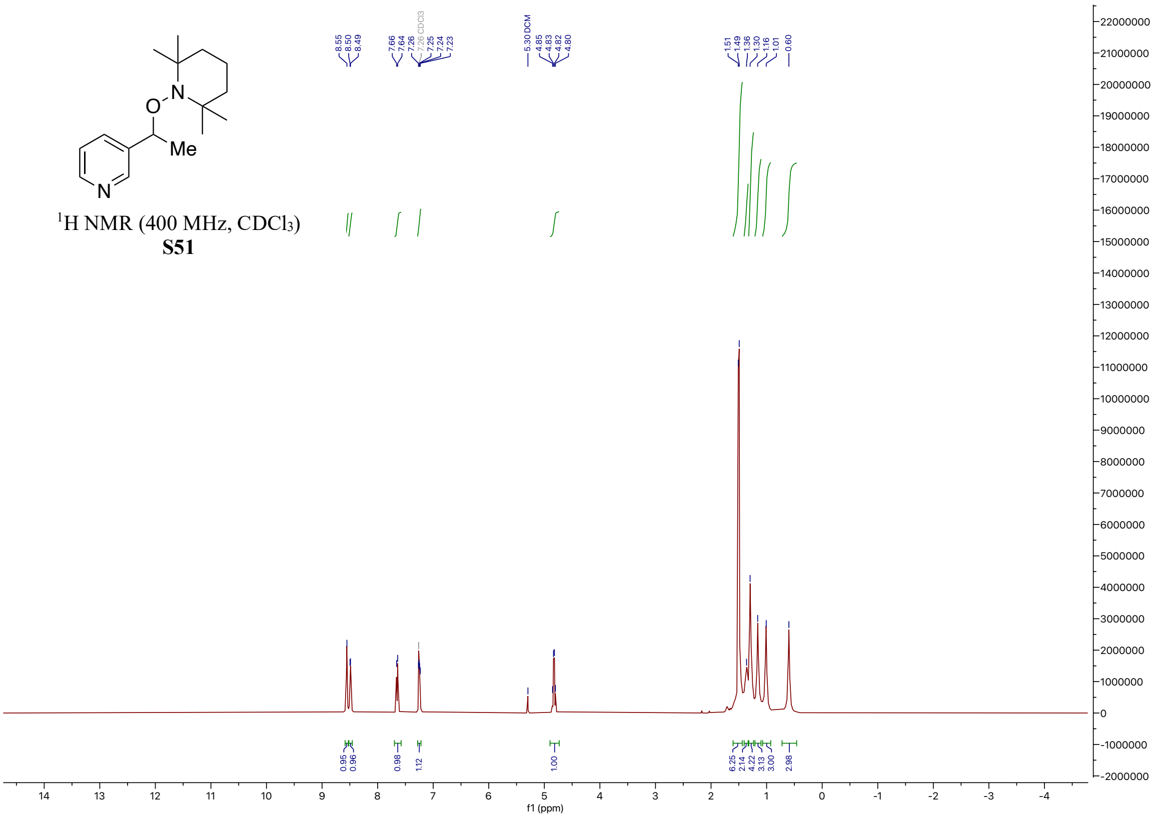

N
Me
1H NMR (400 MHz, CDCl3)
S51
8.55
8.50
8.49
7.66
7.64
7.26 CDCl3
7.25
7.24
7.23
5.30 DCM
4.85
4.83
4.82
4.80
1.51
1.49
1.38
1.30
1.16
1.01
0.60
0.95
0.96
0.98
1.12
1.00
6.25
2.14
4.22
3.13
3.00
2.98
f1 (ppm)

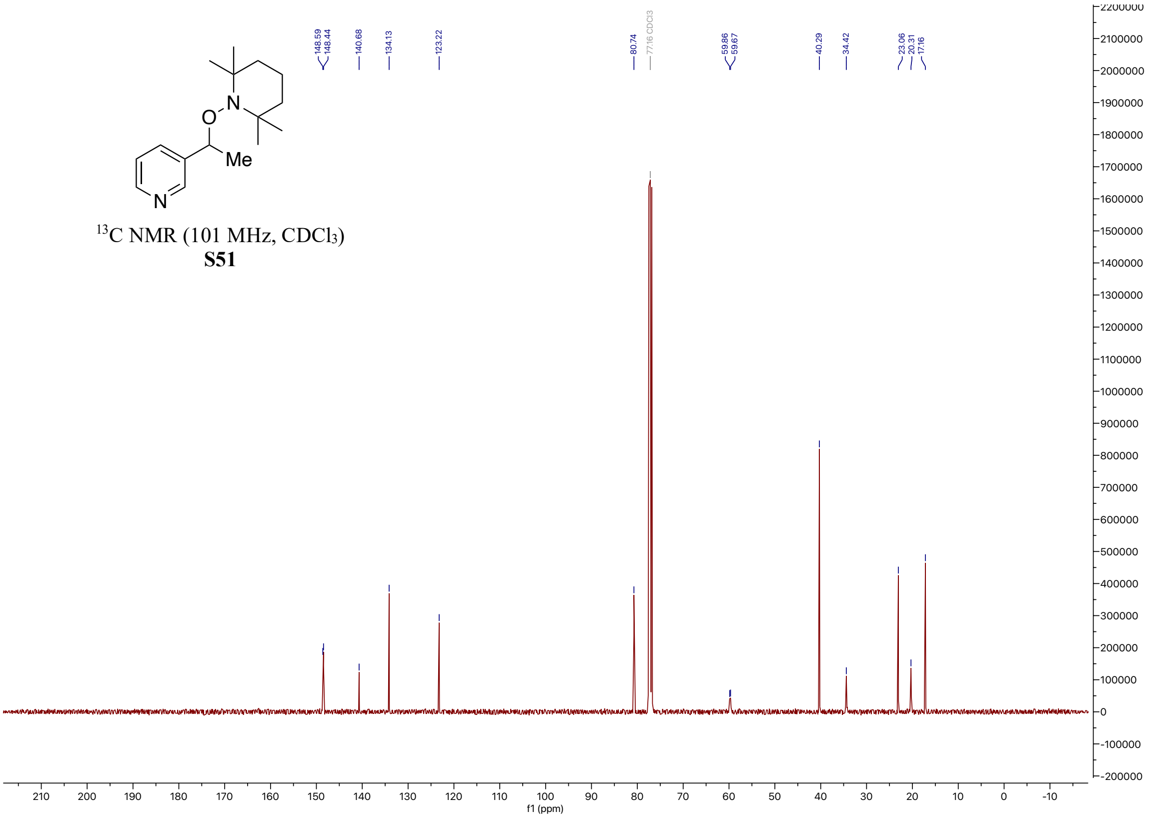

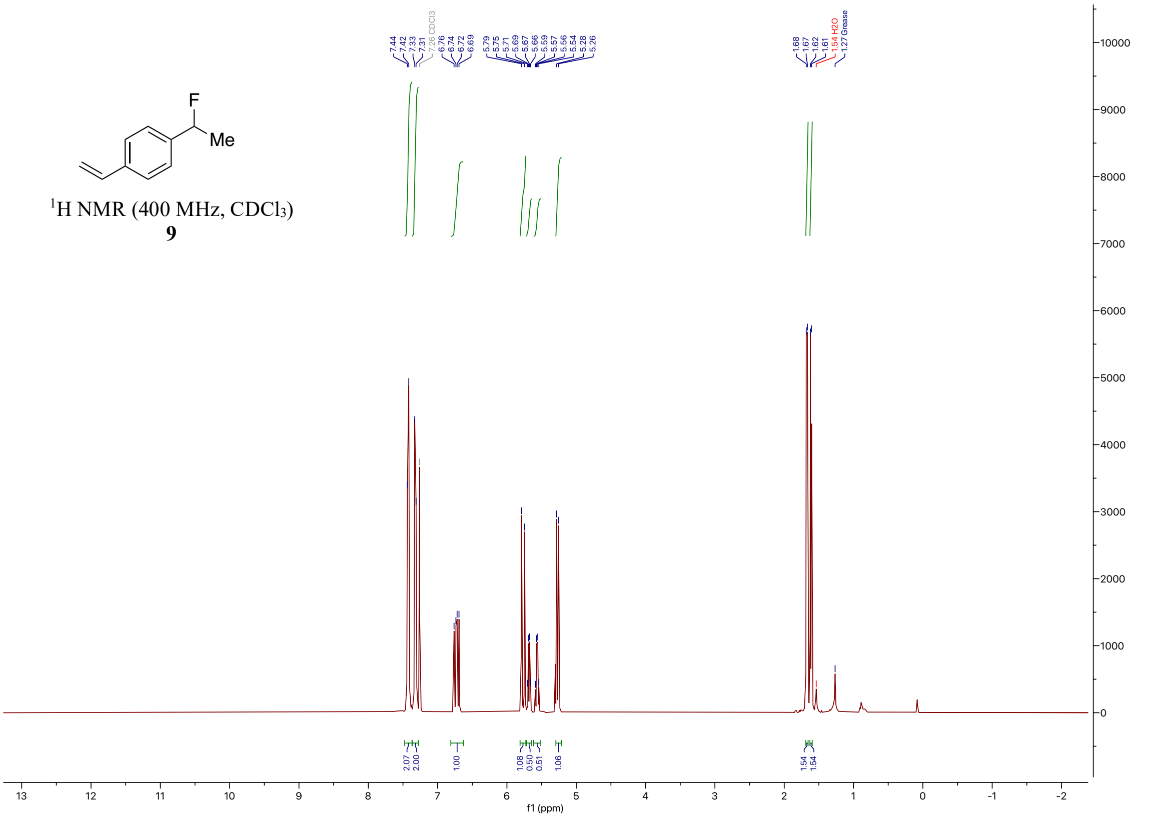

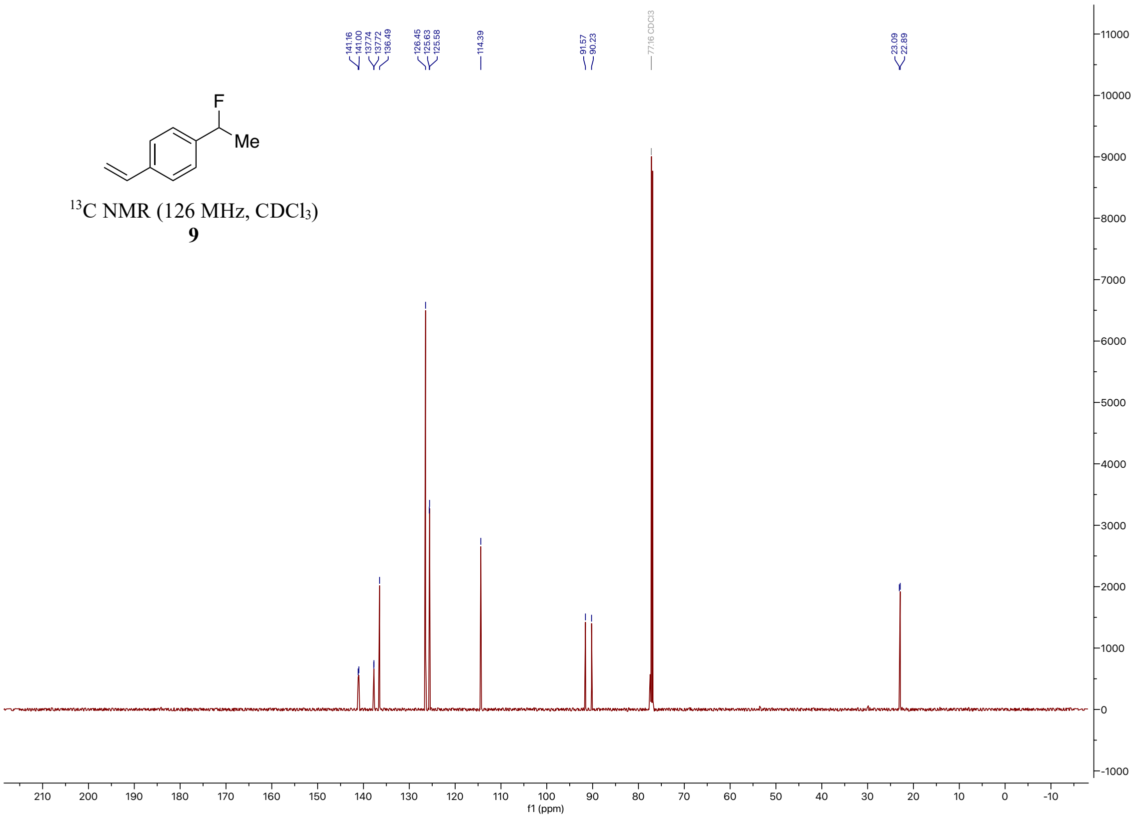
F
Me
^{13}C NMR (126 MHz, CDCl$_3$)
9
141.16
141.00
137.74
137.72
136.49
126.45
125.63
125.58
114.39
91.57
90.23
77.16 CDCl3
23.09
22.89
f1 (ppm)

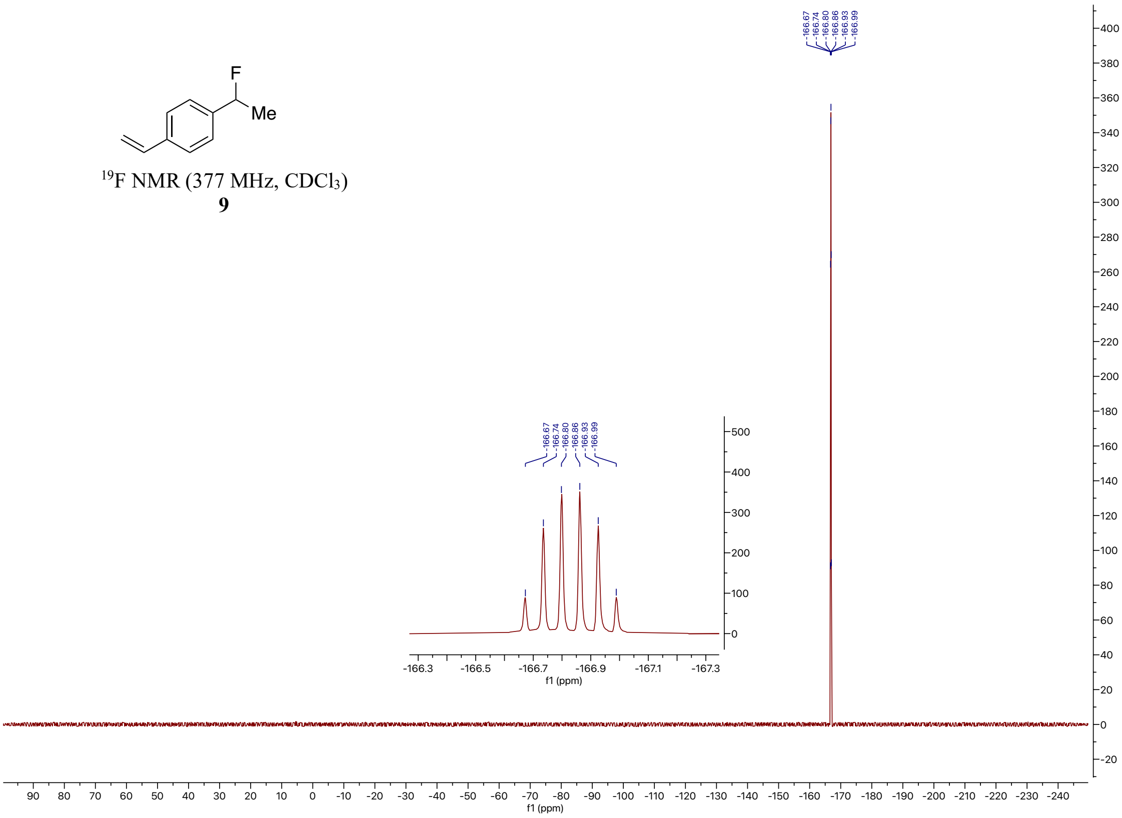
F
Me
^{19}F NMR (377 MHz, CDCl$_3$)
9
-166.67
-166.74
-166.80
-166.86
-166.93
-166.99
f1 (ppm)

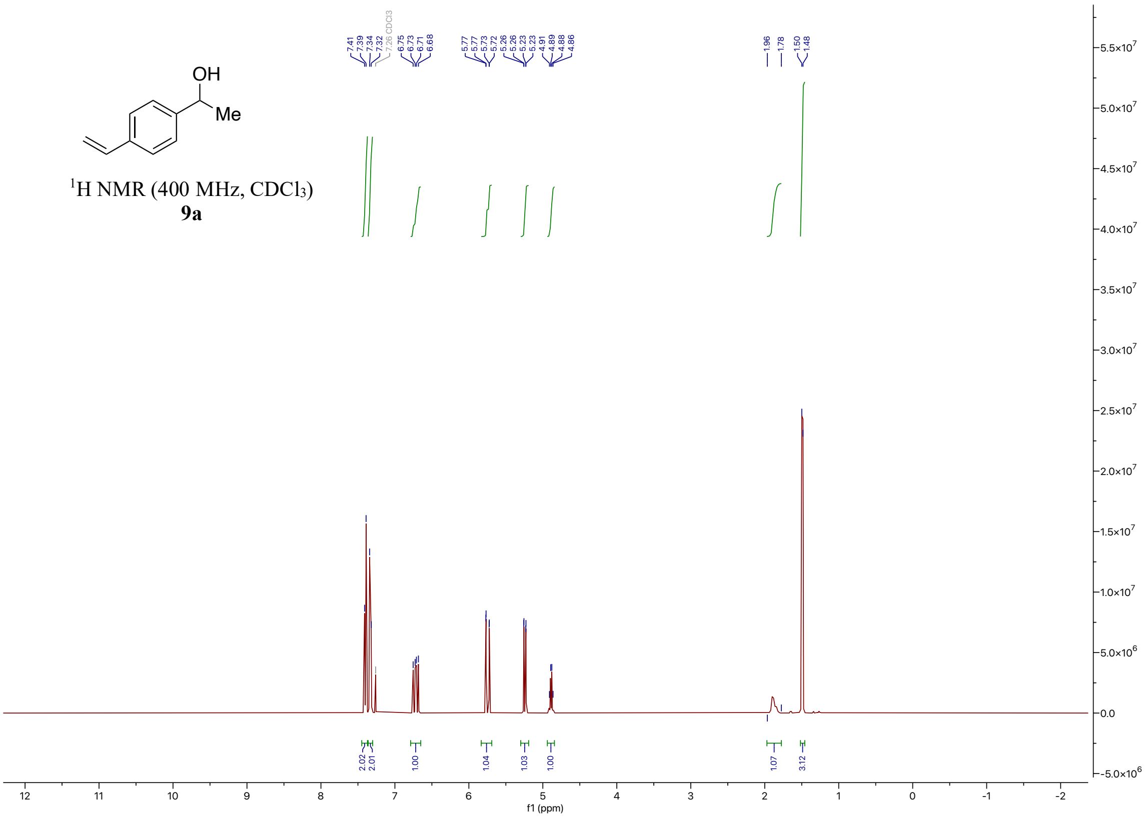
OH
Me
^{1}H NMR (400 MHz, CDCl$_3$)
9a
f1 (ppm)

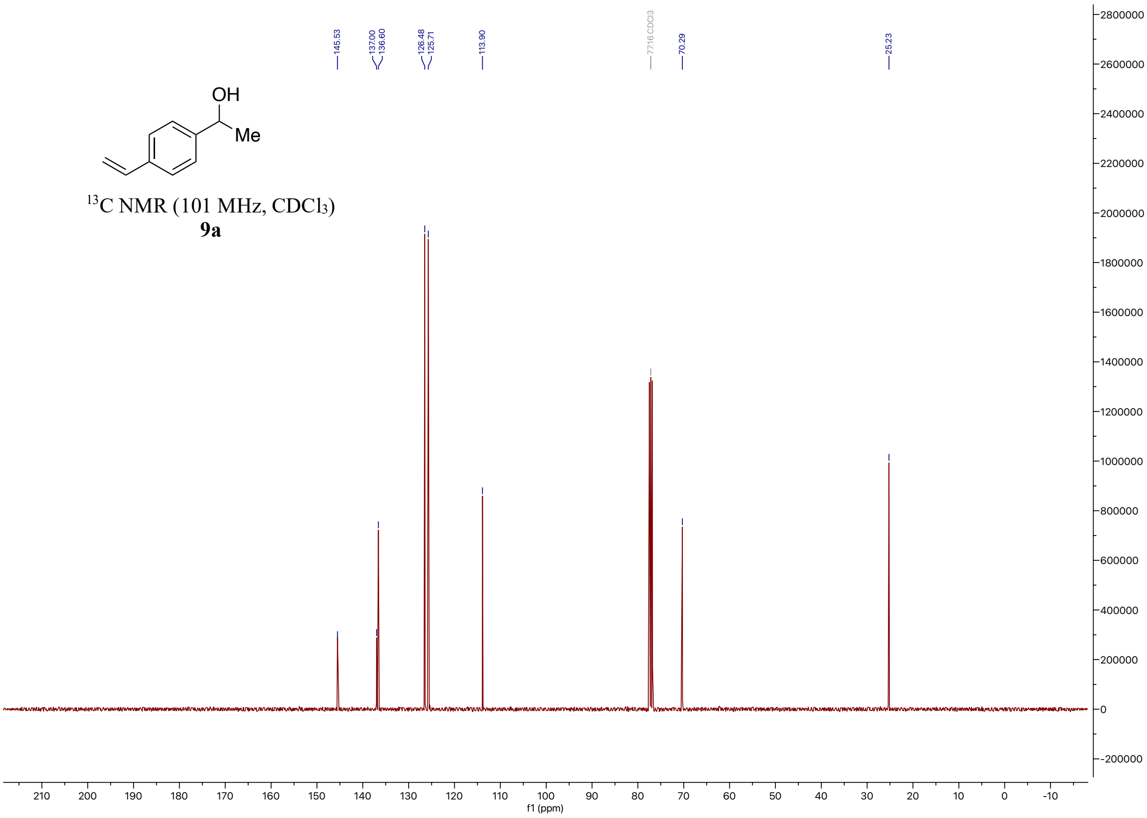
OH
Me
^{13}C NMR (101 MHz, CDCl$_3$)
9a
f1 (ppm)

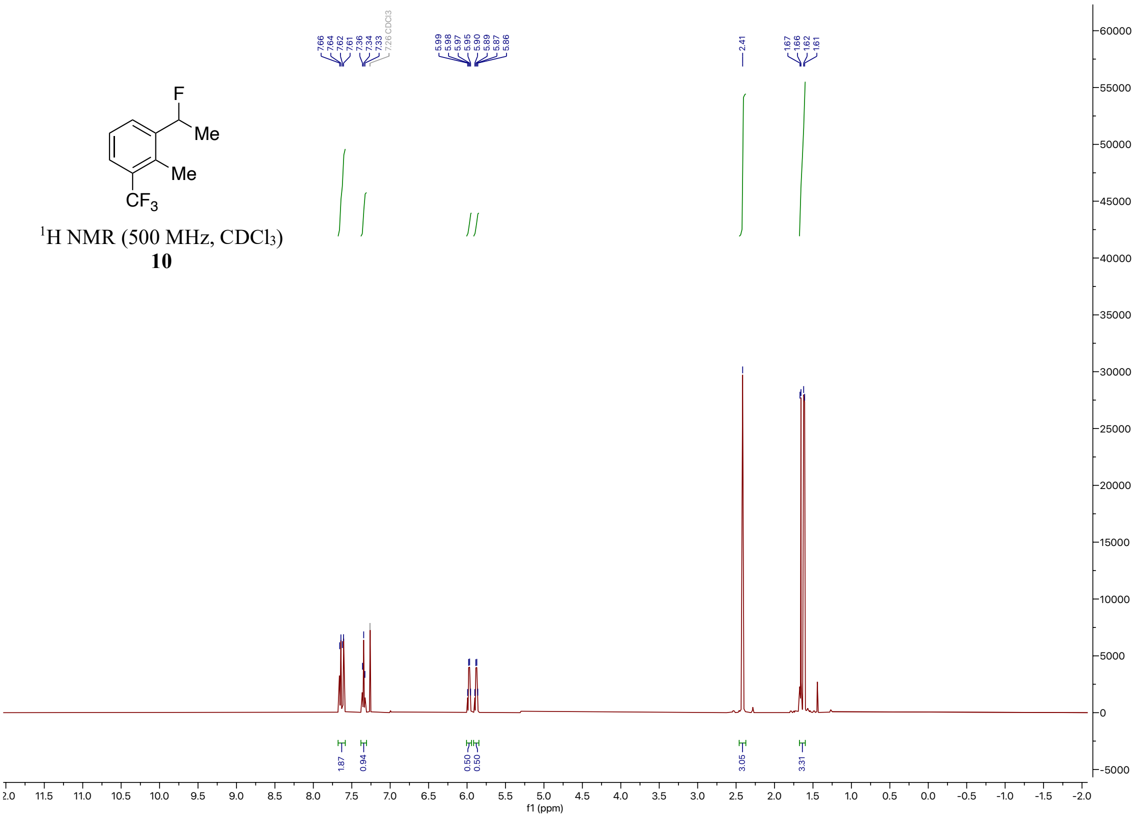

^{1}H NMR (500 MHz, $CDCl_3$)
10

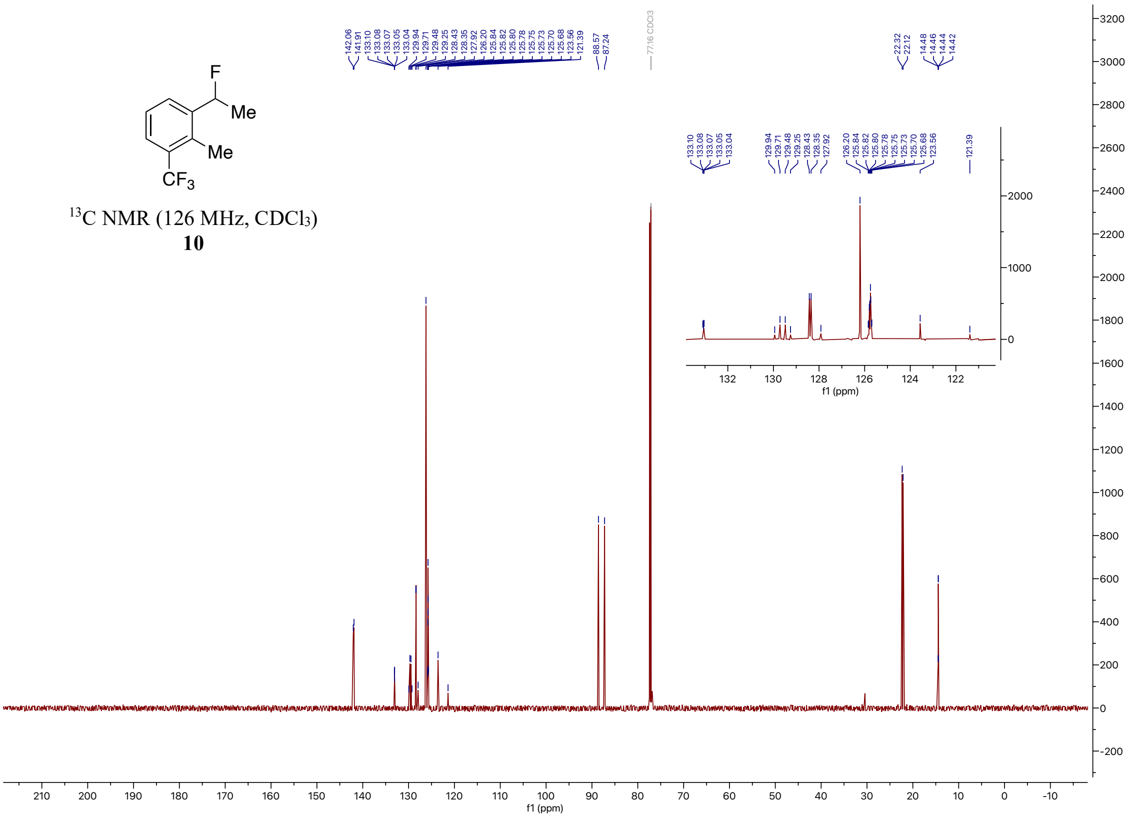

^{13}C NMR (126 MHz, $CDCl_3$)
10

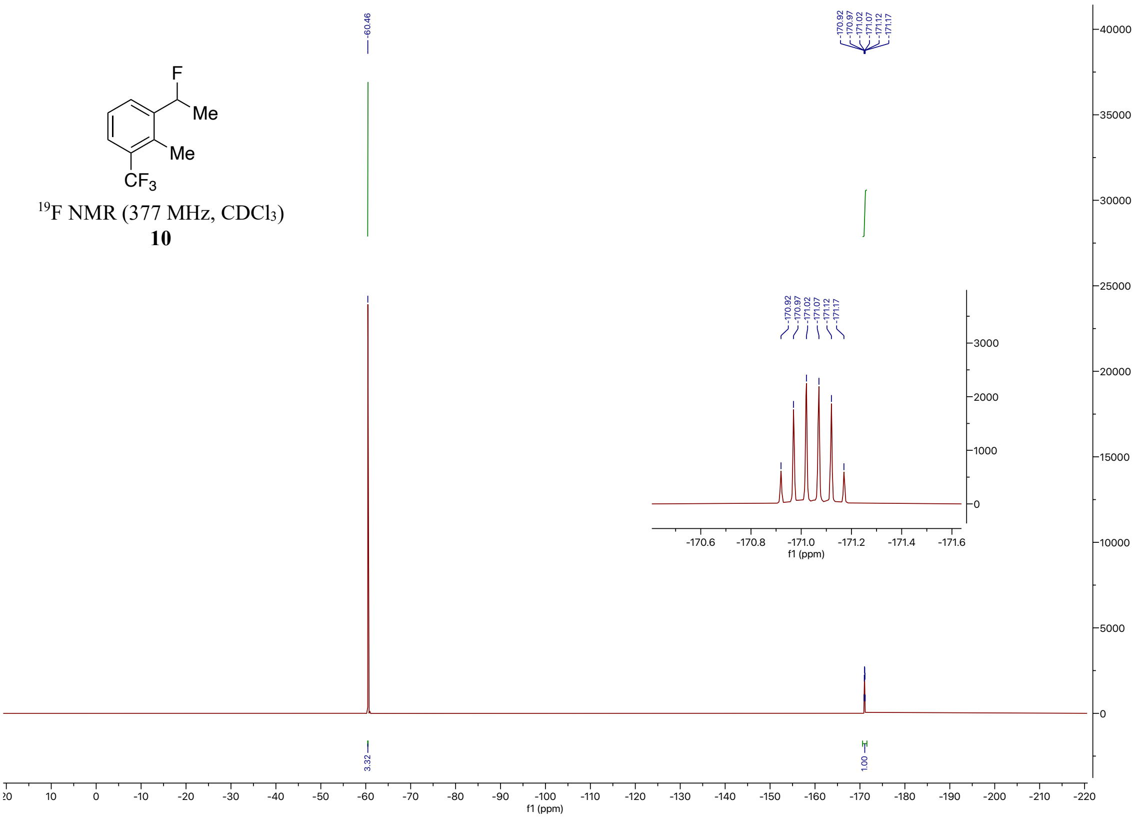

^{19}F NMR (377 MHz, $CDCl_3$)
10

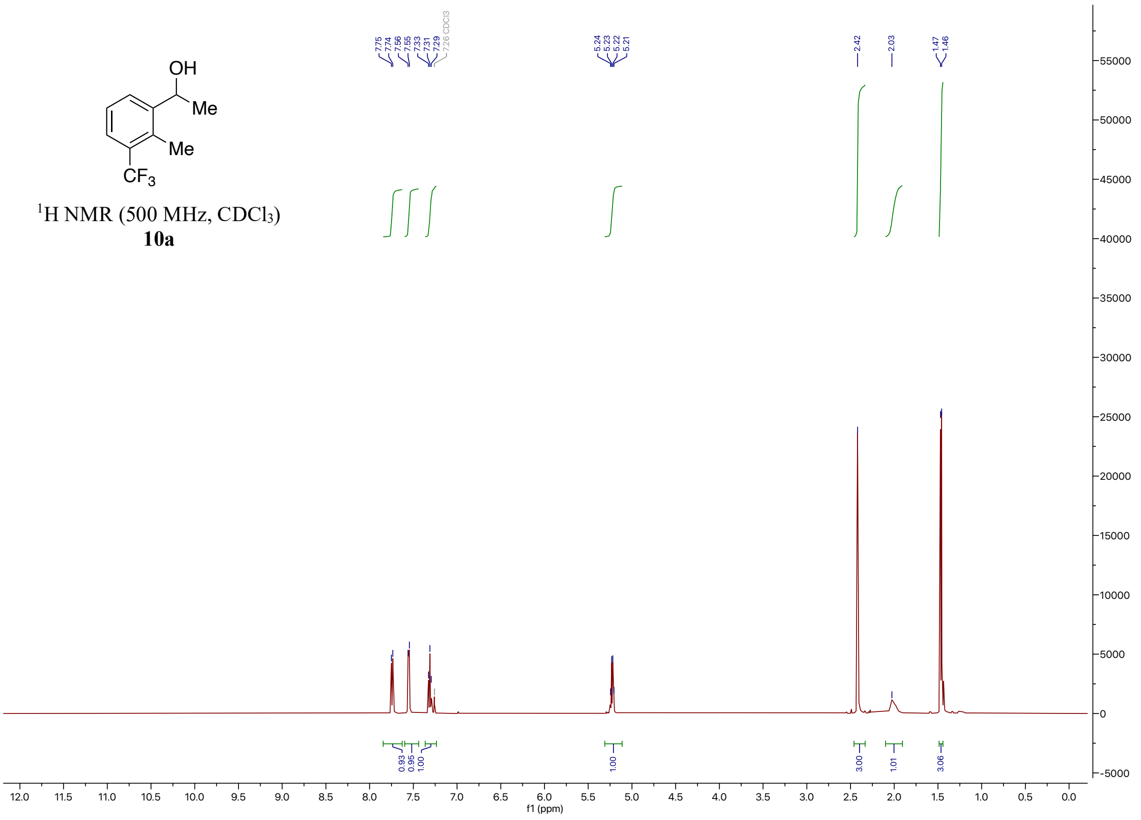

^{1}H NMR (500 MHz, $CDCl_3$)
10a

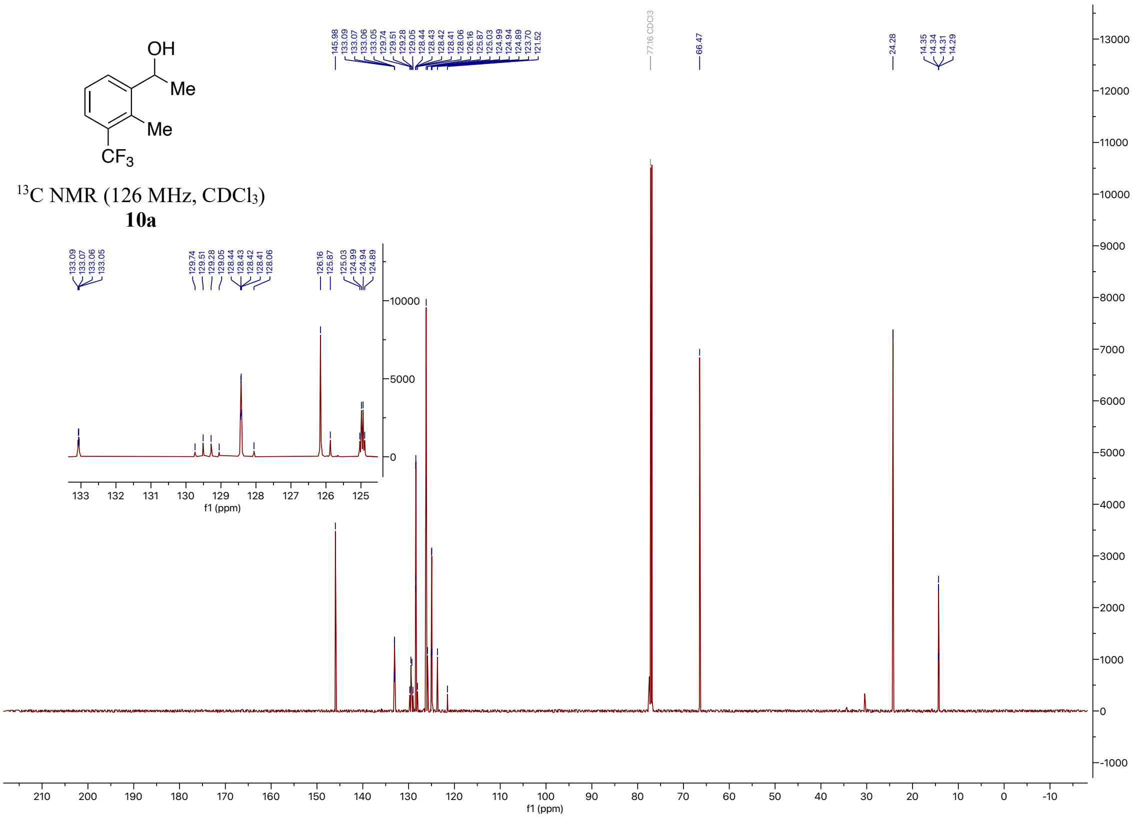

^{13}C NMR (126 MHz, $CDCl_3$)
10a

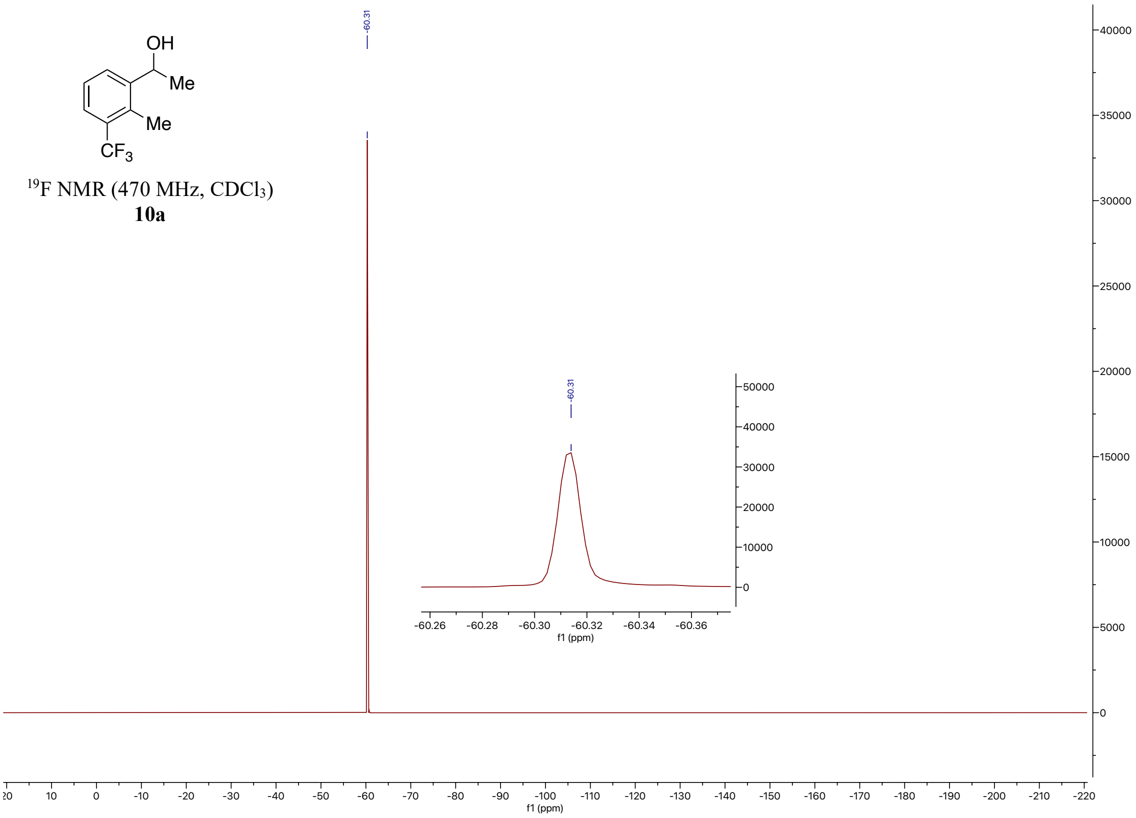

^{19}F NMR (470 MHz, $CDCl_3$)
10a

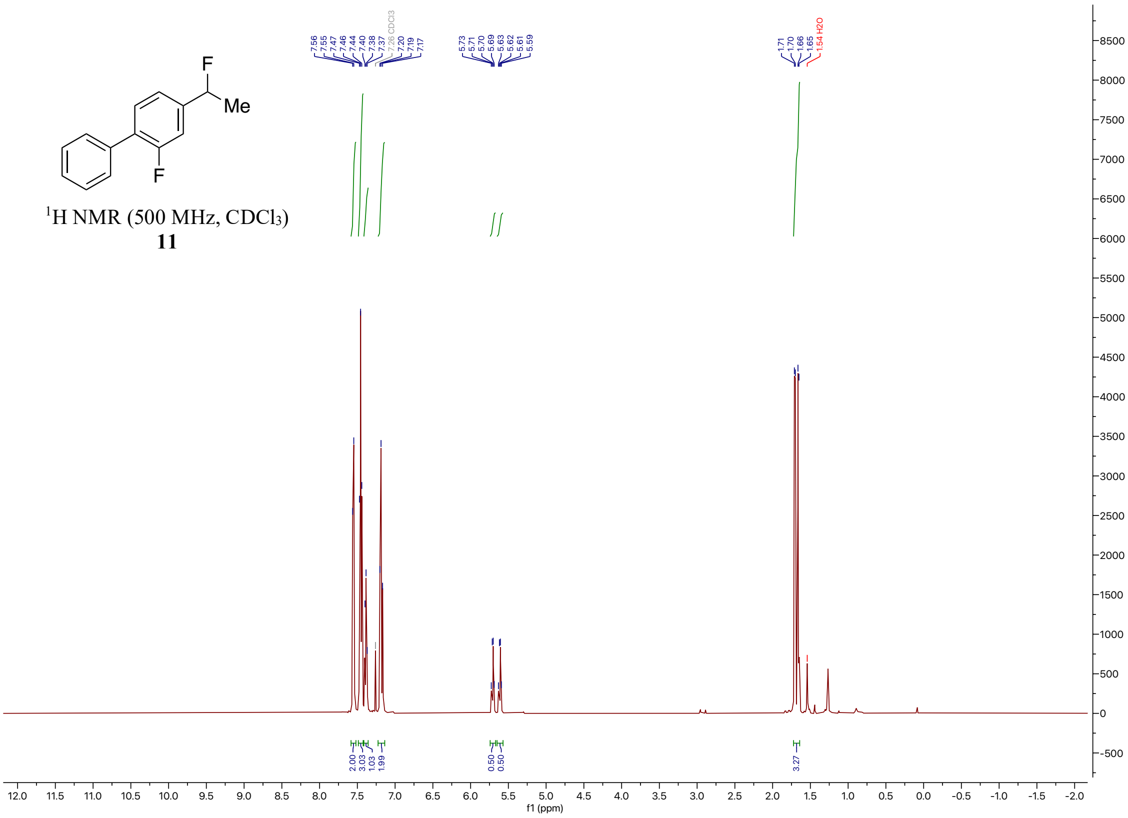

^{1}H NMR (500 MHz, $CDCl_3$)

11

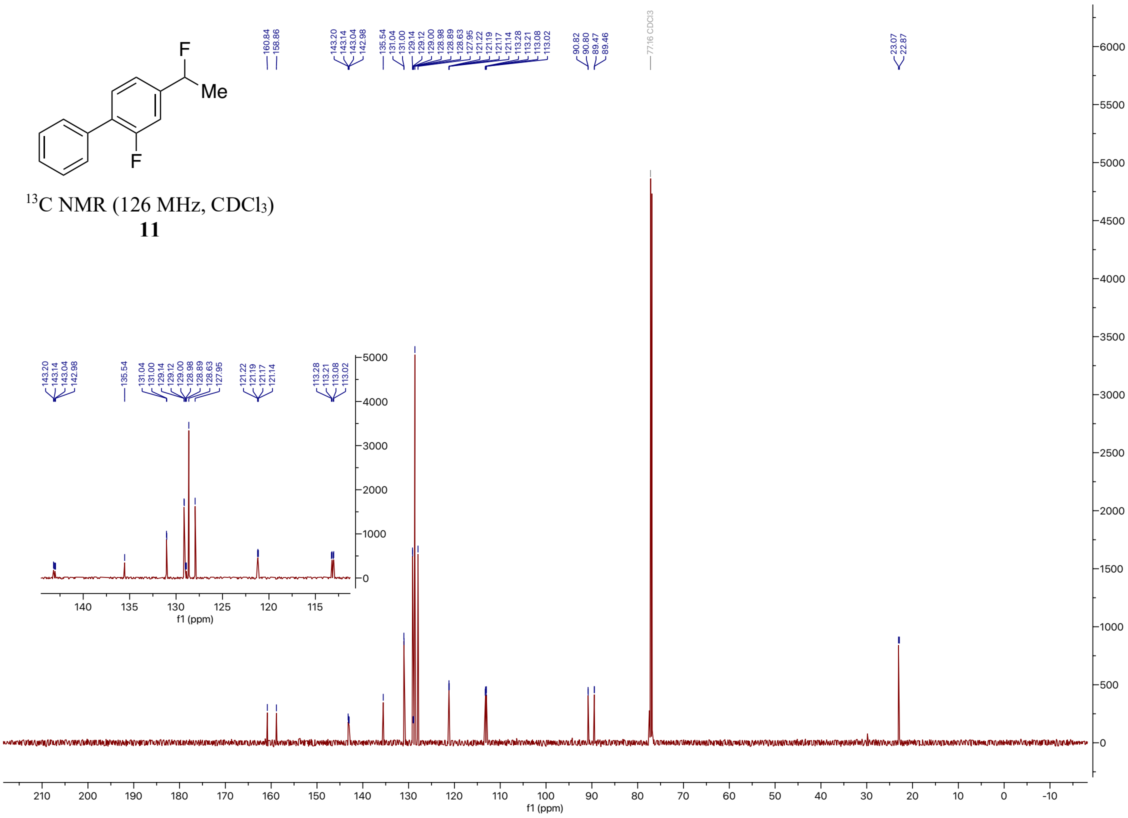

^{13}C NMR (126 MHz, $CDCl_3$)

11

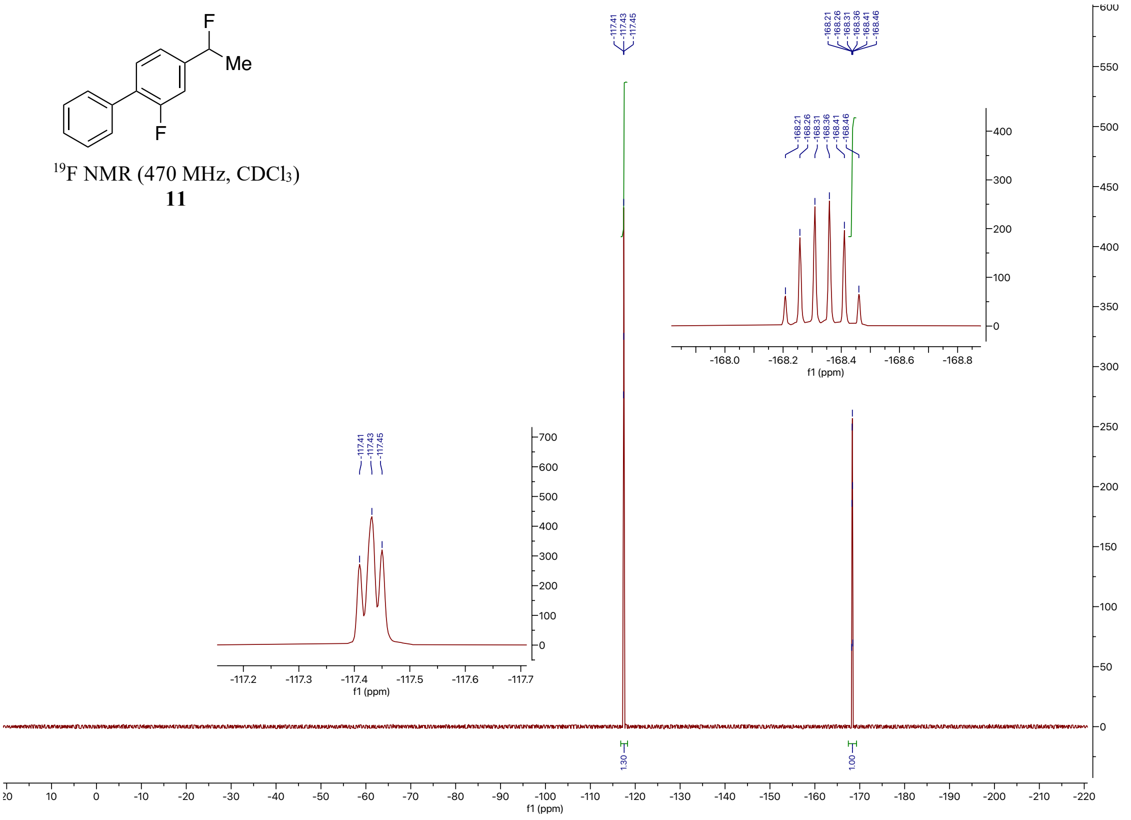

^{19}F NMR (470 MHz, CDCl$_3$)
11

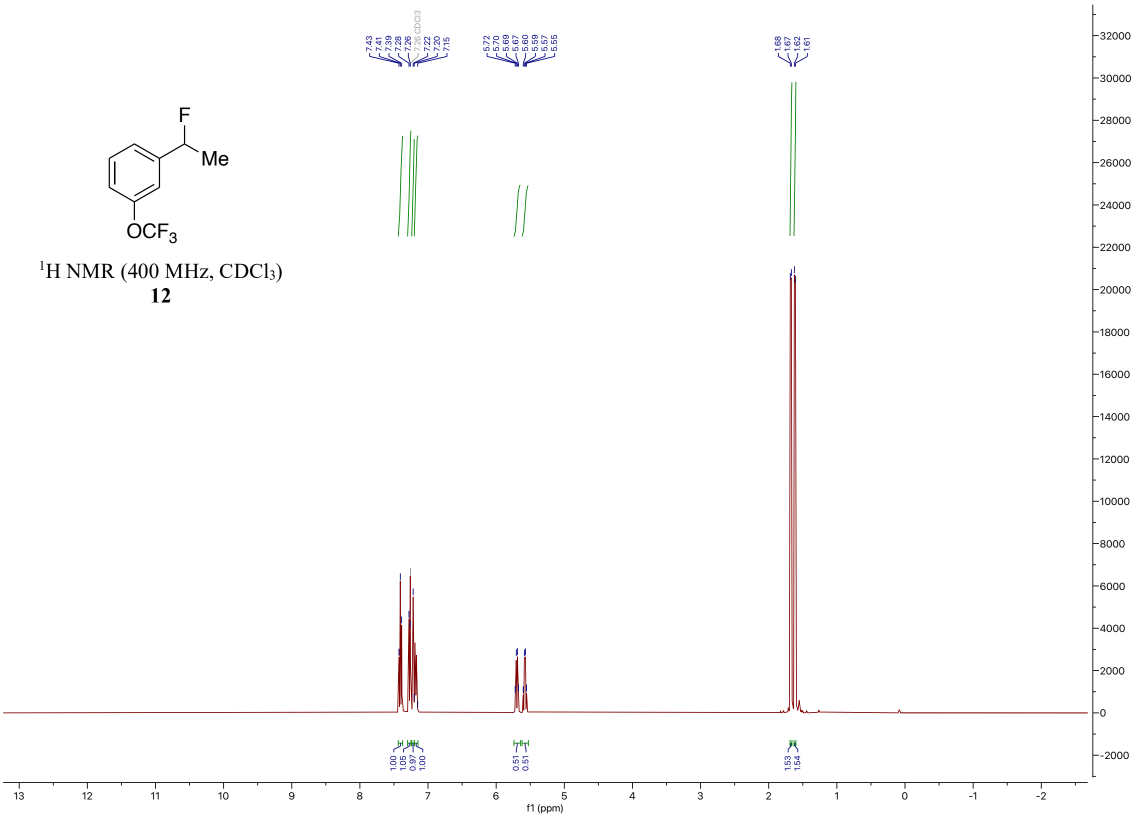

^{1}H NMR (400 MHz, CDCl$_3$)
12

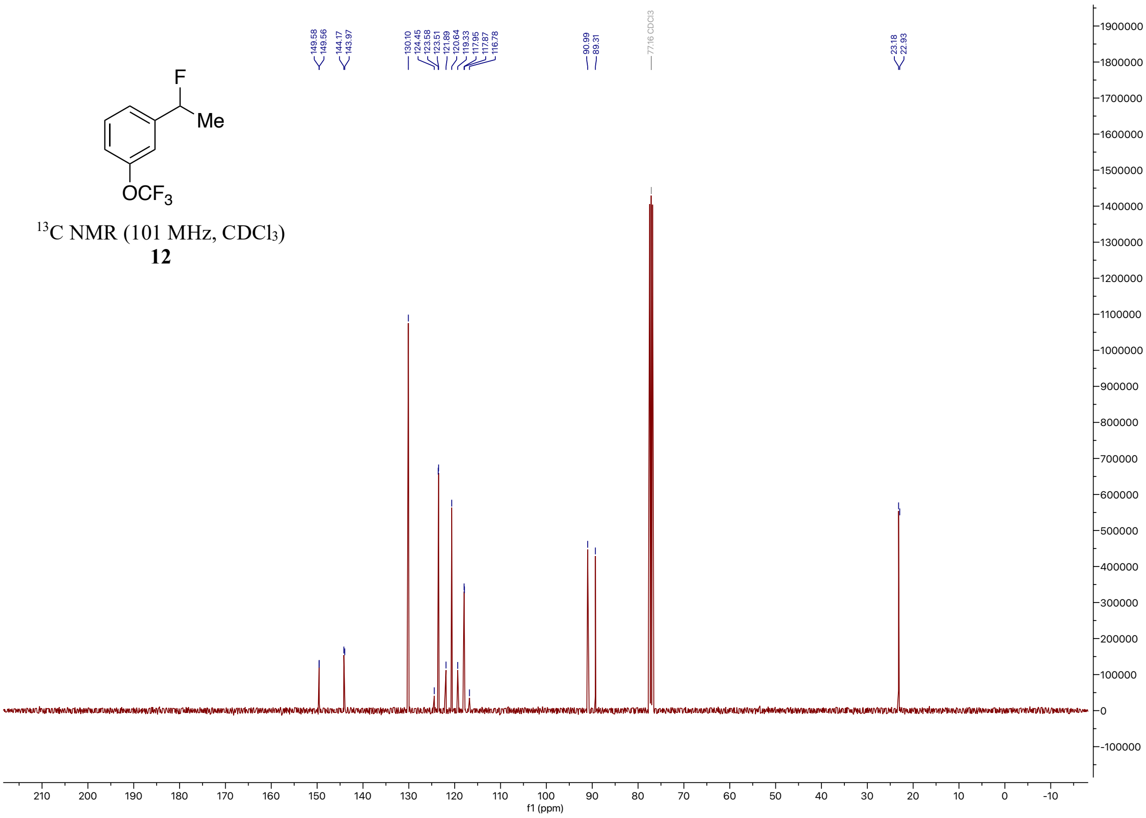

^{13}C NMR (101 MHz, $CDCl_3$)
12

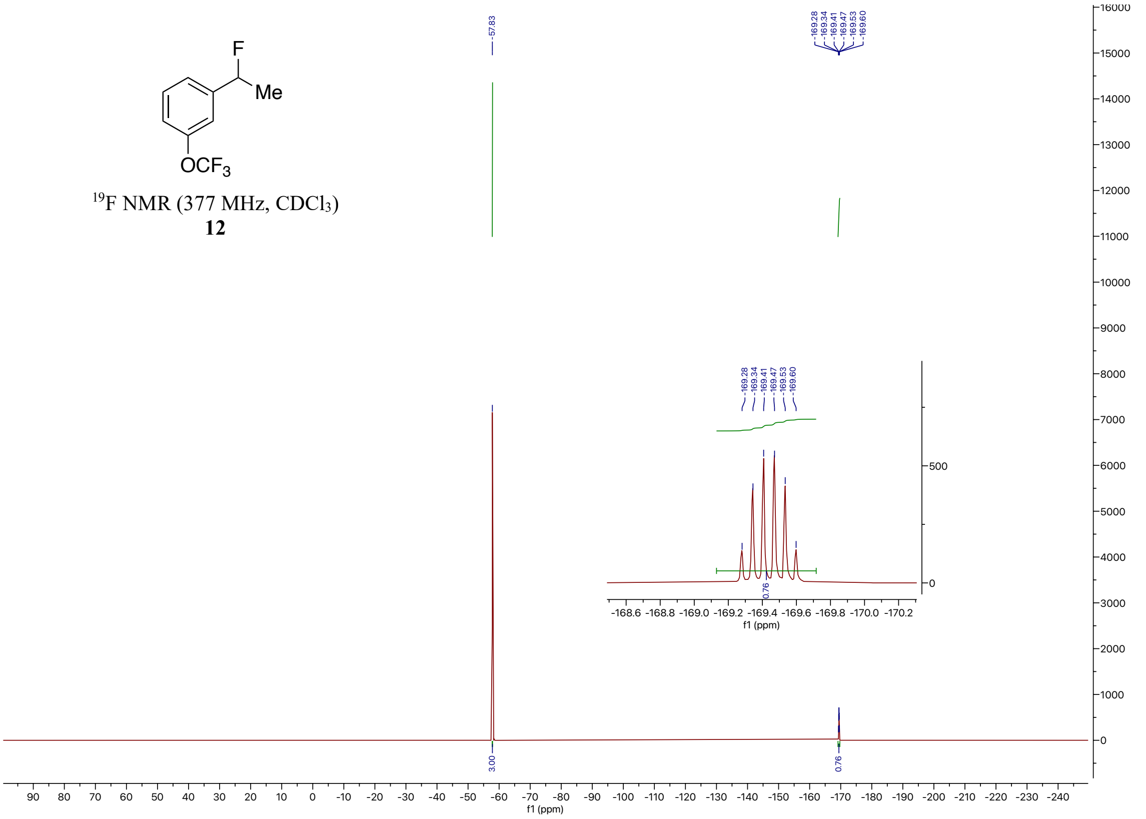

^{19}F NMR (377 MHz, $CDCl_3$)
12

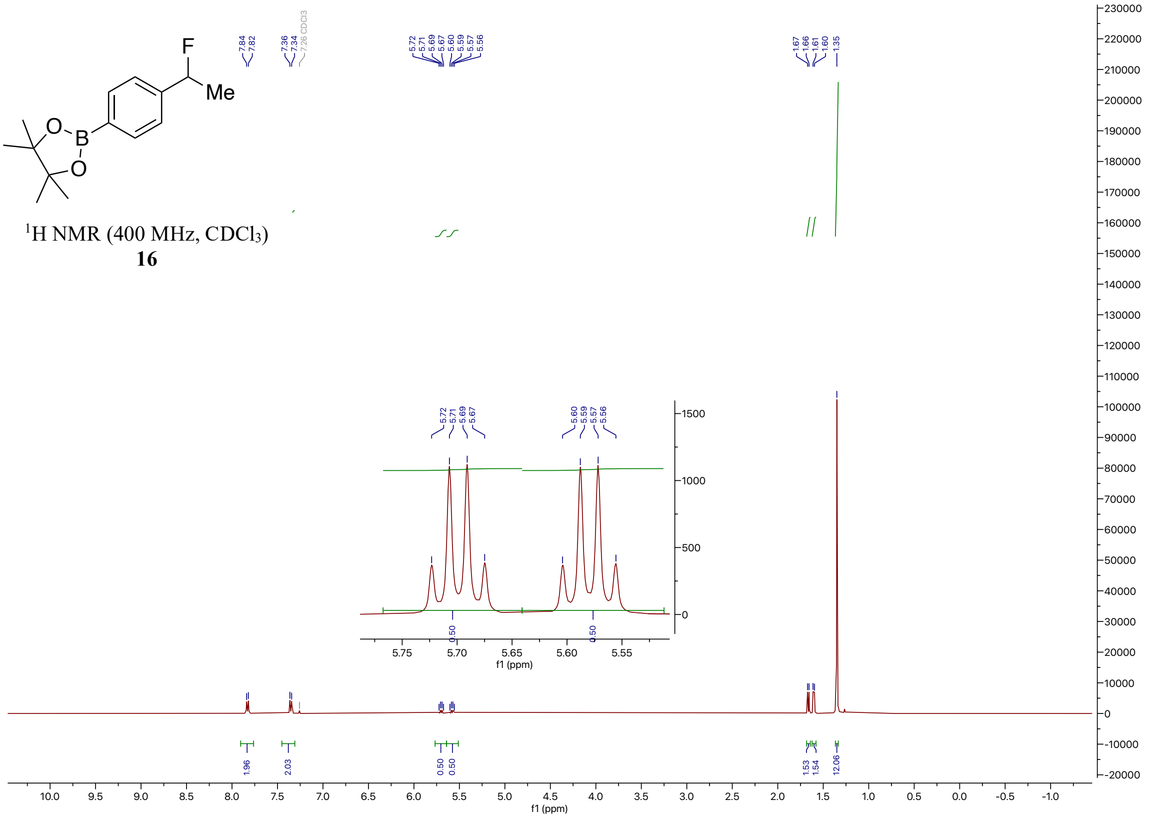

^{1}H NMR (400 MHz, $CDCl_3$)
16

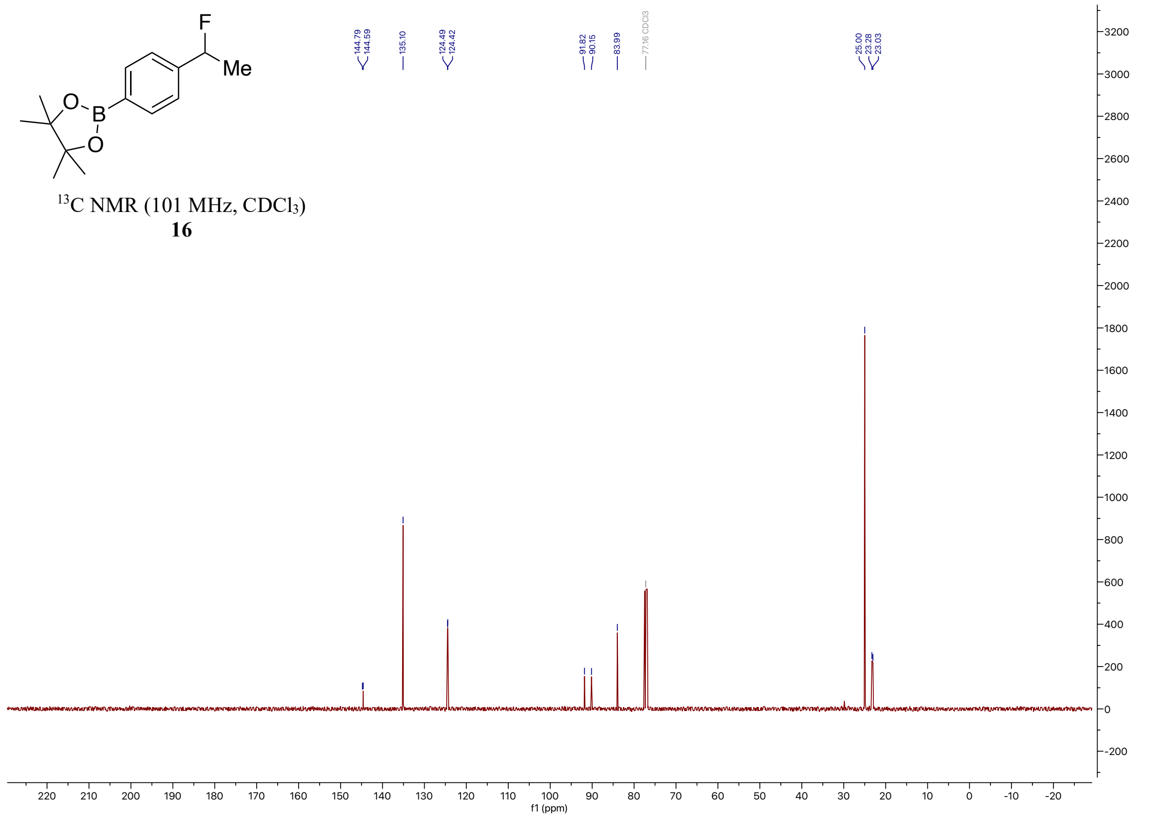

^{13}C NMR (101 MHz, $CDCl_3$)
16

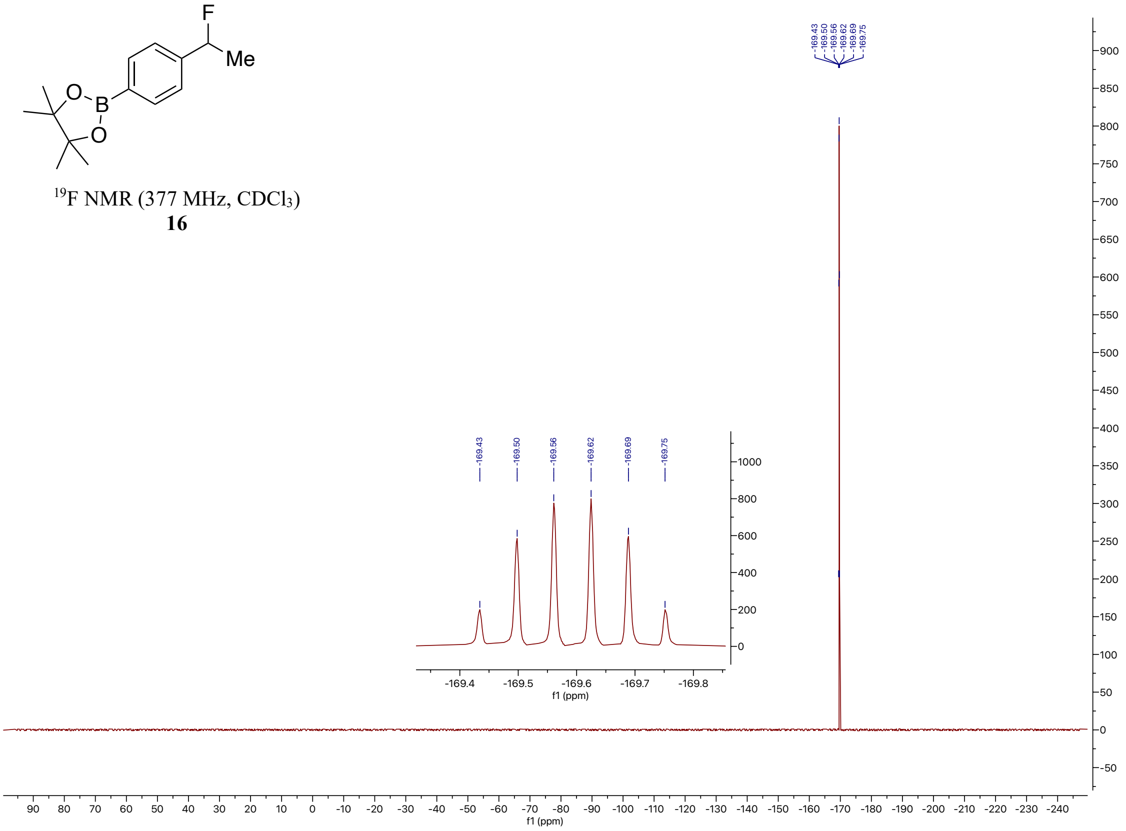

^{19}F NMR (377 MHz, $CDCl_3$)
16

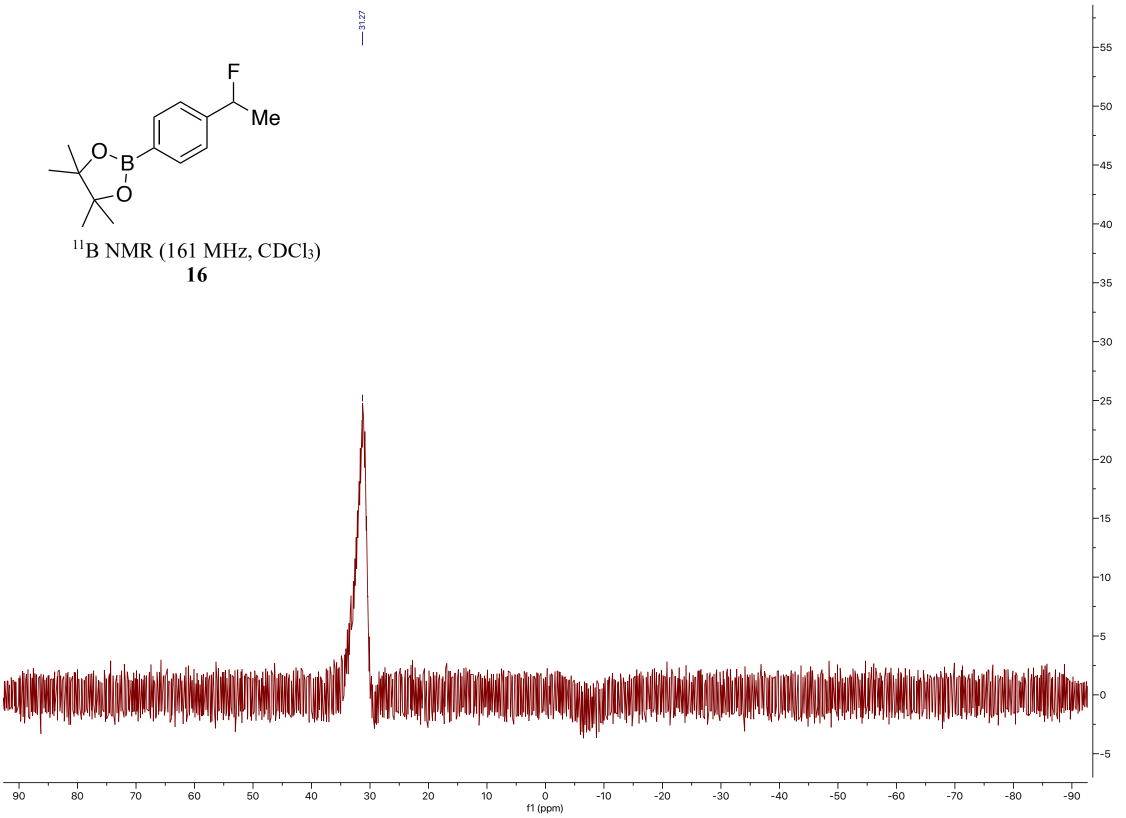

^{11}B NMR (161 MHz, $CDCl_3$)
16

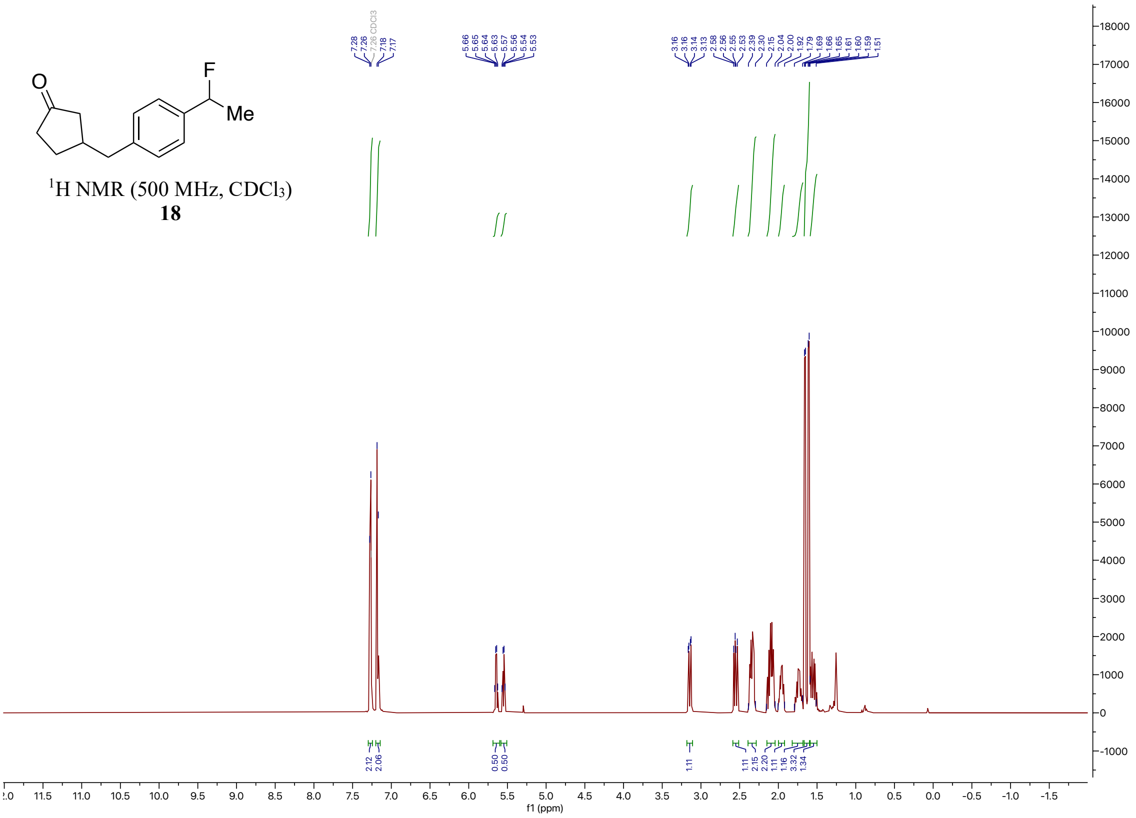

^{1}H NMR (500 MHz, $CDCl_3$)
18

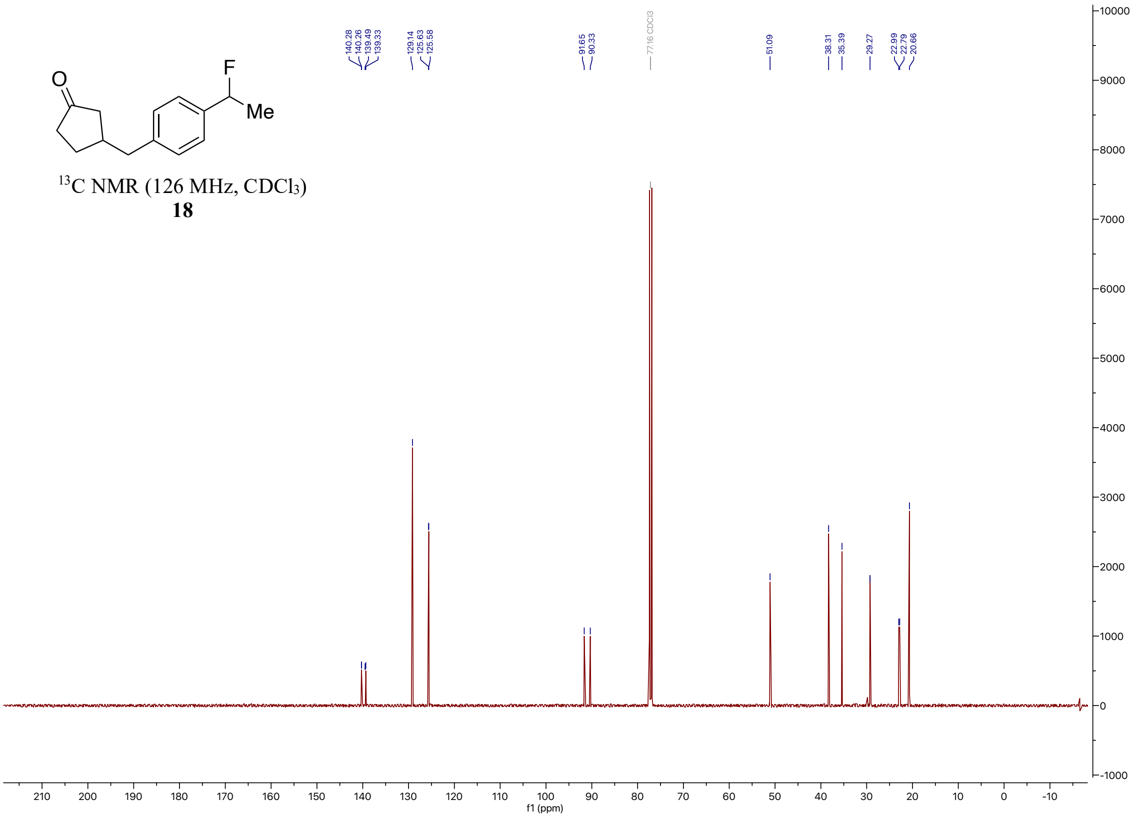

^{13}C NMR (126 MHz, $CDCl_3$)
18

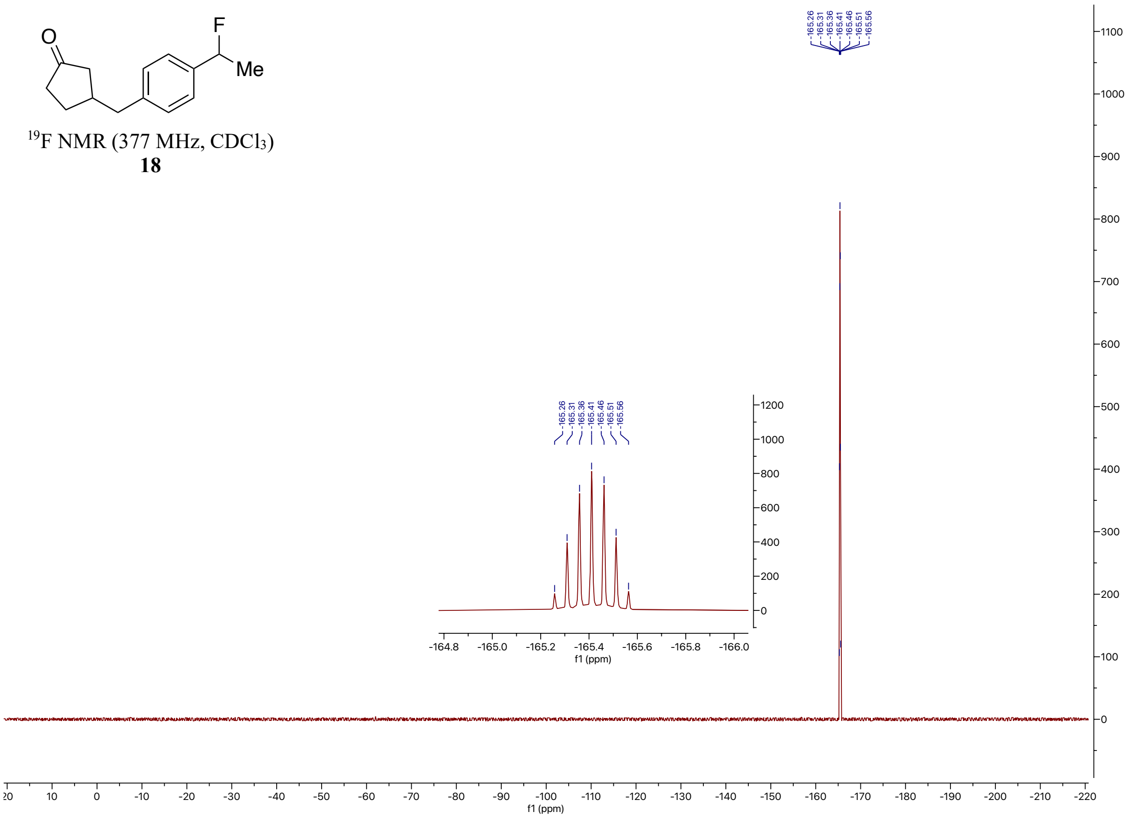

^{19}F NMR (377 MHz, $CDCl_3$)
18

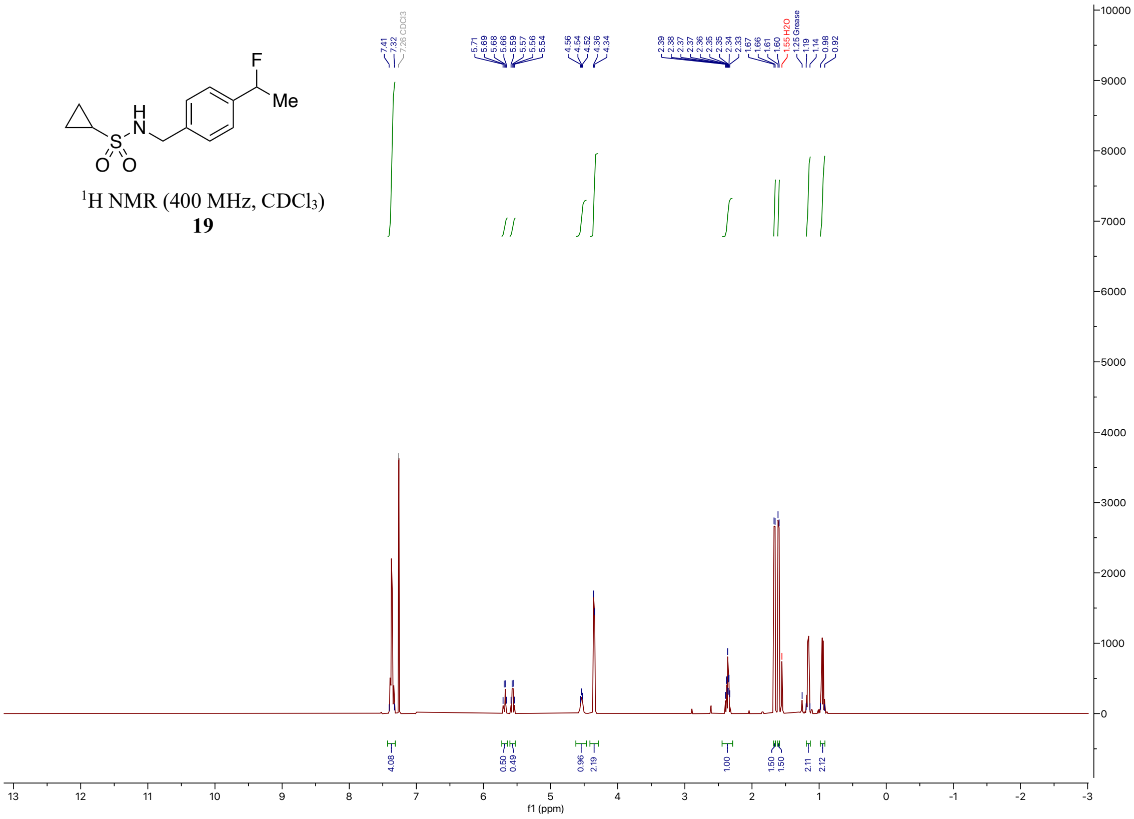

^{1}H NMR (400 MHz, $CDCl_3$)
19

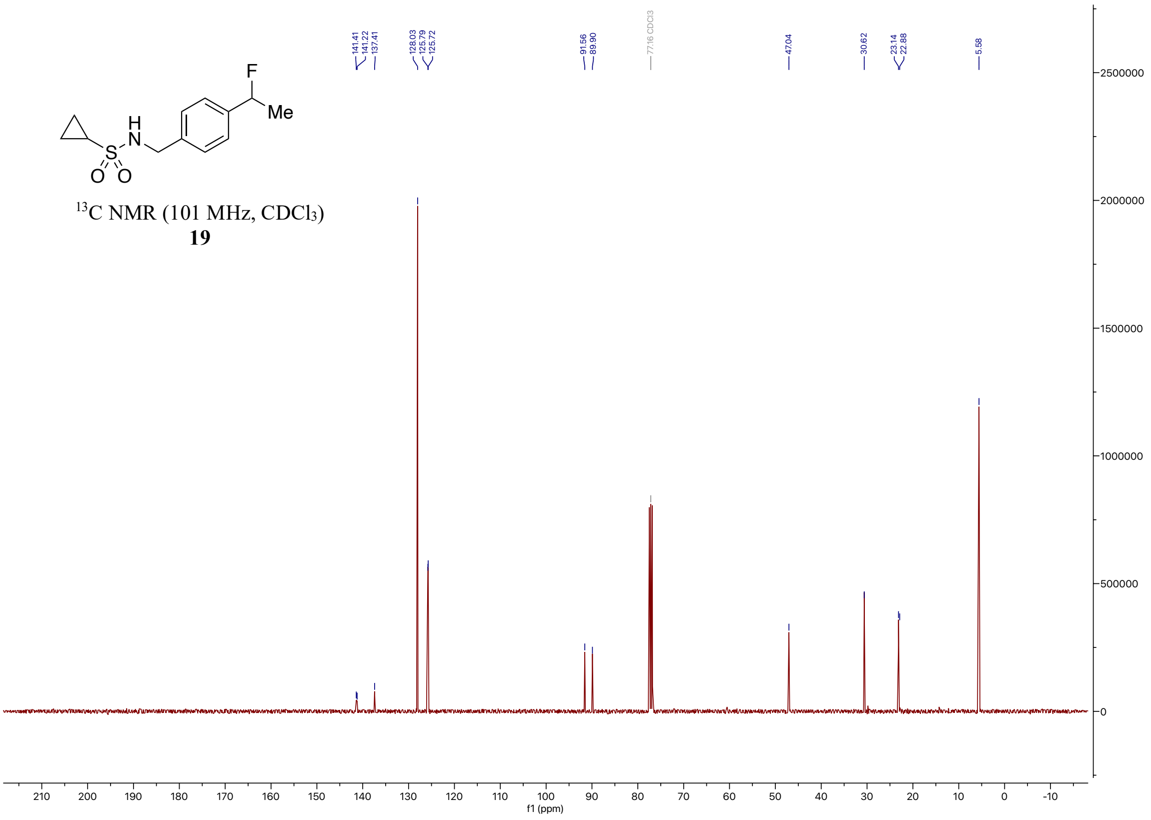

^{13}C NMR (101 MHz, $CDCl_3$)
19

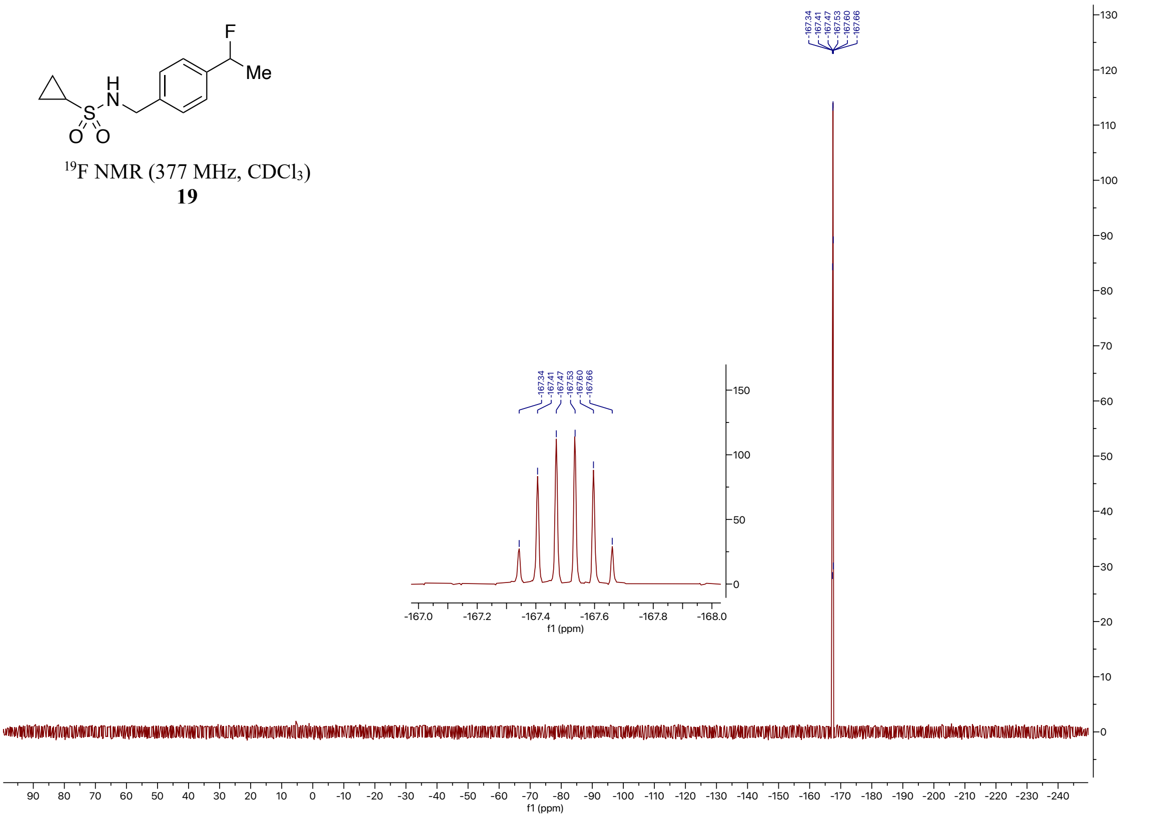

^{19}F NMR (377 MHz, $CDCl_3$)
19

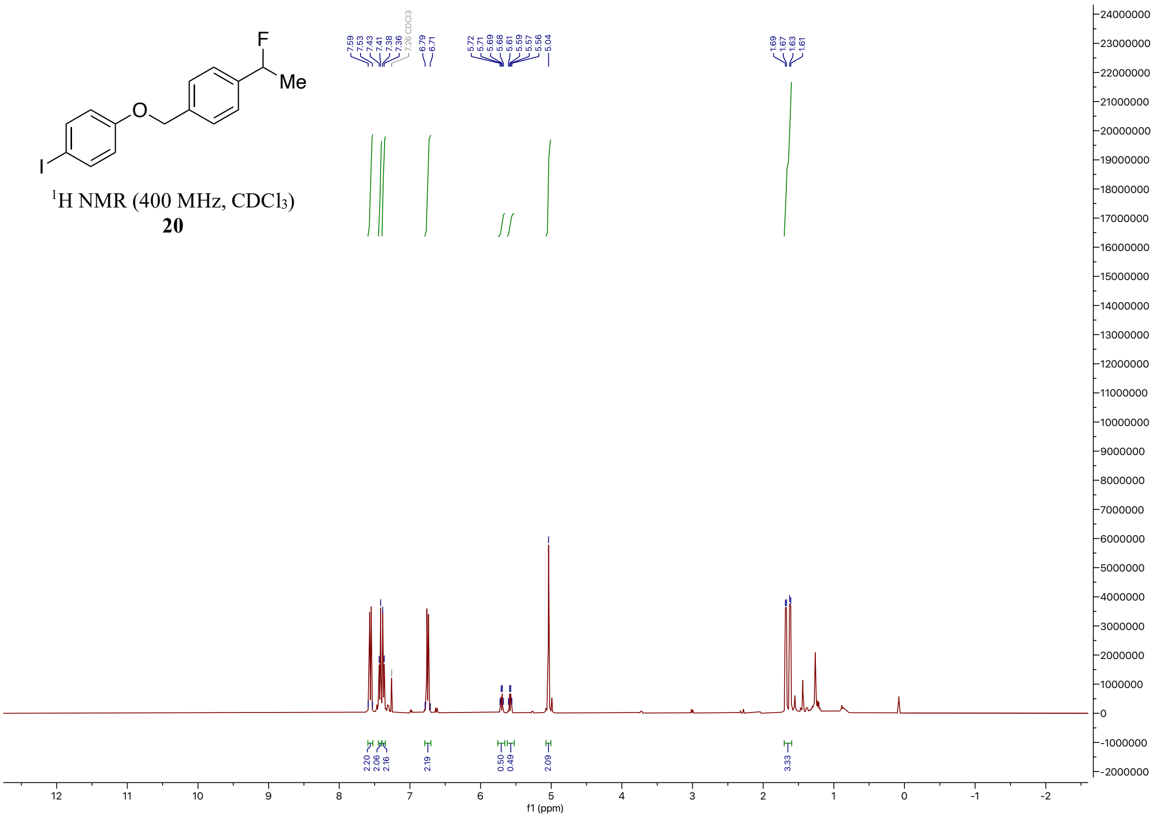
F
Me
O
I
^{1}H NMR (400 MHz, CDCl$_3$)
20
f1 (ppm)

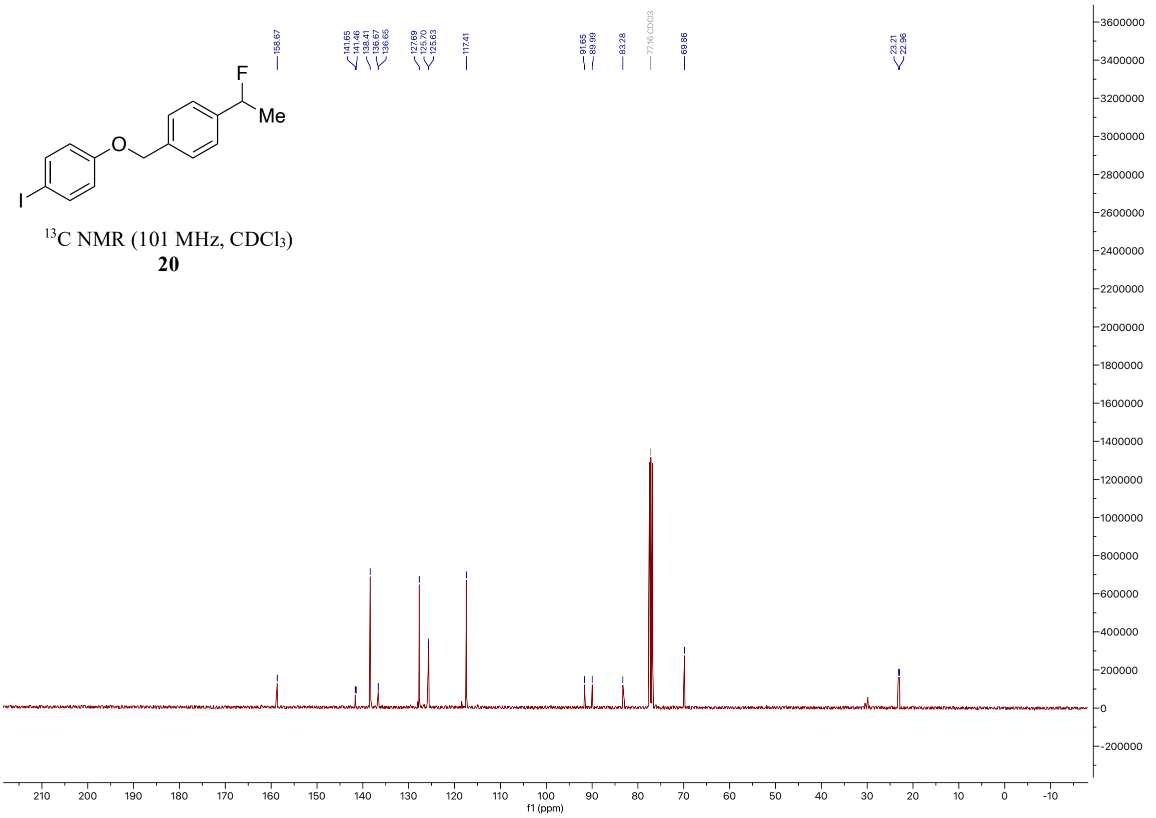
F
Me
O
I
^{13}C NMR (101 MHz, CDCl$_3$)
20
f1 (ppm)

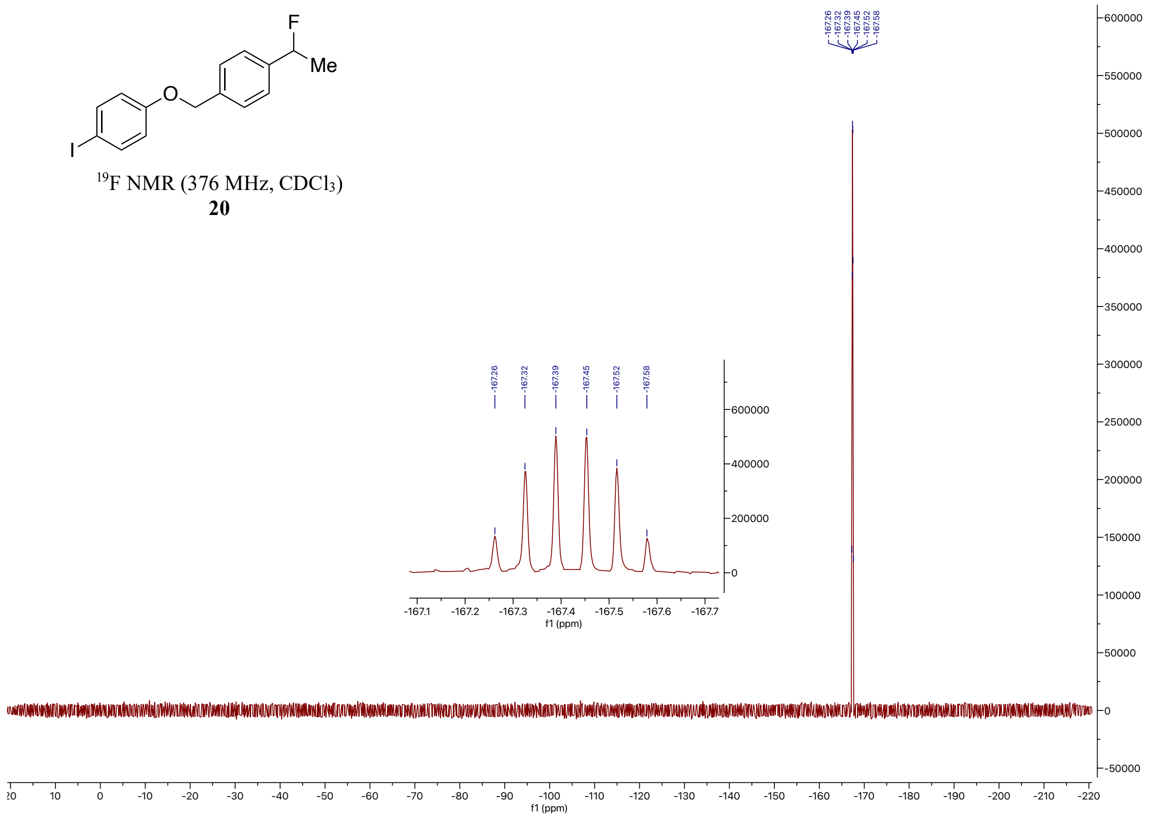

^{19}F NMR (376 MHz, CDCl$_3$)
20

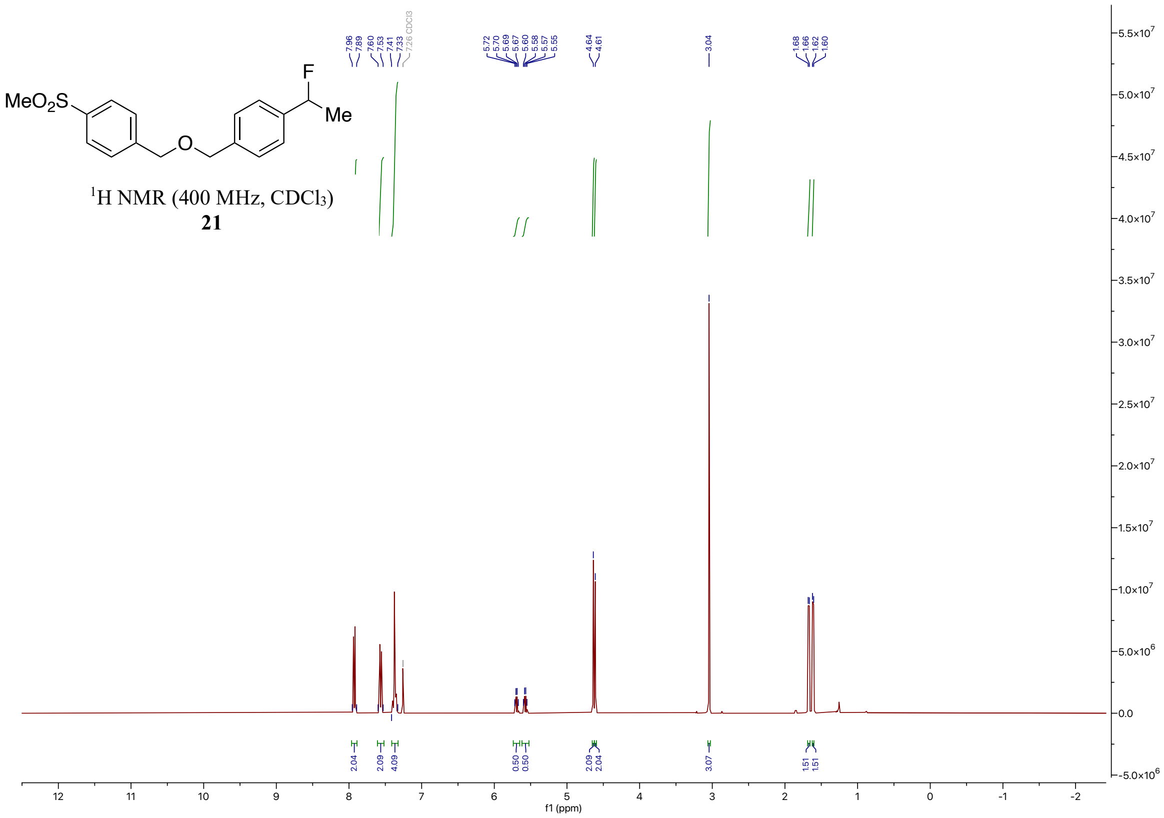

^{1}H NMR (400 MHz, CDCl$_3$)
21

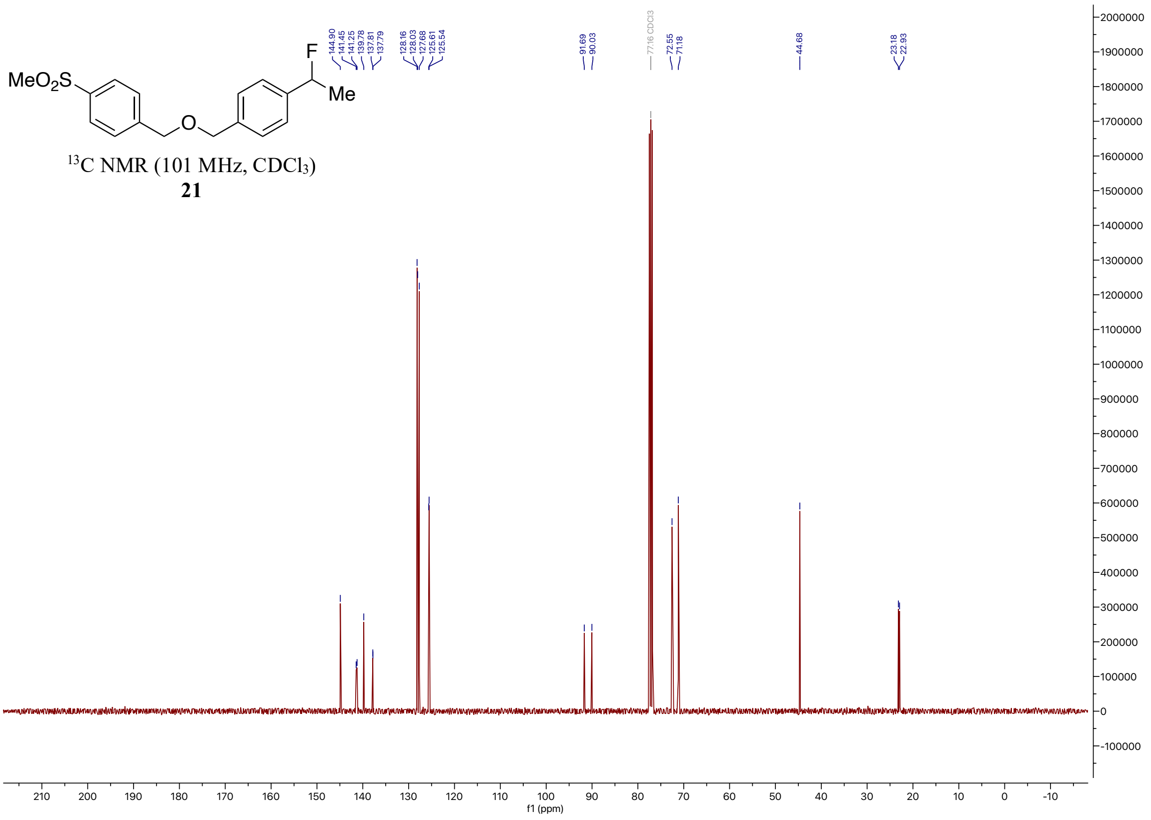

^{13}C NMR (101 MHz, $CDCl_3$)
21

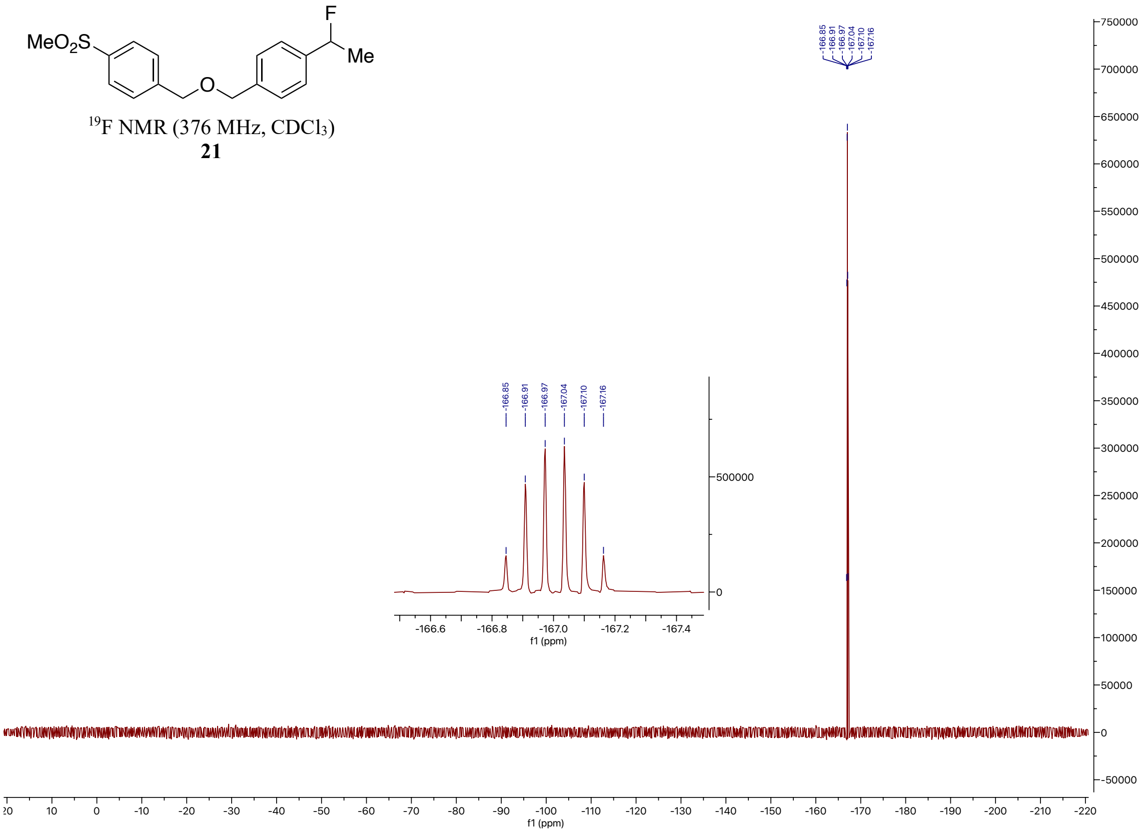

^{19}F NMR (376 MHz, $CDCl_3$)
21

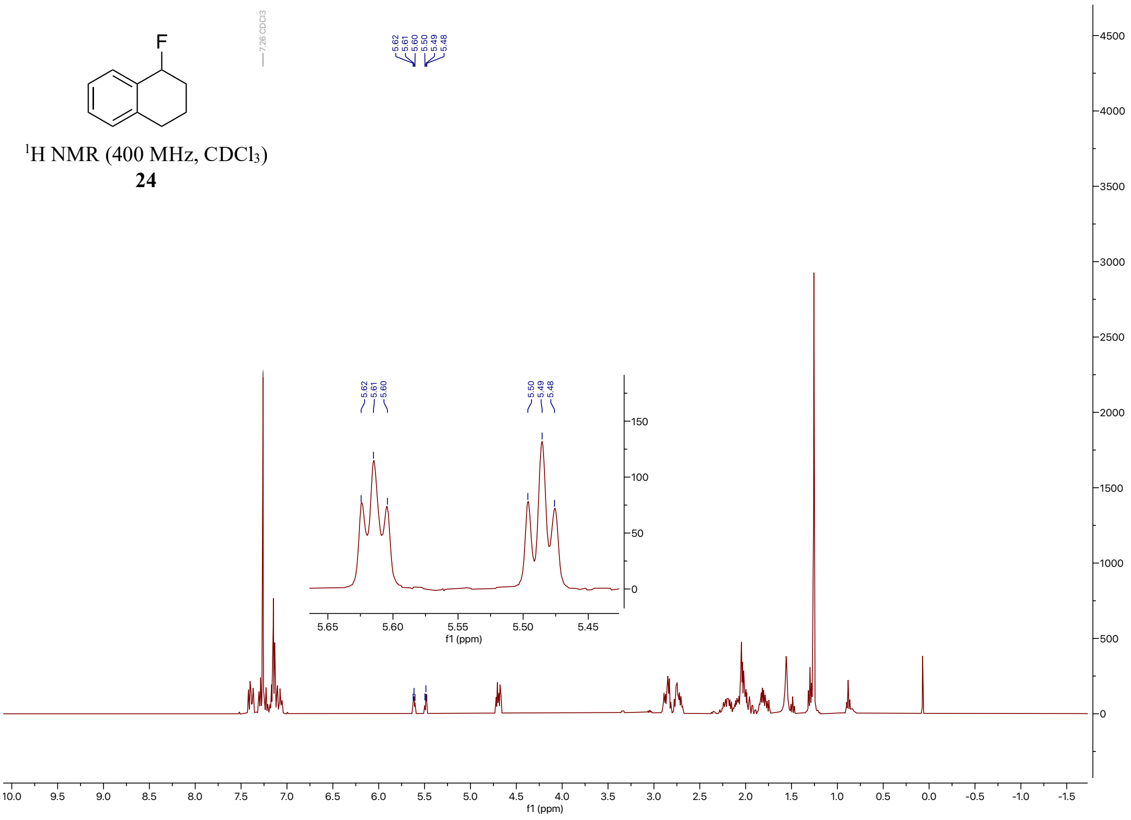

^{1}H NMR (400 MHz, $CDCl_3$)
24

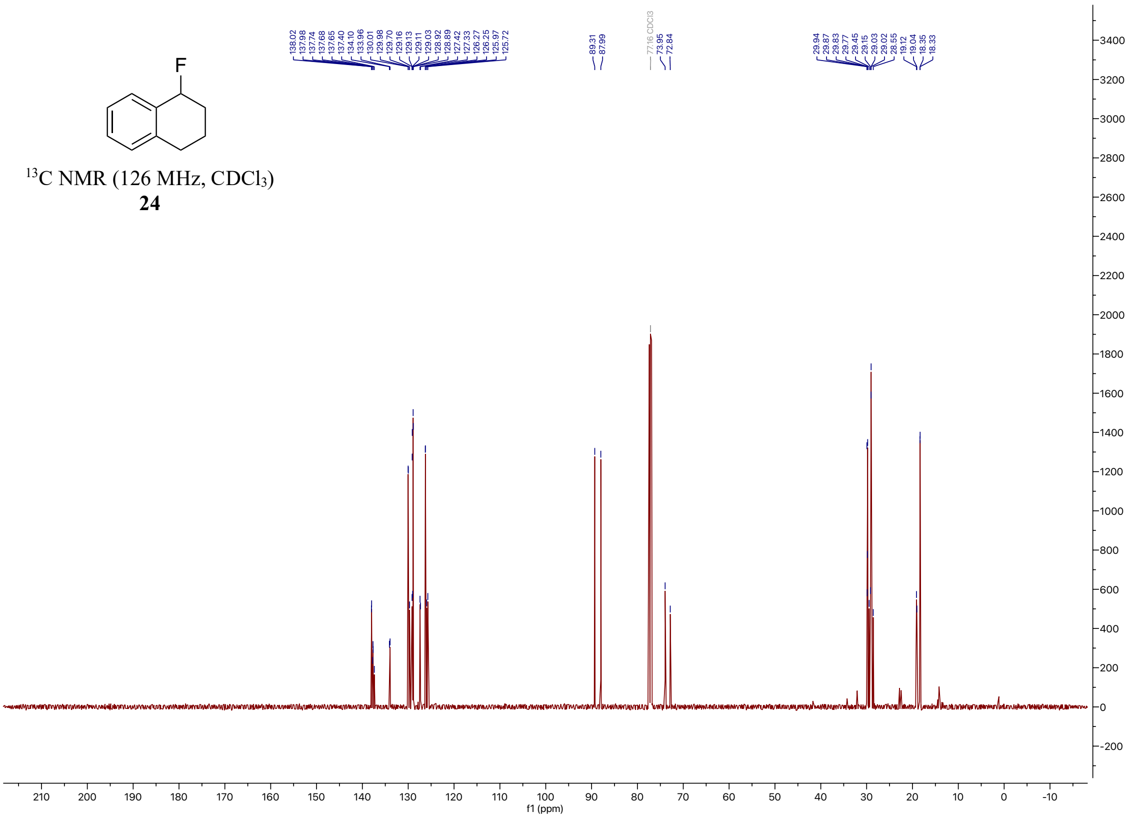

^{13}C NMR (126 MHz, $CDCl_3$)
24

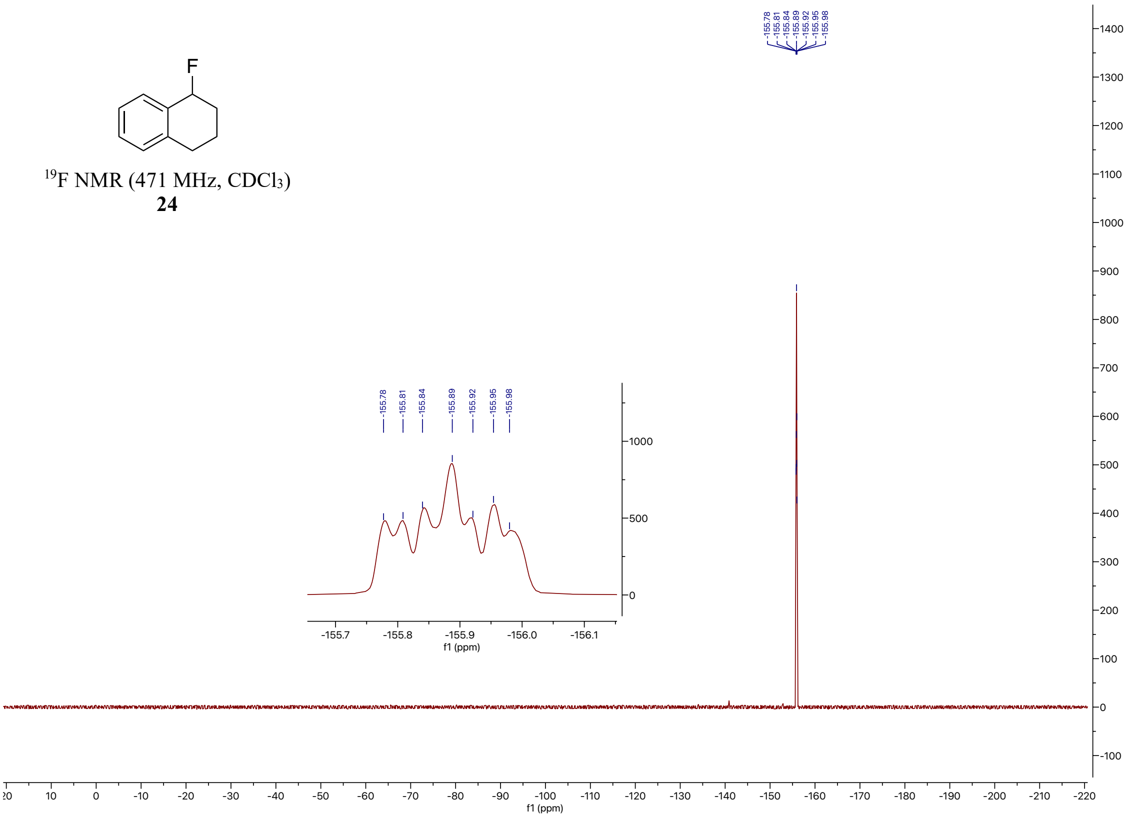

^{19}F NMR (471 MHz, $CDCl_3$)
24

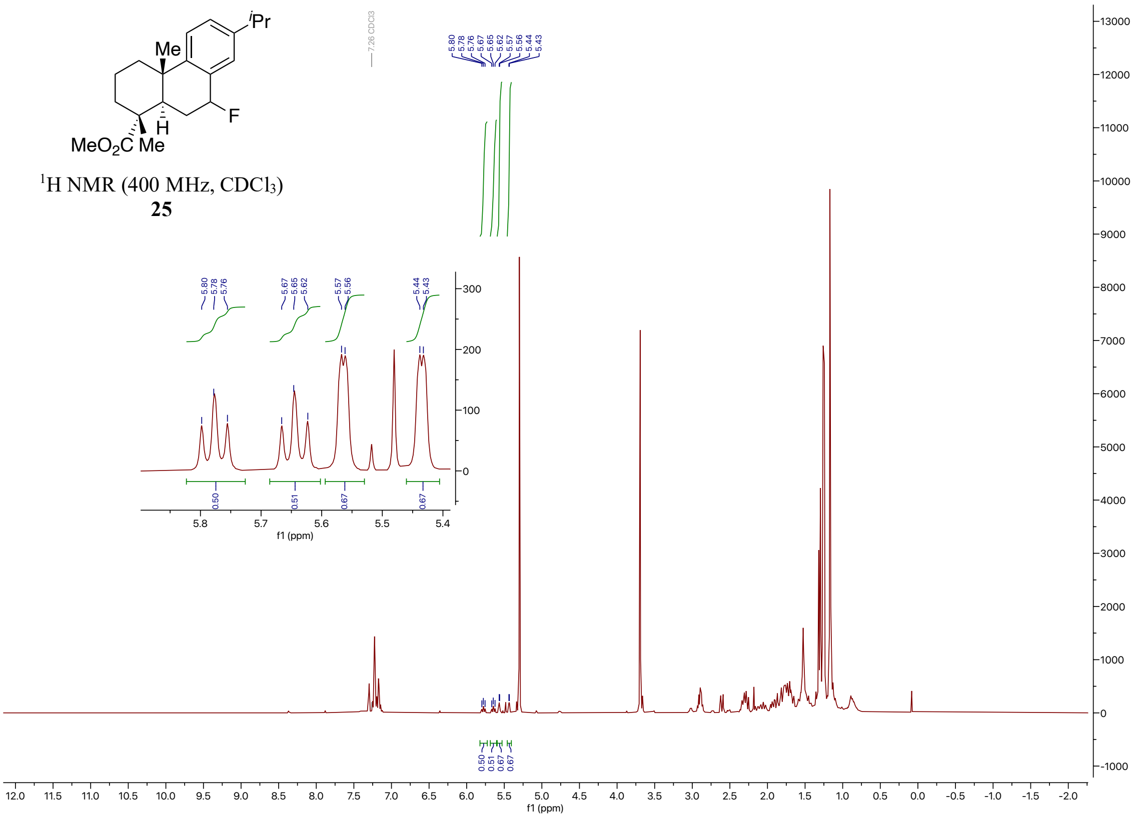

^{1}H NMR (400 MHz, $CDCl_3$)
25

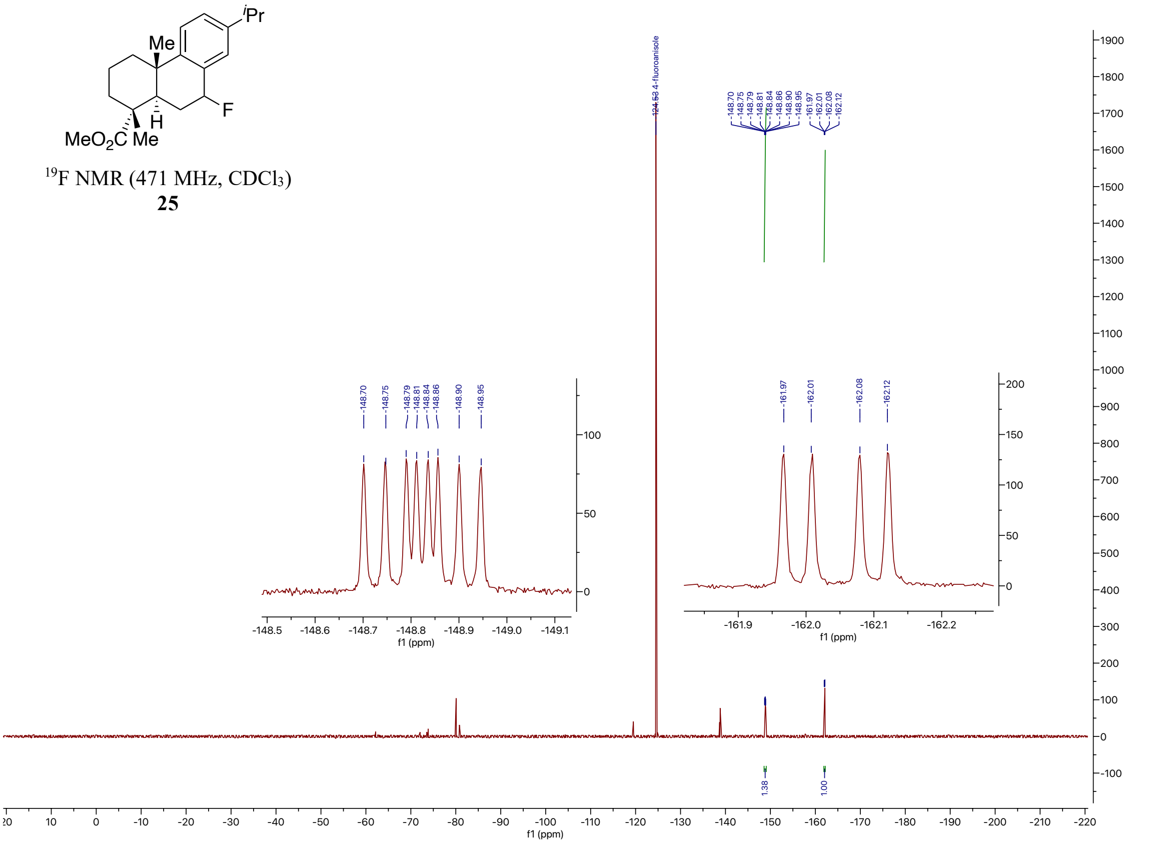

^{19}F NMR (471 MHz, CDCl$_3$)
25

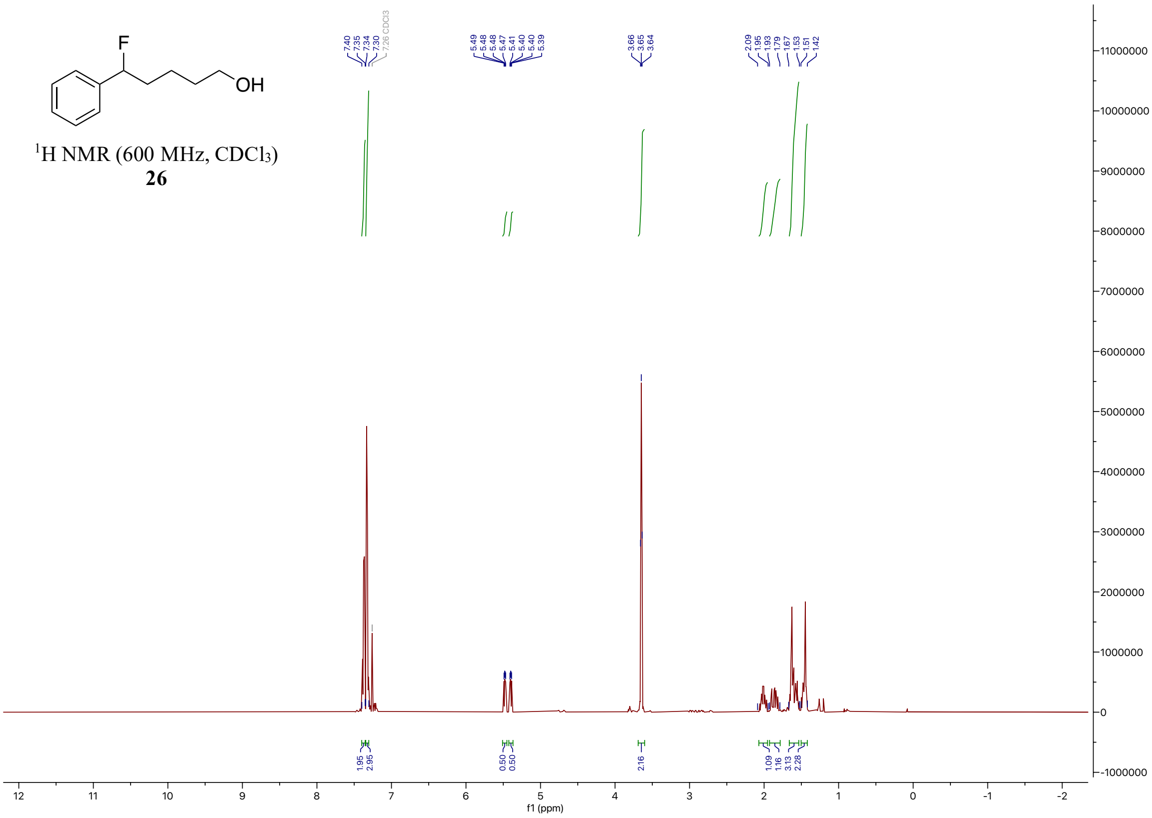

^{1}H NMR (600 MHz, CDCl$_3$)
26

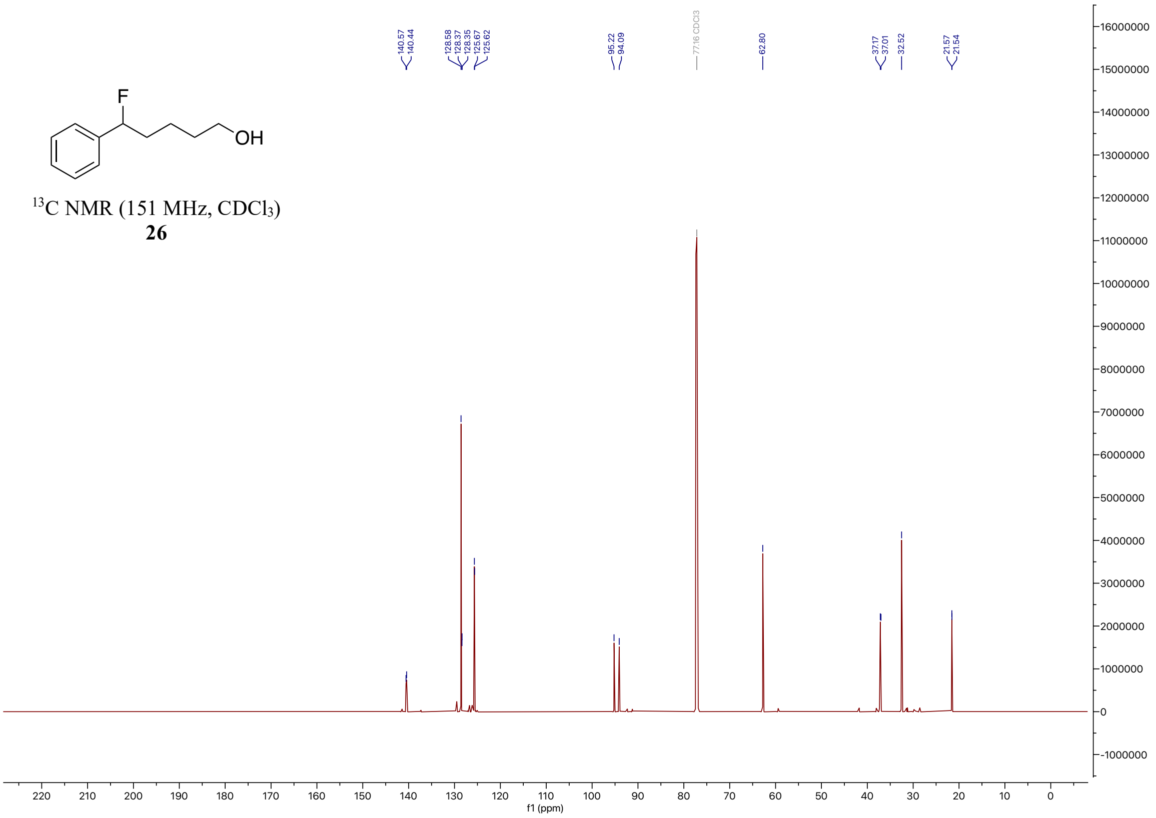

^{13}C NMR (151 MHz, $CDCl_3$)

26

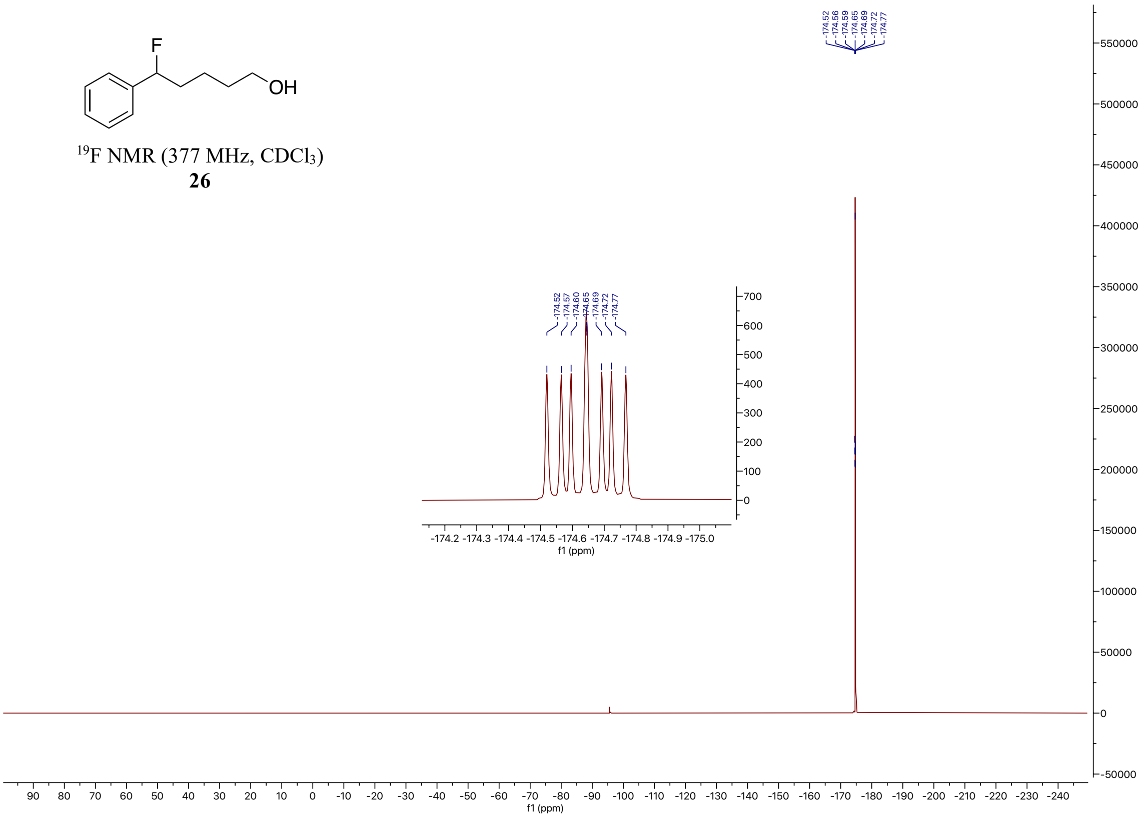

^{19}F NMR (377 MHz, $CDCl_3$)

26

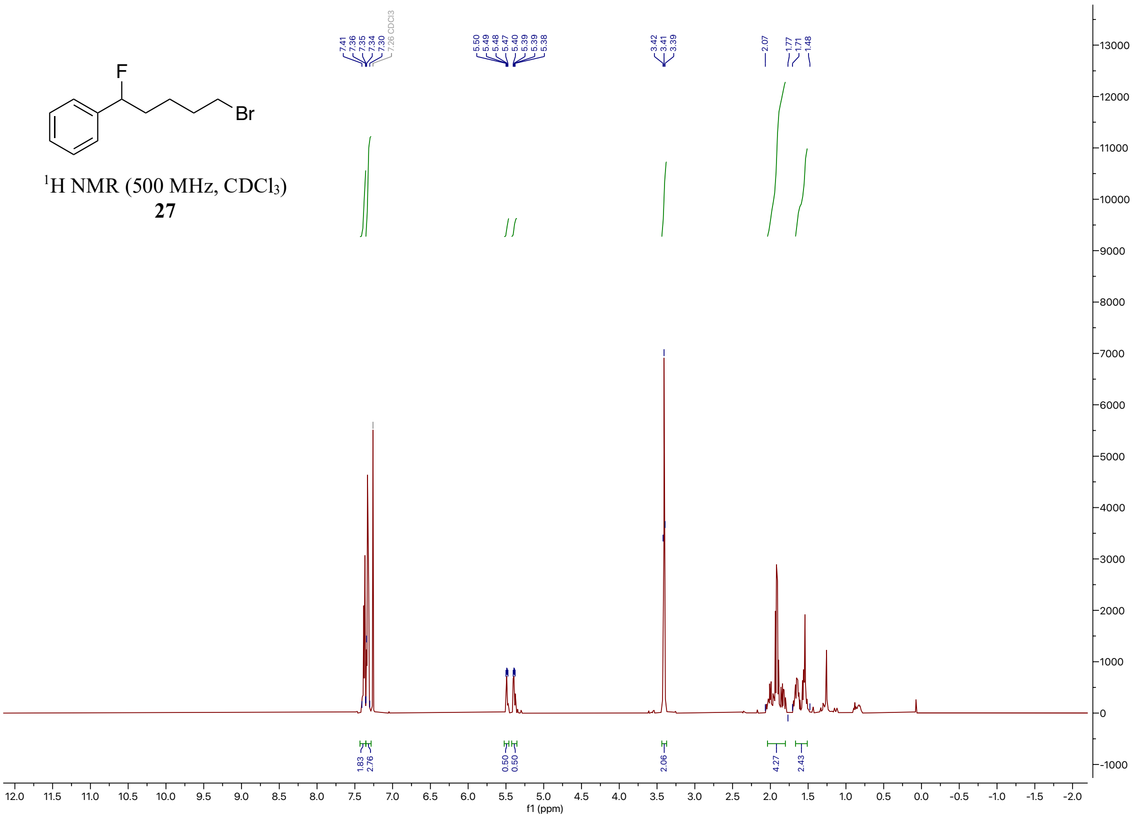

^{1}H NMR (500 MHz, $CDCl_3$)
27

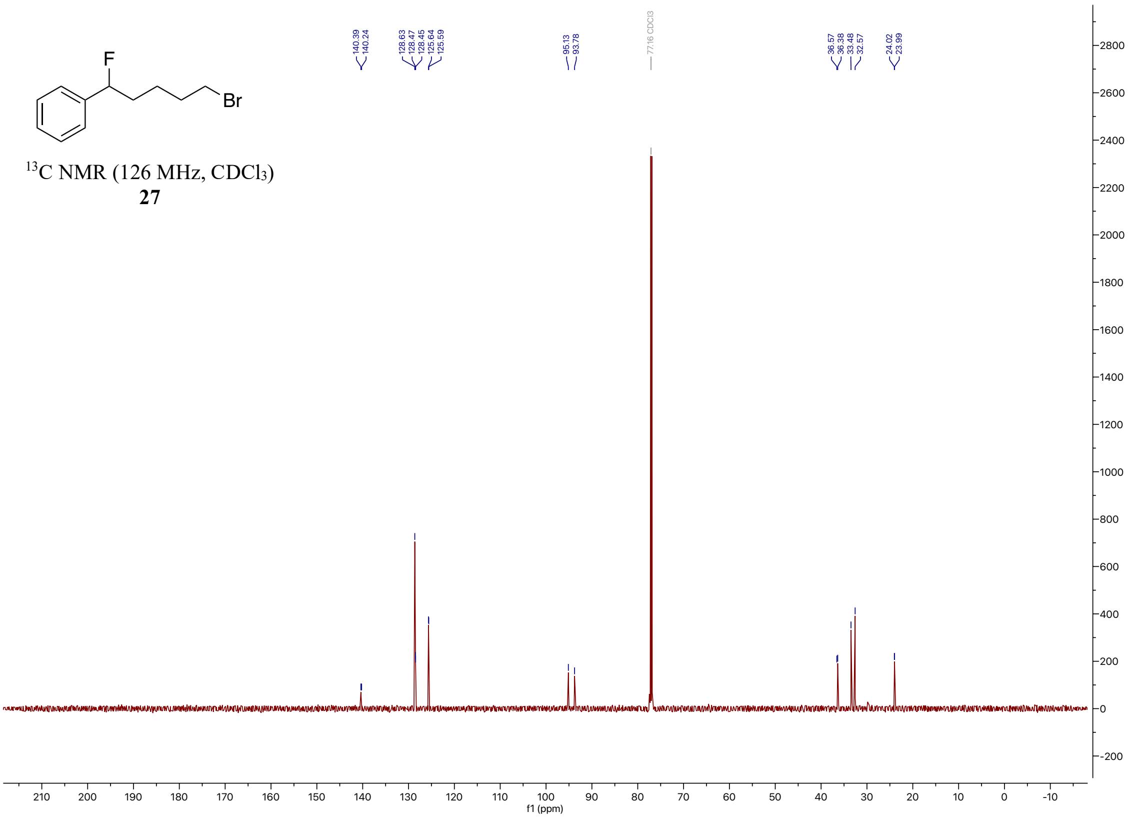

^{13}C NMR (126 MHz, $CDCl_3$)
27

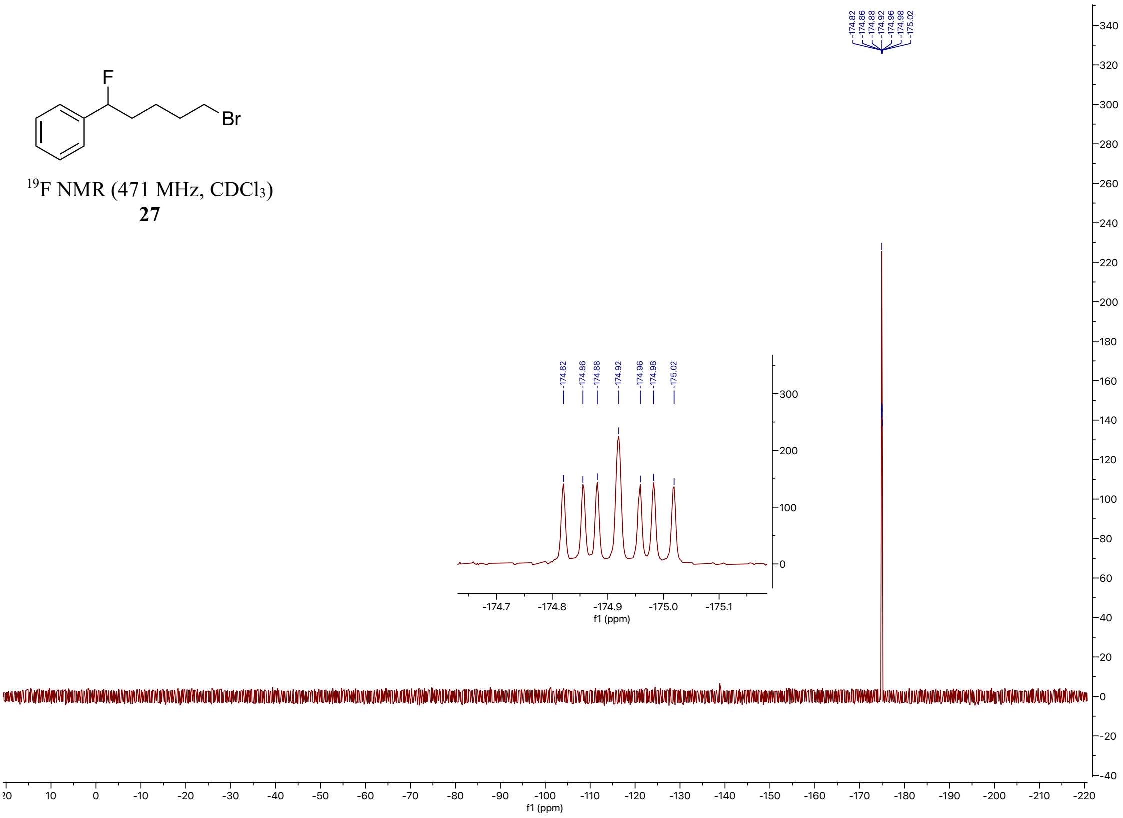

F
Br
^{19}F NMR (471 MHz, $CDCl_3$)
27
f1 (ppm)

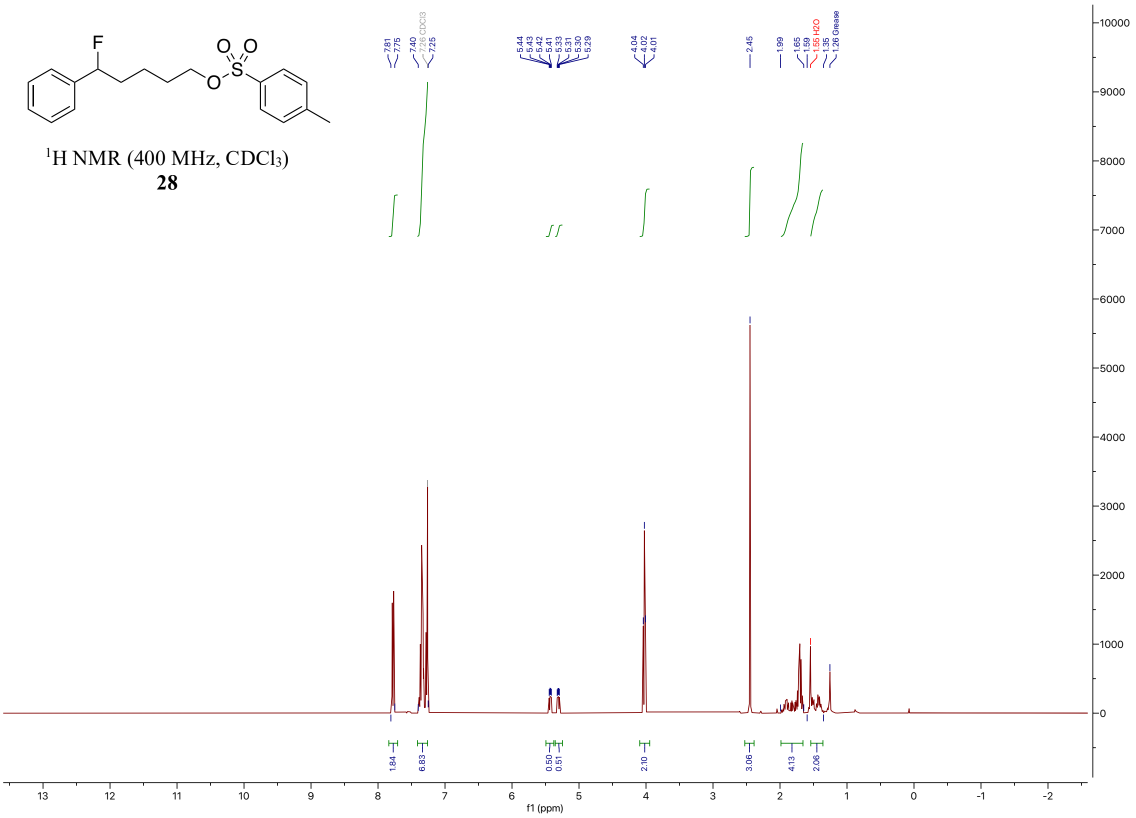

F
O
O
S
O
^{1}H NMR (400 MHz, $CDCl_3$)
28
f1 (ppm)

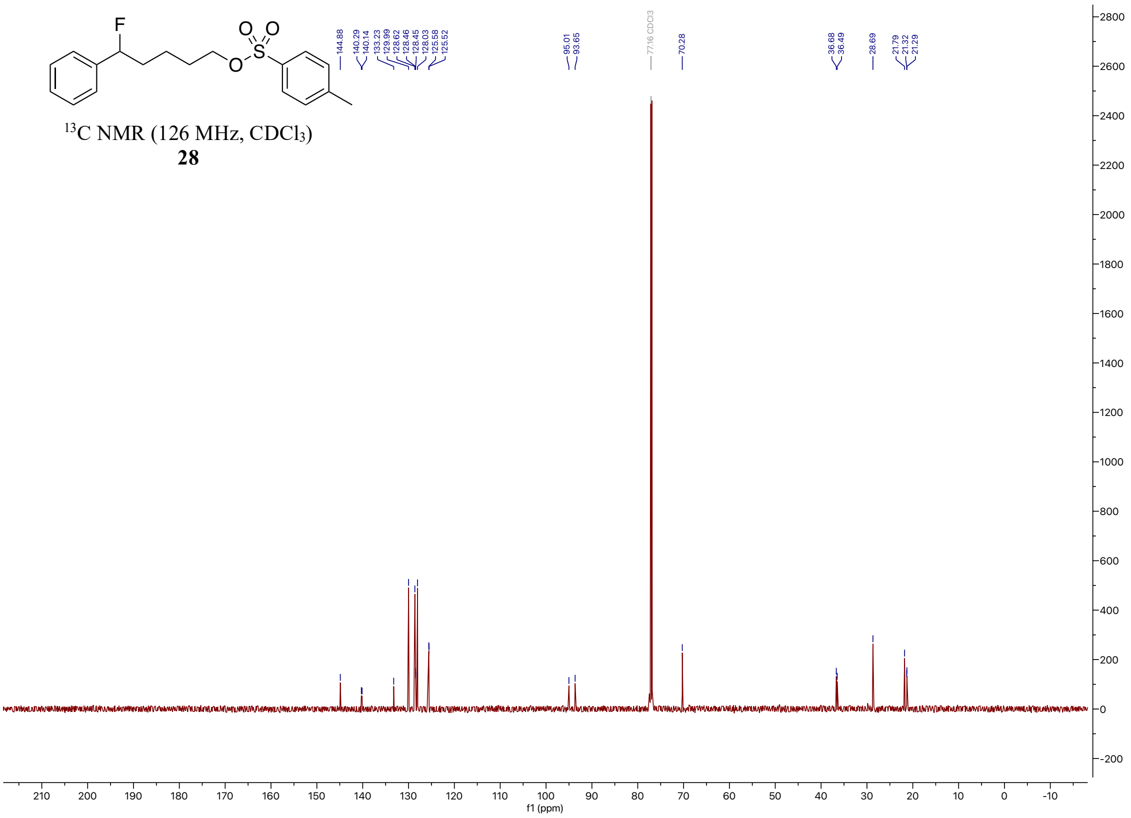

^{13}C NMR (126 MHz, $CDCl_3$)
28

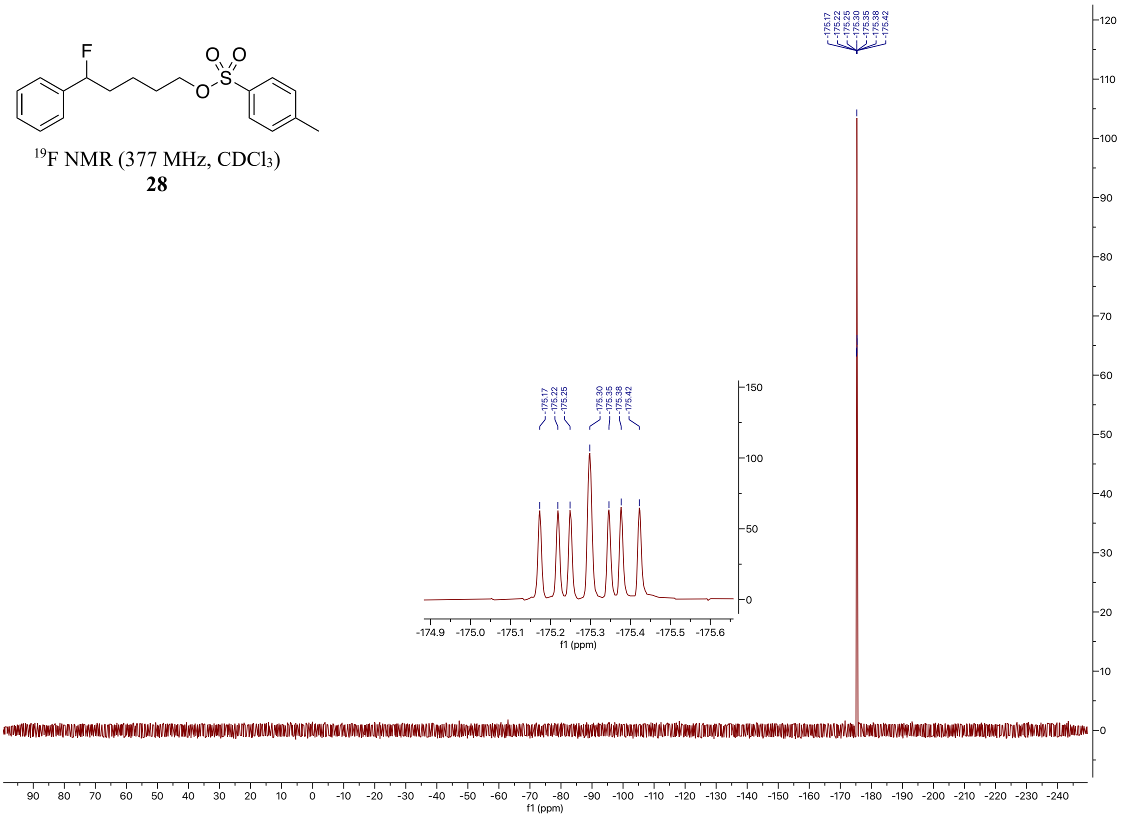

^{19}F NMR (377 MHz, $CDCl_3$)
28

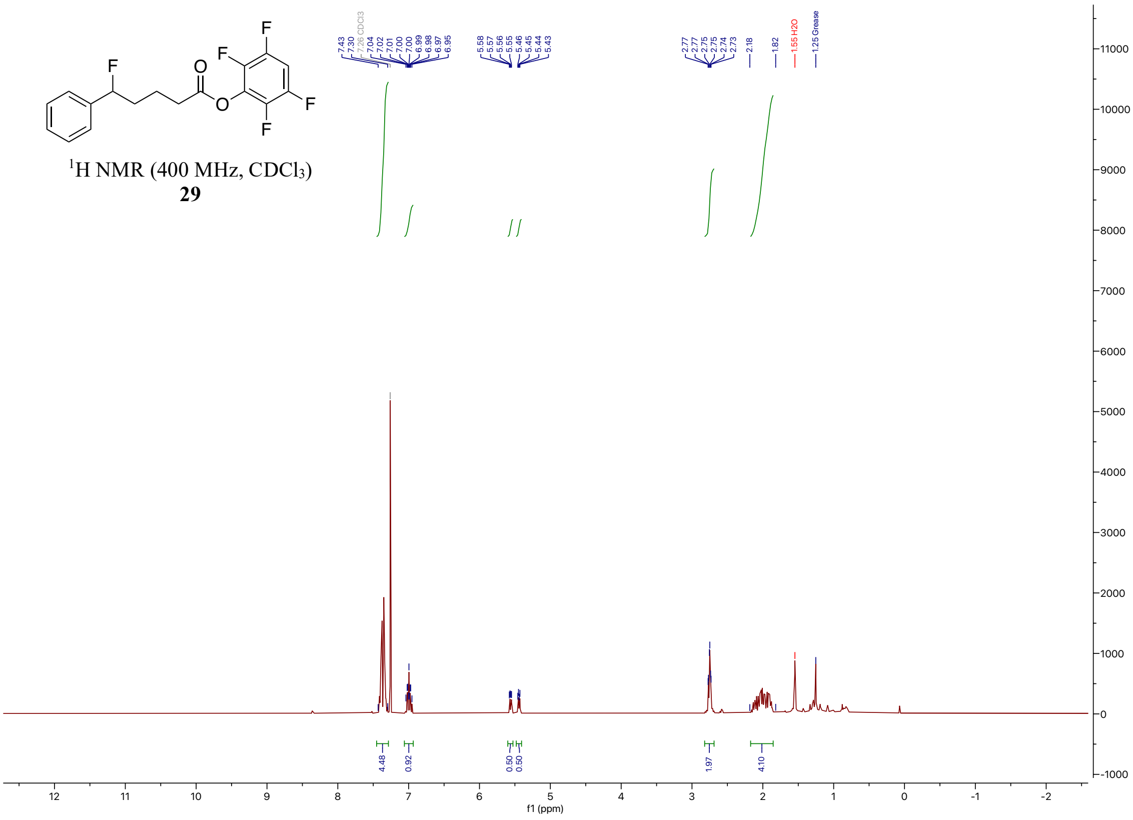

^{1}H NMR (400 MHz, $CDCl_3$)
29

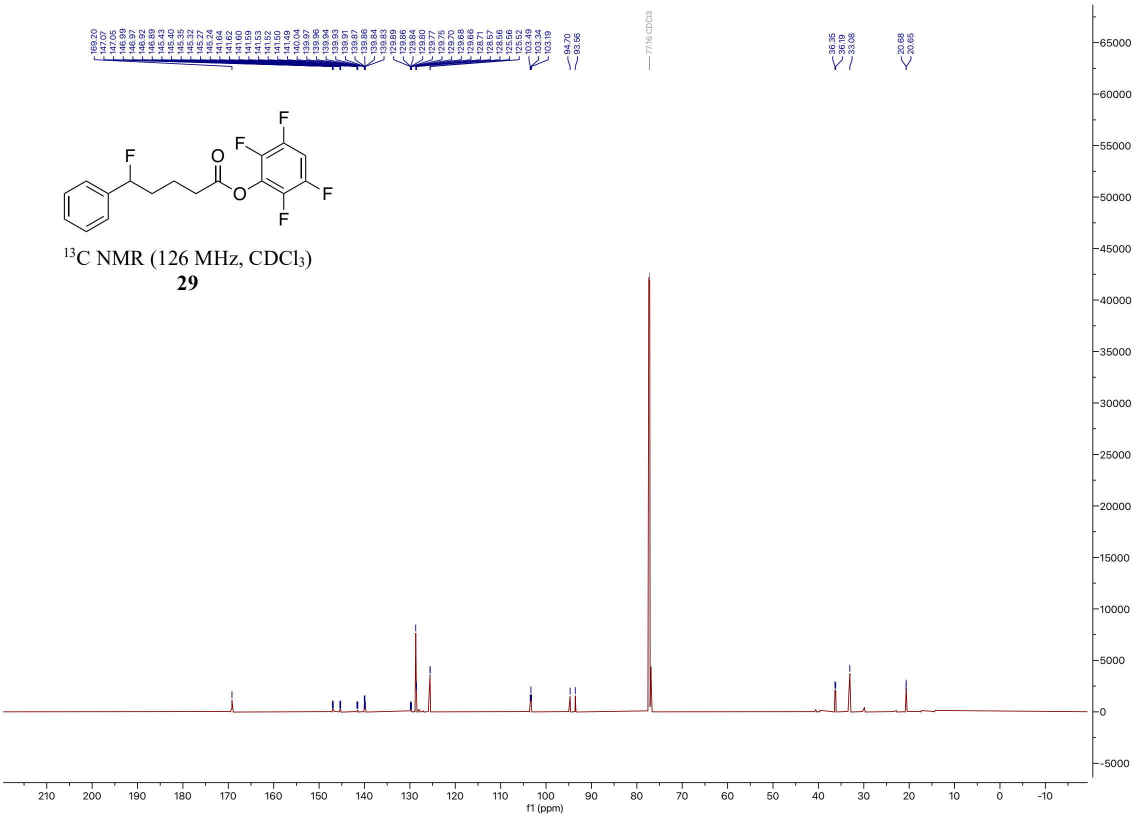

^{13}C NMR (126 MHz, $CDCl_3$)
29

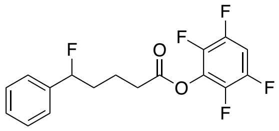

^{19}F NMR (377 MHz, $CDCl_3$)
29

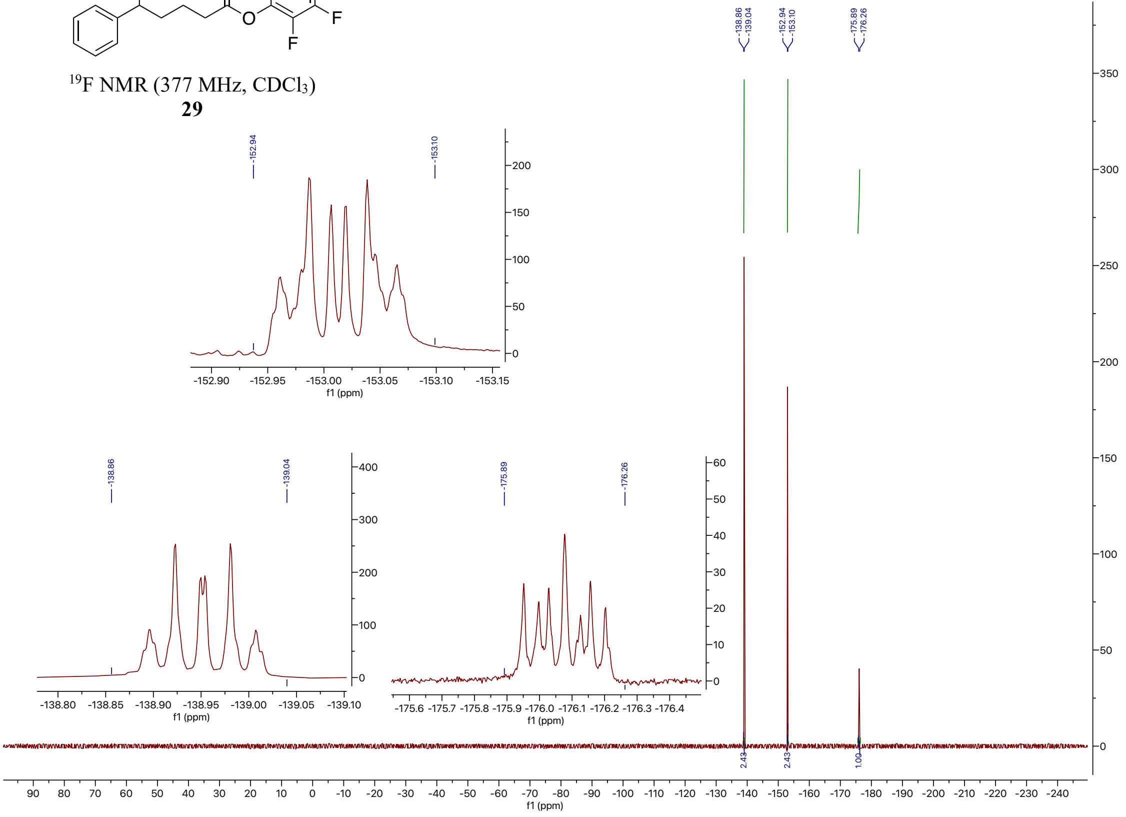

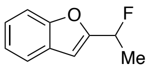

^{1}H NMR (400 MHz, $CDCl_3$)
30

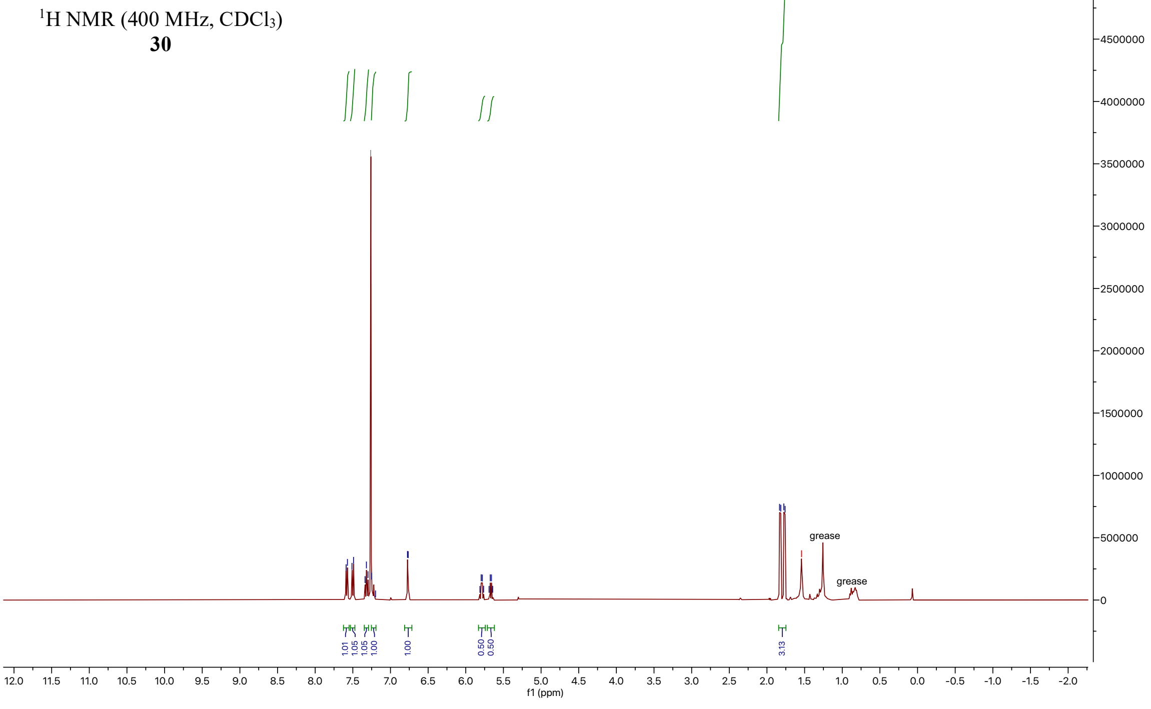

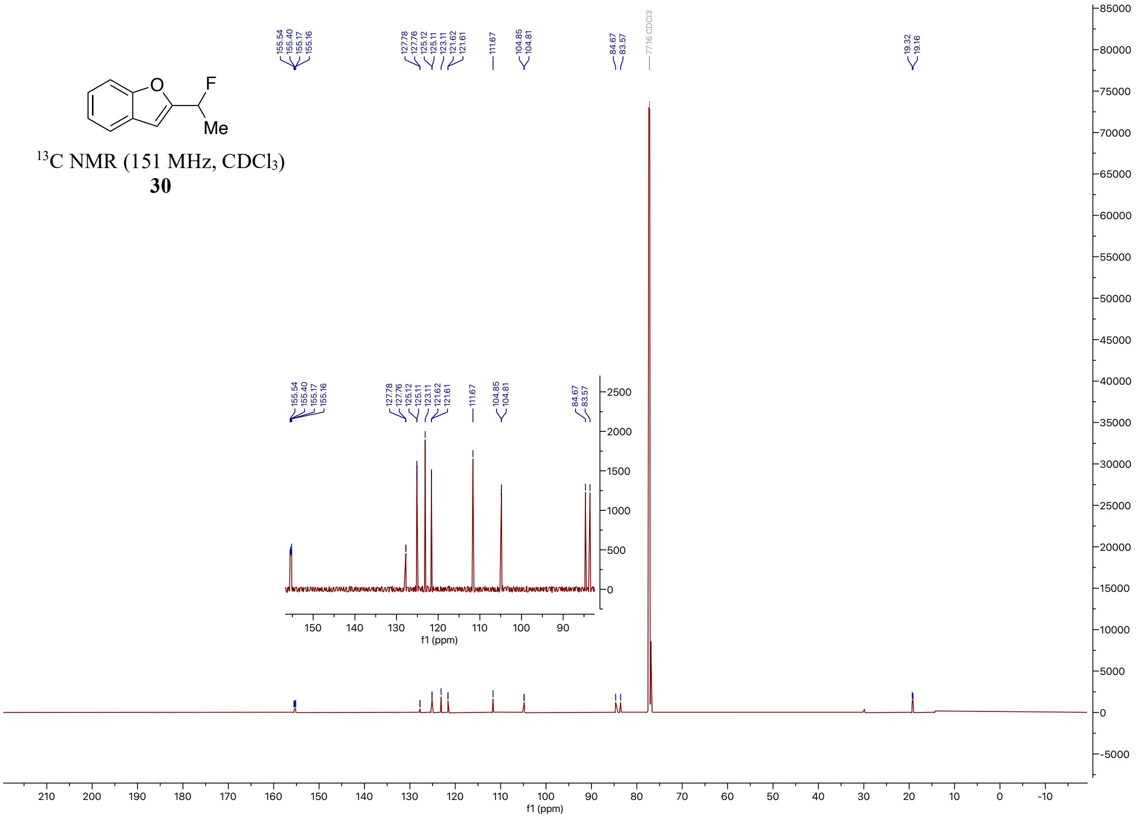

^{13}C NMR (151 MHz, $CDCl_3$)
30

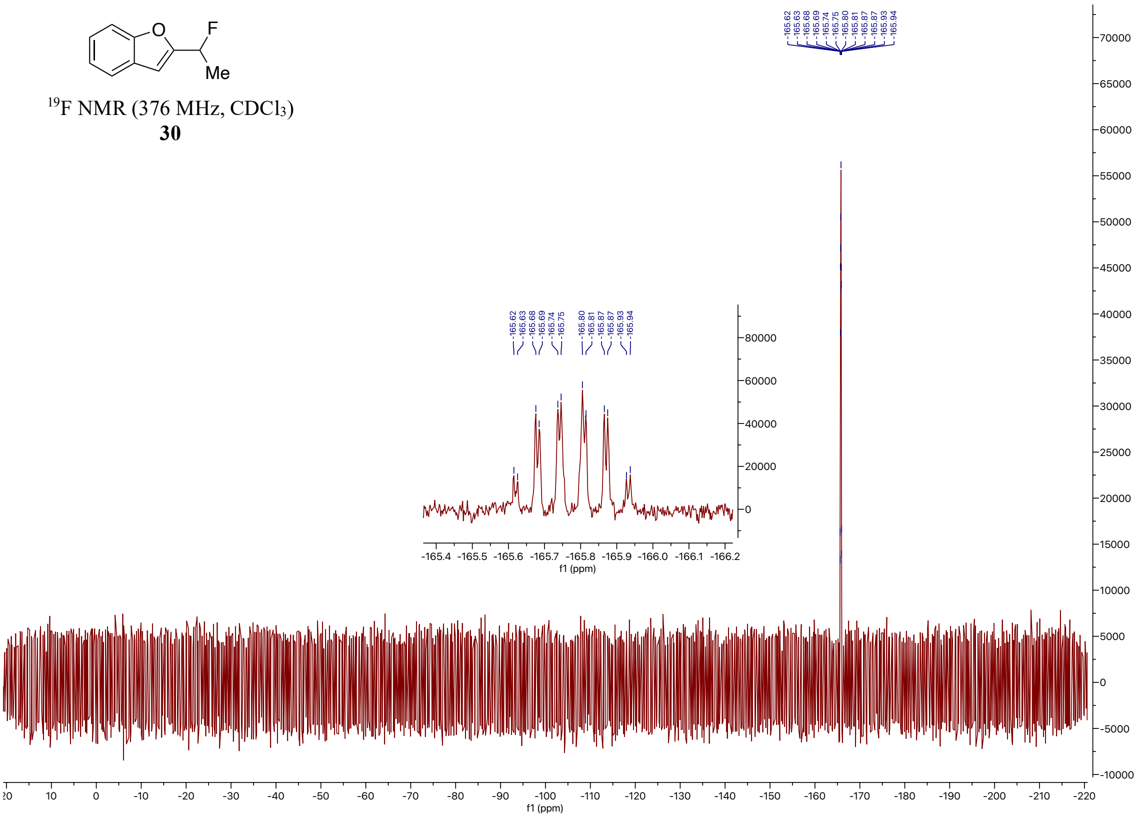

^{19}F NMR (376 MHz, $CDCl_3$)
30

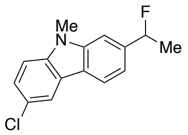

^{1}H NMR (400 MHz, $CDCl_3$)
31

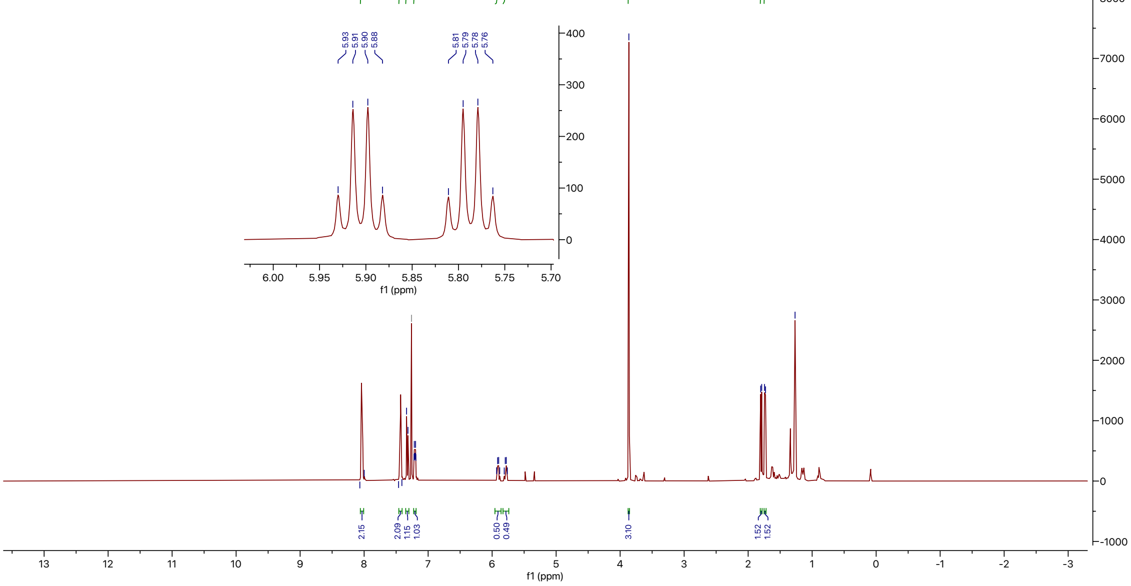

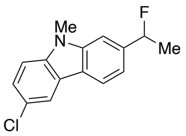

^{13}C NMR (101 MHz, $CDCl_3$)
31

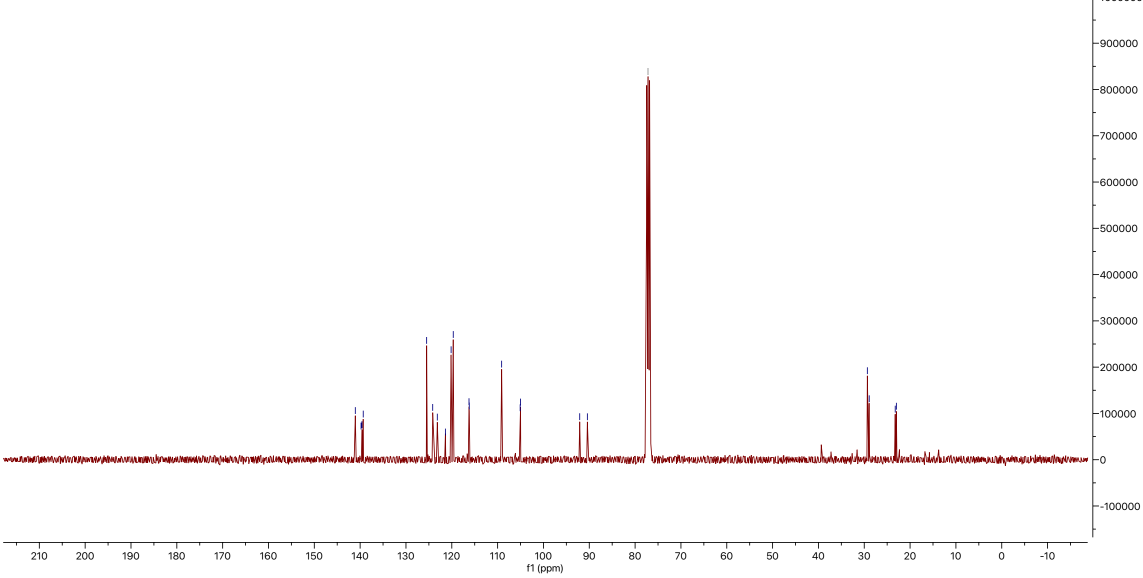

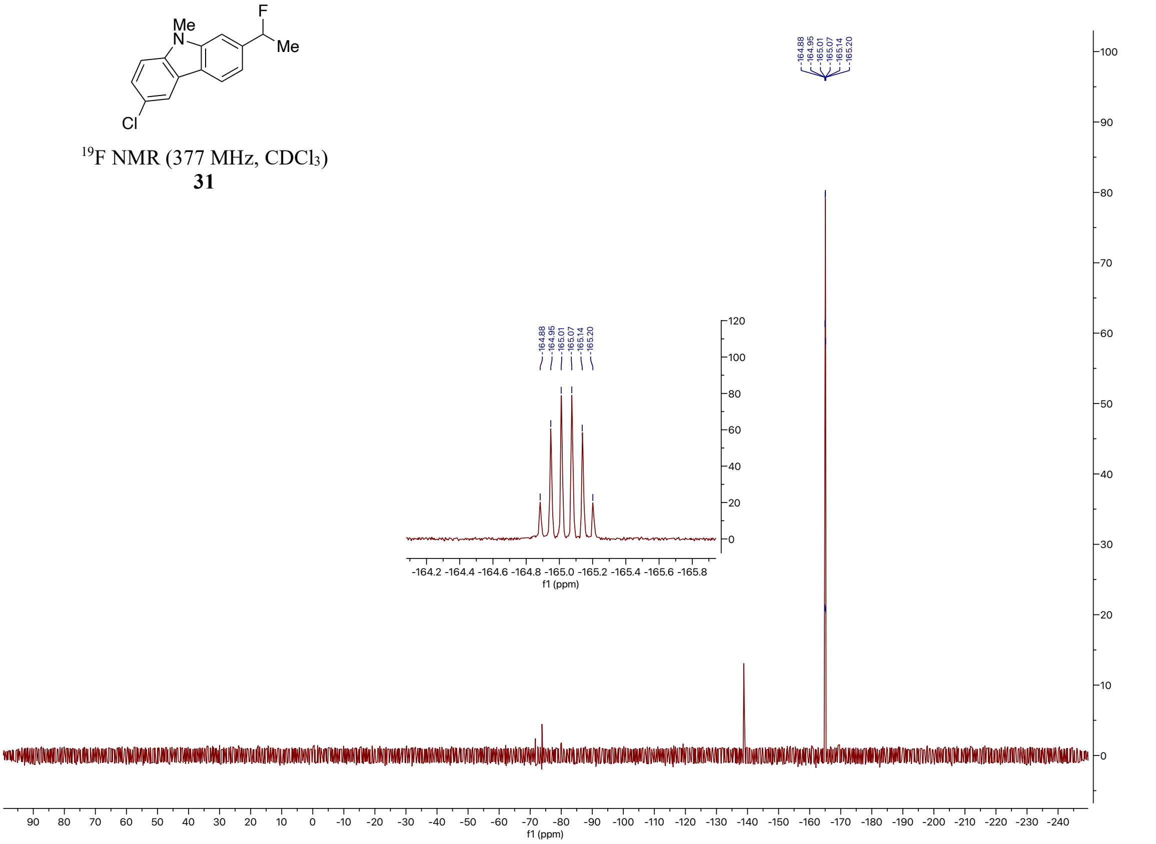

^{19}F NMR (377 MHz, $CDCl_3$)
31

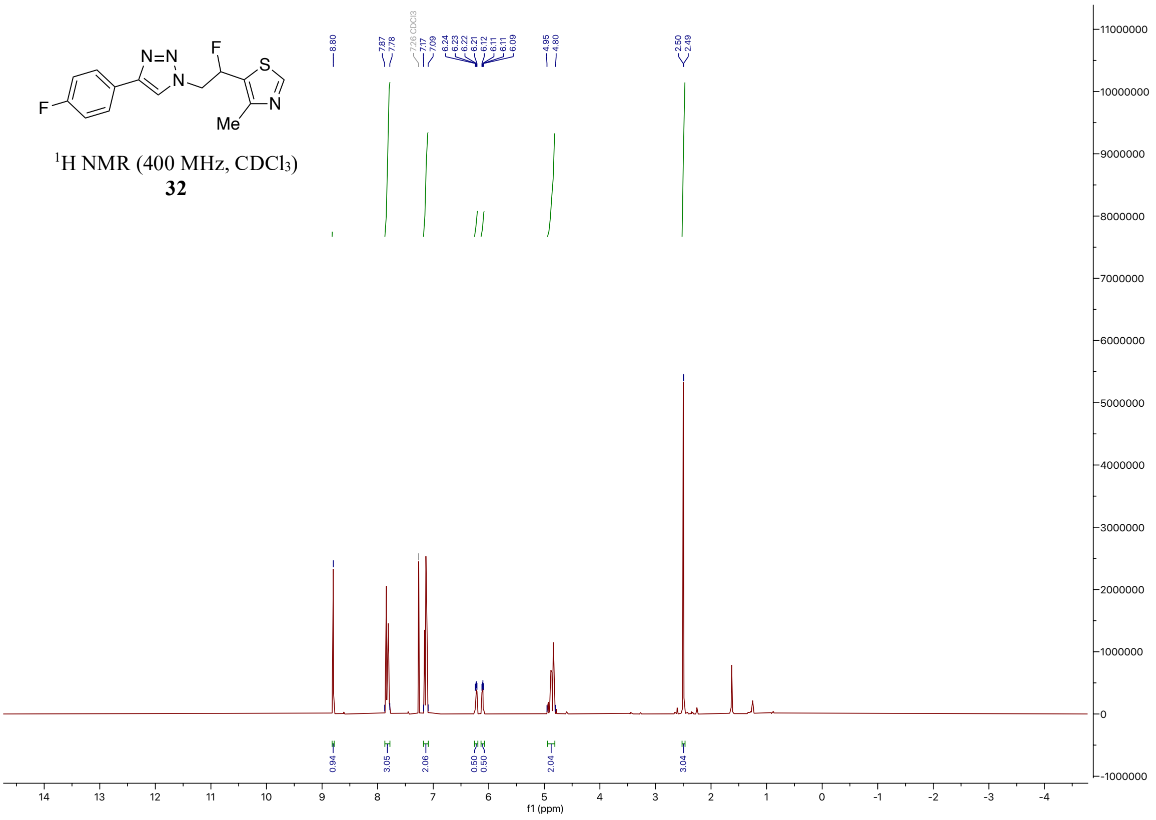

^{1}H NMR (400 MHz, $CDCl_3$)
32

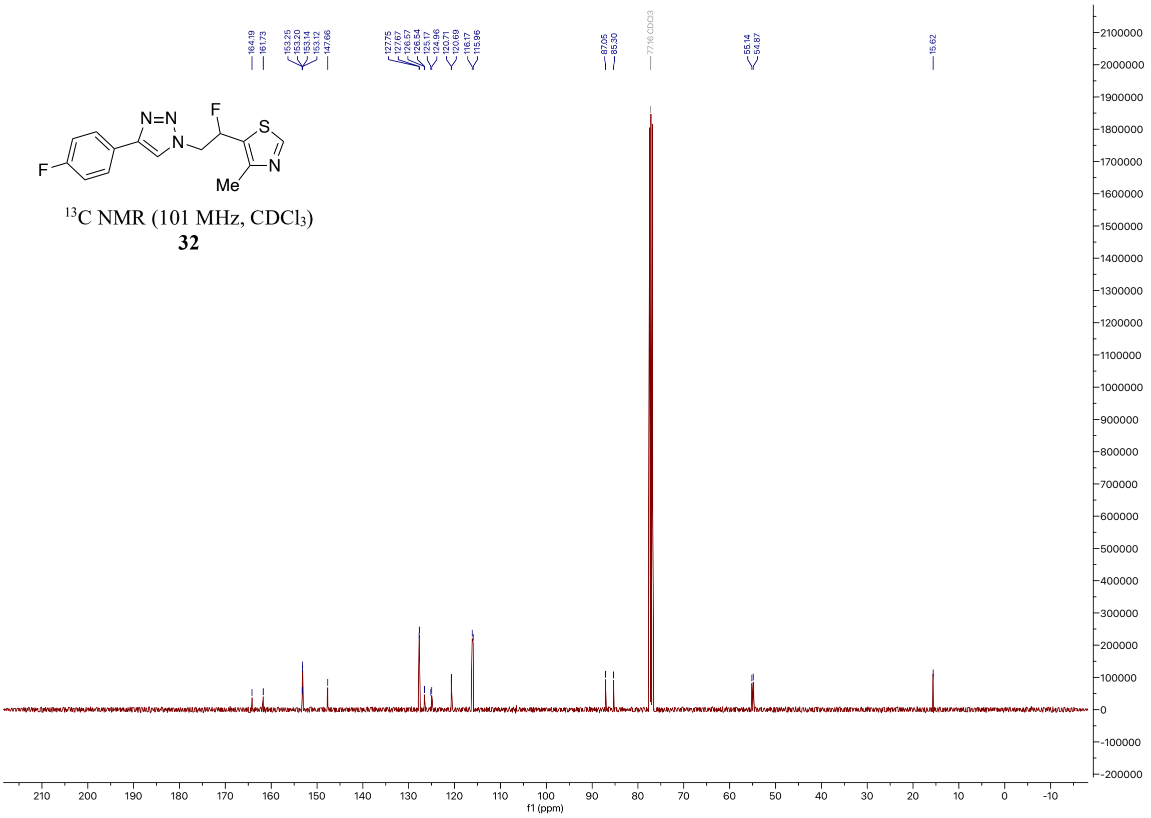
164.19
161.73
153.25
153.20
153.14
153.12
147.66
127.75
127.67
126.57
126.54
125.17
124.96
120.71
120.69
116.17
115.96
87.05
85.30
77.16 CDCl3
55.14
54.87
15.62
^{13}C NMR (101 MHz, CDCl$_3$)
32
f1 (ppm)

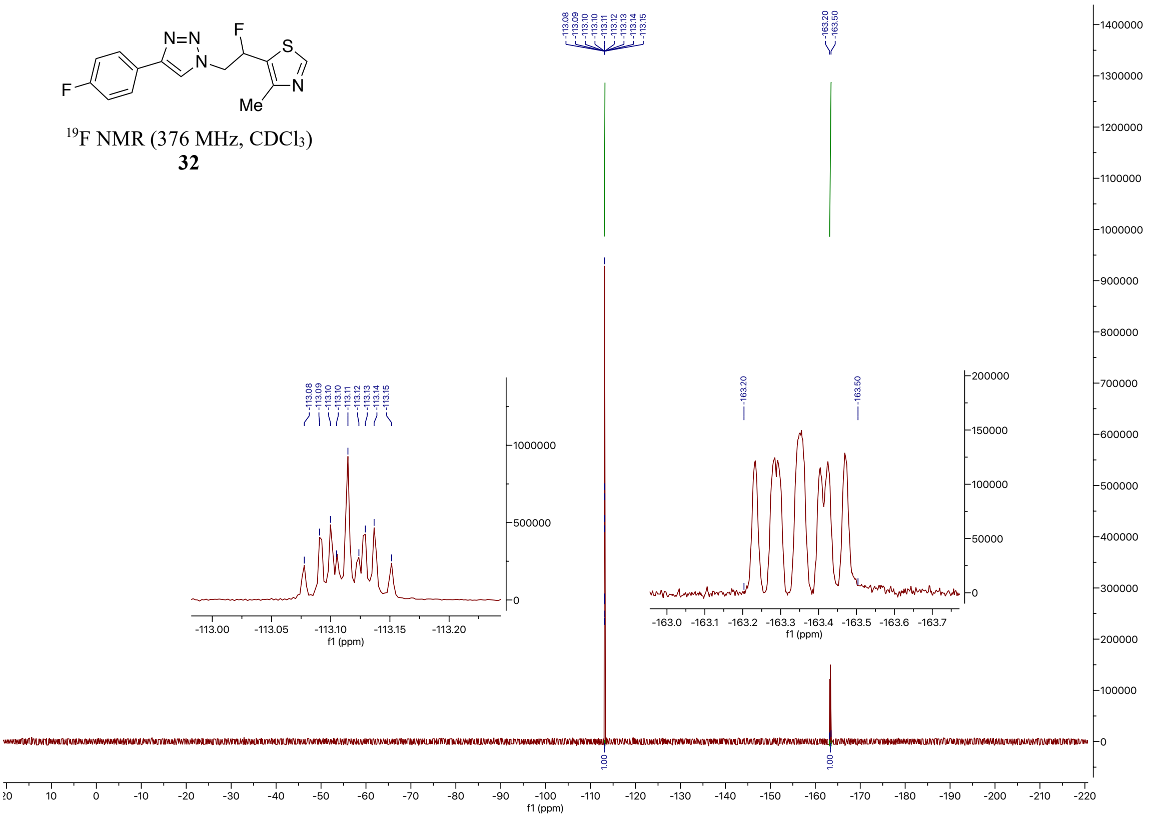
-113.08
-113.09
-113.10
-113.10
-113.11
-113.12
-113.13
-113.14
-113.15
-163.20
-163.50
^{19}F NMR (376 MHz, CDCl$_3$)
32
1.00
1.00
f1 (ppm)

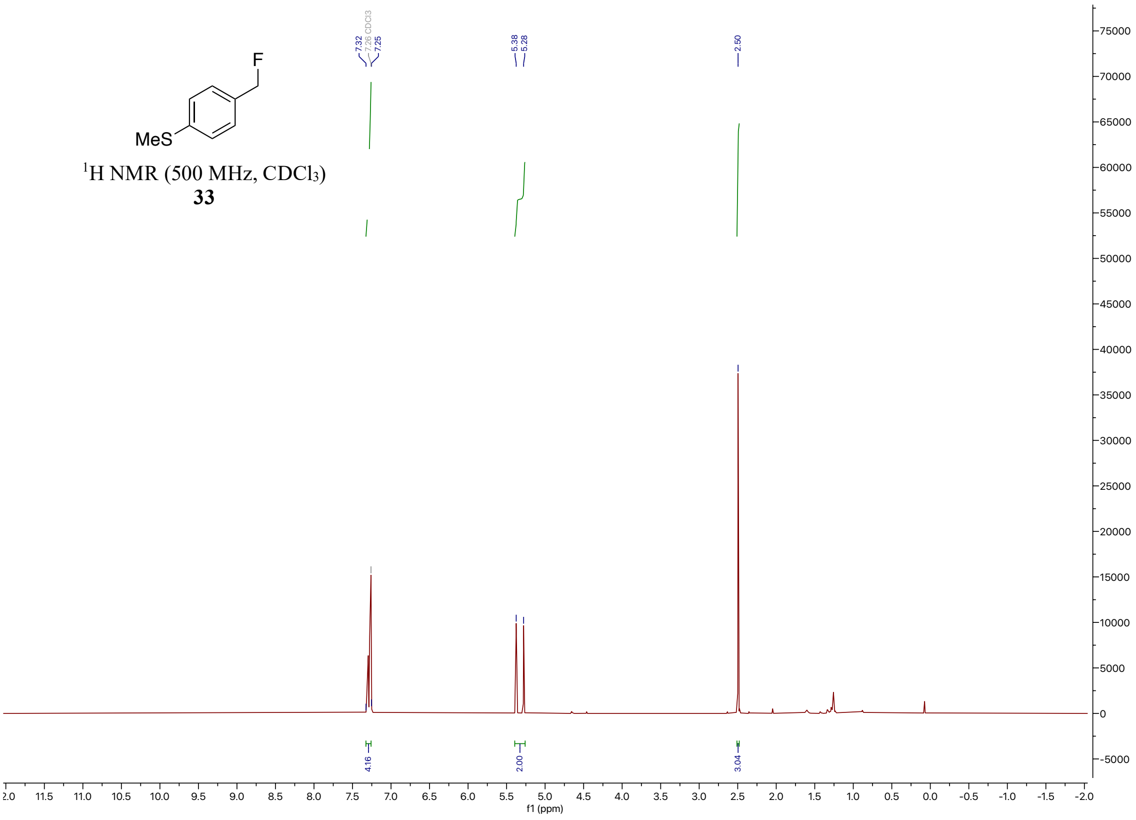

^{1}H NMR (500 MHz, $CDCl_3$)
33

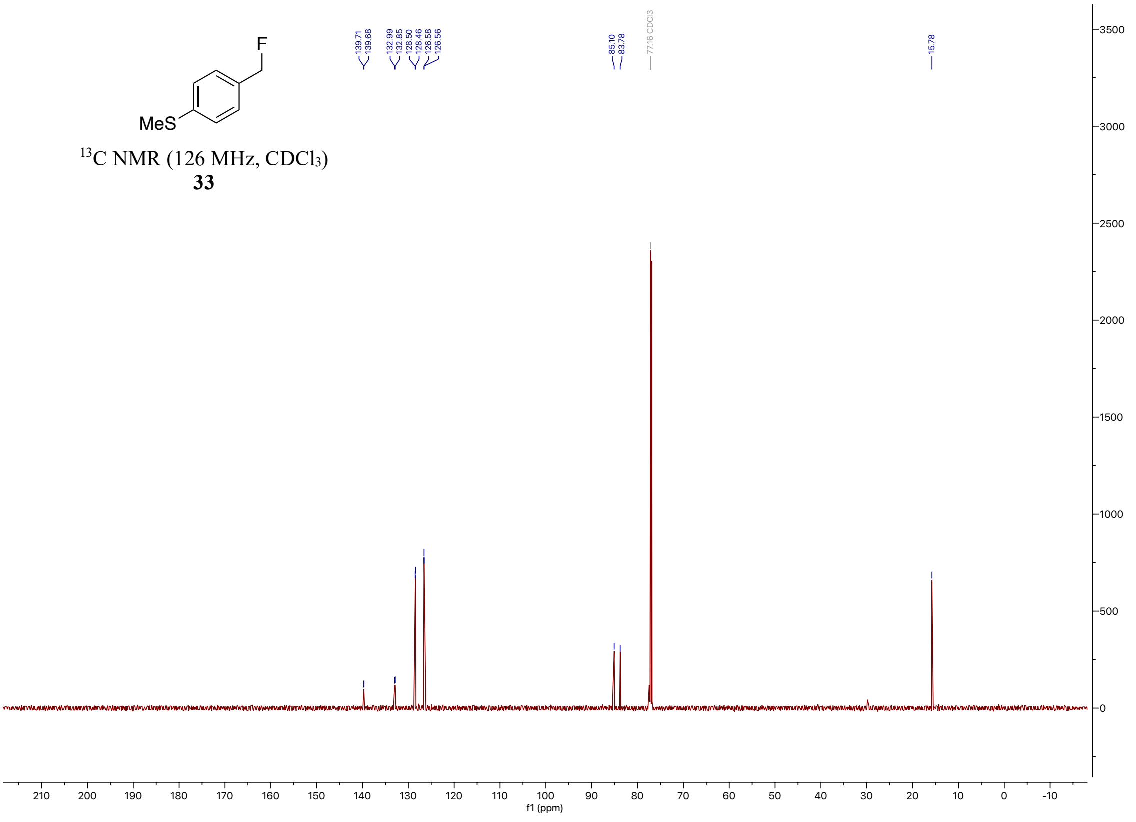

^{13}C NMR (126 MHz, $CDCl_3$)
33

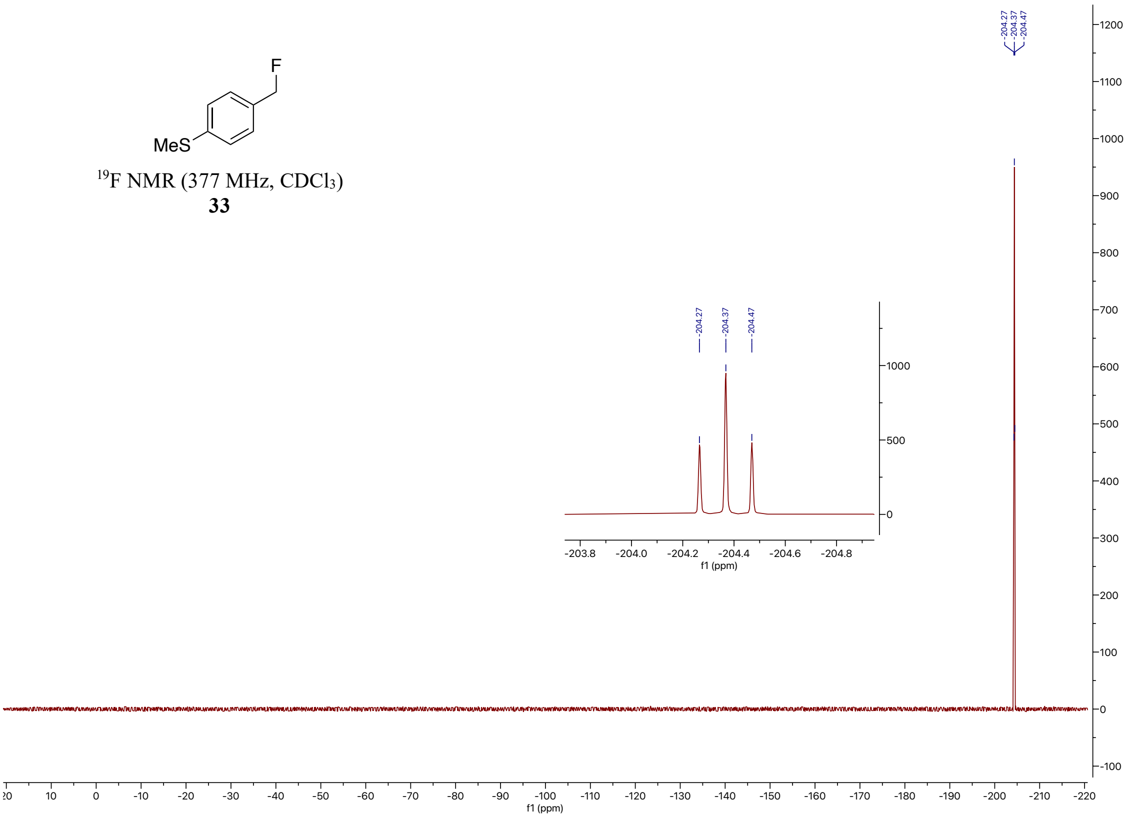

^{19}F NMR (377 MHz, $CDCl_3$)
33

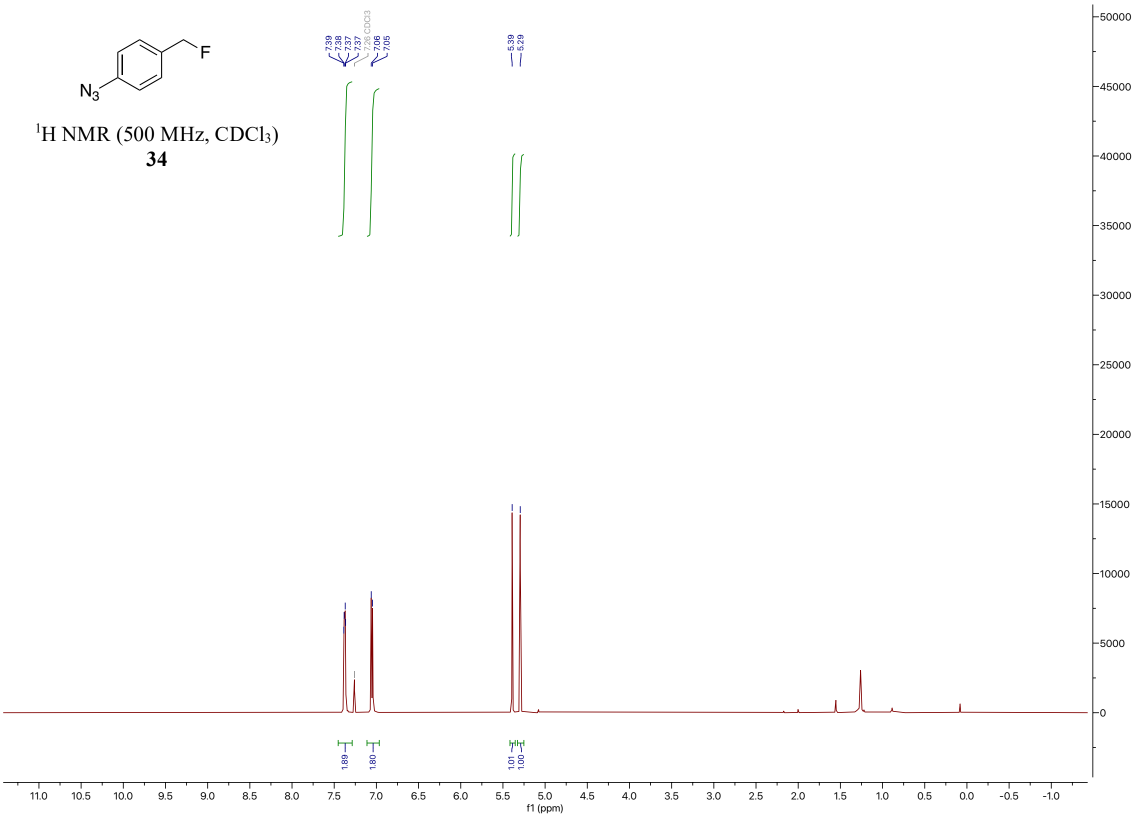

^{1}H NMR (500 MHz, $CDCl_3$)
34

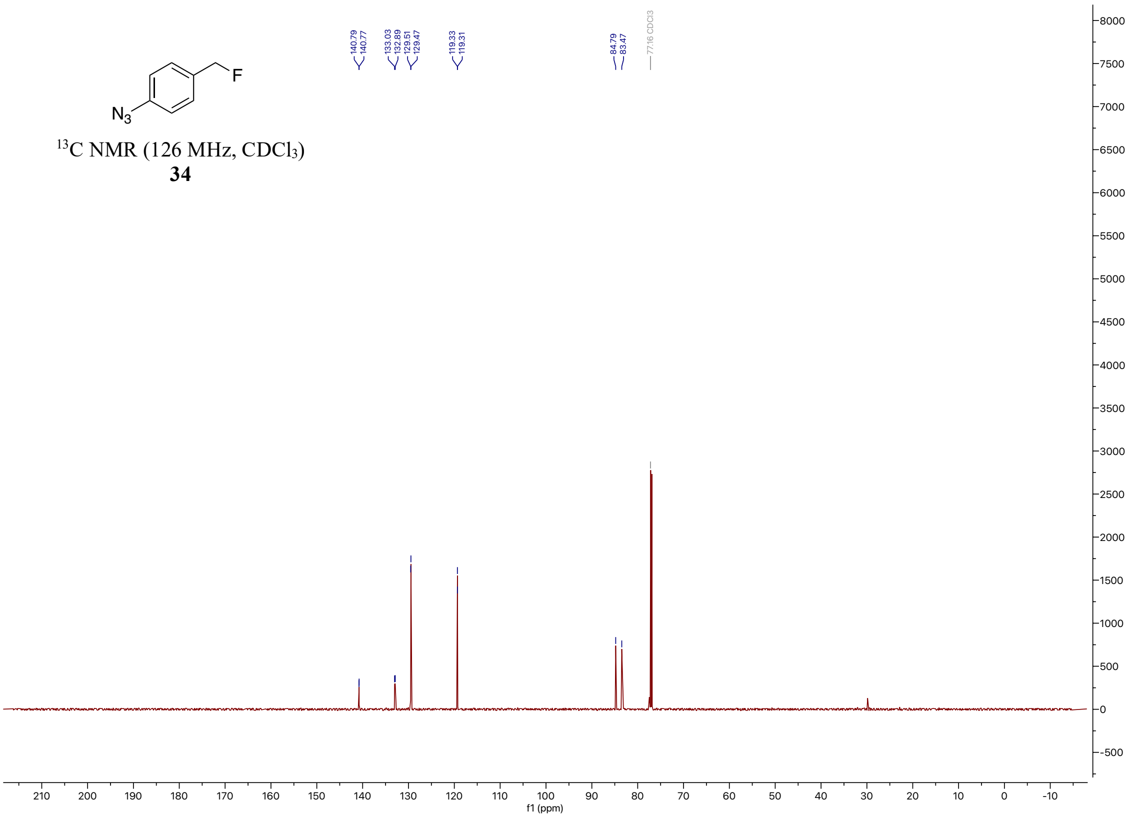

F
N3
13C NMR (126 MHz, CDCl3)
34
140.79
140.77
133.03
132.89
129.51
129.47
119.33
119.31
84.79
83.47
77.16 CDCl3
f1 (ppm)

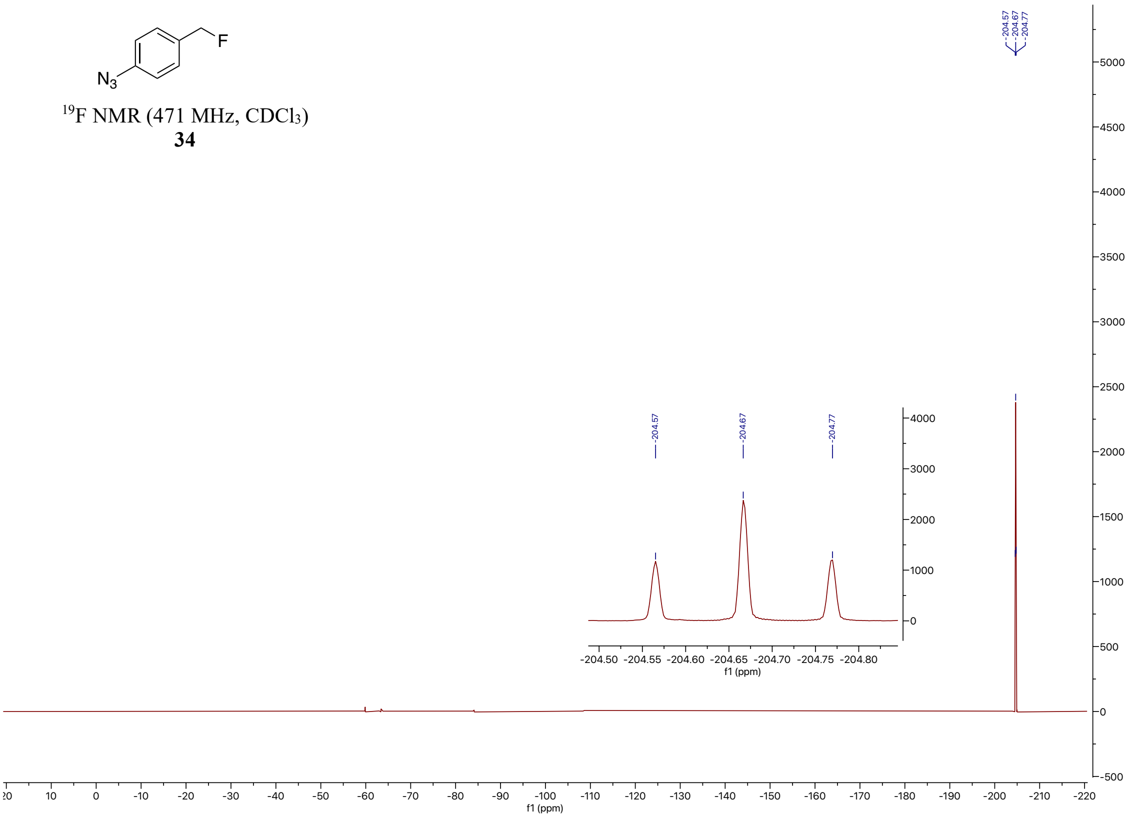

F
N3
19F NMR (471 MHz, CDCl3)
34
-204.57
-204.67
-204.77
f1 (ppm)

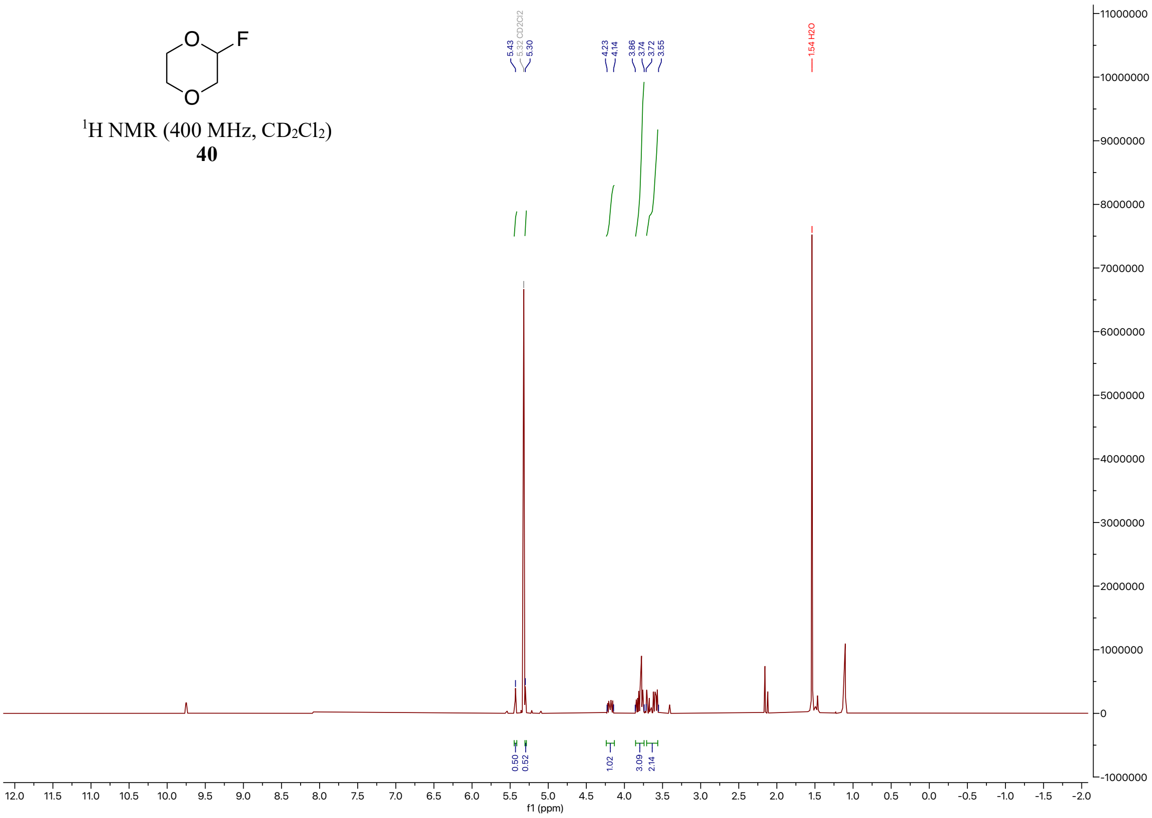
O
F
O
1H NMR (400 MHz, CD2Cl2)
40
5.43
5.32 CD2Cl2
5.30
4.23
4.14
3.86
3.74
3.72
3.55
1.54 H2O
0.50
0.52
1.02
3.09
2.14
f1 (ppm)

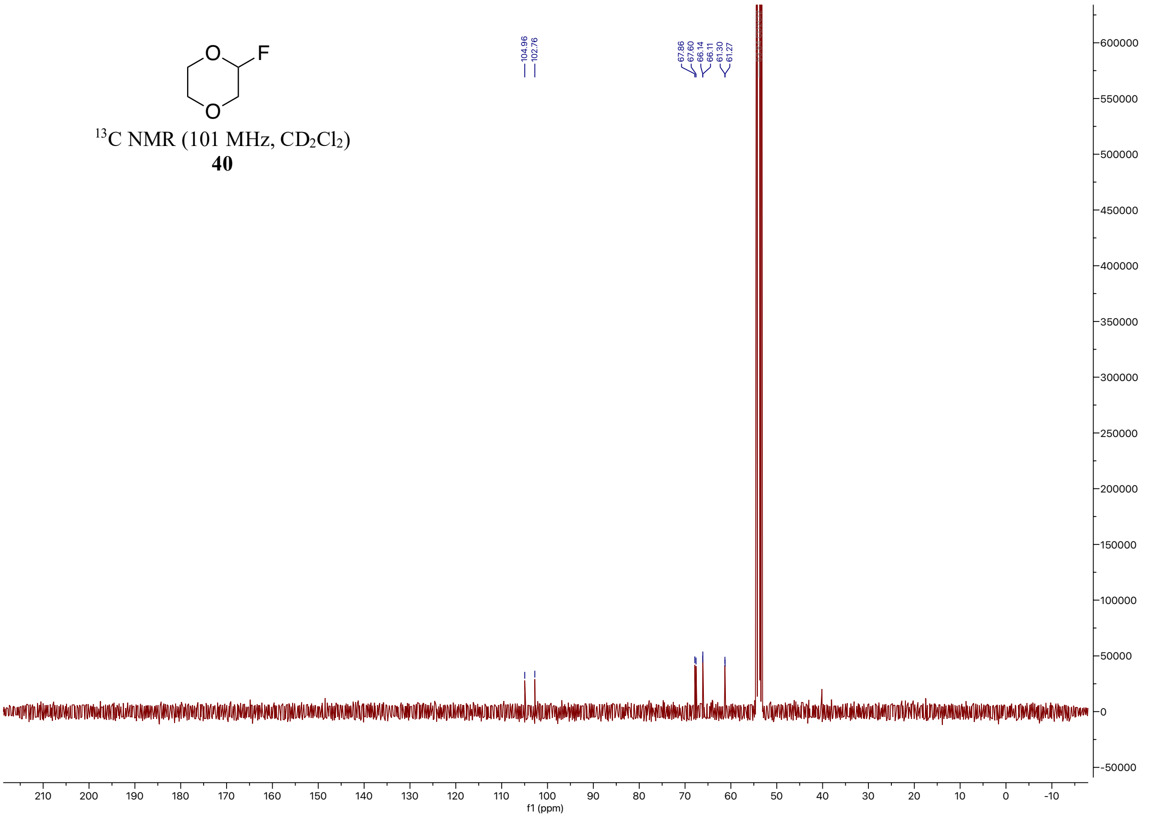
O
F
O
13C NMR (101 MHz, CD2Cl2)
40
104.96
102.76
67.86
67.60
66.14
66.11
61.30
61.27
f1 (ppm)

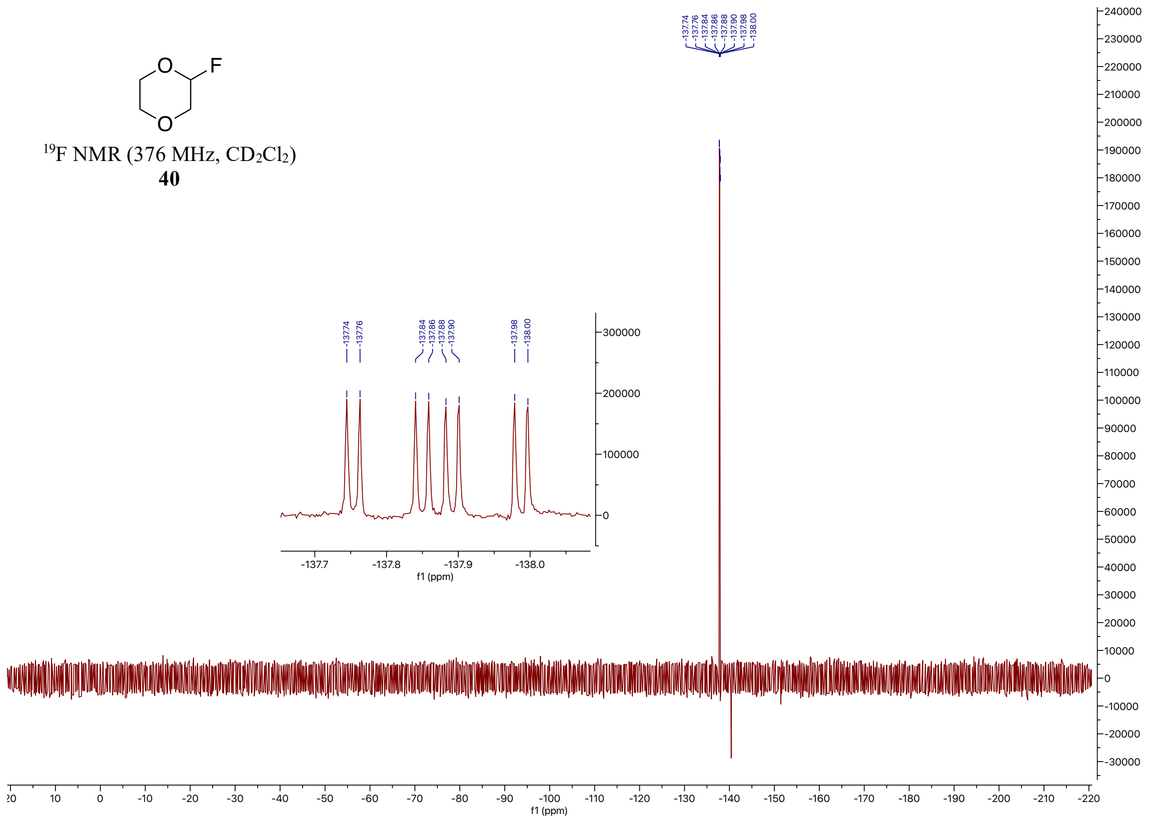

^{19}F NMR (376 MHz, CD_2Cl_2)
40

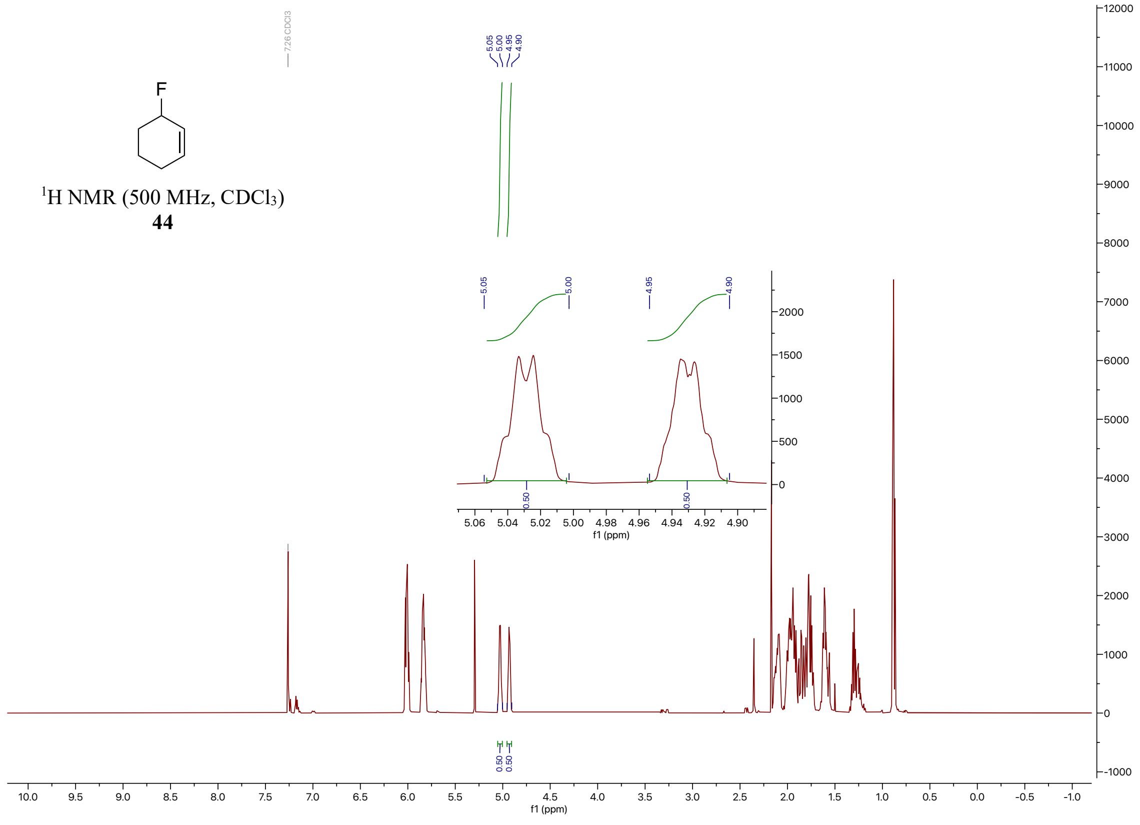

^{1}H NMR (500 MHz, $CDCl_3$)
44

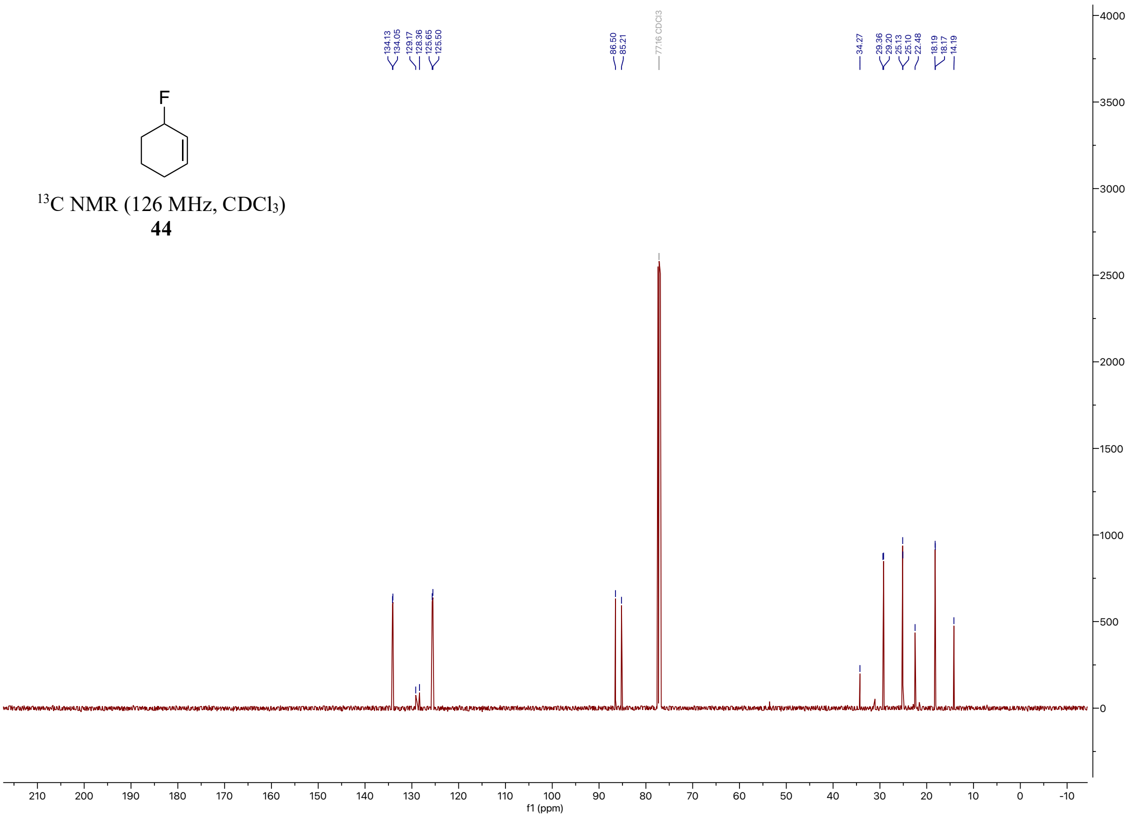

^{13}C NMR (126 MHz, $CDCl_3$)
44

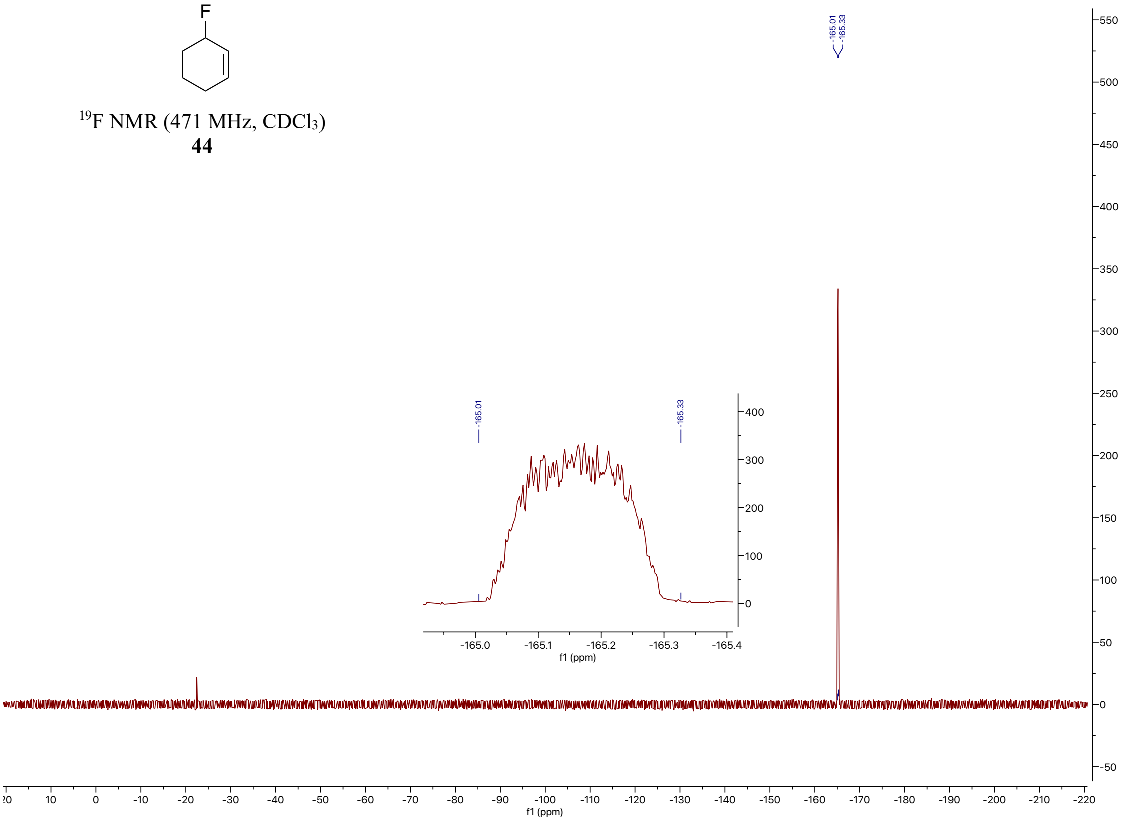

^{19}F NMR (471 MHz, $CDCl_3$)
44

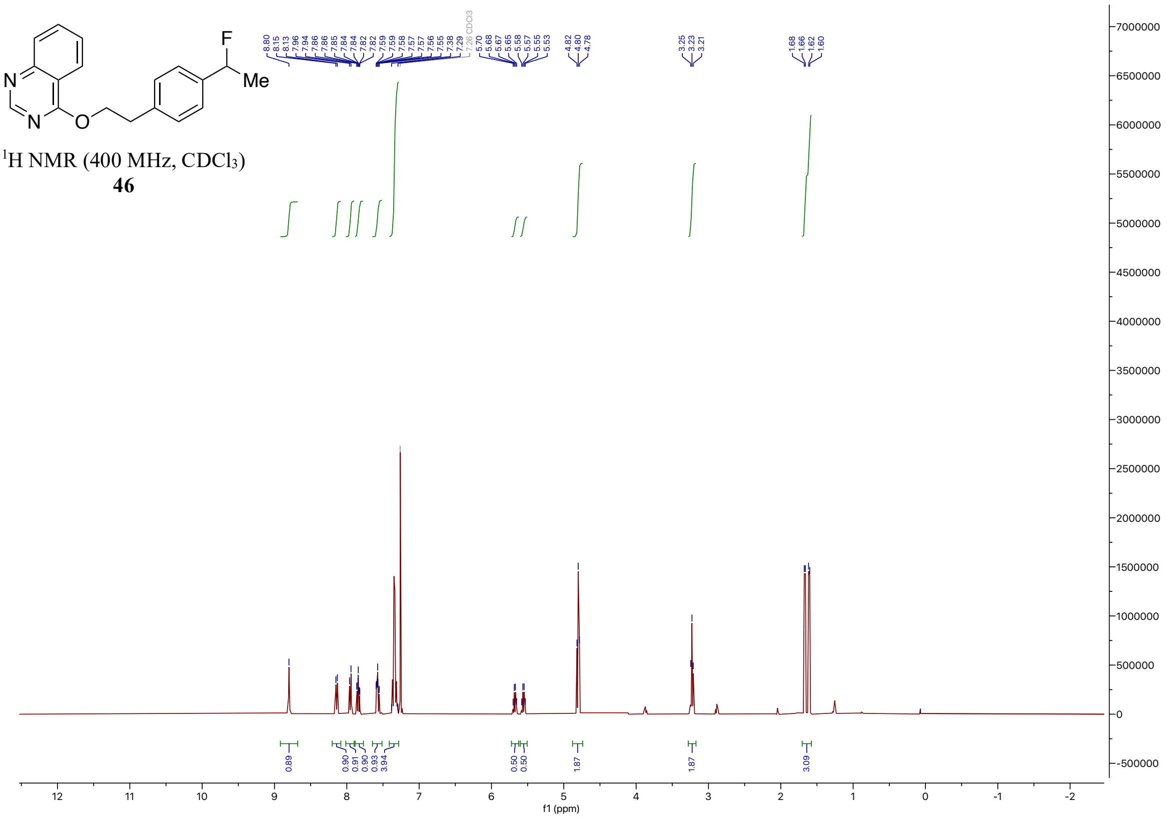

^{1}H NMR (400 MHz, $CDCl_3$)
46

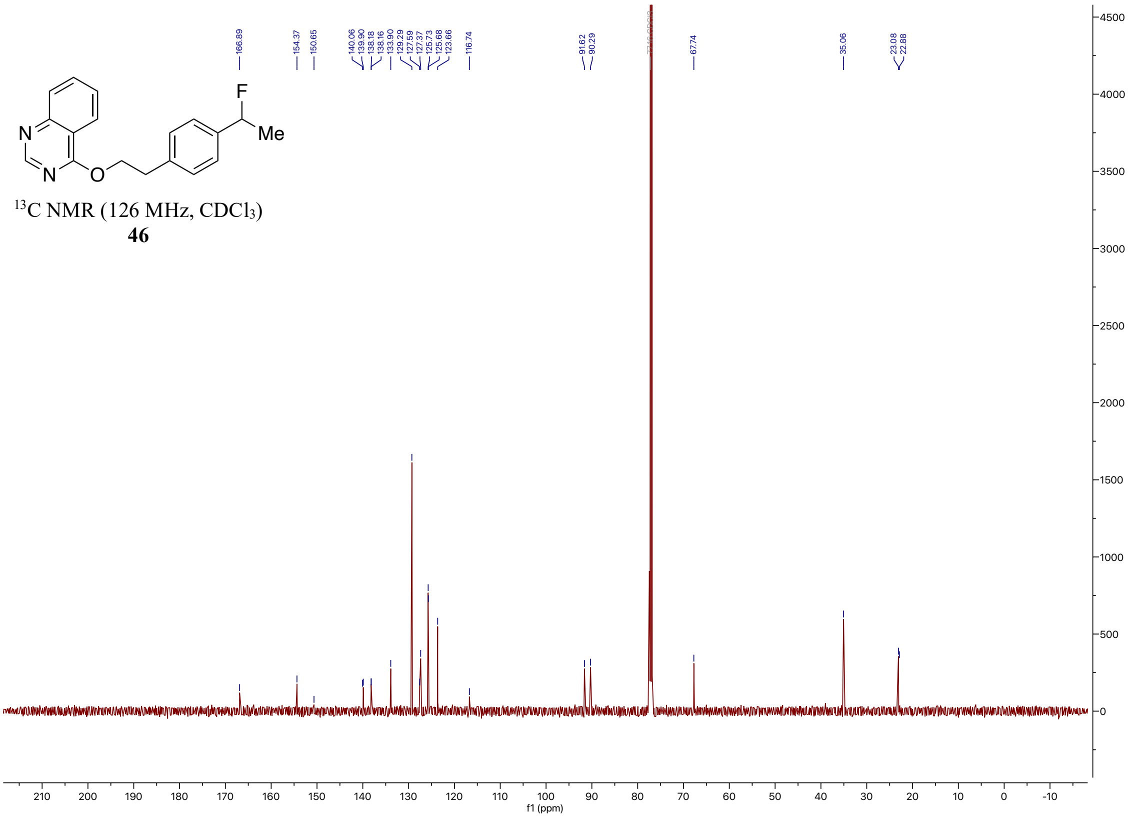

^{13}C NMR (126 MHz, $CDCl_3$)
46

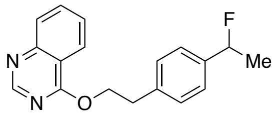

^{19}F NMR (376 MHz, $CDCl_3$)

46

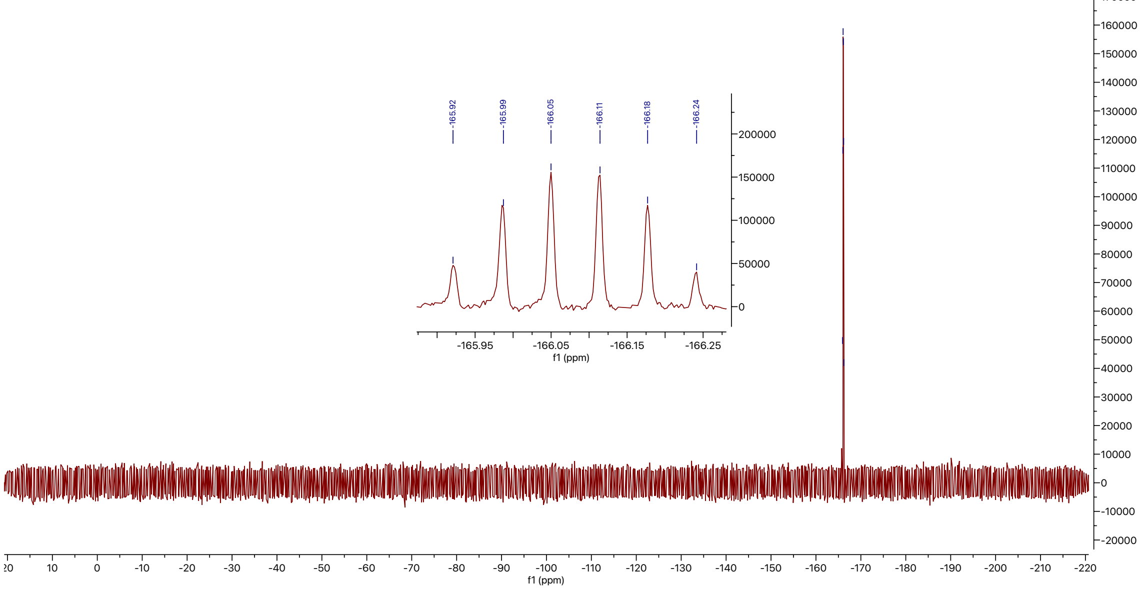

Supplement: Supplementary file 1 — ja4c02474_si_001.pdf [file ja4c02474_si_001.pdf]
